# Recommended Approaches to the Scientific Evaluation of Ecotoxicological Hazards and Risks of Endocrine-Active Substances

**DOI:** 10.1002/ieam.1885

**Published:** 2017-01-27

**Authors:** Peter Matthiessen, Gerald T Ankley, Ronald C Biever, Poul Bjerregaard, Christopher Borgert, Kristin Brugger, Amy Blankinship, Janice Chambers, Katherine K Coady, Lisa Constantine, Zhichao Dang, Nancy D Denslow, David A Dreier, Steve Dungey, L Earl Gray, Melanie Gross, Patrick D Guiney, Markus Hecker, Henrik Holbech, Taisen Iguchi, Sarah Kadlec, Natalie K Karouna-Renier, Ioanna Katsiadaki, Yukio Kawashima, Werner Kloas, Henry Krueger, Anu Kumar, Laurent Lagadic, Annegaaike Leopold, Steven L Levine, Gerd Maack, Sue Marty, James Meado, Ellen Mihaich, Jenny Odum, Lisa Ortego, Joanne Parrott, Daniel Pickford, Mike Roberts, Christoph Schaefers, Tamar Schwarz, Keith Solomon, Tim Verslycke, Lennart Weltje, James R Wheeler, Mike Williams, Jeffrey C Wolf, Kunihiko Yamazaki

**Affiliations:** 1 independent Consultant, Dolfan Barn, Beulah, Llanwrtyd Wells, Powys, United Kingdom; 2 US Environmental Protection Agency, Duluth, Minnesota; 3 Smithers Viscient Laboratories, Wareham, Massachusetts, USA; 4 Department of Biology, University of Southern Denmark, Odense M, Denmark; 5 Applied Pharmacology and Toxicology, Gainesville, Florida, USA; Dept Physiol Sciences, CEHT, Univ of Florida College of Veterinary Medicine, Gainesville, Florida, USA; 6 DuPont Crop Protection, Stine-Haskell Research Center, Newark, New Jersey, USA; 7 Office of Pesticide Programs, United States Environmental Protection Agency, Washington DC; 8 College of Veterinary Medicine, Mississippi State University, Mississippi, USA; 9 The Dow Chemical Company, Toxicology and Environmental Research and Consulting, Midland, Michigan, USA; 10 Pfizer, Groton, Connecticut, USA; 11 RIVM, Bilthoven, The Netherlands; 12 Center for Environmental and Human Toxicology, Department of Physiological Sciences, College of Veterinary Medicine, University of Florida, Gainesville, Florida, USA; 13 Environment Agency, Wallingford, Oxfordshire, United Kingdom; 14 US Environmental Agency, Reproductive Toxicology Branch, Research Triangle Park, North Carolina; 15 wca, Volunteer Way, Faringdon, United Kingdom; 16 Molecular & Environmental Toxicology Center, University of Wisconsin, Madison, Wisconsin, USA; 17 Toxicology Centre and School of the Environment & Sustainability, University of Saskatchewan, Saskatoon, Saskatchewan, Canada; 18 National Institute for Basic Biology, Myodaiji, Okazaki, Japan; 19 University of Minnesota, Integrated Biosciences Graduate Program, Duluth, Minnesota, USA; 20 US Geological Survey Patuxent Wildlife Research Center, Beltsville, Maryland; 21 Centre for Environment Fisheries and Aquaculture Science (Cefas), Weymouth, Dorset, United Kingdom; 22 Japan NUS Co, Shinjuku-Ku, Tokyo, Japan; 23 Leibniz Institute of Freshwater Ecology and Inland Fisheries, Berlin, Germany; 24 Wildlife International, Easton, Maryland, USA; 25 CSIRO, Glen Osmond, South Australia, Australia; 26 Bayer AG, Crop Science Division, Environmental Safety, Ecotoxicology, Monheim am Rhein, Germany; 27 Caldris Environment BV, Warnsveld, The Netherlands; 28 Global Regulatory Sciences, Monsanto Company, St Louis, Missouri, USA; 29 German Environment Agency (UBA), Dessau-Roßlau, Germany; 30 Dow Chemical Company, Midland, Michigan, USA; 31 Ecotoxicology and Environmental Fish Health Program, Northwest Fisheries Science Center, NOAA, Seattle, Washington, USA; 32 Environmental and Regulatory Resources, Durham, North Carolina, USA; 33 Regulatory Science Associates, Binley Business Park, Coventry, United Kingdom; 34 Bayer CropScience, Research Triangle Park, North Carolina, USA; 35 Environment and Climate Change Canada, Water Science and Technology Directorate, Burlington, Ontario, Canada; 36 Syngenta, Jealotts Hill International Research Centre, Bracknell, United Kingdom; 37 Independent Consultant, Burnham-on-Crouch, Essex, United Kingdom; 38 Fraunhofer IME, Applied Ecology, Schmallenberg, Germany; 39 Centre for Toxicology, School of Environmental Sciences, University of Guelph, Ontario, Canada; 40 Gradient, Cambridge, Massachusetts, USA; 41 BASF SE, Ecotoxicology, Rheinland-Pfalz, Germany; 42 Dow AgroSciences, Abingdon, Oxfordshire, United Kingdom; 43 CSIRO Land and Water, Waite Campus, SA, Australia; 44 Experimental Pathology Laboratories, Sterling, Virginia, USA; 45 Department of Environmental Health, Ministry of the Environment, Tokyo, Japan

**Keywords:** Endocrine disruptors, Ecotoxicological hazard assessment, Ecotoxicological risk assessment

## Abstract

A SETAC Pellston Workshop® “Environmental Hazard and Risk Assessment Approaches for Endocrine-Active Substances (EHRA)” was held in February 2016 in Pensacola, Florida, USA. The primary objective of the workshop was to provide advice, based on current scientific understanding, to regulators and policy makers; the aim being to make considered, informed decisions on whether to select an ecotoxicological hazard- or a risk-based approach for regulating a given endocrinedisrupting substance (EDS) under review. The workshop additionally considered recent developments in the identification of EDS. Case studies were undertaken on 6 endocrine-active substances (EAS—not necessarily proven EDS, but substances known to interact directly with the endocrine system) that are representative of a range of perturbations of the endocrine system and considered to be data rich in relevant information at multiple biological levels of organization for 1 or more ecologically relevant taxa. The substances selected were 17α-ethinylestradiol, perchlorate, propiconazole, 17β-trenbolone, tributyltin, and vinclozolin. The 6 case studies were not comprehensive safety evaluations but provided foundations for clarifying key issues and procedures that should be considered when assessing the ecotoxicological hazards and risks of EAS and EDS. The workshop also highlighted areas of scientific uncertainty, and made specific recommendations for research and methods-development to resolve some of the identified issues. The present paper provides broad guidance for scientists in regulatory authorities, industry, and academia on issues likely to arise during the ecotoxicological hazard and risk assessment of EAS and EDS. The primary conclusion of this paper, and of the SETAC Pellston Workshop on which it is based, is that if data on environmental exposure, effects on sensitive species and life-stages, delayed effects, and effects at low concentrations are robust, initiating environmental risk assessment of EDS is scientifically sound and sufficiently reliable and protective of the environment. In the absence of such data, assessment on the basis of hazard is scientifically justified until such time as relevant new information is available.

## INTRODUCTION AND BACKGROUND

The purpose of the present consensus paper is to provide scientific information on current best practices in the evaluation of hazards and risks to wildlife populations of endocrine-active substances (EAS) and endocrine-disrupting substances (EDS), developed using a cross-section of international expertise. There have been many descriptions of environmental EDS and their effects, including those of the World Health Organization and International Programme on Chemical Safety (WHO/IPCS 2002) and the WHO and United Nations Environment Programme (WHO/ UNEP 2012), and it is well established that some EDS are, or have been, present in the environment at concentrations harmful to wildlife populations (e.g., Jobling et al. 2006; Matthiessen 2013).

Although other definitions have also been proposed (e.g., Kavlock et al. 1996; EC 1997; Zoeller et al. 2012; Weltje et al. 2013), the broad WHO definition of an EDS has been most widely adopted, and is used herein:

“An endocrine disruptor is an exogenous substance or mixture that alters function(s) of the endocrine system and consequently causes adverse health effects in an intact organism, or its progeny, or (sub) populations” [emphasis added] (WHO/IPCS 2002).

In contrast to EDS (which generally can only be identified by definitive dose-response studies), an EAS is any substance able to interact with an endocrine system to cause responses that may or may not give rise to adverse effects (see Glossary for full definitions of terms used). An EAS may therefore be identified using screening-level information.

In response to concerns about the ecotoxicological effects of EDS, individual countries and international governments and organizations, including Japan, the United States of America (USA), the European Union (EU), and the Organisation for Economic Co-operation and Development (OECD) have, over the past 20 y, initiated programs for assessing potential impacts of EAS to wildlife (as well as human health) (Coady et al. this issue). In most jurisdictions, the goal of regulation is to prevent adverse effects on wildlife populations rather than on individuals. This goal has led to many discussions about how to conduct risk assessments of these substances, or even whether this is appropriate (Zoeller et al. 2015; Coady et al. 2016). In other words, should some or all EDS be treated as persistent organic pollutants (POPs); persistent, bioaccumulative, and toxic (PBT) substances; or genotoxic carcinogens, for which it is presumed a risk exists if exposure, no matter how small, occurs?

Several jurisdictions have initiated regulatory approaches to EDS, but these have varied, partly because until now there has been little consensus about some key scientific questions. For example, some scientists believe that EDS can be reliably assessed using the standard risk assessment paradigm (i.e., comparison of predicted environmental concentrations with predicted no-effect concentrations [PNECs]), whereas others do not believe this is sufficiently precautionary and propose risk management on the basis of hazard alone (i.e., regulation based solely on endocrine-disrupting properties) (Endocrine Society 2009, 2015). Regulation by hazard has been championed for the following reasons:

- the occasional occurrence of nonmonotonic dose- concentration responses,
- the possible absence of thresholds of effects in some instances,
- concerns for possible insensitivity of current toxicological and ecotoxicological tests to detect certain types of endocrine system perturbation, and
- the possibility that short-term exposures to EDS may lead to long-term (i.e., latent) consequences not addressed during testing.

It has been suggested that these factors prevent the confident prediction of no-effect doses or concentrations (ED EAG 2013; EFSA 2013), although this point is controversial.

A key question is “How are regulators and policy makers to decide whether to select a hazard or a risk-based approach for a given EDS under review?” Some (inter)governmental guidance already is available on evaluation of the (eco)toxicological properties of potential endocrine disruptors (e.g., DK EPA 2011; USEPA 2011a; OECD 2012c) but, to date, it has been unclear how or whether this information can be used to derive acceptable environmental exposures, that is, assessment of risk. There is a clear need for objective advice, based on the current level of scientific understanding, to allow regulators and policy makers to make comprehensive, science-based decisions.

The paper was a product of the SETAC Pellston Workshop^®^ “Environmental Hazard and Risk Assessment Approaches for Endocrine-Active Substances (EHRA),” held 31 January to 5 February 2016 in Pensacola, Florida, USA, with the participation of 48 invited international experts from 9 countries, as authors of the present paper. Backgrounds of the participants were varied, with 27% of the participants from government, 27% from academia, 21% from industry, and 25% attending as independent consultants. In addition to the present paper, 4 companion papers, based on insights gained from case studies of specific EAS, are being published simultaneously as output of this workshop.

With expert contributions from industry, government, and academia, the SETAC Pellston Workshop developed consensus-based advice on scientifically defensible approaches for the assessment of EDS. The present paper outlines the circumstances in which risk assessment of an environmental EDS may be acceptable and those in which a hazard-only approach is warranted. The paper is primarily aimed at scientists and hazard or risk assessors responsible for the development and regulation of chemicals, whether in industry, government, or academia, and it provides guidance on scientifically justifiable assessment procedures. It also highlights areas of scientific uncertainty and presents recommendations for research to address these issues. Regulators and others are invited to take note of the paper’s recommendations when drafting their own guidance for evaluating EAS and EDS.

## METHODS

To facilitate the identification of key factors when evaluating EAS and EDS, 6 substances for case studies were chosen as representative of a range of endocrine modes of action:

- 17α-ethinylestradiol (EE2),
- perchlorate,
- propiconazole,
- 17β-trenbolone,
- tributyltin (TBT), and
- vinclozolin.

(Supplemental Data S1 through S6 present the case study summaries and literature selected; S7 gives the methods used to perform the case studies).

The substances for the case studies were selected so that they covered a range of endocrine pathways or actions of concern (estrogen agonism, thyroid antagonism, steroidogenesis inhibition, androgen agonism, retinoid receptor modulation, and androgen antagonism, respectively). In all cases, these chemicals were considered to be data rich in relevant information for 1 or more ecologically relevant taxa at multiple levels of biological organization from the biochemical to the whole organism and, sometimes, the population. However, it is important to note that the case studies are not comprehensive safety evaluations but, rather, provided the foundations for examining the key issues and procedures discussed in this paper.

The case study groups conducted hazard and risk assessments making use of published guidance. The guidance used and full details of the case study assessments can be found in the Supplemental Data S1. Each group followed a similar process but with differences according to the information available. The general work flow for the case studies is summarized in Supplemental Data Figure S7–1.

All groups conducted searches of the published literature, openly accessible regulatory datasets, and other sources such as test guideline validation studies and high throughput in-vitro assays (ToxCast™; USEPA 2016a). Literature studies were first assessed for relevance, and then evaluated for reliability using the Toxicological Data Reliability Assessment Tool (ToxRTool) (Schneider et al. 2009) and/or Klimisch criteria (Klimisch et al.1997).

A potential shortcoming identified prior to the workshop was the lack of reliable assessment data for histopathology endpoints and an inability to access studies submitted in confidence to regulatory agencies. Evaluation of histopathology data requires specialized expertise, and despite the frequent occurrence and integral role of this evaluation among the reviewed studies, it was recognized that this subject is not addressed specifically within either the Klimisch or ToxRTool frameworks. Consequently, histopathology data were assessed for reliability in a parallel exercise, the results of which were incorporated into the case study evaluations.

Studies were then assembled in a framework in order to collate data on effects relevant for assessing the endocrine axes. In most cases (vinclozolin, trenbolone, TBT, EE2, and perchlorate), the levels of assessment established by the OECD Framework (CF) for the Testing and Assessment of Endocrine Disruptors (OECD 2012b) were used as a guide. Each group examined the available data to determine whether their substance exhibited the potential for interaction with a specific pathway in vitro or in vivo and exhibited adverse effects potentially mediated by that pathway. Adverse effects observed in higher-tier tests were queried to determine whether they were corroborated by lower-tier tests, and whether they could be concluded to be a consequence of endocrine activity.

The groups used weight-of-evidence (WoE) assessments of various types to determine interaction with endocrine systems and potential associations with adverse effects. The propiconazole and perchlorate groups used a system similar to that of the US Environmental Protection Agency (USEPA 2011a) in which the responses (positive, negative, or no change) of each relevant endpoint were tabulated and organized according to interaction with endocrine axes. The propiconazole group explicitly used the hypothesis-testing methods recommended by Becker et al. (2015). The TBT, trenbolone, and EE2 groups used adverse outcome pathways (AOP) (Ankley et al. 2010) to structure the WoE process.

Adverse outcome pathways are designed to depict causal linkages between a specific endocrine activity or molecular initiating event (MIE) such as receptor activation or inhibition, and adverse apical outcomes (e.g., reduced fecundity, altered sex ratios). Finally, following the hazard characterization, exposure estimates were generally incorporated in order to assess possible differences in hazard- versus riskbased decisions.

The results of the case studies then provided many examples of crosscutting, data availability, and interpretation issues, typically common to several substances, which may have an impact on decision making. These are shown in detail in the case study Supplemental Data and have been used to design the suggested strategy (see Section Proposed Decision-Making Strategy to Support Endocrine Disruptor Ecotoxicological Hazard VERSUS Risk Assessment) for deciding on whether a sound risk assessment of a particular EDS can be undertaken.

## CROSSCUTTING ISSUES RELEVANT TO THE EVALUATION OF HAZARDS AND RISKS OF EAS AND EDS

As case study groups conducted their analyses of the 6 EAS, a series of crosscutting issues with relevance to the hazard and risk assessment of EAS and EDS were identified. By “crosscutting issues,” we mean problems of evaluation that were common to several of the case studies. Some of these are discussed with respect to their application in an improved ecotoxicological hazard and risk assessment. A number of issues were also identified that play a role in determining whether an EDS can confidently be subjected to ecotoxicological risk assessment, or whether regulation by hazard is the most appropriate option. Finally, issues were identified that aid in distinguishing between endocrineversus nonendocrine-specific responses. These crosscutting issues are broadly outlined below and discussed in detail in the associated companion papers (Coady et al. this issue; Marty et al. this issue; Mihaich et al. this issue; Parrott et al. this issue).

### Challenges in assigning endocrine-specific modes of action

A major challenge in the assessment of EAS is understanding the primary mechanism of action, in the context of perturbation of an endocrine target of concern (i.e., the MIE). Whereas identifying the mechanistic basis for how a substance acts is not necessarily a requirement for performing a traditional risk assessment, the ability to distinguish between endocrine and nonendocrine-mediated responses is necessary when specific regulatory outcomes are tied to assigning causality between perturbation of a specific pathway and an adverse effect. Thus, there is a need for careful study design and data interpretation to distinguish between endocrine versus nonendocrine-specific responses. The WHO IPCS definition of an endocrine disruptor is broad, and a very precautionary interpretation might capture many mechanisms that, in general, would not specifically be considered to be endocrine disruption (Dang 2016; Wheeler and Coady 2016). For instance, hepatotoxicity can potentially cause decreased levels of vitellogenin in female fish (Miller et al. 1999), leading to reproductive failure, an effect analogous to some chemical effects on estrogen, androgen, and thyroid (EAT) pathways.

The likelihood of indirect effects is increased in (eco) toxicological studies requiring the use of maximum tolerated dose or concentration test levels, which must produce some adverse effects (Wheeler et al. 2013; Witorsch 2016). The OECD CF levels 4 and 5, which cover aquatic tests with apical endpoints, recommend a maximum test concentration of 1/10th of the acute LC50, or range-finding studies to avoid overt toxicity (see OECD TG 234, 240, 241; in OECD 2017; Wheeler et al. 2013), which decrease the likelihood of indirect effects (i.e., apparent endocrine responses caused by interactions mediated via nonendocrine mechanisms). The misidentification of endocrine disruption as a direct cause of effects where it is actually an indirect cause has serious consequences in terms of triggering animal and resource-intensive testing and potentially severe regulatory outcomes. A WoE approach, similar to that used by Becker et al. (2015), can be used to explore endocrine-specific modes of action. This approach is based on biological plausibility, empirical support, and essentiality of key events in an AOP. It has been used to evaluate diagnostic (endocrine-specific and nonendocrine mechanisms) and apical endpoints to investigate whether an endocrine mechanism can be conclusively assigned to the effects observed for a given substance. The use of an AOP approach to assemble the lines of evidence that lead to an adverse effect helps to put into context the various mechanisms that may be responsible. This approach was used to examine 3 of the case study substances, EE2, propiconazole, and 17β-trenbolone (Supplemental Data S1, S3, and S5; Mihaich et al. this issue).

### Uncertainties in biological responses that influence hazard and risk approaches to the regulation of EAS and EDS

Endocrine-disrupting substances may have certain biological effects, including delayed or multigenerational impacts (i.e., latent effects), or they may display nonmonotonic dose- response relationships (NMDRs) experimentally that require careful consideration when determining ecotoxicological hazard or risk. This topic is addressed in detail in a companion paper (Parrott et al. this issue). For example, EDS can have specific and profound effects when exposure occurs during sensitive windows of the life cycle. This exposure creates the potential for delayed responses where the actual adverse effect is manifest at life stages different from those during which exposure occurred. An example is sex reversal in fish, if exposure to certain EDS occurs over the period of sexual differentiation (e.g., McAllister and Kime 2003), where the actual adverse population-relevant effect is not manifested until the fish reach sexual maturity with consequent impaired reproductive capacities (Nash et al. 2004). This underscores the need for testing during appropriate (sensitive) life stages and, when necessary, full life cycle designs that are intended to capture adverse effects where and whenever they occur. The potential for effects to be manifest in subsequent generations (multigenerational effects) also has been raised as a potential issue in the derivation of appropriate endpoints for EDS. Concern for this potential is reflected in the design of the new higher-tier tests to assess EAS developed under the auspices of the OECD and USEPA, which are moving toward extended 1-generation designs for fish (OECD TG 240) and mammals (OECD TG 443 in OECD 2017).

It has been hypothesized that the occurrence of NMDRs is also an uncertainty for reliable risk assessment of EDS. Substantial data reviews are underway to inform on their occurrence and relevance (e.g., EFSA external report http://www.efsa.europa.eu/en/supporting/pub/1027e). However, at this time evidence indicates that NMDRs may be most prevalent in in-vitro tests (e.g., due to cell toxicity; Berckmans et al. 2007; Dang 2009; Lagarde et al. 2015) and in in-vivo mechanistic studies (e.g., due to feedback-mediated compensatory responses [Ankley and Villeneuve 2015]), and not generally translated to adverse apical endpoints that would be employed in risk assessment, although such examples have been documented (Örn et al. 2003). Others have provided guidance for characterizing NMDRs (Lagarde et al. 2015), and a flowchart of how to evaluate NMDRs in the context of endocrine hazard and risk assessment procedures is presented in a companion paper (Parrott et al. this issue).

Overall we can conclude that, if careful consideration of delayed, multigenerational (i.e., latent), and NMDR effects is made, it is feasible to assess ecotoxicological endocrine hazards and derive robust endpoints for risk assessment procedures ensuring a high level of environmental protection. It should, however, be noted that these types of data are currently available for relatively few chemicals.

### Improved methods for the assessment of EAS and EDS

To assess either hazards or risks of possible EAS or EDS requires robust, validated test methods that detect perturbation of endocrine pathways of concern, provide insights as to potential adverse apical effects, and offer information on the concentrations at which these effects occur. Further, the assays should be capable of generating necessary information in a timely and cost-effective manner that minimizes, as much as possible, use of test animals. A number of in-vivo test systems have been developed and are available for the assessment of EAS or EDS in different regulatory settings. However, there are several inherent limitations to the collection and interpretation of data from these assays, which are addressed in detail in a companion paper (Coady et al. this issue).

One issue of significant concern to current EAS screening and testing programs involves resources in terms of cost, time, personnel, and animal use. This issue is especially problematic when considering the number of chemicals that some regulatory authorities need to assess; for example, the Endocrine Disruptor Screening Program (EDSP) in the United States has been charged with considering potential endocrine-mediated effects of around 10000 chemicals, a task that clearly cannot be achieved solely with in-vivo tests (USEPA 2011b). One way to address this challenge is to prioritize chemicals for possible in-vivo testing using in-vitro high-throughput (HTP) assays focused on a suite of MIEs of concern. An example of how this type of approach could be used was recently described for estrogen receptor activation in mammals (USEPA 2014). Consideration of additional endocrine MIEs of concern, and expansion of the prioritization strategy to consider non-mammalian species, based on concepts of pathway conservation, is a technically reasonable prospect (Ankley et al. 2016; Coady et al. this issue).

One challenge associated with the design and conduct of in-vivo EAS screening and testing is the selection of appropriate (i.e., sensitive) species, endpoints, and life stages. A component of this involves the experience gained from existing tests to determine, for example, particular assays that may be exceptionally sensitive to perturbation of a given MIE of concern (e.g., Ankley and Gray 2013). In addition, though, there is promise for the strategic use of HTP data and/or early screening-level information (based, e.g., on computational models) to help guide the selection of existing assays that are most likely to be sensitive to a given EAS modality. For example, Coady et al. (this issue) show how HTP data for 17ß-trenbolone would help subsequent in-vivo testing to focus on assays that measure vertebrate reproduction and sexual development.

Additional challenges for EAS in-vivo screening and testing involve guidance for, and optimization of, a number of pragmatic issues inherent to the conduct of in-vivo (and occasionally in-vitro) assays, such as concentration setting, statistical power and sensitivity, delivery and analytical measurement of test substances, availability of technical expertise, and study interpretation, including the linking of mechanistic and apical effects. Coady et al. (this issue) address these challenges and offer several potential solutions, where applicable. Finally, a number of recommendations are provided for longer-term research efforts to address areas of uncertainty, including the need for a better understanding of the endocrine system of invertebrates, followed by the development of assays in potentially sensitive species (including invertebrates) for which (endocrine) test methods currently do not exist. One area of uncertainty is the role of key endocrine pathways in addition to EAT signaling (e.g., glucocorticoid, progesterone, and retinoid pathways) and an understanding of the relationship of perturbations in these pathways to population-relevant effects.

### Population-relevant endpoints in the evaluation of EAS for ecological hazard and risk assessment

Many endpoints (from subcellular through intact organism individual-level changes) have been used to evaluate endocrine mechanisms and effects in different taxa, but the link between these endpoints and population-level effects is often undefined (Kramer et al. 2011). This lack of definition is a source of major uncertainty for both hazard and risk assessment. The companion paper by Marty et al. (this issue) used data from the EAS case studies (Supplemental Data S1–S6) to evaluate the population relevance of collected study endpoint data in the context of ecotoxicological hazard and risk assessment for various taxa (invertebrates, fish, amphibians, birds, and mammals).

Population-relevant endpoints generally include effects at the individual level on fitness (i.e., behavior, growth and development, reproduction, and survival). Examples of such effects are described by Marty et al. (this issue). The development of new methodologies, including AOPs and population modeling, will foster a more complete understanding of the relationship between endocrine perturbations at lower levels of biological organization and adverse population-level effects. These methods may allow quantitative inferences about population-relevant effects from physiological changes (e.g., dynamic energy budgets: Martin et al. 2012), and predictive system models also show promise (Forbes and Calow 2012; Watanabe et al. 2016). However, until an established linkage between these endpoints and subsequent population changes are evident, such endpoints should not be used to drive the risk assessment of EDS. Marty et al. (this issue) have, in addition, examined recovery in endpoint responses, which may be particularly important when evaluating effects of EDS on populations.

## PROPOSED DECISION-MAKING STRATEGY TO SUPPORT ENDOCRINE DISRUPTOR ECOTOXICOLOGICAL HAZARD VERSUS RISK ASSESSMENT

Methods for identifying EAS and EDS have been available for some time (see Supplemental Data). The main area where guidance is lacking concerns the decision to subject these substances to ecotoxicological risk as opposed to hazard assessment. This problem is particularly relevant for datapoor substances for which few species or endpoints will have been studied. A number of potential questions that address the reliability of ecotoxicological risk assessment have been identified:

- Is exposure of wildlife probable?
- Is prediction (or measurement) of exposure reliable?
- Have the most appropriate taxa been tested (with relevant endpoints)?
- Have sensitive life stages, or the entire life cycle, been tested (again with relevant endpoints)?
- Have delayed and multigenerational (i.e., latent) effects been considered?
- Do NMDRs or other unusual temporal patterns of toxicity affect the ability to predict reliable no-adverse-effect levels?
- Does a threshold for adverse endocrine-mediated effects exist?

Each of these issues has the potential to make an ecotoxicological risk assessment uncertain. It might be argued that most of the uncertainties could be addressed through the use of additional assessment factors (AF), otherwise known as “uncertainty factors.” In general, their use may be acceptable but should be justified by reference to the available data and any relevant regulatory guidance. It is also possible that uncertainty can be reduced by using tools such as ToxCast (USEPA 2016a) to identify potential endocrine activities, by reading across from data derived from substances that share the same AOP (e.g., Becker et al. 2015), or by obtaining additional test data.

**Figure 1.**
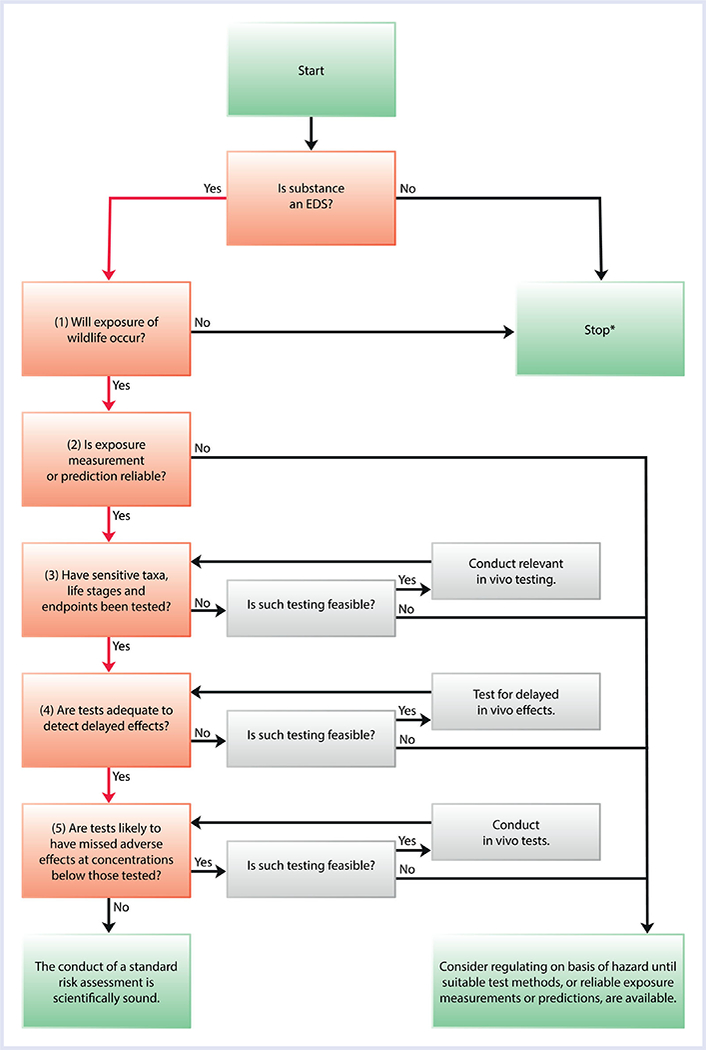
A suggested decision-making strategy for assessing whether a scientifically sound risk assessment of an EDS can bereliably performed. *On exiting at Stop, consider whether a risk assessment of non-EDS hazards is required. This of course applies only if wildlife exposure is expected to occur. EDS = endocrine- disrupting substance.

A possible strategy for addressing some of the questions listed above is shown in Figure 1. It should be noted that the flow chart is intended for use in situations where a substance has already been clearly identified as an EDS, and therefore decisions about whether or not to initiate ecotoxicological risk assessment need to be made. The issues underpinning these decisions have been discussed more fully in the section entitled Cross-Cutting Issues Relevant to the Evaluation of Hazards and Risks of EAS/EDS, and at length in the accompanying papers (Coady et al. this issue; Marty et al. this issue; Mihaich et al. this issue; Parrott et al. this issue).

The first substantive question (Figure 1, Question 1) is whether exposure to wildlife will occur. Under certain situations, this question can be excluded if exposure is limited to closed systems such as greenhouses, with no or extremely limited routes to the wider environment. In such cases, neither an ecotoxicological hazard nor risk assessment is required.

The second question (Figure 1, Question 2) is whether exposure measurement or prediction can be conducted reliably across compartments within ecosystems. There may be many reasons for difficulties with this question, but perhaps the most important concerns very potent substances, such as EE2, that may be active below their limit of quantitation in water or food items. Difficulties also arise for substances with some similarities to the POPs, such as methyl Hg, whose persistence, long-range transport, and bioaccumulation potential lead to their global distribution and biomagnification in food chains at sites remote from their point of use. Prediction of exposure may also be difficult or impossible for some chemicals that enter the environment by poorly understood routes or in unknown quantities. Guidance to suitable exposure prediction methodologies can be found in the European Chemicals Agency (ECHA 2016a, 2016b), FOCUS (2016), JRC (2016), and USEPA (2016b). Inability to measure or predict ambient concentrations of an EDS (or indeed any other substance) precludes the use of toxicity data in risk assessment, and for purposes of regulation it would then be assumed that exposure to levels sufficient to produce adverse effects could occur.

If exposure measurement or prediction is deemed sufficiently reliable, the next question (Figure 1, Question 3) is whether the responses of a relevant taxon, life stage, and endpoints have been adequately assessed. Even quite closely related species can vary considerably in their sensitivity to EDS. For example, in a whole-lake experiment with EE2, some short-lived fish species failed to reproduce, whereas others were apparently unaffected (see Supplemental Data S1 EE2; Palace et al. 2009). Similar issues arise for other EDS and species; for example, 17β-trenbolone causes androgenic effects in a variety of species, but with greatly varying potency (see Supplemental Data S5 Trenbolone). This issue underscores the importance of tiered and intelligent testing strategies that identify the relevant receptors and perform the most extensive testing and assessments of those. This uncertainty can be addressed by testing additional species that share responsiveness to a common signaling pathway, read-across from related chemicals, and knowledge about the degree of cross-species conservation of relevant endocrine MIEs and AOPs, to make judgments about whether sensitive species are likely to have been tested (Coady et al. this issue).

There can also be a wide range of sensitivity of different life stages within a species, with the window of greatest sensitivity to EAT substances often occurring during early sexual development (see EE2 and trenbolone SI). For this reason, datasets that lack information derived from exposure of developing organisms should be treated with caution.

If the sensitivity of test organisms has been adequately addressed, it becomes necessary to deal with the potential for delayed and multigenerational effects (Figure 1, Question 4). These effects may be of particular concern if sensitive developmental stages have been exposed but not followed through to maturity or into the next generation. In some cases where sufficient information concerning perturbation of a given endocrine pathway is known, study of delayed effects may not be necessary if the appropriate sensitive life-stage has been covered (in line with intelligent testing strategies). A good example of delayed effects concerns alterations of phenotypic sex ratios in juvenile and adult fish exposed as fry to EDS such as estrogens and androgens (see case studies and tables for Supplemental Data S1 for EE2 and S5 for trenbolone). Although data on multigenerational effects are still scarce (and exceptions to the following statements do exist, e.g., Chen et al. [2015]), information from the case studies suggests that fish from the second (F2) generation only rarely show greater sensitivity than the first (F1) generation during continuous exposure (Supplemental Data S1 and S3). Indeed, this is the basis of extended 1- generation test designs implemented for higher-tier testing of EAS (OECD TGs 240 and 443 - see OECD 2017). If there is sufficient information to suggest delayed or multigenerational effects, additional testing is likely needed if suitable methods such as life cycle tests are available.

When it has been concluded that delayed toxicity and possible multigenerational effects have been adequately accounted for, it becomes important to consider whether the substance possesses properties that might impair the ability to predict no-effect concentrations or doses (Figure 1, Question 5). In other words, has the dose- or concentration-response relationship been adequately described? Nonmonotonic dose-response relationships can occur in both in-vitro and, for some endpoints, short-term in-vivo studies with EDS. However, such response curves may not be broadly predictive of similar effects in long-term in-vivo studies with apical endpoints (see case studies and tables for Supplemental Data S4 for TBT, S1 for EE2, and S5 for trenbolone; USEPA 2013). A structured approach to tackling the NMDR issue for both mechanistic and apical endpoints is proposed in Lagarde et al. (2015) and Parrott et al. (this issue). The 2 NMDR flowcharts in Parrott et al. (this issue) consider aspects of reproducibility and biological plausibility, and whether a threshold can be determined. In summary, if an NMDR is observed and confirmed in an apical test with population relevance, further testing at lower concentrations and appropriate exposure times should be considered in order to establish a defensible no-effect concentration (NOEC) or ECx.

Regulation of EDS on the basis of hazard alone may be partly driven by a perception that these substances do not have a toxic threshold (Parrott et al. this issue). However, it is conceptually impossible to prove that toxic thresholds for EDS do not exist, and attempts to do so would involve the use of impractical, not to mention unreasonable, numbers of test organisms. Most significantly, a viable physiological basis for such absent thresholds has not been clearly identified. Furthermore, there is no evidence that such effects apply to populations of organisms, the defined protection goal of most global policies and regulations (with the exception of those aimed at protecting rare or endangered species). Thresholds of toxicity were present for all the case study substances (see Supplemental Data), and theoretical considerations suggest that endocrine systems could not function if such thresholds were absent (Borgert et al. 2013). However, as indicated in the section entitled Cross-Cutting Issues Relevant for the Evaluation of Hazards and Risks of EAS/EDS, the absence of thresholds may be truly applicable only to population-level effects because a small proportion of individuals may show background endocrine effects unrelated to EDS exposure. Probabilistic approaches to the identification of true thresholds show some promise (Hanson and Solomon 2002).

If the concerns articulated in this section are considered to have been satisfactorily addressed, then it is technically defensible to conduct an ecotoxicological risk assessment using specific exposure and dose-response data. Otherwise, the precautionary approach of deriving PNECs using AFs could be considered if further data generation cannot resolve outstanding issues. In the absence of adequate data or modeling results, assessment on the basis of hazard is scientifically justified until such time as relevant new information is available. It is important to bear in mind that although current internationally standardized tests are not diagnostic for non-EAT endocrine modalities, available methods such as life cycle tests probably detect the majority of apical effects regardless of whether endocrine or nonendocrine mechanisms are involved, and development of new test methods (e.g., OECD 2012a) will further expand our level of confidence that serious effects have not been missed.

## KNOWLEDGE GAPS IN ENDOCRINE SCREENING AND TESTING

Several areas where further research is needed were identified at the workshop, and the main points are highlighted here; for more detail, see the companion papers in this series (Coady et al. this issue; Marty et al. this issue; Mihaich et al. this issue; Parrott et al. this issue). Many of these areas involve the need for fundamental biological research, but there is also a need for the development of new testing methods.

### Consideration of additional endocrine pathways

There is a clear need to consider a wider range of endocrine pathways of concern; there are at least 48 different soluble nuclear receptors that bind with ligands to produce their actions, of which many are currently ignored. There also are more hormones than are analyzed at present. Consequently there is a need to develop a wider understanding of the ways in which endocrine pathways can be perturbed, and to produce implementable tools for their study.

### Test methods for under-represented taxa and pathways

There is a need for invertebrate tests with mechanistic endpoints in the context of chemical perturbations (MIEs, AOPs). For example, it would be desirable to develop a screening assay that evaluates the retinoid X receptor (RXR) pathway, which is important in mollusks as well as vertebrates (e.g., fish). However, most developments of this type will depend on improvements in our understanding of invertebrate endocrinology, particularly for nonarthropods. More screening assays are also needed for vertebrates, especially for some birds and reptiles with which apical studies cannot be readily conducted for logistical reasons.

Secondly, there are methodological gaps that affect the current EDS testing paradigm of progression from screening to apical testing. For example, there are standardized apical tests for mollusks, mysids, and birds, but screening tests are needed for these taxa so that triggers of such apical tests can be defined. These issues will need to be explored as additional higher-tier data are generated.

### Behavioral endpoints

Endocrine-disrupting substances are known to alter behavior by affecting the central nervous system (CNS) via endocrine-mediated mechanisms during intrauterine or neonatal life, puberty, or adulthood (e.g., Gray and Ostby 1998). Risk to populations from inappropriate or ill-timed courtship or parental behavior (e.g., migration, nesting, lactation) is as significant as the repercussions from disrupted ovulation or spermatogenesis. Consequently, there is a need to identify such additional, potentially sensitive behavioral endpoints in the context of endocrine perturbation, specifically for birds in which such endpoints are not sufficiently included in regulatory testing and to link these, on the one hand to MIEs and on the other hand to population-level effects.

### Determining adversity of effects

There is a need for more population-level predictive work for a representative range of organisms, in particular to determine

1. the extent to which delays in development or reduction in reproductive output constitute adverse effects at the individual and population levels;
2. whether the loss of age classes as a result of affected growth has an impact at the community and ecosystem levels;
3. the extent to which adaptation and recovery affect population-level impacts; and
4. the quantitative relationship among initiating events, key events, and adverse population-level effects.

### Species sensitivity and sensitive life stages or windows of exposure

More information is needed to ascertain how sensitivity to EDS varies with developmental stage. In addition, we need to determine whether short-lived species are more likely to be impacted by EDS, or whether it is simply easier to identify population-level effects within their shorter experimental timeframe (e.g., Palace et al. 2009).

### Predicting no-effect concentrations or toxic thresholds

Probabilistic methods for prediction of true thresholds have been proposed (Hanson and Solomon 2002). There is no reason to expect that these types of methods could not be used for EAS and EDS, but more research in this field is required, particularly on the mechanistic basis of issues such as NMDRs, etc.

## CONCLUSIONS AND RECOMMENDATIONS

As summarized herein, and in the accompanying papers, substantial guidance already is available on how to consider the hazardous properties of an EAS and support a decision on whether it is an EDS, using WoE approaches. However, the present paper also identifies additional issues that should be considered in hazard characterization and provides guidance on how they can be addressed.

Key questions that should be asked before a risk assessment is attempted include these:

- Is exposure of wildlife probable?
- Is prediction (or measurement) of exposure reliable?
- Have the most appropriate taxa and species been tested (with relevant endpoints)?
- Have sensitive life stages, or the entire life cycle, been tested (again with relevant endpoints)?
- Have delayed and multigenerational effects been considered?
- Can reliable no-adverse-effect levels be predicted, despite the possible presence of NMDRs or other unusual temporal patterns of toxicity?
- Does a threshold for adverse endocrine effects exist?

The primary conclusion of the present paper, and of the SETAC Pellston Workshop on which it is based, is that if responses to all of these questions are positive, it is scientifically defensible to proceed with a standard risk assessment.

If the response to any of these questions is negative (except in those specific cases identified) or equivocal, there may be an opportunity to address the uncertainty by further modeling or testing before it is considered scientifically sound to proceed to ecotoxicological risk assessment. However, if suitable test or modeling methods are unavailable, the only alternative may be to regulate the substance on the basis of hazard alone, at least until such time as relevant additional data become available.

## GLOSSARY AND DEFINITIONS

Meanings of terms might not always be the same in different regulatory jurisdictions or scientific disciplines. Table 1 therefore provides meanings of acronyms and terms as used in this and the accompanying papers.

## Supplementary Material

Supplement1 S1 — Case Study Summary EE2
S1 — EE2 Tables S1–7

Supplement5 S5 — Case Study Summary Trenbolone
S5 — Trenbolone Table S5–5
S5 — Trenbolone Table S5–6

Supplement6 S6 — Case Study Summary Vinclozolin
S6 — Vinclozolin — Table S6–5

Supplement7 S7 — Methods Used for Case Studies

Supplement8

Supplement9

Supplement10

Supplement11

Supplement12

Supplement13

Supplement14

Supplement2 S2 — Case Study Summary Perchlorate

Supplement3 S3 — Perchlorate Table S2–4

Supplement4 S4 — Case Study Summary Propiconazole
S4 — Case Study Summary TBT
S4 — TBT Table S4–1
S4 — TBT Table S4–2
S4 — TBT Table S4–3

## Figures and Tables

**Table 1.** Glossary of terms and acronyms used in the present paper and in the accompanying papers

Term or acronym	Definition^a^
Adverse effect	Change in the morphology, physiology, growth, development, reproduction, or life span of an organism, system, or (sub)population that results in an impairment of functional capacity, an impairment of the capacity to compensate for additional stress, or an increase in susceptibility to other influences.
AOP	Adverse outcome pathway
AF	Assessment factor
CNS	Central nervous system
EAS	Endocrine-active substance. A substance that can interact with an endocrine system to cause responses that may or may not give rise to adverse effects.
EAT	Estrogen, androgen, and thyroid pathways
ECx	Effect concentration x. A toxicant concentration causing effects in x% of a test population.
EDS	Endocrine-disrupting substance. An exogenous substance or mixture that alters functions of the endocrine system and consequently causes adverse health effects in an intact organism, or its progeny, or (sub)populations (WHO/ IPCS 2002).
Hazard	Inherent property of an agent or situation having the potential to cause adverse effects when an organism, system, or (sub)population is exposed to that agent.
Hazard assessment	A process designed to determine the possible adverse effects of an agent or situation to which an organism, system, or (sub)population could be exposed.
HTP assays	High-throughput assays
MIE	Molecular initiating event
NMDR relationships	Nonmonotonic dose-response relationships
NOEC	No-observed-effect concentration
PBT	Persistent, bioaccumulative, and toxic substances
PNEC	Predicted no-effect concentration
POPs	Persistent organic pollutants
Risk	The probability of an adverse effect in an organism, system, or (sub)population caused under specified circumstances by exposure to an agent.
Risk assessment	A process intended to calculate or estimate the risk to a given target organism, system, or (sub)population, including the identification of attendant uncertainties, following exposure to a particular agent, taking into account the inherent characteristics of the agent of concern as well as the characteristics of the specific target system and exposure.
Threshold	Dose or exposure concentration of an agent below which a stated effect is not observed or expected to occur.
WoE	Weight of evidence

a Some definitions adapted from IPCS (2004).

## EDITOR’S NOTE:

This is 1 of 5 articles generated from the SETAC Pellston Workshop “Ecotoxicological Hazard and Risk Assessment Approaches for Endocrine-Active Substances (EHRA)” (February 2016, Pensacola, Florida, USA). The primary aim of the workshop was to provide objective advice, based on current scientific understanding, to regulators and policy makers, whether in industry, government, or academia. The goal is to make considered, informed decisions on whether to select an ecotoxicological hazard- or risk-based approach for regulating a given endocrine disrupting substance under evaluation.
