# Supplementary material for: Recommended Approaches to the Scientific Evaluation of Ecotoxicological Hazards and Risks of Endocrine-Active Substances
Source: Integr Environ Assess Manag. Author manuscript; Available in PMC 2018 Aug 1. (PMC6069525; doi:10.1002/ieam.1885)
Supplement: Supplement1 — S1 — Case Study Summary EE2 S1 — EE2 Tables S1–7 [file NIHMS1500348-supplement-Supplement1.docx]

**Supplemental Data S1**

**Draft Case Study for the Ecotoxicological Hazard and Risk Evaluation of EE2**

Joanne L. Parrott†, Poul Bjerregaard‡, Kristin E. Brugger§, Taisen Iguchi#, Sarah M. Kadlec††, and Lennart Weltje‡‡.

† Environment and Climate Change Canada, 867 Lakeshore Road, Burlington, ON, L7S 1A1, Canada; phone 905 336 4551, fax 905 336 6430, Email [joanne.parrott@canada.ca](mailto:joanne.parrott@canada.ca)

‡ Department of Biology, University of Southern Denmark, Odense, Denmark

§ DuPont Crop Protection, Stine-Haskell Research Center, Newark, USA

# Department of Bioenvironmental Research, Okazaki Institute for Integrative Bioscience, National Institute for Basic Biology, National Institutes of Natural Sciences, Okazaki, Japan

††University of Minnesota, Integrated Biosciences Graduate Program, Duluth, Minnesota, USA

‡‡BASF SE, Crop Protection – Ecotoxicology, Limburgerhof, Germany

1. Overview of the Purpose of the case study
   1. General text on hazard and risk assessment of endocrine active substances to be provided by the synthesis group to all case study groups.
   2. Include some of the Level 1 of OECD Conceptual Framework information

17α-Ethinylestradiol (EE2, CAS number 57-63-6) is a synthetic steroid estrogen derived from the naturally occurring estrogen 17β-estradiol (E2). The following information is derived from the European EQS dossier on ethinylestradiol 2011 (Water-Framework-Directive 2011), unless otherwise noted.

Uses

The most common use for EE2 is in the estrogenic component of oral contraceptives at 20-50 µg per dose (Endrikat et al. 1997), where its function is to inhibit ovulation via estrogen receptor (ER) agonism. It is also indicated for pharmaceutical treatment of menopausal and post-menopausal symptoms and other hormonal disorders.

Physical and chemical properties

Its water solubility is reported as 4.7 - 19 mg/L at 20°C. The calculated vapor pressure of 1.5 x 10^-7^ Pa at 25°C indicates low volatility. The octanol-water partition coefficient (Kow) reported as log Kow = 3.67 - 4.2 indicates that it is lipophilic. The bioconcentration factor (BCF) has been measured in *Pimephales promelas* as 600 – 610 L/kg. The Log KOC has been measured in a range of 2.92 – 5.44, indicating high affinity for organic carbon.

Abiotic degradation rates in surface water are estimated to result in a half-life of 17-46 days via hydrolysis and 10 days via photolysis. EE2 is not readily biodegradable. The half-life in activated sludge has been reported as 1.3 – 12 hours under aerobic conditions and 1.0 – 8.3 days under anaerobic conditions. Biodegradation in a natural freshwater environment (River Thames) has been measured at 17 days.

Metabolism

It has been reported that a range of 27-85% of the ingested dose of EE2 is excreted unaltered. Therefore, in a region where 8.5% of the population (i.e. 17% of women) is ingesting EE2 as a daily oral contraceptive, the average excretion rate is 0.89 µg/day per person. Excretion can also occur via conjugation to glucuronides or sulphation in human liver (Temellini et al. 1991); (Ying et al. 2002). The EE2-conjugates are metabolized by bacteria (the glucuronide or sulphate used as a food source) in wastewater treatment plants (WWTP), which results in release of free EE2 in effluent (Ternes et al. 1999); (Racz and Goel 2010).

Potential Exposure Routes

The principal exposure route for aquatic taxa to EE2 is via WWTP effluent that is released into natural surface waters. The average removal efficiency of EE2 by primary and secondary sewage treatment plants has been estimated at 10% and 82%, respectively (Water-Framework-Directive 2011). It is expected that the environmental concentrations are likely to be highest in waters with closest proximity to WWTP effluent outflow under low flow conditions and that dilution of the compound will depend on hydrological factors (e.g. rainfall). Other aquatic exposure routes are via sediment and trophic pathways due to the affinity of EE2 to organic phases, as evidenced by the Koc (see above).

EE2-containing biosolids from WWTP (sewage sludge) may be used as fertilizer on agricultural fields and thus presents a potential pathway for EE2 into terrestrial environments, and aquatic environments via runoff. However, the dominant route of EE2 into waterways is through WWTP effluents, and therefore is the one considered in this document.

Articulation of which taxa will be considered in the case study (based on uses, exposure routes, available data etc)

For the purpose of this risk assessment document, only fish will be considered, due to the many data available and also because this vertebrate taxon, which possesses estrogen receptors, readily responds to EE2 exposure.

1. Methods
   1. Literature Search (regulatory, open and grey literature) and selection of data
      1. Search terms

For EE2 we had to limit the searches to receptors in the aquatic environment as there were too many papers (over 15,000) to review otherwise. This limited search resulted in 1371 papers for EE2 to be screened for relevance (Table S1-1).

Table S1-1. Literature search terms for EE2

| **Term target** | **Search term** |  |
| --- | --- | --- |
| 17 alpha ethinyloestradiol | 17 alpha ethinylestradiol, 17α-ethinyl *estradiol, Ethinylestradiol, ethynyl estradiol, ethinyl œstradiol, 17*-Ethinylestradiol, ethinylestradiol, CAS RN 57-63-6, and IUPAC name (17α)-19-Norpregna-1(10),2,4-trien-20-yne-3,17-diol |  |
| General aquatic ecotoxicity terms + general aquatic endocrine terms | Ecotox*, Reproduction, Development, Chronic, NOEC, vitellogen*, disorder, disrupt*, imposex, intersex, metamorphosis, mimic, modulat*, ovotest*, steroidogen*, testis-ova, xenoestrogen, gonado-somatic  **AND/OR**  Fish, Fathead minnow, Pimephales promelas, Rainbow trout, Oncorhynchus mykiss, Sheepshead minnow, Cyprinidon variegatus, Zebrafish, Danio rerio, Medaka,  Japanese ricefish, Oryzias latipes, Amphibia*, |  |
|  | (17 alpha ethinyloestradiol OR 17α-ethinyl *estradiol OR ethinylestradiol OR ethynyl estradiol OR ethinyl œstradiol OR 17*-Ethinylestradiol OR ethynylestradiol OR 57-63-6) AND (Ecotox* OR Reproduction OR Development OR Chronic OR NOEC OR vitellogen* OR disorder OR disrupt* OR imposex OR intersex OR metamorphosis OR mimic OR modulat* OR ovotest* OR steroidogen* OR testis-ova OR xenoestrogen OR gonado-somatic) **AND** (Fish OR Fathead minnow OR Pimephales promelas OR Rainbow trout OR Oncorhynchus mykiss OR Sheepshead minnow OR Cyprinidon variegates OR Zebrafish OR Danio rerio OR Medaka OR Japanese ricefish OR Oryzias latipes OR Amphibia*) | **Thomson (1995-2015): 677 hits**  Substance string taken from previous search strategy.  (“17 alpha ethinyloestradiol” OR “17*-ethinyl *estradiol” OR “ethinylestradiol” OR “ethynyl estradiol” OR “ethinyl oestradiol” OR “17*-Ethinylestradiol” OR “ethynylestradiol” OR “57-63-6”)  **Toxline: 920 hits**  Substance string taken from previous search strategy.  (“17 alpha ethinyloestradiol” OR “ethinylestradiol” OR “ethynyl estradiol” OR “ethinyl œstradiol” OR “ethynylestradiol” OR “57-63-6”) |
|  | (17 alpha ethinyloestradiol OR 17α-ethinyl *estradiol OR ethinylestradiol OR ethynyl estradiol OR ethinyl œstradiol OR 17*-Ethinylestradiol OR ethynylestradiol OR 57-63-6) AND (Ecotox* OR Reproduction OR Development OR Chronic OR NOEC OR vitellogen* OR disorder OR disrupt* OR imposex OR intersex OR metamorphosis OR mimic OR modulat* OR ovotest* OR steroidogen* OR testis-ova OR xenoestrogen OR gonado-somatic **OR** Fish OR Fathead minnow OR Pimephales promelas OR Rainbow trout Or Oncorhynchus mykiss OR Sheepshead minnow OR Cyprinidon variegates OR Zebrafish OR Danio rerio OR Medaka OR Japanese ricefish OR Oryzias latipes OR Amphibia*) | **Thomson (1995-2015): 1781 hits**  Substance string taken from previous search strategy.  **Toxline: 24899 hits (not downloaded)**  Substance string taken from previous search strategy. |
| Assay types | ER-CALUX, Hershberger, MCF-7, receptor binding, reporter gene, uterotrophic, YAS  Yeast, YES, steroidogenic |  |

- - 1. Brief statement cross referencing a table discussing types of issues the made studies relevant or not relevant.

For EE2, 1371 studies were found in the initial search from 2 a. i. (above) search terms. Duplicates were removed and the studies were assessed for relevance to EE2. This search resulted in 640 papers that screened in and could be classified into Levels 1-5 using the OECD conceptual framework (OECD 2012b) (Table S1-2). The 640 papers were distributed as follows: 121 Level 1, 58 Level 2, 223 Level 3, 184 Level 4, and 54 Level 5. From these groups we selected 202 studies to find PDFs for: 20 Level 2, 22 Level 3, 116 Level 4 studies, and 44 level 5 studies. We were able to obtain about 178 of the 202 selected PDFs, 96 papers from search engines available to us and 82 papers were available open access. These are the 178 papers we worked with for the EE2 risk assessment. We were also able to obtain the EE2 Water Framework Directive EQS Dossier (Water-Framework-Directive 2011), which we used to provide reviews of information such as exposure concentrations of EE2.

Table S1-2. Selection of relevant Level 1 to Level 5 literature for EE2.

| OECD Conceptual Framework (OECD 2012b) and rules of selection of EE2 database from 1371 papers down to 180 papers approx. | |
| --- | --- |
| OECD Level of Test | Number of papers selected and rationale |
| Level 1. Existing data and non-test information (e.g., physico-chemical properties, biodegradability, base set mammalian toxicity and ecotoxicity data, in silico data such as predicted log Kow, etc.) | Will extract this data from EE2 EQS Dossier (Water-Framework-Directive 2011) |
| Level 2. In vitro assays providing data about selected endocrine mechanism(s) or pathways(s) for both mammalian and non-mammalian methods (usually with an emphasis on agonism or antagonism against important endocrine system targets, such as nuclear hormone receptors or enzymes involved in sex hormone metabolism that may be shared across mammals and other taxa) | Aim for 20 level 2 studies = **In vitro** studies, MOA, biomarker, binding, **HPG axis focused endpoints**. Fish, mammalian, and invertebrate tissues. |
| Level 3. In vivo assays providing data about selected endocrine mechanism(s) or pathways(s) for either mammalian or wildlife (non-mammalian) species (the separation of mammalian versus non-mammalian data at this level reflects the need to consider differences in toxicant exposure routes, metabolism and excretion, as well as critical interspecies differences in endocrinology) | Aim for 20 level 3 studies, few early classical papers, and focus on papers in past 5 yrs. **In vivo** – **biomarker** endpoint. |
| Level 4. In vivo assays providing data on adverse effects on endocrine relevant end points for either mammalian or non-mammalian species (partial life cycle tests) | Selected all level 4 studies for non-mammalian species, primarily aquatic and avian. In vivo – apical endocrine-relevant endpoint. |
| Level 5. In vivo assays providing more comprehensive data on adverse effects data on endocrine relevant end points over more extensive parts of the life cycle of the organisms (e.g., multi-generation and full lifecycle tests with either mammalian or non-mammalian species) | Selected all level 5 **lifecycle** **+** studies for aquatic organisms and avian species (not terrestrial). |

- 1. Quality Evaluation of Relevant data
     1. Agree on classification of regulatory data

For EE2 most data are publically available and regulatory data play a minor role (compared to e.g. a plant protection product or REACH chemical).

- - 1. Use ToxRTool, a simple Excel-based system derived from the widely-used Klimisch method (Klimisch et al. 1997); (Schneider et al. 2009)
  1. Discuss methodology assessing the data
     1. Organize relevant and reliable studies/endpoints in accordance with the OECD conceptual framework for ED (OECD Guidance Document 150) (OECD 2012a)
     2. Recognize study/endpoint gaps in accordance with the OECD conceptual framework for ED (OECD 2012b)

EE2 is an example of a data-rich substance and there are very few gaps in the data for fish.

- - 1. Weight of Evidence Assessment

Many aspects of the adverse outcome pathway (AOP) for EE2 are defined.

1. Exposure Assessment – PEC (Predicted exposure concentration) values derived from regulatory submissions. (Each group needs to consider whether EU, US or combined approaches are appropriate.) Measured exposure levels in the field (if available) may also be considered, although these may not be representative of current use patterns.

A recent evaluation (Hannah et al. 2009) reported a worst-case scenario predicted environmental concentration (PEC) based on the highest population usage rate with 50% metabolism, 10% removal by primary sewage treatment, and no stream water loss or dilution as 5.8 ng/L [13 ng/L with no metabolism and no removal]. A summary of predicted environmental concentration (PEC) of EE2 in natural surface waters of North America and Europe resulted in 90th percentile low-flow PECs of 0.2 and 0.3 ng/L, respectively. The mean flow 90th percentile PEC for Europe was reported as 0.15 ng/L. Similar values generated by other modeling methods are summarized in Table S1-3, below.

In a model based on national drug consumption information, excretion, national water use, and sewage removal rates, 2-25% (by length) of Europe’s rivers would exceed 0.035 ng EE2/L (Johnson et al. 2013).

A summary of measured environmental concentrations (MEC) of EE2 in surface waters from 16 countries ranged from non-detects (< 0.01 [lowest LoD]) to 273 ng/L) (Hannah et al. 2009). The highest values are not typical and are unlikely to result from normal human use patterns (Anderson et al. 2012). A cumulative probability distribution of all 1,652 MECs determined by a range of analytical methods (detection limits 0.01 to 30 ng/L) showed a 90th percentile MEC of 1.7 ng/L (Hannah et al. 2009). A subset of 360 MECs determined by tandem mass spectrometry methods with an additional cleanup step following extraction (detection limits 0.1 to 1.0 ng/L) ranged from non-detect to 4.6 ng/L, with a 90th percentile concentration of 0.43 ng/L (Hannah et al. 2009) Hannah et al. (2009) (Hannah et al. 2009) concluded that, barring unusual environmental circumstances, environmental EE2 concentrations are unlikely to exceed 1.0 ng/L.

A summary of the ranges of all reported PECs and MECs for aquatic compartments is found in Table S1-3 below. No data is currently available for sediment or biota.

Table S1-3. Summary of EE2 PECs and MECs for freshwater (ng/L) as reported in EQS EE2 dossier (Water-Framework-Directive 2011)

PEC Worst-case scenario More-realistic estimate

5.8-13 0.075 – 0.11

50 1

0.15-0.3

MEC n=1652, all data n=360, refined dataset

1.7 [<0.01-273] 0.43 [<0.1-4.6] *

* 90^th^ percentile from Hannah et al 2009.

Overall, the refined dataset of measured environmental concentrations is consistent with the model output that considers more realistic input parameters.

1. Effects assessment

Organized according to OECD Conceptual Framework – perhaps in table form (see example provided at the end of this document).

- 1. Level 1 – summary of short term *in vivo* toxicology data; (may be informative concerning systemic effects) – suggest these data are not reviewed in great detail unless they become decisive in the data evaluation **Not reviewed**
  2. Level 2 – *in vitro* assays providing data about selected endocrine mechanisms/pathways (suggest including EDSP21)
     1. Potency could be discussed here based on specific MoA/mechanistic assays
  3. Level 3 – *In vivo* assays providing data about selected endocrine mechanisms/pathways
  4. Level 4 – *in vivo* assays providing data on adverse effects on endocrine relevant endpoints
     1. Fish (see Table S1-4 for list of studies, endpoints, and Klimisch score for Level 4 fish studies of EE2)
  5. Level 5 – *in vivo* assays providing more comprehensive data on adverse effects on endocrine relevant endpoints over more extensive parts of the life cycle of the organism
     1. Fish (Table S1-5 for list of studies, endpoints, and Klimisch score for Level 5 lifecycle fish studies of EE2)

5. WoE for Estrogen, Androgen, Thyroid and RXR Pathways (including Steroidogenesis Pathway disruption in estrogen, androgen; (RXR pathway is the most probable MOA for TBT in molluscs). This could be abbreviated depending on the substance, since the pathways of concerns will be known *a priori.*

1. Estrogen
   - 1. Does the chemical exhibit the potential for interaction with the estrogen pathway *in vitro* or *in vivo*?

EE2 is designed to act as an estrogen receptor agonist in humans. *In vitro* and *in vivo* estrogen receptor (ER) binding is well-demonstrated in mammals and fish. EE2’s potency for ER binding is 20 x higher than for estradiol (E2) (reviewed in (Caldwell et al. 2012)).

Lange et al (2012) compared E2 and EE2 estrogen receptor binding potency in several fish species. Of the fish species tested, the order of the most responsive to least was for EE2: zebrafish > medaka > roach > fathead minnow > carp > stickleback. For VTG mRNA induction in vivo, the order of species responsiveness was: rainbow trout (not tested in the ERα activation assays) > zebrafish > fathead minnow > medaka > roach > stickleback > carp. Overall, the responses to steroid estrogens in vitro via ERα compared well with those seen in vivo (VTG induction for exposure to EE2) (Lange et al. 2012).

There is ample in vivo evidence that exposed fish (especially male fish of gonochoristic species) react to low-level aqueous EE2 exposure with the induction of several biomarkers (Kime et al. 1999). These biomarkers can be induced in the laboratory, but also in the field (in situ) downstream of WWTP effluents. Well-known observations in male fish are an increase in the VTG plasma levels (preceded by increases in transcribed mRNA coding for VTG) and the induction of testis-ova (i.e. egg cells in testis tissue) (Kime et al. 1999); (Kirby et al. 2004).

- - 1. Does the chemical exhibit adverse effects potentially mediated by estrogen pathway?

There are three Adverse Outcome Pathway (AOP) Wikis under development for ER agonism:

- ER agonism leading to reduced survival due to renal failure
- ER agonism leading to skewed sex ratios due to altered sexual differentiation in males
- Estrogen receptor agonism leading to reproductive dysfunction

EE2’s adverse effects are mediated by its initial binding to the ER and subsequent activation of the estrogen pathway. Endpoints seen in Level 4 and Level 5 studies of EE2 are tied to the ER-binding mechanism and subsequent events that affect sexual differentiation, secondary sex characteristics, gonadal development, and reproductive performance.

ER binding resulting in VTG induction leading to degenerative changes in the glomerulus and kidney toxicity is one mechanism of specific toxicity and death from EE2 exposure. The specificity of this mechanism relies on EE2 binding causing excess VTG protein production. The apical endpoint of death from kidney toxicity may not be specific to EE2 or to VTG induction (as any protein in excess may cause nephron rupture and kidney toxicity). However, the specific pathway of EE2 binding to the ER and inducing excess vitellogenin causing kidney toxicity in fish is seen in several studies of EE2 at relatively high exposure concentrations. This is the proposed mechanism of lethality for 28 d LC50 in zebrafish of 100 ng/L reported in the Schäfers et al 2007 study (Schäfers et al. 2007).

ER binding leading to sex hormone imbalance or EE2 binding to and blocking AR (anti-androgenic action, (Sohoni and Sumpter 1998); (Wang et al. 2014), which in turn causes in male fish decreased testis growth (↓GSI), and altered differentiation and development of the testes. This is the proposed AOP mechanism for decreased fertilization success (decreased sperm production, impaired sperm maturation in M, impaired egg fertilization abilities) that has been observed in at least eight fish lifecycle studies (see Table S1-5 for references and details of the lifecycle studies).

ER binding leading to sex hormone imbalance, can cause female fish to have decreased ovary growth (↓GSI), and altered differentiation and development of the ovary. These result in impaired growth of the ovary, atreitic oocytes, and decreased egg production. This is the proposed mechanism for decreased egg production in at least six fish lifecycle studies (see Table S1-5 for references and details of the lifecycle studies).

ER binding leading to sex hormone imbalance during gonadal development can result in fish being feminized, intersex gonads or ↑ proportion of phenotypic females (or fish w ovaries). This is the proposed mechanism for feminization and development of testis-ova in several fish lifecycle tests (see Table S1-5 for references and details of the lifecycle studies).

- - 1. Are there Klimisch 1 and 2 *in vivo* tests lacking that include mechanistic endpoints relevant for (anti-)estrogenic MoA (OECD CF Level 3-4)?

We don’t feel tests are lacking. Every step of the AOP has been tested and demonstrated in level 3 and 4 studies.

- - 1. Are the adverse effects observed in higher tiered tests corroborated by lower tiered tests and can they be concluded to be a consequence of endocrine activity?

Adverse effects in higher-tiered tests are observed, and are linked to ER binding. The most sensitive reproductive endpoint in fish exposed to EE2 is often fertilization success. Eight lifecycle studies with several fish species have reported fertilization success as the most sensitive endpoint (Segner et al. 2003b); (Nash et al. 2004); (Parrott and Blunt 2005); (Schäfers et al. 2007); (Larsen et al. 2008); (Xu et al. 2008); (Zha et al. 2008b); (Soares et al. 2009). The molecular mechanism for decreased fertilization is linked to hormone balance in the organism. Several studies have shown EE2-exposed male fish have decreased plasma levels of male sex hormones such as testosterone (T) and 11-ketotestosterone (11-KT) (Nash et al. 2004); (Brown et al. 2007); (Filby et al. 2012); (Depiereux et al. 2014). Sex hormones such as T and 11-KT control the development and maturation of the testes in male fish. Studies assessing stages of testicular development in fish using histopathological tools show that EE2 exposure arrests gonad development (if exposure is in a critical developmental window) or delays gonad development and maturation of spermatozoa (Palace et al. 2002); (Nash et al. 2004); (Palace et al. 2006); (Oropesa et al. 2014).

The mechanism for decreased survival and growth of F1 is not clearly defined, although this is a sensitive endpoint in many studies. The endpoints responding to the lowest concentrations of EE2 in a lifecycle fathead minnow study by Lange et al 2001 was decreased F1 larval length and weight at 28 days post-hatch (dph) (Länge et al. 2001). Some fish lifecycle studies have assessed which phase of embryo development in the F1 offspring is affected by EE2. A study of F1 egg and embryo development in zebrafish showed a significant increase in the percentage of abnormalities at 8 hours post-fertilization (hpf) for the highest EE2 concentration (parents F0 exposed to 1 ng/L measured), resulting in increased embryo mortality at the 24 hpf (Soares et al. 2009). The two lowest tested EE2 concentrations (0.19 and 0.24 ng/L) impacted late gastrulation and/or early organogenesis, whereas exposure to 1 ng/L EE2 also disrupted development in the blastula phase (Soares et al. 2009). Increased embryo mortality occurred at EE2 exposure concentrations below those that induced reproductive impairment and VTG induction in the male parental generation of zebrafish. Further studies of these same zebrafish propose the increased embryo mortality in the F1 offspring after chronic low EE2 exposure of parental fish (F0) may be caused by an impairment of DNA repair in the gonad (particularly in male fish), as EE2 exposure caused an increased transcription of p53 gene in the gonads of males (Soares et al. 2012). Brown et al 2008 (Brown et al. 2008) saw increased aneuploidy in sperm from male rainbow trout exposed to 10 ng/L EE2 for 50 d during the period of sexual development. The defective sperm resulted in aneuploid embryos, which could be the mechanism for the decreased F1 embryonic survival observed in other studies (Brown et al. 2007).

- - 1. Are there Klimisch 1 and 2 higher tier *in vivo* tests lacking that include adverse endpoints relevant for (anti-)estrogenic MoA (OECD CF Level 4-5)?

We don’t feel tests are lacking. Every step of the AOP has been tested and demonstrated in Level 4 and 5 studies.

- - - 1. Derivation of No effect levels per taxa being assessed

Approach may differ based on information available and the chemical under evaluation (i.e. SSD or lowest NOEC with a certain assessment factor applied)

- 1. Discuss position of ‘endocrine’ effects relative to overall no effect levels

|  |  |
| --- | --- |

Figure S1-1. Species Sensitivity Distributions (SSDs) for EE2 NOECs and LOECs in fish lifecycle studies with 8 different species. In three cases NOECs had to be estimated as ½ of the LOEC from that study. Data used in these plots are summarized in Table S1-6.

**HC5 = 0.047 ng/L for NOEC and HC5 = 0.106 ng/L for LOEC**

A refined regulatory acceptable concentration (RAC) for the aquatic environment may be derived from the HC5 of a species sensitivity distribution (SSD) that is based on all available reproduction studies with the different fish species (Caldwell et al. 2012). A proposed EE2 RAC for fish and invertebrates from Caldwell et al 2012 is 0.1 ng/L. This was determined by constructing an SSD from NOECs from 22 reproduction studies of EE2 in eight fish species, and estimating the HC_5,50_. The new lower PNEC (compared to Caldwell et al 2008 PNEC of 2.0 ng/L) (Caldwell et al. 2008) results largely from new data on impaired fertilization success at 0.18 (measured) or 0.2 (nominal) ng/L EE2 in Chinese rare minnow, the most sensitive species (Zha et al. 2008b). Since there was no successful reproduction in F1 female Chinese rare minnows (exposed to 0.18 ng/L EE2) when they were mated with unexposed males for 21 days (Zha et al. 2008b), the NOEC of 0.10 ng/L we have estimated from this study is uncertain.

Endocrine-mediated effects from EE2 exposure are the most sensitive effects seen. Induction of vitellogenin occurs in fish exposed to 0.18-0.5 ng/L (Nash et al. 2004); (Zha et al. 2008b). These are similar concentrations that decrease egg production, decrease fertilization success, and decrease F1 egg viability in several species of fish after lifecycle exposures to EE2 (See Table S1-5 for references and summaries of endpoint data for lifecycle studies).

A sex ratio skewed towards females is an equally-sensitive endpoint in most EE2 lifecycle exposures. Because increasing the proportion of female fish may not result in a negative population effect overall this endpoint is more difficult to interpret. This is further complicated by other (natural) factors contributing to the dynamics in and deviations from a male-to-female sex ratio of 1:1 in gonochoristic species.

Also difficult to interpret is the presence of intersex fish in EE2 long term exposures. The presence of males with ovo-testis is hard to interpret alone, as studies on the breeding success of these males show different impacts on fertility. In some species of fish, significant but mild ovo-testis may not affect overall fertilization rate and therefore presence of ovo-testis alone may have no population-level consequences. Harris et al (2011) saw no effects of mild intersex on male wild roach’s ability to fertilize eggs, while severely intersex males had fertilization reduced by 76 % (Harris et al. 2011). Intersex male roach living downstream of a WWTP outfall for several generations were able to fertilize eggs and show successful reproduction. Further, a study employing satellite DNA analyses to prove that the population was not supported by immigration and reproduction of non-exposed Roach from other areas) (Hamilton et al. 2014). In other fish species such as rainbow darter (*Etheostoma caeruleum*) the severity of intersex in male fish captured downstream of WWTP effluent outfalls was studied to see if it was linked to a reduced ability of collected milt to fertilize eggs in the lab (Fuzzen et al. 2015). However the correlation was weak and the data were scattered, such that from our assessment of the data, it appears that only the few males with the very highest intersex rating had reduced ability to fertilize eggs. In lab studies mild to moderate intersex in Medaka males does not affect reproductive success (Seki et al. 2002).

Endpoints such as courtship and mating behaviours appear to be slightly less sensitive to EE2, as several studies have seen normal mating behaviours in male fish that had testicular changes and were severely compromised in their ability to fertilize eggs. (Kristensen et al. 2005); (Larsen et al. 2008). Fathead minnow nest defense behaviour after 21 d exposure was affected at higher concentrations than changes in gonad size (Salierno and Kane 2009).

Lee 2014 showed reduced mating behaviour in Brackish Medaka, and these changes occurred at similar concentrations to failed reproduction (50 ng/L) (Lee et al. 2014).

Changes in overall growth, organ size (GSI, LSI), and survival over time occur at higher exposure concentrations than the NOEC for reproductive effects (See Table S1-5 for references and endpoints).

- 1. Agreement and rationale for which endpoints will be used quantitatively in the risk assessment

For the risk assessment the most appropriate endpoints to use are reproductive endpoints such as decreased fertilization success, decreased viable offspring, and decrease in egg production, taken from long-term exposure studies. These are sensitive, population-relevant endpoints that have been demonstrated in lab EE2 exposures of several fish species. They are also endpoints that have been linked to observed population decreases in fish after a multi-year controlled field exposure to EE2 field (Kidd et al. 2007); (Palace et al. 2009).

- - - 1. Risk Assessment

1. Compare PEC to various No Effect Levels following established risk assessment practices

PEC and MEC may be compared to a regulatory acceptable concentration (RAC) derived from (in the case of EE2) chronic fish studies. Various approaches exist:

Deterministic: lowest NOEC or EC10 divided by an assessment factor (AF; usually between 1 and 10)

Refined deterministic: The geometric mean of all available, robust NOEC or EC10 values divided by an AF (usually between 1 and 10)

Probabilistic: The HC5 derived from an SSD that is based on relevant and robust NOEC or EC10 values divided by a low AF.

Further higher tier information, such as results of field testing (Kidd et al. 2007; Palace et al. 2009) and observations on exposed wildlife populations should be considered in a weight of evidence for setting an appropriate RAC.

The value of the AF differs between geographies, but should reflect the confidence one has in the data, i.e. more studies and/or longer-term studies allow for a reduction in uncertainty and thus for a lower AF.

For EE2 the MECs are considered more relevant/realistic than the PECs as exposure estimates for use in the risk assessment. Instead of using single maximum peak concentrations it is considered more relevant to employ a 90^th^ percentile or mean/median value to characterize chronic exposure. Various MEC values can be used that reflect different scenarios from worst case (e.g. low flow river conditions large amount of sewage effluent from STP in highly populated area) to realistic to best case.

**From the SSD, the HC5 = 0.047 ng/L for NOECs and HC5 = 0.106 ng/L for LOEC for reproductive effects (8 species) (from Figure S1-1). This is compared to the freshwater MECs (from Table S1-3) of 0.43 ng/L (90^th^ percentile).**

The HC5 estimated from the SSD for lifecycle effects of EE2 on fish reproduction is lower than the median EE2 concentration range in European rivers. This suggests that in a majority or rivers the most sensitive 5 % of fish species will be affected. If we use the predicted EE2 river concentration model from (Johnson et al. 2013), about 8 % of European rivers would be above the HC5 of 0.05 ng/L.

1. Are the endocrine-mediated **adverse** effects on wildlife possible at environmentally relevant exposure levels?

Adverse effects on fish downstream of wastewater treatment plant (WWTP) effluent outfalls have been observed in Europe and North America (Jobling and Tyler 2003); (Tetreault et al. 2011); (Sumpter and Jobling 2013). The endpoints affected are often VTG induction and testis-ova (Harries et al. 1996); (Tyler and Jobling 2008). Exposures are to the mixture of industrial and pharmaceutical chemicals found in WWTP effluents. However in some cases, concentrations of EE2 alone in the effluents were the likely cause of the observed effects (Desbrow et al. 1998); (Jobling et al. 2006). In most cases adverse effects were limited to a small area where effluent was most concentrated.

Adverse effects on fish exposed to 5-6 ng/L EE2 have been demonstrated in long-term lake dosing experiment with EE2 (Kidd et al. 2007; Palace et al. 2009). These exposure concentrations were not environmentally realistic, but several observations could be made on the timing of events after the exposure began, and on relative species sensitivities.

Species differences exist in the ER-binding sensitivity to EE2 and VTG induction among fish species (Lange et al. 2012). In vivo, experiments have shown that some shorter-lived species are particularly sensitive to EE2 exposure (at relatively high concentrations of 5-6 ng/L), as reproductive failure can cause complete population collapse within a year or two for fathead minnow, and within 3 years for pearl dace (Kidd et al. 2007; Palace et al. 2009). Longer lived species such as lake trout appeared to suffer from secondary non-specific effects in these EE2 lake-dosing studies from the loss of smaller fish as food sources. This may result in slower growth, reduced reproduction, or death by starvation (Palace et al. 2009). Some species such as the white sucker did not appear to be affected (Palace et al. 2009). It is recognized that species differences in the whole-lake EE2 dosing study could be related to exposure conditions (where the fish lived in the lake, in deep or shallow areas that differed in their average EE2 concentration) or life history traits (some fish move to streams prior to spawning etc.) (Palace et al. 2009).

- - - 1. Sources of Uncertainty/Data gaps for hazard assessment

a. Potential concerns for ED hazard assessment

i. Multi-generational effects

We reviewed EE2 studies that have studied multigenerational effects, where eggs from F1 were hatched in clean water. This type of exposure is best to determine true multigenerational effects, as the effects in the F1 offspring would arise from exposure of the parent only. Nash et al 2004 studied clean water-hatched eggs (from EE2-exposed parents at concentrations of 0.5 and 4.5 ng/L) and saw no multigenerational effects (on embryo viability, embryo mortality, or larval integrity) (Nash et al. 2004). There were also no multigenerational effects on breeding success in the F1 later on when reared in clean water to adulthood. The F2 offspring hatched and survived similar to control fish offspring (Nash et al. 2004). Exposure of rainbow trout F0 males to 0.8 ng/L EE2 resulted in decreased survival of F1 offspring (Brown et al. 2007). However, the surviving unexposed male F1 trout, when mature (1 and 2 yrs old), were able to sire normal offspring (F2) with same survival rate as controls. There are other studies that also show little difference in effects from one generation to the next. F2 zebrafish embryo growth was decreased by EE2 (162 d LOEC = 2.0 ng/L) at similar exposure concentrations to F1 growth (75 d LOEC = 1.1 ng/L) (Schäfers et al. 2007). Overall, a review by Schwindt 2015 states that there appear to be no clear demonstrations of heritable multigenerational effects in aquatic wildlife exposed to EDS (Schwindt 2015).

There are some multigenerational studies that are suggestive of effects, however the study designs prevent clear conclusions to be drawn as only one EE2 test concentration was used (Bhandari et al. 2015) or EE2 exposure concentrations were variable and overlapping (Schwindt et al. 2014). There is some evidence that extremely high EE2 exposures can result in multigenerational effects. Bhandari et al 2015 saw multigenerational effects in Medaka F2, F3, and F4 after very brief in-ovo 7-day exposures of F0 to 61 ng/L EE2 (measured). Medaka gametes are formed in-ovo during this 7-day period, so the F1 were exposed only as germ cells in the developing gametes of the F0 embryonic parents (Bhandari et al. 2015). The study utilized only one concentration and so we scored it as a Klimisch 3 level. F1 adults produced significantly more eggs, and F2, and F3 adults produced normal egg numbers. There were multigenerational effects on F3 and F4 embryo survival, while effects on fertilization success were found in the F2 only. Schwindt et al 2014 studied multigenerational effects in mesocosm studies of EE2, however exposure concentrations varied, and replication of the progeny-exposure aquaria was low (Schwindt et al. 2014). Because of this the study rated a Klimisch 3 score.

If eggs are raised and hatched in EE2-containing water, multigenerational effects cannot be easily separated from early-exposure effects. Some studies showed increasing effects with EE2 generational exposures and lower NOECs from subsequent generations when exposure was continuous. F1 swim up success of Chinese Rare Minnow was negatively affected at 0.91 ng/L, while parental F0 survival at swim up was not affected until 13.6 ng/L (Zha et al. 2008b). Fathead minnow F1 larval length and weight were decreased at 0.16 ng/L EE2, while F0 length and weight were negatively affected at higher concentrations of 12 ng/L (Länge et al. 2001). These studies suggest the uptake of EE2 into the egg during its development in the exposed female parent fish is important. The egg’s exposure during egg maturation inside the parent increases the sensitivity of the hatched larval fish to further EE2 exposure once the egg is laid and fertilized.

ii. Sensitive species

Chinese Rare minnow appears to be the most sensitive fish species tested to date, both in short-term exposures of adults (fertilization rate was zero at 3.6 ng/L,(Zha et al. 2008a) and in multi-generational exposures (no reproduction of F1 0.18 ng/L, (Zha et al. 2008b)). Is there evidence that short lived species are more affected by EE2 (like in (Palace et al. 2009) whole-lake EE2 dosing species sensitivity comparison), or is it just easier to see the population-level effects within the experimental timeframe? Palace et al 2009 saw changes first in fathead minnow, then pearl dace, then lake trout. White sucker were unaffected by exposure to 5-6 ng/L EE2 (Palace et al. 2009).

Fish with non-standard reproductive strategies may be more tolerant to the effects of EE2. One study performed on the hermaphroditic fish *Kryptelobias marmaratus* larvae showed that they were incredibly EE2 tolerant, with nominal-concentration-LOECs of 100,000 ng/L for gene expression, and 500,000 ng/L for adverse effects on egg fertility (Farmer and Orlando 2012).

iii Sensitive windows of exposure

Exposure during sexual differentiation and gonadal development appears to be necessary for most dramatic and non-reversible responses on sex ratios and fertilization success. Nash et al 2004 showed F1 zebrafish (from exposed F0 parents) exposed to 0.5 ng/L EE2 had lower fertilization success even after 5 months’ depuration in clean water (Nash et al. 2004). Exposure of zebrafish to EE2 during sexual differentiation leads to altered sex ratios with a LOEC < 0.6 ng/L (Örn et al. 2003) whereas exposure to 15.4 ng EE2/L before 25 dph does not affect sex ratio; the sensitive window is 20 to 60 dph (Andersen et al. 2003). For fathead minnow the window of sensitivity for male fish is 5-20 dph, where early EE2 exposure (for 5 days at 10 ng/L nominal) can result in delayed testicular development (reduced spermatozoa) at 100 dph (Van Aerle et al. 2002). For Medaka the sensitive window is from hatching to 1 week post-hatch (Koger et al. 2000).

iv Potency

EE2 is a very potent ER-binding compound designed to have a specific MOA. The high potency contributes to easier-to-interpret studies that for the most part do not have systemic toxicity as an interfering factor. In most cases the endpoints affected in EE2-exposed fish are directly related to ER binding.

The specificity of the mode of action of EE2 (i.e. its activity at sub ng/L concentrations) contributes to it being a difficult to measure compound. In some experiments, very low concentrations of EE2 can cause adverse reproductive effects in fish, and sometimes these concentrations are non-detectable, or at least, difficult to detect analytically (as concentration steps must be employed prior to measurement of exposure waters). Thus, in 7 of 18 lifecycle studies, the lowest concentrations tested were below the level of detection of this compound, although it did cause effects. Unfortunately, lack of analytical measurements means also the correct dosing of the water cannot be verified. This affects the quality of the data and the Klimisch rating is lower for studies where measured concentrations are not reported for some exposure concentrations.

v Non-monotonic dose response or lack of a threshold dose

There are a few examples of EE2 causing non-monotonic dose-response curves. The most common of these is related to stimulation of egg production. It appears that low concentrations of EE2 can stimulate egg production in female zebrafish and fathead minnow (Nash et al. 2004); (Parrott and Blunt 2005), but that higher EE2 exposure concentrations reduce egg production.

vi Data gaps with dose setting

There are some studies where NOECs for reproductive endpoints could not be determined as decreased fertilization, egg production, or % viable offspring were negatively affected at the lowest exposure concentration tested (Nash et al. 2004); (Parrott and Blunt 2005); (Brown et al. 2007); (Xu et al. 2008); (Zha et al. 2008b). The ratios of LOECs to NOECs ranged from 2.5 to 10 for fish lifecycle studies where both were determined (see Table S1-6 for data).

In addition, some lifecycle studies of EE2 used only one exposure concentration (Robinson et al. 2003); (Fenske et al. 2005); (Hallgren et al. 2014), in some cases because EE2 was the positive control in the testing of another substance. Studies using only one test concentration were rated as a 3 on the Klimisch scale, and provided no useful LOECs or NOECs, although they could contribute to a weight of evidence approach.

vii Recovery of Reproduction & Reasons for Decreased Fertility

Some experiments show no potential for recovery if EE2 exposure was during a critical window of exposure. Xu et al 2008 exposed larval zebrafish to EE2 for 90 days, and then transferred the adult fish to clean water for a further 90-d depuration and grow-out. They found no recovery of egg production or fertilization success (when fish were 180 dph) after 90 days in clean water (Xu et al. 2008). Similarly Schäfers et al (2007) examined recovery in adult zebrafish exposed to 9.6 ng/L EE2 (from the egg stage) that produced no eggs after 177 days of exposure (Schäfers et al. 2007). These fish, when transferred for 108 d to clean water, did recover their sex characteristics, reproductive behaviours, and had 2/3 of normal egg production. However, the impact on fertilization success remained, with only 3 % of eggs being fertilized (vs 95 % in controls) (Schäfers et al. 2007).

Xu et al 2008 also performed experiments assessing which sex was responsible for the breeding failure after chronic EE2 exposure. They performed female and male replacement trials where unexposed control fish were mated with EE2 exposed zebrafish (exposed to EE2 for 90 d from larvae to adult). The results showed that the reproductive problems resided with both sexes. In trials where EE2 exposed males were mated with control females they observed that males still had decreased ability to fertilize eggs, even with good eggs from control females (Xu et al. 2008). As well, control females laid fewer eggs when paired with EE2 exposed males. In trials where EE2 exposed females were mated with control males, they observed that females exposed to low EE2 (0.4 ng/L) were not producing as many eggs, but the ones they did produce were of good quality as control males fertilized them well (Xu et al. 2008).

9. Sources of Uncertainty/Data gaps for risk assessment

a. Potential concerns for ED risk assessment

1. See above under hazard assessment plus uncertainties related to exposure

There do not appear to be any real data gaps that would make the EE2 risk assessment uncertain. The specificity of the MOA and high potency mean the very low concentrations are necessary to cause effects. Often these concentrations are difficult measure, and so some studies do not attempt exposures at low enough concentrations. The lack of determined NOEC in some lifecycle studies impairs the information available from these long-term exposures. In these cases it would have provided more meaningful information if a few lower concentrations had been tested so that a real NOECs could be determined and not approximated. In several studies the lack of measured EE2 concentrations (especially at sub ng/L) decreases the Klimisch score and the applicability to risk assessment. Related to this issue is the difficulty in measuring EE2 reduces the usability of some MEC data. Some compilations of measurements of EE2 in river water have over 70 % of the values as non-detectable (Hannah et al. 2009); (Water-Framework-Directive 2011).

1. Conclusions for the Case Study – Hazard and Risk assessment
2. Can we reach consensus on the appropriateness of risk and hazard in order to inform regulatory decision making?

EE2 is a data-rich substance with a clear mechanism of action. The adverse effects are mediated by an AOP resulting in adverse reproductive effects in many fish species tested. Because of the huge database of effects in EE2-exposed fish, we suggest a risk-assessment approach is suitable for assessment of this substance.

Table S1-4. Summary of studies and Klimisch score for Level 4 studies of EE2 in fish.

| **Taxonomic Group/**  **Species name** | **Study Type** | **Klimisch Score** | **Conc’ns**  **Tested** | **Results**  Separate endpoints into **population-relevant (adverse)** and non-population-relevant (biomarker)  **LOEC NOEC** | | **Reference** |
| --- | --- | --- | --- | --- | --- | --- |
| *Oncorhynchus mykiss* | 56 d exposure at 587 dpf. 10 male fish/group. Duplicates. 11-KT & LH determined. Semen collected. Fry survival determined at 19 dpf. Fertilization rate **not** determined. | 1 | 0.8, 8.3, 65 ng/L | ↓ fry survival: 0.8  ↓11-KT: 65  ↑LH: 65 | 11-KT: 8.3  LH: 8.3 | (Brown et al. 2007) |
| *Oncorhynchus mykiss* | 50 d exposure. Frequency of aneuploidy determined in semen. N=4. No replication. | 2 | 9 ng/L | ↑ Semen aneuploidy |  | (Brown et al. 2008) |
| *Danio rerio* | 17 d exposure; adults. 4 replicates. Egg production, 11-KT and behavioral hierarchies determined | 1 | 2.4 & 10.6 ng/L | Behavioral hierarchy. : 10.6 | Egg production: 10.6  11-KT: 10.6 | (Coe et al. 2008) |
| *Danio rerio* | 40 d exposure 20-60 dpf. No replication. Sex ratio, fecundity and behavioral hierarchies determined at 300 and 485 dpf.  Note: potential reversibility (sex ratio) | 2 | 2.8 & 9.9 ng/L | ↑Egg viability: 2.8 | Intersex: 9.9  11-KT: 9.9  Sex ratio: 9.9 | (Coe et al. 2010) |
| *Oncorhynchus mykiss* | All male. 76 d from 60 dpf. 108/group. No replication. Gene expression, testes histology, 11KT, T, E2 determined | 1 | 10 (nom), 80, 1620 & 9880 ng/L | ↓11-KT: 9880  Testes histology: 10  Many genes: 80-1620 | 11-KT: 1620  E2: 9880  T: 9980 | (Depiereux et al. 2014) |
| *Oreochromis aureus* (tilapia) | F1 exposed in food. No exposure in F2. Very high doses. | 3 | 150 mg EE2/kg food | ↓ Reproduction in pseudofemales: 150 mg/kg food |  | (Desprez et al. 1995) |
| *Gasterosteus aculeatus* | 7 d adult exposure. Semi-static 40%/d. Behavioral studies | 1 | 15 ng/L | Behavior ♂→♀ ↓ 15  Behavior ♂→♂ ↓ 15 |  | (Dzieweczynski and Forrette 2015) |
| *Betta*  *splendens* Siamese fighting fish | 28 d adult exposure. Semi-static 100%/d. 45/group, no replication in exposure. Individual behavioral traits. | 1 | 9 ng/L | Behavior ♂→♀ ↓ 9  Behavior ♂→♂ ↓ 9 |  | (Dzieweczynski et al. 2014) |
| *Kryptelobias marmaratus (*hermafroditic) | 0-28 dph. Semi-static 50% twice a week. 15/group. No replication. Gene expression and fertility examined at ≈200dph.  No effect on gonad *cyp19a1a, cyp19a1b, ERβ, figα, rpl8* | 2 | 100000, 500000 & 1000000 ng/L | ↓brain *cyp19a1a*: 100000  ↓ gonad *ERα*: 1000000  ↓ gonad *dmrt1*: 500000  ↓ fertility: 500000 | Brain *cyp19a1b*: 1000000  gonad *ERα*: 500000  gonad *dmrt1*: 100000  Fertility: 100000 | (Farmer and Orlando 2012) |
| *Pimephales promelas* | 28 d adult exposure. Flow-through. 6♂+6♀. Duplicated. Fatpad, tubercles | 1 | 13 ng/L | ↓ ♂ tubercles: 13  ↓ ♂ fatpad: 13  ↑ ♂&♀ plasma vtg: 13  ↑ ♂ *vtg* hepatic expression: 13  ↑ ♂ *esr1* hepatic expression: 13  ↑ ♂ *esr1* gonad expression: 13 | ♀ *vtg* hepatic expression: 13  ♀ *esr1* hepatic expression: 13  ♀ *esr1* gonad expression: 13 | (Filby et al. 2007) |
| *Danio rerio* | 2♂&2♀ in 12 aquaria. 10 d adult exposure of dominant male (n=6) to 10 ng/L – nominal only. Flow-through. Behaviour, 11-KT and gene expression determined. | 2 | 10 ng/L | ↓ fertility of dominant male: 10  ↓ aggressive behavior of dominant male: 10  ↓ 11-KT of dominant male: 10  ↓ expression of *ar*, *cyp17*and *hsd17b3* of dominant male: 10 |  | (Filby et al. 2012) |
| *Tinca tinca* | Exposure by injection. 7 tench/group – duplicate. 30 d exposure. Injection once a week. | 1 | 50, 100 & 500 μg/kg | Not applicable for risk assessment |  | (Oropesa et al. 2015) |
| *Tinca tinca* | Exposure by injection. 7 tench/group – duplicate. 30 d exposure. Injection once a week.  Same experiment as Oropesa et al 2015? Some data on weight and length are the same, others are not!!!??? | 1 | 50, 100 & 500 μg/kg | Not applicable for risk assessment |  | (Oropesa et al. 2015) |
| *Danio rerio* | 2 experiments: 0-35 dpf and adult ♀15 d exposed to nominal 5000 ng/L. Various endpoints. | 1 | 5000 ng/L | Exposure concentration too high to be relevant for risk assessment |  | (Ortiz-Zarragoitia et al. 2006) |
| *Margariscus margarita* | Dosed experimental lake.  Indications and trends of several effects: ovary steroidogenesis, fewer young-of-the-year size classes, but no obvious population effects | Field trial | 5-6 ng/L | All 5-6:  ↑ ♂&♀ plasma vtg:  ↑ Ovary edema  ↓ Testicular development  ↑ intersex  ↑ kidney lesions |  | (Palace et al. 2006) |
| *Pimephales*  *promelas* | 21 d exposure of adult ♂&♀ (gonadal recrudescence assay). Flow-through. Nominal – except 1 & 10.  Note that number of eggs ↑ at 0.1 and 1 ng/L, but for higher concentrations a dose-dependent decrease was seen. | 1 | 0.1, 1, 3, 10, 100 ng/L | ↓ ♀GSI & ♀ CF: 100  ↓ ♂GSI, ♂CF, number of batches of eggs, fertilization rate: 10  ↓ extent of parenchymatic areas in ovaries: 3  ↑ ♂&♀ plasma vtg: 1  ↓ ♂ tubercles: 1  ↑ number of eggs: 0.1 | ♀GSI & ♀ CF: 10  ♂GSI, ♂CF, number of batches of eggs, fertilization rate: 3  extent of parenchymatic areas in ovaries: 1  ♂&♀ plasma vtg: 0.1  ♂ tubercles: 0.1 number of eggs: No NOEC | (Pawlowski et al. 2004) |
| *Odontesthes bonariensis* | 0-42 dph exposure via food. Semistatic 25%/d. Duplicated. Gene expression determined at 2, 4 & 6 weeks. Sex and gonad histology at 9 or 11 weeks. |  | 100, 500 & 1000 ng/g food | Sex ratio (↑♀): 100 ng/g  ↑*cyp19a1a*: 1000 ng/g  ↓ *hsd11b2*: 100 ng/g  ↓*arα*: 1000 ng/g | *cyp19a1a*: 100 ng/g  *erα* & *erβ*: 1000 ng/g *arα*: 100 ng/g | (Pérez et al. 2012) |
| *Danio rerio* | 21 d adult exposure. 16♂&16♀/treatment. Semistatic; once a d (%?).  Actual conc. **Nominal & actual suspiciously identical (Table S1-1).** |  | 100 ng/L | ↓♂&♀ GSI: 100 |  | (Silva et al. 2012) |
| *Clarias gariepinus*  Catfish | Exousure 0-50 dph. Semistatic (Daily shift; %?). Triplicates. Unclarity: Gene expression vs. concentration? Nominal conc. only  Malformations | 3 | 50, 100, 500, 1000 & 5000 ng/L | Sex ratio (↑♀): 50 |  | (Sridevi et al. 2015) |
| *Danio rerio* | 14 d exposure of adult (54 sets of 2♂&1♀). Two strains, WIK & BLD01. Actual conc. |  | 0.4 & 2.2 ng/L | ↑ ♂ plasma vtg: 2.2  ↑ Unfertilized eggs in strain BLD01: 2.2  ↑♂sexual behavior in strain WIK: 0.4 | ♂ plasma vtg: 0.4  Egg production: 2.2 Unfertilized eggs in strain BLD01: 0.4  Unfertilized eggs in strain WIK: 2.2  ♂sexual behavior in strain BLD01: 2.2 | (Söffker et al. 2012) |
| *Pimephales*  *promelas* | 29-30 d exposure from 2hpf. Semistatic (100%/d).  Skeletal malformations determined. No specific ED related endpoints. | 1 | 100, 1000, 10000 & 100000 ng/L | ↑ Skeletal malformations: 100 |  | (Warner and Jenkins 2007) |
| *Gobiocypris rarus* | 21 d exposure of adults. 6 breeding pairs/treatment. Flow-through.  Renal somatic index: ↑ & liver lesions | 1 | 3.6 ng/L | All 3.6:  ↑ ♂&♀ plasma vtg:  ↑ ♀ HSI  ↑ Egg laying interval  ↓ Eggs/♀  ↓ Eggs/batch  ↓ fertilization rate (=0)  ↑ testes-ova | ♂&♀ GSI: 3.6  ♂ HSI: 3.6 | (Zha et al. 2008a) |
| *Gobiocypris rarus* | Multigeneration exposure from F0 2hpf. Flow-through. Actual conc.  Malformations, gonad, liver and renal lesions (EDC effects ?). **An important study** |  | 0.18, 0.91, 3.6, 14, 56 ng/L | ↑ ♂ plasma vtg: 0.18  ↑ ♀ plasma vtg: 0.91  Sex ratio (↑♀): 0.18  ↑ testes-ova: 0.18 | ♀ plasma vtg: 0.18 | (Zha et al. 2008b) |
| *Oryzias latipes* | Exposure 0-90 dph. Screening 75 ng/L. Main: 5.6 – 51 ng/L. Duplicates. Flow-through. 6 ♂ from each treatment examined.  Search for genes associated with the development of intersex. Lots of information on ‘ovary’-genes | 1 | 5.6, 16, 30, 41, 51 ng/L | ↑ intersex: 30 | Intersex: 16 | (Zhao and Hu 2012) |
| *Oryzias latipes & Danio rerio* | Exposure 0-60 dph; semistatic (50%/2^nd^ d). Nominal concentrations.  Vtg and sex ratio determined | 1 | 10 & 100 ng/L | ↑ zebrafish-vtg: 10  ↑ medaka-vtg: 100  Zebra Sex ratio (↑♀): 10  Medaka Sex ratio (↑♀): 100 | medaka-vtg: 10 Medaka Sex ratio: 10 | (Örn et al. 2006) |
| *Dania rerio* | 7 d adult exposure. d. Sex’l differentiation; vitellogenin induction | 1 | 15.4 ng EE2/L  9 dev’l exposure periods (1 dose) | Sex diff 15.4 ng/L  Vit indxn 15.4 ng/L | <15.4 ng/L | (Andersen et al. 2003) |
| *Fathead minnows (P. promelas)* | Expt 1: static daily renewal; 2 chemicals, 1 dose  Expt 2: flow through; 1 CAFO mixture, 1 dose | 1 | EXPT 1: 5-10 dph 5 ng EE2/L  or 5 ng TRB/L  EXPT 2:0-45 dph  2-16 ng CAFO mix/L  Control tanks exposed to AND, E1, 17BE2. | EXPT 1:  ♀ EE2: ↑ dmrt1, ↓cyp19a, ↓cyp17, ↓star, ↑ esr1  ♀ TRB: ↓ dmrt1, ↓cyp19a, ↓cyp17, ↓star  ♂ EE2: ↑ esr1  ♂ TRB: ↓star  EXPT 2: Males with ovarian cavities ♂→♀ | EXPT 1: EE2 or TRB <3.3 ng/L  EXPT 2: <nominal concentration CAFO | (Leet et al. 2015) |
| *Danio rerio* | Water exposure  EXPT 1: 10 ng EE2/L 3 developmental stages; recovery of 150-180 dof and 213-250 pf; 4 x 80 larvae/treatment; 16 aquaria.  Repro – 8♂&4♀ in 12 aquaria  EXPT 2: 1.67, 3, 10 ng EE2/L gonad transitional stage (43-71 dpf); 4 x 15 df larvae/treatment  28 d adult exposure.  Semi-static. | 1 |  | EXPT 1: 1.67 ng EE2/L  ↓ fecundity: 10 ng/L  ↓ fecundity, fertilization success (43-71 dpf) all conc.  EXPT 2: 3 ng EE2/L  ↑ ♂&♀ (mixed gender) gonads for 43-71 dpf group at 3 ng EE2/L | EXPT 1: <1.67 ng EE2/L  EXPT 2: 1.67 ng EE2/L | (Maack and Segner 2004) |
| *Fathead minnows (P. promelas)* | Predator avoidance behavior (C-start)  Water exposure; 4 treatments (E1, E2, EE2, mix); static renewal  embryo exposure 2 x 20/trt; 12-d exp.  larval exposure 2x20/trt; 12-d exp. | 1 | E1 5, 50, 100 ng/L  E2 1, 10, 28 ng/L  EE2 0.1, 1, 10 ng/L  Mix=sum of all 3 | Embryos: escape delay in E1 medium dose  Larvae: no sig diffs | Not applicable for risk assessment | (McGee et al. 2009) |
| *Japanese medaka (O. latipes)* | Larval exposure to 8 compounds; static renewal, growth & histology  E2, EE2, Estrone, Estriol, NP1EO/NP2EO, NP1EC/NP2EC, bisphenol A, DEHP  1-d after hatch: 90-d exposure  YES screening assay | 1 | 3-5 conc. | Larval exp: dose-related sex ratios;  E2 1 ng/L;  estrone 10 ng/L; bisphenol A 10 µg/L  YES: Dose-related responses | E2 0.1 ng/L;  estrone 1 ng/L; bisphenol A 1 µg/L | (Metcalfe et al. 2001) |
| *Japanese medaka (O. latipes)* | Repro: 14d EE2 exp to breeding fish  25 breeding groups of 1♂ and 3♀.  Fertility & Testicular histo, gene expression (sampled Days 1, 7, 14) | 1 | 1 or 10 µg EE2/L | Fert: 1 µg EE2/L (62.8%)  Histo: 1 µg EE2/L  ↑ interstitial tissue thickness;  ↑ apoptic spermatocytes and spermatids  Genes: 1 µg EE2/L; time and conc dependent  ↑ # significant transcripts expressed | Fert: <1 µg EE2/L (some recovery)  Histo: <1 µg EE2/L  Genes: <1 µg EE2/L | (Miller et al. 2012) |
| *Cunner (Tautogolabrus adspersus)* | exposure of adult 2♂&3♀  Long term: 7-8 wk when fish were acclimated from overwintering to spring spawning conditions  Short term: 2-wk once spawning was well underway | 1 | Implants 0.05, 0.5, 2.5 mg/kg bw (E1, EE2, estrone) |  |  | (Mills et al. 2003) |
| *Cunner (Tautogolabrus adspersus)* | 14-d exposures with subcutaneous implants; 4 to 6 tanks/rep w/1♂& 2 or 3♀.  Aromatase activity (♂&♀ brain; ovarian)  Plasma VTG  Egg production, # fertile eggs, %viable eggs, GSI, | 3  (1 dose for E2, EE2; 2 doses for ATD, OP; no NOECs) | E2 4.8 mg/kg  EE2 1.2 mg/kg  ATD 40,200 mg/kg  OP 20, 200 mg/kg | E2 4.8 mg/kg; EE2 1.2 mg/kg; ↓ egg prodxn, ↑♂ brain aromatase, ↓ ovarian aromatase  ATD 200 mg/kg ↓ egg prodxn,  OP – no effect | E2 <4.8 mg/kg  EE2 <1.2 mg/kg  ATD: 40 mg/kg (egg prodxn)  OP: >200 mg/kg | (Mills et al. 2014) |
| *Betta splendens* | 28 d adult semi-static exposure (145 fish ??/treatment??). Semistatic; 3x/wk 25% renewal.  Swimming performance & fertilization potential  Gonad size, sperm performance, parental beh ,fert. Success; sperm performance related to ATP in sperm | 1  (2 doses) | EE2 0, 10, 100 ng/L | Sperm parameters – no effects at 100 ng/L;  ↓GSI, sperm count, ATP in sperm, Fert success, Nest size, fert success: 100 ng/L | Sperm (direct exposure), motility: >100 ng/L  GSI, sperm count, ATP in sperm, Fert success, Nest size, fert success: 10 ng/L | (Montgomery et al. 2014) |
| *Dania rerio* | Semi life cycle. Semistatic; 3x/wk 50% renewal. Measured conc.(LC-MS)  EE2 – 20-60 dph; 2C, 3 reps/treatments (40 larval fish/tank), mortality, VTG, histopath  MT – 20-60 dph; 1tank/treatment (40? Larval fish/tank?) | 1 | EE2 0, 1, 2, 5, 10, 25 ng/L  MT 0, 26, 50, 100, 260, 500, 1000 ng/L | EE2 Mortality: >25 ng/L  EE2 VTG ↑♂ 2 ng/L  EE2 Histo sex ratio 1 ng/L (↑♀ all groups)  MT mortality 26 ng/L  MT VTG ↓26 to 500 ng/L; ↑1000 ng/L  MT sex ratio 26 ng/L | EE2 mortality >25 ng/L;  EE2 VTG 1 ng/L  EE2 Sex ratio <1 ng/L  MT mortality>1000 ng/L  MT VTG <26 ng/L  MT sex ratio <26 ng/L | (Örn et al. 2003) |
| *Sygnathus abaster, S. scovelli* (pipefish) | 45 d exposure of wild caught adults; 2 species; measured concentrations (Jec ELISA kit),  Video of courtship beh;  photos of oocyte and egg postures,  VTG levels (PCR). | 3 (low statistical power of study design) | EE2: 0, 3, 9, 18 ng/L (measured 3.34, 6.12, 26, 89 ng/L) | Courtship: 3 ng/L  Egg postures: 9 ng/L  ↑ ♂ plasma vtg: 3 ng/L | Courtship: <3 ng/L  Egg postures: 3 ng/L  ♂ plasma vtg: <3 ng/L | (Sárria et al. 2013) |
| *Sygnathus abaster* (pipefish) | 6-month exposure of newborns. Static renewal (50% renewal every 2nd day). Measured concentrations (Jec ELISA kit),  Mortality, vertical distribution patterns up to 6 months; sexual behaviors, presence of brood pouch. | 1 | EE2: 0, 8, 12, 36 ng/L | Marsupium: 8 ng/L  Vert Dist: 8 ng/L  Sex ratio: 8 ng/L | <8 ng/L | (Sárria et al. 2011) |
| *Oryzia latipes* | 60 d exposure freshly hatched fish. Semistatic (75%/3d). 6-week recovery.  LSI, GSI; survival, sex ratio, aromatase & actin gene expression. | 1 | EE2: 0, 1, 10, 100 ng/L | Survival, sex ratio: 100 ng/L;  Growth: 100 ng/  LSI: 100 ng/L  GSI: 10 ng/L  Egg prodxn: 10 ng/L  Testis histology: 100 ng/L  Aromatase: 10 ng/L  Actin: 1 ng/L | Survival, sex ratio: 10 ng/L;  Growth: 10 ng/L  LSI: 10 ng/L  GSI: 1 ng/L  Egg prodxn: 1 ng/L  Testis histology: 10 ng/L  Aromatase: 1 ng/L  Actin: <1 ng/L | (Scholz and Gutzeit 2000) |
|  |  |  |  |  |  |  |

Table S1-5. Summary of studies and Klimisch score for Level 5 lifecycle studies of EE2 in fish.

| **Taxonomic Group/**  **Species name** | **Study Type** | **Klimisch Score** | **Conc’ns**  **Tested** | **Results**  Separate endpoints into **population-relevant (adverse)** and non-population-relevant (biomarker) or positive endpoint  **LOEC NOEC** | | **Reference** |
| --- | --- | --- | --- | --- | --- | --- |
| Rainbow trout | exposure of male F0 for 56 d starting at testicular development stage - followed for 1 ½ gen | 1 | 0.8, 8.3, 65 ng/L for 56 d measured concns | **↓ embryo survival F1 at 19dpf**  **= 0.8 ng/L**  **↓ blood 11-KT, ↑ blood lutenizing hormone**  **= 65 ng/L** | None, <0.8 ng/L lowest concn affected surv of F1 embryos | (Brown et al. 2007) ; (Brown et al. 2009) |
| Zebrafish | Exposed fert to ovary 42 dpf, or fert to reprod’n 118 dpf | 3 | 3 ng/L – only 1 exposure concn tested but it was measured | ↑ VTG 42 d  **↑ feminization 118 d**  **↓ fertilization 118 d**  **↓ egg production 118 d**  **= 3 ng/L** | None < 3 ng/L | (Fenske et al. 2005) |
| Roach (*Rutilis rutilis*) | Mesocosm exposure 147 days – not sure…  Also lab exposure of roach for feeding success study | 3 – wt of evidence – may use for species comparison - only one exposure concn tested, and it dropped over time | 27.8 ng/L dropped to 13.2 ng/L – only 1 exposure concn tested but it was measured  roach exposed for feeding study = 50 ng/L nominal | ↓ biomass roach  = 27.8 ng/L  ↓ feeding success  = 50 ng/L (nominal) | None | (Hallgren et al. 2014) |
| Zebrafish | Fert egg to 4 months | 2 – three exposure concns were tested, but only highest one was measured | Nominal 0.05, 0.5, 5 ng/L, top concn measured 5.58 ng/L | ↓ weight M  ↓ secondary sex char  ↑ spawning (M with ctrl F)  = 0.05 ng/L  **↑ proportion female**  **↓ fertility**  **= 0.5 ng/L**  ↓ courtship behaviour M  **↓ spawning**  ↑ weight M  **= 5.58 ng/L** | ↑ proportion female  ↓ fertility NOEC = 0.05 ng/L | (Larsen et al. 2008) |
| Fathead minnow | Multi generation whole lake dosing | 3 – only one concn - use for weight of evidence | 5-6 ng/L measured, only 1 concn | ↑ VTG in M  ↑ intersex in M  ↑ feminization of M  **↓ reproductive success**  **= 5-6 ng/L** | None  < 5 ng/L | (Kidd et al. 2007) |
| Guppy (*Poecilia reticulate*) | Flow thru, 108 d exposure, birth to adulthood | 1 | 10.5, 44 112 ng/L measured (10, 50, 200 ng/L nominal) | ↑ GSI M  ↑ sperm count M  = 10.5 ng/L  **↑ Proportion F**  ↓ GSI M  ↓ M colouration  ↓ courtship behaviour M  ↑ body size M  **↓ # sired offspring when competing w unexposed M**  **=112 ng/L** | ↑ Proportion F  ↓ courtship behaviour M  ↓ # sired offspring NOEC=44 ng/L | (Kristensen et al. 2005) |
| Fathead minnow | Fert egg to adult 150 dph exposure, flow thru | 2 – lowest two concns not measured | 5 concns, top three measured, lowest 2 nominal: 0.32, 0.96 ng/L nominal, 3.54, 9.55, 22.7 ng/L measured | **↓ fertilization success**  **↓ proportion M**  ↑ egg production  ↑ size F  **= 0.32 ng/L**  ↓ secondary sex char M  = 0.96 ng/L  **↓ reproduction (to zero)**  ↑ ovipositor size  ↓ GSI F  **= 3.5 ng/L**  ↑ LSI = 9.6 ng/L  ↓ growth = 23 ng/L | No LOECS for fert success or % F as lowest concn caused effects  ↓ fertilization success and % F NOEC <0.32 ng/L  ↓ total viable eggs overallNOEC = 0.96 ng/L | (Parrott and Blunt 2005) |
| Mummichog (*Fundulus heteroclitus*) | Egg to 61 weeks | 2 – four concns nominal only, static renewal | 0.1, 1, 10, 100 ng/L static renewal daily, nominal only | ↓ time to hatch Fo  ↑ proportion vitellogenic oocytes  = 10 ng/L  **↓ hatch success F0**  ↑ length at hatch F0  ↑ survival F0 over time  ↓ vertebral abnormalities F0  **↑ proportion F by gonad histo**  ↑ secondary sex char F  ↓ CF, GSI, LSI in F and M  **= 100 ng/L** | ↓ hatch success F0 NOEC = 10 ng/L | (Peters et al. 2010) |
| Sand Goby (*Pomatoschistus minutus*, Pallas) | 7 months flow thru exposure of immature fish to maturity and breeding -EE2 used as +ve ctrl | 3 – only one exposure concn as EE2 was +ve ctrl in this expt, EE2 concn was not measured – may use for species comparisons | 6 ng/L nominal, not measured, only one concn | **↓ male maturation**  **↓ male reproductive behaviour**  ↑ZRP & VTG mRNA expression in liver  **↓ fecundity F**  **↓ egg fertility (by 90 %)**  ↓ weight, length, GSI, Seminal Vessicle SI, Urogenital papilla length, M  ↓ sex char fin colouration M  **= 6 ng/L** | None < 6 ng/L  ↓ egg fertility | (Robinson et al. 2003) |
| Zebrafish | Egg to adult 177 dpf, F2 exposed to higher concns til 162 dpf, flow thru, 2 reps per trt | 1 | 0.05, 0.31, 1.1, 9.2 ng/L measured (0.05, 0.28, 1.7, 10 ng/L nominal)  F2 exposed to 0.09, 0.36, 2.0 ng/L measured | F1 ↓ growth  **75 d LOEC = 1.1 ng/L**  **F1 ↓ time to sex’l maturity**  **↓ egg/f/day**  **↓ fert success**  **75 d and 177 d LOECs = 1.1 ng/L**  **↑ time to first spawning**  **= 9.3 ng/L**  **Very little recovery of reprod’n exposed 177 dpf then 3 months clean water –**  F2 ↓ growth  **↓onset spawning**  **↓ fecundity**  **↓ fertility**  **F2 to 162 d = 2.0 ng/L**  **From other studies data not shown –**  **4 d LC50 = 1.7 mg/L, 28 d LC50 = 100ng/L** | F1 ↓ egg/f/day  ↓ fert success  NOEC = 0.31 ng/L | (Schäfers et al. 2007) |
| Zebrafish | exposure 2 dph til 3 months, daily static renewal -followed by 3 month recovery in clean water | 2 - three concns, nominal only, static renewal | Nominal only, 3 concns, 0.4, 2, 10 ng/L  Static renewal | **↓ # eggs/tank**  **↓ % viable eggs**  **= 0.4 ng/L**  **90 dph ↓ survival**  **↑ Female sex ratio**  180 dph ↑ malformed sperm ducts  ↓ # spermatozoa  ↓ milt volume  **= 2.0 ng/L**  90 dph ↓ weight, ↓ length, ↑ condition factor  ↑ VTG 28 dph  = 10 ng/L  Interesting breeding study - M replaced w control M for 21 d = 2, 10 ng/L still **no** **egg production**, 0.4 recovered, but still **fewer eggs compared to ctrls** | None as repro effects seen at lowest concn tested so NOEC < 0.4 ng/L (nominal) | (Xu et al. 2008) |
| Chinese rare minnow (*Gobiocypris rarus*) | Multi-gen F0, F1, F2 assessed as eggs - static-renewal til 10 dph then flow thru | 1 | Measured concns every 2 wks 0.18, 0.91, 3.61, 13.6, 55.8 ng/L - (nominal 0.2, 1, 4, 16) | F0 180 dph ↓ GSI M  ↑VTG M plasma  **↑ proportion Female F0**  ↑ ovo-testis in M F0  **↓ fertility F0**  **F1 ↓ Males (no phenotypic males)**  **↑ proportion Female F1**  **↓ Female F1 repro w unexposed males (no repro in 21 d)**  **↓ survival F1 60 and 180 dph**  **↑ deformities F1**  ↓ GSI Females F1 240 dph  ↑ LSI, RSI, VTG Males F1 240 dph  ↑ testis-ova in M F1  **All LOEC = 0.18 ng/L measured**  **F0 ↑ deformities fry**  ↓ weight, ↑ LSI, ↓ Renal-SI M at 180 dph  ↑ VTG plasma F F0 180 dph  ↑ degeneration ovaries (histo) F F0 180 dph  **F1 ↓ swim up success**  ↑ LSI, RSI, VTG of F at 240 dph  **LOEC = 0.91 ng/L (measured)** | None – as repro effects seen in F0 at lowest concn tested↑ proportion Female F0  ↓ fertility F0  NOEC = <0.18 ng/L (meas) | (Zha et al. 2008b) |
| CONT …Chinese rare minnow (*Gobiocypris rarus*) |  |  |  | **F0↓ survival**  ↓ Length, weight F0 at 180 dph  ↑ LSI Female F0 at 180 dph  **LOEC = 3.61 ng/L (measured)**  **F0↓ survival at swim-up**  ↓ length, weight F0 at 90 dph and 120 dph  ↓ GSI, LSI, RSI at 120 dph  **LOEC 13.6 ng/L measured** |  |  |
| Fathead minnow | Flow thru F0 exposed from egg to 301 dph, F1 raised to 28 dph | 1 | 0.16, 0.76, 2.8, 12, 47 ng/l measured - (0.2, 1, 4, 16, 64 ng/L nominal) | F1 ↓ larval length, weight at 28 dph  **LOEC = 0.16 ng/L (meas)**  **F0 ↓ egg production (to zero)**  ↓ juvenile length F0  ↓ sex char Males at 301 dph  **↑ proportion Female at 56 dph**  ↓ testic tissue M at 172 dph  ↑ ovo-testis at 56 and 172 dph  F1 gonad histo affected 28 dph  **LOEC = 2.8 ng/L measured**  ↑ F0 VTG plasma 172 dph  ↓ F0 larval length  **LOEC = 12 meas ng/L**  **F0 ↓ embryo-larval and juvenile survival**  **LOEC = 47 ng/L (meas)** | F0 ↓ egg production  NOEC = 0.76 ng/L (meas) | (Länge et al. 2001) |
| Zebrafish | Breeding adult F0 exposed 40 d, F1 exposed entire life 210 dpf, breeding assessed for 10 d, F2 assessed at 100 hpf | 1 | 0.5, 4.5, 50 ng/L lowest two concns measured (0.5, 5, 50 nominal) | F0 ↑ VTG ↓ 11-KT in blood of M at 40 d exposure  **F1 ↓ fert success (still lower even after 5 months depur’n)**  **0.5 ng/L (meas)**  **F1 ↑ proportion F (no phenotypic males)**  **↓ sperm expression M (none)**  **↓ fertilization of eggs (none)**  ↓ normal testes in M (none) by histo  ↑ % w non-differentiated gonads (43 %), w ↓ VTG vs real F with differentiated F gonads (but had natural M spawning behav)  ↑ malformations ovarian and sperm ducts  Interesting – mated F 5 ng/L w 2 ctrl males – ↑ egg prodn but still **↓ fert of eggs and ↓surv of eggs** to 14 hpf vs ctrls – EE2 males competed to fert w ctrl males  4.5 ng/L (measured)  **F0 ↓ survival**, HC, GSI  **↓ spawning (none)**  **↑ deformities**  **50 ng/l (nominal)** | No NOEC as repro effects in F1 seen at lowest concn tested  F1 ↓ fert success  NOEC < 0.5 ng/L (meas) | (Nash et al. 2004) |
| Fathead minnow | Mesocosm F0, F1 for 127 d then F1 into lab for 114 d, F2 surv | 3 -Spiked once per day into ms’cosm and aquaria…huge variation in EE2 meas’d concns – | 3.2, 5.3, 10.9 ng/L (meas) in mesocosms and in lab (not sure if lab concns were same as ms’cosm or were 4.95 and 10.8 ng/L) |  |  | (Schwindt et al. 2014) |
| Zebrafish | Fert egg to adult, to juvenile 75 dpf gonad diff stage, or 42-75 dpf exposure | 2 ( or 3?) – no meas’d concns – no details of any fish exposuremethods given at all | NO Measured concs -Nominal 0.05, 0.28, 1.7, 10 ng/L flow thru n=2 reps | Fert egg to adult exposure:  ↑ VTG  ↓ juv growth 0-75 dpf  **↑ time to spawn**  ↓ mating behaviour  **↓ eggs/female**  **↓ fertilization success**  **LOEC = 1.67 ng/L (meas)** | ↓ eggs/female  ↓ fertilization success  NOEC = 0.28 ng/L | (Segner et al. 2003a)  review of IDEA project – no methods given |
| Zebrafish | Flow thru egg to 8 mos, and F1 offspring surv, 2 reps per trt | 1 but many endpoints left out ln, wt, cf, egg prod’n | 0.19, 0.24, 1 ng/L measured 2 times during exposure (0.5, 1, 2 nom) | F0 ↑ length Males  **F0 ↑ proportion males ?? only at lowest concn (not dose-related)**  **F1 ↑ egg mortality between 8-24 hpf, deformities 8 hpf**  **= 0.19 ng/L meas, 0.5 nominal**  F0 ↑ VTG m RNA in Male (parental generation)  **F1↑ % abnormal eggs**  **= 1 ng/L meas, 2 nominal** | No NOEC as lowest concn tested affected reprod’n  F1 ↑ egg mortality  NOEC < 0.19 ng/L (meas) | (Soares et al. 2009) |
| Japanese Medaka | 4 to 6 month exposure of M and F then mated w unexposed | 2 (or 3?) – no measured concns, no solv ctrl, static renewal 3 x per week – may use for species comparisons | 0.2, 2, 10 nominal, static renewal 3 x per week, no solv ctrl | ↑ Males approaches to F  ↑ % fert eggs when exposed M mated w ctrl F  = 0.2 ng/L nominal  **↑intersex males = 2 ng/L**  ↓ copulatory activity  **↓ breeding grps w fertilized eggs**  ↑ % fish w mixed secondary sex char  **= 10 ng/L nominal** | ↓ breeding grps w fertilized eggs  NOEC = 2 ng/L (nominal) | (Balch et al. 2004; Stokes et al. 2004) |

Table S1-6. Data used for Figure S1-1 Species Sensitivity Distributions for EE2 lifecycle studies in fish.

| **Species** | **Rank for NOEC** | **NOEC ng/L** | **Rank for LOEC** | **LOEC ng/L** | **Ratio of LOEC to NOEC** | **Reference(s)** |
| --- | --- | --- | --- | --- | --- | --- |
| Chinese Rare Minnow | 1 | 0.09 EST | 1 | 0.18 |  | (Zha et al. 2008b) |
| Rainbow Trout | 3 | 0.4 EST | 2 | 0.8 |  | (Brown et al. 2007) |
| Zebrafish mean | 2 | 0.213333 | 3 | 1.0514 | 5 | mean of 3 studies for NOEC (Segner et al. 2003b); (Schäfers et al. 2007); (Larsen et al. 2008); & 7 studies for LOEC (Segner et al. 2003b); (Nash et al. 2004); (Fenske et al. 2005); (Schäfers et al. 2007); (Larsen et al. 2008); (Xu et al. 2008); (Soares et al. 2009) |
| Fathead Minnow mean | 4 | 0.86 | 4 | 3.15 | 3.7 | mean of 2 studies for NOEC and LOEC (Länge et al. 2001); (Parrott and Blunt 2005) |
| Sand Goby*  Klimisch 3 study | 6 | 3.0 EST | 5 | 6 |  | (Robinson et al. 2003) |
| Japanese Medaka | 5 | 2.0 | 6 | 10 | 5 | (Balch et al. 2004) |
| Estuarine Mummichog | 7 | 10 | 7 | 100 | 10 | (Peters et al. 2010) |
| Guppy | 8 | 44 | 8 | 112 | 2.5 | (Kristensen et al. 2005) |

EST = Estimated NOEC as ½ of LOEC

**References Cited**

Andersen L, Holbech H, Gessbo Å, Norrgren L, Petersen GI. 2003. Effects of exposure to 17α-ethinylestradiol during early development on sexual differentiation and induction of vitellogenin in zebrafish (danio rerio). Comparative Biochemistry and Physiology - C Toxicology and Pharmacology 134:365-374.

Anderson PD, Johnson AC, Pfeiffer D, Caldwell DJ, Hannah R, Mastrocco F, et al. 2012. Endocrine disruption due to estrogens derived from humans predicted to be low in the majority of u.S. Surface waters. Environmental Toxicology and Chemistry 31:1407-1415.

Balch GC, Mackenzie CA, Metcalfe CD. 2004. Alterations to gonadal development and reproductive success in japanese medaka (oryzias latipes) exposed to 17α-ethinylestradiol. Environmental Toxicology and Chemistry 23:782-791.

Bhandari RK, Vom Saal FS, Tillitt DE. 2015. Transgenerational effects from early developmental exposures to bisphenol a or 17α-ethinylestradiol in medaka, oryzias latipes. Scientific Reports 5.

Brown KH, Schultz IR, Nagler JJ. 2007. Reduced embryonic survival in rainbow trout resulting from paternal exposure to the environmental estrogen 17α-ethynylestradiol during late sexual maturation. Reproduction 134:659-666.

Brown KH, Schultz IR, Cloud JG, Nagler JJ. 2008. Aneuploid sperm formation in rainbow trout exposed to the environmental estrogen 17α-ethynylestradiol. Proceedings of the National Academy of Sciences of the United States of America 105:19786-19791.

Brown KH, Schultz IR, Nagler JJ. 2009. Lack of a heritable reproductive defect in the offspring of male rainbow trout exposed to the environmental estrogen 17α-ethynylestradiol. Aquatic Toxicology 91:71-74.

Caldwell DJ, Mastrocco F, Hutchinson TH, Länge R, Heijerick D, Janssen C, et al. 2008. Derivation of an aquatic predicted no-effect concentration for the synthetic hormone, 17α-ethinyl estradiol. Environmental Science and Technology 42:7046-7054.

Caldwell DJ, Mastrocco F, Anderson PD, Länge R, Sumpter JP. 2012. Predicted-no-effect concentrations for the steroid estrogens estrone, 17β-estradiol, estriol, and 17α-ethinylestradiol. Environmental Toxicology and Chemistry 31:1396-1406.

Coe TS, Hamilton PB, Hodgson D, Paull GC, Stevens JR, Sumner K, et al. 2008. An environmental estrogen alters reproductive hierarchies, disrupting sexual selection in group-spawning fish. Environmental Science and Technology 42:5020-5025.

Coe TS, Söffker MK, Filby AL, Hodgson D, Tyler CR. 2010. Impacts of early life exposure to estrogen on subsequent breeding behavior and reproductive success in zebrafish. Environmental Science and Technology 44:6481-6487.

Depiereux S, Liagre M, Danis L, De Meulder B, Depiereux E, Segner H, et al. 2014. Intersex occurrence in rainbow trout (oncorhynchus mykiss) male fry chronically exposed to ethynylestradiol. PLoS ONE 9.

Desbrow C, Routledge EJ, Brighty GC, Sumpter JP, Waldock M. 1998. Identification of estrogenic chemicals in stw effluent. 1. Chemical fractionation and in vitro biological screening. Environmental Science and Technology 32:1549-1558.

Desprez D, Mélard C, Philippart JC. 1995. Production of a high percentage of male offspring with 17α-ethynylestradiol sex-reversed oreochromis aureus. Ii. Comparative reproductive biology of females and f2 pseudofemales and large-scale production of male progeny. Aquaculture 130:35-41.

Dzieweczynski TL, Hentz KB, Logan B, Hebert OL. 2014. Chronic exposure to 17α-ethinylestradiol reduces behavioral consistency in male siamese fighting fish. Behaviour 151:633-651.

Dzieweczynski TL, Forrette LM. 2015. Timescale effects of 17α-ethinylestradiol on behavioral consistency in male threespine stickleback. Acta Ethologica 18:137-144.

Endrikat J, Müller U, Düsterberg B. 1997. A twelve-month comparative clinical investigation of two low-dose oral contraceptives containing 20 μg ethinylestradiol/75 μg gestodene and 30 μg ethinylestradiol/75 μg gestodene, with respect to efficacy, cycle control, and tolerance. Contraception 55:131-137.

Farmer JL, Orlando EF. 2012. Creating females? Developmental effects of 17α-ethynylestradiol on the mangrove rivulus' ovotestis. Integrative and Comparative Biology 52:769-780.

Fenske M, Maack G, Schäfers C, Segner H. 2005. An environmentally relevant concentration of estrogen induces arrest of male gonad development in zebrafish, danio rerio. Environmental Toxicology and Chemistry 24:1088-1098.

Filby AL, Thorpe KL, Maack G, Tyler CR. 2007. Gene expression profiles revealing the mechanisms of anti-androgen- and estrogen-induced feminization in fish. Aquatic Toxicology 81:219-231.

Filby AL, Paull GC, Searle F, Ortiz-Zarragoitia M, Tyler CR. 2012. Environmental estrogen-induced alterations of male aggression and dominance hierarchies in fish: A mechanistic analysis. Environmental Science & Technology 46:3472-3479.

Fuzzen MLM, Bennett CJ, Tetreault GR, McMaster ME, Servos MR. 2015. Severe intersex is predictive of poor fertilization success in populations of rainbow darter (etheostoma caeruleum). Aquatic Toxicology 160:106-116.

Hallgren P, Nicolle A, Hansson LA, Brönmark C, Nikoleris L, Hyder M, et al. 2014. Synthetic estrogen directly affects fish biomass and may indirectly disrupt aquatic food webs. Environmental Toxicology and Chemistry 33:930-936.

Hamilton PB, Nicol E, De-Bastos ESR, Williams RJ, Sumpter JP, Jobling S, et al. 2014. Populations of a cyprinid fish are self-sustaining despite widespread feminization of males. BMC biology 12.

Hannah R, D'Aco VJ, Anderson PD, Buzby ME, Caldwell DJ, Cunningham VL, et al. 2009. Exposure assessment of 17α-ethinylestradiol in surface waters of the united states and europe. Environmental Toxicology and Chemistry 28:2725-2732.

Harries JE, Sheahan DA, Jobling S, Matthiessen P, Neall P, Routledge EJ, et al. 1996. A survey of estrogenic activity in united kingdom inland waters. Environmental Toxicology and Chemistry 15:1993-2002.

Harris CA, Hamilton PB, Runnalls TJ, Vinciotti V, Henshaw A, Hodgson D, et al. 2011. The consequences of feminization in breeding groups of wild fish. Environmental Health Perspectives 119:306-311.

Jobling S, Tyler CR. 2003. Endocrine disruption in wild freshwater fish. Pure and Applied Chemistry 75:2219-2234.

Jobling S, Williams R, Johnson A, Taylor A, Gross-Sorokin M, Nolan M, et al. 2006. Predicted exposures to steroid estrogens in u.K. Rivers correlate with widespread sexual disruption in wild fish populations. Environmental Health Perspectives 114:32-39.

Johnson AC, Dumont E, Williams RJ, Oldenkamp R, Cisowska I, Sumpter JP. 2013. Do concentrations of ethinylestradiol, estradiol, and diclofenac in european rivers exceed proposed eu environmental quality standards? Environmental Science and Technology 47:12297-12304.

Kidd KA, Blanchfield PJ, Mills KH, Palace VP, Evans RE, Lazorchak JM, et al. 2007. Collapse of a fish population after exposure to a synthetic estrogen. Proceedings of the National Academy of Sciences of the United States of America 104:8897-8901.

Kime DE, Nash JP, Scott AP. 1999. Vitellogenesis as a biomarker of reproductive disruption by xenobiotics. Aquaculture 177:345-352.

Kirby MF, Allen YT, Dyer RA, Feist SW, Katsiadaki I, Matthiessen P, et al. 2004. Surveys of plasma vitellogenin and intersex in male flounder (platichthys flesus) as measures of endocrine disruption by estrogenic contamination in united kingdom estuaries: Temporal trends, 1996 to 2001. Environmental Toxicology and Chemistry 23:748-758.

Klimisch HJ, Andreae M, Tillmann U. 1997. A systematic approach for evaluating the quality of experimental toxicological and ecotoxicological data. Regul Toxicol Pharmacol 25.

Koger CS, Teh SJ, Hinton DE. 2000. Determining the sensitive developmental stages of intersex induction in medaka (oryzias latipes) exposed to 17β-estradiol or testosterone. Marine Environmental Research 50:201-206.

Kristensen T, Baatrup E, Bayley M. 2005. 17α-ethinylestradiol reduces the competitive reproductive fitness of the male guppy (poecilia reticulata). Biology of Reproduction 72:150-156.

Lange A, Katsu Y, Miyagawa S, Ogino Y, Urushitani H, Kobayashi T, et al. 2012. Comparative responsiveness to natural and synthetic estrogens of fish species commonly used in the laboratory and field monitoring. Aquatic Toxicology 109:250-258.

Länge R, Hutchinson TH, Croudace CP, Siegmund F, Schweinfurth H, Hampe P, et al. 2001. Effects of the synthetic estrogen 17α-ethinylestradiol on the life-cycle of the fathead minnow (pimephales promelas). Environmental Toxicology and Chemistry 20:1216-1227.

Larsen MG, Hansen KB, Henriksen PG, Baatrup E. 2008. Male zebrafish (danio rerio) courtship behaviour resists the feminising effects of 17α-ethinyloestradiol-morphological sexual characteristics do not. Aquatic Toxicology 87:234-244.

Lee PY, Lin CY, Chen TH. 2014. Environmentally relevant exposure of 17α-ethinylestradiol impairs spawning and reproductive behavior in the brackish medaka oryzias melastigma. Marine Pollution Bulletin 85:338-343.

Leet JK, Sassman S, Amberg JJ, Olmstead AW, Lee LS, Ankley GT, et al. 2015. Environmental hormones and their impacts on sex differentiation in fathead minnows. Aquatic Toxicology 158:98-107.

Maack G, Segner H. 2004. Life-stage-dependent sensitivity of zebrafish (danio rerio) to estrogen exposure. Comparative Biochemistry and Physiology - C Toxicology and Pharmacology 139:47-55.

McGee MR, Julius ML, Vajda AM, Norris DO, Barber LB, Schoenfuss HL. 2009. Predator avoidance performance of larval fathead minnows (*pimephales promelas*) following short-term exposure to estrogen mixtures. Aquatic Toxicology 91:355-361.

Metcalfe CD, Metcalfe TL, Kiparissis Y, Koenig BG, Khan C, Hughes RJ, et al. 2001. Estrogenic potency of chemicals detected in sewage treatment plant effluents as determined by in vivo assays with japanese medaka (oryzias latipes). Environmental Toxicology and Chemistry 20:297-308.

Miller HD, Clark BW, Hinton DE, Whitehead A, Martin S, Kwok KW, et al. 2012. Anchoring ethinylestradiol induced gene expression changes with testicular morphology and reproductive function in the medaka. PLoS ONE 7.

Mills LJ, Gutjahr-Gobell RE, Horowitz DB, Denslow ND, Chow MC, Zaroogian GE. 2003. Relationship between reproductive success and male plasma vitellogenin concentrations in cunner, tautogolabrus adspersus. Environmental health perspectives 111:93-100.

Mills LJ, Gutjahr-Gobell RE, Zaroogian GE, Horowitz DB, Laws SC. 2014. Modulation of aromatase activity as a mode of action for endocrine disrupting chemicals in a marine fish. Aquatic Toxicology 147:140-150.

Montgomery TM, Brown AC, Gendelman HK, Ota M, Clotfelter ED. 2014. Exposure to 17α-ethinylestradiol decreases motility and atp in sperm of male fighting fish betta splendens. Environmental Toxicology 29:243-252.

Nash JP, Kime DE, Van der Ven LTM, Wester PW, Brion F, Maack G, et al. 2004. Long-term exposure to environmental concentrations of the pharmaceutical ethynylestradiol causes reproductive failure in fish. Environmental Health Perspectives 112:1725-1733.

OECD. 2012a. Guidance document on standardised test guidelines for evaluating chemicals for endocrine disruption. (Series on Testing and Assessment).

OECD. 2012b. Oecd conceptual framework for testing and assessment of endocrine disrupters.

Örn S, Holbech H, Madsen TH, Norrgren L, Petersen GI. 2003. Gonad development and vitellogenin production in zebrafish (danio rerio) exposed to ethinylestradiol and methyltestosterone. Aquatic Toxicology 65:397-411.

Örn S, Yamani S, Norrgren L. 2006. Comparison of vitellogenin induction, sex ratio, and gonad morphology between zebrafish and japanese medaka after exposure to 17α- ethinylestradiol and 17β-trenbolone. Arch Environ Contam Toxicol 51:237-243.

Oropesa AL, Jiménez B, Gil MC, Osswald J, Fallola C, Pula HJ, et al. 2014. Histological alterations in the structure of the testis in tench (tinca tinca) after exposure to 17 alpha-ethynylestradiol. Environmental Toxicology 29:1182-1192.

Oropesa AL, Martín-Hidalgo D, Fallola C, Gil MC. 2015. Effects of exposure to 17-alpha-ethynylestradiol on sperm quality of tench (tinca tinca). Ecotoxicology and Environmental Safety 120:318-325.

Ortiz-Zarragoitia M, Trant JM, Cajaraville MP. 2006. Effects of dibutylphthalate and ethynylestradiol on liver peroxisomes, reproduction, and development of zebrafish (danio rerio). Environmental Toxicology and Chemistry 25:2394-2404.

Palace VP, Evans RE, Wautier K, Baron C, Vandenbyllardt L, Vandersteen W, et al. 2002. Induction of vitellogenin and histological effects in wild fathead minnows from a lake experimentally treated with the synthetic estrogen, ethynylestradiol. Water Quality Research Journal of Canada 37:637-650.

Palace VP, Wautier KG, Evans RE, Blanchfield PJ, Mills KH, Chalanchuk SM, et al. 2006. Biochemical and histopathological effects in pearl dace (margariscus margarita) chronically exposed to a synthetic estrogen in a whole lake experiment. Environmental Toxicology and Chemistry 25:1114-1125.

Palace VP, Evans RE, Wautier KG, Mills KH, Blanchfield PJ, Park BJ, et al. 2009. Interspecies differences in biochemical, histopathological, and population responses in four wild fish species exposed to ethynylestradiol added to a whole lake. Canadian Journal of Fisheries and Aquatic Sciences 66:1920-1935.

Parrott JL, Blunt BR. 2005. Life-cycle exposure of fathead minnows (pimephales promelas) to an ethinylestradiol concentration below 1 ng/l reduces egg fertilization success and demasculinizes males. Environmental Toxicology 20:131-141.

Pawlowski S, Van Aerle R, Tyler CR, Braunbeck T. 2004. Effects of 17α-ethinylestradiol in a fathead minnow (pimephales promelas) gonadal recrudescence assay. Ecotoxicology and Environmental Safety 57:330-345.

Pérez MR, Fernandino JI, Carriquiriborde P, Somoza GM. 2012. Feminization and altered gonadal gene expression profile by ethinylestradiol exposure to pejerrey, odontesthes bonariensis, a south american teleost fish. Environmental Toxicology and Chemistry 31:941-946.

Peters REM, Courtenay SC, Hewitt LM, MacLatchy DL. 2010. Effects of 17α-ethynylestradiol on early-life development, sex differentiation and vitellogenin induction in mummichog (fundulus heteroclitus). Marine Environmental Research 69:178-186.

Racz L, Goel RK. 2010. Fate and removal of estrogens in municipal wastewater. Journal of Environmental Monitoring 12:58-70.

Robinson CD, Brown E, Craft JA, Davies IM, Moffat CF, Pirie D, et al. 2003. Effects of sewage effluent and ethynyl oestradiol upon molecular markers of oestrogenic exposure, maturation and reproductive success in the sand goby (pomatoschistus minutus, pallas). Aquatic Toxicology 62:119-134.

Salierno JD, Kane AS. 2009. 17α-ethinylestradiol alters reproductive behaviors, circulating hormones, and sexual morphology in male fathead minnows (pimephales promelas). Environmental Toxicology and Chemistry 28:953-961.

Sárria MP, Santos MM, Reis-Henriques MA, Vieira NM, Monteiro NM. 2011. Drifting towards the surface: A shift in newborn pipefish's vertical distribution when exposed to the synthetic steroid ethinylestradiol. Chemosphere 84:618-624.

Sárria MP, Santos MM, Castro LFC, Vieira NM, Monteiro NM. 2013. Estrogenic chemical effects are independent from the degree of sex role reversal in pipefish. Journal of Hazardous Materials 263:746-753.

Schäfers C, Teigeler M, Wenzel A, Maack G, Fenske M, Segner H. 2007. Concentration- and time-dependent effects of the synthetic estrogen, 17α-ethinylestradiol, on reproductive capabilities of the zebrafish, danio rerio. Journal of Toxicology and Environmental Health - Part A: Current Issues 70:768-779.

Schneider K, Schwarz M, Burkholder I, Kopp-Schneider A, Edler L, Kinsner-Ovaskainen A, et al. 2009. "Toxrtool", a new tool to assess the reliability of toxicological data. Toxicology Letters 189:138-144.

Scholz S, Gutzeit HO. 2000. 17-α-ethinylestradiol affects reproduction, sexual differentiation and aromatase gene expression of the medaka (oryzias latipes). Aquatic Toxicology 50:363-373.

Schwindt AR, Winkelman DL, Keteles K, Murphy M, Vajda AM. 2014. An environmental oestrogen disrupts fish population dynamics through direct and transgenerational effects on survival and fecundity. Journal of Applied Ecology 51:582-591.

Schwindt AR. 2015. Parental effects of endocrine disrupting compounds in aquatic wildlife: Is there evidence of transgenerational inheritance? General and Comparative Endocrinology 219:152-164.

Segner H, Caroll K, Fenske M, Janssen CR, Maack G, Pascoe D, et al. 2003a. Identification of endocrine-disrupting effects in aquatic vertebrates and invertebrates: Report from the european idea project. Ecotoxicology and Environmental Safety 54:302-314.

Segner H, Navas JM, Schäfers C, Wenzel A. 2003b. Potencies of estrogenic compounds in in vitro screening assays and in life cycle tests with zebrafish in vivo. Ecotoxicology and Environmental Safety 54:315-322.

Seki M, Yokota H, Matsubara H, Tsuruda Y, Maeda M, Tadokoro H, et al. 2002. Effect of ethinylestradiol on the reproduction and induction of vitellogenin and testis-ova in medaka (oryzias latipes). Environmental Toxicology and Chemistry 21:1692-1698.

Silva P, Rocha MJ, Cruzeiro C, Malhão F, Reis B, Urbatzka R, et al. 2012. Testing the effects of ethinylestradiol and of an environmentally relevant mixture of xenoestrogens as found in the douro river (portugal) on the maturation of fish gonads-a stereological study using the zebrafish (danio rerio) as model. Aquatic Toxicology 124-125:1-10.

Soares J, Coimbra AM, Reis-Henriques MA, Monteiro NM, Vieira MN, Oliveira JMA, et al. 2009. Disruption of zebrafish (danio rerio) embryonic development after full life-cycle parental exposure to low levels of ethinylestradiol. Aquatic Toxicology 95:330-338.

Soares J, Castro LFC, Reis-Henriques MA, Monteiro NM, Santos MM. 2012. Zebrafish (danio rerio) life-cycle exposure to chronic low doses of ethinylestradiol modulates p53 gene transcription within the gonads, but not ner pathways. Ecotoxicology (London, England) 21:1513-1522.

Söffker M, Stevens JR, Tyler CR. 2012. Comparative breeding and behavioral responses to ethinylestradiol exposure in wild and laboratory maintained zebrafish (danio rerio) populations. Environmental Science and Technology 46:11377-11383.

Sohoni P, Sumpter JP. 1998. Several environmental oestrogens are also anti-androgens. Journal of Endocrinology 158:327-339.

Sridevi P, Chaitanya RK, Prathibha Y, Balakrishna SL, Dutta-Gupta A, Senthilkumaran B. 2015. Early exposure of 17α-ethynylestradiol and diethylstilbestrol induces morphological changes and alters ovarian steroidogenic pathway enzyme gene expression in catfish, clarias gariepinus. Environmental Toxicology 30:439-451.

Stokes EA, Lonergan W, Weber LP, Janz DM, Poznanski AA, Balch GC, et al. 2004. Decreased apoptosis in the forebrain of adult male medaka (oryzias latipes) after aqueous exposure to ethinylestradiol. Comparative Biochemistry and Physiology - C Toxicology and Pharmacology 138:163-167.

Sumpter JP, Jobling S. 2013. The occurrence, causes, and consequences of estrogens in the aquatic environment. Environmental Toxicology and Chemistry 32:249-251.

Temellini A, Giuliani L, Pacifici GM. 1991. Interindividual variability in the glucuronidation and sulphation of ethinyloestradiol in human liver. British Journal of Clinical Pharmacology 31:661-664.

Ternes TA, Stumpf M, Mueller J, Haberer K, Wilken RD, Servos M. 1999. Behavior and occurrence of estrogens in municipal sewage treatment plants — i. Investigations in germany, canada and brazil. Science of The Total Environment 225:81-90.

Tetreault GR, Bennett CJ, Shires K, Knight B, Servos MR, McMaster ME. 2011. Intersex and reproductive impairment of wild fish exposed to multiple municipal wastewater discharges. Aquatic Toxicology 104:278-290.

Tyler CR, Jobling S. 2008. Roach, sex, and gender-bending chemicals: The feminization of wild fish in english rivers. BioScience 58:1051-1059.

Van Aerle R, Pounds N, Hutchinson TH, Maddix S, Tyler CR. 2002. Window of sensitivity for the estrogenic effects of ethinylestradiol in early life-stages of fathead minnow, pimephales promelas. Ecotoxicology (London, England) 11:423-434.

Wang S, Rijk JCW, Besselink HT, Houtman R, Peijnenburg AACM, Brouwer A, et al. 2014. Extending an in vitro panel for estrogenicity testing: The added value of bioassays for measuring antiandrogenic activities and effects on steroidogenesis. Toxicological Sciences 141:78-89.

Warner KE, Jenkins JJ. 2007. Effects of 17alpha-ethinylestradiol and bisphenol a on vertebral development in the fathead minnow (pimephales promelas). Environmental toxicology and chemistry / SETAC 26:732-737.

Water-Framework-Directive. 2011. Alpha-ethinylestradiol eqs dossier.

Xu H, Yang J, Wang Y, Jiang Q, Chen H, Song H. 2008. Exposure to 17α-ethynylestradiol impairs reproductive functions of both male and female zebrafish (danio rerio). Aquatic Toxicology 88:1-8.

Ying GG, Kookana RS, Ru YJ. 2002. Occurrence and fate of hormone steroids in the environment. Environment International 28:545-551.

Zha J, Sun L, Spear PA, Wang Z. 2008a. Comparison of ethinylestradiol and nonylphenol effects on reproduction of chinese rare minnows (gobiocypris rarus). Ecotoxicology and Environmental Safety 71:390-399.

Zha J, Sun L, Zhou Y, Spear PA, Ma M, Wang Z. 2008b. Assessment of 17α-ethinylestradiol effects and underlying mechanisms in a continuous, multigeneration exposure of the chinese rare minnow (gobiocypris rarus). Toxicology and Applied Pharmacology 226:298-308.

Zhao Y, Hu J. 2012. Development of a molecular biomarker for detecting intersex after exposure of male medaka fish to synthetic estrogen. Environmental Toxicology and Chemistry 31:1765-1773.
