# Supplementary material for: Recommended Approaches to the Scientific Evaluation of Ecotoxicological Hazards and Risks of Endocrine-Active Substances
Source: Integr Environ Assess Manag. Author manuscript; Available in PMC 2018 Aug 1. (PMC6069525; doi:10.1002/ieam.1885)
Supplement: Supplement5 — S5 — Case Study Summary Trenbolone S5 — Trenbolone Table S5–5 S5 — Trenbolone Table S5–6 [file NIHMS1500348-supplement-Supplement5.docx]

**Supplemental Data S4**

**Draft Case Study for the Ecotoxicological Hazard and Risk Evaluation of Tributyltin (TBT)**

**L. Lagadic**^1^**, I. Katsiadaki**^2^**, R. Biever**^3^**, S. Dungey**^4^**, P.D. Guiney**^5^**, N. Karouna-Renier**^6^**,**

**J.P. Meador**^7^**, T. Schwarz**^2^

^1^Bayer AG, Crop Science Division, Environmental Safety, Alfred-Nobel-Straße 50, 40789 Monheim am Rhein, Germany.

^2^Centre for Environment, Fisheries and Aquaculture Science, Barrack Road, The Nothe, Weymouth, Dorset, DT4 8UB, UK.

^3^Smithers Viscient, 790 Main Street, Wareham, MA 02571, USA.

^5^University of Wisconsin-Madison, 777 Highland Avenue, Madison, WI 53705-2222, USA.

^4^Environment Agency, Red Kite House, Howbery Park, Wallingford, Oxfordshire OX10 8BD, UK.

^6^USGS Patuxent Wildlife Research Center, BARC East Bldg 308, 10300 Baltimore Avenue, Beltsville, MD 21042, USA.

^7^Ecotoxicology and Environmental Fish Health Program, Northwest Fisheries Science Center, NOAA, Seattle, WA 98112, USA.

1. **Overview of the Purpose of the Case Study**

**1.1. Uses of TBT compounds**

Historically, TBT compounds were widely used as biocides in anti-fouling products, parasite control products and wood preservatives. Regulatory controls have been implemented in many jurisdictions (*e.g.* under the International Maritime Organization’s Anti-Fouling Systems Convention), but some residual biocidal uses in these and other types of industrial and consumer products (including sports clothing) may still occur in some parts of the world. They are also used as chemical intermediates for the production of other organotins (*e.g*. dibutyltin stabilizers for PVC), and may therefore occur as unintentional impurities (*e.g.* OECD, 2007).

**1.2. Metabolism**

TBT can be metabolized sequentially by cytochrome P450 (CYP450) system enzymes to dibutyl- and monobutyltin (*e.g.* Cooke *et al.,* 2008; Strand *et al.,* 2009). Metabolic capabilities vary widely between species, including molluscs and mammals (*e.g.* Ohhira *et al.,* 2003, 2006a,b; Bartlett *et al.,* 2007; Oehlmann *et al.,* 2007; Yang *et al.,* 2009) and also between sexes in mammals (Ohhira *et al.,* 2006b).

**1.3. Potential exposure routes**

TBT has a degradation half-life of days to months in water and up to several years in sediment (*e.g.* ECHA, 2008). Aquatic organisms can be exposed to TBT *via* both the water column and ingestion of contaminated food (including sediment), although there are no data to suggest that biomagnification occurs in food webs. TBT is very bioaccumulative, with whole fish bioconcentration factors in the range 2 000 – 50 000 L/kg; the bioaccumulation potential in molluscs can be higher, but is generally similar to that for fish (*e.g.* ECHA, 2008; Meador, 2006). High levels of bioaccumulation in invertebrates and fish are believed to be due to a low capacity for metabolism/excretion and high rates of uptake.

Terrestrial organisms may also be exposed *via* TBT‑contaminated sediments (*e.g.* during flood events or disposal of sediment dredging), application of biocidal products and/or contaminated sewage sludge to soil, atmospheric deposition, and by ingestion of contaminated food or water (Antizar-Ladislao, 2008; Silva *et al.,* 2014).

1. **Methods**

A wide variety of aquatic taxa have been tested using TBT, as well as amphibians, birds and mammals. All reliable data reviewed for this case study have been considered.

**2.1. Literature search (regulatory, open and grey literature) and selection of data**

Around 160 references were selected from an initial list of approximately 965, in an attempt to capture relevant data from original studies on fish (45 references), molluscs (55 references) and other groups (60 references), including mammals; these were sorted according to type of effect. This was not intended to be a comprehensive review of the extensive TBT literature, so it is possible that some relevant studies were inadvertently omitted.

**2.2. Quality evaluation of relevant data**

TBT is a data-rich chemical with tests at various levels of biological organization but it does not have many standardised test guideline studies conducted using OECD protocols. Nevertheless, several full or partial life cycle tests are available for mammals, fish and several invertebrate groups. Data previously validated for regulatory reviews (*e.g*. EU, 2005; US EPA, 2003 & 2008) were assumed reliable, so are mostly reported as such and have not been systematically re-evaluated for the purpose of this analysis. Other ecotoxicity studies were quality checked and ranked using Klimisch scores (*e.g*. using the ToxRTool:<https://eurl-ecvam.jrc.ec.europa.eu/about-ecvam/archive-publications/toxrtool>). Only studies ranked as Klimisch 1 and 2 were used for the subsequent analysis (although a small number of papers ranked as Klimisch 3/4 were used as supporting information if their findings were verified by other studies). However, Klimisch scores do not necessarily apply to field studies, which were a major source of data. Therefore, expert judgment was used to evaluate the reliability/credibility of the studies. The same applies to histopathological investigations.

Many laboratory studies were performed using static or semi-static exposure regimes, and reported effect concentrations are frequently based on nominals due to a lack of analytical verification of the test concentrations over the whole study duration. TBT is adsorptive and test concentrations in non-flow-through studies are likely to be highly variable. Controls can also be affected by contaminated equipment. In addition, a variety of units have been used to express the TBT concentration. To aid comparisons between studies, concentrations should be expressed in a common unit (TBT). However, for simplicity, any concentrations reported as TBTO or tributyltin chloride (TBTCl) are assumed to be effectively the same as TBT, since the difference in molecular weight is small. Conversion to TBT has only been made when the unit is expressed in terms of tin (Sn), although this could be misleading due to the possible presence of other compounds such as triphenyltin and TBT metabolites (*e.g.* Bryan *et al.* (1993) reported that TBT accounted for only 44-48% of tissue organotin levels in a field study).

Other important limitations of the available data include wide separation between the LOEC and NOEC in some laboratory studies (very few report data in terms of ECx values), some poor methodological descriptions/statistical analyses, and uncertainties in the association between reported dissolved concentrations and observed effects in field studies (since the concentration at the time of adverse event initiation may be different, and organisms may accumulate TBT over a long period).

**3. Exposure assessment**

For the purposes of this analysis, measured environmental concentrations post-2008 were preferred for assessment. Regulatory monitoring performed in English and Welsh estuarine surface waters during 2012-2014 were selected to represent current aquatic levels in a region known to be previously contaminated (Environment Agency, pers. com., 2016). The data are influenced by a number of non-detects, but the following information is considered to be reasonably conservative: 90^th^ percentile: 0.5 ng/L; arithmetic mean: 0.3 ng/L; median: 0.2 ng/L; range: 0.1 – 8 ng/L (n = 269; two outliers were removed prior to the calculation). Disclaimer: This calculation is solely for the purposes of this evaluation and has not been endorsed by the original data supplier.

**4. Effect assessment**

**4.1. Organization according to the OECD Conceptual Framework**

As previously mentioned, only a few studies were performed according to standard test guidelines, which correspond to the OECD Conceptual Framework (OECD, 2012). Non-standard studies do not necessarily fit within the different levels of this framework. The studies with TBT are therefore organized according to the test design, including the exposure duration and portion of the life-cycle exposed, and to the type of biological responses. As EC_x_ were usually not given in and/or could not be deduced from the studies, endpoints are mainly reported as LOEC and/or NOEC.

**4.2. Level 1 – Summary of short-term *in vivo* toxicology data**

TBT has been evaluated in EPAs ToxCast^TM^ program (<http://epa.gov/ncct/toxcast/>). TBT was classified as being a “promiscuous” chemical as it showed activity in approximately 285 assays across 20 target families. This type of profile suggests that TBT will act across a number of toxic pathways potentially including endocrine and non-endocrine molecular initiating events (MIE).

The nuclear receptor family includes MIEs that are associated with endocrine and metabolism pathways. All of these assays utilize mammalian systems. However, it is assumed that there is interspecies conservation of the structural and functional aspects of each receptor, and thus one would expect these to be representative of non-mammalian species where the nuclear receptors are present. TBT activity in ER and AR assays generally occurred at levels in excess of the cytotoxicity limit. In addition, the EDSP21 Dashboard identified TBT as inactive for EAT (estrogen, androgen and thyroid) screens (<http://actor.epa.gov/edsp21/>). However, TBT activity in RXR and PPAR assays typically occurred at levels less than the cytotoxicity limit and at lower levels than most ER or AR assays. Thus, ToxCast^TM^ potentially identified endocrine MIEs through RXR and/or PPAR pathways.

**4.3. Level 2 – *In vitro* assays providing data about selected endocrine mechanisms/pathways**

***4.3.1. Mammals***

Studies using mammalian *in vitro* systems have examined interactions between TBT and nuclear receptors, and the effects of TBT on enzymes involved in steroidogenesis (Saitoh *et al.*, 2001a,b; McVey and Cooke, 2003; Cooke *et al.,* 2002; Heidrich *et al.,* 2001). Together, these studies initially suggested a possible inhibitory role for TBT in steroidogenesis as the primary means of endocrine disruption. However, Kanayama *et al.* (2005) found that TBT induced the transactivation function of RXR_α_ and PPAR_ϒ_ at lower concentrations than those causing aromatase inhibition and proposed that this receptor-based pathway is the more likely route for low-dose effects such as imposex in gastropods. Additional evidence for interaction of TBT with RXR and PPAR *via* direct ligand binding followed (Grun *et al.,* 2006; le Maire *et al.,* 2009), whilst multiple lines of *in vitro* evidence exist demonstrating the roles of RXR and PPAR in TBT-induced adipogenesis in mammals (Kanayama *et al.,* 2005; Grun *et al.,* 2006; Kirchner *et al.,* 2010; Yanik *et al.,* 2011; Belcher *et al.,* 2014). Further evidence for the critical role for RXR in mediating effects of TBT comes from *in vitro* studies of effects on thyroid hormone receptors (Sharan *et al.,* 2014). Given the importance of RXR in the negative transcriptional regulation of genes of the hypothalamic–pituitary axis by T3 (LaFlamme *et al.,* 2002), the effects of TBT on the thyroid axis may involve multiple nuclear receptor pathways.

***4.3.2. Fish***

Organotins, including TBT, are well known inhibitors of the hepatic microsomal CYP450 systems in a variety of fish species (review by Fent and Hunn, 1996), affecting protein transcription and enzyme activity. The isoform CYP450 1A1 (CYP1A or EROD) appears to be particularly sensitive to TBT exposure although in general, CYP450 inhibition occurs at levels that are close to cytotoxicity and as such it is difficult to establish whether the inhibitions observed constitute an additional mechanism of toxicity. More recently it has been established that not only phase I but also phase II metabolism enzymes are affected by TBT (Morcillo *et al.,* 2004). The effect of TBT on hormonal and biotransformation pathways using salmon hepatocytes has also confirmed the general inhibitory effect using gene expression patterns (VTG, ER, AR) and CYP-mediated enzyme activities as endpoints (Mortensen and Arukwe, 2009) when used at moderate to high doses. This consistent decrease (with time and TBT concentration) of the studied responses in cellular systems suggests a possible transcription inhibitory effect of TBT. The effect of TBT on other transcription factors such as RXR and PPAR in fish cell systems is surprisingly under-studied. Nevertheless, TBTO inhibited plaice (*Pleuronectes platessa*) PPAR_α_ and PPAR_β_ at 1 nM in transfection assays although it had no effect on PPAR_γ_ (Colliar *et al.,* 2011).

***4.3.3. Molluscs***

In molluscs, only a few *in vitro* assays with TBT reported on initial endocrine mechanisms (*i.e*. MIE). They concern transactivation assays using mammalian cells transfected with RXR from *Thais clavigera* (Urishitani *et al.,* 2013) and *Lymnaea stagnalis* (Boulahtouf *et al.,* 2015). These studies showed that TBT binds the ligand binding domain of RXR of these species, and its affinity is equivalent to that of 9-cis-RA, the natural ligand of RXR.

**4.4. Level 3 – *In vivo* assays providing data about single endocrine mechanisms/pathways**

***4.4.1. Mammals***

In mammals, *in vivo* exposure to TBT has been shown to cause reproductive and other apical effects, although early studies used relatively high exposure levels, at which the effects of TBT are unlikely to be through the RXR/PPAR pathway. Effects include a higher rate of pregnancy (Harazono *et al.* 1996). significantly lower body weights of fetuses and increase in the incidence of post-implantation loss (Harazono *et al.,* 1998), significant reduction of dam’s body weight at high concentrations (Cooke *et al.,* 2008), and increased body weight at low concentrations both for dams (Cooke *et al.,* 2008) and male mice (Zuo *et al.* 2009). Susceptibility to, and manifestation of, the antifertility effects of TBTCl vary with the gestational stage at the time of administration, whilst the variation in body weight responses appears to reflect both life stage during exposure and dose, with exposure *in utero* likely predisposing the animal to increased adipose mass as it ages and high dose exposures resulting in body weight loses. Several changes in transcription of adipogenesis mediators has also been reported (Grun *et al.* 2006).

Thyroid-related effects of TBT have been reported *in vivo* in mammals (*e.g.*, Decherf *et al.,* 2010; Sharan *et al.,* 2014). Thyrotropin-releasing hormone (TRH) production is controlled at the transcriptional level by T3 through TRs but also *via* RXR and PPAR_ϒ_ (LaFlamme *et al.,* 2002). Decherf *et al.* (2010) demonstrated that TBT significantly reduced T3-independent transcription but also abolished T3-dependent regulation, confirming the role of TBT as a T3 antagonist that had been reported *in vitro*. Although the exposure levels in this study were relatively high, the results lend important insights into the hypothalamic effects of TBT. Thyroid system effects in mice have been demonstrated at significantly lower exposure levels (Sharan *et al.,* 2014), concluding that TBT induces hypothyroidism by suppressing transcriptional activity of thyroid-responsive genes and inhibiting T3 binding to thyroid receptors, thereby preventing recruitment of co-activators and corepressors on the promoters of target genes.

***4.4.2. Fish***

TBT is toxic to fish and fish embryos with a number of pathways affected, including reduced CYP450 induction (Bentivegna *et al.* 1998), osteoblastic activity and calcium metabolism (Suzuki *et al.*, 2006), gonadal histopathology and thyroid disruption (Zhang *et al.,* 2007*,* 2009a,b), altered RXR_α_ expression and ectopic lipid accumulation in ovarian interstitial cells (Zhang *et al.*, 2011, 2013a,b), inhibition of VTG production in females (Lv *et al.,* 2009). The reported effects of TBT on aromatase (CYP19) are of particular interest since evidence is controversial; some studies reported increased CYP19b expression (Zhang *et al.,* 2013a,b) whereas some reported reduced activity, particularly in the brain (Kuhl and Brouwer, 2006; Tian *et al.*, 2015) but also in the ovary (Pereira *et al.,* 2011a,b). Although these differences could be due to the study design (duration, critical window of sexual differentiation), the species used, and the tissue studied, increased aromatase activity does not appear critical for TBT-induced masculinisation (Kuhl and Brouwer, 2006; McGinnis and Crivello, 2011), nor TBT exposure affected testosterone or estradiol concentrations (Pereira *et al.,* 2011a,b).

A series of studies with juvenile Atlantic salmon (*Salmo salar*) that were force-fed TBT alone and in combination with forskolin, reported a number of affected gene expression patterns, including CYP3a, CYP11b, CYP19a, SF-1, glucocorticoid receptor, ERα PXR, PPARs, glutathione-S-transferase (GST), ACOX 1, IL-b, TNFa, IFNγ, IFNα, Mx3, IGF-1, IL-10 and TGFb (Pavlikova *et al.,* 2010; Pavlikova and Arukwe, 2011; Kortner *et al.,* 2010). Since most effects observed after TBT exposure were modulated by forskolin exposure, these studies suggested that TBT may exert its endocrine, biotransformation and lipid peroxidation effects *via* the cAMP/PKA second messenger system.

Taken together, the fish studies suggest that the CYP19 modulating effects of TBT are not mediated through direct inhibition of CYP19 activity and that RXR and PPAR are central on the plethora of pathways affected by TBT exposures.

***4.4.3. Amphibians***

*Xenopus tropicalis* embryos showed developmental and survival effects when exposed to TBT at 50 to 400 ng/L in the frog embryo teratogenesis assay-*Xenopus* (FETAX) (Guo *et al*., 2010). The most common malformations were abnormal eyes and skin hypopigmentation, with increased time of exposure. Thyroid hormone is linked to eye development in *Xenopus* embryos. The authors suggest that the eye malformations and other malformations are linked to TBT exposure through binding with RXRs and that RXRs form heterodimers with the thyroid hormone receptors.

Shi *et al.* (2014) exposed *Xenopus laevis* to TBT in an Amphibian Metamorphosis Assay (AMA) (OECD TG 231) and a complete AMA (CAMA) where Nieuwoop and Faber (NF) stage 46 to stage 66 were exposed. They found TBT to have anti-thyroid activity in the AMA at concentrations of 12.5 to 200 ng/L based on decreased hind limb length in the absence of growth effects or overt toxicity, delayed development, and thyroid lesions. The CAMA confirmed developmental delays based on front limb emergence and total metamorphosis time; however, these effects were seen in the presence of decreased body length and weight at metamorphosis at 10 and 100 ng/L. The CAMA also found that the intersex and sex ratio increased in favour of males with increasing concentrations of TBT. Apical endpoints in *Xenopus* sp. metamorphosis and embryo eye development were affected by TBT concentrations as low as 12.5 ng/L (Shi *et al.*, 2014). Although these endpoints are regulated by thyroid hormones, it is unclear whether TBT acts directly on thyroid hormones, or indirectly through binding to RXRs.

***4.4.4. Molluscs and other invertebrates***

In the reproductive tissues of female dog whelk *N. lapillus*, RXR gene transcription is increased in the penis-forming area associated with the formation of a penis and/or vas deferens (Lima *et al.,* 2011). In this species, 9-cis-RA is as potent as TBT in inducing imposex, indicating that TBT toxicity in gastropods is mediated through the modulation of the RXR signaling pathways (Nishikawa *et al.,* 2004; Castro *et al.,* 2007). It should be noted that imposex was also induced by rosiglitazone, a PPAR agonist, suggesting that the heterodimer of RXR-PPAR is a critical pathway for this phenomenon, at least in *Nucella* (Pascoal *et al.,* 2013). Female prosobranch molluscs can develop imposex in laboratory exposures with LOECs of 1 to 83 ng/L (Abidli *et al.,* 2012, 2013; Gooding *et al.,* 2003).

Shell development in molluscs may be as sensitive as imposex and reproduction with a number of species exhibiting shell growth and development effects in aqueous concentrations ranging from 8 to 1000 ng TBT/L (Ruiz *et al.,* 1995; Leung *et al.,* 2006, 2007; Giusti *et al.,* 2013).

TBT may affect the endocrine system of insects. In an *in vivo* study on *Chironomus riparius*, an environmentally relevant concentration of TBT (1 ng/L) caused effects on many endocrine related genes, including up-regulation of the ecdysone receptor, ultraspiracle gene (the orthologue of RXR in insects), estrogen-related receptor, and E74 early ecdysone inducible gene, whilst the VTG gene remained unaffected (Morales *et al.,* 2013).

**4.5. Level 4 – *In vivo* assays providing data about multiple endocrine mechanisms and effects**

***4.5.1. Mammals and Birds***

Zuo *et al.* (2014) exposed male KM mice to TBT (by gavage) and examined effects on the pancreas, glucose homeostasis, and circulating steroid and thyroid hormone levels; all endpoints examined were affected. One of the first studies to examine the effects of *in utero* exposure to TBT on lipid homeostasis and adipogenesis was conducted by Grun *et al.* (2006). Histological examination demonstrated that TBT exposure caused a disorganization of hepatic and gonadal architecture in the pups at birth, and liver sections exhibited signs of steatosis whilst adipose mass in 10-week old TBT-treated males was significantly higher than in controls, although no overt increases in body mass were noted. White adipose tissue stroma cells from TBT-exposed mice showed increased adipogenic capacity and lipid accumulation, reduced osteogenic capacity, increased Fapb4 and PPAR_ϒ_ mRNA expression, decreased adipogenesis inhibitor Pref-1 mRNA, hypomethylation of the promoter/enhancer region of the Fapb4 locus, and an increased number of preadipocytes in the cells (Kirchner *et al.* 2010). This study provided the first evidence that *in utero* exposure to TBT counteracts osteogenesis and induces preferential differentiation of ADSCs into adipocytes. Effects of *in utero* exposure to TBT on fetal gonad morphology have been reported in Sprague–Dawley rats (Kishta *et al.,* 2007). Other studies reported reduction in body weight, early puberty and impaired estrous cyclicity in female mice exposed perinatally to TBT with no effects on circulating sex steroids (Si *et al.,* 2012), as well as a dramatic decrease in sperm counts and motility in male offspring with no or limited effects on intratesticular and serum hormone levels, suggesting that altered expression of receptors rather than hormone levels may be involved (Si *et al.,* 2013).

Although few studies of the effects of TBT have been conducted in birds, these have demonstrated some reproductive effects from exposure; these included reduced hatchability, increased embryo mortality, significantly reduced serum calcium values (Coenen *et al.,* 1992). This same study was repeated by five laboratories in an inter-laboratory comparison test (Schlatterer *et al.,* 1993), which reported similar findings: dose-related decreases in egg weight, egg production, fertility, hatching success and survival of 14 day-old. However, none of these studies in quail examined steroid hormone levels or any related genomic indicators from the HPG axis, nor were any effects on nuclear receptors investigated.

***4.5.2. Fish***

TBT can alter the sex ratio towards males in zebrafish (McAllister and Kime, 2003; Santos *et al.,* 2006) and Japanese flounder (*Paralichthys olivaceus*) (Shimasaki *et al.,* 2003). Zebrafish exposed for 70 days to 0.1 ng/L of TBT showed a male-biased population and produced a high incidence of sperm lacking ﬂagella. Both timing and duration of exposure was very important in the phenotypic responses. However, Lima *et al.* (2015) exposed zebrafish larvae from 5 days post-fertilization (dpf) up to 120 dpf to TBT via the diet, and found that sex ratios shifted towards females in contrast to other studies reported above. The expression of gonadal aromatase was unaffected but in female brain, TBT down-regulated CYP19a1b mRNA. There was also a brain-specific down-regulation of PPAR_γ_ in both males and females. This could be a strain difference or a concentration effect. Reduced sperm counts were also observed in guppies (*Poecilia reticulata*) (Haubruge *et al.,* 2000), whilst damage to epithelial cells of seminal ducts, and slight decrease in spermatozoa numbers was reported in mummichog (*Fundulus heteroclitus*) following TBT exposure (Mochida *et al.,* 2007). Growth effects in fish were also found at very low tissue concentrations. Shimasaki *et al.* (2003) reported a statistically significant decrease in body weight and length at 18 ng/g ww. Several studies examined effects of maternally transferred TBT using different routes of exposure including dietary (Shimasaki *et al.,* 2006; Nakayama *et al.,* 2005) and injection (Hano *et al.,* 2007). These studies support the conclusion that fish embryos are very sensitive to TBT and indicate that maternal transfer might be an important route of exposure.

***4.5.3. Molluscs and other invertebrates***

In gastropods, adverse effects resulting from *in vivo* exposure to TBT mainly concern reproductive impairment associated with alterations of the sexual organs in females, including imposex. Several studies indicate that the imposex threshold concentration starts at 1 ng TBT/L, with increasing sterilization as concentrations increase (*e.g*., Gibbs *et al.,* 1988). Gibbs (1996) found that juvenile female *Ocenebra erinacea* exposed to 7.3 ng TBT/L developed a longitudinal split of the oviduct wall. Adult *O. erinacea* collected from TBT contaminated sites with advanced imposex exhibited the same lesions and through laboratory spawning experiments were found to be sterile (no capsules produced). Significant reductions in gastropod reproduction were generally found at levels slightly higher than those that induced imposex, with effective concentrations ranging from 12 to 1000 ng TBT/L (Duft *et al.,* 2007; Leung *et al.,* 2007; Giusti *et al.,* 2013).

Other invertebrate species are known to exhibit reproductive effects across a wide aqueous concentration range of 10 to 2225 ng TBT/L (Oberdorster *et al.,* 1998; Huang *et al.,* 2010; Ohji *et al.,* 2003a,b).

**4.6. Level 5 – *In vivo* assays providing more comprehensive data on adverse effects on endocrine
relevant endpoints over more extensive parts of the life cycle of the organism**

***4.6.1. Mammals***

A two-generation reproductive toxicity study was conducted in rats using dietary exposure to TBT to evaluate its effect on sexual development and the reproductive system (Omura *et al.,* 2001; Ogata *et al.,* 2001). Omura *et al.* (2001) reported significant effects on monitored endpoints (reduced body weight, delayed eye opening, reduced testis, epididymis and ventral prostate weights, and decreased spermatid count) for males only and primarily at the high concentration tested. As is commonly seen in high dose exposures, the data suggest that these results were primarily related to direct toxic effects of TBT rather than functioning through specific endocrine pathways. A similar conclusion was drawn from the results of the companion study on the female offspring of these rats (Ogata *et al.,* 2001).

Chammorro-Garcia *et al.* (2013) conducted a follow-up study to the one by Kirchner *et al.* (2010), in which they exposed female mice prior to conception and during pregnancy to TBT in drinking water to determine whether prenatal exposure would affect subsequent generations (F1, exposed *in utero* ; F2, exposed as germ cells; F3, no exposure). Prenatal TBT exposure elicited striking transgenerational effects in males including increased white adipose tissue depot weights, adipocyte size, and adipocyte number at most doses in the three generations; more modest changes were observed in females. All three generations exhibited hepatic lipid accumulation and up-regulation of hepatic genes involved in lipid storage/transport, lipogenesis, and lipolysis. These results show that early-life exposure to TBT can have transgenerational effects on adipogenesis at least through the F3 generation.

***4.6.2. Fish***

Mochida *et al.* (2010) exposed mummichog *Fundulus heteroclitus* in a fish full-life-cycle assay from the embryo stage until the hatch of the F1 generation to TBT. In the F0 generation, TBT exposure resulted in a male-biased sex ratio, an increase in the frequency of the appearance of apoptotic cells in the testis in maturing stages, and a decrease in fecundity. In the F1 generation, time to hatch and hatchability were all markedly affected. Exposure did not affect the proliferation of the germ cells in the testis; however, a signiﬁcant increase in the number of apoptotic cells in the testis was induced.

***4.6.3. Molluscs and other invertebrates***

For gastropods exposed to TBT, level 5 mainly represents long-term laboratory exposure (> 1 year) or field monitoring. Long-term effects of TBT have been described for populations of stenoglossan snails (Bailey *et al.,* 1995; Harding *et al.,* 1997; Spence *et al.,* 1990). These population-level effects were associated with TBT water concentrations in the 1 to 10 ng TBT/L range. Oyster populations have also been impacted by TBT. For example, a correlation was found between rock oyster (*Saccostrea glomerata*) population density and the discontinued use of TBT in estuaries with high densities of boat moorage (Birch *et al.,* 2014).

Limited level-5 full life-cycle or multigenerational laboratory tests were conducted with other invertebrate species using TBT. The calanoid copepods *Pseudodiaptomus marinus* and *Schmackeria poplesia* exhibited similar sensitivity, with LOECs of 6 and 20 ng TBT/L, respectively (Huang *et al.,* 2006, 2010). The amphipod *Caprella danilevskii* exhibited similar sensitivity to the copepods with a LOEC of 10 ng TBT/L (Ohji *et al.,* 2003a,b). *Daphnia magna* was thought to be much less sensitive to TBT, with a LOEC of 2225 ng TBT/L in a two generation study (Oberdorster *et al.,* 1998). However, a recent study reported reduced clutch size, total offspring, neonate volume, and neonate length at 88 ng TBT/L, many of these effects being observed in the F1 and F2 generations (Jordão *et al.,* 2015).

**5. Weight-of-Evidence for RXR and PPAR_ϒ_ pathways**

**5.1. Mammals**

Kanayama *et al.* (2005) presented some of the first evidence implicating the RXRα and PPAR_ϒ_ pathways in TBT-mediated endocrine effects in mammals but also proposed that this pathway is the likely route for the low-dose imposex response in gastropods. Additional studies (Nakanishi *et al.,* 2005; Grun *et al.,* 2006; Le Maire *et al.,* 2009) provided further evidence that TBT exerts its biological effects *via* transcriptional regulation of gene expression through activation of these receptors, implicating this pathway in the adipogenetic effects of TBT. Mammalian aromatase gene expression is also regulated through the RXRα/PPAR_ϒ_ pathway by various ligands, including TBT. However, the direction of the regulation in response to TBT appears to be dose and tissue specific, potentially because aromatase is regulated through tissue-specific promoters (Simpson *et al.,* 1993).

**5.2. Fish**

The initiating event for reproductive and metabolic impairment in fish is also expected to occur *via* the RXR and/or PPAR receptors; several studies reported TBT-induced effects on adipogenesis *via* RXR/PPAR activation (Tingaud-Sequeira *et al.,* 2011; Meador *et al.,* 2011; Ouadah-Boussouf and Babin, 2016). In addition to the well-characterized role of PPARs in adipogenesis, diverse indications point to the existence of PPAR and ER crosstalk, although the exact mechanisms are not yet fully understood. It has been shown however that PPAR and RXR, or their heterodimer, can bind directly to estrogen responsive elements (EREs) in the gene promoters (Nunez *et al*., 1997). Several recent studies may be critical in providing the link between the well-documented effects on TBT on CYP expression (including aromatase) *via* RXR/PPAR activation (Chechenko *et al*., 2008; Kazeto *et al.,* 2001; Chang *et al.,* 2005; Nakanishi *et al.,* 2005, 2006; Mu *et al*., 2001). Retinoic acid (RA) has long been recognised as a morphogen, important for axial patterning and organ formation in developing vertebrates and comparative analysis of data from rat whole embryo culture, embryonic stem cells and the zebrafish embryotoxicity test identified certain conserved pathways on RA signaling between mammals and fish (Tonk *et al*., 2015). Analysis of genomic data revealed that the important morphogenic role of RA extends not only to invertebrate chordates (tunicates and cephalochordates), but other invertebrate groups, such as hemichordates and sea urchins (Campo-Paysaa *et al*., 2008).

Altogether, evidence suggests that most of the endocrine effects of TBT have their origin in RXR and RXR/PPAR activation. An important determinant of the severity and magnitude of responses in different species is the structure of the ligand binding domain, as studies have demonstrated the evolutionary plasticity of this domain, whilst the function of RAR, RXR and PPAR appears to be largely conserved.

**5.3. Molluscs**

From both *in vivo* (injection experiments) and *in vitro* (transactivation assays) experiments, there is strong evidence that TBT interacts with RXR in gastropods, both marine (*e.g.*, Castro *et al.,* 2007) and freshwater (Boulahtouf *et al.,* 2015) species. Such effects were observed at TBT concentrations of 1 nM TBT (equiv. 290 ng TBT/L) in transactivation assays and 1000 ng TBT/g wet body weight through injection. Interaction of TBT with RXR seems to be the main initiating event of changes in the development of sexual organs in female snails, ultimately resulting in imposex. More recently, the use of an open transcriptomic approach (Pascoal *et al.,* 2013) also suggested the involvement of PPAR pathways in *N. lapillus* chronically exposed to TBT. Nevertheless, although it is certain that activation of RXR and/or RXR/PPAR are the molecular initiating events, the mechanistic links between these different events and subsequent pathways remain largely unexplained.

Other primary molecular mechanisms have been also suggested, such as the activation of gonadotropin releasing hormone receptor (GnRHR) along with the gonadotropin releasing hormone (GnRH) (Castro *et al.,* 2007) or the aberrant secretion of neurohormones, primarily the neuropeptide APGWamide, which regulates male sexual differentiation in these species (Oberdorster and McClellan-Green, 2002). The body of literature is not large enough to allow full evaluation of these alternative pathways.

For many years, the mechanism of TBT-induced imposex was dominated by the steroid hypothesis. This was primarily due to researchers measuring high levels of free testosterone in tissues of impacted molluscs. The link between free testosterone and penis formation was instantly made and supported by numerous publications. However, besides extensive experimental efforts, treatment with either testosterone or fadrozole (a potent aromatase inhibitor) did not replicate this condition (*e.g*. Iguchi and Katzu*,* 2008). Although it is well established that TBT does affect many CYPs (including those involved in vertebrate steroidogenesis) and other metabolizing pathways (*e.g*. esterification), these effects alone do not constitute evidence of a physiological role for these steroids in molluscs. In fact, both the origin and physiological role of sex steroids in molluscs are still controversial (for review see Scott, 2012, 2013). However, the lack of a nuclear AR or AR-like homologues in the genomes of thus far studied molluscan species supports the lack of a physiological role for androgens at least in these species (Vogeler *et al.,* 2015; Kaur *et al.,* 2015).

A wide variety of organ responses are known in molluscs exposed to low concentrations of TBT. These include penis development in female snails, abnormal testis histopathology, and sperm alteration (count, motility, abnormality). These responses occur in the 1 – 10 ng TBT/L range for aqueous concentrations (*e.g*., Leung *et al.,* 2007, Horiguchi *et al.,* 1994) and 10 to 100 ng TBT/g wet wt. whole-body tissue (Meador, 2011) in affected gastropods. A large number of studies reported decline to extinction for populations with increasing proportions of imposexed females (Gibbs, 1996, 2009). Several studies indicate that TBT in the marine environment can impact populations of stenoglossan snails through female sterilization associated with imposex (Bailey *et al.,* 1995; Harding *et al.,* 1997; Spence *et al.,* 1990). These population-level responses were associated with water concentrations in the range 1 to 10 ng TBT/L, which is consistent with molecular studies characterizing the affinity of TBT for the RXR-PPAR receptor, in addition to other known ligands. Some studies have shown that imposex is irreversible for individuals in some species (Bryan *et al.,* 1987); however, populations of snails have recovered significantly after the reduction in use for this compound as an antifoulant (Bray *et al.,* 2012; Birchenough *et al.,* 2002; Nicolaus and Barry, 2015).

**6. Derivation of no-effect levels per taxa being assessed**

**6.1. Species Sensitivity Distribution (SSD)**

During the TBT case-study analysis, NOEC- and LOEC-based Species Sensitivity Distributions (SSDs) were constructed using apical, population-relevant data (reproductive outcomes, including *e.g.*, fertilization, embryonic development, hatching, larval and juvenile growth, as well as sex ratio) mainly from long-term aqueous exposure studies matching levels 4 and 5 in the OECD Conceptual Framework

The HC5 based on NOECs is 0.392 ng/L, whereas that based on LOECs is 1.03 ng/L. The NOEC-based HC5 value is close to current EU regulatory thresholds (*e.g.* 0.2 ng/L; EU, 2005) that incorporate an assessment factor, so should be treated with caution. For the US, the ambient water quality criteria (seawater chronic) has been set to 7 ng/L (US EPA, 2003).

Some fish and invertebrate species are as sensitive to TBT as molluscs, in particular some copepods and the zebrafish. The lowest endocrine-sensitive LOEC identified in the TBT Case Study literature research exercise was 0.1 ng TBT/L for a male-biased sex ratio change and abnormal sperm in zebrafish (McAllister and Kime, 2003). This value then drives the TBT risk assessment for fish and interestingly also for molluscs and other species. However, the effect observed in zebrafish may be restricted to fish species that share the same strategy in sexual differentiation rather than all fish.

Even though a given ambient toxicity metric (*e.g*., LC_50_) that is based on aqueous or sediment concentrations can result in a range spanning orders of magnitude for different species, the equivalent tissue residue toxicity metric (*e.g*., LR_50_) often exhibits low variability (Meador, 1997). TBT provided one example of the utility for this approach based on tissue residue toxicity data for mortality and reduced growth. While some of the datasets are limited, they do indicate a relatively consistent response among species for a given endpoint and whole-body tissue concentration (Meador, 2011).

An analysis of the imposex endpoint as a function of whole-body tissue concentrations has been done for different species of marine gastropods using the SSD approach. This SSD showed that the sensitivity of one given species may vary according to the imposex stage and TBT tissue burden, and there is a relatively narrow range of concentrations for the imposex response spanning from threshold to 100% induced. Based on these data, the HC5 was determined to be 11.4 ng TBT/g wet weight (95% CI = 9.1 – 14.1).

**6.2. Rationale for endpoints to be used quantitatively in the risk assessment**

Based on the sublethal population-relevant data reviewed, risks would be expected to occur at TBT water concentrations equal to or exceeding 1 ng TBT/L (lowest LOEC values for both fish and molluscs). This is broadly consistent with field observations that suggest that imposex in some prosobranch molluscs begins to be observed at around the same concentration (*e.g.* Bryan *et al.,* 1987, 1993). If an assessment factor were also applied, the critical concentration would be lower.

**7. Risk Assessment: Comparison of PEC to various No Effect Levels**

**following established risk assessment practices**

The estimated 90^th^ percentile of TBT concentrations in English estuarine waters exceeds the estimated NOEC-LOEC ‘band’ of 0.23-0.44 ng/L, and the arithmetic mean concentration also falls within this range (the median is just below). The level of risk would be greater if an assessment factor were applied. Risks to the marine environment from TBT may therefore still exist seven years since the use of TBT as an antifoulant was entirely restricted at a global level (and almost thirty years since the original ban in the UK). This reflects both the high toxicity of the substance and its persistence in the environment.

**8. Sources of Uncertainty/Data gaps**

**8.1. Transgenerational effects**

TBT is bioaccumulative, and maternal transfer to eggs does occur (*e.g*. Inoue *et al.,* 2006; Ohji *et al.,* 2006). Effects can occur over multiple generations in some invertebrate species (*e.g*. chironomids (Lilley *et al*., 2012) and copepods (Huang *et al.,* 2006)), as well as in fish, birds, and mammals. However, it is not clear whether or not this is exclusively linked to endocrine-mediated mechanisms.

**8.2. Sensitive species**

The general academic perception is that prosobranch molluscs are the most sensitive taxonomic group, due to the observation of imposex in wild species at low environmental concentrations. However, this analysis has highlighted that a number of fish species and some invertebrate groups (*e.g*. copepods) have a similar or greater level of sensitivity in terms of population-relevant endpoints.

**8.3. Potency**

TBT is a highly toxic substance, causing a variety of significant sublethal effects in aquatic organisms at levels of parts per trillion (ng/L) and lower, and low ng/g tissue concentrations.

**8.4. Non-monotonic dose response or lack of a threshold dose**

Inverted U-shape responses have been observed for some endpoints, depending on the concentration, mainly involving gene expression studies (*e.g.* Mortensen and Arukwe, 2007; Kortner *et al.,* 2010; Morales *et al.,* 2013; Pascoal *et al.,* 2013).

From *in vivo* studies, there is also some evidence that TBT has been shown to display an inverted U-shape response for several endpoints. A good example is the impact of TBT on body weight in fish and mammals (Cooke *et al.,* 2008; Meador *et al.,* 2011; Si *et al.,* 2011). Meador *et al.* (2011) for example found that TBT exposure in fish enhanced growth and lipogenesis at low doses and inhibited growth and reduced lipid content at high doses; this was attributed to two modes of action operating at different doses. Most likely, the cause of a non-linear response for TBT is different dose-dependent mechanisms of action. Such non-linear concentration-response should be considered with caution regarding the magnitude and consistency of the changes (are they significant/important? do they consistently occur in different species?).

One should also consider whether U-shaped dose-responses are a result of endocrine-mediated perturbations, multiple mechanisms of action or adaptive process. They also may relate to exposure artefacts (*e.g.* precipitation due to lack of solubility or pump failures).

**8.5. Data gaps with dose setting**

Many studies performed in academic laboratories used relatively few treatment groups (perhaps due to space and resource constraints), and seem not to be based on range-finding studies. A number therefore report unbounded LOECs, or have a large spacing between the LOEC and NOEC. Other problems include study designs with only one or two doses and the lack of independent replicates (pseudoreplication).

**8.6. Reversibility of the effect**

The degree of imposex reversibility in molluscs depends on the species; once TBT exposure stops, female penis length slowly declines in *Nassarius reticulatus.* The process seems to be faster in female *Ilyanassa obsoleta*, whereas in *N. lapillus*, imposex appeared to be largely irreversible (Bryan *et al.,* 1993). Population recovery can therefore be slow, especially for species that are long-lived and/or for which recruitment is limited, such as *N. lapillus* (Matthiessen and Gibbs, 1998; Oehlmann *et al.,* 2007; OECD 2010) and *N. reticulatus* (Couceiro *et al.,* 2009). Nevertheless, there appears to be a widespread amelioration worldwide (*e.g*., Canada, US EPA, 2003; Hong-Kong, Leung *et al.,* 2006; Spain, Couceiro *et al.,* 2009; England and Wales, Nicolaus and Barry, 2015).

**9. Conclusions for the TBT Case Study – Hazard *vs.* Risk assessment**

TBT was introduced on the market in the early 1960s, at a time when regulatory assessment of chemicals was at an embryonic stage. Using deliberate “retrospective thinking”, and considering the information gathered in this case study, the question should be: would TBT be identified as an ED using current screening and testing methods?

ToxCast^TM^ uses high-throughput screening methods and computational toxicology approaches to rank and prioritize chemicals. Refinements to ToxCast^TM^ now allow the identification of endocrine molecular initiating events through RXR and/or PPAR pathways. TBT activity in *in vitro* RXR and PPAR assays typically occurs at levels less than the cytotoxicity limit. Within the OECD EDTA framework, TBT is shown to alter the sex-ratio and to induce sperm abnormality in a Fish Sexual Development Test (FSDT) with zebrafish (McAllister and Kime, 2003) and to delay frog development in the AMA (Shi *et al*., 2014), at low concentrations (0.1 ng/L and 10 ng/L, respectively). It also appears positive in a FETAX-like assay (Guo *et al*., 2010). Based upon these findings, TBT can be identified as acting on endocrine pathways mediated by RXR and/or PPAR, even though the exact sequence of events is unknown.

Further testing first considers tier-1 data on additional standard species. Chronic tests on *D. magna*, *Crassostrea gigas*, *Oncorhynchus mykiss* and *Pimephales promelas* provide the endpoints values of 88, 5, 60 and 150 ng TBT/L, respectively (N.B.: these data are a mixture of NOECs and LOECs). However, zebrafish *D. rerio* remains the most sensitive species, with a NOEC value of 0.01 ng/L for highly population-relevant endpoints (sex-ratio), including delayed effects (sperm abnormality 3 to 5 months post-exposure). By applying an assessment factor (AF) of 10 on the lowest NOEC value, the **Regulatory Acceptable Concentration (RAC) is 0.001 ng/L**.

In a refined tier-2 assessment option, the HC5 from a population-relevant NOEC-based SSD is 0.392 ng/L. Depending on regulatory guidance and jurisdictions, an AF of 1-5 would normally be applied on the HC5, resulting in a higher RAC than that derived from tier-1 assessment. Thus, the tier-2 RAC might not be fully protective for aquatic fauna since it exceeds the NOEC from the most sensitive fish species. The environmental risk assessment of TBT should therefore be based on the tier-1 RAC derived from a chronic, endocrine-relevant test on the most sensitive species (*i.e*., FSDT on zebrafish).

Interestingly, tier-2 testing clearly shows that TBT is highly toxic to many aquatic species, the most sensitive clearly being fish. This is an important secondary conclusion since it appears that fish lead the risk assessment for TBT rather than molluscs, for an environmental contaminant long recognized for its uniquely sensitive impacts on molluscs.

For the exposure assessment, measured environmental concentrations post-2008 show that **exposure may still occur in the range 0.1 – 8 ng/L** (arithmetic mean = 0.3 ng/L) in representative European surface waters. This exceeds the preferred RAC by at least two orders of magnitude, suggesting that there is still an environmental risk from legacy contamination. Risks may be higher in regions of the world with less effective controls.

In conclusion, the combination of high persistence, high bioaccumulation, and multiple endocrine-mediated effects on many species at low concentrations mean that the risk assessment approach for TBT would be suitably precautionary only if the most sensitive species is tested using an endocrine-relevant assay at tier 1. Otherwise, assessment factors higher than those currently used (*e.g*., 100 for chronic tier-1 RAC and 10 for chronic tier-2 RAC) should be applied. Alternately, TBT could be evaluated using a hazard assessment approach, which in fact has been the default regulatory tactic to date.

**References**

Abidli S, Santos MM, Lahbib Y, Castro LFC, Reis-Henriques MA, El Menif NT. 2012. Tributyltin (TBT) effects on *Hexaplex trunculus* and *Bolinus brandaris* (Gastropoda: Muricidae): Imposex induction and sex hormone levels insights. *Ecological Indicators* 13, 13-21.

[Abidli S](http://apps.webofknowledge.com/OneClickSearch.do?product=UA&search_mode=OneClickSearch&SID=V1pEzxlvVM4tcM2KeTG&field=AU&value=Abidli,%20S&ut=47456&pos=%7b2%7d&excludeEventConfig=ExcludeIfFromFullRecPage), [Castro LFC](http://apps.webofknowledge.com/OneClickSearch.do?product=UA&search_mode=OneClickSearch&SID=V1pEzxlvVM4tcM2KeTG&field=AU&value=Castro,%20LFC&ut=3828853&pos=%7b2%7d&excludeEventConfig=ExcludeIfFromFullRecPage), [Lahbib Y](http://apps.webofknowledge.com/OneClickSearch.do?product=UA&search_mode=OneClickSearch&SID=V1pEzxlvVM4tcM2KeTG&field=AU&value=Lahbib,%20Y&ut=10491383&pos=%7b2%7d&excludeEventConfig=ExcludeIfFromFullRecPage), [Reis-Henriques MA](http://apps.webofknowledge.com/OneClickSearch.do?product=UA&search_mode=OneClickSearch&SID=V1pEzxlvVM4tcM2KeTG&field=AU&value=Reis-Henriques,%20MA&ut=16889609&pos=%7b2%7d&excludeEventConfig=ExcludeIfFromFullRecPage), [El Meni NT](http://apps.webofknowledge.com/OneClickSearch.do?product=UA&search_mode=OneClickSearch&SID=V1pEzxlvVM4tcM2KeTG&field=AU&value=El%20Menif,%20NT&ut=5081582&pos=%7b2%7d&excludeEventConfig=ExcludeIfFromFullRecPage), [Santos MM](http://apps.webofknowledge.com/OneClickSearch.do?product=UA&search_mode=OneClickSearch&SID=V1pEzxlvVM4tcM2KeTG&field=AU&value=Santos,%20MM&ut=17652299&pos=%7b2%7d&excludeEventConfig=ExcludeIfFromFullRecPage). 2013. Imposex development in *Hexaplex trunculus* (Gastropoda: Caenogastropoda) involves changes in the transcription levels of the retinoid X receptor (RXR). *Chemosphere* 93, 1161-1167.

Antizar[-Ladislao B](http://apps.webofknowledge.com/OneClickSearch.do?product=UA&search_mode=OneClickSearch&SID=V1pEzxlvVM4tcM2KeTG&field=AU&value=Antizar-Ladislao,%20B&ut=566089&pos=%7b2%7d&excludeEventConfig=ExcludeIfFromFullRecPage). 2008. Environmental levels, toxicity and human exposure to tributyltin (TBT)-contaminated marine environment. *Environment International* 34, 292-308.

Bailey SK, Davies IM, Harding MJC. 1995. Tributyltin contamination and its impact on *Nucella lapillus* populations. *Proceedings of the Royal Society of Edinburgh. Section B. Biological Sciences* 103, 113-126.

Bartlett AJ, Borgmann U, Dixon DG, Batchelor SP, Maguire RJ. 2007.Comparison of toxicity and bioaccumulation of tributyltin in Hyalella azteca and five other freshwater invertebrates. *Water Quality Research Journal of Canada* 42, 1-10.

Belcher SM, Cookman CJ, Patisaul HB, Stapleton HM. 2014. *In vitro* assessment of human nuclear hormone receptor activity and cytotoxicity of the flame retardant mixture FM 550 and its triarylphosphate and brominated components. *Toxicology Letters* 228, 93-102.

Bentivegna CS, Piatkowski, T. 1998. Effects of tributyltin on medaka (*Oryzias latipes*) embryos at different stages of development. *Aquatic Toxicology* 44, 117-128.

Birch GF, Scammell MS, [Besley CH](http://www.ncbi.nlm.nih.gov/pubmed/?term=Besley%20CH%5BAuthor%5D&cauthor=true&cauthor_uid=24062067) (2014). The recovery of oyster (*Saccostrea glomerata*) populations in Sydney estuary (Australia). *Environmental science and pollution research international* 21, 766-773.

Birchenough AC, Barnes N, Evans SM, Hinz H, Kronke I, Moss C. 2002. A review and assessment of tributyltin contamination in the North Sea, based on surveys of butyltin tissue burdens and imposex/intersex in four species of neogastropods. *Marine Pollution Bulletin* 44, 534-543.

Boulahtouf A, Grimaldi M, Coutellec M-A, Besnard A-L, Echasseriau Y, Bourguet W, Balaguer P, Lagadic L. 2015. Ligand affinity of the *Lymnaea stagnalis* estrogen and retinoid-X receptors (LsER and LsRXR): implications for detecting endocrine disruptors. *25th SETAC Europe Annual Meeting, Barcelona, Extended Abstract*.

Bray S, McVean EM, Nelson A, Herbert RJH, and Hawkins SJ, and Hudson, MD. 2012. The regional recovery of *Nucella lapillus* populations from marine pollution, facilitated by man-made structures. Jour Mar Biol Assoc U.K. 92, 1585-1594.

Bryan GW, Gibbs PE, Burt GR, Hummerstone LG. 1987. The effects of tributyltin (TBT) accumulation on adult dogwhelks, *Nucella lapillus*: long term field and laboratory experiments. *Journal of the Marine Biological Association of the UK* 67, 525-544.

Bryan GW, Burt GR, Gibbs PE, Pascoe PL. 1993. *Nassarius reticulatus* (Nassariidae: Gastropoda) as an indicator of tributyltin pollution before and after TBT restrictions. *Journal of the Marine Biological Association of the UK* 73, 913–929.

Campo-Paysaa F., Marlétaz F, Laudet V, Schubert M. 2008. Retinoic acid signaling in development: tissue-specific functions and evolutionary origins. *Genesis*, 46, 640-656.

Castro LFC, Lima D, Machado A, Melo C, Hiromori Y, Nishikawa J, Nakanishi T, Reis-Henriques MA, Santos MM. 2007. Imposex induction is mediated through the Retinoid X Receptor signaling pathway in the neogastropod *Nucella lapillus*. *Aquatic Toxicology* 85, 57–66.

Coenen TMM, Brouwer A, Enninga IC, Koeman JH. 1992. Subchronic toxicity and reproduction effects of tri-n-butyltin oxide in Japanese quail. *Archives of Environmental Contamination and Toxicology* 23, 457-463.

Colliar L, Sturm, A, Leaver, MJ. 2011. Tributyltin is a potent inhibitor of piscine peroxisome proliferator-activated receptor alpha and beta. *Comparative Biochemistry and Physiology C* 153, 168-173.

Cooke, GM. 2002. Effect of organotins on human aromatase activity in vitro. *Toxicology Letters* 126, 121–130.

Cooke GM, Forsyth DS, Bondy GS, Tachon R, Tague B, Coady L, 2008. Organotin speciation and tissue distribution in rat dams, fetuses, and neonates following oral administration of tributyltin chloride. *Journal of Toxicology and Environmental Health A* 71, 384-395.

Couceiro L, Diaz J, Albaina N, Barreiro R, Irabien JA, Ruiz JM. 2009. Imposex and gender-independent butyltin accumulation in the gastropod *Nassarius reticulatus* from the Cantabrian coast (N Atlantic Spain). *Chemosphere* 76, 424-427.

Decherf S, Seugnet I, Fini J-B, Clerget-Froidevaux M-S, Demeneix BA. 2010. Disruption of thyroid hormone-dependent hypothalamic set-points by environmental contaminants. *Molecular and Cellular Endocrinology* 323, 172-182.

Duft M, Schmitt C, Bachmann J, Brandelik C, Schulte-Oehlmann U, Oehlmann J. 2007. Prosobranch snails as test organisms for the assessment of endocrine active chemicals - an overview and a guideline proposal for a reproduction test with the freshwater mudsnail *Potamopyrgus antipodarum*. *Ecotoxicology* 16, 169-182.

EU (2005). Common Implementation Strategy for the Water Framework Directive: Environmental Quality Standards (EQS), Substance Data Sheet. Priority Substance No. 30: Tributyltin compounds (TBT-ion). Final version, Brussels, January 2005.

ECHA (2008). Member State Committee Support Document for Identification of Bis(tributyltin) Oxide as a Substance of Very High Concern. European Chemicals Agency, October 2008.

Fent K, Hunn J. 1996. Cytotoxicity of organic environmental chemicals to fish liver cells (PLHC-1). *Marine Environmental Research* 42, 377–382.

Gibbs PE, Pascoe PL, Burt GR. 1988. Sex change in the female dog-whelk, *Nucella lapillus*, induced by tributyltin from antifouling paints. *Journal of the Marine Biological Association of the UK* 68, 715-731.

Gibbs PE. 1996. Oviduct as a sterilising effect of tributyltin (TBT)-induced imposex in *Ocenebra erinacea* (Gastropoda: Muricidae). *Journal of Molluscan Studies* 62, 403-413.

Gibbs PE. 2009. Long-term tributyltin (TBT)-induced sterilization of neogastropods: persistence of effects in Ocenebra erinacea over 20 years in the vicinity of Falmouth (Cornwall, UK). *Journal of the Marine Biological Association of the UK* 89, 135–138.

Giusti A, Barsi A, Dugue M, Collinet M, Thome JP, Joaquim-Justo C, Roig B, Lagadic L, Ducrot V. 2013. Reproductive impacts of tributyltin (TBT) and triphenyltin (TPT) in the hermaphroditic freshwater gastropod *Lymnaea stagnalis*. *Environmental Toxicology and Chemistry* 32, 1552-1560.

Gooding MP, Wilson VS, Folmar LC, Marcovich DT, LeBlanc GA. 2003. The Biocide Tributyltin Reduces the Accumulation of Testosterone as Fatty Acid Esters in the Mud Snail (*Ilyanassa obsoleta*). *Environmental Health Perspectives* 111, 426-430.

Grun F, Watanabe H, Zamanian Z, Maeda L, Arima K, Cubacha R, Gardiner DM, Kanno J, Iguchi T, Blumberg B. 2006. Endocrine-disrupting organotin compounds are potent inducers of adipogenesis in vertebrates. *Molecular Endocrinology* 20, 2141-2155.

Guo S, Quan L, Shi H, Barry T, Cao Q, Liu J. 2010. Effects of tributyltin (TBT) on *Xenopus tropicalis* embryos at environmentally relevant concentrations. *Chemosphere* 79, 529-533.

Hano T, Oshima Y, Kim SG, Satone H, Oba Y, Kitano T, Inoue S, Shimasaki Y, Honjo T. 2007. Tributyltin causes abnormal development in embryos of medaka, *Oryzias latipes*. *Chemosphere* 69, 927-933.

Harazono A, Ema M, Ogawa Y. 1996. Pre-implantation embryonic loss induced by tributyltin chloride in rats. *Toxicology Letters* 89, 185-190.

Harazono A, Ema M, Ogawa Y. 1998. Evaluation of Early Embryonic Loss Induced by Tributyltin Chloride in Rats: Phase- and Dose-Dependent Antifertility Effects. *Archives of Environmental Contamination and Toxicology* 34, 94-99.

Harding MJC, Rodger GK, Davies IM, Moore JJ. 1997. Partial recovery of the dogwhelk (*Nucella lapillus*) in Sullom Voe, Shetland from tributyltin contaminations. *Marine Environmental Research* 44, 285-304.

Haubruge E, Petit F, Gage MJ. 2000. Reduced sperm counts in guppies (*Poecilia reticulata*) following exposure to low levels of tributyltin and bisphenol A. *Proceedings of the Royal Society London B* 67, 2333-2337.

Heidrich DD, Steckelbroeck S, Klingmuller D. 2001. Inhibition of human cytochrome P450 aromatase activity by butyltins. *Steroids* 66, 763-769.

Horiguchi T, Shiraishi H, Shimizu M, Morita M. 1994. Imposex and organotin compounds in *Thais clavigera* and *T. bronni* in Japan. *Journal of the Marine Biological Association of the UK* 74, 651-669.

Huang Y, Zhu L, Liu G. 2006. The effects of bis(tributyltin) oxide on the development, reproduction and sex ratio of calanoid copepod *Pseudodiaptomus marinus*. *Estuarine, Coastal and Shelf Science* 69, 147-152.

Huang Y, Zhu LY, Qiu XC and Zhang TW. 2010. Effect of bis(tributyltin) oxide on reproduction and population growth rate of calanoid copepod *Schmackeria poplesia*. *Chinese Journal of Oceanology and Limnology* 28, 280-287.

Iguchi T, Katsu Y. 2008. Commonality in signaling of endocrine disruption from snail to human. *Bioscience* 58, 1061-1067.

Inoue S, Oshima Y, Usuki H, Hamaguchi M, Hanamura Y, Kai N, Shimasaki Y & Honjo T. 2006. Effects of tributyltin maternal and/or waterborne exposure on the embryonic development of the Manila clam, *Ruditapes philippinarum. Chemosphere* 63, 881–888.

Jordão R, Casas J, Fabrias G, Campos B, Piña B, Lemos MF, Soares AM, Tauler R, Barata C. 2015. Obesogens beyond vertebrates: lipid perturbation by tributyltin in the crustacean *Daphnia magna*. *Environmental Health Perspectives* 123, 813–819.

Kanayama T, Kobayashi N, Mamiya S, Nakanishi T, Nishikawa J. 2005. Organotin Compounds Promote Adipocyte Differentiation as Agonists of the Peroxisome Proliferator-Activated Receptor γ/Retinoid X Receptor Pathway. *Molecular Pharmacology* 67, 766-774.

Kaur S, Jobling S, Jones CS, Noble LR, Routledge EJ, Lockyer AE. 2015. The nuclear receptors of Biomphalaria glabrata and Lottia gigantea: implications for developing new model organisms. *PLOS One* 10 (4).

Kirchner S, Kieu T, Chow C, Casey S, Blumberg B. 2010. Prenatal exposure to the environmental obesogen tributyltin predisposes multipotent stem cells to become adipocytes. *Molecular Endocrinology* 24, 526-539.

Kishta O, Adeeko A, Li D, Luu T, Brawer JR, Morales C, Hermo L, Robaire B, Hales BF, Barthelemy J, Cyr DG, Trasler JM. 2007. In utero exposure to tributyltin chloride differentially alters male and female fetal gonad morphology and gene expression profiles in the Sprague–Dawley rat. *Reproductive Toxicology* 23, 1-11.

Kortner TM, Pavlikova N, Arukwe A. 2010. Effects of tributyltin on salmon interrenal CYP11 beta, steroidogenic factor-1 and glucocorticoid receptor transcripts in the presence and absence of second messenger activator, forskolin. *Marine Environmental Research* 69, S56-S58.

Kuhl AJ, Brouwer M. 2006. Antiestrogens inhibit xenoestrogen-induced brain aromatase activity but do not prevent xenoestrogen-induced feminization in Japanese medaka (*Oryzias latipes*). *Environmental Health Perspectives* 114, 500-506.

Laflamme L, Hamann G, Messier N, Maltais S, Langlois M-F. 2002. RXR acts as a coregulator in the regulation of genes of the hypothalamo-pituitary axis by thyroid hormone receptors. *Journal of Molecular Endocrinology* 29, 61-72.

le Maire A, Grimaldi M, Roecklin D, Dagnino S, Vivat‐Hannah V, Balaguer P, Bourguet W. 2009. Activation of RXR-PPAR heterodimers by organotin environmental endocrine disruptors. *EMBO reports* 10, 367–373.

Leung KMY, Kwong RPY, Ng WC, Horiguchi T, Qiu JW, Yang RQ, Song MY, Jiang GB, Zheng GJ, Lam PKS. 2006. Ecological risk assessments of endocrine disrupting organotin compounds using marine neogastropods in Hong Kong. *Chemosphere* 65, 922-938.

Leung KM, Grist EP, Morley NJ, Morritt D, Crane M. 2007. Chronic toxicity of tributyltin to development and reproduction of the European freshwater snail *Lymnaea stagnalis* (L.). *Chemosphere* 66, 1358-1366.

Lilley TM, Ruokolainen L, Pikkarainen A, Laine VN, Kilpimaa J, Rantala MJ, Nikinmaa M. 2012. Impact of tributyltin on immune response and life history traits of *Chironomus riparius*: single and multigeneration effects and recovery from pollution. *Environmental Science and Technology* 46, 7382-7389.

Lima D, Reis-Henriques MA, Silva R, Santos AI, Castro LFC, Santos MM. 2011. Tributyltin-induced imposex in marine gastropods involves tissue-specific modulation of the retinoid X receptor. *Aquatic Toxicology* 101, 221–227.

Lima D, Castro LFC, Coelho I, Lacerda R, Gesto M, Soares J, André A, Capela R, Torres T, Carvalho AP, Santos MM. 2015. Effects of tributyltin and other retinoid receptor agonists in Reproductive-related endpoints in the zebrafish (*Danio rerio*). *Journal of Toxicology and Environmental Health A* 78, 747-760.

Matthiessen P, Gibbs PE. 1998. Critical appraisal of the evidence for tributyltin-mediated endocrine disruption in molluscs. *Environmental Toxicology and Chemistry* 17, 37-43.

McAllister BG, Kime DE. 2003. Early life exposure to environmental levels of the aromatase inhibitor tributyltin causes masculinisation and irreversible sperm damage in zebrafish (*Danio rerio*). *Aquatic Toxicology* 65, 309-316.

McGinnis CL, Crivello JF. 2011. Elucidating the mechanism of action of tributyltin (TBT) in zebrafish. *Aquatic Toxicology* 103, 25-31.

McVey MJ, Cooke GM. 2003. Inhibition of rat testis microsomal 3β-hydroxysteroid dehydrogenase activity by tributyltin. *The Journal of Steroid Biochemistry and Molecular Biology* 86, 99-105

Meador JP. 1997. Comparative toxicokinetics of tributyltin in five marine species and its utility in predicting bioaccumulation and toxicity. *Aquatic Toxicology* 37, 307-326.

Meador JP. 2006. Rationale and procedures for using the tissue-residue approach for toxicity assessment and determination of tissue, water, and sediment quality guidelines for aquatic organisms. *Human and Ecological Risk Assessment* 12, 1018-1073.

Meador JP. 2011. Organotins in aquatic biota: Occurrence in tissue and toxicological significance. *In*: Beyer WN and Meador JP (eds), Environmental Contaminants in Biota: Interpreting Tissue Concentrations. Taylor and Francis, Boca Raton, FL. pp. 255-284.

Meador JP, Sommers FC, Cooper K and Yanagida G. 2011. Tributyltin and the obesogen metabolic syndrome in a salmonid. *Environmental Research* 111, 50-56.

Mochida K, Ito K, Kono K, Onduka T, Kakuno A, Fujii K. 2007. Molecular and histological evaluation of tributyltin toxicity on spermatogenesis in a marine fish, the mummichog (*Fundulus heteroclitus*). *Aquatic Toxicology* 83, 73-83.

Mochida K, Ito K, Kono K, Onduka T, Kakuno A, Fujii K. 2010. Effect of tributyltin oxide exposure on the F-0 and F-1 generations of a marine teleost, the mummichog *Fundulus heteroclitus*. *Fisheries Science* 76, 333-341.

Morales M, Martinez-Paz P, Ozaez I, Martinez-Guitarte JL, Morcillo G. 2013. DNA damage and transcriptional changes induced tributyltin (TBT) after short *in vivo* exposures of *Chironomus riparius* (Diptera) larvae. *Comparative Biochemistry and Physiology C* 158, 57-63.

Morcillo Y, Janer G, O’Hara SCM, Livingstone DR, Porte C. 2004. Interaction of tributyltin with hepatic cytochrome P450 and uridine diphosphate-glucoronosyl transferase systems of fish: In vitro studies. *Environmental Toxicology and Chemistry* 23, 990-996.

Mortensen AS, Arukwe A. 2007. Modulation of xenobiotic biotransformation system and hormonal responses in Atlantic salmon (*Salmo salar*) after exposure to tributyltin (TBT). *Comparative Biochemistry and Physiology C* 145, 431-441.

Mortensen AS, Arukwe A. 2009. Effects of tributyltin (TBT) on in vitro hormonal and biotransformation responses in Atlantic salmon (*Salmo salar*). *Journal of Toxicology and Environmental Health* A 72, 209-218.

Mu Y-M, Yanase T, Nishi Y, Takayanagi R, Goto K, Nawata H. 2001. Combined treatment with specific ligands for PPAR[gamma]: RXR nuclear receptor system markedly inhibits the expression of cytochrome P450arom in human granulosa cancer cells. *Molecular and Cellular Endocrinology* 181, 239–248.

Nakanishi T, Kohroki J, Suzuki S, Ishizaki J, Hiromori Y, Takasuga S, Itoh N, Watanabe Y, Utoguchi N, Tanaka K. 2002. Trialkyltin compounds enhance human CG secretion and aromatase activity in human placental choriocarcinoma cells. *The Journal of Clinical Endocrinology and Metabolism* 87, 2830-2837.

Nakayama K, Oshima Y, Nagafuchi K, Hano T, Shimasaki Y, Honjo T. 2005. Early-life stage toxicity in offspring from exposed parent Medaka, *Oryzias latipes*, to mixtures of tributyltin and polychlorinated biphenyls. *Environmental Toxicology Chemistry* 24, 591-596.

Nicolaus EEM, Barry J. 2015. Imposex in the dogwhelk (*Nucella lapillus*): 22-year monitoring around England and Wales. *Environmental Monitoring and Assessment* 187, 736.

Nishikawa J, Mamiya S, Kanayama T, Nishikawa T, Shiraishi F, Horiguchi T. 2004. Involvement of the retinoid X receptor in the development of imposex caused by organotins in gastropods. *Environmental Science and Technology* 38, 6271-6276.

Nunez SB, Medin JA, Braissant O, Kemp L, Wahli W, Ozato K, Segars JH. 1997. Retinoid X receptor and peroxisome proliferatoractivated receptor activate an estrogen responsive gene independent of the estrogen receptor. *Molecular and Cellular Endocrinology* 127, 27–40.

Oberdorster E, Rittschof D, LeBlanc GA. 1998. Alteration of [C-14]-testosterone metabolism after chronic exposure of *Daphnia magna* to tributyltin. *Archives of Environmental Contamination and Toxicology* 34, 21-25.

Oberdorster E, McClellan-Green P (2002) Mechanisms of imposex induction in the mud snail, *Ilyanassa obsoleta*: TBT as a neurotoxin and aromatase inhibitor. *Marine Environmental Research* 54, 715–718.

OECD (2007). SIDS Initial Assessment Profile for SIAM 24, 19-20 April, 2007. Tributyltin chloride, CAS No. 1461-22-9. <http://webnet.oecd.org/Hpv/ui/handler.axd?id=738dc513-4ec8-4d55-ac11-4e1ff79123b8>.

OECD (2010) Detailed review paper (DRP) on molluscs life-cycle toxicity testing. *OECD Environment, Health and Safety Publications, Series on Testing and Assessment*, No. 121, pp. 182.

OECD (2012). Detailed review paper on the state of the science on novel *in vitro* and *in vivo* screening and testing methods and endpoints for evaluating endocrine disruptors. *OECD Environment, Health and Safety Publications, Series on Testing and Assessment*, No. 178, pp. 213

Oehlmann J, Di Benedetto P, Tillmann M, Duft M, Oetken M, Schulte-Oehlmann U. 2007. Endocrine disruption in prosobranch molluscs: evidence and ecological relevance. *Ecotoxicology* 16, 29-43.

Ogata R, Omura M, Shimasaki Y, Kubo K, Oshima Y, Aou S, Inoue N. 2001. Two-generation reproductive toxicity study of tributyltin chloride in female rats. *Journal of Toxicology and Environmental Health* 63, 127-144.

Ohhira S, Watanabe M, Matsui H. 2003. Metabolism of tributyltin and triphenyltin by rat, hamster and human hepatic microsomes. *Archives of Toxicology* 77, 138-144.

Ohhira S, Enomoto M, Matsui H. 2006a. In vitro metabolism of tributyltin and triphenyltin by human cytochrome P-450 isoforms. *Toxicology* 228, 171-177.

Ohhira S, Enomoto M, Matsui H. 2006b. Sex difference in the principal cytochrome P-450 for tributyltin metabolism in rats. *Toxicology and Applied Pharmacology* 210, 32-38.

Ohji M, Arai T, Miyazaki N. 2003a. Biological effects of tributyltin exposure on the caprellid amphipod, *Caprella danilevskii*. *Journal of the Marine Biological Association of the UK* 83, 111-117.

Ohji M, Arai T, Miyazaki N. 2003b. Chronic effects of tributyltin on the caprellid amphipod *Caprella danilevskii*. *Marine Pollution Bulletin* 46, 1263-1272.

Ohji M, Arai T, Miyazaki N. 2006. Transfer of tributyltin from parental female to offspring in the viviparous surfperch *Ditrema temmincki*. *Marine Ecology Progress Series* 307, 307-310.

Omura M, Ogata R, Kubo K, Shimasaki Y, Aou S, Oshima Y, Tanaka A, Hirata M, Makita Y, Inoue N. 2001. Two-generation reproductive toxicity study of tributyltin chloride in male rats. *Toxicological Sciences* 64, 224-232.

Ouadah-Boussouf N, Babin PJ. 2016. Pharmacological evaluation of the mechanisms involved in increased adiposity in zebrafish triggered by the environmental contaminant tributyltin. *Toxicology and Applied Pharmacology* 294, 32–42.

Pascoal S, Carvalho G, Vasieva O, Hughes R, Cossins A, Fang Y-X, Ashelford K, Olohan L, Barroso C, Mendo S, Creer S. 2013. Transcriptomics and *in vivo* tests reveal novel mechanisms underlying endocrine disruption in an ecological sentinel, *Nucella lapillus*. *Molecular Ecology* 22, 1589–1608.

Pavlikova N, Kortner TM, Arukwe A. 2010. Peroxisome proliferator-activated receptors, estrogenic responses and biotransformation system in the liver of salmon exposed to tributyltin and second messenger activator. *Aquatic Toxicology* 99, 176-185.

Pavlikova N, Arukwe A. 2011. Immune-regulatory transcriptional responses in multiple organs of Atlantic salmon after tributyltin exposure, alone or in combination with forskolin. *Journal of Toxicology and Environmental Health A* 74, 478-493.

Pereira ML, Eppler E, Thorpe KL, Wheeler JR, Burkhardt-Holm P. 2011a. Molecular and cellular effects of chemicals disrupting steroidogenesis during early ovarian development of brown trout (*Salmo trutta fario*). *Environmental Toxicology* 29, 199-206.

Pereira ML, Wheeler JR, Thorpe KL, Burkhardt-Holm P. 2011b. Development of an *ex vivo* brown trout (*Salmo trutta fario*) gonad culture for assessing chemical effects on steroidogenesis. *Aquatic Toxicology* 101, 500-511.

Saitoh K, Nagai F, Aoki N. 2001a. Several environmental pollutants have binding affinities for both androgen receptor and estrogen receptor alpha. *Journal of Health Sciences* 47, 495-501.

Saitoh M, Yanase T, Morinaga H, Tanabe M, Mu Y-M, Nishi Y, Nomura M, Okabe T, Goto K, Takayanagi R, Nawata H. 2001b. Tributyltin or triphenyltin inhibits aromatase activity in the human granulosa-like tumor cell line KGN. *Biochemical and Biophysical Research Communications* 289, 198-204.

Santos MM, Micael J, Carvalho AP, Morabito R, Booy P, Massanisso P, Lamoree M, Reis-Henriques MA. 2006. Estrogens counteract the masculinizing effect of tributyltin in zebraﬁsh. *Comparative Biochemistry and Physiology C* 142, 151–155.

Schlatterer B, Coenen TM, Ebert E, Grau R, Hilbig V, Munk R. 1993. Effects of bis(tri-n-butyltin)oxide in Japanese quail exposed during egg laying period: An interlaboratory comparison study. *Archives of Environmental Contamination and Toxicology* 24, 440-448.

Scott AP. 2012. Do mollusks use vertebrate sex steroids as reproductive hormones? Part I: Critical appraisal of the evidence for the presence, biosynthesis and uptake of steroids. *Steroids* 77, 1450–1468.

Scott AP. 2013. Do mollusks use vertebrate sex steroids as reproductive hormones? Part II. Critical review of the evidence that steroids have biological effects. *Steroids* 78, 268–281.

Sharan S, Nikhil K, Roy P. 2014. Disruption of thyroid hormone functions by low dose exposure of tributyltin: An *in vitro* and *in vivo* approach. *General and Comparative Endocrinology* 206, 155-165.

Shi H, Zhu P, Guo S. 2014. Effects of tributyltin on metamorphosis and gonadal differentiation of *Xenopus laevis* at environmentally relevant concentrations. *Toxicology and Industrial Health* 30, 297-303.

Shimasaki Y, Kitano T, Oshima Y, Inoue S, Imada N, Honjo T. 2003. Tributyltin causes masculinization in fish. *Environmental Toxicology and Chemistry* 22, 141-144.

Shimasaki Y, Oshima Y, Inoue S, Inoue Y, Kang IJ, Nakayama K, Imoto H, Honjo T. 2006. Effect of tributyltin on reproduction in Japanese whiting, *Sillago japonica*. Marine Environmental Research, 62, S245-S248.

Si J, Wu X, Wan C, Zeng T, Zhang M, Xie K, Li J. 2011. Peripubertal exposure to low doses of tributyltin chloride affects the homeostasis of serum T, E2, LH, and body weight of male mice. *Environmental Toxicology* 26, 307–314.

Si J, Han X, Zhang F, Xin Q, An L, Li G, Li C. 2012. Perinatal exposure to low doses of tributyltin chloride advances puberty and affects patterns of estrous cyclicity in female mice. *Environmental Toxicology* 27, 662-670.

Si J, Li P, Xin Q, Li X, An L, Li J. 2013. Perinatal exposure to low doses of tributyltin chloride reduces sperm count and quality in mice. *Environmental Toxicology* 30, 44-52.

Silva PV, Silva ARR, Mendo S, Loureiro S. 2014. Toxicity of tributyltin (TBT) to terrestrial organisms and its species sensitivity distribution. *Science of the Total Environment*, 466–467, 1037-1046.

Simpson ER, Mahendroo MS, Means GD, Kilgore MW, Corbin CJ, Mendelson CR. 1993. Tissue-specific promoters regulate aromatase cytochrome P450 expression. *Clinical Chemistry* 39, 317-324.

Spence SK, Bryan GW, Gibbs PE, Masters D, Morris L, Hawkins SJ. 1990. Effects of TBT contaminations on *Nucella* populations. *Functional Ecology* 4, 425-432.

Strand J, Jorgensen A, Tairova Z. 2009. TBT pollution and effects in molluscs at US Virgin Islands, Caribbean Sea. *Environment International* 35, 707-711.

Suzuki N, Tabata MJ, Kambegawa A, Srivastav AK, Shimada A, Takeda H, Kobayashi M, Wada S, Katsumata T, Hattori A. 2006. Tributyltin inhibits osteoblastic activity and disrupts calcium metabolism through an increase in plasma calcium and calcitonin levels in teleosts. *Life Sciences* 78, 2533-2541.

Tian H, Wu P, Wang W, Ru SG. 2015. Disruptions in aromatase expression in the brain, reproductive behavior, and secondary sexual characteristics in male guppies (*Poecilia reticulata*) induced by tributyltin. *Aquatic Toxicology* 162, 117-125.

Tingaud-Sequeira A, Ouadah N, Babin PJ. 2011. Zebrafish obesogenic test: a tool for screening molecules that target adiposity. *Journal of Lipid Research* 52, 1765–1772.

Tonk E.C., Pennings J.L., Piersma A.H. 2015. An adverse outcome pathway framework for neural tube and axial defects mediated by modulation of retinoic acid homeostasis. *Reproductive Toxicology* 55, 104-113.

Urishitani H, Katsu Y, Ohta Y, Shiraishi H, Iguchi T and Horiguchi T. 2013. Cloning and characterization of the retinoic acid receptor-like protein in the rock shell, *Thais clavigera*. *Aquatic Toxicology* 142-143, 203-213.

US EPA (2003). Ambient Aquatic Life Water Quality Criteria for Tributyltin (TBT) - Final. United States Environmental Protection Agency, Office of Water. EPA 822-R-03-031, December 2003.

US EPA (2008). Ecological Hazard and Environmental Risk Assessment for Tributyltin-containing Compounds (TBT). United States Environmental Protection Agency, Office of Prevention, Pesticides, and Toxic Substances, January 2008.

Vogeler S, Galloway TS, Lyons BP, Bean TP. 2014. The nuclear receptor gene family in the Pacific oyster, Crassostrea gigas, contains a novel subfamily group. *BMC Genomics* 15, 369.

Yang J, Oshima Y, Sei I, Miyazaki N. 2009. Metabolism of tributyltin and triphenyltin by Dall’s porpoise hepatic microsomes. *Chemosphere* 76, 1013-1015.

Yanik SC, Baker AH, Mann KK, Schlezinger JJ. 2011. Organotins are potent activators of PPAR_γ_ and adipocyte differentiation in bone marrow multipotent mesenchymal stromal cells. *Toxicological Sciences* 122, 476-488.

Zhang JL, Zuo ZH, Chen YX, Zhao Y, Hu S, Wang CG. 2007. Effect of tributyltin on the development of ovary in female cuvier (*Sebastiscus marmoratus*). *Aquatic Toxicology* 83, 174-179.

Zhang JL, Zuo ZH, He CY, Cai JL, Wang YQ, Chen YX, Wang CG. 2009a. Effect of tributyltin on testicular development in *Sebastiscus marmoratus* and the mechanisms involved. *Environmental Toxicology and Chemistry* 28, 1528*-*1535.

Zhang JL, Zuo ZH, He CY, Wu D, Chen YX, Wang CG. 2009b. Inhibition of thyroidal status related to depression of testicular development in *Sebastiscus marmoratus* exposed to tributyltin. *Aquatic Toxicology* 94, 62-67.

Zhang JL, Zuo ZH, Wang YQ, Yu A, Chen YX, Wang CG. 2011. Tributyltin chloride results in dorsal curvature in embryo development of *Sebastiscus marmoratus* via apoptosis pathway. *Chemosphere* 82, 437-442.

Zhang JL, Zuo ZH, Xiong JL, Sun P, Chen YX, Wang CG. 2013a. Tributyltin exposure causes lipotoxicity responses in the ovaries of rockfish, *Sebastiscus marmoratus*. *Chemosphere* 90, 1294-1299.

Zhang JL, Zuo ZH, Zhu WW, Sun P, Wang CG. 2013b. Sex-different effects of tributyltin on brain aromatase, estrogen receptor and retinoid X receptor gene expression in rockfish (*Sebastiscus marmoratus*). *Marine Environmental Research* 90, 113-118.

Zuo ZH, Cai JL, Wang XL, Li BW, Wang CG, Chen YX. 2009. Acute administration of tributyltin and trimethyltin modulate glutamate and N-methyl-D-aspartate receptor signaling pathway in *Sebastiscus marmoratus*. *Aquatic Toxicology* 92, 44-49.

Zuo Z, Wu T, Lin M, Zhang S, Yan F, Yang Z, Wang Y, Wang C. 2014. Chronic exposure to tributyltin chloride induces pancreatic islet cell apoptosis and disrupts glucose homeostasis in male mice. *Environmental Science and Technology* 48, 5179-5186.
