# Supplementary material for: Recommended Approaches to the Scientific Evaluation of Ecotoxicological Hazards and Risks of Endocrine-Active Substances
Source: Integr Environ Assess Manag. Author manuscript; Available in PMC 2018 Aug 1. (PMC6069525; doi:10.1002/ieam.1885)
Supplement: Supplement10 [file NIHMS1500348-supplement-Supplement10.docx]

**Supplemental Data S5**

**Draft Case Study for the Ecotoxicological Hazard and Risk Evaluation of Trenbolone**

Katherine K. Coady1, Melanie Gross2, Henrik Holbech3, Steven L. Levine4, Gerd Maack5, Mike Williams6, Gary Ankley7

1 The Dow Chemical Company, Toxicology and Environmental Research and Consulting, 1803 Building, Midland, MI 48674, USA.

2 wca, Brunel House, Volunteer Way, Faringdon SN7 7YR, UK.

3 Department of Biology, University of Southern Denmark, Campusvej 55, 5230 Odense M., Denmark.

4 Global Regulatory Sciences, Monsanto Company, 800 N. Lindbergh Blvd, St Louis, MO 63017, USA.

5 German Environment Agency (UBA), Wörlitzer Platz 1, D-06844 Dessau-Roßlau, Germany

6 CSIRO Land and Water, Waite Campus, SA 5064, Australia.

7 US Environmental Protection Agency, 6201 Congdon Blvd, Duluth, MN 55804, USA.

*Introduction*

Trenbolone (TRB) acetate is a synthetic anabolic agent that mimics the actions of natural steroids. TRB is registered for use as a growth promoter for cattle in a number of countries, including the USA, Canada, Australia and New Zealand, but not in the EU (Stephany, 2010). TRB acetate is predominantly excreted from cattle as 17α TRB (~90% of dosed TRB acetate) with 17β TRB (~10%) and trendione (~1%) as minor metabolites (Blackwell et al., 2014). Thus, there is the potential for environmental exposure via contribution of animal waste to the soil and water environment.

Based on both the mode of action and the expected route of exposure to the aquatic environment, vertebrate aquatic organisms were the focus of a case study exercise to determine hazard and risk associated with TRB exposure. This approach is consistent with other risk assessment approaches for TRB (Zoetis, 2014).

The overall objective of the TRB case study was to identify issues that impact or may impact hazard and risk assessment of endocrine active chemicals as part of the SETAC Pellston Workshop™ entitled “Environmental Hazard and Risk Assessment Approaches for Endocrine-Active Chemicals (EHRA) - A case studies approach for developing technical guidance to support decision-making”, held in Pensacola FL on Jan 31- Feb 5, 2016.

*Methods*

In order to conduct the case study of the environmental hazard and risk assessment of TRB, a team of environmental scientists with expertise in environmental fate, toxicity, and endocrine active chemistries was assembled to review and synthesize the available data for TRB as part of a SETAC Pellston Workshop. A literature search was conducted for TRB using the search terms summarized in **Table S5-1**. Following the literature search, a total of 805 references were identified for TRB. The abstracts of these papers were reviewed in order to select relevant exposure, fate, mode of action as well as toxicity information for TRB. Route of exposure, species, endpoints examined and experimental design aspects were all considered when determining if a paper was relevant for the hazard and risk assessment case study. The hazard and risk assessment for TRB was focused on aquatic vertebrate species since these organisms are at increased risk based on the mode of action of TRB (*i.e*. steroid hormone activity) and the major route of exposure (*i.e.* via animal waste run-off into aquatic systems). Following a review of the abstracts for relevance to the hazard and risk assessment of TRB, a total of approximately 155 references were selected for a more in depth review by the TRB case study team (included as **Supplemental Information “Selected literature Trenbolone Case Study.pdf”**). These references included environmental fate and exposure information for TRB, *in vitro* assays conducted with TRB, as well as invertebrate, fish, amphibian, avian, and mammalian toxicity studies with TRB.

Members of the TRB case study work group reviewed the selected studies to further determine relevance for the hazard and risk assessment of TRB. If studies were relevant for the hazard and risk assessment case study, they were reviewed for reliability using the ToxRTool, a simple Excel-based system derived from the widely-used Klimisch method (Klimisch et al., 1997; Schneider et al., 2009; tool available at <https://eurl-ecvam.jrc.ec.europa.eu/about-ecvam/archive-publications/toxrtool> ). Only reliable and relevant data were ultimately used to conduct the TRB hazard and risk assessment case study.

Relevant and reliable toxicity data for TRB was organized in accordance with the OECD Guidance Document 150 on Standardised Test Guildelines for Evaluated Chemicals for Endocrine Disruption (OECD, 2012). The conceptual framework is a valuable tool for use in organizing or grouping existing data into different levels of complexity. It is a logical and scientifically developed approach that can be used to assist in evaluating an existing data set for evidence of effects on the endocrine system. Generically, the conceptual framework consists of 5 levels with level 1 corresponding to the lowest level of complexity and level 5 data being the most biologically complex (*i.e*. apical studies).

Following the organization of all available relevant data according to the OECD conceptual framework, a simplified weight of evidence (WoE) evaluation for TRB and endocrine activity was conducted. A full WoE evaluation was not conducted for TRB in the hazard and risk assessment case study since the androgenic mode of action has previously been well established for TRB (Lange et al., 2002; Lee et al., 2007; Kolok and Sellin, 2008).

Environmental exposure information for TRB was compared to NOEC and LOEC values from relevant and reliable toxicity studies in a screening level risk assessment.

As the hazard and risk assessment for TRB was conducted, participants in the TRB case study group were tasked with identifying issues that impacted the hazard and risk assessment of TRB. These issues were further examined and discussed for other endocrine active case study chemicals within the EHRA SETAC Pellston Workshop.

*Exposure considerations*

The principle route of TRB entering the environment is through leaching of wastes (urine and feces) from cattle dosed with TRB acetate. There is some evidence that aerial transport of TRB associated with dust can also occur, with potential implications for terrestrial vertebrates (Blackwell et al. 2011).

Measurement of both TRB isomers in run-off from cattle feedlots is generally infrequent (<10%) and at ng/L concentrations (Table S5-2). TRB is predominantly excreted from cattle as 17α TRB (~90% of dosed TRB acetate) with 17β TRB (~10%) and trendione (~1%) as minor metabolites (Blackwell et al. 2014). This is reflected in measured environmental concentrations from a number of studies where 17α TRB was greater than 17β TRB in liquid manure (0.4-1.8 μg/kg 17α compared with 0.06-0.16 μg/kg 17β), dung (4.7-75 μg/kg 17α; 0.5-4.2 μg/kg 17β), soil (<LOQ-11800 μg/kg 17α; <LOQ-6100 μg/kg 17β), final effluent water (0.3-1.5 μg/L 17α; 0.02-0.1 μg/L 17β) and run-off (<0.034 μg/L 17α; <LOQ 17β) from cattle feedlots (Schiffer et al., 2001; Bartelt-Hunt et al., 2012, Khan and Lee, 2012, Webster et al., 2012; Jones et al., 2014). One study, however, measured maximum concentrations of 17β TRB similar to or even greater than those of 17α TRB (0.001-0.023 μg/L 17α; 0.003-0.162 μg/L 17β) in run-off from fields fertilized with TRB-implanted cattle manure (Gall et al., 2011). Another two year monitoring program recorded a maximum measured concentration of 17β TRB of 270 ng/L, while not detecting 17α TRB (Bartelt-Hunt et al., 2012).There was no explanation given for these results, which are also inconsistent with fate studies. For example, the affinity of 17β TRB to solids is expected to be greater than 17α TRB, based on a number of sorption studies (Khan et al., 2009; Card et al., 2012; Qu et al., 2014). Also, Qu et al. (2014) found that sorption of 17β TRB was less reversible than 17α TRB, indicating a lesser degree of 17β TRB mobility in soils. In this same study, the degradation rate of 17β was greater than 17α in soil solution. Preferential degradation of 17β TRB has also been observed in other soil and water degradation studies (Khan and Lee, 2008, 2010; Cole et al., 2015). Photolysis of TRB is also isomer specific, with degradation of 17β TRB more rapid than 17α TRB and reversion of hydroxylated photoproducts (also endocrine active) to the parent compounds occurring to a greater extent for 17α TRB (Kolodziej et al., 2013; Qu et al., 2013).

Stereochemistry of TRB is therefore important for considering its environmental risk with 17α TRB likely to be the most relevant metabolite in the aquatic environment due to its higher input concentrations, persistence and mobility. Furthermore, there is also limited evidence of equivalent potency of 17α TRB with 17β TRB to fish, possibly due to partial *in vivo* conversion of 17α to 17β TRB (Jensen et al., 2006). 17β TRB, however, may still be present at environmental concentrations close to reported environmental concentrations (low ng/L concentrations) based on its concentrations of final feedlot effluent collected for irrigation purposes measured in the range of 0.02-0.1 μg/L (Khan and Lee, 2012).Also, interconversion from trendione to 17β TRB under suitable conditions can also contribute to its environmental presence (Cole et al., 2015).

*In vivo Toxicokinetics/Toxicodynamics in fish*

Schultz et al. (2013) used single concentrations of 17β TRB (e.g. generally 0.5 μg/L) to investigate toxicokinetics and toxicodynamics of TRB in adult female fathead minnows and rainbow trout. Using the two fish species provided comparison between synchronous (trout) and asynchronous (minnows) breeders. In both species, 17β TRB was rapidly adsorbed, reaching a plateau at approximately 8 hours. Plasma concentrations were approximately 2-6 x higher in trout than minnows. Depuration was also measured in trout and this was also fairly rapid with a t1/2 of 14.5 hours. Thus, changes in environmental concentrations are predicted to be rapidly reflected in changes in fish plasma and other tissues.

*Level 2: In vitro data* A summary of the *in* vitro data that was reviewed for the TRB case study is available in **Supplemental Information (Trenbolone data summaries.xls)**. In competitive receptor binding assays with recombinant androgen receptor (rAR), TRB displaced R1881 as much as 5α-dihydro-testosterone (DHT) with IC50 values in the range of 1.3 to 5.7 x 10-8 M (Kim et al., 2010). 17β TRB has a similar binding affinity as DHT with the recombinant hAR (Bauer et al., 2000). However, values for the metabolites 17α TRB and trendione have approximately 5% and 0.4% of the relative activity of 17β TRB, respectively (Bauer et al., 2000, Blake et al., 2010). The significantly lower affinity of 17α TRB compared to 17β TRB reported by Bauer et al. (2000) for the human AR in competitive receptor binding assays is consistent with results from AR transcriptional activation assays using cell lines derived from human tissue (Blake et al., 2010). Overall, results obtained from competitive receptor binding assays with 17β TRB using mammalian ARs is consistent with results obtained in functionally equivalent AR transcriptional activation assays with mammalian cell lines (Blankvoort et al., 2001; Blake et al., 2010; Wilson et al., 2002). In support of the MoA shown in AR binding and transcriptional activation assays, Wilson et al. (2002) confirmed nuclear translocation of the AR after binding 17β TRB and at concentrations as low as 1 pM.

Competitive receptor binding assays and transcriptional activation assays performed with 17β TRB and AR receptors from fish species have shown comparable affinities to those reported values for mammalian assays (Wilson et al., 2002; Wilson et al., 2004; Katsu et al. 2007). 17β TRB has been shown to induce AR-dependent spiggin production in stickleback kidney cell culture assay with a lowest observed effect concentration (LOEC) of 10-10 M and a maximum response at 10-6 M (Jolly et al., 2006). Concentrations of 17β TRB that produce induction of spiggin are comparable to IC50 values for 17β TRB in competitive receptor binding assays with an AR derived from fathead minnow.

Additional work has been published characterizing binding of 17β TRB to other nuclear receptors responsible for endocrine function. 17β TRB was shown to have a higher affinity for the bovine progesterone receptor than progesterone, however, significantly lower binding affinity was demonstrated for 17α TRB and trendione (Bauer et al., 2001). In addition, 17β TRB has been shown to poorly compete with DHT for the human sex-hormone binding globulin and failed to activate glucocorticoid receptor mediated gene transcription (Bauer et al., 2001; Wilson et al., 2002). Forsgren et al. (2014) assessed the relative estrogenicity of the three metabolites of TRB acetate (*i.e.* 17α TRB, 17β TRB, and trendione) using an *in vitro* rainbow trout cell line and the induction of the estrogen sensitive mRNA VTG biomarker. 17α TRB showed no estrogenic activity, while both 17β TRB and trendione showed some estrogenic activity. 17β TRB also showed some estrogenic activity in estrogen-sensitive, transgeneic medaka larvae at the relatively high concentration of 100 μg/L (Kurauchi et al., 2008).

17β TRB (CAS No. 10161-33-8) has been evaluated in U.S. EPA’s ToxCast™ program, which is a collection of high throughput screening assays that expose living cells or isolated proteins to chemicals to screen for many different types of biological activity (epa.gov/ncct/toxcast/index.html). Results are primarily reported as AC50 values, which are the chemical concentrations required to cause 50% activity in the assay endpoint/biological target. 17β TRB was evaluated in a total of 428 assay endpoints in ToxCast™ (Download date of November 17, 2015). 17β TRB showed activity (*i.e.* measurable AC50 values) in 24% of the assays (n= 101 positive hits total), nine of which are designated as cytotoxicity assays in the ToxCast™ battery. Based on the results from these nine cytotoxicity assays, the lower cytotoxicity limit for 17β TRB was determined to be 1.52 μM. While 17β-trenbolone interacted with multiple biological targets within the ToxCast™ battery, the biological activity below the lower cytotoxicity limit is of most interest for determining the specific activity of 17β-trenbolone. 17β-trenbolone had AC50 values that were below the lower cytotoxicity limit for the following biological targets: 1) androgen receptor, 2) progesterone receptor, 3) estrogen receptor, 4) mineralocorticoid receptor, 5) glucocorticoid receptor, 6) serine proteinase inhibitor, and 7) cytokines associated with chemotactic activity for monocytes and basophils (**Table S5-3**). The results for 17β-trenbolone in the endocrine specific high throughput assays from the ToxCast™ database (available at: actor.epa.gov/edsp21/) indicated activity in assays related to the estrogen, androgen, and, in one case, the thyroid receptor (**Table S5-4**). Specifically, 17β-trenbolone was active in 10/11 androgen receptor assays, 15/18 estrogen receptor assays, and 1/3 thyroid receptor assays for which it was evaluated (**Table S5-4**). Notably, nine of the androgen receptor assay AC50 values and five of the estrogen receptor assay AC50 values were lower than the lower cytotoxicity limit for 17β-trenbolone, indicating specific activity in both the estrogen and androgen receptor high through-put screens in ToxCast. The single positive hit in the thyroid receptor assay displayed an AC50 that was much higher than the lower limit of cytotoxicity for 17β-trenbolone, indicating that this positive hit was most likely non-specific.

*Level 3: In vivo mechanistic data*

*In vivo* mechanistic data for TRB is summarized in **Supplemental Information (Trenbolone data summaries.xls)**. Various types of mechanistic endpoints have been examined in response to TRB exposure including: genes and proteins known to be sensitive to endocrine active modes of action, gonad and kidney histopathology, secondary sex characteristics, organ weights, and sex steroid concentrations. No Observable Effect Concentrations (NOEC) and Low Observable Effect Concentrations (LOEC) for the mechanistic endpoints varied over several orders of magnitude. Endpoints, such as changes in plasma vitellogenin, male gonad histopathology changes, and presence of male secondary sex characteristics in female fish were all sensitive mechanistic endpoints in response to TRB exposure.

There are quite a number of studies investigating effects of mechanistic-level endpoints in both short- and medium-term studies in fish. Many of these studies used TRB as a model AR agonist to probe, for example, interactions with known/possible AR antagonists like flutamide, vinclozolin and bisphenol A(e.g., Ankley et al., 2004; 2010; Martinovic et al., 2007; Garcia-Reyero et al., 2009; Collette et al., 2010; Ekman et al., 2012). These studies typically only employed one concentration of TRB, so their contribution to identifying NOEC/LOEC values is limited.

Several studies have demonstrated morphological masculinization of adult females (e.g., nuptial tubercle formation in fathead minnows; Ankley et al. 2003; 2004; 2010; Martinovic et al. 2007; Seki et al., 2006; anal fin elongation in Eastern mosquitofish; Brockmeier et al., 2013 and Western mosquitofish; Sone et al., 2005). This is, of course, consistent with activation of the AR. Also increased male masculinization has been observed as increased maturity of male zebrafish gonads (Baumann et al., 2013; 2014).

Aquaculture studies using high concentrations of trenbolone acetate have also demonstrated the masculinization effect on a wider range of fish species (i.e. effect is not limited to standard test fish species), including black crappie, Nile Tilapia and channel catfish (Arslan and Phelps, 2004, Bart et al., 2003, Davis et al., 2000). Depending on the exposure regime and concentrations, up to 100% male populations were achieved. Of interest is the study by Davis et al. (2000), which showed that although treatment with TBA resulted in all male populations, treatment did not functionally masculinize catfish. Masculinized males paired with untreated females resulted in spawns, but the eggs were not fertilized and did not develop.

Other studies have reported histological changes of gonads of both male and female fish (e.g. fibrosis, atresia, tubule length and egg debris in oviducts; Velasco-Santamaría et al., 2009; 2013; Hemmer et al., 2011; Boettcher et al., 2011)

There also have been a number of studies documenting decreases in gonadal sex steroid (T, E2) synthesis and/or plasma T/E2 concentrations in both sexes, but especially females, exposed to TRB (e.g., Ankley et al., 2003; Garcia-Reyero et al., 2009; Ekman et al., 2011; Zhang et al, 2008b; Shultz et al., 2013). Down-regulation of steroid synthesis is consistent with over-stimulation of the HPG axis by exogenous androgen. For example, 11-keto T decreased in juvenile male rainbow trout and in adult male zebrafish (Massart et al., 2015; Boettcher et al., 2011). Concurrent with decreased steroid synthesis (i.e. E2) in adult females are observations of decreased plasma VTG levels (or hepatic expression of VTG mRNA) associated with TRB exposure, consistent with decreased E2 signaling (Ankley et al., 2003; 2004; 2010; Miracle et al., 2006; Martinovic et al., 2007; Ekman et al., 2011; Zhang et al., 2008b; Shultz et al., 2013).

Some authors have investigated the development of androgen responsive biomarkers using TRB as a model compound. Allen et al. (2008) reported on an intercalibration exercise using the stickleback screening assay in three laboratories. A significant induction of spiggin was observed in females exposed to 5000 µg/L (nominal) at all three laboratories. Villeret et al. (2013) found a concentration dependent increase in Kidney Epithelium Height (KEH) in European bullhead fish (*Cottus sp.*) exposed to trenbolone acetate (1.26 and 6.5 µg/L).

A number of studies have evaluated changes in gene expression in TRB-exposed fish at different life-stages, using both targeted (PCR) and non-targeted (e.g., microarray) techniques. For example, Leet et al. (2015) observed down-regulation of several genes involved in steroid synthesis in larval fathead minnows exposed to TRB, consistent with over-stimulation of the HPG axis by exogenous steroids. Ekman et al (2011) reported analogous results for several HPG-responsive genes in adult fathead minnows exposed to TRB during a time-course study. This over-stimulation could also explain the observation by Sone et al. (2005) where mRNA expression of both AR genes was up-regulated by all concentrations of TB in the anal fin of adult female Western mosquitofish after 3 days exposure but at or below control level after 28 days exposure. Dorts et al. (2009) demonstrated downregulation of liver VTG and brain aromatase and ER upregulation in fathead minnows exposed to 17β TRB. Collectively, the preponderance of transcriptomic responses in the Dorts et al. (2009) study indicated that genes in the fathead minnow ovary were generally down-regulated, supporting the inhibitory effect of androgens on estrogen related functions in the ovary. Garcia-Reyero et al. (2009) used a microarray approach to evaluate ovarian gene expression in TRB-exposed fathead minnows and noted several up- and down-regulated genes, the majority of which were not seemingly directly related to AR interactions. Brockmeier et al. (2013) used a custom microarray to investigate transcriptomic responses in the liver of female mosquitofish (*Gambusia holbrooki*) that were exposed to 1 μg/L 17β TRB for 14-days. Many metabolic processes were increased in response to TRB exposure, including lipid metabolic processes, regulation of protein metabolic processes, lipoprotein metabolic processes, cholesterol biosynthesis processes, cholesterol transport and metabolism.

Non-targeted proteomic and metabolomic studies with TRB-exposed fathead minnows (e.g., Collette et al., 2010; Ekman et al., 2011; Martyniuk et al., 2009) have yielded results qualitatively similar to those of Garcia-Reyero et al. (2009), in that the majority of the observed ‘omic’ changes could not be directly related to known AR-signaling pathways. Martyniuk and Denslow (2012) conducted a review of the ‘omic’ responses of several known androgenic chemicals in teleost fish and noted the difficulty in teasing apart molecular responses that are a result of general toxicity or are the results of a chemical’s mode of action. In the case of TRB, significant cell processes that were affected by TRB-regulated protein included lymphocyte differentiation, xenobiotic clearance, LDL oxidation, proliferation of smooth muscle cells, permeability of blood vessels, and DNA degradation. Many of these genomic responses are similar to pathways identified in a protein stress interactome for fish and mammals.

Overall, the Level 3 studies conducted with TRB in fish clearly demonstrate that it is a potent AR agonist that can affect multiple life stages. Further, as described below, the mechanistic studies provide a basis for constructing adverse outcome pathways (AOPs) for the effects of androgens like TRB on fish as a basis to link focused mechanistic data to apical effects.

*Level 4: In vivo Apical Effects-Short Term*

*In vivo* apical effects data for TRB is summarized in **Supplemental Information (Trenbolone data summaries.xls)**. Various population-level apical effects (primarily in fish and amphibians) have been examined in response to TRB exposure including effects on survival, growth, reproduction, hatching success, and alterations in sex ratios. No Observable Effect Concentrations (NOEC) and Low Observable Effect Concentrations (LOEC) for these apical, population-level endpoints varied over several orders of magnitude. Endpoints related to decreases in fish reproduction and altered sex ratios were all sensitive apical endpoints in response to TRB exposure in level 4 studies.

Fish

The effects of 17β TRB and 17α TRB on survival, growth, sex ratios and reproduction has been evaluated in several species of fish **Supplemental Information (Trenbolone data summaries.xls)**. In these studies, the NOEC and LOEC values for population level *in vivo* effects range from 0.0006->50 μg/L and from 0.0006-100 μg/L, respectively.

Consistent with expectations for a potent endocrine-active chemical, TRB can adversely affect fish both during two windows of susceptibility--early development/sexual differentiation (organizational effects) and active reproduction (activational effects). Relative to reproductive effects, Ankley et al. (2003) conducted a 21-d fathead minnow reproduction assay in which the LOEC for effects on egg production was 0.05 μg/L (0.027 ug/L measured) and the NOEC was 0.005 μg/L (ca. 0.002 μg/L). Significantly, a number of mechanistic endpoints were measured in that same study and exhibited the same LOEC/NOEC values (e.g., increases in tubercles in females; decreased plasma VTG in females). In two shorter 7-day exposures to 17β TRB, medaka fish showed reduced reproduction at 0.5 and 5 μg/L (Park et al., 2009; Zhang et al., 2008a) and a NOEC for reproductive effects at 0.05 μg/L.

The period of sexual differentiation seem to be a very sensitive period to adversely affect fish by exposure to TRB: Six studies with zebrafish covering the period of sexual differentiation consistently report skewed sex ratio and all male populations around 0.01 µg/L (Holbech et al., 2006, Larsen et al., 2010; Morthorst et al., 2010; Boettcher et al., 2011; Baumann et al., 2013; 2014). Three of these studies investigated reversibility of this effect during long depuration periods and all found the phenotypic sex change irreversible. Orn et al (2006) investigated VTG, sex ratio, and gonad morphology in zebrafish and medaka exposed to 17β TRB from 1-60 days post hatch. The reversal to a male phenotype was more sensitive in zebrafish (LOEC = 0.05 μg/L; 100% sex reversal to males) as compared to medaka (no effect at 0.05 μg/L). The sensitivity to TRB during zebrafish sex differentiation could be a consequence of the period of juvenile hermaphroditism that zebrafish undergoes, and medaka may be less sensitive based on a lack of a juvenile hermaphroditism stage in the sexual differentiation process. A feeding study with blue tilapia also produced more than 98% males after 28 days exposure of undifferentiated fingerlings but the lowest tested dose was 25 mg/kg diet so comparison to aquatic exposure is difficult. The strength of effect on phenotypic sex (occurring below increased mortality) is that it is regarded as endocrine specific (OECD, 2012).

Amphibians

The effects of 17β TRB and 17α TRB on amphibian survival, growth, and development has been evaluated in several species of frogs (Li et al., 2015; Olmstead et al., 2012; Finch et al., 2013). In these studies, the NOEC and LOEC values for population level *in vivo* effects range from 0.033 – 10 μg/L and from 0.078 – 10 μ/L, respectively. Unique to amphibians is the effect of laryngeal hypertrophy and possible suffocation in tadpoles in response to exposure to TRB. The effects of suffocation/survival in tadpoles exposed to TRB occurred at concentrations ranging from 0.1 to > 0.5 μg/L (Li et al., 2015; Olmstead et al., 2012; Finch et al., 2013). *Pelophylax nigromaculatus* tadpoles exposed to 17β were completely sex reversed to males at 0.1 μg/L (Li et al., 2014). Thus, the androgenic effects of TRB among amphibian sex ratios are similar to those observed in fish.

*Level 5: In vivo Apical Effects- Long Term*

*In vivo* apical effects data from long term, multiple life stage or multiple generational toxicity tests with TRB are summarized in **Supplemental Information (Trenbolone data summaries.xls)**. Various population-level apical effects in multiple generation toxicity studies (primarily in fish) have been examined in response to TRB exposure including effects on survival, growth, reproduction, hatching success, and alterations in sex ratios.

Cripe et al. (2010) conducted a three-generation flow-through study exposing Sheepshead minnow (*Cyprinidon variegatus*) to TRB. Reproduction was the most consistently sensitive parameter measured. Reproductive rate (daily reproductive rate per female) was significantly reduced at 0.87, 0.027 and 0.027 µg/L (mean measured concentrations) in the F0, F1 and F2 generations, respectively. The corresponding NOECs were 0.13, 0.007, and 0.007 µg/L in the F0, F1 and F2 generations, respectively. Cumulative embryo production was affected at 0.87, 0.13, and 0.027 µg/L in the F0, F1 and F2 generations, respectively (corresponding NOECs were 0.13, 0.027, and 0.007 µg/L, respectively). This study provided evidence of some effects occurring at lower concentrations in subsequent generations in comparison to F0. Additionally, TRB was evaluated in a multigenerational toxicity study with the medaka (*Oryzias latipes*) as a part of the validation of the Medaka Extended One Generation Reproduction Test (MEOGRT). In this multigenerational study, the NOEC for fecundity was reduced in the F1 generation (i.e. fecundity NOEC = 0.013 μg/L) as compared to the F0 generation (i.e. fecundity NOEC = 0.032 μg/L). The full TRB concentration series could not be evaluated among the F2 generation in this study since reproduction was entirely inhibited in the F1 generation at 0.032 μg/L (USEPA 2013.

Boettcher (2011) conducted a two-generation test with zebrafish starting with fertilized eggs from adults pre-exposed for 21 days and continuing until the F2 generation reached 35 days (in total 177 days). The NOEC for F1 sex ratio was 0.001 µg/L and LOEC was 0.004 µg/L. Boettcher also observed decreased cumulative fecundity and increased fertilization at the two lowest test concentrations in F0. But no effect at higher concentrations (0.015 and 0.031 µg/L).

*Population Modeling*

Miller and Ankley (2004) developed a fathead minnow population model and utilized data from Ankley et al. (2003) to project that fish exposed to a nominal concentration of >0.05 μg trenbolone/L (0.027 μg/L measured) would have an average equilibrium population size that approached zero. Accordingly, the Miller and Ankley analysis provides a critical link between lab-based fecundity data and population-level effects of TRB.

*Weight of Evidence for Androgenic Activity*

The evidence from *in vitro* assays from the peer-reviewed literature, the high throughput bioassay results in the ToxCast battery, along with mechanistic and apical effects noted in fish and amphibians all indicate that TRB has an androgenic mode of action.

Data from *in vitro* assays also indicate that TRB is active with other endocrine pathway receptors including the progesterone and estrogen receptors, however this activity occurs at higher concentrations than the androgenic activity of TRB.

*Risk Assessment*

Maximal concentrations of TRB in the aquatic environment (primarily in areas that are adjacent to livestock rearing facilities) can overlap with NOEC and LOEC values derived in toxicity studies with aquatic organisms. However, it should be noted that exposure data is very limited.

*Sources of Uncertainty/ Identification of Issues with Hazard and Risk Assessment of Endocrine Active Chemicals*

Through the process of examining the fate, exposure, as well as the *in vitro* and *in vivo* toxicity data among various environmental species, several issues emerged for TRB that require further discussion in the context of conducting hazard and risk assessment of endocrine active chemicals. These issues include:

- Interferences from systemic toxicity or other modes of action
  - No evidence of systemic/non-target toxicity was noted in the papers reviewed at a concentration of up to 50 μg/L for 21-d in fish (Ankley et al., 2003). We suspect that for potent, targeted chemicals like TRB and/or EE2, this type of interference is far less likely to be problematic than for endocrine-active pesticides or most types of industrial chemicals.
- Differences in species responses and sensitivity to endocrine active chemicals (also difference in life-stage sensitivity)
  - There is strong evidence for conservation of AR-mediated apical effects across vertebrate species. For example, Ankley and Gray (2013) compared responses of the rat and fish EDSP Tier 1 test systems to a number of known endocrine-active chemicals, including TRB. The TRB analysis showed that pathway-based responses (tubercle induction in female fathead minnows; increases in organ weights of androgen-responsive tissues in the Hershberger [rat] assay) indicated cross-species conservation of androgen signaling in terms of apical outcomes. This is consistent with the theoretical predictions of LaLone et al. (2013) who conducted a SeqAPASS homology analysis of structure of the AR based on data from NCBI, using the bovine AR as a “probe” sequence. This analysis showed that representative vertebrate, but not invertebrate or plant species possessed proteins orthologous to the bovine AR.
  - Although invertebrate toxicity data for TRB are quite limited, what is available confirms these relative susceptibility predictions. For example, LaLone et al. (2013) conducted a *Ceriodaphnia dubia* life cycle test with TRB, and found that the daphnids were indeed several orders of magnitude less sensitive than fish to the reproductive effects of the androgen. Similarly, in molluscs, no AR has been identified. This type of predictive insight could help prioritize/guide testing for certain types of EDCs.
  - Regarding sensitive life-stages, TRB exposure during sexual development (organizational events) causes irreversible effects on phenotypic sex (at least in zebrafish) whereas reproductive effects in adults (activational events) seem to be generally reversible.
  - Internal concentrations of TRB were 2-6 times greater in trout blood plasma as compared to fathead minnow plasma following equivalent exposure regimens in a toxicokinetic/toxicodynamic study (Schultz et al., 2013). Binding of TRB to trout plasma also appeared to be higher compared with minnows (*i.e.* the unbound percent in trout = 4.5% and the unbound percent in minnows = 18.3%). This difference in unbound concentrations of TRB is likely due to differences in plasma binding proteins in trout vs. minnows. Trout are known to have sex hormone binding globulin (SHBG), while the presence of SHBG has not been identified in minnows (Schultz et. al, 2013). These plasma binding differences could contribute to differences in toxic effects among various fish species.
  - Effects on decreased VTG levels in response to 21-day TRB exposure were slightly more sensitive in medaka liver as compared to fathead minnow and zebrafish levels in blood plasma (Seki et al., 2006).
  - Following exposure to TRB during early development, including the sensitive window of sexual differentiation, medaka and zebrafish were assessed for effects on VTG, sex ratio and gonad morphology (Örn et al 2006). Zebrafish were more sensitive than medaka to TRB exposure in regard to sex ratio. The explanation for this was attributed the fact that the developmental history of zebrafish differs from medaka *(i.e.* zebrafish have a juvenile hermaphroditism stage whereas medaka do not). Thus, fish species with a juvenile hermaphroditism phase during development may be more sensitive to androgenic compounds, such as TRB. This could be an important consideration when determining the most appropriate test species for evaluating chemicals suspected of having androgenic activity.
  - The effects of TRB exposure differ among different vertebrate taxa. In developing frogs, exposure to TRB results in hypertrophy of laryngeal muscles (since these are androgen- responsive tissues) which can lead to suffocation and death (Li et al., 2015; Olmstead et al, 2012). These type of effects are not noted among fish species. However, reproductive effects in fish due to TRB exposure are more sensitive than suffocation/survival effects in frogs/tadpoles.
- Non-monotonic dose responses
  - Ankley et al. (2003) described non-monotonic (U-shaped) dose-response relationships for plasma T, E2 and VTG concentrations in females exposed to TRB for 21 d, but upward inflection of the curve occurred only at concentrations orders of magnitude higher than those in the environment, so this observation would be of little significance to assessing risk.
  - Much more problematic/challenging is the occurrence of non-monotonic data for mechanistic endpoints at lower biological levels of organization in shorter-term tests as exemplified by Ekman et al. (2011) who tested TRB with adult fathead minnows in a design where the animals were exposed for 8 days, and then allowed to recover for 8 days, with a total of eight periodic sampling dates over the time course. Several examples of non-monotonic dose-response relationships for steroid synthesis/concentrations and/or HPG gene expression were observed. There is a plausible biological basis for this phenomenon that was described by Ankley and Villeneuve (2015), who conducted a meta-analysis to explore the phenomenon of system compensation/adaptation in the context of testing for endocrine-active chemicals (including TRB). This analysis could contribute to deliberations associated with the identification on non-monotonic dose-response curves as a cross-cutting issue.
- Exploring the connection of mechanistic and apical endpoints
  - A significant challenge in regulating endocrine-active chemicals involves making the linkage between mechanistic endpoints indicative of perturbation of endocrine pathways of concern (e.g., AR activation in the case of TRB) and apical, population-relevant responses meaningful to risk assessment. The overall TRB dataset offers the opportunity to explore this challenge in the context of prioritization, screening and testing through use of the AOP framework. That is, there has been focused testing at multiple biological levels of organization in fish that has featured collection of different types of ‘omic data (i.e., transcriptomics, Garcia-Reyero 2009; proteomics, Martyniuk et al. 2009; metabolomics, Ekman et al. 2011), data directly indicative of endocrine function (HPG-oriented genes, steroids, VTG) and apical data (in some cases from the same study, e.g., Ankley et al. 2003), and even prediction of population-level responses (Miller and Ankley 2004; 2007).
  - Sex ratio can be affected by endocrine-active chemicals (including TRB) and is an apical population relevant endpoint. Models for population effects have been discussed for chemicals that affect sex ratios (e.g., Hazlerigg et al. 2014).
- Determining when endocrine effects are adverse
  - Ankley et al. (2003) noted a concentration-dependent increase in the wet weight of female fathead minnows exposed to TRB for 21-d, starting at a concentration of about 0.05 μg/L, which also corresponded to the LOEC for decreased egg production and changes in some mechanistic endpoints (e.g., decreased plasma VTG) in females. This growth effect almost certainly is mediated through AR activation, reflecting the anabolic nature of the steroid. Is weight gain a positive response?
  - Somewhat analogously, numerous studies have shown morphological masculinization of adult females exposed to TRB (see above). Although this is an excellent biomarker of AR activation, its direct contribution to adversity is uncertain and difficult to define because masculinization occurs at the same concentrations where reduced fecundity is noted.
  - Bertram et al. (2015) reported an alteration of mating behavior in guppies, which they considered could affect male reproductive success and Saaristo et al. (2013) observed changes in female mating behavior in Eastern mosquitofish at 0.006 µg/L. In a new study by Heniz et al. (2015), the risk taking behavior was changed in both male and female guppies. How should behavioral studies be considered within the OECD CF? Are their study designs and endpoints sufficiently robust to be considered at this stage?
- Study Design limitation
  - A potential issue is the use of single concentrations, which occurred in several papers. Studies using single or two concentrations can be used to inform mechanisms, but present issues such as demonstration of concentration dependence. They are usually mainly used as supporting information when considering overall NOEC/LOECs. So they may have use in a hazard assessment, but are less useful for risk assessment.
  - Mechanistic studies or screening assays typically use fewer replicates, fewer concentrations (≤3), with sometimes fairly wide concentration spacing (e.g. one order of magnitude). They are usually intended to provide information on a mechanistic endpoint, which would then trigger further testing for any apical effects. When NOECs and LOECs are derived in/from screening studies, these points should be borne in mind.
  - The translation of the effects from mammalian studies to population related effects is often not possible, especially as most mammalian studies do not have classical apical endpoints and none of the assessed mammalian studies documented an analytical confirmation of the used exposure concentrations.
- Exposure limitations
  - Photolysis of TRB isomers was found to be rapid (and variable depending on the isomer), however reversion of hydroxylated degradation products to the parent TRB means that its persistence in the aquatic environment may be greater than its photolability might predict (Qu et al. 2013). Furthermore, an ectoxicological assessment of TRB photoproducts (produced from a 100 ng/L TRB solution) demonstrated they can increase the number of vitellogenic stage follicles and reduce whole body E2 production in Japanese medaka (*Oryzias latipes*) (Kolodziej et al. 2013). In a separate study, degradation of 17β TRB spiked in sediments led to low to non-detectable concentrations of 17β  in solution, along with the formation of 17α TRB and trendione (Sangster et al. 2014). Fathead minnows exposed to the sediments for 7-14 days had significantly reduced VTG expression, despite the degradation of TRB, with the formation of undetermined endocrine active metabolites a potential reason for the noted effects. These studies highlight the need to also consider degradation products during exposure assessments.

*Conclusion*

- TRB has an androgenic mode of action that is apparent from examining *in vitro*, as well as *in vivo* toxicity data from fish and amphibians.
- Concentrations of TRB in runoff from livestock facilities can contain maximal concentrations of TRB that overlap with effects observed in toxicity lab studies with fish and amphibians.
- Species and life-stage differences are apparent with TRB exposure. Fish appear to be most sensitive with amphibians having generally similar sensitivity to TRB. In the limited invertebrate toxicity data that was available for TRB, it was apparent that invertebrates were less sensitive to TRB as compared to fish and amphibians. Decreased survival and reproduction and altered sex ratio changes (i.e. greater proportion of males) in fish are among the most sensitive, apical endpoints. Data from multiple generational fish toxicity studies and early life stage studies generally yielded the lowest population-level adverse NOEC and LOEC values.
- Overall, mechanistic and apical endpoint responses to TRB exposure occur over similar concentration ranges. Some sensitive mechanistic endpoints are altered vitellogenin levels, alterations in gonadal histopathology, and appearance of male secondary sex characteristics in female fish. These sensitive mechanistic endpoints are consistent with the known androgenic mode of action of TRB.

*References*

*Klimisch, H.J., Andreae, M., Tillmann, U. 1997. A Systematic Approach for Evaluating the Quality of Experimental Toxicological and Ecotoxicological Data, Regulatory Toxicology and Pharmacology Vol 25, pp 1–5.*

*OECD, 2012. Guidance Document on standardised Test Guidelines for evaluating chemicals for Endocrine Disruption. OECD series on testing and assessment NO. 150. ENV/JM/MONO(2012)22.*

*Schneider, K., Schwarz, M., Burkholder, I., Kopp-Schneider, A., Edler, L., Kinsner-Ovaskainen,, A., Hartung, T., Hoffmann, S. 2009. ToxRTool", a new tool to assess the reliability of toxicological data. Toxicology Letters Volume 189: pp 138-144*

*US EPA 2013. Validation of the Medaka Multigeneration Test: Integrated Summary Report. U.S. Environmental Protection Agency Endocrine Disruptor Screening Program Washington, D.C. available at: http://www.oecd.org/env/ehs/testing/MMT%20ISR%20final.pdf"*

*Zoetis, 2014. Environmental Assessment for Synovex ONE (Estradiol Benzoate and Trenbolone Acetate Extended Release Implant) Feedlot and Grass for Beef Steers and Heifers. Environmental Assessment PNU-0090851, PNU-0023173*

***All Trenbolone references evaluated for the present Case Study are included in* Supplemental Information “Selected literature Trenbolone Case Study.pdf”**

**Table S5-1: Search terms used in the Literature Search for the Trenbolone Case Study**

| **Term target** | **Search term** |
| --- | --- |
| Trenbolone  CAS RN  IUPAC name | *trenbolone  trenbolone ester  trenbolone acetate  trenbolone enanthate  trenbolone cyclohexylmethylcarbonate  parabolan  10161-33-8  17β-Hydroxyestra-4,9,11-trien-3-one |
| General terms | behaviour  behavior  development*  endocrin*  fertil*  gonad*  growth  homeostasis  hormon*  immun*  moult*  neuroendocrin*  pregnan*  pubert*  repro*  spawn*  sperm*  teratogen*  uter*  brain  neuro*  smolt  ano-genital distance  AGD |
| Glands/organs | adrenal  hypothalam*  ovar*  pancreas  parathyroid  pituitary  placenta  testis  testes  testical*  testicul*  thymus  thyroid*  brain  prostate  epidymides |
| Hormone class | (anti-)androgen*  (anti-)estrogen*  (anti-)oestrogen*  androgen*  anti-estrogen*  anti-oestrogen*  aromatase*  estrogen*  glucocorticoid*  iodide  iodothyronine*  thyroid  mineralocorticoid*  oestrogen*  progestagen*  steroid* |
| Vertebrate  hormones | "growth hormone*"  adrenocorticotropic  aldosterone  androstenedione  corticotropin*  cortisol  estradiol  follicle*  glucocorticoid  gonadotrop*  luteinizing*  mineralocorticoid  oxytocin*  vasotocin  mesotosin  corticosterone  parathyroid  progesterone  prolactin*  prostaglandin  testosterone  11-keto-testosterone  thyrotropin*  thyroxine  triiodothyronine*  T3  T4  thyroid |
| Invertebrate hormones | "androgenic hormone"  "diapause hormone"  "ecdysis triggering hormone"  "eclosion hormone"  "egg laying hormone"  "gonad inhibiting hormone"  "gonad stimulating hormone"  "gonad stimulating substance"  "gonadotrophin releasing hormone analogue"  "juvenile hormone"  "mandibular organ inhibiting hormone"  "maturation promoting factor"  "methyl farnesoate"  "molluscan insulin-like peptides"  "moult inhibiting hormone"  "oestrogen like receptor"  "prothoraciotrophic hormone"  "vitellogenesis inhibiting hormone"  "vitellogenesis stimulating ovarian hormone"  adipokinetic*  allatostatin  allatotrophin  APGWamide  bursicon  ecdys*  FMRFamide  neuropeptides  ponasterone  retino*  terpenoid*  RXR |
| Assay types | ER-CALUX  AR-CALUX  Hershberger  MCF-7  ESCREEN  ASCREEN  TSCREEN  GH3  receptor binding  reporter gene  uterotrophic  YAS  yeast  YES  steroidogenic  steroidogenesis  RIA  Radioimmunoassay  ELISA  RBA  Transfection  Luciferase  GAL4  Expression  transactivation |
| Misc | vitellogen*  disorder  disrupt*  imposex  intersex  vas deferens  sex ratio  gender  metamorphosis  mimic  modulat*  ovotest*  steroidogen*  testis-ova  xenoestrogen  condition index  gonadosomatic index  hepatosomatic index  atresia  spermatogonia  sertoli cells  ovulation  secondary sexual characteristics  sexual differentiation  breeding  spawning  nest building  egg laying  spiggin |

**Table S5-2: Environmental water concentrations of Trenbolone reviewed for the Trenbolone Case Study**

**Table S5-3: Active Calls below the Lower Cytotoxicity Limit for 17β-trenbolone in the ToxCast™ Battery**

| **Assay** | **Target/Biological Activity** | **AC50 (µM)** | **% of chemicals evaluated that were active in this assay** |
| --- | --- | --- | --- |
| Tox21_AR_LUC_MDAKB2_Agonist | Androgen Receptor | 1.00E-05 | 2% |
| Tox21_AR_BLA_Agonist_ratio | Androgen Receptor | 0.000318 | 5% |
| OT_AR_ARSRC1_0480 | Androgen Receptor | 0.000374 | 7% |
| NVS_NR_hAR | Androgen Receptor | 0.000409 | 50% |
| OT_AR_ARSRC1_0960 | Androgen Receptor | 0.00145 | 12% |
| NVS_NR_hPR | Progesterone Receptor | 0.005 | 31% |
| NVS_NR_cAR | Androgen Receptor | 0.00605 | 67% |
| NVS_NR_rAR | Androgen Receptor | 0.0149 | 50% |
| ACEA_T47D_80hr_Positive | Estrogen Receptor | 0.0171 | 18% |
| NVS_NR_hER | Estrogen Receptor | 0.046 | 21% |
| ATG_AR_TRANS_up | Androgen Receptor | 0.0549 | 1% |
| NVS_NR_rMR | Mineralocorticoid receptor | 0.0549 | 22% |
| NVS_NR_hGR | Gluccocorticoid receptor | 0.0569 | 67% |
| NVS_NR_bPR | Progesterone Receptor | 0.0722 | 42% |
| Tox21_ERa_LUC_BG1_Agonist | Estrogen Receptor | 0.073 | 15% |
| BSK_hDFCGF_PAI1_up | Serpin peptidase inhibitor, inhibitor of tissue plasminogen activator and urokinase, inhibitor of fibrinolysis, high concentrations of the gene product are associated with thrombophilia | 0.162 | 1% |
| OT_AR_ARELUC_AG_1440 | Androgen Receptor | 0.214 | 8% |
| ATG_ERa_TRANS_up | Estrogen Receptor | 0.468 | 26% |
| ATG_ERE_CIS_up | Estrogen Receptor | 0.644 | 31% |
| Tox21_GR_BLA_Antagonist_ratio | Gluccocorticoid receptor | 1.01 | 8% |
| BSK_CASM3C_MCP1_down | Cytokine, chemotactic activity for monocytes and basophils | 1.29 | 14% |

**Table S5-4: Summary of 17β-trenbolone Results in the Endocrine Disruption Screening Program for the 21st Century**

| **Assay** | **Biological Target** | **Endocrine Pathway** | **AC50 (µM)** | **% of chemicals evaluated that were active in this assay** |
| --- | --- | --- | --- | --- |
| ATG_AR_TRANS_up | AR Transcription factor Activity (human) | Androgen | **0.0549** | 1% |
| NVS_NR_cAR | AR Binding (Chimpanzee) | Androgen | **0.0061** | 67% |
| NVS_NR_hAR | AR Binding (Human) | Androgen | **0.0004** | 50% |
| NVS_NR_rAR | AR Binding (rat) | Androgen | **0.0149** | 50% |
| OT_AR_ARELUC_AG_1440 | Gene Expression via AR and ARE (human receptor, Chinese hamster cell line) | Androgen | **0.214** | 8% |
| OT_AR_ARSRC1_0480 | AR protein dimerization with SRC-1 (human) | Androgen | **0.0004** | 7% |
| OT_AR_ARSRC1_0960 | AR protein dimerization with SRC-1 (human) | Androgen | **0.0015** | 12% |
| Tox21_AR_BLA_Agonist_ratio | Gene Expression via AR and ARE (human) | Androgen | **0.0003** | 5% |
| Tox21_AR_BLA_Antagonist_ratio | Gene Expression via AR and ARE (human) | Androgen | 35.1332 | 21% |
| Tox21_AR_LUC_MDAKB2_Agonist | Gene Expression via AR and ARE (human) | Androgen | **1.00E-05** | 2% |
| Tox21_AR_LUC_MDAKB2_Antagonist | Gene Expression via AR and ARE (human) | Androgen | Inactive | 14% |
| ACEA_T47D_80hr_positive | Proliferation of estrogen-sensitive human breast cells | Estrogen | **0.0171** | 18% |
| ATG_ERE_CIS_up | Gene Expression via ER and ERE (human) | Estrogen | **0.6437** | 31% |
| ATG_ERa_TRANS_up | ESR1 Transcription factor Activity (human) | Estrogen | **0.4683** | 26% |
| NVS_NR_bER | ESR1 Binding (Bovine) | Estrogen | 3.7082 | 14% |
| NVS_NR_hER | ER Binding (Human) | Estrogen | **0.046** | 21% |
| NVS_NR_mERa | ESR1 Binding (mouse) | Estrogen | 4.9756 | 27% |
| OT_ER_ERaERa_0480 | ESR1 protein homodimerization (human) | Estrogen | 30.7851 | 8% |
| OT_ER_ERaERa_1440 | ESR1 protein homodimerization (human) | Estrogen | Inactive | 6% |
| OT_ER_ERaERb_0480 | ESR1 and ESR2 protein heterodimerization (human) | Estrogen | 10.5137 | 12% |
| OT_ER_ERaERb_1440 | ESR1 and ESR2 protein heterodimerization (human) | Estrogen | 5.7885 | 11% |
| OT_ER_ERbERb_0480 | ESR2 protein homodimerization (human) | Estrogen | 13.6771 | 12% |
| OT_ER_ERbERb_1440 | ESR2 protein homodimerization (human) | Estrogen | 5.6691 | 10% |
| OT_ER_EREGFP_0120 | Gene Expression via ESR1 and ERE (human) | Estrogen | 9.3826 | 10% |
| OT_ER_EREGFP_0480 | Gene Expression via ESR1 and ERE (human) | Estrogen | 5.8444 | 8% |
| Tox21_ERa_BLA_Agonist_ratio | Gene Expression via ESR1 and ERE (human) | Estrogen | 2.6042 | 6% |
| Tox21_ERa_BLA_Antagonist_ratio | Gene Expression via ESR1 and ERE (human) | Estrogen Receptor | Inactive | 18% |
| Tox21_ERa_LUC_BG1_Agonist | Gene Expression via ESR1 and ERE (human) | Estrogen | **0.073** | 15% |
| Tox21_ERa_LUC_BG1_Antagonist | Gene Expression via ESR1 and ERE (human) | Estrogen | Inactive | 12% |
| ATG_THRa1_TRANS_up | THRA Transcription factor Activity (human) | Thyroid | Inactive | 3% |
| NVS_NR_hTRa | THRA binding (human) | Thyroid | Not Tested | 25% |
| Tox21_TR_LUC_GH3_Agonist | Regulation of catalytic activity via THRA and THRB and TRE (rat cell line, human receptor) | Thyroid | Inactive | 1% |
| Tox21_TR_LUC_GH3_Antagonist | Regulation of catalytic activity via THRA and THRB and TRE (rat cell line, human receptor) | Thyroid | 48.5264 | 25% |

AR = Androgen Receptor, ER = Estrogen Receptor, ARE = Androgen Response Element, ERE= Estrogen Response Element; ESR1 = Estrogen Receptor 1; ESR2 = Estrogen Receptor 2; THRB = Thyroid Hormone Receptor Beta; THRA = Thyroid Hormone Receptor Alpha; TRE = Thyroid Response Element. Bolded AC50 values were below the lower limit of cytotoxicity for 17β-trenbolone (i.e. 1.52 μM)

**Information on *in vivo* Mechanistic Data (OECD CF Level 3), *in vivo* Population-level Data (OECD CF Level 4) and *in vivo* Multiple Life Stage/ Multiple Generation Population-level Data (OECD CF Level 5) reviewed for the trenbolone case study can be found in Supplemental Information (Trenbolone data summaries.xls).**
