# Supplementary material for: Recommended Approaches to the Scientific Evaluation of Ecotoxicological Hazards and Risks of Endocrine-Active Substances
Source: Integr Environ Assess Manag. Author manuscript; Available in PMC 2018 Aug 1. (PMC6069525; doi:10.1002/ieam.1885)
Supplement: Supplement11 [file NIHMS1500348-supplement-Supplement11.pdf]

# *Selected literature for Trenbolone Case Study*

| Title | Author | Source | Abstract | Cat. | Utility | Keep? |
|---|---|---|---|---|---|---|
| Cross-species conservation of endocrine pathways: a critical analysis of tier 1 fish and rat screening assays with 12 model chemicals | Ankley GT;Gray LE; | Environ Toxicol Chem 2013 | Many structural and functional aspects of the vertebrate hypothalamic-pituitary-gonadal (HPG) axis are known to be highly conserved, but the significance of this from a toxicological perspective has received comparatively little attention. High-quality data generated through development and validation of Tier 1 tests for the U.S. Environmenal Protection Agency Endocrine Disruptor Screening Program (EDSP) offer a unique opportunity to compare responses of mammals versus fish to chemicals that may affect shared pathways within the HPG axis. The present study focuses on data generated with model chemicals that act (primarily) as estrogen receptor agonists (17α-ethynylestradiol, methoxychlor, bisphenol A), androgen receptor agonists (methyltestosterone, 17β-trenbolone), androgen receptor antagonists (flutamide, vincolozolin, p,p'-DDE), or inhibitors of different steroidogenic enzymes (ketoconazole, fadrozole, fenarimol, prochloraz). All 12 chemicals had been tested in the EDSP fish short-term (21 d) reproduction assay and in one or more of the four in vivo Tier 1 screens with rats (uterotrophic, Hershberger, male and female pubertal assays). There was a high concordance between the fish and rat assays with respect to identifying chemicals that impacted specific endocrine pathways of concern. Although most chemicals were detected as positive in both rat and fish assays, eliminating data from one class of vertebrate or the other would weaken the battery. For example, the effects of competitive inhibitors of steroid hormone synthesis were far more obvious in the fish assay, whereas the activity of androgen receptor antagonists was clearer in mammalian assays. The observations are significant both to the cross-species extrapolation of toxicity of HPG-active substances and the optimization of screening and testing frameworks for endocrine-disrupting chemicals | Fish EDC | Limited, uses high dose with trenbolone as a "postive" model for method validation | Yes, species sensitivity |
| Effects of the androgenic growth promoter 17-beta-trenbolone on fecundity and reproductive endocrinology of the fathead minnow | Ankley GT;Jensen KM;Makynen EA;Kahl MD;Korte JJ;Hornung MW;Henry TR;Denny JS;Leino RL;Wilson VS;Cardon MC;Hartig PC;Gray LE; | Environ Toxicol Chem 2003 | Trenbolone acetate is a synthetic steroid that is extensively used in the United States as a growth promoter in beef cattle. The acetate is administered to livestock via slow-release implants; some is converted by the animal to 17-beta-trenbolone, a relatively potent androgen receptor agonist in mammalian systems. Recent studies indicate that excreted 17-beta-trenbolone is comparatively stable in animal waste, suggesting the potential for exposure to aquatic animals via direct discharge, runoff, or both. However, little is known concerning the toxicity of trenbolone to fish. Our goal was to assess the effects of 17-beta-trenbolone on reproductive endocrinology of the fathead minnow (Pimephales promelas). An in vitro competitive binding study with the fathead minnow androgen receptor demonstrated that 17-beta-trenbolone had a higher affinity for the receptor than that of the endogenous ligand, testosterone. Male and female fish were exposed for 21 d to nominal (target) concentrations of 17-beta-trenbolone ranging from 0.005 to 50 microg/L. Fecundity of the fish was significantly reduced by exposure to measured test concentrations > or = 0.027 microg/ L. The 17-beta-trenbolone was clearly androgenic in vivo at these concentrations, as evidenced by the de novo production in females of dorsal (nuptial) tubercles, structures normally present only on the heads of mature males. Plasma steroid (testosterone and beta-estradiol) and vitellogenin concentrations in the females all were significantly reduced by exposure to 17-beta-trenbolone. The 17-beta-trenbolone also altered reproductive physiology of male fathead minnows, albeit at concentrations much higher than those producing effects in females. Males exposed to 17-beta-trenbolone at 41 microg/L (measured) exhibited decreased plasma concentrations of 11-ketotestosterone and increased concentrations of beta-estradiol and vitellogenin. Overall, our studies indicate that 17-beta-trenbolone is a potent androgen and reproductive toxicant in fish. Given the widespread use of trenbolone acetate as a growth promoter, and relative stability of its metabolites in animal wastes, further studies are warranted to assess potential ecological risk | Fish EDC | High | Yes, mechanism and toxicity endpoints |

| | | | | | | |
|---|---|---|---|---|---|---|
| Evaluation of the model anti-androgen flutamide for assessing the mechanistic basis of responses to an androgen in the fathead minnow (Pimephales promelas) | Ankley, GT \| Defoe, DL \| Kahl, MD \| Jensen, KM \| Miracle, A \| Hartig, P \| Gray, LE \| Cardon, M \| Wilson, V | Environ Sci & Techn 38 (23): 6322-6327 DEC 1 2004 | In this study, we characterized the effects of flutamide, a model mammalian androgen receptor (AR) antagonist, on endocrine function in the fathead minnow (Pimephales promelas), a small fish species that is widely used for testing endocrine-disrupting chemicals (EDCs). Binding assays with whole cells transiently transfected with cloned fathead minnow AR indicated that flutamide binds competitively to the receptor. However, as is true in mammalian systems, a 2-hydroxylated metabolite of flutamide binds to the AR with a much higher affinity than the parent chemical. Mixture experiments with flutamide and the androgen 17beta-trenbolone demonstrated that the antiandrogen effectively blocked trenbolone-induced masculinization (nuptial tubercle production) of female fathead minnows, indicating antagonism of an AR receptor-mediated response in vivo. Conversely, reductions in vitellogenin in trenbolone-exposed females were not blocked by flutamide, suggesting that the vitellogenin response is not directly mediated through the AR. The results of these studies provide data demonstrating the validity of using the fathead minnow as a model species for detecting EDCs that exert toxicity through interactions with the AR. | Fish EDC | Limited, uses high dose with trenbolone as a "postive" model for method validation | Yes, mechanistic data on anti-androgen interactions |
| Use of chemical mixtures to differentiate mechanisms of endocrine action in a small fish model | Ankley, GT \| Jensen, KM \| Kahl, MD \| Durhan, EJ \| Makynen, EA \| Cavallin, JE \| Martinovic, D \| Wehmas, LC \| Mueller, ND \| Villeneuve, DL | AQUAT TOXICOL \| 99 (3): 389-396 SEP 1 2010 | Various assays with adult fish have been developed to identify potential endocrine-disrupting chemicals (EDCs) which may cause toxicity via alterations in the hypothalamic-pituitary-gonadal (HPG) axis These assays can be sensitive and highly diagnostic for key mechanisms such as agonism of the estrogen and androgen receptors (ERs, ARs) and inhibition of steroid synthesis However, most of the tests do not unambiguously identify AR antagonists. The purpose of this work was to explore the utility of a mixture test design with the fathead minnow (Pimephales promelas) for detecting different classes of EDCs including AR antagonists Adults of both sexes were exposed via the water to EDCs with diver se mechanisms of action in the absence or presence of 17 beta-trenbolone (TB), a potent AR agonist which masculinizes female fathead minnows. Similar to previous studies with the model AR antagonists flutamide and vinclozolin, exposure of females to the AR antagonist cyproterone acetate in the presence of TB decreased expression of an easily-observed masculinization response, nuptial tubercle formation Mixture studies with TB and the model ER agonists. 17 alpha-ethinylestradiol and bisphenol A. also showed inhibition of tubercle formation in the females, but unlike the AR antagonists, the estrogens markedly induced synthesis of vitellogenin(VTG egg yolk protein), particularly in males The ER agonists also offset TB-induced depressions in plasma VTG concentrations in female fish Additional mixture experiments were conducted with TB and triclocarban, an anti-microbial reported to enhance AR-mediated responses, or ammonia, a ""negative control"" with no known direct effects on HPG function. Neither chemical affected VTG status in males or females in the absence or presence of TB, however, both slightly enhanced TB-induced tubercle formation in females Based on studies described herein and elsewhere with the fathead minnow, a TB co-exposure assay appears to be an effective approach for clearly identifying AR antagonists as well as potential EDCs with other relevant mechanisms of action | Fish EDC | Limited, uses high dose with trenbolone as a "postive" model for method validation | Yes, mechanistic data on anti-androgen interactions |
| Integrated assessment of runoff from livestock farming operations: Analytical chemistry, in vitro bioassays, and in vivo fish exposures | Cavallin JE; Durhan EJ; Evans N; Jensen KM; Kahl MD; Kolpin DW; Kolodziej EP; Foreman WT; Lalone CA; Makynen EA; | Environ Toxicol Chem %2014 , Aug | Animal waste from livestock farming operations can contain varying levels of natural and synthetic androgens and/or estrogens, which can contaminate surrounding waterways. In the present study, surface stream water was collected from 6 basins containing livestock farming operations. Aqueous concentrations of 12 hormones were determined via chemical analyses. Relative androgenic and estrogenic activity was measured using in vitro cell assays (MDA-kb2 and T47D-Kbluc assays, respectively). In parallel, 48-h static-renewal in vivo exposures were conducted to examine potential endocrine-disrupting effects in fathead minnows. Mature fish were exposed to surface water dilutions (0%, 25%, 50%, and 100%) and 10-ng/L of 17α-ethynylestradiol or 50-ng/L of 17β-trenbolone as positive controls. Hepatic expression of vitellogenin and estrogen receptor α mRNA, gonadal ex vivo testosterone and 17β-estradiol production, and plasma vitellogenin concentrations were examined. Potentially estrogenic and androgenic steroids were detected at low nanogram per liter concentrations. In vitro estrogenic activity was | Residu e/Mon itoring Study | Limited, some in vivo fish work and in vitro assays for potency and MoA | Yes |

| | | | | | | |
|---|---|---|---|---|---|---|
| | Seidl SM; Thomas LM; Villeneuve DL; Weberg MA; Wilson VS; Ankley GT; | | detected in all samples, whereas androgenic activity was detected in only 1 sample. In vivo exposures to the surface water had no significant dose-dependent effect on any of the biological endpoints, with the exception of increased male testosterone production in 1 exposure. The present study, which combines analytical chemistry measurements, in vitro bioassays, and in vivo fish exposures, highlights the integrated value and future use of a combination of techniques to obtain a comprehensive characterization of an environmental chemical mixture | | | |
| Impacts of an Anti-Androgen and an Androgen/Anti-Androgen Mixture on the Metabolite Profile of Male Fathead Minnow Urine | Collette, TW \| Teng, Q \| Jensen, KM \| Kahl, MD \| Makynen, EA \| Durhan, EJ \| Villeneuve, DL \| Martinovic-Weigelt, D \| Ankley, GT \| Ekman, DR | Environ Sci & Techn \| 44 (17): 6881-6886 SEP 1 2010 | Male and female fathead minnows (Pimephales promelas, FHM) were exposed via water to 20 or 200 mu g/L of cyproterone acetate (CA), a model androgen receptor (AR) antagonist FHM were also exposed to 500 ng/L of 17 beta-trenbolone (TB), a model AR agonist, and to mixtures of TB with both concentrations of CA. The urine metabolite profile (as measured by (1)H NMR spectroscopy) of male FHM exposed to the high concentration of CA was markedly different from that of controls, and this difference was less for males coexposed to the associated TB+CA mixture. The exposure to TB alone had almost no impact on the male urine profile. These results suggest that male FHM urinary metabolite profiling may be useful for directly detecting effects of anti-androgens. In contrast, the urinary profile of male FHM exposed to the lower concentration of CA was not very different from that of controls, but, unexpectedly, this difference was increased when coexposed to the associated TB+CA mixture. This suggests that TB with CA at the lower concentration impacts male FHM through an interactive effect possibly unrelated, or in addition, to AR antagonism. The relative occurrence of male-like nuptial tubercles in female FHM exposed to TB and to the mixtures of TB and CA supported the metabolomics data. | Fish EDC | Limited, uses high dose with trenbolone as a "postive" model for method validation | Yes, mechanistic data on anti-androgen interactions |
| Metabolite profiling and a transcriptional activation assay provide direct evidence of androgen receptor antagonism by bisphenol A in fish | Ekman DR;Hartig PC;Cardon M;Skelton DM;Teng Q;Durhan EJ;Jensen KM;Kahl MD;Villeneuve DL;Gray LE;Collette TW;Ankley GT; | Environ Sci Technol 2012 | Widespread environmental contamination by bisphenol A (BPA) has created the need to fully define its potential toxic mechanisms of action (MOA) to properly assess human health and ecological risks from exposure. Although long recognized as an estrogen receptor (ER) agonist, some data suggest that BPA may also behave as an androgen receptor (AR) antagonist. However, direct evidence of this activity is deficient. To address this knowledge gap, we employed a metabolomic approach using in vivo exposures of fathead minnows (FHM; Pimephales promelas ) to BPA either alone or in a binary mixture with 17β-trenbolone (TB), a strong AR agonist. Changes in liver metabolite profiles in female FHM in response to these exposures were determined using high resolution (1)H NMR spectroscopy and multivariate and univariate statistics. Using this approach, we observed clear evidence of the ability of BPA to mitigate the impact of TB, consistent with an antiandrogenic MOA. In addition, a transcriptional activation assay with the FHM AR was used to confirm the AR antagonistic activity of BPA in vitro. The results of these in vivo and in vitro analyses provide strong and direct evidence for ascribing an antiandrogenic MOA to BPA in vertebrates | Fish EDC | Limited, uses high dose with trenbolone as a "postive" model for method validation | Yes, mechanistic interaction with anti-androgens |
| Use of gene expression, biochemical and metabolite profiles to enhance exposure and effect assessment of the model | Ekman, DR \| Villeneuve, DL \| Teng, Q \| Ralston-Hooper, KJ \| Martinovic-Weigelt, D \| Kahl, MD \| Jensen, KM \| Durhan, EJ \| | ENVIRONMENTAL TOXICOLOGY AND CHEMISTRY \| 30 (2 319-329. 2010 | The impact of exposure by water to a model androgen, 17 beta-trenbolone (TRB), was assessed in fathead minnows using an integrated molecular approach. This included classical measures of endocrine exposure such as impacts on testosterone (T), 17 beta-estradiol (E2), and vitellogenin (VTG) concentrations in plasma, as well as determination of effects on the hepatic metabolome using proton nuclear magnetic resonance spectroscopy. In addition, the rates of production of T and E2 in ovary explants were measured, as were changes in a number of ovarian gene transcripts hypothesized to be relevant to androgen exposure. A temporally intensive 16-d test design was used to assess responses both during and after the TRB exposure (i.e., depuration/recovery). This strategy revealed time-dependent responses in females (little impact was seen in the males), in which changes in T and E2 production in the ovary, as well as levels in plasma, declined rapidly (within 1 d), followed shortly by a return to control levels. Gene expression measurements revealed dynamic control of transcript levels in the ovary and suggested potential | Fish EDC | Limited, mostly mechanistic data | Yes |

| | | | | | | |
|---|---|---|---|---|---|---|
| androgen 17 beta-trenbolone in fish | Makynen, EA \| Ankley, GT \| Collette, TW | | mechanisms for compensation during the exposure phase of the test. Proton nuclear magnetic resonance spectroscopy revealed a number of hepatic metabolite changes that exhibited strong time and dose dependence. Furthermore. TRB appeared to induce the hepatic metabolome of females to become more like that of males at both high test concentrations of TRB (472 ng/L) and more environmentally relevant levels (33 ng/L). | | | |
| Expression Signatures for a Model Androgen and Antiandrogen in the Fathead Minnow (Pimephales promelas) Ovary | Garcia-Reyero, N \| Villeneuve, DL \| Kroll, KJ \| Liu, L \| Orlando, EF \| Watanabe, KH \| Sepulveda, MS \| Ankley, GT \| Denslow, ND | Environ Sci & Techn 43 (7): 2614-2619 APR 1 2009 | Trenbolone, an anabolic androgen, and flutamide, an antiandrogen, are prototypical model compounds for agonism and antagonism of the androgen receptor. We hypothesized that 48 h exposures of female fathead minnows (Pimephales promelas) to environmentally relevant concentrations of these chemicals would alter genes regulated by the androgen receptor and that a mixture of the two compounds would block the effects. Gene expression in the ovaries was analyzed using a fathead minnow-specific 22 000-gene microarray. Flutamide altered about twice the number of genes as trenbolone, most of which appeared to be through pathways not associated with the androgen receptor. A group of 70 genes, of which we could identify 37, were reciprocally regulated by trenbolone and flutamide. These are candidates for specific biomarkers for androgen receptor mediated gene expression. Four genes stand out as specifically related to reproduction: sperm associated antigen 8 (SPAG8), CASP8 and FADD-like apoptosis regulator (CFLAR), corticotropin releasing hormone (CRH), and 3 beta-hydroxysteroid dehydrogenases (3 beta-HSD). Three notable transcriptional regulators including myelocytomatosis viral oncogene homologue (MYC), Yin Yang 1 (YY1), and interferon regulator factor 1 (IRF1) may function as early molecular switches to control phenotypic changes in ovary tissue architecture and function in response to androgen or antiandrogen exposure. | Fish EDC | Limited, gene expression only | Yes, mechanistic study on interactinos with anti-androgens |
| Effects of the feedlot contaminant 17alpha-trenbolone on reproductive endocrinology of the fathead minnow | Jensen KM;Makynen EA;Kahl MD;Ankley GT; | Environ Sci & Techn 40 (9): 3112-3117 MAY 1 2006 | Trenbolone acetate is a growth promoter widely used for beef production in the U.S. Two biologically active metabolites of the acetate, 17beta- and 17alpha-trenbolone, are ligands of vertebrate androgen receptors and comparatively stable in the waste of treated animals. Both have been detected in surface water associated with beef feedlots, suggesting a potential risk to aquatic animals. In previous work we evaluated the effects of beta-trenbolone on reproductive endocrinology of the fathead minnow (Pimephales promelas) in a 21-day test. The purpose of the present study was to conduct a similar set of experiments with alpha-trenbolone which, based on binding to mammalian androgen receptors, was expected to be less potent than beta-trenbolone. Fecundity of the fish was significantly reduced by alpha-trenbolone with an EC50 (95% confidence interval) of 0.011 (0.007-0.016) microg/L. In females, alpha-trenbolone reduced plasma vitellogenin and steroid concentrations and also induced the production of dorsal nuptial tubercles, structures normally present only in spawning males. Overall, effects of alpha-trenbolone on the reproductive system of the fish were qualitatively and quantitatively quite similar to those caused by beta-trenbolone. Part of this similarity might arise from the fact that a substantial amount of the alpha-trenbolone appeared to be converted to beta-trenbolone by the fish. Tissue concentrations of the beta-isomer were consistently similar to or greater than concentrations of alpha-trenbolone, despite the fact that no beta-trenbolone was detected in the exposure water. The present study demonstrates the importance of considering both alpha- and beta-trenbolone in assessing the potential ecological risk of androgens associated with beef feedlot discharges | Fish EDC | High | Yes, mechanistic and apical endpoints |

| | | | | | | |
|---|---|---|---|---|---|---|
| Evaluation of a commercial kit for measuring vitellogenin in the fathead minnow (Pimephales promelas) | Jensen, KM \| Ankley, GT | ECOTOX & ENVIRON SAFETY \| 64 (2): 101-105 2006 | Vitellogenin (vtg) concentrations in oviparous animals such as fish represent an integrated indicator of the status of the reproductive endocrine system. As such, vtg is a common measurement endpoint in tests designed to detect certain classes of endocrine-disrupting chemicals (EDCs). The most common approach to measuring vtg is via enzyme-linked immunosorbent assays (ELISAs). However, because labs testing EDCs in fish often use slightly different ELISAs (e.g., in terms of antibodies, binding antigens, standards), results among studies are not always comparable. One approach to obviating this would be for researchers to use standardized ELISA kits from a common source(s). The fathead minnow (Pimephales promelas) is a small fish model commonly used for EDC testing. The purpose of this study was to evaluate a recently developed commercial ELISA kit for measuring vtg in the fathead minnow. The commercial ELISA, based on a monoclonal antibody to fathead minnow vtg, was compared to an ELISA that utilizes a fathead minnow polyclonal antibody, which has been used extensively in our lab and others for several years. Plasma samples for this comparison came from three studies in which fathead minnows had been exposed to different model EDCs, including an androgen (17 beta-trenbolone), an anti-androgen (flutamide), and two CYP19 (aromatase) inhibitors (prochloraz, fadrozole). Results obtained using the two different ELISA methods were consistently similar. | Fish EDC | Limited, uses high dose with trenbolone as a "postive" model for method validation. VTG response for trenbolone (summarized elsewhere?) | Yes |
| Molecular target sequence similarity as a basis for species extrapolation to assess the ecological risk of chemicals with known modes of action | Lalone CA;Villeneuve DL;Burgoon LD;Russom CL;Helgen HW;Berninger JP;Tietge JE;Severson MN;Cavallin JE;Ankley GT; | Aquat Toxicol 2013 , | It is not feasible to conduct toxicity tests with all species that may be impacted by chemical exposures. Therefore, cross-species extrapolation is fundamental to environmental risk assessment. Recognition of the impracticality of generating empirical, whole organism, toxicity data for the extensive universe of chemicals in commerce has been an impetus driving the field of predictive toxicology. We describe a strategy that leverages expanding databases of molecular sequence information together with identification of specific molecular chemical targets whose perturbation can lead to adverse outcomes to support predictive species extrapolation. This approach can be used to predict which species may be more (or less) susceptible to effects following exposure to chemicals with known modes of action (e.g., pharmaceuticals, pesticides). Primary amino acid sequence alignments are combined with more detailed analyses of conserved functional domains to derive the predictions. This methodology employs bioinformatic approaches to automate, collate, and calculate quantitative metrics associated with cross-species sequence similarity of key molecular initiating events (MIEs). Case examples focused on the actions of (a) 17α-ethinyl estradiol on the human (Homo sapiens) estrogen receptor; (b) permethrin on the mosquito (Aedes aegypti) voltage-gated para-like sodium channel; and (c) 17β-trenbolone on the bovine (Bos taurus) androgen receptor are presented to demonstrate the potential predictive utility of this species extrapolation strategy. The examples compare empirical toxicity data to cross-species predictions of intrinsic susceptibility based on analyses of sequence similarity relevant to the MIEs of defined adverse outcome pathways. Through further refinement, and definition of appropriate domains of applicability, we envision practical and routine utility for the molecular target similarity-based predictive method in chemical risk assessment, particularly where testing resources are limited | Fish EDC | High | Yes, species sensitivity predictions (including inverts) |
| Environmental hormones and their impacts on sex differentiation in fathead minnows | Leet JK;Sassman S;Amberg JJ;Olmstead AW;Lee LS;Ankley GT;Sep£lveda MS; | Aquat Toxicol %2015 , Jan | Runoff from lands fertilized with animal manure from concentrated animal feeding operations (CAFOs) is a source of hormones to surface water. In this study we tested the hypothesis that larval fathead minnows exposed to sex steroids singly or in a 'typical' CAFO mixture during sex differentiation would respond with changes in the expression of a set of target genes, leading to gonadal abnormalities later in life. In the first experiment, a static daily-renewal system was used to expose larvae during the period of 10-20 days post-hatch (dph) to either 5 ng/L 17β-trenbolone (17β-TRB) or 5 ng/L 17α-ethinylestradiol (EE2). In a second experiment, fish were exposed from 0 to 45 dph in a flow-through system to a CAFO mixture composed of steroids and degradates (2-16 ng/L), atrazine and degradates (15-250 ng/L), and nitrate (3-11 mg/L). In the single hormone experiment, expression of genes involved in steroidogenesis (cyp19a, cyp17, and star) was decreased in females. In contrast, no differences in gene expression were observed in fish exposed to the CAFO mixture. However, the majority (84%) of treated males | Fish EDC | Limited, few dose, poorly defined mixtures, some Trenbolone exposure? | Yes |

| | | | | | | |
|---|---|---|---|---|---|---|
| | | | had testes containing an ovarian cavity, indicative of feminization, compared to 0% in the control males. Overall, our results show that: (1) changes in gene expression after single hormone exposures are sex-specific, with females more responsive than males; and (2) phenotypic alterations in testicular development can be elicited by a simulated 'CAFO' mixture when fathead minnows are exposed during the first 45 days of development. More research is needed to further discern the complex response of fish to steroid mixtures, especially those associated with runoff from land-applied CAFO waste | | | |
| A computational model of the hypothalamic: pituitary: gonadal axis in female fathead minnows (Pimephales promelas) exposed to 17α-ethynylestradiol and 17β-trenbolone | Li Z;Kroll KJ;Jensen KM;Villeneuve DL;Ankley GT;Brian JV;Sep£lveda MS;Orlando EF;Lazorchak JM;Kostich M;Armstrong B;Denslow ND;Watanabe KH; | BMC Syst Biol %2011 | BACKGROUND: Endocrine disrupting chemicals (e.g., estrogens, androgens and their mimics) are known to affect reproduction in fish. 17 alpha-ethynylestradiol is a synthetic estrogen used in birth control pills. 17 beta-trenbolone is a relatively stable metabolite of trenbolone acetate, a synthetic androgen used as a growth promoter in livestock. Both 17 alpha-ethynylestradiol and 17 beta-trenbolone have been found in the aquatic environment and affect fish reproduction. In this study, we developed a physiologically-based computational model for female fathead minnows (FHM, Pimephales promelas), a small fish species used in ecotoxicology, to simulate how estrogens (i.e., 17 alpha-ethynylestradiol) or androgens (i.e., 17 beta-trenbolone) affect reproductive endpoints such as plasma concentrations of steroid hormones (e.g., 17 beta-estradiol and testosterone) and vitellogenin (a precursor to egg yolk proteins). RESULTS: Using Markov Chain Monte Carlo simulations, the model was calibrated with data from unexposed, 17 alpha-ethynylestradiol-exposed, and 17 beta-trenbolone-exposed FHMs. Four Markov chains were simulated, and the chains for each calibrated model parameter (26 in total) converged within 20,000 iterations. With the converged parameter values, we evaluated the model's predictive ability by simulating a variety of independent experimental data. The model predictions agreed with the experimental data well. CONCLUSIONS: The physiologically-based computational model represents the hypothalamic-pituitary-gonadal axis in adult female FHM robustly. The model is useful to estimate how estrogens (e.g., 17 alpha-ethynylestradiol) or androgens (e.g., 17 beta-trenbolone) affect plasma concentrations of 17 beta-estradiol, testosterone and vitellogenin, which are important determinants of fecundity in fish | Fish EDC | Limited, mechansitic modeling based on published toxicity data | Yes |
| A computational model for asynchronous oocyte growth dynamics in a batch-spawning fish | Li, ZH \| Villeneuve, DL \| Jensen, KM \| Ankley, GT \| Watanabe, KH | CAN JOURN FISH AND AQUAT SCI 68 (9): 1528-1538 2011 | A computational model of oocyte growth dynamics (i.e., oocyte recruitment, growth, and spawning) in a batch-spawning fish, fathead minnow (FHM, Pimephales promelas), has been developed. The model provides a quantitative link between oocyte growth dynamics and biochemical processes in FHMs through the absorption of vitellogenin (a lipoprotein precursor of egg yolk proteins) into oocytes, which contributes significantly to oocyte growth in fish. The model simulates the number and volume of oocytes in different batches within a FHM ovary. Model-predicted clutch sizes and spawning intervals matched the experimental data well for both unexposed FHMs and FHMs exposed to 17 beta-trenbolone (a relatively stable metabolite of trenbolone acetate, a synthetic androgen used as a growth promoter in livestock). Overall, the model presents a novel approach to simulating oocyte growth dynamics in a batch-spawning fish and meets an urgent need in eco-toxicological studies to link the effects of endocrine disrupting chemicals at a biochemical level to adverse effects upon reproduction. | Fish EDC | Limited, mechansitic modeling based on published toxicity data | Yes |
| Reproductive toxicity of vinclozolin in the fathead minnow: confirming an anti-androgenic mode of action | MartinovićBlake LS;Durhan EJ;Greene KJ;Kahl MD;Jensen KM;Makynen EA;Villeneuve | Environ Toxicol Chem %2008 , Feb | The objective of the present study was to characterize responses of the reproductive endocrine system of the fathead minnow (Pimephales promelas) to the fungicide vinclozolin (VZ), using a 21-d reproduction assay, and a shorter-term (approximately two weeks) test in which fish were cotreated with the VZ (a putative anti-androgen) and the androgen 17beta-trenbolone (TB). Effects on fecundity, gonadal histology, secondary sexual characteristics, reproductive hormones, and relative abundance of androgen receptor (AR) and 11beta-hydroxysteroid dehydrogenase (11betaHSD) mRNA transcripts were evaluated in one or both of these studies. Fecundity of VZ-exposed fish was decreased in a concentration-dependent manner in the 21-d test, culminating in complete reproductive failure at a concentration of 700 microg/L. Exposure to VZ decreased expression of male secondary | Fish EDC | Limited, mixture study with anti-androgen | Yes, chemical interactions |

| | | | | | | |
|---|---|---|---|---|---|---|
| | DL;Ankley GT; | | sexual characteristics -- an effect typical of anti-androgens. The finding that exposure of females to TB-induced expression of prominent, male-like tubercles, which could be effectively blocked with VZ, provides powerful evidence of the anti-androgenic activity of VZ in vivo. In the two experiments VZ produced several responses possibly indicative of compensation or adaptation of the fish to the anti-androgen, including increases in gonad weight, AR and 11 betaHSD mRNA transcript abundance, and ex vivo gonadal production of testosterone and 11-ketotestosterone. Overall, our results demonstrate that the model anti-androgen VZ, which also is an environmental contaminant, impairs reproductive success of fathead minnows and elicits endocrine responses consistent with an anti-androgenic mode of action | | | |
| Quantitative Proteomic Profiles of Androgen Receptor Signaling in the Liver of Fathead Minnows (Pimephales promelas) | Martyniuk, CJ \| Alvarez, S \| McClung, S \| Villeneuve, DL \| Ankley, GT \| Denslow, ND | JOURNAL OF PROTEOME RESEARCH \| 8 (5): 2186-2200 MAY 2009 | Androgenic chemicals are present in the environment at concentrations that impair reproductive processes in fish. The objective of this experiment was to identify proteins and cell processes mediated through androgen receptor signaling using an androgen receptor agonist (17 beta-trenbolone) and antagonist (flutamide) in the liver. Female fathead minnows were exposed to nominal concentrations of either 17 beta-trenbolone (0.05, 0.5, or 5 mu g/L), flutamide (50, 150, or 500 mu g/L), or a mixture (500 mu g flutamide/L and 0.5 mu g 17 beta-trenbolone/L) for 48 h. The iTRAQ method was used to label peptides after protein extraction and trypsin-digestion from livers of untreated controls or from fish treated with 17 beta-trenbolone (5 mu g/L), flutamicle (500 mu g/L), or a mixture of both compounds. Forty-five proteins were differentially altered by one or more treatments ($p < 0.05$). Many altered proteins were involved in cellular metabolism (e.g., glyceraldehyde 3-phosphate dehydrogenase, phosphoglycerate mutase), general and oxidative stress response (e.g., superoxide dismutase and heat shock proteins), and the regulation of translation (e.g., ribosomal proteins). Cellular pathway analysis identified additional signaling cascades activated or inhibited by flutamide that may not be androgen receptor mediated. We also compared changes in select proteins to changes in their mRNA levels and observed, in general, that proteins and mRNA changes did not correlate, suggesting complex regulation at the level of both the transcriptome and proteome. It is concluded that both transcriptomic and proteomic approaches offer unique and complementary insights into mechanisms of regulation. We demonstrate the utility of proteomic profiling for use on a model species with value to ecotoxicology but having limited genomic information. | Fish EDC | Limited, only protein expression | Yes, background |
| Modeling impacts on populations: fathead minnow (Pimephales promelas) exposure to the endocrine disruptor 17 beta-trenbolone as a case study | Miller, DH \| Ankley, GT | ECOTOX & ENVIRON SAFETY \| 59 (1): 1-9 SEP 2004 | Evaluation of population-level impacts is critical to credible ecological risk assessments. In this study, a predictive model was developed to translate changes in fecundity of the fathead minnow (Pimephales promelas) in a short-term laboratory toxicity test to alterations in population growth rate. The model uniquely combines a Leslie population projection matrix and the logistic equation. Application of the model requires only a life table for the organism of interest, a measure of carrying capacity for the given population. and an estimation of the effect of a stressor on vital rates. The model was applied to investigate population dynamics for fathead minnow exposed to the androgen receptor agonist 17beta-trenbolone. Organismal-level responses for fathead minnows exposed to varying levels of 17beta-trenbolone were used to determine projected alterations in a population existing in a small body of water containing varying concentrations of the androgen. Fathead minnow populations occurring at carrying capacity and subsequently exposed to 0.027 mug/L of 17beta-trenbolone exhibited a 51% projected decrease in average population size after 2 years of exposure. Populations at carrying capacity exposed to concentrations of 17beta-trenbolone greater than or equal to0.266 mug/L exhibited a 93% projected decrease in average population size after 2 years of exposure. Overall, fathead minnow populations exposed to continued concentrations of 17beta-trenbolone equal to or greater than 0.027 mug/L were projected to have average equilibrium population sizes that approached zero. | Fish EDC | High, population responses | Yes |

| | | | | | | |
|---|---|---|---|---|---|---|
| Linkage of biochemical responses to population-level effects: A case study with vitellogenin in the fathead minnow (Pimephales promelas) | Miller, DH \| Jensen, KM \| Villeneuve, DL \| Kahl, MD \| Makynen, EA \| Durhan, EJ \| Ankley, GT | ENVIRONMENTAL TOXICOLOGY AND CHEMISTRY \| 26 (3): 521-527 MAR 2007 | A challenge in the field of ecotoxicology is the linkage of alterations at molecular and biochemical levels of organization to adverse outcomes in individuals and populations. In the present study, a predictive relationship between plasma vitellogenin (VTG) concentration and fecundity in female fathead minnows (Pimephales promelas) was derived from 21-d laboratory toxicity tests with five chemicals (17 beta-trenbolone, 17 alpha-trenbolone, prochloraz, fenarimol, and fadrozole) that inhibit VTG production through different mechanisms. Because VTG is key to egg production in female oviparous animals, changes in the lipoprotein could, theoretically, serve as an indicator of reproductive success. Regression of fecundity versus VTG concentration from the various studies yielded a highly significant linear model (fecundity = -0.042 + 0.95-VTG, $p < 0.01$, r(2) = 0.88). This relationship was integrated into a population model to translate changes in VTG concentrations of female fathead minnows to alterations in population growth. The model predicted relatively profound effects on population size of fish experiencing moderate decreases in vitellogenesis. For example, a fathead minnow population at a carrying capacity exposed to a chemical stressor that causes a 25% decrease in VTG concentration in females from baseline values would exhibit a 34.6% projected decrease in size after two years of exposure and reach an equilibrium population size that was only 30.2% of the preexposed population. Overall, the current study provides an example of how changes in a biomarker (VTG concentration) can be quantitatively translated into adverse effects at the individual and population levels. | Fish EDC | High, population responses | Yes |
| Expression of two vitellogenin genes (vg1 and vg3) in fathead minnow (Pimephales promelas) liver in response to exposure to steroidal estrogens and androgens | Miracle, A \| Ankley, G \| Lattier, D | ECOTOX & ENVIRON SAFETY \| 63 (3): 337-342 MAR 2006 | In this study, we describe the sequence for the fathead minnow (Pimephalts promelas) vitellogenin 3 gene (vg3) and compare the responses of vg1 and vg3 following exposure to steroidal estrogens and androgens. The fathead minnow vg3 sequence is:the second nucleotide sequence described in teleosts, following the original description of this isoform in zebrafish (Danio rerio). Following a brief water exposure (24 h) to 2, 5, and 10 ng/L 17 alpha-ethynylestradiol (EE2), both vg1 and vg3 were upregulated in male liver. However, levels of vg3 induction were four orders of magnitude lower than levels of induction of vy1. Suppression of vg in female liver following exposure to 50 or 500 ng/L of the synthetic androgen 17 beta-trenbolone occurs at similar magnitude for both vg1 and vg3 isoforms. The results of this study Support the use of vg1 as an indicator of estrogenic exposure in male fish and indicate the potential for vg1 and/or vg3 for use as indicators of androgenic exposure. | Fish EDC | Limited, high doses | Yes |
| Structural and functional diversity of microbial communities from a lake sediment contaminated with trenbolone, an endocrine-disrupting chemical | Radl, V \| Pritsch, K \| Munch, JC \| Schloter, M | ENVIRONMENTAL POLLUTION \| 137 (2): 345-353 SEP 2005 | Effects of trenbolone (TBOH), a hormone used in cattle production, on the structure and function of microbial communities in a fresh water sediment from a lake in Southern Germany were studied in a microcosm experiment. The microbial community structure and the total gene pool of the sediment, assessed by 16S rRNA/rDNA and RAPD fingerprint analysis, respectively, were not significantly affected by TBOH. In contrast, the N-acetyl-glucosaminidase activity was almost 50% lower in TBOH treated samples ($P<0.05$). Also, the substrate utilization potential, measured using the BIOLOG(R) system, was reduced after TBOH treatment. Interestingly, this potential did not recover at the end of the experiment, i.e. 19 days after the addition of the chemical. Repeated application of TBOH did not lead to an additional reduction in the substrate utilization potential. Overall results indicate that microbial community function was more sensitive to TBOH treatment than the community structure and the total gene pool. | Microbial toxicity | Limited | Yes, includes microbial effects of trenbolone |

| | | | | | | |
|---|---|---|---|---|---|---|
| Improvements in body composition, cardiometabolic risk factors and insulin sensitivity with trenbolone in normogonadic rats | Donner, DG \| Beck, BR \| Bulmer, AC \| Lam, AK \| Du Toita, EF | STEROIDS \| 94: 60-69 FEB 2015 | Trenbolone (TREN) is used for anabolic growth-promotion in over 20 million cattle annually and continues to be misused for aesthetic purposes in humans. The current study investigated TREN's effects on body composition and cardiometabolic risk factors; and its tissue-selective effects on the cardiovascular system, liver and prostate. Male rats (n = 12) were implanted with osmotic infusion pumps delivering either cyclodextrin vehicle (CTRL) or 2 mg/kg/day TREN for 6 weeks. Dual-energy X-ray Absorptiometry assessment of body composition; organ wet weights and serum lipid profiles; and insulin sensitivity were assessed. Cardiac ultrasound examinations were performed before in vivo studies assessed myocardial susceptibility to ischemia reperfusion (I/R) injury. Circulating sex hormones and liver enzyme activities; and prostate and liver histology were examined. In 6 weeks, fat mass increased by 34 +/- 7% in CTRLs (p < 0.01). Fat mass decreased by 37 +/- 6% and lean mass increased by 11 +/- 4% with TREN (p < 0.05). Serum triglycerides, HDL and LDL were reduced by 62%, 57% and 78% (p < 0.05) respectively in TREN rats. Histological examination of the prostates from TREN-treated rats indicated benign hyperplasia associated with an increased prostate mass (149% compared to CTRLs, p < 0.01). No evidence of adverse cardiac or hepatic effects was observed. In conclusion, improvements in body composition, lipid profile and insulin sensitivity (key risk factors for cardiometabolic disease) were achieved with six-week TREN treatment without evidence of adverse cardiovascular or hepatic effects that are commonly associated with traditional anabolic steroid misuse. Sex hormone suppression and benign prostate hyperplasia were confirmed as adverse effects of the treatment. | Livestock Efficacy Study | Mammalian EDC MoA ? | Yes |
| Evaluation of the rodent Hershberger bioassay: Testing of coded chemicals and supplementary molecular-biological and biochemical investigations | Freyberger, A \| Ellinger-Ziegelbauer, H \| Krotlinger, F | TOXICOLOGY \| 239 (1-2): 77-88 SEP 24 2007 | Under the auspices of the Organization for Economic Cooperation and Development (OECD) the Hershberger assay is being validated as an in vivo screen for compounds with (anti)androgenic potential. We participated in the final activity, the testing of coded chemicals. Test compounds included trenbolone (TREN; 1.5, 40 mg/kg), testosterone propionate (TP; 0.4 mg/kg), flutamide (FLUT; 3 mg/kg), linuron (LIN; 10, 100 mg/kg), 1, 1 -bis-(4-chlorophenyl)-2,2-dichloroethylene (p,p'-DDE; 16, 160 mg/kg), and two negative reference substances, i.e., compounds not considered to affect androgen-sensitive tissue weights (ASTWs) in the Hershberger assay, namely 4-nonylphenol (NP; 160 mg/kg) and 2,4-dinitrophenol (DNP; 10 mg/kg); TREN, LIN, p,p'-DDE, NP, and DNP being used under code. Compounds were administered for 10 days by oral intubation or subcutaneous injection (TP). Additional investigations not mandatorily requested by OECD included organ gravimetry of the liver, gene expression analysis in prostate using quantitative RT PCR for prostate specific binding protein polypeptide C3 (PBPC3) and ornithine decarboxylase 1 (ODC1) and determination of testosterone metabolizing and phase 11 conjugating enzymes in the liver. After submission of all study reports to OECD by participants uncoding revealed the following results: (A) When assessing androgenic potential in castrated rats, administration of TREN increased the weights of ventral prostate (VP), seminal vesicles (SV), glans penis, levator ani and bulbocavernosus muscles, and Cowper's glands at the high dose. A similar or stronger (VP, SV) increase of ASTWs was observed for TP; NP and DNP were ineffective. TREN dose-dependently increased gene expression of ODC1 and PBPC3, TP induced expression of these genes even more strongly (almost) to the level of untreated intact animals, whereas NP and DNP were inactive. Liver enzyme activities depending on physiological androgen levels were lower in castrated than in intact rats and could not be restored by androgen treatment. (B) When assessing antiandrogenic potential in TP-supplemented castrated rats, administration of LIN and p,p '-DDE decreased ASTWs only at the high dose. FLUT even more effectively decreased ASTWs, NP and DNP were again without effect. Decreases in androgen-responsive gene expression in the prostate corresponding to the organ weight changes were only observed for p,p'-DDE (high dose) and flutamide (PBPC3 only). p,p '-DDE dose-dependently induced liver weights and most liver enzyme activities including androgen-dependent ones. Our study accurately reproduced ASTW changes obtained in previous studies also under code suggesting that the Hershberger assay is a robust tool to screen for an (anti)androgenic potential. Assessment of ODC1 and PBPC3 gene expression in | Mammalian EDC Study | Limited, uses high dose with trenbolone as a "postive" model for method validation, Mammalian EDC, Level 3 | Yes |

| | | | | | | |
|---|---|---|---|---|---|---|
| | | | prostate, however, may only represent a sensitive tool for the detection of an androgenic potential. Finally, p,p '-DDE may affect ASTWs by several mechanisms including enhanced testosterone metabolism. | | | |
| Prenatal programming of mammalian growth - a review of the role of steroids | Gill, JW \| Hosking, BJ \| Egan, AR | LIVESTOCK PROD SCI 54 (3): 251-267 1998 | The steroid milieu of differentiating mammals influences aspects of growth and development. Changes to the steroid milieu of a differentiating mammal results in physiological changes to that animal. These changes can be permanent and identified as phenomena measured in later life. During prenatal development, steroids programme the timing of critical events such as birth, puberty and death. They also influence body shape and composition, growth rates, physiology, placental morphology, behaviour and sexual differentiation. Prenatal steroids programme livestock and their manipulation can result in enhanced growth characters in domestic livestock. | Mammalian EDC Study | Limited, review | Yes, background |
| Effect of 17 beta-trenbolone on male and female reproduction in Japanese quail (Coturnix japonica) | Henry, PFP \| Akuffo, VG \| Chen, Y \| Karouna-Renier, NK \| Sprague, DT \| Bakst, MR | AVIAN BIOLOGY RESEARCH \| 5 (2): 61-68 2012 | The anabolic steroid 17 beta trenbolone (17 beta-TB), a known endocrine disrupting chemical, may influence reproductive functions in avian wildlife. We evaluated the effects of dietary exposure to 17 beta-TB at 5 and 20 ppm on reproductive functional endpoints in Japanese quail during and after sexual maturation. In the male, 5 and 20 ppm treatments revealed no differences in body and testes weight, testes histology, plasma testosterone concentrations, or size and weight of the foam glands. However, the onset of foam production was significantly earlier (days of age) in the 20 ppm males. In females, dietary 17 beta-TB at 20 ppm caused a reduction in the number of maturing yellow yolk follicles and overall egg production. Plasma testosterone concentrations were reduced compared to controls. Histology of the oviductal sperm storage tubules was normal in all treatments. The number of sperm holes, sites on the perivitelline layer (PVL) where sperm bound and hydrolyzed a path through the PVL, was significantly greater in the 10th egg laid compared to the 1st egg laid in the 20 ppm treatment. Potential effects, albeit transient, on endpoints associated with male maturation warrant further investigation into the sensitivity of these measures in the event of embryonic and/or trans-generational exposure to 17 beta-TB. | Avian EDC Study | High | Yes |
| In Utero Exposure To An Environmental Androgen, Trenbolone, Masculinizes Female Rats | Hotchkiss A;Furr J;Makynen E;Ankley G;Gray E; | | Recently, in vitro studies have detected androgenic activity in pulp mill effluents and cattle feedlot operations. Trenbolone acetate is one of the anabolic steroids used as a growth promoter in cattle. The primary active metabolites of trenbolone acetate, 17alpha-trenbolone and 17beta-trenbolone (TB), are potent androgens, both of which have been detected in solid and liquid waste from cattle. Laboratory studies in rats have shown that TB, similar to other C-19 norandrogens, causes tissue-selective anabolic and androgenic effects in castrate males, potentially due to the inability of 5-alpha reduction to enhance the potency of TB. We present the results of studies characterizing the permanent reproductive/developmental effects of prenatal trenbolone exposure on female rat offspring. In addition, we compare the potency of developmental exposure to TB to testosterone propionate (TP) for a number of different reproductive endpoints. Briefly, pregnant Sprague-Dawley rats were injected subcutaneously with 0, 0.1, 0.5, 1.0, or 2.0 mg/day of TB from gestational day 14-19. Dams were allowed to deliver and female pups were then assessed postnatally for reproductive development and function. In subsets of treated animals, fetuses were collected on prenatal day 18 to determine the fetal tissue concentrations of TB or T. Female fetuses from TP and TB-treated females had detectable concentrations of T or TB in both their amniotic fluid and reproductive tracts. Female offspring prenatally exposed to TP or TB displayed increased anogenital distance at postnatal day 2 (PND 2) and decreased numbers of normal areolae/nipples at PND 13 and as adults. Females exposed to TP or TB in utero also had delayed puberty and decreased fertility. Additionally, TP or TB-treated females displayed increased incidences of external genital malformations (e.g. cleft phallus, vaginal opening absent) and the presence of prostatic tissue. Interestingly, malformations were present in adult females exposed prenatally to either TB or TP, suggesting that TB is capable of significantly affecting testosterone-dependent tissues, including the DHT-dependent tissues | Mammalian EDC Study | Limited, only rats | Yes, maybe useful for terrestrial species |

| | | | | | | |
|---|---|---|---|---|---|---|
| An environmental androgen, 17beta-trenbolone, affects delayed-type hypersensitivity and reproductive tissues in male mice | Hotchkiss AK;Nelson RJ; | J Toxicol Environ Health A %2007 , Jan 15 | Recently, a growth promoter for farm animals, trenbolone acetate, was identified as an environmental androgen that potentially affects reproduction. Because androgens also suppress immunity, it was hypothesized that an active metabolite of trenbolone acetate, 17beta-trenbolone (TB), might impair immune responses. Castrated adult CD-1 mice were injected daily with either one of two different doses of 17beta-trenbolone (TB), testosterone propionate (TP), or corn oil (vehicle). The antigen-specific immune response was assessed by measuring delayed-type hypersensitivity (DTH) responses. Reproductive response was assessed by measuring reproductive tissue mass and determining testosterone concentrations. Mice treated with TB or TP displayed larger reproductive tissue mass than males treated with corn oil. Furthermore, males exposed to the highest dose of TB displayed a reduced DTH response compared to vehicle-treated animals. In comparison, TP, at a similar dose, only minimally reduced the DTH response. These data support the reproductive and potentially immunosuppressive effects of this environmental androgen, and raise the possibility of health concerns for individuals or populations in contact with high concentrations of TB | Mammalian EDC Study | Limited | Yes, maybe useful for terrestrial species |
| Of Mice and Men (and Mosquitofish): Antiandrogens and Androgens in the Environment | Hotchkiss, AK \| Ankley, GT \| Wilson, VS \| Hartig, PC \| Durhan, EJ \| Jensen, KM \| Martinovi, D \| Gray, LE | BIOSCIENCE \| 58 (11): 1037-1050 DEC 2008 | Androgens are hormones produced by the gonads and other endocrine organs of vertebrates. Testosterone, along with its metabolite dihydrotestosterone, is critical for the differentiation of the fetal male reproductive tract from an indifferent state, for the development of male traits during puberty, and for the maintenance of reproductive function in mature animals. The androgen signaling pathway is highly conserved in the reproductive system of all vertebrates from fish to humans; therefore, environmental chemicals have the potential to induce adverse effects in any vertebrate species. There are synthetic androgens present in the environment, and several pesticides and toxic substances display antiandrogenic activity For example, exposure to mixtures of antiandrogens during sexual differentiation results in cumulative adverse effect.; in male rat offspring. Continued characterization of the role of androgens in reproductive and other systems is warranted to enable better understanding of the potential adverse effects of chemical disruption of androgen signaling. | Mammalian EDC Study | Limited, review | Yes, species sensitivity |
| In utero exposure to the environmental androgen trenbolone masculinizes female Sprague-Dawley rats | Hotchkiss, AK \| Furr, J \| Makynen, EA \| Ankley, GT \| Gray, LE | TOXICOLOGY LETTERS \| 174 (1-3): 31-41 NOV 1 2007 | Recently, the occurrence of environmental contaminants with androgenic activity has been described from pulp and paper mill effluents and beef feedlot discharges. A synthetic androgen associated with beef production is trenbolone acetate, which is used to promote growth in cattle. A primary metabolite, 17(3 Trenbolone (TB), has been characterized as a potent androgen in both in vitro and in vivo studies with rats. The current study was designed to characterize the permanent morphological and functional consequences of prenatal TB exposure on female rats compared with those produced in an earlier study with testosterone propionate (TP). Female rat offspring were exposed to 0 mg/day, 0.1 mg/day, 0.5 mg/day, 1.0 mg/day, or 2.0 mg/day TB on gestational days 14-19. The 0.5 mg/day, 1.0 mg/day, or 2.0 mg/day TB groups displayed increases in neonatal anogenital distance (AGD) which persisted in the high dose group. Puberty was delayed in the high dose group and there were increased incidences of external genital malformations and the presence of male prostatic tissue in the 0.5 mg/day, 1.0 mg/day, or 2.0 mg/day groups. These changes were associated with amniotic fluid concentrations of TB that compare favorably with concentrations known to be active in both in vitro systems and in fish. | Mammalian EDC Study | Limited, only rats | Yes, maybe useful for terrestrial species |
| An environmental androgen, 17 beta-trenbolone, affects delayed-type hypersensitivity | Hotchkiss, AK \| Nelson, RJ | JOURNAL OF TOXICOLOGY AND ENVIRON HEALTH-PART A- | Recently, a growth promoter for farm animals, trenbolone acetate, was identified as an environmental androgen that potentially affects reproduction. Because androgens also suppress immunity, it was hypothesized that an active metabolite of trenbolone acetate, 17 beta-trenbolone (TB), might impair immune responses. Castrated adult CD-1 mice were injected daily with either one of two different doses of 17 beta-trenbolone (TB), testosterone propionate (TP), or corn oil (vehicle). The antigen-specific immune response was assessed by measuring delayed-type hypersensitivity (DTH) responses. Reproductive response was assessed by measuring reproductive tissue mass and determining testosterone concentrations. Mice treated with TB or TP displayed larger reproductive tissue mass than | Mammalian EDC Study | Limited, only rats | Yes, maybe useful for terrestrial species |

| | | | | | | |
|---|---|---|---|---|---|---|
| and reproductive tissues in male mice | | 70 (2): 138-140 2007 | males treated with corn oil. Furthermore, males exposed to the highest dose of TB displayed a reduced DTH response compared to vehicle-treated animals. In comparison, TP, at a similar dose, only minimally reduced the DTH response. These data support the reproductive and potentially immunosuppressive effects of this environmental androgen, and raise the possibility of health concerns for individuals or populations in contact with high concentrations of TB. | | | |
| OECD validation of phase 3 Hershberger assay in Korea using surgically castrated male rats with coded chemicals | Moon, HJ \| Kang, TS \| Kim, TS \| Kang, IH \| Ki, HY \| Kim, SH \| Han, SY | JOURNAL OF APPLIED TOXICOLOGY \| 29 (4): 350-355 MAY 2009 | As a participating laboratory for the OECD Hershberger validation program, we conducted a phase 3 trial to test the reliability of the Hershberger assay using coded substances. Male Sprague-Dawley rats were castrated at 6 weeks of age and allowed to recover for 8 days. All the coded substances were administered orally once daily for 10 consecutive days. In the antagonist version of the assay, 0.4 mg kg(-1) of testosterone propionate (TIP), a reference androgen, was co-administered with the coded compounds C, D, H, I or K, by a subcutaneous injection. As anticipated, TIP alone produced statistically significant increases in the five mandatory accessory sex organ weights. The coded substance L (trenbolone 40 mg kg(-1)), the test agonist, caused significant increases in the weights of the androgen-dependent tissues. The five coded compounds, p,p'-DDE at two doses (codes C and I), linuron at two doses (codes D and K) and flutamide (code H), all significantly decreased the weights of the TP-stimulated sex organs. These results suggest the OECD Hershberger assay to be a reliable screening method for detecting androgen agonists and antagonists. | Mammalian EDC Study | Limited, test validation work, Mammalian EDC | Yes |
| OECD validation of phase-3 Hershberger assay using the stimulated weanling male rat in Korea | Moon, HJ \| Kang, TS \| Kim, TS \| Kang, IH \| Kim, SH \| Han, SY | JOURNAL OF APPLIED TOXICOLOGY \| 30 (4): 361-368 MAY 2010 | The OECD has proposed a new, validated test guideline with the stimulated weanling male Hershberger assay to avoid the surgical castration step. In the present study, we assessed the relevance and reliability of the stimulated weanling Hershberger assay in four stages. All chemicals except for testosterone propionate (TP) were orally administered to sexually immature male rats of 22 days old for 10 days. The weights of four mandatory accessory sex organs, two additional reproductive tissues and optional systemic organs were evaluated. At the first two stages, TP, as reference androgen, significantly increased the weights of epididymides and accessory sex organs (ASO) at 1.0 mg kg(-1) and flutamide (FLU), as a positive antiandrogen control, decreased the TP-stimulated organ weights at 3.0 mg kg(-1). At stage 3, trenbolone (40 mg kg(-1)), an anabolic steroid, significantly increased ASO weights, and weak anti-androgens (DDE and linuron) decreased the TP-stimulated ASO weights at each high dose. The above results were confirmed in a blind test with coded substances provided by OECD. Compared with results from our previous castrated male assay, the intact weanling version is less sensitive than the castrated male version, in terms of a smaller response at the reference dose of TP or FLU. However, this study suggests that the stimulated weanling Hershberger assay can detect the effects of both potent and weak anti-androgens on androgen-producing and androgen-dependent tissues. | Mammalian EDC Study | Limited, test validation work, Mammalian EDC | Yes |
| Pharmacological and endocrinological studies on anabolic agents | Neumann F; | Environ Qual Saf Suppl %1976 (5):253 - 64 [Environmental quality and safety Suppleme | When used in connection with animal production the term 'anabolic agents' covers a wide range. Ther steroidal male and female sex hormones are included in this list, as are the nonsteroidal estrogens. For the clinician and for the endocrinologist, anabolics are only steroids chemically related to testosterone and 19-nortestosterone. Estrogens, though possessing anabolic properties, too, do not belong to this class. This paper will deal with anabolic agents in in the stricter sense of which mainly trenbolone acetate combined with hexestrol has been recommended for bull and heifer fattening. To consider possible consumer injury from ingestion of meat from anabolic agent treated animals, it is necessary to know the pharmacological properties of the agents, the doses producing certain effects or might produce, and the levels of residues in the meat. Trenbolone acetate will be compared with the following anabolic agents: methenolone acetate, methandrostenolone, nandrone, androstanazole, and 19-nortestosterone. The activity spectrum of trenbolone acetate is similar to that of 19-nortestosterone or those anabolics that are derived from 19-nortestosterone. The compound has about three times stronger androgenic effect than testosterone propionate. Its | Mammalian EDC Study | High, background/review | Yes |

| | | | | | | |
|---|---|---|---|---|---|---|
| | | nt ] | index of dissociation between anabolic/androgenic activity is 2--3. This index is 3--10 for the other anabolic agents. As regards the virilizing potency, trenbolone acetate is also on the top of the list. It seems that androgenicity and degree of virilization run paralle. The antigonadotropic activity (inhibition of ovulation and testicular growth) of trenbolone acetate exceeds that of testosterone propionate by the factor 3. The compound is not estrogenic and seemingly not or only weakly progestationally active. In principle, the androgenic activity (symptoms of virilization) as well as the antigonadotropic effect (disturbances of the menstrual cycle in women, inhibition of spermiogenesis in men) of trenbolone acetate might be noted. This risk, however, can be excluded by mere calculation. In rats, 0.1 mg/kg trenbolone acetate have an antigonadotropic effect. This corresponds to a daily dose of 5--7 mg in humans. By the same extrapolation, a daily human dose of 100 mg can be calculated for androgenic activity. Such factors of conversion are, of course, not precise because rats are much less sensitive to androgens and anabolics than humans. Thus, testosterone propionate is active only in daily doses of 10--20 mg. If in humans trenbolone acetate also has three times the activity of testosterone propionate, effects in man had to be counted with not less than a daily intake of 3--5 mg trenbolone acetate. The dose which is recommended for livestock fattening is 300 mg. IT can, therefore, be excluded almost with certainty that the meat would contain such large amounts of hormone residues | | | |
| Embryonic Exposure To Environmental Endocrine Disruptors Impairs Adult Reproductive Behavior And Hypothalamic Neuroendocrine Systems | Ottinger MA;Quinn M;Lavoie ET;McKernan M;Thompson N;Barton A;Abdelnabi M; | | Environmental chemicals (EDCs) are receiving attention for their capacity to interact with endogenous endocrine systems. However, most regulatory testing paradigms depend on gross measures, such as fertility or egg production and neglect to evaluate more subtle effects on neuroendocrine systems. Of particular concern are potential effects of EDCs on neural targets from embryonic exposure and subsequent impact on reproductive function in adults. Effects of estrogen- and androgen-active compounds were investigated on hypothalamic neural systems in the hatchling and adult. Fertile quail eggs (n=85-95/group) were injected with 20 ul sesame oil (control), 17beta estradiol, trenbolone, or DDE into the yolk at either embryonic day 4 or 11. These days were chosen to coincide with gonadal differentiation or sexual differentiation of hypothalamic systems. Birds were either raised to adulthood or sampled at hatch. Nontraditional measures for endocrine disruption were evaluated, including hypothalamic aromatase (AROM), catecholamines, and GnRH-I. Reproductive maturation and copulatory behavior were measured in birds that were raised. Results showed EDC exposure impaired reproductive behavior and altered rates of sexual maturation. Estradiol treatment increased AROM in hatchlings injected at E11; androgenic EDCs did not affect AROM. Catecholamines were altered by the treatments, especially the higher doses of EDCs and the differences were most apparent in adults. GnRH-I was sexually dimorphic with small effects from trenbolone that was observed in adults. In total, there were effects of EDCs that were visible in hatchlings in some variables. These effects become more pronounced in the sexually mature individuals, suggesting that chemical exposure of the embryo is expressed to some degree immediately (observable in the hatchling), but is more clinically apparent during activation of the reproductive neuroendocrine system. Finally, hypothalamic neurotransmitters that modulate reproductive function may provide valuable indices of endocrine disruption associated with later consequences of embryonic exposure to EDCs | Avian EDC Study | Uncertain as to source | Yes, need to check |

| | | | | | | |
|---|---|---|---|---|---|---|
| Is the gonadotropin releasing hormone system vulnerable to endocrine disruption in birds? | Ottinger, MA \| Lavoie, ET \| Thompson, N \| Bohannon, M \| Dean, K \| Quinn, MJ | GENERAL AND COMPARATIVE ENDOCRINOLOGY \| 163 (1-2): 104-108 AUG-SEP 2009 | Endocrine disrupting chemicals (EDCs) from a variety of sources occur widely in the environment, but relationships between exposure to EDCs and long term effects on bird populations can be difficult to prove. Embryonic exposure to EDCs may be particularly detrimental, with potential long-term effects on reproduction and ultimately individual fitness. Because many EDCs may have subtle sublethal effects, it is necessary to establish sensitive end points as biomarkers of EDC exposure in birds. Because the effects of EDCs may be both short- and long-term, it is important to determine if embryonic exposure impacts sexual differentiation and development of the reproductive axis in hatchlings and if there are effects on reproductive function in adults. Our studies have focused on the effects of estrogen- and androgen-active EDCs on the hypothalamic gonadotropin releasing hormone-I (GnRH-I) system in an avian model of precocial species, the Japanese quail. Estrogen- or androgen-active EDCs were administered between 0 and embryonic day 4, and hypothalamic GnRH-I was measured in hatchlings and adults. Treatment with vinclozolin and PCB126 depressed the concentration of embryonic GnRH-I peptide while methoxyclor had an inconsistent stimulatory effect. Treatment with atrazine or trenbolone had no significant effects on hypothalamic GnRH-I in adults. Overall these observations support the view that the developing avian GnRH-I neural system may be vulnerable to EDCs with potential to alter lifelong reproductive function. | Avian EDC Study | High, baseline work | Yes |
| The OECD program to validate the rat Hershberger bioassay to screen compounds for in vivo androgen and antiandrogen responses: Phase 2 dose-response studies | Owens, W \| Gray, LE \| Zeiger, E \| Walker, M \| Yamasaki, K \| Ashby, J \| Jacob, E | ENVIRONMENTAL HEALTH PERSPECTIVES \| 115 (5): 671-678 MAY 2007 | OBJECTIVE: The Organisation for Economic Co-operation and Development (OECD) has completed phase 2 of an international program to validate the rodent Hershberger bioassay. DESIGN: The Hershberger bioassay is designed to identify suspected androgens and antiandringens based on changes in the weights of five androgen-responsive tissues (ventral prostate, paired seminal vesicles and coagulating glands, the levator ani and bulbocavernosus muscles, the glans penis, and paired Cowper's or bulbourethral glands). Protocol sensitivity and reproducibility were tested using two androgen agonists (17 alpha-methyl testosterone and 17 beta-trenbolone), four antagonists [procymidone, vinclozolin, linuron, and 1,1-dichoro-2,2-bis-[(rho-chlorophenyl)ethylene (rho rho'-DDE)], and a 5 alpha-reductase inhibitor (finasteride). Sixteen laboratories from seven countries participated in phase 2. RESULTS: In 40 of 41 studies, the laboratories successfully detected substance-related weight changes in one or more tissues. The one exception was with the weakest antiandrogen, linuron, in a laboratory with reduced sensitivity because of high coefficients of variation in all tissue weights. The protocols performed well under different experimental conditions (e.g., strain, diet, housing protocol, bedding, vehicle). There was good agreement and reproducibility among laboratories with regard to the lowest dose inducing significant effects on tissue weights. CONCLUSIONS: The results show that the OECD Hershberger bioassay protocol is reproducible anal transferable across laboratories with androgen agonists, weak androgen antagonists, and a 5 alpha-reductase inhibitor. The next validation phase will employ coded test substances, including positive substances and negative substances having no androgenic or antiandrogenic activity. | Mammalian EDC Study | Limited, validation of method | Yes |

| | | | | | | |
|---|---|---|---|---|---|---|
| Reproductive toxicity of trenbolone acetate in embryonically exposed Japanese quail | Quinn, MJ \| Lavoie, ET \| Ottinger, MA | CHEMOSPHERE \| 66 (7): 1191-1196 JAN 2007 | This study was conducted to assess the effects of a one time embryonic exposure to trenbolone acetate on reproductive development and function in Japanese quail (Coturnix japonica). Embryos were exposed to either 0.05, 0.5, 5, or 50 mu g trenbolone or a sesame oil vehicle control at embryonic day 4. Onset of puberty, gonadal histopathology, sperm motility, cloacal gland size, and male copulatory behavior were assessed in adults. Trenbolone delayed onset of puberty in males, inhibited cloacal gland development, and reduced male reproductive behaviors. Industry laboratories have shown trenbolone acetate to be non-teratogenic in mammalian studies. Our study, however, shows that this one time in ovo exposure delayed onset of puberty in and suppressed adult copulatory behavior in quail males. These results suggest that this one time embryonic exposure to trenbolone may have disrupted development of either the central nervous system or the hypothalamic-pituitary-gonadal axis. This is the first study to demonstrate a demasculinizing effect on copulatory behavior in Japanese quail from embryonic exposure to a non-aromatizable androgenic chemical. More studies are needed to determine the mechanisms behind the observed effects. | Avian EDC Study | High | Yes |
| Immunotoxicity of trenbolone acetate in Japanese quail | Quinn, MJ \| McKernan, M \| Lavoie, ET \| Ottinger, MA | JOURN TOXICOL AND ENV HEALTH-PART A-70 (1): 88-93 2007 | Trenbolone acetate is a synthetic androgen that is currently used as a growth promoter in many meat-exporting countries. Despite industry laboratories classifying trenbolone as nonteratogenic, data showed that embryonic exposure to this androgenic chemical altered development of the immune system in Japanese quail. Trenbolone is lipophilic, persistent, and released into the environment in manure used as soil fertilizer. This is the first study to date to assess this chemical's immunotoxic effects in an avian species. A one-time injection of trenbolone into yolks was administered to mimic maternal deposition, and subsequent effects on the development and function of the immune system were determined in chicks and adults. Development of the bursa of Fabricius, an organ responsible for development of the humoral arm of the immune system, was disrupted, as indicated by lower masse, and smaller and fewer follicles at day 1 of hatch. Morphological differences in the bursas persisted in adults, although no differences in either two measures of immune function were observed. Total numbers of circulating leukocytes were reduced and heterophil-lymphocyte ratios were elevated in chicks but not adults. This study shows that trenbolone acetate is teratogenic and immunotoxic in Japanese quail, and provides evidence that the quail immune system may be fairly resilient to embryonic endocrine-disrupting chemical-induced alterations following no further exposure posthatch. | Avian EDC Study | High | Yes |
| Fluctuating asymmetry and growth as biomarkers for exposure to androgen disrupting chemicals in Japanese quail | Quinn, MJ \| Summitt, CL \| Burrell, K \| Ottinger, MA | ECOTOXICOLOGY \| 14 (6): 637-643 AUG 2005 | The effects of embryonic exposure to androgen disrupting chemicals (ADCs) on growth and fluctuating asymmetry (FA) were determined in Japanese quail chicks. Embryos were exposed to an anti-androgenic chemical, 1,1,1-Trichloro-2,2-bis(p-chlorophenyl)ethane (pp'-DDE) at 20 or 40 mu g, or to an androgenic chemical, trenbolone acetate, at 5 or 50 mu g on day one of incubation. Growth was measured by body weight and tarsus and culmen lengths from day of hatch until day 29. FA was measured as differences in right versus left lengths of the tarsus, radius, zygomatic process, and premaxilla in day old carcasses. No differences in FA were observed for either treatment. Embryonic exposure to DDE resulted in no significant differences in all measures of growth, although the same quail exhibited significant differences in immunological, reproductive, and behavioral measurements (reported elsewhere). Chicks exposed to trenbolone exhibited no differences in body weight or measures of FA at day of hatch, however, subsequent growth was inhibited. This study shows that although growth and FA are often used as measures of chemical stress experienced during embryonic development, they are not sensitive measures for exposure to these ADCs at these levels in Japanese quail. | Avian EDC Study | High | Yes |
| Seasonality, estrous cycle characterization, | Robeck, TR \| Steinman, KJ \| Greenwell, | REPRODUCTION \| 138 (2): | The reproductive physiology of the Pacific white-sided dolphin, Lagenorhynchus obliquidens, was characterized to facilitate the development of artificial insemination (AI) using cryopreserved spermatozoa. Specific objectives were to: 1) describe reproductive seasonality of the Pacific white sided dolphins; 2) describe urinary LH and ovarian | Mammalian EDC | Uncertain, doesn't appear to be | Yes, check design |

| | | | | | | |
|---|---|---|---|---|---|---|
| estrus synchronization, semen cryopreservation, and artificial insemination in the Pacific white-sided dolphin (*Lagenorhynchus obliquidens*) | M \| Ramirez, K \| Van Bonn, W \| Yoshioka, M \| Katsumata, E \| Dalton, L \| Osborn, S \| O'Brien, JK | 391-405 AUG 2009 | steroid metabolites during the estrous cycle; 3) correlate LH and ovarian steroidal metabolite patterns to ultrasound-monitored follicular growth and ovulation; and 4) assess the efficacy of synchronizing estrus, sperm collection/cryopreservation, and intrauterine insemination. Ovulations (64%, n=37) and conceptions (83%, n=18) occurred from August to October. Peak mean serum testosterone (24 ng/ml), cross-sectional testicular area (41.6 cm(2)), and sperm concentration (144.3 x 10(7) sperm/ml) occurred in July, August, and September respectively. Spermatozoa were only found in ejaculates from July to October. Estrous cycles (n=22) were 31 d long and were comprised of a 10 d follicular and 21 d luteal phase. Ovulation occurred 31.2 h after the onset of the LH surge and 19.3 h after the LH peak. Follicular diameter and circumference within 12 h of ovulation were 1.52 and 4.66 cm respectively. Estrus synchronization attempts with altrenogest resulted in 17 (22%) ovulatory cycles with ovulation occurring 21 d post-altrenogest. Ten AI attempts using cryopreserved semen resulted in five pregnancies (50%). The mean gestation length was 356 days (range 348-367). These data provide new information on the Pacific white-sided dolphin's reproductive physiology and collectively enabled the first application of AI in this species. | Study | a trenbolone study | |
| Is exposure to endocrine disrupting compounds during fetal/post-natal development affecting the reproductive potential of farm animals? | Sweeney, T | DOMESTIC ANIMAL ENDOCRINOLOGY \| 23 (1-2): 203-209 Sp. Iss. SI JUL 2002 | Concerns have been raised about the potential adverse effects on reproductive health and immune status of farm animals following exposure to a range of natural and synthetic environmental compounds that disrupt normal hormonal actions. These compounds range from natural plant oestrogens (e.g. genistein, coumesterol) and mycoestrogens (e.g. Aflatoxins, zearalenone) to growth promoting pharmaceuticals (e.g. trenbolone acetate, melengastrol acetate) to chemicals spread in water, sewage sludge or the atmosphere such as detergents and surfactants (e.g. octylphenol, nonylphenol), plastics (e.g. bisphenol-A, phthalates), pesticides (e.g. methoxychlor, dieldrin, DDT) and industrial chemicals (e.g. PCB, TCDD). These compounds are commonly termed 'endocrine disrupting compounds' (EDCs) or 'endocrine disruptors' due to their ability to act as either hormone agonists or antagonists or the ability to disrupt hormone synthesis, storage or metabolism. A similar group of compounds are called 'immunotoxicants' and are thought to affect the immune system mainly by disrupting B and T cell homeostasis. As more studies are performed it is becoming clear that many compounds can directly or indirectly affect both the endocrine and immune systems. The susceptibility of target tissues is related to the stage of development, the cumulative exposure dose and the immune status of the individual. While some of the effects of the EDCs on the endocrine and immune systems are quite distinct, many are subtle and identifying the causative agent from the vast array of environmental challenges including EDCs, nutrition, temperature, etc. can be problematic. Identifying the causative agent is confounded by the possibility that effects that are observed in the adult may be due to exposure to EDCs during fetal life. This has major implications for the determination of universal end-point measurements to assess exposure to EDCs in farm animals. | Mammalian EDC Study | Limited, review on effects in livestock | Yes, background |

| | | | | | | |
|---|---|---|---|---|---|---|
| Sensitive embryonic endpoints with in ovo treatment for detecting androgenic and anti-androgenic effects of chemicals in Japanese quail (Coturnix japonica) | Utsumi, T \| Yoshimura, Y | POULTRY SCIENCE \| 88 (5): 1052-1059 MAY 1 2009 | The aim of the current study was to establish the sensitive embryonic endpoints and a test system for detecting androgenic and anti-androgenic potential of chemicals using an in ovo treatment assay in Japanese quail. In ovo injection with 0 to 75 mu g of cyproterone acetate (CA) was performed on d 12 of incubation, followed by 0 to 300 mu g of testosterone propionate (TP) injection on d 13 and histological examination on d 16. Experimental groups were composed of control (twice injected corn oil injections; on d 12 and d 13, respectively), TP-L (corn oil and 30 mu g of TP), TP-H (corn oil and 300 mu g of TP), CA-L + TP-H (7.5 mu g of CA and 300 mu g of TP), and CA-H+ TP-H (75 mu g of CA and 300 mu g of TP). Histological examinations were performed in the cloacal gland, liver, kidneys, testes, ovaries, uropygial gland, and bursa of Fabricius. The cloacal gland consists of many glandular units (tubular gland structures) lined by developed or undeveloped glandular cells. The developed glandular cells were tall in height and contained mucous substance in the cytoplasm. The glandular units containing developed glandular cells were termed as the developing glandular units. The developing glandular units were observed in the TP-H, CA-L + TP-H, and CA-H + TP-H groups, but not in the control and TP-L groups, in both males and females. The ratio of developing glandular units to the total number of glandular units was significantly greater in TP-H than control and TP-L and was significantly decreased in CA-L + TP-H and CA-H + TP-H compared with TP-H in both males and females. The ratio was significantly greater in males than in females of CA-L + TP-H. No significant structural differences were observed in the other organs. These results suggest that the most sensitive endpoint of androgenic effects in quail embryo appeared in the cloacal glands. The ratio of the developing glandular units could be used for evaluation of androgenic and anti-androgenic effects of compounds. | Avian EDC Study | High | Yes |
| The approach taken and conclusions reached by the Joint FAO-WHO Expert Committee on Food Additives | van Leeuwen FX; | Ann Rech Vet %1991 | The synthetic anabolic steroid trenbolone acetate (TBA) was evaluated by the Joint FAO-WHO Expert Committee on Food Additives (JECFA) in 1981, 1982, 1987 and 1989. Effects on reproductive function in rats were observed, with no-effect level of 0.5 mg TBA/kg diet. No evidence was found for a teratogenic potential of TBA in rats. From the results of in vitro as well as in vivo mutagenicity assays it was concluded that TBA was probably not genotoxic and that the increased tumour incidence observed in long-term studies in mice and rats arose as a consequence of the hormonal activity of TBA. The concentration of sex hormones in the circulation was significantly reduced and histopathological abnormalities (particularly in testes, ovaries and uteri) were observed in male and female pigs fed with high doses of TBA. The marginal no-effect level for these effects was 0.1 mg/kg diet, equal to approximately 2 micrograms/kg bw. The 34th JECFA meeting established an acceptable daily intake of 0-0.02 micrograms/kg bw of TBA | Mammalian EDC Study | Limited, review with livestock focus | Yes, background |
| Persistence of endocrine disruption in zebrafish (Danio rerio) after discontinued exposure to the androgen 17β-trenbolone | Baumann L;Kn”rr S;Keiter S;Nagel T;Rehberger K;Volz S;Oberrauch S;Schiller V;Fenske M;Holbech H;Segner H;Braunbeck T; | Environ Toxicol Chem %2014 , Nov | The aim of the present study was to investigate the effects of the androgenic endocrine disruptor 17β-trenbolone on the sexual development of zebrafish (Danio rerio) with special emphasis on the question of whether adverse outcomes of developmental exposure are reversible or persistent. An exposure scenario including a recovery phase was chosen to assess the potential reversibility of androgenic effects. Zebrafish were exposed to environmentally relevant concentrations of 17β-trenbolone (1 ng/L-30 ng/L) from fertilization until completion of gonad sexual differentiation (60 d posthatch). Thereafter, exposure was either followed by 40 d of recovery in clean water or continued until 100 d posthatch, the age when zebrafish start being able to reproduce. Fish exposed for 100 d to 10 ng/L or 30 ng/L 17β-trenbolone were masculinized at different biological effect levels, as evidenced from a concentration-dependent shift of the sex ratio toward males as well as a significantly increased maturity of testes. Gonad morphological masculinization occurred in parallel with decreased vitellogenin concentrations in both sexes. Changes of brain aromatase (cyp19b) mRNA expression showed no consistent trend with respect to either exposure duration or concentration. Gonad morphological masculinization as well as the decrease of vitellogenin persisted after depuration over 40 d in clean water. This lack of recovery | Fish EDC | High | Yes, mechanism and toxicity endpoints |

| | | | | | | |
|---|---|---|---|---|---|---|
| | | | suggests that androgenic effects on sexual development of zebrafish are irreversible | | | |
| The maturity index as a tool to facilitate the interpretation of changes in vitellogenin production and sex ratio in the Fish Sexual Development Test | Baumann, L \| Holbech, H \| Keiter, S \| Kinnberg, KL \| Knorr, S \| Nagel, T \| Braunbeck, T | AQUAT TOXICOL \| 128: 34-42 MAR 15 2013 | In July 2011, the Fish Sexual Development Test (FSDT) has officially been adopted as OECD test guideline 234 for the detection of endocrine disrupting chemicals (EDCs). Sex ratio and vitellogenin (VTG) induction are the mandatory endocrine endpoints within this test, whereas gonad staging is only included as an option. In the present study, five FSDTs with zebrafish (Danio rerio) were conducted with EDCs with different modes of action (17 alpha-ethinylestradiol, dihydrotestosterone, 17 beta-trenbolone, prochloraz and 4-tert-pentylphenol). Results document that not only sex ratio and VTG production of the exposed fish were massively affected, but also gonad maturation. As a novel approach for the quantification of gonad maturation in zebrafish, the maturity index was developed to allow not only an improved assessment of dose-dependent EDC-related effects on gonad maturation, but also statistical analysis of histological data. VTG induction and maturity index showed an excellent correlation for all five EDCs tested. Most importantly, the maturity index often helped to find appropriate interpretations for results that seemed contradictory at first sight. Results show that histological analyses and their predictive power for population fitness are currently Underestimated and should become a standard component in the evaluation of potential EDCs. | Fish EDC | Limited, uses high dose with trenbolone as a "postive" model for method validation | Yes, species sensitivity |
| Development and validation of an OECD reproductive toxicity test guideline with the pond snail Lymnaea stagnalis (Mollusca, Gastropoda) | Ducrot, V \| Askem, C \| Azam, D \| Brettschneider, D \| Brown, R \| Charles, S \| Coke, M \| Collinet, M \| Delignette-Muller, ML \| Forfait-Dubuc, C \| Holbech, H \| Hutchinson, T \| Jach, A \| Kinnberg, KL \| Lacoste, C \| Le Page, G \| Matthiessen, P \| Oehlmann, J \| Rice, L \| Roberts, E \| Ruppert, K \| Davis, JE \| Veauvy, C \| Weltje, L \| Wortham, R \| | REGULATORY TOXICOLOGY AND PHARMACOLOGY \| 70 (3): 605-614 DEC 2014 | The OECD test guideline development program has been extended in 2011 to establish a partial life-cycle protocol for assessing the reproductive toxicity of chemicals to several mollusk species, including the great pond snail Lymnaea stagnalis. In this paper, we summarize the standard draft protocol for a reproduction test with this species, and present inter-comparison results obtained in a 56-day prevalidation ring-test using this protocol. Seven European laboratories performed semi-static tests with cultured snails of the strain Renilys (R) exposed to nominal concentrations of cadmium chloride (from 53 to 608 mu g Cd L-1). Cd concentrations in test solutions were analytically determined to confirm accuracy in the metal exposure concentrations in all laboratories. Physico-chemical and biological validity criteria (namely dissolved oxygen content >60% ASV, water temperature 20 +/- 1 degrees C, control snail survival >80% and control snail fecundity >8 egg-masses per snail over the test period) were met in all laboratories which consistently demonstrated the reproductive toxicity of Cd in snails using the proposed draft protocol. Effect concentrations for fecundity after 56 days were reproducible between laboratories (68 < EC50-56d < 124 mu g L-1) and were consistent with literature data. EC50-56d and EC10-56d values were comprised within a factor of 1.8 and 3.6, respectively, which is in the range of acceptable variation defined for reference chemicals in OECD test guidelines for invertebrates. The inter-laboratory reproducibility coefficient of variation (CV) for the Cd LC50-56d values was 8.19%. The inter-laboratory comparison of fecundity within the controls gave a CV of 29.12%, while exposure to Cd gave a CV of 25.49% based on the EC50-56d values. The OECD has acknowledged the success of this prevalidation exercise and a validation ring-test involving 14 laboratories in Europe, North- and South-America is currently being implemented using four chemicals (Cd, prochloraz, trenbolone and tributyltin). | Invertebrate EDC Study | High | Yes |

| | | | | | | |
|---|---|---|---|---|---|---|
| | Lagadic, L | | | | | |
| Ineffectiveness of steroid immersion treatments for sex reversal of brook trout | Galbreath, PF \| Stocks, SD | NORTH AMERICAN JOURNOF AQUACULTURE 61 3: 206-212 1999 | Immersion applications of androgenic steroids to sac fry of brook trout Salvelinus fontinalis were ineffective in inducing phenotypic sex reversal. Four experiments were conducted to test the effects of paired 2-h immersions in solutions of 17 alpha-methyldihydrotestosterone (MDHT) at 1 or 3 mg/L and applied at times ranging from 1 to 25 d after completion of hatch. In a fifth experiment, similar tests were conducted with 0.4 mg/L solutions of MDHT, 17 alpha-methyltestosterone, and trenbolone acetate. The experimental fish were of two types: normal mixed-sex fish and all-female gynogenetic fish. Phenotypic sex was determined by visual examination of the gonads when fish were sacrificed 6-8 months after initiation of feeding. In no experiment was there a significant increase in the percentage of males or a significant incidence of intersex gonads among treated fish. A generally low level of sterile and partially sterile fish was observed among control fish of both types, and these percentages tended to increase among treated fish. | Aquaculture Application Study | Limited, dosing not environmentally relevant | Yes, species sensitivity |
| Efficacy of trenbolone acetate in sex inversion of the blue tilapia Oreochromis aureus | Galvez, JI \| Morrison, JR \| Phelps, RP | JOURN WORLD AQUACUL SOC 27 (4): 483-486 1996 | Trenbolone acetate (TEA) is a synthetic anabolic androgenic steroid approved in the United States as a growth promoter for beef cattle. Pooled populations of blue tilapia Oreochromis aureus with more than 98% males were produced by feeding diets containing TEA. This hormone effectively inversed the sex of blue tilapia when doses of 25-100 mg/kg of diet were fed for 28 d. Fish treated with 60 mg of 17-alpha-methyltestosterone (MT)/kg of feed resulted in 88.7% males. The percentage of male tilapia masculinized with TEA was significantly higher ($P < 0.05$) than with MT. Intersex fish were found in both control and hormone-treated groups, and were significantly more common ($P < 0.05$) in the MT-treated batches. Survival, feed conversion ratio, and average weight of O. aureus fry fed diets containing either TEA, MT or no hormone were not different ($P > 0.05$) at the end of the 28-d treatment period. | Aquaculture Application Study | Limited, oral dosing | Yes, species sensitivity |
| Trouble with trenbolone? Examining the influence of a common run-off pollutant on Gambusia holbrooki development and behavior | Guise, EG \| O'Brien, S | INTEGR AND COMP BIOLOGY \| 55: E266-E266 Suppl. 2015 | Trenbolone is a relatively new endocrine disrupting chemical that acts as a testosterone mimic, and is considered to be one of the most powerful anabolic steroids in use (Saaristo 2013). Trenbolone has three times the bonding affinity of testosterone and has a half-life of ¾ a year (Orlando 2004). With extensive usage in the beef cattle industry as a growth promoter, trenbolone has been found to appear in animal waste and runoff from cattle feed lots (Bartelt-Hunt 2012). Such a stable and potent molecule being released into the environment could potentially cause devastating effects on freshwater environments. As a potent androgen, trenbolone could increase masculine traits in freshwater species, and may disrupt reproductive processes. Here we explore the effects of ecologically relevant levels of trenbolone, as determined by sampling, on the freshwater fish species, Gambusia holbrooki. We elucidate influences on morphological, breeding, and behavioral characteristics in the fish and their subsequent offspring | Fish EDC | Likely low, no abstract | Yes, need to check |

| | | | | | | |
|---|---|---|---|---|---|---|
| Endocrine Disrupting Compounds Alter Risk-Taking Behavior in Guppies (Poecilia reticulata) | Heintz, MM \| Brander, SM \| White, JW | ETHOLOGY \| 121 (5): 480-491 MAY 2015 | Endocrine disrupting compounds (EDCs) enter aquatic habitats from a variety of anthropogenic sources and can mimic, block, or modulate the synthesis of natural hormones. EDCs affect both reproductive and non-reproductive behaviors because hormones mediate responses associated with aggression and fear. We examined the effects of two EDCs on risk-taking behaviors in guppies (Poecilia reticulata). We quantified risk-taking in terms of propensity to forage in a risky location and tendency to join groups in the presence of a predator. We found that male and female guppies responded oppositely to environmentally relevant concentrations of an estrogenic EDC, 17-ethinylestradiol (EE2), or an androgenic EDC, 17-trenbolone (TB). Males decreased risk-taking with increasing EE2 concentration (as predicted), but females increased risk-taking (contrary to prediction). In contrast, females increased risk-taking with increasing TB concentrations (as predicted), but males decreased risk-taking (contrary to prediction). These results did not match our expectation that EE2 would reduce risk-taking and TB would increase risk-taking in both sexes. We suspect EE2 and TB produced these counterintuitive effects by downregulating their corresponding hormone receptors and thus reducing levels of circulating endogenous hormones in females and males, respectively. These results show that EDCs can alter fish behavior and potentially reduce fitness in unexpected ways. | Fish EDC | High | Yes, behavioral endpoints |
| Comparison of estrogen-responsive plasma protein biomarkers and reproductive endpoints in sheepshead minnows exposed to 17 beta-trenbolone | Hemmer, MJ \| Cripe, GM \| Hemmer, BL \| Goodman, LR \| Salinas, KA \| Fournie, JW \| Walker, CC | AQUAT TOXICOL \| 88 (2): 128-136 JUN 23 2008 | Protein profiling can be used for detection of biomarkers that can be applied diagnostically to screen chemicals for endocrine modifying activity. In previous studies, mass spectral analysis revealed four peptides (2950.5, 2972.5, 3003.4, 3025.5m/z) in the plasma of estrogen agonist-treated male and gravid female sheepshead minnows (Cyprinodon variegatus, SHM), which served as distinct estrogenic biomarkers. In this study, a 21-day reproductive assay with adult SHM was conducted to investigate possible dose-related effects of the synthetic androgen, 17beta-trenbolone, on expression of these four estrogen-responsive peptides. In addition, the response of the peptide biomarkers were compared to traditional reproductive endpoints of fecundity, histopathology, secondary sex characteristics, length, weight, hepatosomatic index, female gonadosomatic index and plasma vitellogenin (VTG) levels. Fish were continuously exposed to 0.005, 0.05, and 5.0 microg/l, a solvent control (triethylene glycol, TEG), and a seawater control (SW) using an intermittent flow-through dosing system. Plasma was analyzed for the presence of the four peptide biomarkers by MALDI-TOF MS and VTG protein by quantitative ELISA. Male fish from the trenbolone treatments and controls showed no expression of the four peptide biomarkers or measurable levels of VTG. The estrogen-responsive biomarkers and plasma VTG were constitutively expressed in females from the SW, TEG, 0.005 and 0.05 microg/l exposures. All four peptide biomarkers were significantly reduced ($p<0.0002$ to $p<0.005$) at the 5.0 microg/l treatment level which corresponded with significant reductions in fecundity and changes in ovarian morphology. A distinct but non-significant reduction in VTG was also observed in female fish from the 5.0 microg/l treatment. Results of this study suggest application of these estrogen-responsive protein biomarkers may be a cost effective alternative to fecundity measures which are labor intensive and expensive to conduct. | Fish EDC | High | Yes, mechanistic and apical endpoints |

| | | | | | | |
|---|---|---|---|---|---|---|
| Application of protein expression profiling to screen chemicals for androgenic activity | Hemmer, MJ \| Salinas, KA \| Harris, PS | AQUAT TOXICOL \| 103 (1-2): 71-78 MAY 2011 | Protein expression changes can be used for detection of biomarkers that can be applied diagnostically to screen chemicals for endocrine modifying activity. In this study, surface enhanced laser desorption/ionization time-of-flight mass spectrometry (SELDI-TOF-MS) coupled with a short term fish assay was used to investigate changes in plasma protein expression as a means to screen chemicals for androgenic activity. Adult gravid female sheepshead minnows (Cyprinodon variegatus) were placed into separate aquaria for seawater control, ethanol solvent control, and the following androgen agonist treatments at 5.0µg/L: dihydrotestosterone (DHT), methyldihydrotestosterone (MDHT), testosterone (T), methyltestosterone (MT) and trenbolone (TB). Treatments of 0.6µg/L endosulfan and 40µg/L chlorpyrifos (CP) served as non-androgenic negative stressor controls. Test concentrations were maintained using an intermittent flow-through dosing apparatus supplying exposure water at 20L/h. Fish were sampled at 7 days, the plasma diluted, processed on weak cation exchange CM10 ProteinChip arrays and analyzed. Spectral processing resulted in 249 individual m/z peak clusters for the androgen exposed fish. Partial least squares-discriminant analysis was used to develop an androgen-responsive model using sample spectra from exposures with DHT and unexposed solvent control fish as the training set. The androgen classification model performed with ≥79% specificity (% true negative) and ≥70% sensitivity (% true positive) for non-aromatizable androgens. The aromatizable androgens T and MT were classified as androgenic with specificities of 42 and 79%, respectively. The reduction in sensitivity observed with T is thought to be caused by its metabolic conversion to an estrogen by aromatase. The results of these studies show diagnostic plasma protein expression models can correctly classify chemicals by their androgenic activity using a combination of high throughput mass spectrometry and multivariate approaches. | Fish EDC | Limited, only protein expression | Yes, mechanistic endpoints |
| Anabolic effect induced by trenbolone acetate steroid on the Carassius auratus (Pisces : Cyprinidae) growth | Herrera, SM \| Demesa, VT \| Zamora, NS \| Pena, EM | HIDROBIOLOGICA \| 18 (1): 41-50 MAR 2008 | The anabolic efficiency of steroid trenbolone acetate was evaluated in 60 days old juveniles of Carassius auratus. Fish were exposed during 120 days to steroid at a dose of 300 mg/kg food. Total length, standard length, height and weight were registered every two weeks. The benefit of the steroid was characterized with a model that relates the weight as a function of time, coupled to other two models: one where size is related with time and an alometric one which correlates weight with size. The models showed that growth of steroid treated fish was superior to that of untreated (control) fish, differing significantly ($p < 0.001$), while the alometric model for each treatment, indicated a similar growth ($p > 0.05$). Analysis of the variability of the three models demonstrated that the estimates adequately described the growth. This was further confirmed by the determination coefficient (r2) that fluctuated between 72.9 and 93.5% and by the distribution analysis of size and weight by means of box plots. It was concluded that application of the steroid trenbolone acetate to Carassius auratus was successful. A survival rate of 100% was registered coupled to an efficient anabolic effect, since a 48.0% gain of biomass and 41.3% increase in size were obtained as compared to the control group | Aquaculture Application Study | Limited, oral dosing | Yes, species sensitivity |

| | | | | | | |
|---|---|---|---|---|---|---|
| Detection of endocrine disrupters: Evaluation of a Fish Sexual Development Test (FSDT) | Holbech, H \| Kinnberg, K \| Petersen, GI \| Jackson, P \| Hylland, K \| Norrgren, L \| Bjerregaard, P | COMPE BIOCHEM AND PHYSIOL C-TOXICOLO & PHARMACOL 144 (1): 57-66 P 2006 | Managed by the Organisation for Economic Co-operation and Development (OECD), a comprehensive work is carried out in numerous laboratories to develop test guidelines for the detection of endocrine disrupting chemicals in humans, and various animal species. Development of tests to detect chemicals with endocrine disrupting properties in fish is a part of that work. A Fish Sexual Development Test (FSDT) (an extension of the existing OECD TG 210, fish early life stage toxicity test), proposed as an international test guideline for the detection of endocrine disrupting chemicals, was evaluated by water exposure of juvenile zebrafish to the three natural estrogens: estrone, 17β-estradiol, and estriol and the synthetic androgen trenbolone (trenbolone acetate). As endpoints, vitellogenin induction and histological changes including changes in sex ratios were investigated. The sex ratio was significantly altered towards females from 49 ng/l estrone, 54 ng/l 17β-estradiol and 22 µg/l estriol, respectively. An all male population was observed from exposure to 9.7 ng/l trenbolone and above. Significant vitellogenin induction in whole body homogenate was measured after exposure to 14 ng/l estrone, 54 ng/l 17β-estradiol and 0.6 µg/l estriol, respectively. Significant vitellogenin reduction was measured after exposure to 193 ng/l trenbolone or higher. The present results provide strong evidence that the FSDT is a sensitive test toward estrogenic and especially androgenic exposure and the validation of the FSDT as an OECD test guideline should continue | Fish EDC | Limited, study using trenbolone as model compound for test development/ confirmation | Yes, species sensitivity |
| FUNCTIONAL BEHAVIOR AND REPRODUCTION IN ANDROGENIC SEX REVERSED ZEBRAFISH (DANIO RERIO) | Larsen, MG \| Baatrup, E | ENVIRON TOXICOL AND CHEM 29 (8): 1828-1833 2010 | Endocrine-disrupting chemicals released into natural watercourses may cause biased sex ratios by sex reversal in fish populations. The present study investigated the androgenic sex reversal of zebrafish (Danio rerio) exposed to the androgenic compound 17beta-trenbolone (TB) and whether sex-changed females would revert to the female phenotype after cessation of TB exposure. 17beta-Trenbolone is a metabolite of trenbolone acetate, an anabolic steroid used as a growth promoter in beef cattle. 17beta-Trenbolone in runoff from cattle feedlots may reach concentrations that affect fish sexual development. Zebrafish were exposed to a concentration of 20 ng/L TB in a flow-through system for five months from egg until sexual maturity. This resulted in an all-male population. It was further found that all these phenotypic males displayed normal male courtship behavior and were able to reproduce successfully, implying that the sex reversal was complete and functional. None of the phenotypic males developed into females after six months in clean water, demonstrating that androgenic sex reversal of zebrafish is irreversible | Fish EDC | High, chronic responses (although only at high doses) | Yes |
| The trenbolone acetate affects the immune system in rainbow trout, Oncorhynchus mykiss | Massart, S \| Redivo, B \| Flarnion, E \| Mandiki, SNM \| Falisse, E \| Milla, S \| Kestemont, P | AQUAT TOXICOL \| 163: 109-120 JUN 2015 | In aquatic systems, the presence of endocrine-disrupting chemicals (EDC) can disrupt the reproductive function but also the immune system of wildlife. Some studies have investigated the effects of androgens on the fish immune parameters but the mechanisms by which the xenoandrogens alter the immunity are not well characterized. In order to test the effects of trenbolone acetate (TbA) on fish immune system, we exposed rainbow trout male juveniles during three weeks to TbA levels at 0.1 and 1 mu g/L. The present results suggest that TbA impacts, in a tissue-dependent manner, the rainbow trout immunity by affecting primarily the humoral immunity. Indeed, TbA inhibited lysozyme activity in plasma and liver and enhanced the alternative complement pathway activity (ACHSO) in kidney. In plasma, the modulation of the complement system was time-dependent. The mRNA expression of genes encoding some cytokines such as renal TGF-beta 1, TNF-alpha in skin and hepatic IL-1 beta was also altered in fish exposed to TbA. Regarding the cellular immunity, no effect was observed on the leucocyte population. However, the expression of genes involved in the development and maturation of lymphoid cells (RAG-1 and RAG-2) was decreased in TbA-treated fish. Among those effects, we suggest that the modulation of RAG-1 and mucus apolipoprotein-A1 gene expression as well as plasma and hepatic lysozyme activities are mediated through the action of the androgen receptor. All combined, we conclude that trenbolone affects the rainbow trout immunity. | Fish EDC | High, immune effects | Yes |

| | | | | | | |
|---|---|---|---|---|---|---|
| The effects of estrogenic and androgenic endocrine disruptors on the immune system of fish: a review | Milla, S \| Depiereux, S \| Kestemont, P | ECOTOXICOLOGY \| 20 (2): 305-319 MAR 2011 | During the last decade, a number of studies have shown that, in addition to their classically described reproductive function, estrogens and androgens also regulate the immune system in teleosts. Today, several molecules are known to interfere with the sex-steroid signaling. These chemicals are often referred to as endocrine disrupting contaminants (EDCs). We review the growing evidence that these compounds interfere with the fish immune system. These studies encompass a broad range of approaches from field studies to those at the molecular level. This integrative overview improves our understanding of the various endocrine-disrupting processes triggered by these chemicals. Furthermore, the research also explains why fish that have been exposed to EDCs are more sensitive to pathogens during gametogenesis. In this review, we first discuss the primary actions of sex-steroid-like endocrine disruptors in fish and the specificity of the fish immune system in comparison to mammals. Then, we review the known interactions between the immune system and EDCs and interpret the primary effects of sex steroids (estrogens and androgens) and their related endocrine disruptors on immune modulation. The recent literature suggests that immune parameters may be used as biomarkers of contamination by EDCs. However, caution should be used in the assessment of such immunotoxicity. In particular, more attention should be paid to the specificity of these biomarkers, the external/internal factors influencing the response, and the transduction pathways induced by these molecules in fish. The use of the well-known mammalian models provides a useful guide for future research in fish. | Fish EDC | Limited, broad review on immune effects | Yes, background |
| Trenbolone causes irreversible masculinization of zebrafish at environmentally relevant concentrations | Morthorst, JE \| Holbech, H \| Bjerregaard, P | AQUAT TOXICOL \| 98 (4): 336-343 JUL 15 2010 | Feminization of fish caused by certain estrogenic compounds e.g. 17 alpha-ethinylestradiol (EE2) has been shown to be partly reversible. So far it has not been studied if this applies for androgenic compounds too. The androgenic steroid trenbolone acetate (TbA) is used as growth promoter in beef cattle in the United States, South America, and Australia. TbA metabolites are stable in animal waste and have been detected in surface waters associated with feedlot areas and studies on both fish and mammals have demonstrated a strong androgenic effect of those metabolites. Zebrafish (Danio rerio) were exposed to environmentally relevant concentrations of the TbA metabolite 17 beta-trenbolone from 0 to 60 days post-hatch (dph) and either sacrificed at 60 dph, transferred to clean water for 170 days or kept in exposure for 170 days. At 60 dph gonadal histology and vitellogenin analyses revealed all-male populations in groups exposed to 15.5 and 26.2 ng/L, and at 9.2 ng/L a skewed sex ratio towards males was observed. After the depuration period no sign of reversibility was observed. Environmentally relevant concentrations of 17 beta-trenbolone cause a strong and irreversible masculinization of zebrafish and that raises concern about the effects of androgenic discharges in the aquatic environment In addition this study also aids in understanding of the so far unknown sex determination process in zebrafish. | Fish EDC | High | Yes |
| An androgenic agricultural contaminant impairs female reproductive behaviour in a freshwater fish | Saaristo M;Tomkins P;Allinson M;Allinson G;Wong BB; | Plos One %2013 | Endocrine disrupting chemicals (EDCs) are a large group of environmental pollutants that can interfere with the endocrine system function of organisms at very low levels. One compound of great concern is trenbolone, which is widely used as a growth promoter in the cattle industry in many parts of the world. The aim of this study was to test how short-term (21-day) exposure to an environmentally relevant concentration of 17β-trenbolone (measured concentration 6 ng/L) affects reproductive behaviour and fin morphology in the eastern mosquitofish (Gambusia holbrooki). The mosquitofish is a sexually dimorphic livebearer with males inseminating females using their modified anal fin, the gonopodium, as an intromittent organ. Although the species has a coercive mating system, females are able to exert some control over the success of male mating attempts by selectively associating with, or avoiding, certain males over others. We found that females exposed to trenbolone approached males less and spent more time swimming away from males than non-exposed (control) females. By contrast, we found no difference in the behaviour of exposed and non-exposed males. Furthermore, exposure did not affect the anal fin morphology of males or females. This is the first study to demonstrate that exposure to an androgenic EDC can impair female (but | Fish EDC | High, behavioral study | Yes |

| | | | | | | |
|---|---|---|---|---|---|---|
| | | | not male) behaviour. Our study illustrates how anthropogenic contaminants can have sex-specific effects, and highlights the need to examine the behavioural responses of environmental contaminants in both sexes | | | |
| Effect of parental exposure to trenbolone and the brominated flame retardant BDE-47 on fertility in rainbow trout (Oncorhynchus mykiss) | Schultz, I \| Brown, KH \| Nagler, JJ | MARINE ENVIRON MENTAL RESEARCH \| 66 (1): 47-49 JUL 2008 | We exposed sexually maturing male rainbow trout (Oncorhynchus mykiss) to BDE-47 (a polybrominated diphenyl ether) and female rainbow trout to trenbolone (an anabolic steroid). Male trout were orally exposed for 17 days to 55 pg/kglday BDE-47 and female trout continuously exposed for 60-77 days to a measured trenbolone water concentration of 35 ng/L. After the exposure, eggs and semen were collected and in vitro fertilization trials performed using a sperm:egg ratio of 300,000: 1. In the BDE-47 study, eggs from control females were fertilized with semen from exposed males, while in the trenbolone study, eggs from exposed females were fertilized with semen from control males. All treatments were evaluated at two-three early developmental time-points representing first cleavage (0.5 day), embryonic keel (9 days), and eyed stages (19 days), respectively. The results indicated that BDE-47 exposure did not alter fertility as embryonic survival was similar between control and exposed groups. Trenbolone exposure also did not alter embryo survival. However, in the embryos fertilized with eggs from trenbolone exposed females, a noticeable delay in developmental progress was observed. On day 19 when eye development is normally complete, the majority of the embryos either lacked eyes or displayed under-developed eyes, in contrast to control embryos. This finding suggests steroidal androgen exposure in sexually maturing female rainbow trout can impact developmental timing of F1 offspring. | Fish EDC | Limited, only one dose | Yes, species sensitivity |
| Effects of an androgenic growth promoter 17beta-trenbolone on masculinization of Mosquitofish (Gambusia affinis affinis) | Sone K;Hinago M;Itamoto M;Katsu Y;Watanabe H;Urushitani H;Tooi O;Guillette LJ;Iguchi T; | Gen Comp Endocrinol %2005 , Sep 1 | Endocrine disrupting chemicals can affect normal hormone dependent processes through numerous mechanisms, including ligand mimicky. 17beta-Trenbolone (TB), a pharmaceutical, androgenic, anabolic steroid, is a potent agonist of androgen receptors, and has been extensively used as a growth promoter for beef cattle in the US. The effects of TB on adult and newborn mosquitofish (Gambusia affinis affinis) were examined. Two forms of mosquitofish androgen receptor (AR), ARalpha and ARbeta, were cloned. The mRNA expression levels of ARalpha and ARbeta were transiently increased in the anal fin of adult females at day 3 following exposure to TB (1-10 microg/L) or methyltestosterone (MT) (0.1-10 microg/L), a pharmaceutical androgen used as a positive control. Gonopodium differentiation from the adult female anal fin was induced after 28 days of exposure to TB (1-10 microg/L) or MT (0.1-10 microg/L). Gonopodium differentiation also was induced in all mosquitofish fry exposed for 28 days to 0.3, 1 or 10 microg/L TB. Furthermore, spermatozoa were observed histologically in the testes of male fry exposed for 28 days to 1 or 10 microg/L TB; spermatozoa are normally observed only in the testes of mature males. Surprisingly, all female fry exposed for 28 days to 1 or 10 microg/L TB displayed the formation of an ovotestis, as spermatozoa were found in the ovary. Thus, TB, like MT, induced masculinization of the anal fin accompanied by a transient up-regulation of ARalpha and ARbeta in adult females. TB also induced differentiation of the anal fin into a gonopodium in fry of both sexes, stimulated precocious spermatogenesis in the testes of males and the formation of ovotestes in females | Fish EDC | High, variety of mechanistic and apical endpoints, albeit it at pretty high doses | Yes |
| Evidence of small modulation of ethinylestradiol induced effects by concurrent exposure to trenbolone in male eelpout Zoarces viviparus | Velasco-Santamar¡a YM;Bjerregaard P;Korsgaard B; | Environ Pollut %2013 , Jul | The interaction of xenobiotics is common in aquatic ecosystems; therefore, we wanted to evaluate if trenbolone (TB) modulates the effects of 17α-ethinylestradiol (EE2). Male eelpout (Zoarces viviparus) were exposed to 5 ng L(-1) EE2 continuously for 19 d (EE2-C) or discontinuously (11 d, EE2-D) alone or in combination with low (50 ng L(-1), TBL) or high (500 ng L(-1), TBH) concentrations of TB (19 d). Exposure to EE2 caused reduced gonadosomatic index, increased plasma vitellogenin concentrations, up-regulated vtg and era mRNA expression and severe alterations in gonadal histology. TBL and TBH did not affect plasma vitellogenin, era or vtg mRNA expression. TBL and TBH did not counteract the EE2-induced increase in plasma vitellogenin and reduction in 11-ketotestosterone whereas TBH counteracted the EE2 induced increase in vtg and era mRNA expression. Exposure to TBH and EE2-C + TBH lead to severe gonadal histology alterations. TBL and EE2-D + TBH exposed fish showed less histopathological alterations | Fish EDC | Limited, some interaction data, but not exhaustive study | Yes |

| | | | | | | |
|---|---|---|---|---|---|---|
| Effects of 17beta-trenbolone in male eelpout Zoarces viviparus exposed to ethinylestradiol | Velasco-Santamar¡a YM;Madsen SS;Bjerregaard P;Korsgaard B; | Anal Bioanal Chem %2010 , Jan | To evaluate the interaction between 17beta-trenbolone (TB) and 17alpha-ethinylestradiol (EE2), male eelpout, Zoarces viviparus, was exposed for 21 days (April to May 2008) to 5 ng l(-1) EE2 and 5 or 20 ng l(-1) TB, separately or in combination in a flow-through SW system. The effects on hepatosomatic (HSI) and gonadosomatic index (GSI), plasma vitellogenin (Vtg) concentration, gonadal histology, hepatic and testicular Vtg mRNA and estrogen receptor (ERalpha) mRNA expression were investigated. No effects on HSI were observed. A significant decrease was observed in the GSI of all males exposed to EE2 ( < 0.7%) when compared to controls (1.4%). Histological alterations and immature stages were observed in the testis of all exposed males; however, males exposed to EE2 were the most affected. Increased tubule number and proportionally decreased tubule diameter were observed in the testis of all EE2 groups. No effects in Vtg mRNA expression were observed in the testis; however, a significant decrease in testis ERalpha mRNA was observed in males exposed to 20 ng l(-1) TB. The groups exposed to EE2 showed a significant increase in plasma Vtg ( > 300-fold), hepatic Vtg mRNA ( > 450-fold), and ERalpha mRNA ( > 100-fold) when compared to controls. This study shows that lower concentrations of 17beta-trenbolone are unable to counteract the EE2 estrogenic effects when the exposure is simultaneous | Fish EDC | Limited, some interaction data, but not exhaustive study | Yes |
| Anabolic and androgenic effect of steroid trenbolone acetate on guppy (Poecilia reticulata) | Zamora, HS \| Hernandez, AA \| Herrera, SM \| Pena, EM | VETERINARIA MEXICO \| 39 (3): 269-277 2008 | The effect of semi-synthetic steroid trenbolone acetate (TBA) on the ornamental fish Poecilia reticulata (guppy) was studied. The steroid at a dose of 300 mg/kg feed was administered to 30 days old juvenile specimens during 60 days. Forty days after treatment was ended, an evaluation aimed to determine the steroid residual effect was undertaken. Survival, masculinization ratio and the drug anabolic effect were analyzed. Results showed TBA to be effective to induce masculinization, differing (P < 0.001) with the control group, which registered 32% males and 68% females. TBA turned out to be an excellent anabolic as well, since treated fish showed weight increase, a larger body and an increased size of caudal fin. The steroid did not cause any damage on the treated population. The survival factor was 93.3% for the treated fish as compared to 83% for the control group. | Fish EDC | Limited, oral dosing | Yes, species sensitivity |
| Steroid-induced meiotic division in xenopus laevis oocytes: Surface and calcium | Baulieu EE;Godeau F;Schorderet M;Schorderet-Slatkine S; | Nature(London) 275 :593 -598 ,1978 Tax - Xenopus Laevis | Progesterone reinitiates meiotic maturation in Xenopus oocytes. Evidence is reported which indicates that the steroid acts at the level of the cell surface and suggests that an induced change of Ca2+ distribution triggers in turn a cascade of cytoplasmic events including protein synthesis and germinal vesicle (nucleus) breakdown. These novel features of steroid hormone action in amphibian oocytes are discussed in relation to presently accepted views of the mechanism of action of steroid hormones in somatic cells. | Amphibian EDC Study | Uncertain | Yes, species sensitivity |

| | | | | | | |
|---|---|---|---|---|---|---|
| Custom microarray construction and analysis for determining potential biomarkers of subchronic androgen exposure in the Eastern Mosquitofish (Gambusia holbrooki) | Brockmeier, EK \| Yu, FH \| Amador, DM \| Bargar, TA \| Denslow, ND | BMC GENOMICS \| 14: - SEP 28 2013 | Background: The eastern mosquitofish (Gambusia holbrooki) has the potential to become a bioindicator organism of endocrine disrupting chemicals (EDCs) due to its androgen-driven secondary sexual characteristics. However, the lack of molecular information on G. holbrooki hinders its use as a bioindicator coupled with biomarker data. While traditional gene-by-gene approaches provide insight for biomarker development, a holistic analysis would provide more rapid and expansive determination of potential biomarkers. The objective of this study was to develop and utilize a mosquitofish microarray to determine potential biomarkers of subchronic androgen exposure. To achieve this objective, two specific aims were developed: 1) Sequence a G. holbrooki cDNA library, and 2) Use microarray analysis to determine genes that are differentially regulated by subchronic androgen exposure in hepatic tissues of 17 beta-trenbolone (TB) exposed adult female G. holbrooki. Results: A normalized library of multiple organs of male and female G. holbrooki was prepared and sequenced by the Illumina GA IIx and Roche 454 XLR70. Over 30,000 genes with e-value <= 10(-4) were annotated and 14,758 of these genes were selected for inclusion on the microarray. Hepatic microarray analysis of adult female G. holbrooki exposed to the vehicle control or 1 mu g/L of TB (a potent anabolic androgen) revealed 229 genes upregulated and 279 downregulated by TB (one-way ANOVA, p < 0.05, FDR alpha = 0.05, fold change > 1.5 and < -1.5). Fifteen gene ontology biological processes were enriched by TB exposure (Fisher's Exact Test, p < 0.05). The expression levels of 17 beta-hydroxysteroid dehydrogenase 3 and zona pellucida glycoprotein 2 were validated by quantitative polymerase chain reaction (qPCR) (Student's t-test, p < 0.05). Conclusions: Coupling microarray data with phenotypic changes driven by androgen exposure in mosquitofish is key for developing this organism into a bioindicator for EDCs. Future studies using this array will enhance knowledge of the biology and toxicological response of this species. This work provides a foundation of molecular knowledge and tools that can be used to delve further into understanding the biology of G. holbrooki and how this organism can be used as a bioindicator organism for endocrine disrupting pollutants in the environment. | Fish EDC | Limited, only gene expression measured | Yes, basic mechanisms |
| The genomic transcriptional response of female fathead minnows (Pimephales promelas) to an acute exposure to the androgen, 17beta-trenbolone | Dorts J;Richter CA;Wright-Osment MK;Ellersieck MR;Carter BJ;Tillitt DE; | Aquat Toxicol %2009 , Jan 18 | We investigated the genomic transcriptional response of female fathead minnows (Pimephales promelas) to an acute (4 days) exposure to 0.1 or 1.0microg/L of 17beta-trenbolone (TB), the active metabolite of an anabolic androgenic steroid used as a growth promoter in cattle and a contaminant of concern in aquatic systems. Our objectives were to investigate the gene expression profile induced by TB, define biomarkers of exposure to TB, and increase our understanding of the mechanisms of adverse effects of TB on fish reproduction. In female gonad tissue, microarray analysis using a 22K oligonucleotide microarray (EcoArray Inc., Gainesville, FL) showed 99 significantly upregulated genes and 741 significantly downregulated genes in response to 1microg TB/L. In particular, hydroxysteroid (17beta) dehydrogenase 12a (hsd17b12a), zona pellucida glycoprotein 2.2 (zp2.2), and protein inhibitor of activated STAT, 2 (pias2) were all downregulated in gonad. Q-PCR measurements in a larger sample set were consistent with the microarray results in the direction and magnitude of these changes in gene expression. However, several novel potential biomarkers were verified by Q-PCR in the same samples, but could not be validated in independent samples. In liver, Q-PCR measurements showed a significant decrease in vitellogenin 1 (vtg1) mRNA expression. In brain, cytochrome P450, family 19, subfamily A, polypeptide 1b (cyp19a1b, previously known as aromatase B) transcript levels were significantly reduced following TB exposure. Our study provides a candidate gene involved in mediating the action of TB, hsd17b12a, and two potential biomarkers sensitive to acute TB exposure, hepatic vtg1 and brain cyp19a1b | Fish EDC | Limited, high -dose, acute | Yes, mechanistic data |

| | | | | | | |
|---|---|---|---|---|---|---|
| Effects of 17 alpha-trenbolone and melengestrol acetate on Xenopus laevis growth, development, and survival | Finch, BE \| Blackwell, BR \| Faust, DR \| Wooten, KJ \| Maul, JD \| Cox, SB \| Smith, PN | ENVIRON MENTAL SCIENCE AND POLLUTIO N RESEARCH \| 20 (2): 1151-1160 FEB 2013 | The synthetic growth-promoting hormones trenbolone and melengestrol acetate have been detected in the environment near beef cattle feedlots and are reportedly transported via wind-borne particulate matter. Therefore, movement of synthetic hormones from beef cattle feedlots to water bodies via particulate matter is possible. Our objective was to evaluate potential effects of 17 alpha-trenbolone (17 alpha-TB), melengestrol acetate (MGA), and combinations of both on growth, development, and survival of Xenopus laevis larvae. On post-hatch day 2 (stage 33/34), X. laevis larvae were exposed to three nominal concentrations of 17 alpha-TB (10, 100, and 500 ng/L), MGA (1, 10, and 100 ng/L), a combination of both (1/10, 10/100, and 100/500 ng/L MGA/17 alpha-TB), frog embryo teratogenesis assay-Xenopus medium, or a solvent control. Significant increases in all X. laevis growth metrics were observed among larvae in the 1 ng/L MGA + 10 ng/L 17 alpha-TB and 10 ng/L MGA + 100 ng/L 17 alpha-TB treatments. Stage of development was increased among larvae in the 1 ng/L MGA + 10 ng/L 17 alpha-TB treatment group and significantly decreased among those in the 500 ng/L 17 alpha-TB treatment. Total body mass and snout-vent length of X. laevis larvae were significantly reduced in the 100 ng/L MGA and 100 ng/L MGA + 500 ng/L 17 alpha-TB treatment groups. Larvae exposed to 500 ng/L 17 alpha-TB had decreased total body mass, snout-vent length, and total length. In general, growth measurements decreased with increasing concentration of MGA, 17 alpha-TB, or a combination of both. Survival among all treatments was not significantly different from controls. Amphibians exposed to MGA and 17 alpha-TB in the environment may experience alterations in growth and development. | Amphi bian EDC Study | Limited, just short-term probably fairly insensitive endpoints | Yes |
| Real-time PCR-based prediction of gonad phenotype in medaka | Flynn, K \| Haasch, M \| Shadwick, DS \| Johnson, R | ECOTOX & ENVIRON SAFETY 73 (4): 589-594 2010 | An important endpoint in aquatic bioassays for potential endocrine disrupting chemicals (EDCs) is the gonadal phenotype of exposed fish, with special interest in intersex and sex-reversed individuals. Traditionally, the assessment of gonad phenotype is done via histology, which involves specialized and time-consuming techniques. The method detailed here increases the efficiency of the analysis by first determining the relative expression of four genes involved in gonad development/maintenance in Japanese medaka (Oryzias latipes), and then by using principal component analysis, assigning a phenotype to each gonad based upon the gene expression data. The gonad phenotype and the sexual genotype, which can be determined in medaka, can then be compared to assess potential adverse effects of exposure to endocrine disrupting chemicals. Published by Elsevier Inc. | Fish EDC | Limited, uses high dose with trenbolone as a "postive" model for method validation | Yes |
| Use of gene expression data to determine effects on gonad phenotype in japanese medaka after exposure to trenbolone or estradiol | Flynn, K \| Swintek, J \| Johnson, R | ENVIRON MENTAL TOXICOLO GY AND CHEMISTR Y \| 32 (6): 1344-1353 JUN 2013 | Various aquatic bioassays using one of several fish species have been developed or are in the process of being developed by organizations like the US Environmental Protection Agency and the Office of Economic Cooperation and Development for testing potential endocrine-disrupting chemicals (EDCs). Often, these involve assessment of the gonad phenotype of individuals as a key endpoint that is inputted into a risk or hazard assessment. Typically, gonad phenotype is determined histologically, which involves specialized and time-consuming techniques. The methods detailed here utilize an entirely different methodology, reverse-transcription quantitative polymerase chain reaction, to determine the relative expression levels of 4 genes after exposure to either 17-estradiol or 17-trenbolone and, by extension, the effects of EDCs on the phenotypic status of the gonad. The 4 genes quantified, Sox9b, protamine, Fig1, and ZPC1, are all involved in gonad development and maintenance in Japanese medaka (Oryzias latipes); these data were then inputted into a permutational multivariate analysis of variance to determine whether significant differences exist between treatment groups. This information in conjunction with the sexual genotype, which can be determined in medaka, can be used to determine adverse effects of exposure to EDCs in a similar fashion to the histologically determined gonad phenotype. | Fish EDC | Limited, uses high dose with trenbolone as a "postive" model for method validation | Yes |

| | | | | | | |
|---|---|---|---|---|---|---|
| Trenbolone acetate metabolites promote ovarian growth and development in adult Japanese medaka (Oryzias latipes) | Forsgren, KL \| Qu, S \| Lavado, R \| Cwiertny, D \| Schlenk, D | GENERAL AND COMPARATIVE ENDOCRINOLOGY \| 202: 1-7 JUN 1 2014 | Trenbolone acetate, a synthetic androgen, has been used as a growth promoter in beef cattle in the US since 1987. While several teleost studies have investigated the masculinization effects of the metabolite 17 beta-trenbolone, few have focused on the reproductive impacts of all three trenbolone acetate (TBA) metabolites including trendione. Adult female medaka (Oryzias latipes) were exposed to TBA metabolites (10, 100, and 1000 ng/L) for 14 days (n = 3). Histological examination revealed that TBA metabolites (1000 ng/L) significantly reduced the percentage of primary ovarian follicles and increased the percentage of vitellogenic follicles compared to control fish. 17 alpha-Trenbolone significantly increased whereas trendione reduced whole body levels of estradiol-17 beta. Testosterone was significantly reduced by trendione treatment and only the highest dose of 17 beta-trenbolone and lowest dose of trendione altered 11-ketotestosterone. Additionally, TBA metabolites may be further broken down and/or metabolized or converted by the animal influencing both sex steroid levels and ovarian development. | Fish EDC | High | Yes |
| Gene expression patterns in rainbow trout, Oncorhynchus mykiss, exposed to a suite of model toxicants | Hook, SE \| Skillman, AD \| Small, JA \| Schultz, IR | AQUAT TOXICOL \| 77 (4): 372-385 MAY 25 2006 | The increased availability and use of DNA microarrays has allowed the characterization of gene expression patterns associated with exposure to different toxicants. An important question is whether toxicant induced changes in gene expression in fish are sufficiently diverse to allow for identification of specific modes of action and/or specific contaminants. In theory, each class of toxicant may generate a gene expression profile unique to its mode of toxic action. In this study, isogenic (cloned) rainbow trout Oncorhynchus mykiss were exposed to sublethal levels of a series of model toxicants with varying modes of action, including ethynylestradiol. (xeno-estrogen), 2,2,4,4'-tetrabrornodiphenyl ether (BDE-47, thyroid active), diquat (oxidant stressor), chromium VI, and benzo[a]pyrene (BaP) for a period of 1-3 weeks. An additional experiment measured trenbolone (anabolic steroid;'I model androgen) induced gene expression changes in sexually mature female trout. Following exposure, fish were euthanized, livers removed and RNA extracted. Fluorescently labeled cDNA were generated and hybridized against a commercially available Atlantic Salmon/Trout array (GRASP project, University of Victoria) spotted with 16,000 cDNA's. The slides were scanned to measure abundance of a given transcript in each sample relative to controls. Data were analyzed via Genespring (Silicon Genetics) to identify a list of up- and downregulated genes, as well as to determine gene clustering patterns that can be used as ""expression signatures"". The results indicate each toxicant exposure caused between 64 and 222 genes to be significantly altered in expression. Most genes exhibiting altered expression responded to only one of the toxicants and relatively few were co-expressed in multiple treatments. For example, BaP and Diquat, both of which exert toxicity via oxidative stress, upregulated 28 of the same genes, of over 100 genes altered by either treatment. Other genes associated with steroidogenesis, p450 and estrogen responsive genes appear to be useful for selectively identifying toxicant mode of action in fish, suggesting a link between gene expression profile and mode of toxicity. Our array results showed good agreement with quantitative real time polymerase chain reaction (qRT PCR), which demonstrates that the arrays are an accurate measure of gene expression. The specificity of the gene expression profile in response to a model toxicant, the link between genes with altered expression and mode of toxic action, and the consistency between array and qRT PCR results all suggest that cDNA microarrays have the potential to screen environmental contaminants for biomarkers and mode of toxic action. | Fish EDC | Limited, largely just gene expression | Yes, mechanistic work |

| | | | | | | |
|---|---|---|---|---|---|---|
| Developmental reproductive effects of exposure to pharmaceutical steroids in the aquatic environment: Studies on mosquitofish (Gambusia affinis affinis), roach (Rutilus rutilus) and medaka (Oryzias latipes) | Iguchi, T \| Katsu, Y \| Urushitani, H \| Lange, A \| Tyler, CR | JOURNAL OF MARINE SCIENCE AND TECHNOLOGY-TAIWAN \| 15: 29-36 Sp. Iss. SI AUG 2007 | Pharmaceutical androgens and estrogens discharged into the aquatic environment are now known to induce adverse effects in fish and are a health concern for wildlife. Mosquitofish (Gambusia affinis) exposed to a pharmaceutical androgen, trenbolone, used to enhance cattle growth and found to pollute waters below feedlots in the USA, has been shown to alter the development of the anal fin of the females and disrupt gonopodium development in fry. In our work, we showed that altered gonopodimu development was associated with disruption in the normal patterns of expression of 2 cloned androgen receptors. Furthermore, exposure to trenbolone at 1 mu g/L induced spermatocytes in the ovary of sexually mature females. Roach (Rutilus rutilus) living in UK rivers are exposed to estrogenic chemicals in effluents derived from sewage treatment works and this causes feminizing effects, including the development of oocytes in the testis of males. The contraceptive estrogen ethinylestradiol (EE2) is believed to contribute to these feminized responses. Our lab-based studies showed that gonadal feminization of roach could be induced by exposure to EE2 at 4 ng/L and the phenotypic responses were associated with altered patterns of expression of 2 cloned estrogen receptors (ERs) and aromatase genes. EE2 was shown to induce similar feminized responses in the medaka (Oryzias latipes). We established a reporter gene assay system for roach and medaka ERs and showed that specific environmental estrogens differentially activated the two fish ER subtypes. We also found evidence for an enhanced sensitivity for some estrogens to activate the medaka ERs compared with the roach ERs. | Fish EDC | Limited, high doses | Yes, mechanistic and apical dat |
| Characteristics of ChgH-GFP transgenic medaka lines, an in vivo estrogenic compound detection system | Kurauchi, K \| Hirata, T \| Kinoshita, M | MARIN POLLUT BULLETIN \| 57 (6-12): 441-444 2008 | We previously reported the characteristics of a ChgH-GFP transgenic medaka line that indicates estrogenic compound pollution in environmental water by the green fluorescence of their liver. Recently, we established four more lines. In this study, the characteristics of the five transgenic medaka lines were investigated. The intensity of reporter gene expression varied among transgenic lines and generally correlated well with the amount of integrated transgene in each line. Line-specific ectopic expression was also observed. However, the sensitivity to 17-beta estradiol did not differ among transgenic lines. Three transgenic lines are considered to be suitable as bio-indicators of estrogenic activity, due to the ease of observing green fluorescence in their livers. The transgenic lines can also detect the estrogenic activity of testosterone and 17-beta trenbolone at the nominal concentration of 30 and 100 mu g/l, respectively. | Fish EDC | Limited, in vitro assay development | Yes |
| Low concentrations of 17β-trenbolone induce female-to-male reversal and mortality in the frog Pelophylax nigromaculatus | Li YY;Xu W;Chen XR;Lou QQ;Wei WJ;Qin ZF; | Aquat Toxicol %2015 , Jan | Trenbolone, as a growth promoter in animal agriculture, has become an environmental androgen in surface water. Here, we aimed to reveal the effects of 17β-trenbolone on survival, growth, and gonadal differentiation in the frog Pelophylax nigromaculatus, which is widespread in East Asia and undergoing population decline. P. nigromaculatus tadpoles were exposed to 17β-trenbolone (0.1, 1, 10 μg/L) from Gosner stage 24/25 to complete metamorphosis. We found that 17β-trenbolone resulted in significantly high mortality in a concentration-dependent manner, with a decrease in body weight in the high concentration group compared with the solvent control. Based on gross gonadal morphology, no females were observed, instead of about 15% ambiguous sexes and 85% males, in all 17β-trenbolone treatment groups. Like normal testes, the gonads with sex-ambiguous morphology exhibited testicular histology, showing that the sex-ambiguous gonads were incomplete ovary-to-testis reversals (IOTTRs) with certain ovarian morphological features. In the IOTTRs, the transcriptional levels of ovary-biased genes decreased drastically relative to normal ovaries, and even declined to the levels in normal testes. These observations confirmed that all test concentrations of 17β-trenbolone resulted in 100% sex reversal, although some sex-reversed testes retained some ovarian characteristics at the morphological level. To our knowledge, this is the first report strongly demonstrating that trenbolone can cause female-to-male reversal in amphibians. Given that the lowest concentration tested is environmentally relevant, our study highlights the risks of trenbolone and other environmental androgens for P. nigromaculatus and other amphibians, in particular the species with high sensitivity of gonadal differentiation to androgenic chemicals | Amphibian EDC Study | High (although effects occurred at relatively high concentrations) | Yes |

| | | | | | | |
|---|---|---|---|---|---|---|
| Exploring Androgen-Regulated Pathways in Teleost Fish Using Transcriptomics and Proteomics | Martyniuk, CJ \| Denslow, ND | INTEGRATIVE AND COMPARATIVE BIOLOGY \| 52 (5): 695-704 NOV 2012 | In the environment, there are aquatic pollutants that disrupt androgen signaling in fish. Laboratory and field-based experiments have utilized omics technologies to characterize the molecular mechanisms underlying androgen-receptor agonism/antagonism. Transcriptomics and proteomics studies with 17 beta-trenbolone, a growth-promoting pharmaceutical found in water systems surrounding cattle feed lots, and androgens such as 17 alpha-methyltestosterone and 17 alpha-methyldihydrotestosterone, have been conducted in ovary and liver of fish that include the fathead minnow (FHM) (Pimephales promelas), common carp (Cyprinus carpio), Qurt medaka (Oryzias latipes), and zebrafish (Danio rerio). In this mini-review, we survey recent omics studies in fish and reveal that, despite the diversity of species and tissues examined, there are common cellular responses that are observed with waterborne androgenic treatments. Recurring themes in gene ontology include apoptosis, transport and oxidation of lipids, synthesis and transport of hormones, immune response, protein metabolism, and cell proliferation. However, we also discuss other mechanisms other than androgen receptor (AR) activation, such as responses to toxicant stress, estrogen receptor agonism, aromatization of androgens into estrogens, and inhibitory feedback mechanisms by high levels of androgens that may also explain molecular responses in fish. To further explore androgen-responsive protein networks, a sub-network enrichment analysis was performed on protein data collected from the livers of female FHMs exposed to 17 beta-trenbolone. We construct a putative AR-regulated protein/cell process network in the liver that includes B-lymphocyte differentiation, xenobiotic clearance, low-density lipoprotein oxidation, proliferation of smooth muscle cells, and permeability of blood vessels. We demonstrate that construction of protein networks can offer insight into cell processes that are potentially regulated by androgens. | Fish EDC | High | Yes, review |
| Trenbolone causes mortality and altered sexual differentiation in Xenopus tropicalis during larval development | Olmstead, AW Kosian, PA Johnson, R Blackshear, PE Haselman, J Blanksma, C Korte, JJ Holcombe, GW Burgess, E Lindberg-Livingston, A Bennett, BA Woodis, KK Degitz, SJ | ENVIRONMENTAL TOXICOLOGY AND CHEMISTRY \| 31 (10): 2391-2398 OCT 2012 | Trenbolone is an androgen agonist used in cattle production and has been measured in aquatic systems associated with concentrated animal-feeding operations. In this study, the authors characterized the effects of aqueous exposure to 17 beta-trenbolone during larval Xenopus tropicalis development. Trenbolone exposure resulted in increased mortality of post-NieuwkoopFaber stage 58 tadpoles at concentrations =100?ng/L. Morphological observations and the timing of this mortality are consistent with hypertrophy of the larynx. Development of nuptial pads, a male secondary sex characteristic, was induced in tadpoles of both sexes at 100?ng/L. Effects on time to complete metamorphosis or body sizes were not observed; however, grow-outs placed in clean media for six weeks were significantly smaller in body size at 78?ng/L. Effects on sex ratios were equivocal, with the first experiment showing a significant shift in sex ratio toward males at 78?ng/L. In the second experiment, no significant effects were observed up to 100?ng/L, although overall sex ratios were similar. Histological assessment of gonads at metamorphosis showed half with normal male phenotypes and half that possessed a mixed-sex phenotype at 100?ng/L. Hypertrophy of the Wolffian ducts was also observed at this concentration. These results indicate that larval 17 beta-trenbolone exposure results in effects down to 78?ng/L, illustrating potential effects from exposure to androgenic compounds in anurans. | Amphibian EDC Study | High, mechanistic and apical endpoints | Yes |
| Comparison of vitellogenin induction, sex ratio, and gonad morphology between zebrafish and Japanese medaka after exposure to | Örn S;Yamani S;Norrgren L; | Arch Environ Contam Toxicol %2006 , Aug | The pharmaceutical estrogen 17alpha-ethinylestradiol (EE2) and the anabolic androgen 17beta-trenbolone (Tb) can interfere with the endocrine and reproductive systems of fish. The potency of these chemicals in zebrafish (Danio rerio) and Japanese medaka (Oryzias latipes) was assessed using the core end points vitellogenin (Vtg) concentration at 38 days post-hatch and sex ratio and gonad morphology at 60 days post-hatch. Vtg concentrations were measured in fish whole-body homogenate samples using enzyme-linked immunosorbent assay. Increased Vtg concentration and feminization of fish after exposure to 10 ng/L EE2, as well as masculinization after exposure to 50 ng/L of Tb, were observed in zebrafish. Intersex was observed in medaka exposed to EE2. A decrease in Vtg production after Tb exposure (50 ng/L) was measured in both zebrafish and medaka. Analyses of gonad morphology revealed increased testicular area and sperm percentage in Tb-exposed zebrafish, whereas increased sperm percentage was measured | Fish EDC | High | Yes |

| | | | | | | |
|---|---|---|---|---|---|---|
| 17alpha-ethinylestradiol and 17beta-trenbolone | | | in Tb-exposed medaka. The higher sensitivity of zebrafish compared with medaka to both EE2 and Tb was revealed in the study | | | |
| Advanced fluorescence in situ hybridization to localize and quantify gene expression in Japanese medaka (Oryzias latipes) exposed to endocrine-disrupting compounds | Park JW;Tompsett AR;Zhang X;Newsted JL;Jones PD;Au DW;Kong R;Wu RS;Giesy JP;Hecker M; | Environ Toxicol Chem %2009 , Sep | In an earlier study, we described the development of fluorescence in situ hybridization (FISH) using confocal microscopy to localize and quantify gene expression in fish. Here, we report the results of FISH application to investigate effects of model endocrine-disrupting chemicals (EDCs), 17alpha-ethinylestradiol (EE2) and 17beta-trenbolone (TB), on expressions of EDC-responsive genes in Japanese medaka (Oryzias latipes) at the cellular/tissue level paired with histological observation. Gene expressions of vitellogenin-II (Vit-II), androgen receptor (AR), and cytochrome P450 gonadal aromatase (CYP19a) were determined after exposure to 5, 50, or 500 ng/L of EE2 or 50, 500, or 5,000 ng/L of TB for 7 d. Exposure to the greatest concentration of EE2 or TB significantly reduced fecundity and caused histological alterations in gonads. 17alpha-Ethinylestradiol induced Vit-II expression in both male gonads and liver relative to controls and resulted in greater intensity of hematoxylin staining in hepatocytes, which was significantly correlated with Vit-II induction in liver. When exposed to EE2 at less than 50 ng/L, CYP19a expression associated with early stage oocytes was greater than that in controls. However, at 500 ng/L, this trend was reversed. The greater Vit-II expression in testis from all EE2 groups, and the lesser expression of CYP19a in ovaries from the 500 ng/L group, likely is related to changes in the number of cells in which these genes are predominantly expressed rather than to an increase in expression per cell. 17beta-Trenbolone significantly induced AR expression in ovaries but did not alter AR expression in female liver. It was concluded that FISH combined with histology enables advanced elucidation of molecular effects of chemicals by associating changes in gene expression with certain tissues and/or cell types and allows these changes to be related to histological effects | Fish EDC | Limited, test method devlopment | Yes |
| Gene Expression Profiling In Rainbow Trout (Onorhyncus Mykiss), Exposed To A Variety Of Model Toxicants | Schultz I;Hook S;Skillman AD; | | The increased availability and use of DNA microarrays has allowed the characterization of gene expression patterns associated with different toxicants. An important question is whether toxicant induced changes in gene expression in fish are sufficiently diverse to allow for identification of specific modes of action or specific contaminants. In theory, each class of toxicant may generate a gene expression profile unique to its mode of toxic action. The latter will be influenced by dose, route of administration and developmental state among other potential modifying factors. We exposed isogenic (cloned) rainbow trout Onorhyncus mykiss, to sublethal levels of a series of model toxicants with varying modes of action, including ethynylestradiol (xeno-estrogen), trenbolone (anabolic steroid; model androgen), tetrabromodiphenyl ether (BDE-47, thyroid active), diquat (oxidant stressor), chromium VI, and benzo-a-pyrene (BaP) for a period of 1-3 weeks. Following exposure, fish were euthanized, livers harvested and RNA extracted. Fluorescently labeled cDNA were generated and hybridized against a commercially available Atlantic Salmon / Trout array (GRASP project, University of Victoria) spotted with 16, 000 cDNAs. The slides were scanned to measure abundance of a given transcript in each sample relative to controls. Data were analyzed via Genespring (Silicon Genetics) to identify a list of up and down regulated genes, as well as to determine gene clustering patterns that can be used as expression signatures. Initial analysis indicates each toxicant generated specific gene expression profiles. Most genes exhibiting altered expression responded to only one of the toxicants. Relatively few genes are co-expressed in multiple treatments. For example, BaP and Diquat, both of which exert toxicity via oxidative stress, up-regulated 28 of the same genes. Other genes associated with steroidogenesis, p450 and estrogen responsive genes appear to be useful for selectively identifying toxicant mode of in fish | Fish EDC | Limited, gene expression only | Yes (but unsure of source) |

| | | | | | | |
|---|---|---|---|---|---|---|
| Toxicokinetic, toxicodynamic, and toxicoproteomic aspects of short-term exposure to trenbolone in female fish | Schultz IR;Nagler JJ;Swanson P;Wunschel D;Skillman AD;Burnett V;Smith D;Barry R; | Toxicol Sci %2013 , Dec | The toxicokinetics of trenbolone was characterized during 500 ng/l water exposures in female rainbow trout (Oncorhynchus mykiss) and fathead minnows (Pimephales promelas). Related experiments measured various toxicodynamic effects of exposure. In both species, trenbolone was rapidly absorbed from the water and reached peak plasma levels within 8h of exposure. Afterwards, trenbolone concentrations in trout (66-95 ng/ml) were 2-6 times higher compared with minnows (15-29 ng/ml), which was attributable to greater plasma binding in trout. During water exposures, circulating levels of estradiol (E2) rapidly decreased in both species to a concentration that was 25%-40% of control values by 8-24h of exposure and then remained relatively unchanged for the subsequent 6 days of exposure. In trout, changes in circulating levels of follicle-stimulating hormone were also significantly greater after trenbolone exposure, relative to controls. In both species, the pharmacokinetics of injected E2-d3 was altered by trenbolone exposure with an increase in total body clearance and a corresponding decrease in elimination half-life. The unbound percentage of E2 in trout plasma was 0.25%, which was similar in pre- or postvitellogenic female trout. Subsequent incubation with trenbolone caused the unbound percentage to significantly increase to 2.4% in the previtellogenic trout plasma. iTRAQ-based toxicoproteomic studies in minnows exposed to 5, 50, and 500 ng/l trenbolone identified a total of 148 proteins with 19 downregulated including vitellogenin and 18 upregulated. Other downregulated proteins were fibrinogens, α-2-macroglobulin, and transferrin. Upregulated proteins included amine oxidase, apolipoproteins, parvalbumin, complement system proteins, and several uncharacterized proteins. The results indicate trenbolone exposure is a highly dynamic process in female fish with uptake and tissue equilibrium quickly established, leading to both rapid and delayed toxicodynamic effects | Fish EDC | High, PB-PK and omic data | Yes |
| Comparison of response to 17 beta-estradiol and 17 beta-trenbolone among three small fish species | Seki, M \| Fujishima, S \| Nozaka, T \| Maeda, M \| Kobayashi, K | ENVIRONMENTAL TOXICOLOGY AND CHEMISTRY \| 25 (10): 2742-2752 OCT 2006 | Three small fish species, medaka (Oryzias latipes), fathead minnow (Pimephales promelas), and zebrafish (Danio rerio), were exposed to an estrogen, 17 beta-estradiol (E-2), and an androgen, 17 beta-trenbolone (TB), for 21 d under flow-through conditions to compare the susceptibility among these three small fish species to the substances. Effects on gross morphology, including secondary sex characteristics and gonadosomatic index, as well as on blood or liver vitellogenin (VTG) levels were assessed. In E-2 exposures, significant increases in estrogenic activity were observed in both sexes of all three fish species. The lowest-observed-effect concentrations (LOECs) of E-2 for VTG induction in males of medaka, fathead minnow, and zebrafish were less than or equal to 8.94, 28.6, and 85.9 ng/L, respectively. In TB exposures, we observed masculinization of secondary sex characteristics in females as a result of the androgenic activity of TB in medaka with a LOEC of 365 ng/L and in fathead minnow with a LOEC of 401 ng/L. We also found VTG reduction in females of all three fish species. These results suggest that the susceptibility of medaka to estrogenic chemicals may be higher than those of fathead minnow and zebrafish and that the susceptibility of medaka to androgenic chemicals may be almost equal to that of fathead minnow in the 21-d fish assay. | Fish EDC | High, method validation, but some comparative fish data | Yes |
| The endocrine activity of beef cattle wastes: Do growth-promoting steroids make a difference? | Sellin, MK \| Snow, DD \| Gustafson, ST \| Erickson, GE \| Kolok, AS | AQUAT TOXICOL \| 92 (4): 221-227 MAY 17 2009 | The primary objective of this study was to compare the endocrine activity of wastes from trenbolone acetate: estradiol (TBA:E)-implanted steers to that of wastes from unimplanted steers. To accomplish this, fathead minnows (Pimephales promelas) were exposed to urine or fecal slurry from TBA:E-implanted or unimplanted steers for 7 days. Following exposures, hepatic vitellogenin (vtg) mRNA expression and secondary sexual characteristics were assessed. Among both males and females, there were no differences in vtg mRNA expression between fish exposed to urine from implanted or unimplanted steers at any of the concentrations tested. Furthermore, concentrations of steroid hormones in the urine of implanted and unimplanted steers were similar. These findings indicate a lack of differences in the endocrine activity of urine from TBA:E-implanted and unimplanted steers. With regard to the fecal slurry exposures, there were no significant differences in vtg mRNA expression among females from any of the groups; however, significant differences in male vtg mRNA expression were detected. Specifically, males exposed to 1600 mg dry feces/L from implanted cattle experienced an 840-fold increase in vtg mRNA expression relative to both | Fish EDC | Limited, complex mixture analysis | Yes |

| | | | | | | |
|---|---|---|---|---|---|---|
| | | | unexposed males and males exposed to the corresponding fecal concentration from unimplanted steers. These males also appeared to experience a reduction in male secondary sexual characteristics. These findings suggest that steroids associated with the wastes from TBA:E-implanted steers have both feminizing and demasculinizing effects on male fish. Furthermore, these effects are most likely due to the presence of estrogenic compounds, which were detected in the liquid portion of the fecal slurry from TBA:E-implanted steers, but not in that of unimplanted steers. The findings of this study indicate the presence of endocrine-disrupting compounds in the urine and feces of cattle and suggest that the implant history of cattle alters the endocrine activity of feces, but does not alter the endocrine activity of urine. | | | |
| AGRICHEMICALS IN NEBRASKA, USA, WATERSHEDS: OCCURRENCE AND ENDOCRINE EFFECTS | Sellin, MK \| Snow, DD \| Schwarz, M \| Carter, BJ \| Kolok, AS | ENVIRONMENTAL TOXICOLOGY AND CHEMISTRY \| 28 (11): 2443-2448 2009 | The objective of the present study was to determine the occurrence and endocrine effects of agrichemicals in four Nebraska, USA, watersheds-the Elkhorn, Platte, Niobrara, and Dismal rivers. Land use in the Elkhorn River and Platte River watersheds is characterized by intense agriculture, including row crop and beef cattle production. In contrast, land within the Niobrara River and Dismal River watersheds consists primarily of grasslands. Polar organic chemical integrative samplers (POCIS) and caged fathead minnows were deployed at a site within each watershed for 7 d. The POCIS were analyzed for pesticides and hormones, while the caged minnows were analyzed for the expression of estrogen-and androgen-responsive genes. Amounts of pesticides recovered in POCIS extracts from the Elkhorn and Platte rivers were higher than those recovered from the Niobrara and Dismal rivers. Furthermore, female minnows deployed in the Elkhorn River experienced significant reductions in expression of two estrogen-responsive genes (vitellogenin and estrogen receptor alpha) relative to females deployed at the other sites, indicating alterations in endocrine function. However, the defeminization of these females could not be definitely linked to any of the agrichemicals detected in the POCIS recovered from the Elkhorn River. | Fish EDC | Limited, complex mixture analysis | Yes |
| Real-time PCR array to study effects of chemicals on the Hypothalamic-Pituitary-Gonadal axis of the Japanese medaka | Zhang X;Hecker M;Park JW;Tompsett AR;Newsted J;Nakayama K;Jones PD;Au D;Kong R;Wu RS;Giesy JP; | Aquat Toxicol %2008 , Jul 7 | This paper describes the development and validation of a PCR array for studying chemical-induced effects on gene expression of selected endocrine pathways along the hypothalamic-pituitary-gonadal (HPG) axis of the small, oviparous fish, the Japanese medaka (Oryzias latipes). The Japanese medaka HPG-PCR array combines the quantitative performance of SYBR Green-based real-time PCR with the multiple gene profiling capabilities of a microarray to examine expression profiles of 36 genes associated with endocrine pathways in brain, liver and gonad. The performance of the Japanese medaka HPG-PCR array was evaluated by examining effects of two model compounds, the synthetic estrogen, 17alpha-ethinylestradiol (EE2) and the anabolic androgen, 17beta-trenbolone (TRB) on the HPG axis of the Japanese medaka. Four-month-old medaka was exposed to three concentrations of EE2 (5, 50, 500 ng/L) or TRB (50, 500, 5000 ng/L) for 7d in a static renewal exposure system. A pathway-based approach was implemented to analyze and visualize concentration-dependent mRNA expression in the HPG axis of Japanese medaka. The compensatory response to EE2 exposure included the down-regulation of male brain GnRH RI and testicular CYP17. The down-regulation of AR-alpha expression in brain of EE2-exposed males was associated with suppression of male sexual behavior. Compensatory responses to TRB in the female HPG axis included up-regulation of brain GnRH RII and ovary steroidogenic CYP19A. Overall, the results suggested that the Japanese medaka HPG-PCR array has potential not only as a screening tool of potential endocrine-disrupting chemicals but also in elucidating mechanisms of action | Fish EDC | Limited, gene expression only, but good time/dose data | Yes |
| TIME-DEPENDENT TRANSCRIPTIONAL PROFILES OF GENES OF THE | Zhang, XW \| Hecker, M \| Park, JW \| Tompsett, AR \| Jones, PD \| | ENVIRONMENTAL TOXICOLOGY AND CHEMISTR | Both the anabolic androgen 17 beta-trenbolone (TRB) and the aromatase inhibitor fadrozole ( FAD) can cause decreased plasma concentrations of estrogen (E2) and reduce fecundity of fish. However, the underlying mechanisms and the molecular pathways involved are largely unknown. The present study was designed to assess time-dependent effects of FAD and TRB on the transcriptional responses of the hypothalamic-pituitary-gonadal (HPG) axis of Japanese medaka ( Oryzias latipes). Fourteen-week-old Japanese medaka were exposed to 50 mu g | Fish EDC | Limited, gene expression only, but good time/dose | Yes |

| | | | | | | |
|---|---|---|---|---|---|---|
| HYPOTHALAMIC-PITUITARY-GONADAL AXIS IN MEDAKA (ORYZIAS LATIPES) EXPOSED TO FADROZOLE AND 17 beta-TRENBOLONE | Newsted, J \| Au, DWT \| Kong, R \| Wu, RSS \| Giesy, JP | Y \| 27 (12): 2504-2511 DEC 2008 | FAD/L or 2 mu g TRB/L in a 7-d static renewal test, and the expression profiles of 36 HPG axis genes were measured by means of a medaka HPG real-time reverse-transcription polymerase chain reaction array after 8 h, 32 h, or 7 d of exposure. Exposure to TRB or FAD caused lesser fecundity of Japanese medaka and down-regulated transcription of vitellogenin and choriogenin (CHG) gene expression in the liver of females. Exposure to FAD for 8 h resulted in an 8-fold and 71-fold down-regulation of expression of estrogen receptor alpha and choriogenin L ( CHG L), respectively, in female liver. 17 beta-Trenbolone caused similar down-regulation of these genes, but the effects were not observed until 32 h of exposure. These results support the hypothesis that FAD reduces plasma E2 more quickly by inhibiting aromatase enzyme activity than does TRB, which inhibits the production of the E2 precursor testosterone. Exposure to FAD and TRB resulted in rapid ( after 8 h) down-regulation of luteinizing hormone receptor and low-density-lipoprotein receptor in the testis to compensate for excessive androgen levels. Overall, the molecular responses observed in the present study differentiate the mechanisms of the reduced fecundity by TRB and FAD. | | data | |
| Paradoxes in exogenous androgen treatments of bluegill | Al-Ablani, SA \| Phelps, RP | JOURNAL APPL ICHTHYOL 18 (1): 61-64 2002 | Three experiments were conducted to investigate the effect of oral administration of trenbolone acetate and 17a-methyltestosterone on sex determination in 28-day-old bluegill, Lepomis macrochirus Rafinesque, fry. Multiple androgen doses and various treatment durations were tested. All treatments produced fewer mates and females than did the control group (P < 0.01). All concentrations of both androgens produced a high proportion of intersex fish (38-81%). The number of males and females declined with the increase in androgen dose or treatment duration, Sterile fish were found in treatments with a higher dose rate or a longer treatment period, The predominance of intersex fish and reductions of both males and females in the androgen treatments suggest that gonadal development of both genotypic male and female fish were being altered by the hormone treatment. | Aquaculture Application Study | Limited, demonstrates endocrine activity but oral dose problematic | Yes, species sensitivity |
| Intercalibration exercise using a stickleback endocrine disrupter screening assay | Allen, YT \| Katsiadaki, I \| Pottinger, TG \| Jolly, C \| Matthiessen, P \| Mayer, I \| Smith, A \| Scott, AP \| Eccles, P \| Sanders, MB \| Pulman, KGT \| Feist, S | ENVIRONMENTAL TOXICOLOGY AND CHEMISTRY \| 27 (2): 404-412 FEB 2008 | The Organisation for Economic Cooperation and Development (OECD) is currently validating a short-term fish screening protocol for endocrine disrupters (estrogens, androgens, and their antagonists and aromatase inhibitors), using three core species: fathead minnow, Japanese medaka, and zebrafish. The main endpoints proposed for the first phase of validation of the screen are vitellogenin (VTG) concentration, gross morphology (secondary sexual characteristics and gonado-somatic index), and gonadal histopathology, A similar protocol is concurrently being developed in the United Kingdom using the three-spined stickleback, with identical endpoints to those for the core species and, in addition, a unique androgen-specific endpoint in the form of spiggin (glue protein) induction. To assess the suitability of this species for inclusion in the OECD protocol alongside the core species, an intercalibration was conducted using 17 beta-estradiol (a natural estrogen) and trenbolone (a synthetic androgen), thus mimicking a previous intercalibration with the core species. All three participating laboratories detected statistically significant increases in VTG in males after 14 d exposure to nominal concentrations of 100 ng/L 17 beta-estradiol and statistically significant increases in spiggin in females after 14 d exposure to nominal concentrations of 5,000 ng/L trenbolone. The stickleback screen is reliable, possessing both relevant and reproducible endpoints for the detection of potent estrogens and androgens. Further work is underway to assess the relevance and suitability of the screen for weakly acting estrogens, anti-androgens, and aromatase inhibitors. | Fish EDC | Limited, uses high dose with trenbolone as a "postive" model for method validation | Yes, species sensitivity |

| | | | | | | |
|---|---|---|---|---|---|---|
| Production of monosex male black crappie, Pomoxis nigromaculatus, populations by multiple androgen immersion | Arslan, T \| Phelps, RP | AQUACULTURE \| 234 (1-4): 561-573 MAY 3 2004 | Monosex populations can solve the problem of stunted black crappie populations in small impoundments and can increase the potential of black crappie as an aquaculture species. In this study, we investigated the effective mode and duration of exogenous androgen administration to produce monosex male black crappie populations. We conducted two experiments using the synthetic androgens, 17alpha-methyltestosterone (MT) and trenbolone acetate (TBA). In the first experiment, the same age (45 days old), two different size fry with mean total lengths ( +/- S.D., N = 50) of 20.1 +/- 1.4 and 26.1 +/- 2.0 mm were either fed 60 mg MT/kg diet for 45 days or immersed in a 1 mg MT/l solution on 10 occasions for 5 h/day every 3-5 days between 45 and 86 days post-hatch (dph). In the second experiment, a different cohort of fry was immersed in a 1 mg TBA/ 1 solution for 5 h/day every 3-5 days either on seven occasions between 40 and 66 dph or on 10 occasions between 45 and 86 dph. Mean total lengths ( +/-S.D., N = 50) of fry at 40 and 45 dph were 20.3 +/- 0.9 and 21.6 +/- 1.2 mm, respectively. Both modes of MT administration were ineffective for altering sex ratios in larger black crappie fry. Oral administration of MT to smaller fry resulted in 23% intersex fish. MT administration via short periodic baths of smaller fry produced 96% male populations. TBA was as potent as MT to induce masculinization in black crappie: both immersion treatments produced all male populations. Results of the present study indicated that initial size of fry as well as initial age should be considered when selecting appropriate size black crappie fry for hormone treatments. Monosex male populations in black crappie can effectively be produced through a series of (seven or less) short (5 h) immersions of young fry (40 - 45 days old and 20 - 21 mm) in a 1 mg/l aqueous solution of MT or TBA. Besides high effectiveness, immersion method will be the preferred mode of androgen administration to black crappie because it reduces variation in hormone uptake associated with the oral administration of steroids and provides more flexibility in feeding during the period of gonadal differentiation. | Aquaculture Application Study | Limited, doses very high | Yes, species sensitivity |
| Detection of immunotoxic effects of estrogenic and androgenic endocrine disrupting compounds using splenic immune cells of the female three-spined stickleback, Gasterosteus aculeatus (L.) | Bado-Nilles A;Techer R;Porcher JM;Geffard A;Gagnaire B;Betoulle S;Sanchez W; | Environ Toxicol Pharmacol %2014 , Sep | Today, the list of endocrine disrupting compounds (EDCs) in freshwater and marine environments that mimic or block endogenous hormones is expanding at an alarming rate. As immune and reproductive systems may interact in a bidirectional way, some authors proposed the immune capacities as attractive markers to evaluate the hormonal potential of environmental samples. Thus, the present work proposed to gain more knowledge on direct biological effects of natural and EDCs on female fish splenic leucocyte non-specific immune activities by using ex vivo assays. After determining the optimal required conditions to analyze splenic immune responses, seven different EDCs were tested ex vivo at 0.01, 1 and 100nM over 12h on the leucocyte functions of female three-spined stickleback, Gasterosteus aculeatus. In summary, we found that natural hormones acted as immunostimulants, whilst EDCs were immunosuppressive | Fish EDC | Limited, little apical information | Yes, novel endpoints |

| | | | | | | |
|---|---|---|---|---|---|---|
| Differential ligand selectivity of androgen receptors alpha and beta from Murray-Darling rainbowfish (Melanotaenia fluviatilis) | Bain, PA \| Ogino, Y \| Miyagawa, S \| Iguchi, T \| Kumar, A | GENERAL AND COMPARATIVE ENDOCRINOLOGY \| 212: 84-91 FEB 1 2015 | Androgen receptors (ARs) mediate the physiological effects of androgens in vertebrates. In fishes, AR-mediated pathways can be modulated by aquatic contaminants, resulting in the masculinisation of female fish or diminished secondary sex characteristics in males. The Murray-Darling rainbowfish (Melanotaenia fluviatilis) is a small-bodied freshwater teleost used in Australia as a test species for environmental toxicology research. We determined concentration-response profiles for selected agonists and antagonists of rainbowfish AR alpha and AR beta using transient transactivation assays. For both AR alpha and AR beta, the order of potency of natural agonists was 11-ketotestosterone (11-KT) > 5 alpha-dihydrotestosterone > testosterone > androstenedione. Methyltestosterone was a highly potent agonist of both receptors relative to 11-KT. The relative potency of the veterinary growth-promoting androgen, 17 beta-trenbolone, varied by more than a factor of 5 between AR alpha and AR beta. The non-steroidal anti-androgen bicalutamide exhibited high inhibitory potency relative to the structurally related model anti-androgen, flutamide. The inhibitory potency of the agricultural fungicide, vinclozolin, was approximately 1.7-fold relative to flutamide for AR alpha, but over 20-fold in the case of AR beta. Fluorescent protein tagging of ARs showed that the rainbowfish AR alpha subtype is constitutively localised to the nucleus, while AR beta is cytoplasmic in the absence of ligand, an observation which agrees with the reported subcellular localisation of AR subtypes from other teleost species. Collectively, these data suggest that M. fluviatilis AR alpha and AR beta respond differently to environmental AR modulators and that in vivo sensitivity to contaminants may depend on the tissue distribution of the AR subtypes at the time of exposure. | Fish EDC | High | Yes, mechanism of action |
| Ultrasound enhanced immersion protocols for masculinization of Nile tilapia, Oreochromis niloticus | Bart, AN \| Athauda, ARSB \| Fitzpatrick, MS \| Contreras-Sanchez, WM | JOURNAL OF THE WORLD AQUACULTURE SOCIETY \| 34 (2): 210-216 JUN 2003 | Androgen immersion protocols have been unsuccessful in consistently producing all-male tilapia at a high enough ratio for them to be commercially viable. This study explored the use of ultrasound to improve on the results of previous immersion studies. Variables tested include two hormones (trenbolone acetate-TBA and 17alpha-methyldihydrotestoterone-MDHT) at two concentrations (100 and 250 mug/L) and with or without ultrasound (cavitation level). All hormone treatments with ultrasound and non-ultrasound resulted in significantly higher masculinization than the appropriate controls ($P < 0.05$). Among pairs of treatments of the same hormone at the same dose, all ultrasound treatments resulted in significantly higher number of males compared with non-ultrasound treatments with the exception of MDHT 250 mug/L ($P < 0.05$). Comparing across all ultrasound treatments, TBA 250 mug/L with ultrasound had higher masculinization than all the other ultrasound treatments ($P < 0.05$). Comparing across all non-ultrasound treatments, TBA 250 mug/L had higher ($P < 0.05$) masculinization than MDHT 100 mug/L and TBA 100 mug/L with nonultrasound. Two of the three replicates of TBA 250 mug/L ultrasound treatment resulted in 100% males and the highest mean percentage (98%) of males. This study thus demonstrated the potential of a short-term immersion protocol using ultrasound to more predictably produce all-male, commercially viable tilapia seed. | Aquaculture Application Study | Limited, only high doses | Yes, species sensitivity |

| | | | | | | |
|---|---|---|---|---|---|---|
| Sex in troubled waters: Widespread agricultural contaminant disrupts reproductive behaviour in fish | Bertram MG;Saaristo M;Baumgartner JB;Johnstone CP;Allinson M;Allinson G;Wong BB; | Horm Behav %2015 , Apr | Chemical pollution is a pervasive and insidious agent of environmental change. One class of chemical pollutant threatening ecosystems globally is the endocrine disrupting chemicals (EDCs). The capacity of EDCs to disrupt development and reproduction is well established, but their effects on behaviour have received far less attention. Here, we investigate the impact of a widespread androgenic EDC on reproductive behaviour in the guppy, Poecilia reticulata. We found that short-term exposure of male guppies to an environmentally relevant concentration of 17β-trenbolone-a common environmental pollutant associated with livestock production-influenced the amount of male courtship and forced copulatory behaviour (sneaking) performed toward females, as well as the receptivity of females toward exposed males. Exposure to 17β-trenbolone was also associated with greater male mass. However, no effect of female exposure to 17β-trenbolone was detected on female reproductive behaviour, indicating sex-specific vulnerability at this dosage. Our study is the first to show altered male reproductive behaviour following exposure to an environmentally realistic concentration of 17β-trenbolone, demonstrating the possibility of widespread disruption of mating systems of aquatic organisms by common agricultural contaminants | Fish EDC | High | Yes, behavioral endpoints |
| Effects of 17 beta-trenbolone on Eastern and Western mosquitofish (Gambusia holbrooki and G-affinis) anal fin growth and gene expression patterns | Brockmeier, EK \| Ogino, Y \| Iguchi, T \| Barber, DS \| Denslow, ND | AQUAT TOXICOL \| 128: 163-170 MAR 15 2013 | The Eastern and Western mosquitofish (Gambusia holbrooki and G. affinis) are potential bioindicator organisms for endocrine disruptors. Male mosquitofish have an elongated anal fin (gonopodium) used for internal fertilization whose formation is driven by androgens. Normal female mosquitofish have a normal, rounded anal fin which undergoes elongation into a gonopodium structure when female mosquitofish are exposed to androgenic chemicals. Significant issues with using mosquitofish as a bioindicator include the lack of knowledge on how anal fin growth in females corresponds to endpoints relevant to biological integrity and the lack of information on the molecular pathways that regulate anal fin growth. The objectives of this study were to understand how androgen-induced anal fin elongation relates to changes in endpoints related to the female reproductive system and to understand how anal fin elongation occurs in androgen-exposed female mosquitofish. To achieve these objectives, adult female G. holbrooki were exposed to a vehicle control or one of three doses of the androgen 17 p-trenbolone (TB) at nominal concentrations of 0.1, 1 or 10 mu g TB/L. Anal fin measurements were taken and livers were used for quantitative polymerase chain reaction analysis of vitellogenin (vtg) mRNA expression at multiple time points. 10 mu g TB/L induced anal fin elongation after 7 days of treatment (one-way ANOVA, $p < 0.05$) as did 0.1 and 1 mu g TB/L at later time points (one-way ANOVA, $p < 0.05$). 10 mu g TB/L significantly reduced hepatic vtg gene expression at all time points assessed (one-way ANOVA, $p < 0.05$). There was no correlation between anal fin elongation levels and vtg gene expression (Spearman's rho, $p > 0.05$). In a separate experiment, female G. holbrooki and G. affinis were exposed to the vehicle control or 1 mu g TB/L. Anal fins were used for qualitative gene expression analysis of the genes sonic hedgehog (shh), muscle segment homeobox C (msxC), and fibroblast growth factor receptor 1 (fgfr1) by in situ hybridization. Shh was expressed in the distal tip of the gonopodium while msxC and fgfr1 were more widely expressed along the same anal fin rays during androgen exposure. These data provide insight into the molecular pathways involved in anal fin elongation and pave the way for future work toward developing the mosquitofish into a bioindicator organism for endocrine disruptors. | Fish EDC | High | Yes, mechanistic and toxicity endpoints |

| | | | | | | |
|---|---|---|---|---|---|---|
| Exposure of three generations of the estuarine sheepshead minnow (Cyprinodon variegatus) to the androgen, 17beta-trenbolone: effects on survival, development, and reproduction | Cripe GM;Hemmer BL;Raimondo S;Goodman LR;Kulaw DH; | Environ Toxicol Chem %2010 , Sep | Estimating long-term effects of endocrine-disrupting chemicals on a species is important to assessing the overall risk to the populations. The present study reports the results of a 42-week exposure of estuarine sheepshead minnows (Cyprinodon variegatus) to the androgen, 17beta-trenbolone (Tb) conducted to determine if partial-(F0) or single-generation (F1) fish exposures identify multigenerational (F0-F3) effects of androgens on fish. Adult F0 fish were exposed to 0.007, 0.027, 0.13, 0.87,and 4.1 microg Tb/L, the F1 generation to < or =0.87 microg Tb/L, the F2 fish to < or =0.13 microg Tb/L, and the F3 fish to < or =0.027 microg Tb/L. The highest concentrations with reproducing populations at the end of the F0, F1, and F2 generations were 4.1, 0.87, and 0.027 microg Tb/L, respectively. Reproduction in the F0, F1, and F2 generations was significantly reduced at 0.87, 0.027, and 0.027 microg Tb/L, respectively. Fish were significantly masculinized in the F1 generation exposed to 0.13 microg Tb/L or greater. Female plasma vitellogenin was significantly reduced in F0 fish exposed to > or =0.87 microg Tb/L. Gonadosomatic indices of the F0 and F1 generations were significantly increased at 0.87 and 0.13 microg Tb/L in the F0 and F1 generation, respectively, and were accompanied by ovarian histological changes. Reproduction was the most consistently sensitive measure of androgen effects and, after a life-cycle exposure, the daily reproductive rate predicted concentrations affecting successive generations. The present study provides evidence that a multiple generation exposure of fish to some endocrine-disrupting chemicals can result in developmental and reproductive changes that have a much greater impact on the success of a species than was indicated from shorter term exposures | Fish EDC | High | Yes, mechanistic and apical endpoints |
| Reproductive characteristics of adult channel catfish treated with trenbolone acetate during the phenocritical period of sex differentiation | Davis, KB \| Morrison, J \| Galvez, JI | AQUACULTURE \| 189 (3-4): 351-360 OCT 2 2000 | Channel catfish fry fed for 60 days with 0, 50 or 100 mg/kg trenbolone acetate (TBA) and judged by dissection of fingerlings to be males were grown to sexual maturity in ponds. Body weight and gonadal development were compared when the fish were 18 months old. Trenbolone-treated fish were significantly lighter, shorter and the gonads less developed than control males. Three-year-old fish were visually examined for external sex characteristics, and sampled for gonadal development and plasma hormone concentrations. Gonad weight, GSI and plasma testosterone were significantly higher in control fish than in either of the trenbolone-treated groups. Twenty fish from each treatment group were placed in spawning cages with normal female fish. Five spawns were obtained from each bf the treatment groups; however, all 10 spawns were composed of infertile eggs. TBA interferes with normal gonadal development of both the testis and ovary but does, not functionally masculinize channel catfish. | Aquaculture Application Study | Limited, oral dosing | Yes, species sensitivity |
| Effects of progesterone on reproduction and embryonic development in the fathead minnow (Pimephales promelas) | DeQuattro, ZA \| Peissig, EJ \| Antkiewicz, DS \| Lundgren, EJ \| Hedman, CJ \| Hemming, JDC \| Barry, TP | ENVIRONMENTAL TOXICOLOGY AND CHEMISTRY \| 31 (4): 851-856 2012 | High concentrations (375?ng/L) of the steroid hormone progesterone (P4) were measured in snowmelt runoff associated with large livestock-feeding operations in Wisconsin. To gain insight into the potential endocrine-disrupting effects of P4 in fish, experiments were conducted to evaluate the effects of short-term exposure to environmentally relevant concentrations of P4 on reproduction and embryonic development in the fathead minnow (Pimephales promelas). For the reproduction assay, groups of reproductively mature fish were exposed for 21?d to nominal concentrations of 0, 10, 100, and 1,000?ng/L P4 in a flow-through system, and various key reproductive endpoints (e.g., egg number, fertilization success) were quantified throughout the exposure period. The embryonic development assay consisted of incubating fathead minnow eggs in static culture to quantify the effects of P4 on early development and hatching success. Progesterone caused dose-dependent decreases in fecundity and fertility and significantly reduced gonadosomatic index and vitellogenin gene expression in females. There were no effects of P4 on early embryonic development or hatching success. Progesterone may be a significant endocrine-disrupting chemical in fish. | Fish EDC | Limited, doesn't appear to be trenbolone study | Yes, check design |

| | | | | | | |
|---|---|---|---|---|---|---|
| Transgenerational reproductive and endocrine response following exposure to the androgen 17-beta-trenbolone | Foran, CM \| Peterson, B \| Benson, W | MARIN ENVIRON RESEARCH 62: S227-S228 Suppl. S 2006 | Supplementary material on Transgenerational reproductive and endocrine response following exposure to the androgen 17-beta-trenbolone | Fish EDC | Likely low, no abstract | Yes, need to check |
| Androgen Agonists Effects On Estrogenresponsive Plasma Peptide Expression In The Sheepshead Minnow | Hemmer MJ;Salinas KA;Harris PS;Watts J;Dobbins LL;Walker CC; | | Endocrine-disrupting chemicals (EDCs) adversely affect the vertebrate hormone system and cause human and animal health problems. Protein profiling can be used for identification of protein patterns indicative of a change in physiological or toxicological status. These biomarkers can be used diagnostically to screen chemicals for mode of action specific effects. We used Surface Enhanced Laser Desorption/Ionization Time-of-Flight Mass Spectrometry (SELDI-TOF MS) and plasma obtained from sheepshead minnows (Cyprinodon variegatus) to examine estrogen and androgen related changes in protein patterns. Adult male and ovigerous female fish were placed into separate flow-through aquaria for aqueous treatment with 17-beta-estradiol, testosterone, the synthetic androgen, 17-beta-trenbolone, a vehicle control (triethylene glycol, TEG) and seawater control. After a 7 day exposure, plasma was applied to ProteinChip® arrays and analyzed. No significant difference was found between peptide profiles of seawater control and TEG-treated fish. Using pattern recognition software, three peptides were found to be differentially expressed in ovigerous females as compared to unexposed males. An identical expression pattern was observed in male fish exposed to estradiol suggesting the three peptides are estrogen-responsive. The estrogen responsive pattern was also observed in males and females treated with testosterone. However, treatment with trenbolone failed to induce the estrogen responsive peptides in male fish, and in females all three peptides were suppressed with two falling below the level of detection. The differences in the estrogenic pattern elicited by the two androgens examined can be explained by the ready conversion of testosterone to estrogen by P450 aromatase where as the synthetic androgen trenbolone is non-aromatizable. This study illustrates that to properly interpret assay results, one needs to take into account the physiological processes involved which may affect assay specificity | Fish EDC | Fish EDC | Yes |
| Exposure to the pesticide linuron affects androgen-dependent gene expression in the three-spined stickleback (Gasterosteus aculeatus) | Hogan, NS \| Gallant, MJ \| van den Heuvel, MR | ENVIRONMENTAL TOXICOLOGY AND CHEMISTRY \| 31 (6): 1391-1395 JUN 2012 | Previous research demonstrated that exposure to exogenous androgens and effluents with androgenic activity can induce spiggin mRNA production in the kidney of the three-spined stickleback (Gasterosteus aculeatus). In the present study, we determine whether a short-term exposure to a known antiandrogenic pesticide, linuron (LN), suppresses spiggin mRNA in male stickleback and in androgenized female stickleback. Primers were designed from previously characterized sequences for each androgen receptor (AR) isoform in stickleback, ara and ar beta, to assess whether these receptors are differentially regulated by androgen or antiandrogen exposure. Fish were exposed for 72?h to one of four treatments: control, LN (250?mu g/L), 17a-methyltestosterone (MT, 500?ng/L), and an LNMT mixture at those same concentrations. There was no effect of LN on spiggin and ar beta mRNA levels in male kidney, while levels of ara were significantly increased twofold. Exposure to LN significantly inhibited MT-induced spiggin RNA production in female kidney with no effect on expression of ara and ar beta. The present study is the first to demonstrate the antiandrogenic effect of LN at the transcript level and to examine androgenic/antiandrogenic responsiveness of the two ARs in the stickleback. From the present study, it was determined that measurement of spiggin RNA is a reliable and sensitive screening tool for the detection of both androgenic and antiandrogenic compounds. | Fish EDC | Limited, doesn't appear to be trenbolone study | Yes, confirm chemcial used |

| | | | | | | |
|---|---|---|---|---|---|---|
| Simultaneous determination of androgenic and estrogenic endpoints in the threespine stickleback (Gasterosteus aculeatus) using quantitative RT-PCR | Hogan, NS \| Wartman, CA \| Finley, MA \| van der Lee, JG \| van den Heuvel, MR | AQUAT TOXICOL \| 90 (4): 269-276 DEC 11 2008 | A method to evaluate the expression of three hormone responsive genes, vitellogenin (estrogens), spiggin (androgens), and an androgen receptor (AR beta) using real-time PCR in threespine stickleback is presented. Primers were designed from previously characterised spiggin and AR beta sequences, while a homology cloning strategy was used to isolate a partial gene sequence for stickleback vitellogenin (Vtg). Spiggin mRNA was significantly higher in kidneys of field-caught males compared to females by greater than five orders of magnitude while AR beta levels were only 1.4-fold higher in males. Female fish had four order of magnitude higher liver Vtg expression than wild-captured males. To determine the sensitivity of these genes to induction by hormones, male and female sticklebacks were exposed to 1, 10 and 100 ng/L of methyltestosterone (MT) or estradiol (E2) in a flow-through exposure system for 7 days. Spiggin induction in females, and Vtg induction in males were both detectable at 10 ng/L of MT and E2, respectively. MT exposure did not induce AR beta expression in the kidneys of female stickleback. In vitro gonadal steroid hormones production was measured in testes and ovaries of exposed stickleback to compare gene expression endpoints to an endpoint of hormonal reproductive alteration. Reduction in testosterone production in ovaries at all three MT exposure concentrations, and ovarian estradiol synthesis at the 100 ng/L exposure were the only effects observed in the in vitro steroidogenesis for either hormone exposure. Application of these methods to assess both androgenic, estrogenic, and anti-steroidogenic properties of environmental contaminants in a single fish species will be a valuable tool for identifying compounds causing reproductive dysfunction in fishes. | Fish EDC | Limited, doesn't appear to be trenbolone study | Yes, confirm chemcial used |
| Sandy sediment and the bioavailability of 17 beta-trenbolone to adult female fathead minnows | Jessick, AM \| Skolness, S \| Kolok, AS | AQUAT TOXICOL \| 148: 48-54 MAR 2014 | Recent studies have detected bioavailable steroids in sediment, however, the mechanism by which these compounds become bioavailable is not completely understood. In this study, two experiments were conducted using a double aquarium system that allowed female fathead minnows to be exposed to sandy sediments without direct contact. In the first experiment, natural sediment from the Elkhorn River (Nebraska, USA) was spiked with 17 beta-trenbolone. Both the fish in direct contact with the sediment as well as the fish excluded from direct contact experienced significant reductions in the hepatic expression of two estrogen-responsive genes, vitellogenin and estrogen receptor a, indicating molecular defeminization. The natural sediment contained particles ranging in size from sand to clay and it was possible that the fish in experiment 1 were being exposed to trenbolone associated with the very fine particles. The sandy sediment was sieved for experiment 2, and only the particles larger than 250 mu m were used. In addition, the experiment was conducted at two different Tb concentrations (1x and 10x). Furthermore nuptial tubercles, a biomarker of exposure to a masculinizing androgen, were also evaluated in the females used in experiment 2. For tubercle number and vtg expression, significant results were obtained, from a two-way ANOVA due to Tb concentration, but not tank location or interaction term (location vs. concentration). For ER alpha expression, results were found in response to Tb concentration and tank location, but not the interaction term. Overall the results from these studies suggest that the primary route of exposure of sediment-associated trenbolone to fish is through ventilation of free compound, rather than ingestion or direct contact with the sediments. | Fish EDC | Limited, sediment bioavailability work | Yes, maybe useful for fate |

| | | | | | | |
|---|---|---|---|---|---|---|
| Occurrence and biological effect of exogenous steroids in the Elkhorn River, Nebraska, USA | Kolok, AS \| Snow, DD \| Kohno, S \| Sellin, MK \| Guillette, LJ | SCIENCE OF THE TOTAL ENVIRONMENT \| 388 (1-3): 104-115 DEC 15 2007 | Recent studies of surface waters in North America, Japan and Europe have reported the presence of steroidogenic agents as contaminants. The current study has three objectives: 1) to determine if steroidogenic compounds are present in the Elkhorn River, 2) to determine if sediments collected from the Elkhorn River can act as a source of steroidogenic compounds to aquatic organisms, and 3) to determine if site-specific biological effects are apparent in the hepatic gene expression of fathead minnows. Evidence was obtained using three approaches: 1) deployment of polar organic chemical integrative samplers (POCIS), 2) deployment of caged fathead minnows, and 3) a laboratory experiment in which POCIS and fish were exposed to sediments from the deployment sites. Deployment sites included: the Elkhorn River immediately downstream from a Nebraska wastewater treatment plant, two waterways (Fisher Creek and Sand Creek) likely to be impacted by runoff from cattle feeding operations, and a reference site unlikely to be impacted by waste water inputs. The POCIS extracts were analyzed for a number of natural steroids and metabolites, as well as four different synthetic steroids: ethinylestradiol, zearalonol, 17 beta-trenbolone and melengestrot acetate. Estrogenic and androgenic metabolites, as well as progesterone and trace levels of melengestrol acetate were detected in POCIS deployed at each site. POCIS deployed in tanks containing field sediments from the four sites did not accumulate the synthetic steroids except for ethinylestradiol, which was detected in the aquarium containing sediments collected near the wastewater treatment plant. Fish deployed in Sand Creek and at the wastewater treatment plant experienced significantly elevated levels of gene expression for two genes (StAR and P450scc) relative to those deployed in Fisher Creek. Fish exposed to the sediments collected from Sand Creek had significantly higher levels of hepatic StAR and P450scc gene expression than did fish exposed to sediments from the two other field sites, as well as the no-sediment control tank. In conclusion: 1) detectable levels of steroidogenic compounds were detected in passive samplers deployed in the Elkhorn River, 2) sediments do not appear to be a significant source for steroidogenic compounds, and 3) site-specific differences were found in mRNA expression among the different treatment groups of fish; however, a functional explanation for these differences is not readily forthcoming. | Fish EDC | Limited, environmental samples | Yes |
| 5 alpha-Dihydrotestosterone is a potent androgen in the fathead minnow (Pimephales promelas) | Margiotta-Casaluci, L \| Sumpter, JP | GENERAL AND COMPARATIVE ENDOCRINOLOGY \| 171 (3): 309-318 MAY 1 2011 | Dihydrotestosterone (DHT) is one of the most physiologically important androgens in many male vertebrates, with the exception of teleost fish, in which 11-ketotestosterone (KT) is generally considered the major circulating male androgen. In the present study, we investigated the effects of KT and DHT on fathead minnow juveniles (Pimephales promelas), with the aim to compare the effects of the two androgens on critical physiological processes, such as somatic growth, male secondary sexual characteristics expression, and gonad maturation. Juvenile fish (60 days post-hatch) were exposed to 20 and 200 ng/L of KT and DHT for 45 days. Exposure to both androgens significantly stimulated somatic growth in both males (20 and 200 ng/L) and females (200 ng/L). Nuptial tubercle formation was induced by both KT and DHT, but only the latter, at 200 ng/L, caused the appearance of dorsal fin spot in 92% of males and 75% of females. Circulating plasma T concentrations showed a sex-specific response; a significant increase was recorded in exposed males and a decrease in females. Both androgens induced a significant advancement of the spermatogenic processes in males at 200 ng/L. In contrast, only DHT caused a severe disruption of ovarian physiology and morphology in females, inducing the development of spermatogenic tissue (intersex). These results show that in fathead minnow juveniles. DHT had in vivo androgenic potency comparable to KT in males, and higher than la in females, suggesting a potential involvement of DHT in the mediation of fathead minnow androgenic responses. | Fish EDC | Uncertain, does not appear to be trenbolone study | Yes, check design |

| | | | | | | | |
|---|---|---|---|---|---|---|---|
| Endocrine-disrupting effects of cattle feedlot effluent on an aquatic sentinel species, the fathead minnow | Orlando, EF \| Kolok, AS \| Binzcik, GA \| Gates, JL \| Horton, MK \| Lambright, CS \| Gray, LE \| Soto, AM \| Guillette, LJ | ENVIRONMENTAL HEALTH PERSPECTIVES \| 112 (3): 353-358 MAR 2004 | Over the last decade, research has examined the endocrine-disrupting action of various environmental pollutants, including hormones, pharmaceuticals, and surfactants, in sewage treatment plant effluent. Responding to the growth of concentrated animal feeding operations (CAFOs) and the pollutants present in their wastewater (e.g., nutrients, pharmaceuticals, and hormones), the U.S. Environmental Protection Agency developed a new rule that tightens the regulation of CAFOs. In this study, we collected wild fathead minnows (Pimephales promelas) exposed to feedlot effluent (FLE) and observed significant alterations in their reproductive biology. Male fish were demasculinized (having lower testicular testosterone synthesis, altered head morphometrics, and smaller testis size). Defeminization of females, as evidenced by a decreased estrogen:androgen ratio of in vitro steroid hormone synthesis, was also documented. We did not observe characteristics in either male or female fish indicative of exposure to environmental estrogens. Using cells transfected with the human androgen receptor, we detected potent androgenic responses from the FLE. Taken together, our morphologic, endocrinologic, and in vitro gene activation assay data suggest two hypotheses: a) there are potent androgenic substance(s) in the FLE, and/or b) there is a complex mixture of androgenic and estrogenic substances that alter the hypothalamic-pituitary-gonadal axis, inhibiting the release of gonadotropin-releasing hormone or gonadotropins. This is the first study demonstrating that the endocrine and reproductive systems of wild fish can be adversely affected by FLE. Future studies are needed to further investigate the effects of agricultural runoff and to identify the biologically active agents, whether natural or pharmaceutical in origin. | Fish EDC | Limited, supporting analytical chemsitry weak/absent | Yes |
| Density dependent functional forms drive compensation in populations exposed to stressors | Raimondo, S | ECOLOGICAL MODELLING \| 265: 149-157 SEP 10 2013 | The interaction between density dependence (DD) and environmental stressors can result in responses that range from compensatory to synergistic impacts to population growth. Models that exclude DD or use generic DD functions for populations in which density may be an important form of regulation may introduce bias into management decisions. Understanding the interaction between DD and stressors on demographic endpoints is needed to ensure models applied in management have the potential to detect compensatory or synergistic interactions between the two. This relationship was explored through the development of a DD demographic model for the sheepshead minnow (Cyprinodon variegatus) containing data-defined functions of DD for adult survival, fecundity, and growth. Concentration response curves were developed for organism-level effects from chronic laboratory studies with four chemicals (estradiol, trenbolone, trifluralin, chlordane) causing impacts that vary in endpoint and magnitude. Concentration-response curves were also developed for three hypothetical chemicals (HC) that affected only adult survival (HC-A), fecundity (HC-B), or juvenile growth (HC-C). Population growth rate (PGR) was determined across a range of densities and concentrations for each chemical. PGR contours revealed potential DD-stressor interactions ranging from compensatory to synergistic, which were a function of the combination of DD forms applied in the model and the organism-level impacts of the stressor. Simulations of population projections verified the potential compensatory and synergistic interactions of density and stressors depicted by the PGR contours. The strongest compensation occurred where survival was both DD and impacted by the stressor. When DD survival was omitted, DD fecundity and growth were drivers of PGR, but had limited compensatory influence. These interactions reflect the importance of DD demographic rates to population projections, which should be incorporated into models applied in the management of species in which density may be an important population driver. | Fish EDC | High, population modeling | Yes |

| | | | | | | |
|---|---|---|---|---|---|---|
| MULTIGENERATIONAL EXPOSURE OF THE ESTUARINE SHEEPSHEAD MINNOW (CYPRINODON VARIEGATUS) TO 17 beta-ESTRADIOL. II. POPULATION-LEVEL EFFECTS THROUGH TWO LIFE CYCLES | Raimondo, S \| Hemmer, BL \| Goodman, LR \| Cripe, GM | ENVIRONMENTAL TOXICOLOGY AND CHEMISTRY \| 28 (11): 2409-2415 NOV 2009 | The evaluation of multigeneration, population-level impacts is particularly important in the risk assessment of endocrine-disrupting compounds, because adverse effects may not be evident during the first generation of exposure. Population models were developed for the sheepshead minnow (Cyprinodon variegatus) exposed to 17 beta-estradiol (E(2)) for two complete generations (F1 and F2) to determine population-level effects of multigenerational exposure to a model estrogen. Stage-structured matrix models were used to determine interactions between treatment and the number of generations exposed. Reproduction was significantly reduced in both the 0.08 and 0.2 mu g E(2)/L treatments in both generations, and embryo and larval stages experienced reduced survival at 0.2 mu g/L in the second generation only. However, increased female to male sex ratio in these treatments compensated for the loss in reproductive output, and significant population-level effects only occurred in the 0.2 mu g E(2)/L treatment of the F2 population. The F2 population in the 0.2 mu g E(2)/L treatment also had an altered, stable stage distribution relative to the control population of both generations and the F1 population in the 0.2 mu g E(2)/L treatment, resulting in additional population-level effects. These results demonstrate that continued exposure to E(2) had compounding effects on sheepshead minnow populations and that long-term exposures may be necessary to understand the risk that exposures to environmental estrogens pose to native populations. Although population-level effects did not occur in the F1 generation, a risk decision based on F1 organism-level effects would be protective of the population exposed for two generations. | Fish EDC | High, population modeling | Yes |
| A potential biomarker of androgen exposure in European bullhead (Cottus sp.) kidney | Villeret, M \| Jolly, S \| Wiest, L \| Vulliet, E \| Bado-Nilles, A \| Porcher, JM \| Betoulle, S \| Minier, C \| Sanchez, W | FISH PHYSIOLOGY AND BIOCHEMISTRY \| 39 (3): 573-580 JUN 2013 | The aim of this study was to identify a signal that could be used as an androgen exposure indicator in the European bullhead (Cottus sp.). For this purpose, the ultra-structure of the kidney was characterized to identify normal structure of this organ, and histological changes previously described in the kidney of breeding male bullheads were quantified using the kidney epithelium height (KEH) assay previously developed and validated for the stickleback. In the next step, the effect of trenbolone acetate (TbA), a model androgen, was assessed to identify potential androgenic regulation of bullhead kidney hypertrophy. Measurement of KEH performed on adult non-breeding male and female bullheads exposed for 14 and 21 days to 0, 1.26 and 6.50 mu g/L showed that kidney hypertrophy is induced in a dose-dependent manner, confirming the hypothesis that the European bullhead possesses a potential biomarker of androgen exposure. Combined with the wide distribution of the European bullhead in European countries and the potential of this fish species for environmental toxicology studies in field and laboratory conditions, the hypothesis of a potential biomarker of androgen exposure offers interesting perspectives for the use of the bullhead as a relevant sentinel fish species in monitoring studies. Inducibility was observed with high exposure concentrations of TbA. Further studies are needed to identify molecular signals that could be more sensitive than KEH. | Fish EDC | Limited, histology effects at fairly high doses | Yes |

| | | | | | | |
|---|---|---|---|---|---|---|
| Effect of Growth Promotants on the Occurrence of Endogenous and Synthetic Steroid Hormones on Feedlot Soils and in Runoff from Beef Cattle Feeding Operations | Bartelt-Hunt, SL \| Snow, DD \| Kranz, WL \| Mader, TL \| Shapiro, CA \| van Donk, SJ \| Shelton, DP \| Tarkalson, DD \| Zhang, TC | ENVIRONMENTAL SCIENCE & TECHNOLOGY \| 46 (3): 1352-1360 FEB 7 2012 | Supplements and growth promotants containing steroid hormones are routinely administered to beef cattle to improve feeding efficiency, reduce behavioral problems, and enhance production. As a result, beef cattle manure will contain both synthetic steroids as well as a range of endogenous steroids including androgens, estrogens, and progestogens. A two-year controlled study was conducted in which beef cattle were administered steroid hormones via subcutaneous implants and feed additives and the occurrence of 16 endogenous and synthetic steroid hormones and metabolites was evaluated in runoff from beef cattle feedlots and in manure and soil collected from feedlot surfaces. Samples were extracted and analyzed using liquid chromatography tandem mass spectrometry for metabolites of the synthetic androgen trenbolone acetate, 17 alpha-trenbolone, 17 beta-trenbolone, for the nonsteroidal semisynthetic estrogen, agonist, alpha-zearalanol, and the synthetic progesterone melengesterol acetate, as well as a wide range of endogeneous estrogens, androgens, and fusarium metabolites. Synthetic steroids including trenbolone metabolites and melengestrol acetate were detected in fresh manure and in feedlot surface soils from cattle administered synthetic steroids at concentrations up to 55 +/- 22 ng/g dry weight (dw) (17 alpha-trenbolone) and 6.5 +/- 0.4 ng/g dw (melengesterol acetate). Melengesterol acetate was detected in 6% of runoff samples from feedlots holding cattle administered synthetic steroids at concentrations ranging up to 115 ng/L. The presence of melengesterol acetate in runoff from beef cattle feeding operations has not been previously reported. Synthetic steroids were not detected in manure or runoff from control cattle. A wide range of endogenous hormones were detected in runoff and feedlot surface soils and manure from cattle given synthetic steroids and from control cattle, with no statistically significant differences in concentration. These results indicate that runoff from confined animal production facilities is of environmental and public health concern regardless of the use of growth promotants. | Residue/Monitoring Study | Limited, mostly fate | Yes, exposure assessment. |
| Current knowledge on the environmental fate, potential impact, and management of growth-promoting steroids used in the US beef cattle industry | Biswas, S \| Shapiro, CA \| Kranz, WL \| Mader, TL \| Shelton, DP \| Snow, DD \| Bartelt-Hunt, SL \| Tarkalson, DD \| van Donk, SJ \| Zhang, TC \| Ensley, S | JOURN OF SOIL AND WATER CONS 68 (4): 325-336 2013 | Implications of using growth promoting steroids in the US beef cattle industry | Residue/Monitoring Study | Likely low, no abstract; may be good background | Yes |

| | | | | | | |
|---|---|---|---|---|---|---|
| Liquid chromatography-tandem mass spectrometry analysis of 17α-trenbolone, 17β-trenbolone and trendione in airborne particulate matter | Blackwell BR;Cai Q;Smith PN;Cobb GP; | Talanta %2011 , Sep 15 | Trenbolone acetate (TbA) is a potent synthetic anabolic steroid that was approved by the FDA as a growth promoter in beef cattle in 1987. Given the endocrine-modulating activity of TbA and its metabolites in all vertebrates, a sensitive and reliable analytical method is needed to detect TbA and related residues in environmental matrices. We have developed a method that incorporates solid phase extraction and liquid chromatography-tandem mass spectrometry (LC-MS/MS) for the simultaneous determination of the three major TbA metabolites (trendione, 17β-trenbolone, 17α-trenbolone) in total suspended particulate matter (TSP) samples. Sample preparation involved pressurized liquid extraction followed by cleanup on solid-phase extraction cartridges. The procedure was optimized to obtain maximum recovery and minimum signal suppression/enhancement from matrix effects. Analytes were separated with a Phenomenex Gemini-NX C18 analytical column (150 mm × 2.0 mm, 3 μm particle size) using an aqueous methanol gradient at a flow rate of 0.2 mL/min. Column effluent underwent positive electrospray ionization (ESI). Two or more diagnostic product ions were acquired from analyte specific precursor ions for unambiguous confirmation and quantification. The method detection limit was 3.27-4.87 ng/g of particulate matter (PM). Method accuracy, determined with analyte recoveries, ranged between 68% and 117%, and method precision, expressed as relative standard deviation, was below 15% at spiked levels of 6.67, 33.3, and 167 ng/g PM. Analysis of TSP samples demonstrated the presence of the target species associated with PM in the vicinity of beef cattle feeding operations | Residue/Monitoring Study | Limited, mostly fate | Yes, exposure assessment. |
| CHARACTERIZATION OF TRENBOLONE ACETATE AND ESTRADIOL METABOLITE EXCRETION PROFILES IN IMPLANTED STEERS | Blackwell, BR \| Brown, TR \| Broadway, PR \| Buser, MD \| Brooks, JC \| Johnson, BJ \| Cobb, GP \| Smith, PN | ENVIRONMENTAL TOXICOLOGY AND CHEMISTRY \| 33 (12): 2850-2858 DEC 2014 | Exogenous growth promoters have been used in US beef cattle production for over 50 yr. The environmental fate and transport of steroid growth promoters suggest potential for endocrine-disrupting effects among ecological receptors; however, the initial excretion of steroid metabolites from cattle administered growth promoters has not been well characterized. To better characterize excretion of trenbolone acetate and estrogen metabolites, steers were assigned to 1 of the following treatment groups: control, given no implant, or treatment, administered a combination implant (200mg trenbolone acetate, 40mg estradiol). Blood, urine, and fecal samples were collected over the course of 112 d following implantation. Samples were extracted and analyzed by liquid chromatography tandem mass spectrometry for trenbolone acetate and estrogen metabolites. In both urine and feces, 17-trenbolone and 17-estradiol were the predominant metabolites following implantation. Mean concentrations of 17-trenbolone and 17-estradiol in feces of implanted steers were 5.9 +/- 0.37ng/g and 2.7 +/- 0.22ng/g, respectively. A best-fit model is presented to predict 17-trenbolone and 17-estradiol excretion from steers receiving implants. The present study provides the first characterization of both trenbolone and estrogen metabolites in excreta from implanted cattle and will help provide estimates of steroid production from feedyards in the United States | Residue/Monitoring Study | Limited, mostly fate | Yes, exposure assessment. |
| Photodegradation and advanced oxidation of endocrine disruptors in aqueous solutions | Bledzka, D \| Gmurek, M \| Gryglik, M \| Olak, M \| Miller, JS \| Ledakowicz, S | CATALYSIS TODAY \| 151 (1-2): 125-130 APR 15 2010 | Endocrine disruptors, sometimes also referred to as hormonally active agents, are exogenous substances that act like hormones in the endocrine system and disrupt the physiological function of endogenous hormones. Our recent studies concern degradation of some representatives of this class of chemicals: n-butylparaben (BP), 4-t-octylphenol (OP), trenbolone (TB) and boldenone (BD). We applied three methods for their elimination from aqueous solution: photolysis by 254 nm irradiation, advanced oxidation process using hydroxyl radicals and photosensitized oxidation using mainly singlet molecular oxygen. The kinetic parameters of those processes were calculated. The most efficient degradation of studied compounds was observed in H(2)O(2)/UV system. | Residue/Monitoring Study | Limited, fate study-photolysis | Yes, exposure assessment. |

| | | | | | | |
|---|---|---|---|---|---|---|
| Prediction and Experimental Evaluation of Soil Sorption by Natural Hormones and Hormone Mimics | Card, ML \| Chin, YP \| Lee, LS \| Khan, B | JOURNAL OF AGRICULTURAL AND FOOD CHEMISTRY \| 60 (6): 1480-1487 FEB 15 2012 | Surface runoff from manure-fertilized fields is a significant source of endocrine-disrupting compounds (EDCs) in the environment. Sorption by soils may play a major role in the environmental fate of manure-borne EDCs, including 17 alpha- and 17 beta-estradiol (17 alpha-E2 and 17 beta-E2), estrone (El), melengestrol acetate (MGA), 17 alpha- and 17 beta-trenbolone (17 alpha-TB and 17 beta-TB), trendione (TND), and zeranol (alpha-ZAL). As a measure of sorption behavior, the organic carbon-normalized partition coefficients (K-OC) of 17 beta-E2, E1, MGA, and alpha-ZAL were experimentally determined for three agricultural soils with initial EDC concentrations spanning from similar to 0.01 to >1 mu M. Sorption isotherms were linear for most solute-soil combinations. Measured K-OC values were compared to those predicted using a suite of single-parameter and polyparameter linear free energy relationships (sp- and pp-LFERs). Sp-LFER models were based on experimentally determined octanol-water partition coefficients (K-OW), whereas pp-LFER solute descriptors were calculated indirectly from experimentally determined solvent-water partition coefficients or the program ABSOLV. Log K-OC predictions by sp-LFERs were closest to the experimentally determined values, whereas pp-LFER predictions varied considerably due to uncertainties in both solute and sorbent descriptors determined by ABSOLV or estimates using the partition coefficient approach. | Residue/Monitoring Study | Limited, fate | Yes, exposure assessment. |
| Hormone Discharges from a Midwest Tile-Drained Agroecosystem Receiving Animal Wastes | Gall, HE \| Sassman, SA \| Lee, LS \| Jafvert, CT | ENVIRONMENTAL SCIENCE & TECHNOLOGY \| 45 (20): 8755-8764 OCT 15 2011 | Manure is increasingly being viewed as a threat to aquatic ecosystems due to the introduction of natural and synthetic hormones from land application to agricultural fields. In the Midwestern United States, where most agricultural fields are tile-drained, there is little known about hormone release from fields receiving animal wastes. To this end, seven sampling stations (four in subsurface tile drains and three in the receiving ditch network) were installed at a Midwest farm where various types of animal wastes (beef, dairy, and poultry lagoon effluent, dairy solids, and subsurface injection of swine manure) are applied to agricultural fields. Water flow was continuously monitored and samples were collected for hormone analysis during storm events and baseline flow for a 15 month study period. The compounds analyzed included the natural hormones 17 alpha- and 17 beta-estradiol, estrone, estriol, testosterone, and androstenedione and the synthetic androgens 17 alpha- and 17 beta-trenbolone and trendione. Hormones were detected in at least 64% of the samples collected at each station, with estrone being detected the most frequently and estriol the least. Testosterone and androstendione were detected more frequently than synthetic androgens, which were detected in fewer than 15% of samples. Hormone concentrations in subsurface tile drains increased during effluent irrigation and storm events. Hormones also appeared to persist over the winter, with increased concentrations coinciding with early thaws and snowmelt from fields amended with manure solids. The highest concentration of synthetic androgens (168 ng/L) observed coincided with a snowmelt. The highest concentrations of hormones in the ditch waters (87 ng/L for total estrogens and 52 ng/L for natural androgens) were observed in June, which coincides with the early life stage development period of many aquatic species in the Midwest. | Residue/Monitoring Study | High | Yes, exposure assessment. |

| | | | | | | |
|---|---|---|---|---|---|---|
| Surface and subsurface attenuation of trenbolone acetate metabolites and manure-derived constituents in irrigation runoff on agro-ecosystems | Jones, GD \| Benchetter, PV \| Tate, KW \| Kolodziej, EP | ENVIRONMENTAL SCIENCE-PROCESSES & IMPACTS \| 16 (11): 2507-2516 NOV 2014 | Although studies have evaluated the ecotoxicity and fate of trenbolone acetate (TBA) metabolites, namely 17 alpha-trenbolone (17 alpha-TBOH), 17 beta-trenbolone (17 beta-TBOH), and trendione (TBO), their environmental transport processes remain poorly characterized with little information available to guide agricultural runoff management. Therefore, we evaluated TBA metabolite transport in representative agricultural systems with concurrent assessment of other manure-derived constituents. Leachate generated using manure from TBA-implanted cattle was applied to a subsurface infiltration plot (4 m) and surface vegetative filter strips (VFSs; 3, 4, and 5 m). In the subsurface experiment, 17 alpha-TBOH leachate concentrations were 36 ng L-1 but decreased to 12 ng L-1 in initial subsurface discharge. Over 75 minutes, concentrations linearly increased to 23 ng L-1 (C/C-o = 0.32-0.64). In surface experiments (n = 4), 17 alpha-TBOH leachate concentrations ranged from 11-150 ng L-1, remained nearly constant with time, but were attenuated by similar to 70-90% after VFS treatment with no statistical dependence on the VFS length. While attenuation clearly occurred, the observations of a highly mobile fraction of all constituents in both surface runoff and subsurface discharge suggest that these treatment strategies may not always be capable of achieving threshold discharge concentrations. To attain no observed adverse effect levels (NOAELs) in receiving waters, concurrent assessment of leachate concentrations and available dilution capacities can be used to guide target treatment performance levels for runoff management. Dilution is usually necessary to achieve NOAELs, and receiving waters with less than 70-100 fold dilution capacity are at the highest risk for steroidal endocrine disruption. | Residue/Monitoring Study | High | Yes, exposure assessment. |
| Soil temperature and moisture effects on the persistence of synthetic androgen 17alpha-trenbolone, 17beta-trenbolone and trendione | Khan B;Lee LS; | Chemosphere %2010 , May | Trenbolone acetate (TBA) is a synthetic androgenic steroid hormone administered as a subcutaneous implant for growth promotion in beef cattle. The primary metabolite excreted in manure from implanted cattle is 17alpha-trenbolone with lesser amounts of 17beta-trenbolone and trendione also present. At 22 degrees C and favorable moisture conditions in a controlled laboratory environment, trenbolone degrades to trendione in a few hours; however, these conditions are often not what exist in the field. Therefore, aerobic degradation rates of 17alpha-trenbolone, 17beta-trenbolone and trendione were determined in a sandy soil and silty clay loam under a range of temperature and water availability combinations that may be expected in the field. A first-order exponential decay model was used to estimate rates and generally resulted in good model fits to the data. Degradation rates decreased with decreasing water availability (i.e., more negative soil matric potential) and decreasing temperature. However, when water availability was substantially reduced (-1.0MPa), hotter temperatures (35 degrees C) significantly reduced trenbolone degradation rates. Once temperature was low enough to limit microbial activity, no further changes were observed with decreasing matric potential. Trendione also exhibited similar moisture and temperature dependent degradation, but persisted longer than the parent trenbolone. The latter was discussed in light of extracellular versus intracellular enzymatic degradation and sorption. Half lives at colder temperatures (5 degrees C) even under favorable moisture conditions were 2-3d for the trenbolone isomers and approached 10d for trendione | Residue/Monitoring Study | Limited, fate | Yes |
| Degradation of synthetic androgens 17alpha- and 17beta-trenbolone and trendione in agricultural soils | Khan B;Lee LS;Sassman SA; | Environ Sci Technol %2008 , May 15 | 17Beta-trenbolone acetate (TBA) is a synthetic androgenic steroid hormone administered as a subcutaneous implant for growth promotion in beef cattle. TBA is converted metabolically to primarily 17alpha-trenbolone and trendione, and excreted in manure from implanted cattle. To predict the persistence of synthetic androgens once land-applied, aerobic degradation rates in two contrasting agricultural soil types (clay loam and a sandy soil) of both trenbolone isomers (17alpha and 17beta) and their primary metabolite trendione were measured and isomer interconversion was assessed. The impact of manure application was also evaluated in the clay loam soil. A pseudo first-order exponential decay model was derived assuming irreversible transformation and no impact of sorption on availability for degradation. The model generally resulted in good fits to the data. Both isomers degraded to trendione in a similar manner with half-lives (t1/2) on the order of a few hours to 0.5 days at applied concentrations of < or = 1 mg/kg. Similar degradation rates were observed in the presence and absence of manure applied at rates typical for | Residue/Monitoring Study | Limited, fate | Yes |

| | | | | | | |
|---|---|---|---|---|---|---|
| | | | land-application of cattle manure. Trenbolone degradation was concentration-dependent with degradation rates decreasing with increasing applied concentrations. Trendione, whether applied directly or produced from trenbolone, persisted longer than trenbolone with t1/2 values of 1 to 4 days. A small amount (1.5%) of conversion of trendione back to 17beta-trenbolone was observed during aerobic incubation regardless of the applied concentration. A small amount of 17alpha-isomer also converted back to 17beta-trenbolone, presumably through trendione. In autoclaved soils, no degradation of 17alpha- or 17beta-trenbolone was observed during the first 3 days, and trendione degradation was relatively small compared to a microbially active soil | | | |
| Estrogens and synthetic androgens in manure slurry from trenbolone acetate/estradiol implanted cattle and in waste-receiving lagoons used for irrigation | Khan, B \| Lee, LS | CHEMOSPHERE \| 89 (11): 1443-1449 NOV 2012 | The increasing size of concentrated animal feeding operations has led to a concomitant increase in the land-application of manure, which has spawned research on the concentrations and environmental risk assessment of natural and synthetic hormones in animal manures. 17 beta-Trenbolone acetate (TBA) is widely used in the United States for improving daily gains in beef cattle and is often administered in combination with 17 beta-estradiol (17 beta-E2). Trenbolone (TB) and E2 isomers and their metabolites were quantified in manure collection pits and lagoon effluent from beef cattle implanted with the commercial anabolic preparation Ravoler-S (containing 140 mg 17 beta-trenbolone acetate and 28 mg 17 beta-E2). Manure pit and lagoon effluent samples were collected weekly for 9 weeks post implanting and analyzed using reverse-phase liquid chromatography tandem mass spectrometry. 17 alpha-TB was the most abundant androgen with the highest concentration observed 2 weeks post implant. 17 beta-TB and trendione peaked at the end of week 2 and 4, respectively. For the estrogens, the highest concentrations for estrone (E1), estriol (E3), and 17 alpha-E2 were observed after week 4.6. and 8, respectively. 17 beta-E2 concentrations were the lowest of the estrogens and erratic over time. In lagoon water, which is used for irrigation, 17 alpha-TB and El had the highest detected hormone concentrations (1.53 and 1.72 mu g L-1, respectively). Assuming a 1-2 order dilution during transport to surface water, these hormone levels could lead to concentrations in receiving waters that exceed some of the lowest observable effect levels (LOELs) reported for hormones (e.g., 0.01-0.03 mu g L-1). | Livestock Efficacy Study | Limited | Yes, fate |
| Chemical contaminants in feedlot wastes: Concentrations, effects and attenuation | Khan, SJ \| Roser, DJ \| Davies, CM \| Peters, GM \| Stuetz, RM \| Tucker, R \| Ashbolt, NJ | ENVIRONMENT INTERNATIONAL \| 34 (6): 839-859 AUG 2008 | Commercial feedlots for beef cattle finishing are potential sources of a range of trace chemicals which have human health or environmental significance. To ensure adequate protection of human and environmental health from exposure to these chemicals, the application of effective manure and effluent management practices is warranted. The Australian meat and livestock industry has adopted a proactive approach to the identification of best management practices. Accordingly, this review was undertaken to identify key chemical species that may require consideration in the development of guidelines for feedlot manure and effluent management practices in Australia. Important classes of trace chemicals identified include steroidal hormones, antibiotics, ectoparasiticides, mycotoxins, heavy metals and dioxins. These are described in terms of their likely sources, expected concentrations and public health or environmental significance based on international data and research. Androgenic hormones such as testosterone and trenbolone are significantly active in feedlot wastes, but they are poorly understood in terms of fate and environmental implications. The careful management of residues of antibiotics including virginiamycin, tylosin and oxytetracycline appears prudent in terms of minimising the risk of potential public health impacts from resistant strains of bacteria. Good management of ectoparasiticides including synthetic pyrethroids, macrocyclic lactones, fluazuron, and amitraz is important for the prevention of potential ecological implications, particularly towards dung beetles. Very few of these individual chemical contaminants have been thoroughly investigated in terms of concentrations, effects and attenuation in Australian feedlot wastes. | Residue/Monitoring Study | Limited | Yes, fate review |

| | | | | | | |
|---|---|---|---|---|---|---|
| Identification and Environmental Implications of Photo-Transformation Products of Trenbolone Acetate Metabolites | Kolodziej, EP \| Qu, S \| Forsgren, KL \| Long, SA \| Gloer, JB \| Jones, GD \| Schlenk, D \| Baltrusaitis, J \| Cwiertny, DM | ENVIRONMENTAL SCIENCE & TECHNOLOGY \| 47 (10): 5031-5041 MAY 21 2013 | Despite the widespread use of the anabolic androgen trenbolone acetate (TBA) in animal agriculture; evidence demonstrating the occurrence of TBA metabolites such as 17 beta-trenbolone (17 beta-TBOH), 17 alpha-trenbolone (17 alpha-TBOH), and trendione (TBO) is relatively scarce; potentially due to rapid transformation processes such as direct photolysis. Therefore, we investigated the phototransformation of TBA metabolites and associated ecological implications by characterizing the photoproducts arising from the direct photolysis of 17 beta-TBOH, 17 alpha-TBOH, and TBO and their associated ecotoxicity. LC-HRMS/MS analysis identified a range of hydroxylated products that were no longer photoactive, with primary photoproducts consisting of monohydroxy species and presumptive diastereomers. Also observed were higher-order hydroxylated products probably formed via subsequent reaction of primary photoproducts. NMR analysis confirmed the formation of 12,17-dihydroxy-estra-5(10),9(11),dien-3-one (12-hydroxy-TBOH; 2.2 mg), 10,12,17-trihydroxy-estra-4,9(11),dien-3-one (10,12-dihydroxy-TBOH; 0.7 mg); and a ring-opened 11,12-dialdehyde oxidation product (TBOH-11,12-dialdehyde; 1.0 mg) after irradiation Of similar to 14 mg of 17 beta-trenbolone. Though unconfirmed by NMR, our data suggest that the formation of additional isomeric products may occur, likely due to the reactivity of the unique 4,9,11 conjugated triene structure of trenbolone. In vivo exposure studies employing Japanese medaka (Oryzias latipes) indicate that low concentrations of 17 alpha-TBOH photoproduct mixtures can alter ovarian follicular. development,: and photoproducts. alter whole-body 17 beta-estradiol levels. Therefore, direct photolysis yields photoproducts with strong structural similarity to parent steroids, and these photoproducts still retain enough biological activity to elicit observable changes to endocrine function at trace concentrations. These data indicate that environmental transformation processes do not necessarily reduce steroid hormone ecotoxicity. | Residue/Monitoring Study | Limited, fate | Yes, background |
| The environmental impact of growth-promoting compounds employed by the United States beef cattle industry: History, current knowledge, and future directions | Kolok, AS \| Sellin, MK | REVIEWS OF ENVIRONMENTAL CONTAMINATION AND TOXICOLOGY, VOL 195 \| 195: 1-30 2008 | The current state of knowledge regarding the environmental impact of growth-promoting compounds associated with the U.S. beef cattle industry is extensive in some areas but virtually nonexistent in others. The compounds administered to the cattle are quite well understood, as are bovine metabolism and excretion. If the sex and age of the cattle on the feedlot are known, the metabolites excreted by the cattle should be predictable with a great deal of accuracy. The fate, transport, and biological effects of growth-promoting compounds are just beginning to be studied. Most of the research conducted on the fate and transport of growth-promoting compounds has focused on 17beta-E2; however, much of this research was not conducted using feedlot runoff or manure. Studies are needed that focus specifically on manures and runoff from experimental or commercial feedlots. To date, the degree to which growth-promoting compounds are released from feedlots in a bioavailable form remains a point of speculation. The environmental fate and transport of TBA, P, and MGA have not been well studied. Comparisons between the fate and transport of T and 17beta-E2, however, make it clear that compounds with similar structure may behave very differently once released into the environment. Considering that 17beta-E2 is a naturally occurring estrogen and that TBA is a nonaromatizable androgen, it is not surprising that these compounds directly impact the reproductive physiology of fishes. The effects of these two compounds have been well documented, as has been described here; however, the effects of P and MGA exposures have gone largely uninvestigated. This is a serious critical gap in our knowledge base because progestogins play an important role in sex steroid synthesis and reproduction. Clearly, additional research on the consequences of exposures to P and MGA is warranted. The majority of research investigating the effects of 17beta-E2 and TBA metabolites on fish has been conducted in the laboratory and has typically focused on continuous, pharmacological exposures to single compounds. These exposures may not bear much similarity to environmentally relevant exposures, and as such may offer little information regarding biological effects seen in nature. Cattle feedlot runoff is likely to contain a suite of growth-promoting compounds rather than any single compound. Clearly, deciphering the biological effects of exposure to | Review of livestock steroid use | High, background/review | Yes |

| | | | | | | |
|---|---|---|---|---|---|---|
| | | | complex mixtures containing androgenic, estrogenic, and progestogenic compounds will remain an important area of study for the next few years. A second complexity associated with the biological runoff from cattle feedlots is the discontinuous nature of the release. It is likely that inadvertent entry of growth-promoting compounds will follow spring snowmelt or rainstorm events. These events will result in intermittent, pulsed exposures to high concentrations of these compounds interspersed by long-term exposures to lower concentrations. The effects of exposure timing and duration should be considered to generate a clearer understanding of the biological consequences of exposures to growth-promoting compounds. To date, a very limited number of studies (only one!) have sought to determine whether fish living in waterways receiving runoff from cattle feedlots are adversely affected by growth-promoting compounds associated with the runoff. Clearly, more field studies need to be conducted before a relationship between cattle feedlot effluent and biological consequences can be elucidated | | | |
| Sex hormones originating from different livestock production systems: fate and potential disrupting activity in the environment | Lange, IG \| Daxenberger, A \| Schiffer, B \| Witters, H \| Ibarreta, D \| Meyer, HHD | ANALYTICA CHIMICA ACTA \| 473 (1-2): 27-37 NOV 25 2002 | Endogenous hormones of human or animal origin have been reaching the environment for thousands of years, even though to an increasing extent due to growing population and more intensive farming. During the last decade the hormonal disrupting activity of different substances of both natural and anthropogenic origin, has been discussed for wildlife populations in various ecosystems and even for human fertility. So far, natural recycling has not been causally linked to any known severe adverse effect on wildlife or human endocrine system, but discussion on environmental endocrine disrupters has to be extended by this important aspect. The amount of sex steroids excreted by humans and livestock seems in the same order of magnitude, but the available data on their importance is still limited. Besides endogenous hormones, exogenous sex steroids used as anabolics in animals are excreted and reach the environment. The environmental fate of steroids originating from livestock excreta seems to be strongly influenced by storage conditions and also by the soil type of the fields where the dung is spread. Particle size and organic components strongly affect adsorption and migration in the soil. Our studies indicate that low concentrations of trenbolone and melengestrol acetate are very mobile in agricultural soils. However, both hormones have a high affinity to the organic fraction of the immobile phase leading to a high retardation within soil materials. | Review of livestock steroid use | Hish, review/background | Yes |

| | | | | | | |
|---|---|---|---|---|---|---|
| Agricultural contributions of antimicrobials and hormones on soil and water quality | Lee, LS \| Carmosini, N \| Sassman, SA \| Dion, HM \| Sepulveda, MS | ADVANCES IN AGRONOMY, VOL 93 \| 93: 1-68 2007 | Detection of many emerging chemicals of concern, including antimicrobials and steroid hormones, in the environment has increased in the past decade with the advancement of analytical techniques. There are several potential sources of these inputs, including municipal wastewater discharge, municipal biosolids, pharmaceutical production, and agriculture-related activities. However, the heavy use of antibiotics in the livestock industry and the dramatic shift in recent years toward more highly concentrated animal feeding operations (CAFOs), thus a concomitant increase in the volume of animal wastes per unit of land, has drawn attention to the role of animal waste-borne antimicrobials, antibiotic-resistant bacteria, and steroid hormones on ecosystem and human health. Antimicrobials, although frequently detected, are typically present in water at concentrations in orders of magnitude below what would be considered inhibitory to most biota. Most antibiotics have a high affinity for soil and sediment, thus residual soil concentrations are usually much higher than noted in water but still often below concentrations of concern. The focal point with antibiotic use in animal production is the development of antibiotic-resistant bacteria. Although there is a growing body of evidence of the presence of numerous antibiotic-resistant genes in animal wastes, in soils where wastes are land applied, and in water bodies receiving runoff from manure-amended fields or discharges from aquacultures, conclusive evidence of animal-derived antibiotic-resistant pathogens compromising human health is lacking. In contrast to antibiotics, hormones and related chemicals can cause significant biological responses at very low concentrations. CAFO discharges will include a variety of estrogens, natural and synthetic androgens and progesterones, and phytoestrogens associated with animal feed. Measurable concentrations of many of these hormones have been detected in soil, and ground and surface waters receiving runoff from fields fertilized with animal manure and downstream from farm animal operations. Overall, hormones appear to be moderately to highly sorbed and to dissipate quickly in an aerobic soil environment, but quantitative information on hormone persistence in manure-applied fields and subsequent effects of hormone loads from CAFOs to the aquatic environment is lacking. Research directed toward evaluating the facilitated transport processes with regards to antimicrobial and hormone inputs from manure-amended fields is in its infancy. With the advances in analytical techniques and what has already been learned with regards to transport of nutrients (nitrogen, phosphorus, and carbon) and pesticides from agricultural fields, a reasonable evaluation of CAFOs and associated activities (land application of animal wastes) should be forthcoming in the next decade. Meanwhile, implementation of management practices that optimize reduction in already regulated nutrient releases from CAFOs should also help to minimize the release of antimicrobials and hormones. | Residue/Monitoring Study | Limited, fate review | Yes, background |
| Product-to-Parent Reversion of Trenbolone: Unrecognized Risks for Endocrine Disruption | Qu, S \| Kolodziej, EP \| Long, SA \| Gloer, JB \| Patterson, EV \| Baltrusaitis, J \| Jones, GD \| Benchetler, PV \| Cole, EA \| Kimbrough, KC \| Tarnoff, MD \| Cwiertny, DM | SCIENCE \| 342 (6156): 347-351 OCT 18 2013 | Trenbolone acetate (TBA) is a high-value steroidal growth promoter often administered to beef cattle, whose metabolites are potent endocrine-disrupting compounds. We performed laboratory and field phototransformation experiments to assess the fate of TBA metabolites and their photoproducts. Unexpectedly, we observed that the rapid photohydration of TBA metabolites is reversible under conditions representative of those in surface waters (pH 7, 25 degrees C). This product-to-parent reversion mechanism results in diurnal cycling and substantial regeneration of TBA metabolites at rates that are strongly temperature-and pH-dependent. Photoproducts can also react to produce structural analogs of TBA metabolites. These reactions also occur in structurally similar steroids, including human pharmaceuticals, which suggests that predictive fate models and regulatory risk assessment paradigms must account for transformation products of high-risk environmental contaminants such as endocrine-disrupting steroids. | Residue/Monitoring Study | Limited, shows environmental conversion from inactive to active isomers | Yes, fate and exposure |

| | | | | | | |
|---|---|---|---|---|---|---|
| Bioavailability and fate of sediment-associated trenbolone and estradiol in aquatic systems | Sangster, JL \| Zhang, Y \| Hernandez, R \| Garcia, YA \| Sivils, JC \| Cox, MB \| Snow, DD \| Kolok, AS \| Bartelt-Hunt, SL | SCIENCE OF THE TOTAL ENVIRONMENT \| 496: 576-584 OCT 15 2014 | Endocrine disrupting effects in aquatic organisms have been observed in systems influenced by steroid hormones. Associating endocrine disruption with aqueous concentrations of steroids alone may overlook the influence of source-sink dynamics in sediments on steroid hormone bioavailability. The objective of this study was to determine the fate of 17 beta-estradiol and 17 beta-trenbolone in two field sediments and to evaluate the corresponding bioavailability of the compounds to the fathead minnow (Pimephales promelas). Steroid fate was evaluated using analytical chemistry and verified by assessing the biological activity using yeast based in vitro assays. Effective bioavailability of the steroids was inferred from changes in hepatic vitellogenin expression (increased expression in males exposed to 17 beta-estradiol, and reduced expression in females exposed to 17 beta-trenbolone). In experiments conducted with 17 beta-estradiol, no induction of hepatic vitellogenin mRNA expression was observed in male fish exposed to sediment-associated 17 beta-estradiol. In contrast, female minnows exposed to sediment-associated 17 beta-trenbolone experienced significant reductions in hepatic vitellogenin compared to negative controls. In both systems, the parent compounds were shown to degrade rapidly to the more persistent metabolites, estrone and trendione, both of which were found predominantly associated with the sediments. Results from the yeast estrogen screen indicate a reduction in biological activity as biotransformation of 17 beta-estradiol occurs; results from the yeast anti-estrogen screen were inconclusive and unable to substantiate 17 beta-trenbolone fate in aquatic systems. Collectively, these data support the contention that steroid hormones associated with the sediment can become bioavailable to fish, and that sediment characteristics influence the observed bioavailability of these compounds. | Residue/Monitoring Study | Limited, fate, but applicability to fish | Yes |
| The fate of trenbolone acetate and melengestrol acetate after application as growth promoters in cattle: environmental studies | Schiffer B;Daxenberger A;Meyer K;Meyer HH; | Environ Health Perspect %2001 , Nov | The steroids trenbolone acetate (TbA) and melengestrol acetate (MGA) are licensed as growth promoters for farm animals in several meat-exporting countries. Although many studies have explored their safety for both animals and consumers, little is known about their fate after excretion by the animal. Our study aimed to determine the residues and degradation of trenbolone and MGA in solid dung, liquid manure, and soil. In animal experiments lasting 8 weeks, cattle were treated with TbA and MGA. Solid dung and, in case of trenbolone, liquid manure were collected and spread on maize fields after 4.5 and 5.5 months of storage, respectively. Determination of the hormone residues in all samples included extraction, clean-up (solid-phase extraction), separation of metabolites and interfering substances by HPLC (RP-18), and quantification by sensitive enzyme immunoassay. Procedures were validated by mass spectrometry (MS) methods. During storage of liquid manure the level of trenbolone decreased from 1,700 to 1,100 pg/g (17alpha-isomer), corresponding to a half-life of 267 days. Before storage, the concentrations in the dung hill ranged from 5 to 75 ng/g TbOH and from 0.3 to 8 ng/g MGA. After storage, levels up to 10 ng/g trenbolone, and 6 ng/g MGA were detected. In the soil samples trenbolone was traceable up to 8 weeks after fertilization, and MGA was detected even until the end of the cultivation period. The results show that these substances should be investigated further concerning their potential endocrine-disrupting activity in agricultural ecosystems | Residue/Monitoring Study | Limited, but good baseline fate study | Yes |
| Detection, Occurrence and Fate of Emerging Contaminants in Agricultural Environments | Snow, DD \| Cassada, DA \| Papastavros, E \| Bartelt-Hunt, SL \| Li, X \| Zhang, Y \| Zhang, YP \| Yuan, Q \| Sallach, JB | WATER ENVIRONMENT RESEARCH \| 85 (10): 869-888 2013 | A total of 62 papers published in 2012 were reviewed ranging from detailed descriptions of analytical methods, to fate and occurrence studies, to ecological effects and sampling techniques for a wide variety of emerging contaminants. New methods and studies on veterinary pharmaceuticals, steroids, antibiotic resistance genes and prion proteins in agricultural environments continue to expand our knowledge base on the occurrence and potential impacts of these compounds. This review is divided into the following sections: Introduction, Analytical Methods for Emerging Contaminants, Monitoring with Passive Samplers, Occurrence and Fate of Steroid Hormones, Antimicrobials and Antibiotic Resistance Genes, and Prions as Emerging Contaminants. | Residue/Monitoring Study | Limited, review on use, fate, etc | Yes |

| | | | | | | |
|---|---|---|---|---|---|---|
| Hormonal growth promoting agents in food producing animals | Stephany RW; | Handb Exp Pharmacol %2010 (195 ):355 -67 [Handbook of experimental pharmacology] | In contrast to the use of hormonal doping agents in sports to enhance the performance of athletes, in the livestock industry hormonal growth promoters ('anabolics') are used to increase the production of muscle meat. This leads to international disputes about the safety of meat originating from animals treated with such anabolics.As a consequence of the total ban in the EU of all hormonal active growth promoters ('hormones') in livestock production, in contrast to their legal use [e.g. of five such hormones (17beta-estradiol, testosterone, progesterone, trenbolone and zeranol) as small solid ear implants and two hormones as feed additives for feedlot heifers (melengestrol acetate) and for swine (ractopamine) in the USA], the regulatory controls also differ sharply between the EU and the USA.In the EU the treatment of slaughter animals is the regulatory offence that has to be controlled in inspection programs. In the USA testing for compliance of a regulatory maximum residue level in the edible product (muscle, fat, liver or kidney) is the purpose of the inspection program (if any).The EU inspection programs focus on sample materials that are more suitable for testing for banned substances, especially if the animals are still on the farm, such as urine and feces or hair. In the case of slaughtered animals, the more favored sample materials are bile, blood, eyes and sometimes liver. Only in rare occasions is muscle meat sampled. This happens only in the case of import controls or in monitoring programs of meat sampled in butcher shops or supermarkets.As a result, data on hormone concentrations in muscle meat samples from the EU market are very rare and are obtained in most cases from small programs on an ad hoc basis. EU data for natural hormones in meat are even rarer because of the absence of 'legal natural levels' for these hormones in compliance testing. With the exception of samples from the application sites - in the EU the site of injection of liquid hormone preparations or the site of application of 'pour on' preparations - the hormone concentrations observed in meat samples of illegally treated animals are typically in the range of a few micrograms per kilogram (ppb) down to a few tenths of a microgram per kilogram. In the EU dozens of illegal hormones are used and the number of active compounds is still expanding. Besides estrogenic, androgenic and progestagenic compounds also thyreostatic, corticosteroidal and beta-adrenergic compounds are used alone or in 'smart' combinations.An overview is given of the compounds identified on the EU black market. An estimate is also given of the probability of consumption in the EU of 'highly' contaminated meat from the application sites in cattle. Finally some data are presented on the concentration of estradiol in bovine meat from animals treated and not treated with hormone implants. These data are compared with the recent findings for estradiol concentrations in hen's eggs. From this comparison, the preliminary conclusion is that hen's eggs are the major source of 17alpha- and 17beta-estradiol in the consumer's daily 'normal' diet | Residue/Monitoring Study | Limited, some good general background | Yes |

| | | | | | | |
|---|---|---|---|---|---|---|
| Occurrence of Trenbolone Acetate Metabolites in Simulated Confined Animal Feeding Operation (CAFO) Runoff | Webster, JP \| Kover, SC \| Bryson, RJ \| Harter, T \| Mansell, DS \| Sedlak, DL \| Kolodziej, EP | ENVIRONMENTAL SCIENCE & TECHNOLOGY \| 46 (7): 3803-3810 APR 3 2012 | Metabolites of androgenic synthetic growth promoters used at confined animal feeding operations (CAFOs) pose a demonstrated ecological risk. To evaluate the transport of trenbolone acetate (TBA) metabolites from beef cattle CAFOs., rainfall simulation experiments were conducted at the University of California, Davis, research CAFO. Steroid concentrations in solid and aqueous samples from the research CAFO and solids samples from a commercial CAFO were analyzed by gas chromatography-tandem mass spectrometry. The data indicate that 17 alpha-trenbolone (17 alpha-TBOH), 17 beta-trenbolone (17 beta-TBOH), and trendione (TBO), the three primary TBA metabolites, occur in soils and runoff. Soils at the research CAFO contained up to 8.2 (+/- 1.1) ng/g-dw of 17 alpha-TBOH and 1.2 (+/- 0.1) ng/g-dw of 17 beta-TBOH, with slightly higher (similar to 20 ng/g-dw) 17 alpha-TBOH concentrations observed in commercial CAFO soils. In simulated runoff, 17 alpha-TBOH concentrations of 1-350 ng/L and TB() concentrations from 1-170 ng/L were observed. The metabolite 17 beta-TBOH intermittently occurred in runoff samples at 5-26 ng/L and may be correlated to anaerobic soils. Metabolite concentrations observed in CAFO runoff correspond to 5-15% of potential maximum steroid concentrations predicted by mass balances. First order transformation rates of 0.028/day (25 day half-life) were estimated for 17 alpha-TBOH in CAFO soils. Results suggest that ecologically relevant concentrations of TBA metabolites can be mobilized from CAFO surfaces in storm runoff and may lead to receiving water concentrations at or above ecological effects thresholds for a very limited number of discharge scenarios. | Residue/Monitoring Study | Limited, all fate | Yes, background |
| Rates and product identification for trenbolone acetate metabolite biotransformation under aerobic conditions | Cole et al | Environmental Toxicology and Chemistry Volume 34, Issue 7, pages 1472–1484, July 2015 | Trenbolone acetate metabolites are endocrine-active contaminants discharged into the aquatic environment in runoff from agricultural fields, rangelands, and concentrated animal feeding operations. To investigate the environmental fate of these compounds and their biotransformation mechanisms, the authors used inocula from a variety of different water sources and dosed biologically active microcosms with approximately 1400 ng/L of trenbolone acetate metabolites, including 17β-trenbolone, trendione, and 17α-trenbolone. To investigate aerobic biotransformation rates and interconversions between known trenbolone acetate metabolites, gas chromatography-tandem mass spectrometry was used to measure concentrations and assess product distributions as a function of time. High-resolution liquid chromatography-tandem mass spectrometry (LC-MS/MS) was used to characterize novel transformation products and potential transformation pathways. Kinetic analysis yields observed half-lives of approximately 0.9 d, 1.3 d, and 2.2 d for 17β-trenbolone, trendione, and 17α-trenbolone, respectively, at 20 °C, although colder conditions increased half-lives to 8.5 d and biphasic transformation was observed. Relative to reported faster attenuation rates in soils, trenbolone acetate metabolites are likely more persistent in aqueous systems. Product distributions indicate an enzymatic preference for biotransformation between trendione and 17β-trenbolone. The LC-MS/MS characterization indicates dehydrogenation products as the major detectable products and demonstrates that major structural elements responsible for bioactivity in steroids are likely retained during biotransformation | Residue/Monitoring Study | Fate | Yes |

| | | | | | | |
|---|---|---|---|---|---|---|
| Stereoselective Sorption by Agricultural Soils and Liquid–Liquid Partitioning of Trenbolone (17α and 17β) and Trendione | Khan et al 2009 | Environ Sci & Techn 43(23):8827-8833 | Trenbolone acetate (TBA) is a synthetic anabolic hormone used for growth promotion in beef cattle, which excrete primarily 17α-trenbolone along with small amounts of 17β-trenbolone and trendione. To aid in predicting transport of manure-borne TBA metabolites, multiconcentration sorption isotherms for 17α- and 17β-trenbolone and trendione were generated with five autoclaved-sterilized soils that represented a range in soil properties. Hormone concentrations were measured independently in solution and soil phases, and quantified using liquid chromatography with electrospray mass spectrometry. In addition, partition coefficients between apolar hexane and water (Khw) and bipolar octanol and water (Kow) were measured for the three androgens to better ascertain the mechanisms that may be responsible for the sorption differences observed between isomers. In all five soils, trendione sorbed the most, and 17α- and 17β-trenbolone isomers exhibited different sorption magnitudes. 17β-trenbolone consistently sorbed a factor of 2 more than 17α-trenbolone. For all three androgens, sorption is proportional to the soil organic carbon (OC) content with average log OC-normalized distribution coefficients (log Koc, L/kg OC) of 2.77 ± 0.12 for 17α-trenbolone, 3.08 ± 0.1 for 17β-trenbolone and 3.38 ± 0.19 for trendione, which suggests the dominance of hydrophobic partitioning. However, differences in Khw values between 17α- and 17β-trenbolone were small indicating differences are not simply due to differences in aqueous activity. In contrast, similarly different Kow and Koc values for the two isomers indicate the likely contribution of H-bonding to stereoselective sorption. | Residue/Monitoring Study | Fate | Yes |
| Sorption and Mineral-Promoted Transformation of Synthetic Hormone Growth Promoters in Soil Systems | Qu et al 2014 | Journal of Agriculture and Food Chemistry 62(51):12277-12286 | This work examines the fate of synthetic growth promoters (trenbolone acetate, melengestrol acetate, and zeranol) in sterilized soil systems, focusing on their sorption to organic matter and propensity for mineral-promoted reactions. In organic-rich soil matrices (e.g., Pahokee Peat), the extent and reversibility of sorption did not generally correlate with compound hydrophobicity (e.g., Kow values), suggesting that specific binding interactions (e.g., potentially hydrogen bonding through C17 hydroxyl groups for the trenbolone and melengestrol families) can also contribute to uptake. In soils with lower organic carbon contents (1–5.9% OC), evidence supports sorption occurring in parallel with surface reaction on inorganic mineral phases. Subsequent experiments with pure mineral phases representative of those naturally abundant in soil (e.g., iron, silica, and manganese oxides) suggest that growth promoters are prone to mineral-promoted oxidation, hydrolysis, and/or nucleophilic (e.g., $H_2O$ or $OH^–$) addition reactions. Although reaction products remain unidentified, this study shows that synthetic growth promoters can undergo abiotic transformation in soil systems, a previously unidentified fate pathway with implications for their persistence and ecosystem effects in the subsurface. | Residue/Monitoring Study | Fate | Yes |

| | | | | | | |
|---|---|---|---|---|---|---|
| Characterisation of the affinity of different anabolics and synthetic hormones to the human androgen receptor, human sex hormone binding globulin and to the bovine progestin receptor | Bauer, ERS \| Daxenberger, A \| Petri, T \| Sauerwein, H \| Meyer, HHD | APMIS \| 109: S452-S460 Suppl. 103 2001 | For the steroidal growth promoters trenbolone acetate (TBA) and melengestrol acetate (MGA) neither the complete spectrum of biological activities nor the potential endocrine disrupting activity of their excreted metabolites in the environment is fully understood. The potency of these substances in [H-3]dihydrotestosterone ([H-3]-DHT) displacement from the recombinant human androgen receptor (rhAR) and from human sex-hormone binding globulin (hSHBG) was evaluated. In addition, the potency for [H-3]-ORG2058 displacement from the bovine uterine progestin receptor (bPR) was tested. For comparison, different anabolics and synthetic hormones were also tested for their binding affinities. For 17 beta -trenbolone (17 beta -TbOH), the active compound after TBA administration, an affinity the rhAR similar to dihydrotestosterone (DHT) and a slightly higher affinity to the bPR than progesterone were demonstrated. The affinity of the two major metabolites, 17 alpha -trenbolone and trendione, was reduced to less than 5% of the 17 beta -TbOH-value. The affinity of these three compounds and of MGA to the hSHBG was much lower compared with DHT MGA showed a 5.3-fold higher affinity than progesterone to the bPR but only a weak affinity to the rhAR. The major MGA metabolites have an affinity to the bPR between 85% and 28% of the affinity of progesterone. In consequence, MGA and TBA metabolites may be hormonally active substances, which will be present in edible tissues and in manure. We conclude that detailed investigations on biodegradation, distribution and bio-efficacy of these substances are necessary. | Mammalian EDC Study | Limited, largely in vitro | Yes, background |
| Uptake of 17 beta-trenbolone and subsequent metabolite trendione by the pinto bean plant (Phaseolus vulgaris) | Blackwell, BR \| Karnjanapiboonwong, A \| Anderson, TA \| Smith, PN | ECOTOX & ENVIRON SAFETY \| 85: 110-114 NOV 1 2012 | Manure from livestock feeding operations is commonly applied to agricultural fields as an alternative to commercial fertilizers. Trenbolone acetate (TbA) is a synthetic growth promoter frequently utilized in beef cattle feeding operations. Metabolites of TbA can be present in manure and subsequently applied to fields. Fate of TbA metabolites 17 beta-trenbolone (17 beta Tb), 17 alpha-trenbolone (17 alpha Tb), and trendione (TbO) have been assessed in manure and soils, but plant uptake in agricultural fields is not fully understood. The objective of this study was to investigate potential plant uptake and biotransformation of 17 beta Tb using the pinto bean plant (Phaseolus vulgaris). Vegetated (n=20) and control sands (n=16) were amended with 17 beta Tb at a level of 1 mu g/g once per week for a total of four weeks. Sand, above-ground plant portion and below-ground plant portion were collected each week and then analyzed for 17 beta Tb, 17 alpha Tb, and TbO. By week four, low concentrations of 17 beta Tb (10 +/- 4.9 mu g/g fresh weight) were taken up into the roots of plants and, to a much lesser extent, translocated throughout the plant (0.04 +/- 0.02 mu g/g fresh weight). Extensive transformation of 17 beta Tb to the metabolite trendione (TbO) occurred in vegetated sand, while minimal TbO was detected in control sand. These results suggest the biotransformation of 17 beta Tb to TbO is predominantly through microbial degradation. Trenbolone (Tb) metabolites can then be taken up into plants but remain concentrated in the roots with only slight translocation to above ground portions of the plant. After four weeks, maximum observed concentrations of total Tb (parent+metabolites) in fresh plant tissues were 33.0 mu g/g in roots and 0.25 mu g/g in leaves. No phytotoxicity was observed to pinto bean plants throughout the four week study. | Residue/Monitoring Study | Limited, fate study-plant uptake | Yes, exposure assessment. |

| | | | | | | |
|---|---|---|---|---|---|---|
| Characterization of the androgen-sensitive MDA-kb2 cell line for assessing complex environmental mixtures | Blake LS;MartinovićGray LE;Wilson VS;Regal RR;Villeneuve DL;Ankley GT; | Environ Toxicol Chem %2010 , Jun | Synthetic and natural steroidal androgens and estrogens and many other non-steroidal endocrine-active compounds commonly occur as complex mixtures in aquatic environments. It is important to understand the potential interactive effects of these mixtures to properly assess their risk. Estrogen receptor agonists exhibit additivity in mixtures when tested in vivo and in vitro. Little is known, however, concerning possible mixture interactions of androgen receptor agonists. In these studies we used the MDA-kb2 cell line, a human breast cancer cell line with endogenous androgen receptors and a stably transfected luciferase reporter gene construct to quantify the androgenic activity of seven natural and synthetic androgens: 17beta-trenbolone, dihydrotestosterone, methyltestosterone, testosterone, trendione, 17alpha-trenbolone, and androstenedione. We tested combinations of these androgens and compared the observed activity to expected androgenic activity based on a concentration addition model. Our analyses support the hypothesis that androgen receptor agonists cause additive responses in a mixture. Binary mixtures of 17beta-trenbolone with 17beta-estradiol or triclocarban (an anti-microbial found in the environment) were also tested. 17beta-Estradiol induced androgenic activity, but only at concentrations 600-fold greater than those found in the environment. Triclocarban enhanced the activity of 17beta-trenbolone. Additionally, three anti-androgens were each paired with three androgens of varying potencies. The relative potencies of the antagonists were a vinclozolin metabolite (M2) > procymidone > prochloraz regardless of the androgen used. The results of our studies demonstrate the potential utility of the androgen-responsive MDA-kb2 cell line for quantifying the activity of mixtures of endocrine-active chemicals in complex wastes such as municipal effluents and feedlot discharges | Mammalian EDC Study | Limited, in vitro | Yes, mixture interactions. |
| Development of an androgen reporter gene assay (AR-LUX) utilizing a human cell line with an endogenously regulated androgen receptor | Blankvoort, BMG \| de Groene, EM \| van Meeteren-Kreikamp, AP \| Witkamp, RF \| Rodenburg, RJT \| Aarts, JMMJG | ANALYTICAL BIOCHEMISTRY \| 298 (1): 93-102 NOV 1 2001 | The aim of the work described in this report is to develop and characterize a cell-based androgen reporter assay. For this purpose, the androgen receptor (AR) expressing human breast cancer cell line T47D was stably transfected with a luciferase gene under transcriptional control of the PB-ARE-2 androgen response element. The application of this cell line in an endogenous Androgen Receptor-mediated LUciferase eXpression assay (ARLUX) was validated. Ain EC50 value of 86 pM was determined for the standard androgen R1881 with a detection limit of 46 pM. Other androgens like dihydrotestosterone, 17 beta -trenbolone, and bolasterone also induced luciferase expression, while anti-androgens suppressed these responses. As expected, AP-mediated responses were also elicited by high concentrations of the steroids progesterone, 17 beta -estradiol, d-aldosterone, and dexamethasone, with observed EC50 values 10 to 350,000 times higher than that for R1881. A unique feature of the AR-LUX assay is that effects on modulation of active endogenous AR-levels are reliably reflected in the luciferase induction response, as exemplified by vitamin D, all-trans-retinoic acid, epigallocatechin gallate, and forskolin. This feature is especially useful when assessing complex mixtures, e.g., environmental samples or natural compound libraries. From these data it is concluded that the AR-LUX assay is a reliable in vitro test system for the detection and quantification of AR-mediated biological effects. The 96-well plate format makes the assay particularly suitable for high-throughput screening. | Mammalian EDC Study | Limited, level 2 in vitro MoA information, possible potency information | Yes |

| | | | | | | |
|---|---|---|---|---|---|---|
| Low-dose effects and biphasic effect profiles: is trenbolone a genotoxicant? | Boettcher M;Kosmehl T;Braunbeck T; | Mutat Res %2011 , Aug 16 | Over the last years, extensive research has documented endocrine-disrupting activities for a significant number of substances including, among others, hormones, pharmaceuticals, pesticides and surfactants. Nonetheless, for most endocrine disruptors, toxicological profiles are still incomplete or even lacking. A systematic review has shown that a number of endocrine disruptors with steroid-modulating effects may also exert mutagenic and carcinogenic activities. For trenbolone, an androgenic compound, there is controversy about its genotoxic properties in the literature, apparently with a strong dependence on the choice of the test system. Since fish and other aquatic animals are at risk of exposure to run-offs from cattle feedlots or sewage-discharge sites containing trenbolone, potential consequences to aquatic ecosystems need to be assessed. To this end, the potential genotoxic hazard of trenbolone was tested in vitro in the permanent rainbow trout-liver cell-line RTL-W1, as well as in primary cell cultures derived from zebrafish (Danio rerio) embryos after in vivo exposure. In either test system, a potential genotoxic hazard characterized by biphasic dose-response curves could be documented even at exposure concentrations of 30μg/L. These results thus confirm the conclusion that the steroid trenbolone may act as a genotoxic substance | Fish EDC | Limited, in vitro work, high dose | Yes, genotox investigation in fish |
| Environmental (anti-)androgenic chemicals affect germinal vesicle breakdown (GVBD) of Xenopus laevis oocytes in vitro | Cao, S \| Xu, W \| Lou, QQ \| Zhang, YF \| Zhao, YX \| Wei, WJ \| Qin, ZF | TOXICOLOGY IN VITRO \| 28 (3): 426-431 APR 2014 | Progesterone-induced germinal vesicle breakdown (GVBD) of Xenopus oocytes in vitro was used to study endocrine disrupting activity of chemicals in previous studies. In this study, we investigated for the first time effects of environmental androgens on oocyte maturation and effects of anti-androgens on androgen-induced oocyte maturation, using Xenopus GVBD in vitro. Trenbolone and nandrolone, two environmental androgens, were found to induce Xenopus GVBD at low concentrations. The potential of trenbolone to induce GVBD was approximately 100-fold lower than that of testosterone, while nandrolone had a several-fold lower potential than testosterone. Our findings have aroused new concerns for effects of environmental androgens on amphibian oocyte maturation at environmentally relevant concentrations, and suggested that Xenopus GVBD can be used to test androgenic activity of suspicious environmental androgens. Androgen receptor (AR) antagonist flutamide at 10 mu M only exhibited a weakly inhibitory effect on androgen-induced GVBD, while another known AR antagonist vinclozolin had no effect even at high concentrations. The results show that Xeno pus GVBD is not sensitive to AR-mediated environmental anti-androgens. In contrast to flutamide and vinclozolin, methoxychlor (a weaker AR antagonist) inhibited dramatically androgen-induced GVBD, suggesting that androgen-induced Xenopus GVBD can be used to study non-AR-mediated effects of chemicals on oocyte maturation. | Amphibian EDC Study | High | Yes, mechanistic and apical endpoints |

| | | | | | | |
|---|---|---|---|---|---|---|
| Highly active human pharmaceuticals in aquatic systems: A concept for their identification based on their mode of action | Christen, V \| Hickmann, S \| Rechenberg, B \| Fent, K | AQUAT TOXICOL \| 96 (3): 167-181 FEB 18 2010 | Widespread occurrence of traces of pharmaceuticals (ng/L to mu g/L) has been reported in aquatic systems. However, their effects on the environment and their environmental risks remain elusive. Generally, the acute toxicity towards non-target organisms has been assessed in laboratory experiments, but chronic toxicity studies have been performed only rarely. The guideline issued by the European Medicines Agency in 2006 is aimed at estimating the potential environmental risks of human pharmaceuticals by a tiered approach. The predicted environmental concentration (PEC) of a compound is estimated in phase I, and pharmaceuticals having a PEC above or equal 10 ng/L undergo phase II testing. Otherwise they are not expected to pose a risk to the environment. Because some highly active Compounds (HC) such as 17-alpha-ethinylestradiol, equine estrogens, trenbolone and the progestin levonorgestrel display adverse effects at concentrations below 10 ng/L the question arises, whether additional HC compounds exist, and how they can be identified for undergoing environmental risk assessment. We addressed this question by searching for HC in the literature, and by developing a concept for identification of HC. The suggested mode of action concept is based on (i) the mode of action of the pharmaceutical taking the available toxicological information into account, (ii) the degree of sequence homology between the human drug target and the potential target in aquatic organisms and (iii) the importance of pathways affected by the pharmaceutical. We evaluated the mode of action concept by comparison to existing approaches, the fish plasma model (Huggett et al., 2003) and a QSAR model, called VirtualTox Lab (www.biograf.ch). All concepts result in similar classifications of the selected pharmaceuticals. However, there are some differences not only in the model assumptions, but also in its results. Our study leads to the conclusion that the mode of action concept is most suitable for the identification of HC. A refinement can be achieved by complementing this concept by the QSAR model (VirtualTox Lab), whereas the fish plasma model seemed to be less suitable due to the necessity of environmental concentration above 10 ng/L for the identification of a risk. | Environment al Risk Assessment | High | Yes, background |
| Identification of metabolites of trenbolone acetate in androgenic runoff from a beef feedlot | Durhan, EJ \| Lambright, CS \| Makynen, EA \| Lazorchak, J \| Hartig, PC \| Wilson, VS \| Gray, LE \| Ankley, GT | ENVIRONMENTAL HEALTH PERSPECTIVES \| 114: 65-68 Suppl. 1 APR 2006 | Little is known concerning the potential ecological effects of hormonally active substances associated with discharges from animal feeding operations. Trenbolone acetate is a synthetic anabolic steroid that is widely used in the United States to promote growth of beef cattle. Metabolites of trenbolone acetate include the stereoisomers 17 alpha- and 17 beta-trenbolone, both of which are stable in animal wastes and are relatively potent androgens in fish and mammals. Our purpose in this study was to evaluate the occurrence of 17 alpha- and 17 beta-trenbolone in a beef cattle feedlot discharge and in river water upstream and downstream from the discharge. In conjunction with that effort, we measured in vitro androgenic activity of the discharge using CV-1 cells that had been transiently cotransfected with human androgen receptor and reporter gene constructs. Samples were collected on nine different occasions during 2002 and 2003. Whole-water samples from the discharge caused a significant androgenic response in the CV-1 cells and contained detectable concentrations of 17 alpha- and 17 beta-trenbolone. Further work is needed to ascertain the degree to which synthetic androgens such as trenbolone contribute to androgenic activity of feedlot discharges. | Fish EDC | High | Yes, exposure assessment. |

| | | | | | | |
|---|---|---|---|---|---|---|
| Environmental Gestagens Activate Fathead Minnow (Pimephales promelas) Nuclear Progesterone and Androgen Receptors in Vitro | Ellestad, LE \| Cardon, M \| Chambers, IG \| Farmer, JL \| Hartig, P \| Stevens, K \| Villeneuve, DL \| Wilson, V \| Orlando, EF | ENVIRONMENTAL SCIENCE & TECHNOLOGY \| 48 (14): 8179-8187 JUL 15 2014 | Gestagen is a collective term for endogenous and synthetic progesterone receptor (PR) ligands. In teleost fishes, 17 alpha,20 beta-dihydroxy-4-pregnen-3-one (DHP) and 17 alpha,20 beta,21-trihydroxy-4-pregnen-3-one (20 beta-S) are the predominant progestogens, whereas in other vertebrates the major progestogen is progesterone (P4). Progestins are components of human contraceptives and hormone replacement pharmaceuticals and, with P4, can enter the environment and alter fish and amphibian reproductive health. In this study, our primary objectives were to clone the fathead minnow (FHM) nuclear PR (nPR), to develop an in vitro assay for FHM nPR transactivation, and to screen eight gestagens for their ability to transactivate FHM nPR. We also investigated the ability of these gestagens to transactivate FHM androgen receptor (AR). Fish progestogens activated FHM nPR, with DHP being more potent than 20 beta-S. The progestin drospirenone and P4 transactivated the FHM nPR, whereas five progestins and P4 transactivated FHM AR, all at environmentally relevant concentrations. Progestins are designed to activate human PR, but older generation progestins have unwanted androgenic side effects in humans. In FHMs, several progestins proved to be strong agonists of AR. Here, we present the first mechanistic evidence that environmental gestagens can activate FHM nPR and AR, suggesting that gestagens may affect phenotype through nPR- and AR-mediated pathways. | Fish EDC | Limited, not clear that trenbolone was studied | Yes, need to check design |
| Anabolics: the approach taken in the USA | Farber TM; | Ann Rech Vet %1991 ;22 (3):295 -8 | In the United States, the Food and Drug Administration has developed a scientifically sound and rational approach to assure human safety from both naturally occurring and synthetically-derived hormones used in animal production. On this basis, estradiol, progesterone, androsterone, zeranol and trenbolone have been registered. For trenbolone a maximal residue limit of 50 ppb for meat has been accepted | Livestock Efficacy Study | Limited | Yes, background and maybe exposure assessment for non-aqueous species |
| Hormones in international meat production: biological, sociological and consumer issues | Galbraith H; | Nutr Res Rev %2002 , Dec | Beef and its products are an important source of nutrition in many human societies. Methods of production vary and include the use of hormonal compounds ('hormones') to increase growth and lean tissue with reduced fat deposition in cattle. The hormonal compounds are naturally occurring in animals or are synthetically produced xenobiotics and have oestrogenic (oestradiol-17beta and its esters; zeranol), androgenic (testosterone and esters; trenbolone acetate) or progestogenic (progesterone; melengestrol acetate) activity. The use of hormones as production aids is permitted in North American countries but is no longer allowed in the European Union (EU), which also prohibits the importation of beef and its products derived from hormone-treated cattle. These actions have resulted in a trade dispute between the two trading blocs. The major concern for EU authorities is the possibility of adverse effects on human consumers of residues of hormones and metabolites. Methods used to assess possible adverse effects are typical of those used by international agencies to assess acceptability of chemicals in human food. These include analysis of quantities present in the context of known biological activity and digestive, absorptive, post-absorptive and excretory processes. Particular considerations include the low quantities of hormonal compounds consumed in meat products and their relationships to endogenous production particularly in prepubertal children, enterohepatic inactivation, cellular receptor- and non-receptor-mediated effects and potential for interference with growth, development and physiological function in consumers. There is particular concern about the role of oestradiol-17beta as a carcinogen in certain tissues. Now subject to a 'permanent' EU ban, current evidence suggests that certain catechol metabolites may induce free-radical damage of DNA in cell and laboratory animal test systems. Classical oestrogen-receptor mediation is considered to stimulate proliferation in cells maintaining receptivity. Mathematical models describing quantitative relationships between consumption of small amounts of oestrogens in | Livestock Efficacy Study | Limited | Yes, perhaps a background review |

| | | | | | | |
|---|---|---|---|---|---|---|
| | | | meat in addition to greater concentrations from endogenous production, chemical stoichiometry at cellular level and human pathology have not been developed. Such an approach will be necessary to establish 'molecular materiality' of the additional hormone intake as a component of relative risk assessment. The other hormones, although generally less well researched, are similarly subject to a range of tests to determine potentially adverse effects. The resulting limited international consensus relates to the application of the 'precautionary principle' and non-acceptance by the European Commission of the recommendations of the Codex Alimentarius Commission, which determined that meat from cattle, hormone-treated according to good practice, was safe for human consumers. The present review considers the hormone issue in the context of current international social methodology and regulation, recent advances in knowledge of biological activity of hormones and current status of science-based evaluation of food safety and risk for human consumers | | | |
| Modulation of steroidogenic gene expression and hormone production of H295R cells by pharmaceuticals and other environmentally active compounds | Gracia, T \| Hilscherova, K \| Jones, PD \| Newsted, JL \| Higley, EB \| Zhang, X \| Hecker, M \| Murphy, MB \| Yu, RMK \| Lam, PKS \| Wu, RSS \| Giesy, JP | TOXICOLOGY AND APPLIED PHARMACOLOGY \| 225 (2): 142-153 DEC 1 2007 | The H295R cell bioassay was used to evaluate the potential endocrine disrupting effects of 18 of the most commonly used pharmaceuticals in the United States. Exposures for 48 It with single pharmaceuticals and binary mixtures were conducted; the expression of five steroidogenic genes, 3 beta HSD2, CYP11 beta 1, CYP11 beta 2, CYP17 and CYP19, was quantified by Q-RT-PCR. Production of the steroid hormones estradiol (E2), testosterone (T) and progesterone (P) was also evaluated. Antibiotics were shown to modulate gene expression and hormone production. Amoxicillin up-regulated the expression of CYP11 beta 2 and CYP19 by more than 2-fold and induced estradiol production up to almost 3-fold. Erythromycin significantly increased CYP11 beta 2 expression and the production of P and E2 by 3.5- and 2.4-fold, respectively, while production of T was significantly decreased. The p-blocker salbutamol caused the greatest induction of CYP17, more than 13-fold, and significantly decreased E2 production. The binary mixture of cyproterone and salbutamol significantly down-regulated expression of CYP19, while a mixture of ethynylestradiol and trenbolone, increased E2 production 3.7-fold. Estradiol production was significantly affected by changes in concentrations of trenbolone, cyproterone, and ethynylestradiol. Exposures with individual pharmaceuticals showed the possible secondary effects that drugs may exert on steroid production. Results from binary mixture exposures suggested the possible type of interactions that may occur between drugs and the joint effects product of such interactions. Dose-response results indicated that although two chemicals may share a common mechanism of action the concentration effects observed may be significantly different. | Mammalian EDC Study | Limited, uses high dose with trenbolone as a "postive" model for method validation | Yes, in vitro data |

| | | | | | | |
|---|---|---|---|---|---|---|
| The anti-estrogenic activity of sediments from agriculturally intense watersheds: Assessment using in vivo and in vitro assays | Jeffries, MKS \| Conoan, NH \| Cox, MB \| Sangster, JL \| Balsiger, HA \| Bridges, AA \| Cowman, T \| Knight, LA \| Bartelt-Hunt, SL \| Kolok, AS | AQUAT TOXICOL \| 105 (1-2): 189-198 SEP 2011 | The goal of the current study was to determine whether sediments from agriculturally intense watersheds can act as a potential source of anti-estrogenic endocrine-disrupting compounds. The specific objectives of the current study were to determine (1) whether female fathead minnows (Pimephaies promelas) experience alterations in endocrine function when exposed to sediments collected from agriculturally intense watersheds and (2) if these sediments display anti-estrogenic activity in an in vitro assay. In addition, sediment samples were analyzed for the presence of steroid hormones and pesticides associated with local agricultural practices. To accomplish this, sediments and water were collected from three sites within two agriculturally intense Nebraska watersheds (Bow Creek and the Elkhorn River). In 2009, minnows were exposed to sediment and/or water collected from the two Bow Creek sites (East Bow Creek and the Confluence) in the laboratory, while in 2010, minnows were exposed to sediment and/or water from East Bow Creek, the Confluence and the Elkhorn River. Following the 7-day exposure period, the hepatic mRNA expression of two-estrogen responsive genes, estrogen receptor alpha (ER alpha) and vitellogenin (Vtg) was determined. In 2009, females exposed to Confluence sediments, in the presence of laboratory water or Confluence water, experienced significant reductions in ER alpha expression relative to unexposed and Confluence water-exposed females. The defeminization of these females suggests the presence of a biologically available anti-estrogenic compound in sediments collected from this site. In 2010, sediments were assessed for anti-estrogenic activity on days 0 and 7 of the exposure period using a 4-h yeast estrogen screen. Lipophilic extracts (LEs) of day 0 sediments collected from the Confluence and the Elkhorn River induced significant reductions in the estrogenic reporter activity of treated yeast cultures suggesting the presence of a lipophilic anti-estrogenic compound in these extracts. Chemical analysis revealed the presence of a variety of steroid hormones, including those associated with the production of beef cattle (i.e. beta-trenbolone, alpha-zearalanol and alpha-zearalenol), in sediments indicating that compounds utilized by local beef cattle operations are capable of entering nearby watersheds. Overall, the results of this study indicate that an environmentally relevant anti-estrogenic compound is present in sediments from agriculturally intense watersheds and that this compound is bioavailable to fish. Furthermore, the presence of steroid hormones in sediments from these watersheds provides evidence indicating that steroids are capable of sorbing to sediments. | Fish EDC | Limited, complex mixture analysis | Yes, background /fate |

| | | | | | | |
|---|---|---|---|---|---|---|
| Development of a stickleback kidney cell culture assay for the screening of androgenic and anti-androgenic endocrine disrupters | Jolly C;Katsiadaki I;Le BN;Mayer I;Dufour S; | Aquat Toxicol %2006 , Aug 23 | Issues raised by the presence in the environment of chemicals able to mimic or antagonize the action of androgenic hormones are of growing concern. Here we report the development of a novel in vitro test for the screening of (anti-)androgenic chemicals, based on primary cultures of stickleback kidney cells that produce a protein, the spiggin, in response to androgenic stimulation. Cell spiggin content was measured by ELISA. Comparison between cell cultures from quiescent males, photoperiodically stimulated males, control females and dihydrotestosterone (DHT)-primed females led to the selection of cell cultures from DHT-primed females for the development of a standardized protocol. 48h of treatment with androgens proved to be sufficient to induce concentration-dependent increase in spiggin cell content with a high sensitivity. DHT induced a significant spiggin increase at 10(-12)M, while testosterone (T) and the teleost specific androgen 11-ketotestosterone (11-KT) had a significant effect at 10(-10)M. Maximal responses were obtained with 10(-8)M DHT and 10(-6)M T and 11-KT. This indicates a higher sensitivity to DHT than to T and 11-KT, in agreement with previous data on stickleback kidney androgen receptor affinity. No effect was observed with other steroids or thyroid hormone, indicating the androgen specificity of the test. The anabolic steroid 17beta-Trenbolone (TB) was able to stimulate spiggin synthesis in a concentration-dependent manner with a significant effect at a concentration as low as 10(-10)M, and a maximal effect at 10(-6)M. The synthetic human androgen receptor antagonist, flutamide had no effect alone, but concentration-dependently inhibited the stimulatory effect of 10(-8)M 11-KT with a complete inhibition at 10(-6)M flutamide. This cell culture system provides an innovative tool for the rapid and sensitive screening of androgenic and anti-androgenic properties of environmental contaminants | Fish EDC | Limited, in vitro assay development | Yes (although ultimate utility uncertain) |
| Trenbolone Acetate Metabolite Transport in Rangelands and Irrigated Pasture: Observations and Conceptual Approaches for Agro-Ecosystems | Jones, GD \| Benchetler, PV \| Tate, KW \| Kolodziej, EP | ENVIRONMENTAL SCIENCE & TECHNOLOGY \| 48 (21): 12569-12576 NOV 4 2014 | To assess the relative ecological risks of trenbolone acetate (TBA) use in agro-ecosystems, we evaluated the spatiotemporal dynamics of TBA metabolite transport during irrigation and rainfall events. Within a pasture, TBA-implanted heifers (40 mg, TBA, 8 mg estradiol) were briefly penned (24 h) at high stocking densities (500 animal units (AU)/ha), prior to irrigation. Irrigation runoff concentrations of 17 alpha-TBOH) 0.3 m downslope were 11 ng/L in the wetting front, but quickly decreased to similar to 0.5 ng/L, suggestions mass transfer limitations to transport. At 3 and 30 m downslope, efficient attenuation of 17 alpha-TBOH concentrations is best explained by infiltration and surface partitioning. At plot scales transport through vegetated filter stripes resulted in <0.5-7 ng/L 17 alpha-TBOH concentrations in rainfall-induced runoff with partial subsequent attenuation. Thus, even under intense grazing scenarios, TBA-metabolite transport potentials is expected to be low in rangelands, with ecological risks primarily arising from uncontrolled animal access to receiving waters. However, 17 alpha-TBOH concentrations in initial turnoff were predicted to exceed threshold levels (i.e., no observed adverse effect levels [NOAELs]) for manure concentrations exceeding 2.0 ng/g-dw, which occurs throughout most of the implant life. For comparison, estrone and 17 beta-estradiol were modeled and are likely capable of exceeding NOAELs by a factor of similar to 2-5 in irrigation runoff, suggesting that both endogenous and exogenous steroids contribute to endogenous exogenous steroids contributed to endocrine disruption potential in agro-ecosystems. | Residue/Monitoring Study | High | Yes, exposure assessment. |

| | | | | | | |
|---|---|---|---|---|---|---|
| Mass Balance Approaches to Characterizing the Leaching Potential of Trenbolone Acetate Metabolites in Agro-Ecosystems | Jones, GD \| Benchetler, PV \| Tate, KW \| Kolodziej, EP | ENVIRONMENTAL SCIENCE & TECHNOLOGY \| 48 (7): 3715-3723 APR 1 2014 | Several studies have documented the occurrence and fate of trenbolone acetate (TBA) metabolites in soil and water. However, considerable uncertainty still exists with respect to TBA risk in agro-ecosystems because limited data are available to quantify excretion, transformation, and leaching processes. To address these uncertainties, we used experimental mesocosms and a mass balance approach to estimate the TBA metabolite leaching potential from manure excreted by implanted (40 mg TBA, 8 mg 17 beta-estradiol) beef cattle. Manure sample analysis indicates that over 113 days, a maximum of 9.3% (3,200 mu g/animal unit [AU]) of the implant dose was excreted as 17 alpha-trenbolone (17 alpha-TBOH), and <1% was excreted as 17 beta-trenbolone (65 mu g/AU) or trendione (3 mu g/AU). While most (>97%) of the total excreted mass of 17 alpha-TBOH transforms to uncharacterized products, 0.3-0.6% (100-220 mu g/AU) of the implant dose accumulates on land surfaces and is available for subsequent transport. During rainfall or irrigation events, a maximum of 0.005-0.06% (1.6-22 mu g/AU 17 alpha-TBOH) or 0.005-0.012% (1.8-4 mu g/AU 17 alpha-TBOH) of the dose leached into runoff, respectively. Leaching potentials peak at 5-30 days postimplantation, suggesting that targeted timing of implantation and irrigation could minimize steroid leaching during rainfall and irrigation events. | Residue/Monitoring Study | High | Yes, exposure assessment. |
| In vitro assessment of transcriptional activation of the estrogen and androgen receptors of mosquitofish, Gambusia affinis affinis | Katsu, Y \| Hinago, M \| Sone, K \| Urushitani, H \| Guillette, LJ \| Iguchi, T | MOLECULAR AND CELLULAR ENDOCRINOLOGY \| 276 (1-2): 10-17 SEP 30 2007 | Sex-steroid hormones are essential for normal reproductive activity in both sexes. Estrogens are necessary for ovarian differentiation during a critical developmental stage in many vertebrates and promote the growth and differentiation of the female reproductive system. Androgens play essential roles in the development and functioning of the vertebrate male reproductive system as well as actively supporting spermatogenesis. Importantly, recent studies suggest that androgens and estrogens have important reproductive roles in both males and females. To understand the molecular mechanisms of estrogen and androgen actions and to evaluate estrogen and androgen receptor-ligand interactions in the mosquitofish, Gainbusia affinis affinis, we used degenerate primer sets and PCR techniques to isolated DNA fragments encoding estrogen receptor alpha (ER alpha; ESR1), ERbeta1 (ER beta 1) and ER beta 2 from the ovary. Full-length mosquitofish ER (mfER) cDNAs were obtained using cDNA library screening and RACE techniques. Amino acid sequences of mfERs showed over-all homology of 46% (alpha versus beta 1), 43% (alpha versus beta 2), and 52% (beta 1 versus beta 2). We applied the ERE-luciferase reporter assay system to characterize these receptors. In this transient transfection assay system using mammalian cells, the mfER proteins displayed estrogen-dependent activation of transcription. In addition to ERs, the transactivation of mosquitofish ARs (mfARs) previously isolated by our group, were examined using an androgen-responsive MMTV-luciferase assay system. Mosquitofish ARs showed androgen-dependent activation of transcription from the MMTV promoter. These data provide a basic tool allowing future studies examining the receptor-ligand interactions and endocrine disrupting mechanisms in mosquitofish and also expands our knowledge of estrogen and androgen receptor evolution. | Fish EDC | Limited, basic physiology | Yes, backgound |
| In vitro study of Organization for Economic Co-operation and Development (OECD) endocrine disruptor screening and testing methods-establishment of | Kim, TS \| Yoon, CY \| Jung, KK \| Kim, SS \| Kang, IH \| Baek, JH \| Jo, MS \| Kim, HS \| Kang, TS | JOURNAL OF TOXICOLOGICAL SCIENCES \| 35 (2): 239-243 APR 2010 | The androgen receptor (AR) binding assay can be used to determine the ability of probable endocrine disruptors (EDs) to compete with synthetic androgen methyltrienolone (R1881) for binding to recombinant rat AR (rrAR). In this study, we assessed AR binding of various chemicals using Lexius Freyberger's method. The rank of relative binding affinity (RBA, IC50) on the tested chemicals was trenbolone 1.3 x 10(-8) M (RBA 138) > dihydrotesterone (DHT) 1.8 x 10(-8) M (RBA 100) > methyl testosterone 5.7 x 10(-8) M (RBA 31.6) > nonylphenol (NP) 1.3 x 10(-5) M (RBA 0.14) > bisphenol A (BPA) 1.1 x 10(-4) M (RBA 0.016) > isobutyl paraben 3.1 x 10(-4) M (RBA 0.0058) > butyl paraben 6.2 x 10(-4) M (RBA 0.0029) > propyl paraben 9.7 x 10(-4) M (RBA 0.0019). However, di(n-butyl) phthalate (DBP) and di(2-ethylhexyl) phthalate (DEHP), known anti-androgenic chemicals, did not show any significant AR binding activity. Our data suggests that in vitro AR binding assay may be useful as a screening tool for potential EDs. | Mammalian EDC Study | Limited, in vitro method validation, Level 2 | Yes |

| | | | | | | |
|---|---|---|---|---|---|---|
| a recombinant rat androgen receptor (rrAR) binding assay | | | | | | |
| Androgenic and estrogenic activity in water bodies receiving cattle feedlot effluent in eastern Nebraska, USA | Soto, AM \| Calabro, JM \| Prechtl, NV \| Yau, AY \| Orlando, EF \| Daxenberger, A \| Kolok, AS \| Guillette, LJ \| le Bizec, B \| Lange, IG \| Sonnenschein , C | ENVIRON MENTAL HEALTH PERSPECTI VES \| 112 (3): 346-352 MAR 2004 | Studies reveal that surface waters worldwide are contaminated with hormonally active agents, many released from sewage treatment plants. Another potential source of aquatic hormonal contamination is livestock feedlot effluent. In this study, we assessed whether feedlot effluent contaminates watercourses by measuring a) total androgenic [methyltrienolone (R1881) equivalents] and estrogenic (17beta-estradiol equivalents) activity using the A-SCREEN and E-SCREEN bioassays and b) concentrations of anabolic agents via gas chromatography-mass spectroscopy and enzyme-based immunoassays. Water samples were collected over 3 years from up to six sites [all confluent with the Elkhorn River, Nebraska, USA: a feedlot retention pond (site 1), a site downstream from site 1 (site 2), a stream with intermediate livestock impact (site 3), and three sites with no observable livestock impact (sites 4-6)] and two sources of tap water. In 1999, samples from site 1 contained 9.6 pM R1881 equivalents and 1.7 pM 17beta-estradiol equivalents. Site 2 samples had estrogen levels similar to those in site 1 samples but lower androgen levels (3.8 pM R1881 equivalents). Androgen levels in site 3 samples were similar to those in site 2 samples, whereas estrogen levels decreased to 0.7 pM 17beta-estradiol equivalents. At site 6, androgen levels were approximately half those found at site 3, and estrogen levels were comparable with those at site 3. Sampling in later years was limited to fewer sites because of drought and lack of permission to access one site. Instrumental analysis revealed estrone but no significant levels of resorcylic acid lactones or trenbolone metabolites. Tap water was devoid of hormonal activity. We conclude that feedlot effluents contain sufficient levels of hormonally active agents to warrant further investigation of possible effects on aquatic ecosystem health. | Residu e/Mon itoring Study | Limited, analytical techniques not very sensitive | Yes, fate |
| In vitro and in vivo effects of 17beta-trenbolone: a feedlot effluent contaminant | Wilson VS;Lambright C;Ostby J;Gray LE; | Toxicol Sci %2002 , Dec | Concern has arisen regarding the presence and persistence of trenbolone in the environment. Trenbolone acetate is an anabolic steroid used to promote growth in beef cattle. It is hydrolyzed to the active compound, 17beta-trenbolone (TB), which is also one of the metabolites excreted by cattle. Reproductive alterations have been reported in fish living in waters receiving cattle feedlot effluent, and in vitro androgenic activity displayed by feedlot effluent samples has been related to these effects. In the current study, the androgenic potency of TB was examined both in vitro and in short-term in vivo assays. TB was a high affinity ligand for the androgen receptor (AR), with an IC(50) of about 4 nM in rat ventral prostate cytosol and about 33 nM in cells transfected with the human AR when competed with 1 nM [3H]R1881. TB induced AR-dependent gene expression in MDA-kb2 cells with a potency equal to or greater than dihydrotestosterone. In immunocytochemistry experiments with the human AR, concentrations as low as 1 pM significantly induced androgen-dependent translocation of the AR into the cell nucleus. TB also displayed antiglucocorticoid activity in vitro, inhibiting dexamethasone-induced transcriptional activity, and reduced adrenal gland size in vivo. In the Hershberger assay (in vivo), TB was as potent as testosterone propionate in tissues that lack 5alpha-reductase but less effective at increasing weight of tissues with this enzyme. Such tissue specificity was anticipated because other C-19 norsteroidal androgens display a similar profile in this assay. Subcutaneous TB treatment was about 50- to 100-fold more effective in stimulating growth of androgen-dependent tissues than was oral treatment. In our in utero screening assay, maternal TB administration increased AGD and attenuated the display of nipples in female offspring in a dose-related manner, similar to the published effects of testosterone propionate. Previous studies have documented that these types of malformations in newborn and infant rats are not only permanent effects but are also highly correlated with serious reproductive malformations as adults. In summary, TB is a potent environmental androgen both in vitro and in vivo and, in contrast to other reports, can | Mamm alian EDC Study | Limited, just rats | Yes, species sensitivity |

| | | | | | | |
|---|---|---|---|---|---|---|
| | | | induce developmental abnormalities in the fetus | | | |
| Cloning and in vitro expression and characterization of the androgen receptor and isolation of estrogen receptor alpha from the fathead minnow (Pimephales promelas) | Wilson, VS \| Cardon, MC \| Thornton, J \| Korte, JJ \| Ankley, GT \| Welch, J \| Gray, LE \| Hartig, PC | ENVIRONMENTAL SCIENCE & TECHNOLOGY \| 38 (23): 6314-6321 DEC 1 2004 | In vitro screening assays designed to identify hormone mimics or antagonists typically use mammalian (rat, human) estrogen (ER) and androgen receptors (AR). Although we know that the amino acid sequences of steroid receptors in nonmammalian vertebrates are not identical to the mammalian receptors, a great deal of uncertainty exists as to whether these differences affect interactions of potential endocrine-disrupting chemicals (EDC) with the receptors. This leads to substantial uncertainty with respect to the utility of mammalian-based screening assays to predict possible effects of EDCs in nonmammalian wildlife. This paper describes preparation of a cDNA library from a small fish model commonly used in ecological risk assessments, the fathead minnow (Pimphales promelas). The cDNA library was subsequently used to isolate and sequence both AIR and Malpha. In addition, the fathead minnow (fh)AR was expressed and characterized with respect to function using saturation and competitive binding assays in COS monkey kidney cells. Saturation experiments along with subsequent Scatchard analysis determined that the K-d of the fhAR for the potent synthetic androgen R1881 was 1.8 nM, which is comparable to that for the human AIR in the same assay system. In COS whole cell competitive binding assays, potent androgens such as dihydrotestosterone and 11-ketotestosterone were also shown to be high affinity ligands for the fhAR. We also report affinity of the receptor for a number of environmental contaminants including the AR agonists androstenedione and 17alpha-and 17beta-trenbolone;AR antagonists such as p,p'-DDE, linuron, and vinclozolin; and the ER agonist 17beta-estradiol. Future plans include comparison of binding affinities of the fhAR to those of the human AR, also expressed in COS cells, using a range of EDCs. | Fish EDC | High, cross-species comparisons of receptor binding | Yes, species sensitivity |
| Airborne particulate matter collected near beef cattle feedyards induces androgenic and estrogenic activity in vitro | Wooten, KJ \| Blackwell, BR \| McEachran, AD \| Mayer, GD \| Smith, PN | AGRICULTURE ECOSYSTEMS & ENVIRONMENT \| 203: 29-35 MAY 1 2015 | Steroid growth promoters are commonly administered to beef cattle residing on feedyards, and a portion of these compounds are excreted in manure along with endogenous steroids. Steroids associated with aerosolized particulate matter (PM) can be transported from feedyards via wind. To assess potential androgenic and estrogenic activity of PM extracts, total suspended particulate samples were collected upwind and downwind of feedyards in the Southern High Plains and subjected to in vitro transcriptional activation assays. Androgen-mediated transcriptional activation induced by exposure to extracts from PM collected downwind of feedyards was significantly higher than that induced by exposure to extracts of corresponding upwind samples, whereas estrogen-mediated transcriptional activation was detected after exposure to upwind and downwind PM sample extracts. Detection and quantitation of metabolites of the synthetic androgen trenbolone acetate downwind, and estradiol both upwind and downwind, suggest that synthetic growth promoters contribute to observed in vitro activity. No significant correlations were observed, however, between individual steroid concentrations or total androgen/estrogen concentration and in vitro activity, indicating the contributions of additional, unquantified compounds to observed androgenic and estrogenic activity. Results indicate that steroids affiliated with feedyard PM have the potential to elicit endocrine-modulating effects. | Residue/Monitoring Study | Limited, novel transport mechanism | Yes, basic fate |
