# Supplementary material for: Recommended Approaches to the Scientific Evaluation of Ecotoxicological Hazards and Risks of Endocrine-Active Substances
Source: Integr Environ Assess Manag. Author manuscript; Available in PMC 2018 Aug 1. (PMC6069525; doi:10.1002/ieam.1885)
Supplement: Supplement12 [file NIHMS1500348-supplement-Supplement12.docx]

**Supplemental Data S6**

**Draft Case Study for the Ecotoxicological Hazard and Risk assessment of Vinclozolin**

James R. Wheeler^1^, Amy Blankinship^2^, Christopher Borgert^3^, Henry Krueger^4^, Markus Hecker^5^, L. Earl Gray^6^, Nancy D. Denslow^7^

^1^ Dow AgroSciences, Abingdon, Oxfordshire, UK.

^2^ Office of Pesticide Programs, United States Environmental Protection Agency, Washington D.C., USA.

^3^ Applied Pharmacology and Toxicology, Inc., Gainesville, Florida, USA; Dept. Physiol. Sciences, CEHT, Univ. of Florida College of Veterinary Medicine, Gainesville, FL, USA.

^4^ Wildlife International, a Division of EAG, 8598 Commerce Drive, Easton, MD 21601, USA

^5^ Toxicology Centre and School of the Environment & Sustainability, University of Saskatchewan, Saskatoon, Saskatchewan, Canada

^6^ USEPA, Reproductive Toxicology Branch, Research Triangle Park, North Carolina, NC 27711, USA.

^7^ Center for Environmental and Human Toxicology, Department of Physiological Sciences, College of Veterinary Medicine, University of Florida, Gainesville, Fl, 32611, USA

1. **Background information:**
   1. **Intended Mode of Action**

Vinclozolin is an agricultural fungicide used to control various blights and rots caused by fungal pathogens (USEPA 2009). It is classified as a dicarboximide targeting signaling of the MAP/Histidine- Kinase in osmotic signal transduction (os-1, Daf1) pathways^[[1]](#footnote-1)^.

- 1. **Exposure profile and anticipated environmental exposure routes**

Based on available soil partitioning data, vinclozolin, metabolite E and 3,5-dichloroanaliline (3,5-DCA) have the potential to move from treatment sites to non-target areas via runoff and leaching. Available data indicate that vinclozolin residues of concern have the potential to be transported off site of treatment areas via volatilization. The compound may also move off-site through spray drift. Bioaccumulation is unlikely to be a concern for vinclozolin residues of concern (USEPA 2009).

1. **Physical-chemistry, fate characteristics, and estimated environmental concentrations of vinclozolin (w/ focus on formation of metabolites)**
   1. **Physicochemical and fate characteristics**

From USEPA 2009, available laboratory studies for vinclozolin indicate that it quickly degrades via hydrolysis in neutral water (half-life = 1.3 d) with its half-live strongly depending on pH ranging from 38 minutes at pH 9 to 42 days at pH 5. According the available environmental fate studies, vinclozolin has several major (≥10% of applied parent) degradates (see Table 1). In aerobic and anaerobic environments, vinclozolin breaks down via microbial degradation, with half-lives ranging between 17.6 and 352 days. Vinclozolin can also be degraded via photolysis, with half-lives between 18.1 and 27.2 days in soil and aqueous environments, respectively (see Table 2). However, as described, vinclozolin breaks down to several degradates that are of concern.

***Table S6-1. Major vinclozolin metabolites (>10% formation) as identified in environmental fate studies.***

| **Metabolite** | **Synonyms** |
| --- | --- |
| Metabolite B | 3,5-dichlorophenyl-carbamic acid- (1-carboxy-1-methyl-allyl) ester  BF 352-22 |
| Metabolite D | 3,5-dichloroaniline  BF 352-31 |
| Metabolite E | N-(3,5-dichlorophenyl)-2-hydroxy-2-methyl-3-butenoic acid-amide  BF352-23  M1 |
| Metabolite F | 2,3,4-trihydroxy-2-methylbutanoic acid-(3,5-dichloroanilide)  BF-352-25  M2 |
| Metabolite S | (N-(3,5-dichlorophenyl)-5-methyl-2,4-oxazolidinedione)  BF 352-41 |

In addition to these degradates, metabolite F (N-(3,5-dichlorophenyl)-2-methyl-2,3,4-trihydroxybutanoic acid amide also known as M2) was observed as a major degrade in an available bioconcentration study with the bluegill sunfish. There are no data available on the persistence of metabolites B, E, F and S. A limited amount of data are available to characterize the environmental fate of 3,5-DCA. It is assumed that metabolites B, E, F and S are intermediate metabolites between vinclozolin and its ultimate degradation product, 3,5-DCA.

*Table S6-2. Environmental fate half-lives relevant to vinclozolin and observed degradation products.*

| **Study** | **Vinclozolin Half-life (d)** | **Major degradates**  **(≥10% of applied)** | **Minor degradates**  **(<10% of applied)** | **Source (MRID)** |
| --- | --- | --- | --- | --- |
| Hydrolysis pH 5 pH 7 pH 9 | 41.8  1.3  0.026 (38 min) | B & E | None reported | 41471006  44025301 |
| Aqueous Photolysis | 27.2 | B & E | None reported | 42394706 |
| Soil Photolysis | 18.1 | B & S | E | 41471008  44025302 |
| Aerobic Soil Metabolism (loamy sand) | 35 | B | E and S | 135376 |
|  | 41 | B | 3,5-DCA, E and S | 135376 |
|  | 53 | B | E and S | 135376 |
|  | 53 | B | 3,5-DCA, E | 88288 |
|  | 352 | None reported | B, E, 3,5-DCA | 43013001 44025303 |
| Anaerobic Soil Metabolism | 17.6 | B | 3,5-DCA, E | 41471009 |
| Aerobic aquatic metabolism | Not available | | | |
| Anaerobic aquatic metabolism | 134 | B, 3,5-DCA | E & S | 43013002  43255801 |

- 1. **Estimated Environmental Concentrations (EECs) (for aquatic exposure and terrestrial modeling)**

According to USEPA 2009, aquatic estimated environmental concentrations (EECs) were modeled using the USEPA aquatic exposure model (PRZM/EXAMS). In addition to parent vinclozolin, metabolites B and E and 3,5-DCA, which are major degradates of vinclozolin and were considered to be residues of concern, were included in the aquatic modeling. Other identified metabolites (i.e., metabolite S, F) were not included in the modeling since their formation occurred in environmental pathways (i.e., soil photolysis, fish bioconcentration) that are not used to parameterize the aquatic modeling. Including metabolites B, E and 3,5-DCA (total residue of concern method) was considered to provide a conservative estimate of potential exposure to aquatic organisms. This approached assumed that the toxicity of degradates were equipotent as parent vinclozolin. Aquatic EECs were also calculated using just parent vinclozolin and they were an order of magnitude lower than EECs using the total residue method.

Terrestrial exposure modeling was also included in USEPA 2009 and included vinclozolin residues on terrestrial dietary items for birds and mammals.

**3. Toxicity Data Considered for the Case Study**

**3.1 Acute toxicity data (terrestrial and aquatic)**

Acute toxicity data for aquatic and terrestrial animals are available for vinclozolin (Table 3). This information may be useful to inform about potential overt/systemic toxicity that may be observed in studies with endocrine-related endpoints. Based on the data in Table 3, vinclozolin is practically non-toxic to birds and mammals on an acute basis, and no more than moderately toxic to fish and aquatic invertebrates (using the USEPA hazard classification scheme).

***Table S6-3. Acute Toxicity Data for Aquatic and Terrestrial Animals for Vinclozolin (from USEPA 2009)***

| **Species** | **Toxicity Value** | **Effect** | **Citation MRID (Author & Date)** | **USEPA Study Classification** |
| --- | --- | --- | --- | --- |
| Rainbow Trout  (*Oncorhynchus mykiss*) | LC_50_ = 2.84 mg/L (nominal) | Mortality | 264302  (Gelbke 1980) | Supplemental |
| Bluegill sunfish | LC_50_ = 47.5 mg/L (nominal) | Mortality | EPA database | -- |
| *Daphnia magna* | EC_50_ = 4.0 mg/L | Immobilization | Union Carbide 1978 | Acceptable |
| Rotifer *(Branchiorius calyciflorus)* | LC50=30.5 mg/L | Mortality | Zavala-Aquirre et al 2007 | -- |
| Northern Bobwhite Quail  (*Colinus virginianus*) | LD_50_ >2,510 mg/kg | Mortality | 92194-002  (Fink, 1978) | Acceptable |
| Northern Bobwhite Quail | LC_50_ >5,620 mg/kg diet | Mortality | 92194-003  (Fink, 1978) | Acceptable |
| Laboratory Rat  (*Rattus norvegicus*) | LD_50_ >10,000 mg/kg bw | Mortality | 921940-10  O’Reilly | Acceptable |

**3.2 Mechanistic Data (parent and metabolites)**

A total of 37 studies that characterized the potential mechanisms of vinclozolin *in vitro* were reviewed as part of this exercise. The main aim of the majority of these studies was to characterize the specific mode of action by which vinclozolin interacts with certain endocrine functions in vertebrates ranging from fish to mammals. In addition, one study focused on characterizing the formation of specific metabolites using rat liver microsomes (Sierra-Santoyo et al. 2012). In general, studies could be categorized into one of the following three types:

1. Cell-free receptor binding studies;
2. Immortalized cell lines; or
3. Primary cell or tissue culture systems.

While there was great variation in the quality of the studies reviewed, there was consensus with regard to two primary modes of action of vinclozolin *in vitro* regardless of study type. Specifically, vinclozolin was shown to act as a potent androgen receptor (AR) antagonist both in transfection assays with stable cell lines as well as in primary culture systems (e.g. Ait-Aissa et al. 2010; Jolly et al. 2009; Roy et al. 2004). Also, it was demonstrated that the metabolite M2, and to a lesser extent M1, were significantly more potent AR antagonists than the parent compound vinclozolin (Kelce et al. 1994; Wilson et al. 2007). In fact, M2 was shown to be equally, and in some cases even more, potent than the model AR antagonist flutamide (Ait-Aissa et al. 2010). Interestingly, some studies identified vinclozolin and its metabolites as weak androgen agonists at low concentrations; however, all compounds act as strong antagonists at greater concentrations (Hartig et al. 2002). The antiandrogenic mode of action of vinclozolin and its metabolites M1 and M2 could also be confirmed across different *in vitro* systems using both mammalian and fish cells. A secondary mechanism by which vinclozolin was shown interact with endocrine functions in vertebrates *in vitro* was alteration of steroidogenesis. Both human and fish cells responded to the exposure with vinclozolin by increased 17beta-estradiol (E2) production, although effects on androgen production were inconsistent with an increase in fish ovarian cells and a decrease in human H295R adrenocarcinoma cells (Villeneuve et al. 2007). Sanderson et al. (2002) hypothesized that the increase in E2 production was due to vinclozolin stimulating P450 aromatase activity. However, no comparable effect was observed in Chinese hamster (CHO) cells (Andersen et al. 2002).

The main uncertainty associated with many of the studies reviewed included the lack of determination of cell viability. However, considering that the overwhelming majority of studies reviewed showed significant AR-antagonism across taxonomic groups and tests systems there is sufficient evidence to classify vinclozolin as a potent antiandrogen *in vitro*. A full analysis of study quality can be found in the accompanying spreadsheet.

**3.3 Mammals**

In screening level assays employed in the EDSP Tier 1 Screening Battery, the literature clearly indicates that vinclozolin and/or metabolites M1 and M2 bind ARs of multiple vertebrate classes, including the rat and human AR (see above). This results in AR antagonism *in vitro* and *in vivo*. *In vitro*, vinclozolin, M1 and M2 inhibit the action of androgens. This effect has been shown in multiple cell types with different reporter-promoter constructs.

The EDSP Tier 1 Screening Battery includes two mammalian *in vivo* assays, the Hershberger Assay and Pubertal Male Rat Assay, designed to detect the endocrine activity of AR antagonists like vinclozolin. Multiple laboratories have shown that vinclozolin displays antiandrogenic activity in the Hershberger Assay; resulting in a reduction in androgen-dependent tissue weights in castrate-testosterone treated-immature male rats. Vinclozolin also displays antiandrogenic activity in the Pubertal Male Rat Assay, delaying the onset of preputial separation (a biomarker of puberty in this species) and reducing androgen-dependent tissue weights. In these two assays, the antagonist activity occurs in animals that do not display any signs of overt toxicity or reduced growth.

The above screening level assays are designed to detect AR antagonism, but are not designed to necessarily determine adverse effects or set thresholds for risk assessment. Multigenerational reproduction tests are the only test guideline protocols that cover the entire reproductive life cycle of the rat. These are intended to determine NOAELS and LOAELs for hazard identification for risk assessment. A comprehensive analysis of the reproductive life cycle in the rat involves a 10 week dosing period prior to mating with continued dosing throughout pregnancy, lactation, weaning and mating of the F1 offspring to produce an F2 generation. A number of high quality multigenerational and one generation studies have been conducted with vinclozolin. The results of these clearly demonstrate that vinclozolin disrupts androgen-dependent development *in utero* and during puberty in the male rat. The *in utero* period appears to be the most sensitive, followed by pubertal alterations and then adult exposures.

Many of the effects of vinclozolin are permanent and not apparent until the male offspring attain sexual maturity. For example, vinclozolin induced prostatitis in the male offspring does not develop until the animals are fully mature. It should be noted that standard test guideline Developmental Toxicity studies that examine near-term rat fetuses after *utero* exposure to vinclozolin are generally negative unless specific androgen-dependent endpoints are added.

Male rat offspring, exposed *in utero* to vinclozolin display a postnatal phenotype that is remarkably similar to the drug flutamide. Flutamide, the pharmaceutical anti-androgen, being about 10-15 fold more potent than vinclozolin *in utero*. At lower dosage levels (3 to 12 mg/kg/d) males may display reduced anogenital distance (AGD) and reduced ventral prostate weight. As the dosage level increases more androgen-dependent tissues are affected including the levator-ani bulbocavernosus muscle, and seminal vesicle which are smaller or in some cases there is complete agenesis of the organ. At higher dose levels (50 to 100 mg/kg/d), some male offspring display hypospadias and testis non descent. In contrast, the vas deferens, epididymis and testis (if normally descended) are less affected at these higher dosage levels.

A review of the literature on chemicals that display AR antagonism, including vinclozolin and flutamide demonstrate that the dose response curves are monotonic. Some endpoints like AGD appear linear in low dose range (with no clear threshold) whereas other effects like hypospadias and undescended testes are clearly non-linear and the dose response curves display very steep slope factors, implying a threshold.

**3.4 Birds**

There is a limited number of publications describing the effects of vinclozolin on avian species. However, during the development of the Avian Two-Generation Toxicity Test in the Japanese quail, vinclozolin was used in three laboratories as a validation substance. There were no treatment-related mortalities, overt signs of toxicity or significant treatment-related effects upon body weight, feed consumption, egg content weight, and levels of testosterone or estradiol in egg yolks in the parental and first filial generations at any of the concentrations tested. There were no significant treatment-related effects upon the development of secondary sex characteristics in the first filial generation birds, or mating behavior in the first or second filial generation birds. Additionally, there were no significant treatment-related effects upon any of the reproductive parameters, blood plasma hormone levels or thyroid weights measured at the 64, 160, 400 or 1000 ppm (mg/kg-diet) test concentrations across the three generations. Results from histological evaluations of tissues identified a potential treatment-related effect in the epididymides of adult male birds in the F0 and F1 generations. However, the potential effect observed at the histological level was not concentration responsive and did not result in subsequent reductions in egg fertility or overall reproductive success across the F0 and F1 generations of birds. The no-observed-effect concentration for Japanese quail exposed to vinclozolin in the diet during the study was 1000 ppm, the highest concentration tested.

One open literature citation(Selzsam et al. 2003), included endocrine specific endpoints to a standard avian reproduction study (analogous to OECD TG 206). Test concentrations were nominally untreated control, 125, and 500 ppm (mg/kg-diet) (125, and 483 ppm measured). The study demonstrated maternal transfer of vinclozolin to egg at 483 ppm, a weak impact on spermatogenesis based on histology of testes which also was confirmed by low spermatid count. Additional endpoints included measurement of estradiol, testosterone, T3, T4, Progesterone (females only). In agreement with the Japanese quail studies there was no reproductive effects. Regulatory mallard duck and bobwhite quail reproduction studies (standard data requirements for pesticides) were available for vinclozolin. In the mallard study hatching rate was affected at 250 ppm due to higher embryonic mortality and reduced hatching success. In the bobwhite the laying rate was diminished at 125 ppm (NOEAC = 50 ppm) and an increase in embryonic deaths resulted in a lower hatching rate.

The lack of effects on the mechanistic and apical endpoints of reproduction at realistic environmental concentrations does not provide convincing evidence of endocrine disruption in birds.

**3.5 Invertebrates**

While there are limited data for invertebrates with respect to endocrine-specific endpoints, compared to vertebrates, studies are available for partial life-cycles (for crustaceans and mollusks) as well as a multi-generational study (mysid shrimp, USEPA 2013). Information on mechanistic data for invertebrates, as for vertebrates, did not appear to be available which limits the ability to evaluate whether reported effects are due to an endocrine-mediated process. In general, most measured reproductive endpoints were apical, and in most cases, endpoints were for fecundity (number of offspring/eggs). Other reproductive endpoints measured included sex ratio, fertilization, and abnormal eggs/offspring. Several studies reported no reproductive effects. In cases with reported apical reproductive effects, often these results may be influenced by other factors as other potentially non-endocrine–relevant endpoints were also affected (i.e., survival and growth). Additionally, in general, there was limited information in regards to measured concentrations (particularly in regards to metabolite formation when nominal values were not maintained or measured). Also, there was limited information on environmental conditions as well as only testing one concentration in some cases which further complicated evaluation. In some cases, effects on survival or other types of clinical toxicity were not reported or it wasn’t clear if reproductive effects may have occurred in the presence of overt or systemic toxicity (reported mortality effects not clearly defined). In these cases, it is difficult to evaluate whether any reproductive effect may have been due to an endocrine-mediated event or due to systemic or overt toxicity.

A short discussion of studies with a Klimish score of 1 or 2 is provided here. Studies with terrestrial invertebrates, isopods (*Porcellio scaber*), were conducted using a formulation, which limits the utility of this data given the uncertainty of the influence of the other ingredients in the formulation (Lemos et al. 2009, 2010a, b). In a study using the freshwater gastropod, *Lymnaea stagnalis*, adult snails were exposed to vinclozolin at concentrations from 3.8 - 92.2 ng/L; there were no effects on parental survival or growth (Giusti et al. 2014). There were no effects observed on mean number of eggs or egg clutches per individual or percent of abnormal eggs. While decreases in mean number of eggs per clutch and increases of percent of polyembryonic eggs were observed, these effects did not follow a dose-response pattern. In a multi-generational reproduction study with mysid shrimp (*Americamysis bahia*), many different reproductive endpoints were measured including number of young per female, time to brood release, time to maturation (USEPA 2013). However, there were no other endocrine-mediated endpoints measured such as biochemical markers or gonadal histopathology. Mysids were exposed to vinclozolin at concentrations from 8.2 - 403 µg a.i./L. There were no statistically significant effects (using an α of 0.05) for most endpoints. At the highest treatment group, there were significant trends (but not a statistically significant result using a pair-wise test) for some endpoints including a decreasing trend for survival and dry weight in the F0 generation, and an increasing trend in the number of days to brood release and percent females. In the F0 generation, there was a statistically significant increase in male/female ratio at the highest treatment group (no observed adverse effect concentration (NOAEC) = 120 µg/L). In the F1 generation, at the highest concentration, there was a decreasing trend in the number of offspring, and an increasing trend in body length at all concentrations. In a one-generation Daphnia magna reproduction study, at 1400 µg/L, there were decreases in parental growth (length and weight) and in the number of young/adult; the overall NOAEC value was 790 µg/L (USEPA 2009).

In regards to other endocrine-mediated endpoints other than fecundity, sex ratio, and fertilization, in a study of the ramshead snail (*Marisa cornuarietis*) Tillmann et al. (2001) report that juvenile snails exposed to vinclozolin for 5 months showed a significant albeit transient effect on penis length and penis sheath at 0.03 and 1.0 µg/L during the first couple of months of the study. By the fourth month of the study, there was no difference between vinclozolin-treated and control animals. However, there were many uncertainties associated with this study including: 1) the identity of the vinclozolin used (technical or formulation); 2) did not use a negative control; 3) did not measure test concentrations; 4) appears to have only used one replicate so may have used psuedoreplicates for statistical analysis. Furthermore, the study is confounded by the fact that neither the treated nor control animals spawned and it is difficult to determine the relevance of the decreased penis length and sheath to the reproductive success of the test animals.

While effects on apical reproductive endpoints were observed in some studies, there were generally no other endpoints measured which would be used to inform whether these effects were endocrine-mediated or not. Furthermore, since there is generally limited knowledge of the endocrine systems of invertebrates it is not possible to conclude whether there is an *a priori* expectation that vinclozolin will interact with invertebrate endocrine pathways.

**3.6 Fish**

There are both screening level and definitive full lifecycle studies in fish. These indicate that in adult fish, vinclozolin appears to be working as an antiandrogen, but at relatively high concentrations. However, there does not appear to be a consistent pattern of adverse effects in the higher tier lifecycle studies.

Martinovic et al (2008) demonstrated a decrease in cumulative egg number for reproductive pairs of fathead minnows exposed to aqueous preparations of vinclozolin (no vehicle carrier added to exposures) for all concentrations tested including 60, 255, and 450 µg/L using the 21 day reproductive study. Plasma vitellogenin in females was elevated at the two highest concentrations suggesting that females were making vitellogenin but that this was not being further incorporated into eggs and the eggs were not being spawned. There were minor effects on secondary sex characteristics in males but only at the highest concentrations tested, suggesting perhaps that vinclozolin was not having a direct effect on AR. No histopathology was performed, so there was no information on whether there was an effect on sperm production. A follow up study by Martinovic et al (2011) with short term exposures of zebrafish (24, 48 and 96 h) to vinclozolin (567 - 632 µg/L) were performed for microarray analysis. Gene expression patterns were comparable with flutamide expression and were consistent with an anti-androgen mode of action.

In medaka (Kisparisis 2003) at higher concentrations (2,500 ug/L nominal concentration), there was evidence for delay of maturation of spermatocytes. Other effects in fish have also been measured that suggest anti-androgen action of nominally 250 µg/L vinclozolin on spiggin production in female three-spinned stickleback females co-exposed with androgens.

However, it should be noted that all these screening level studies were only scored a classification 3 with the ToxRtool. Therefore, the reliability of these effects should be treated with caution regarding regulatory application for hazard or risk assessment. A higher scoring Fish Short-term Reproduction Test (FSTRT) was available but it was not a full study.

In terms of higher tier fish tests, vinclozolin has been used in several lifecyle studies with zebrafish, fathead minnow and medaka. The first embryo to reproduction study with zebrafish was considered unreliable (ToxRtool class 3) due to a semi-static design, no chemical analysis, measurement of environmental parameters or appropriate control treatment. However, some evidence of reproductive and sex ratio effects was observed. However, again these should be treated with caution due to the overall study reliability and issues with certain endpoints (e.g. sex ratio determination).

Vinclozolin was also tested in a fathead minnow full lifecycle test with the additional of endocrine endpoints. Parent and the B and E metabolites were monitored. Reproductive effects were observed at the highest test concentration (150 µg/L) consistent with retarded male and female gonad development. Sex ratio data were highly variable but might suggest an increase in females also at 150 µg/L. The study was conducted for registration purposes and was assumed to have scored a ToxRtool classification of 1 (it was accepted by regulatory authorities).

Vinclozolin was used as a reference substance for the inter-laboratory validation of the test that was to become the Medaka Extended One Generation Test (MEOGRT; OECD conceptual framework level 5). Five concentrations ranging from 8 to 360 µg/L (mean measured) were tested in 3 laboratories. Surprisingly there was no effect on fecundity or fertility. Further, there was limited evidence of the expected anti-androgenic effect (some evidence for decrease anal fin papillary processes). Therefore, there is some uncertainty as to whether the effects observed in zebrafish and fathead minnow are not expressed in medaka over this range of test concentrations. Alternatively, there may have been differences in the amount of metabolites formed in the test system that could potentially be a contributing factor. These studies were also assumed to be reliable.

Overall, the fish data is consistent with an anti-androgen mode of action but only at high concentrations. However, there is some considerable uncertainty to demonstrate a causal link between the assumed endocrine mode-of-action and the effects observed in the OECD conceptual framework level 5 tests.

1. **Risk Assessment Characterization**

**4.1 Exposure characterization**

It was beyond the scope of this exercise to fully characterize the exposure to vinclozolin. However, modelling has been conducted both in the US and EU for the agricultural uses. Here we present this modelling (aquatic and terrestrial) performed by the USEPA (2009). Exposure modelling for pesticides is a well-established science and regulatory process in which there is high confidence. Therefore, it was agreed that this approach would be suitable for any potential risk assessment.

4.1.1 Aquatic modeling

Based on USEPA 2009, the peak one-in-ten year aquatic EEC for vinclozolin residues of concern is 52.0 µg/L. One-in-ten year 21-d and 60-d EECs were 51.1 and 49.9 µg/L, respectively.

4.1.2 Terrestrial modeling

Also, based on the application rates used in USEPA 2009, terrestrial dietary EECs were provided for birds and mammals:

***Table S6-4. Terrestrial Dietary EECs for Birds and Mammals for Vinclozolin***

| **EEC Description** | **Value** | **Unit** |
| --- | --- | --- |
| Dietary Based EEC for birds | 454 | ppm |
| Dose based EEC for small mammals (15g eating short grass) | 770 | mg/kg-bw |
| Contact EEC for small insect (prey) | 454 | Ppm |
| Contact EEC for large insect (prey) | 50 | Ppm |

4.1.3 Monitoring data (surface water for California only)

No California-specific water monitoring data are available for vinclozolin or metabolites B, E , F or S; however, data are available for one of its metabolites of concern, 3,5-DCA, from the United States Geological Survey’s (USGS) National Water Quality Assessment (NAWQA). No data are available in the California Department of Pesticide Regulations Surface Water Database for vinclozolin or 3,5-DCA. 3,5-DCA was detected in 1.3% of 308 surface water samples collected from 2001-2009 in CA. The maximum reported concentration of 3,5-DCA was 0.0268 µg/L. The level of quantification of 3,5-DCA ranged 0.004 - 0.012 µg/L (USGS 2009 in USEPA 2009).

**4.2 Comparison of toxicity to exposure**

4.2.1 Aquatic Animals

Effects on fish endpoints were generally seen at concentrations of 150 µg/L and above, which is greater than the chronic (21 and 60 day) EECs (and even the peak EEC (52 µg/L) reported in USEPA 2009. Effects on reproduction in aquatic invertebrates, mysid and daphids, were also reported at concentrations greater than the peak EEC. Following US-EPA ecological risk assessment approaches this would give a risk quotient (RQ; exposure value divided by toxicity value) of 0.3 which is less than the level of concern (LOC=1), therefore the potential for adverse effects (risk) is likely low.

4.2.2 Terrestrial Animals

For birds, vinclozolin did not appear to affect Japanese quail reproduction or offspring viability in a two-generation study at dietary concentrations up to 1000 ppm which is greater than the reported dietary EECs (USEPA 2009). In other species tested for regulatory purposes, mallard ducks and bobwhite quail, effects on fecundity and/or offspring viability were reported at ≥ 125 ppm which is lower than the dietary EECs (454 ppm) reported in USEPA 2009. However, in these studies, apical endpoints are examined (fecundity, offspring viability) which may be the result of multiple factors that may not necessarily be solely endocrine-mediated. In the study by Selzsam (2001), while maternal transfer to the eggs and effects on spermatogenesis were observed at 483 ppm (roughly equivalent to the EECs), no effects on reproduction were observed. Further, exposure refinement of EECs (for instances using empirically measured residues on treated foliage) could be possible to determine if acceptable risk could be demonstrated.

Effects of vinclozolin exposure on mammals (rats) provided the strongest indication of a potential anti-androgen effect of vinclozolin. Impacts to androgen-dependent reproductive tissues/organs have been observed across several studies (at doses of ≥3 mg/kg/d) with non-descending testes at doses of 50 mg/kg/d and higher.

However, exposure refinement of EECs (for instances using measured residues) would be possible to determine if acceptable risk could be demonstrated. Such approaches, though common in the risk assessment for pesticides, was out of the scope of this exercise.

- 1. **Conclusions**

Mechanistic *in vitro* information demonstrate that vinclozolin and its metabolites can interact with the androgen receptor as an antagonist. However, anti-androgen effects *in vivo* across taxa, particularly in higher-level studies that examine effects on apical endpoints such as reproduction is less clear. Effects are most prominent in the mammalian rat studies. The risk assessment for mammals would need further exposure refinement to investigate if acceptable risk can be demonstrated

- 1. **References**

Ait-Aissa S, Laskowski S, Laville N, Porcher JM, Brion F. 2010. Anti-androgenic activities of environmental pesticides in the mda-kb2 reporter cell line. *Toxicol Vitro* 24:1979-1985.

Andersen H, Vinggaard A, Rasmussen T, Gjermandsen I, Bonefeld-Jorgensen E. 2002. Effects of currently used pesticides in assays for estrogenicity, androgenicity, and aromatase activity *in vitro*. *Toxicol Appl Pharmacol* 179:1-12.

Giusti A, Lagadic L, Barsi A, Thome JP, Joaquim-Justo C, Ducrot V. 2014. Investigating apical adverse effects of four endocrine active substances in the freshwater gastropod *Lymnaea stagnalis*. *Sci Total Environ* 493:147-155.

Hartig PC, Bobseine KL, Britt BH, Cardon MC, Lambright CR, Wilson VS, et al. 2002. Development of two androgen receptor assays using adenoviral transduction of mmtv-luc reporter and/or har for endocrine screening. *Toxicol Sci* 66:82-90.

Jolly C, Katsiadaki I, Morris S, Le Belle N, Dufour S, Mayer I, et al. 2009. Detection of the anti-androgenic effect of endocrine disrupting environmental contaminants using *in vivo* and *in vitro* assays in the three-spined stickleback. *Aquat Toxicol* 92:228-239.

Kelce WR, Monosson E, Gamcsik MP, Laws SC, Gray LE. 1994. Environmental hormone disruptors - evidence that vinclozolin developmental toxicity is mediated by antiandrogenic metabolites. *Toxicol Appl Pharmacol* 126:276-285.

Lemos MFL, van Gestel CAM, Soares A. 2009. Endocrine disruption in a terrestrial isopod under exposure to bisphenol a and vinclozolin. *J Soils Sediments* 9:492-500.

Lemos MFL, van Gestel CAM, Soares A. 2010a. Developmental toxicity of endocrine disrupters bisphenol a and vinclozolin in a terrestrial isopod. *Arch Environ Contam Toxicol* 59:274-281.

Lemos MFL, van Gestel CAM, Soares A. 2010b. Reproductive toxicity of the endocrine disrupters vinclozolin and bisphenol a in the terrestrial isopod *Porcellio scaber* (latreille, 1804). *Chemosphere* 78:907-913.

Martinovic-Weigelt D, Wang RL, Villeneuve DL, Bencic DC, Lazorchak J, Ankley GT. 2011. Gene expression profiling of the androgen receptor antagonists flutamide and vinclozolin in zebrafish (*Danio rerio*) gonads. *Aquat Toxicol* 101:447-458.

Martinovic D, Blake LS, Durhan EJ, Greene KJ, Kahl MD, Jensen KM, et al. 2008. Reproductive toxicity of vinclozolin in the fathead minnow: Confirming an anti-androgenic mode of action. *Environ Toxicol and Chem* 27:478-488.

Roy P, Salminen H, Koskimies P, Simola J, Smeds A, Saukko P, et al. 2004. Screening of some anti-androgenic endocrine disruptors using a recombinant cell-based *in vitro* bioassay. *J Steroid Biochem Mol Biol* 88:157-166.

Sanderson J, Boerma J, Lansbergen G, Berg Mvd. 2002. Induction and inhibition of aromatase (cyp19) activity by various classes of pesticides in h295r human adrenocortical carcinoma cells. *Toxicol Appl Pharmacol* 182:44-54.

Selzsam B, Niemann L, Haider W, Gericke C, Chahoud I. 2003. High dose effects of the pesticide vinclozolin on fertility and reproduction in the male Japanese quail (*Coturnix coturnix japonica*). Naunyn-Schmiedebergs *Arch Pharmacol* 367:R167-R167.

Sierra-Santoyo A, Angeles-Soto E, Lopez-Gonzalez MD, Harrison RA, Hughes MF. 2012. *In vitro* metabolism of the anti-androgenic fungicide vinclozolin by rat liver microsomes. *Arch Toxicol* 86:413-421.

Tillmann M, Schulte-Oehlmann U, Duft M, Markert B, Oehlmann J. 2001. Effects of endocrine disruptors on prosobranch snails (mollusca : Gastropoda) in the laboratory. Part iii: Cyproterone acetate and vinclozolin as antiandrogens. *Ecotoxicology* 10:373-388.

Villeneuve D, Ankley G, Makynen E, Blake L, Greene K, Higley E, et al. 2007. Comparison of fathead minnow ovary explant and h295r cell-based steroidogenesis assays for identifying endocrine-active chemicals. *Ecotox Environ Saf* 68(1): 20-32.

United States Environmental Protection Agency. 2009. Risks of vinclozolin use to federally threatened California red-legged frog (*Rana aurora draytonii*). Environmental Fate and Effects Division. Office of Pesticide Programs. Washington, D.C. October 14, 2009.

United States Environmental Protection Agency. 2013. Endocrine Disruptor Screening Program (EDSP) Tier 2 Ecotoxocity Tests. Scientific Advisory Panel Meeting. June 25-28, 2013. EPA-HQ-OPP-2013-0182.

Wilson VS, Cardon MC, Gray LE, Hartig PC. 2007. Competitive binding comparison of endocrine-disrupting compounds to recombinant androgen receptor from fathead minnow, rainbow trout, and human. *Environ Toxicol and Chem* 26:1793-1802.

Attachment #1


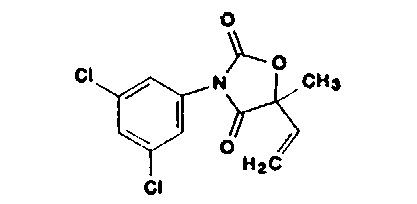


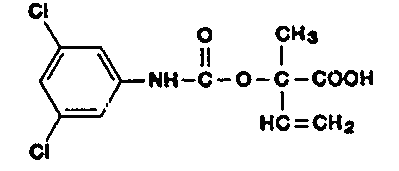


Vinclozolin Metabolite B


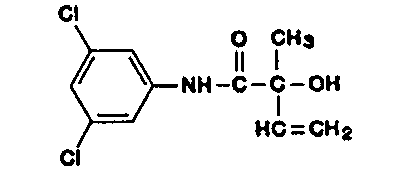


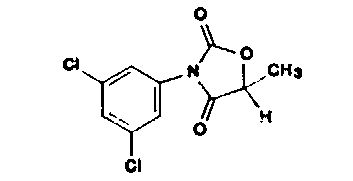


Metabolite E Metabolite S


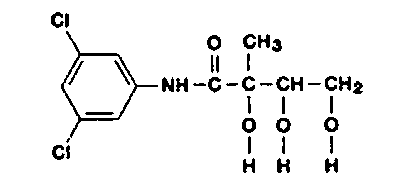


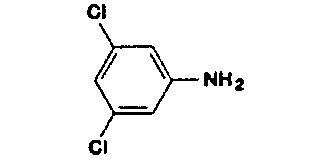


Metabolite F 3,5-DCA

**Figure S6-1. Structures of vinclozolin residues of concern.**

1. http://www.frac.info/docs/default-source/publications/frac-code-list/frac-code-list-2015-finalC2AD7AA36764.pdf?sfvrsn=4 [↑](#footnote-ref-1)
