# Supplementary material for: Recommended Approaches to the Scientific Evaluation of Ecotoxicological Hazards and Risks of Endocrine-Active Substances
Source: Integr Environ Assess Manag. Author manuscript; Available in PMC 2018 Aug 1. (PMC6069525; doi:10.1002/ieam.1885)
Supplement: Supplement14 [file NIHMS1500348-supplement-Supplement14.docx]

**Supplemental Data S7**

**Methods used to put the draft case studies together**

Annegaaike Leopold^1^, Mike Roberts^2^, Peter Matthiessen^3^, Jenny Odum^4^, Gary Ankley^5^, Patrick D. Guiney^6^, Henrik Holbech^7^, Tom Hutchinson^8^, Taisen Iguchi^9^, Karen A. Kidd^10^, Anapuma Kumar^11^, Laurent Lagadic^12^, Ellen Mihaich^13^, Lisa Ortego^14^, Bruce Vigon^15^, Lennart Weltje^16^, James R. Wheeler^17^

^1^ Caldris Environment BV, Bergkampweg 1, Warnsveld, 7231 CL, The Netherlands

^2^ Independent Consultant, 50 Princes Road, Burnham-on-Crouch, Essex CM0 8BX, UK

^3^ Independent Consultant, Dolfan Barn, Beulah, Llanwrtyd Wells, Powys LD5 4UE, UK

^4^ Regulatory Science Associates, One Eastwood, Harry Weston Road, Binley Business Park, Coventry CV3 2UB, UK

^5^ US Environmental Protection Agency, 6201 Congdon Blvd, Duluth, MN 55804, USA

^6^ Molecular & Environmental Toxicology Center, University of Wisconsin, Madison 1010B McArdle Bldg., 1400 University Avenue, Madison, WI 53706, USA

^7^ Department of Biology, University of Southern Denmark, Campusvej 55, 5230 Odense M., Denmark

^8^ School of Biological Sciences, University of Plymouth, Drake Circus, Plymouth, Devon PL4 8AA, UK

^9^ National Institute for Basic Biology, 5-1 Higashiyama, Myodaiji, Okazaki 444-8787, Japan

^10^ Biology Department, University of New Brunswick, 100 Tucker Park Road, Saint John NB, Canada, E2L 4L5

^11^ CSIRO, Private Mail Bay 2, Glen Osmond, South Australia, SA 5064, Australia

^12^ Bayer AG, Crop Science Division, Environmental Safety, Ecotoxicology, Alfred-Nobel-Straße 50, 40789 Monheim am Rhein, Germany

^13^ Environmental and Regulatory Resources, 6807 Lipscomb Dr., Durham, NC 27712, USA

^14^ Bayer CropScience, 2 TW Alexander Drive, Research Triangle Park, NC 27709, USA

^15^ Society of Environmental Toxicology and Chemistry, 229 South Baylen Street, 2nd Floor, Pensacola, FL 32502, USA

^16^ BASF SE, Ecotoxicology, Speyerer Strasse 2, Limburgerhof, Rheinland-Pfalz, D-67117, Germany

^17^ Dow AgroSciences, Abingdon, Oxfordshire, UK

**Data compilation**

Hazard and risk assessment requires the assembly/generation of different types of relevant data in order to make informed decisions. This involves an understanding of which study types and data are useful, the sources from which they may be obtained, the reliability of the data, their organisation and assessment using a weight of evidence (WoE) approach. This may then be followed by a decision-making strategy as to whether the adverse effects could be considered “endocrine disruption” according to the WHO/IPCS (2002) definition. For risk assessment, further characterisation of exposure is required and should include an evaluation of the environmental fate and bioavailability of the chemical being evaluated.

Guidance is available for all parts of the data-gathering process. However, selection of the most appropriate guidance may depend upon the regulatory jurisdiction and the legislative framework e.g. EFSA (2013) in the EU; USEPA (2011) in the USA and EXTEND (2010) in Japan. Exposure should also be assessed as part of the process of risk assessment (JRC, 2016; ECHA, 2016a &b; USEPA, 2016a). In all cases, a transparent approach should be undertaken in the context of the assessment process and ultimately the conclusions.

The Organisation for Economic Co-operation and Development (OECD) and its member countries have been working for more than 15 years on the development and validation of methods for screening and testing chemicals for endocrine disruption under the umbrella of the Advisory Group on Endocrine Disrupters Testing and Assessment (EDTA AG). Many OECD Test Guidelines (TGs) specific to endocrine activity/disruption have been validated and adopted under the EDTA. In addition, a large number of technical guidance documents, validation reports, and workshop reports have been published in support of the TGs and to assist in interpretation and assessment of results. Furthermore, the USEPA has an endocrine disruptor screening program (EDSP) for which endocrine-specific assays similar to those under EDTA were also adopted. These organisations operate in parallel, as the US is a member country of OECD.

The assays for EASs are largely contained within the OECD Conceptual Framework (CF) for Testing and Assessment of Endocrine Disrupters (OECD, 2012a). The CF represents a “toolbox” of methods and includes both TG assays and tests that are “standardised” but are not formally accepted as TGs. The organisation of the CF is valuable for both identifying those assays that will contribute to the hazard/risk assessment and for organising the data once it has been obtained (for examples, see Hutchinson *et al.*, 2013; Juberg *et al.*, 2013). The OECD CF is organized into five levels based on the nature and complexity of the assays, as well as the types of endpoints captured (e.g., mechanistic versus apical) (Table 1). Many of the assays used by the USEPA and Japanese assays for assessing endocrine disruption are represented in the CF. Some standard regulatory (eco)toxicology tests that have endocrine-related endpoints are also included in the OECD CF.

**Table S7 - 1. Levels of the OECD Conceptual Framework for Testing and Assessment of Endocrine Disrupters.**

| Level 1 | Existing Data and Non-Test Information |
| --- | --- |
| Level 2 | *In vitro* assays providing data about selected endocrine mechanism(s) / pathways(s). Applicable to both mammalian and non-mammalian species. |
| Level 3 | *In vivo* assays providing data about selected endocrine mechanism(s) / pathway(s). Some assays may also provide some evidence of adverse effects. Subdivided into mammalian and non-mammalian assays. |
| Level 4 | *In vivo* assays providing data on adverse effects on endocrine relevant endpoints. Effects can be sensitive to more than one mechanism and may be due to non-endocrine mechanisms. Subdivided into mammalian and non-mammalian assays. |
| Level 5 | *In vivo* assays providing more comprehensive data on adverse effects on endocrine relevant endpoints over more extensive parts of the life cycle of the organism. Effects can be sensitive to more than one mechanism and may be due to non-endocrine mechanisms. Subdivided into mammalian and non-mammalian assays. |

At present the assays in the OECD CF and USEPA EDSP focus on facets of pathways involved in estrogen, androgen and thyroid signalling. However, it is recognised that many others endocrine pathways of possible concern exist, for example interactions with the retinoid X receptor appears to be the basis for the the MoA for tributyltin in invertebrates . Additional vertebrate endocrine pathways, possible testing methods for them, and recent activities on endocrine-oriented tests in invertebrates are provided in OECD 2012b & 2016. However, adverse effects are still likely to still be captured using current TGs with endocrine regulated/mediated apical endpoints, regardless of the MoA of the substance.

OECD Guidance Document (GD) 150 on Standardised Test Guidelines for Evaluating Chemicals for Endocrine Disruption (OECD, 2012b) provides support to practitioners and regulatory authorities using or evaluating tests for endocrine disruption. It provides guidance on data selection, endocrine and apical endpoints, use of the tests, how to interpret the outcome of individual tests, and how to increase evidence on whether or not a substance may be an EDS.

Studies considered suitable for providing information relevant for assessment of interaction with endocrine systems include analysis of functional measures of the status of endocrine systems of concern. Measurements may be focused on endocrine organs, and endpoints that may be affected by hormonal changes. Studies including these endpoints comprise short-term, long-term, carcinogenicity, reproduction, and mechanistic studies in many species (OECD CF levels 3-5). *In vitro* studies on endocrine endpoints and molecular interactions also provide valuable MoA data to help interpret *in vivo* results (OECD CF level 2). OECD GD 150 also advises using all available data, including information on structural analogues, in vitro data from high throughput screening programs such as ToxCast and EDSP21(USEPA, 2016b) and *in silico* models (e.g. McRobb *et al*., 2014 a,b).

Exposure assessment practises are already embedded within specific legislation and there are extensive exposure studies on the diverse environmental inputs of some potential EDSs entering aquatic and terrestrial environments. It is necessary to understand the environmental fate and bioavailability of a chemical across different compartments of ecosystems to predict environmental concentrations. These predicted concentrations provide a more realistic context for the interpretation of hazard.

**Assessment of data**

Relevance and reliability of data should be addressed. Globally, different chemical legislations already require assessment and use of relevant and reliable published literature. Relevance is usually assessed first, often at the point of acquiring abstracts from a literature search. Reliability is then assessed for only those papers/reports that are considered relevant.

There are several methods available for addressing reliability. The procedures developed in for this workshop are suitable for the assessment of EASs. Another recent SETAC Pellston^TM^ workshop has addressed the issues specific to the use of open literature data for environmental risk assessment and regulatory decision making (Moermond et al, 2016).

There has been some debate about the use of studies conducted according to Good Laboratory Practice (GLP) and standardised test guidelines, compared with non-standard or non-GLP literature data (Zoeller *et al.*, 2015), but all information used should be scientifically robust. Essentially any information that is deemed scientifically relevant and reliable should be included in the evaluation.

Once the information has been assessed for relevance and reliability, then a WoE assessment should be carried out. A framework may be used for this, or expert judgement without a framework, but accepting this is less transparent. Several methods have been published but the choice may depend upon whether there is a regulatory requirement for the assessment and guidance for doing this. In general, no single test is likely to provide conclusive evidence about the endocrine disrupting potential of a substance. Table 2 provides a summary of some of the published approaches to WoE assessment for EAS/EDSs and their attributes and uncertainties.

**Table S7 - 2. Some weight of evidence approaches for assessment of endocrine effects.**

| **Reference** | **Comments** |
| --- | --- |
| CEFIC – EMSG (2010) | - Guidance for human health and wildlife. Addresses the issues of data relevance, quality and significance - using a WoE. Indicates whether, and what action needs to be taken in order to assess the hazards and risks of a substance. - Slightly outdated. Some more recent assays are missing. |
| Bars *et al*. (2011) | - Output from ECETOC workshop. - Suggests scientific criteria for the determination of endocrine disrupting properties that integrate information from both regulatory (eco)toxicity studies and mechanistic/screening studies. - The criteria suggested are designed for EU regulatory requirements but the paper also discusses US approach. |
| Borgert *et al*. (2011) | - WoE approach for USEPA EDSP but relevant generally. - Suggests hypothesis testing with quantitative weightings for endpoints to give a WoE score and a narrative developed to clearly describe the final determinations. |
| USEPA (2011) | - Suggested WoE approach for USEPA assessment of EDSP Tier 1 studies and need for Tier 2. - Conclusions regarding the potential of a substance to interact with the estrogenic, androgenic or thyroidal hormonal pathways. Uses alignment table of endpoints from all studies across taxa. |
| OECD (2012b) | - Guidance on use of OECD CF, assays, endpoints, data collection etc. - Uses all data, applicable across regulatory areas. - Case study example in Juberg *et al.* (2013) |
| EFSA (2013) | - Provides opinion on criteria, test methods and critical aspects. Uses WHO definition. - An ED is defined by 3 criteria: i) an adverse effect in an intact organism or a (sub)population; ii) an endocrine activity; and iii) a plausible causal relationship between the two. |
| Weltje *et al*. (2013) | - Update to Bars *et al*. (2012) with a focus on ecotoxicology. |
| Borgert *et al*. (2014) | - Follow-up to 2011 paper with detailed rationale for weighting the EDSP endpoints. - Output from expert panel (Endocrine Policy Forum). - Case study example in de Peyster and Mihaich (2014) |
| van Der Kraak *et al*. (2014) | - Quantitative WoE approach used for evaluation of atrazine in fish, amphibians, and reptiles. - All studies scored for relevance of response to adverse outcomes and strength of methods. |
| Lutter *et al*. (2015) | - Review of WoE approaches in literature. Some discussion of USEPA and ECHA approaches. - Not specific for EDs, no decision making tools. |
| Becker et al. (2015) | - Use the Bradford-Hill considerations of biological plausibility, empirical support (dose-response, temporality & incidence) and essentiality in building AOPs. OECD approach. - WoE evaluations and case studies. |
| Christiansen et al. (2015) | - Information/testing strategy for identification of substances with endocrine disrupting properties in the EU. Suggests information/testing strategies for adequate identification of EDs. Based on OECD GD 150 and OECD Fish toxicity framework (OECD STA 171) |
| DK EPA (2011) | - A scientific WoE approach to the establishment of Criteria for Endocrine Disruptors and Options for Regulation in the EU (REACH, PPPR, BPR). |

One approach that is incorporated into many of the WoE processes cited above and the OECD CF, is the concept of adverse outcome pathways (AOPs). AOPs are analytical constructs that describe a sequential chain of causally-linked events at different levels of biological organisation that lead to an adverse health or ecotoxicological effect (see Ankley et al., 2010; OECD 2016). In the context of a WoE analysis, an AOP could provide a basis for identifying regulatory data needs and supporting test interpretation. AOPs are available, or an AOP can be constructed, for the linkage between a substance acting via a known molecular initiating event (MIE), such as activation of the estrogen receptor, and adverse “downstream” consequences (e.g., altered sexual differentiation). Since the linkages between the MIE and subsequent key events leading to adverse outcome are causal in nature, the basic construct directly informs WoE analyses. An example of this type of AOP-based WoE analysis for the effects of inhibition of sex steroid synthesis (aromatase activity) on reproduction in fish is described by Becker et al. (2015). Development of AOPs are a focus for future regulatory data needs

An integrated pathway-based exposure assessment (i.e., the spacing between the concentrations that can cause an effect in the laboratory and measured or predicted levels in the environment) can provide information as to whether or not the substance is likely to interact with an endocrine target. It may also enable a conclusion as to whether the substance is an EDS according to the WHO definition, i.e., whether it alters function(s) of the endocrine system and consequently causes adverse health effects in an intact organism, or its progeny, or (sub) populations. In the environmental health assessment this explicitly means adverse effects at the population level, in line with protection goals (Weltje et al 2013).

**Approach taken for case studies**

The case study groups made use of the approaches described above. The workflow followed by the case study groups is shown in Figure S7-1. The exact methods used are described in detail in the relevant SI documents (S1 to S6). The method selected for each case study varied according to the substance and the issues associated with it. For example, the 17 beta-ethinylestradiol (EE2) focused on fish only, as there were too many papers (over 15,000) to review otherwise.

The literature search terms, numbers of references retrieved and the papers used in the case studies are shown in the corresponding SI documents. Most case study groups made use of the ToxRTool (Schneider et al, 2009) for assessment of reliability, then organised relevant and reliable studies/endpoints in accordance with the OECD Conceptual Framework. All groups then performed a weight of evidence evaluation. The outcome of the case studies, when compared together, provided many examples of cross-cutting, data availability and interpretation issues, typically common to several substances, which may have an impact on decision making.

**Figure S7-1 . Workflow followed by the case studies.**

**References**

Ankley, G. T., Bennett, R. S., Erickson, R. J., Hoff, D. J., Hornung, M. W., Johnson, R. D., Mount, D. R., Nichols, J. W., Russom, C. L., Schmieder, P. K., Serrrano, J. A., Tietge, J. E., and Villeneuve, D. L. (2010). Adverse outcome pathways: a conceptual framework to support ecotoxicology research and risk assessment. Environ. Toxicol. Chem. 29(3), 730-741.

Bars, R., Broeckaert, F., Fegert, I., Gross, M., Hallmark, N., Kedwards, T., Lewis, D., O'Hagan, S., Panter, G. H., Weltje, L., Weyers, A., Wheeler, J. R., and Galay-Burgos, M. (2011). Science based guidance for the assessment of endocrine disrupting properties of chemicals. Regul. Toxicol Pharmacol. 59(1), 37-46.

Becker, R. A., Ankley, G. T., Edwards, S. W., Kennedy, S. W., Linkov, I., Meek, B., Sachana, M., Segner, H., Van Der Burg, B., Villeneuve, D. L., Watanabe, H., and Barton-Maclaren, T. S. (2015). Increasing Scientific Confidence in Adverse Outcome Pathways: Application of Tailored Bradford-Hill Considerations for Evaluating Weight of Evidence. Regul. Toxicol. Pharmacol. 72(3), 514-537.

Borgert, C. J., Mihaich, E. M., Ortego, L. S., Bentley, K. S., Holmes, C. M., Levine, S. L., and Becker, R. A. (2011). Hypothesis-driven weight of evidence framework for evaluating data within the US EPA's Endocrine Disruptor Screening Program. Regul. Toxicol. Pharmacol. 61(2), 185-191.

Borgert, C. J., Stuchal, L. D., Mihaich, E. M., Becker, R. A., Bentley, K. S., Brausch, J. M., Coady, K., Geter, D. R., Gordon, E., Guiney, P. D., Hess, F., Holmes, C. M., LeBaron, M. J., Levine, S., Marty, S., Mukhi, S., Neal, B. H., Ortego, L. S., Saltmiras, D. A., Snajdr, S., Staveley, J., and Tobia, A. (2014). Relevance weighting of tier 1 endocrine screening endpoints by rank order. Birth Defects Res. B Dev. Reprod. Toxicol. 101(1), 90-113.

CEFIC (2010). CEFIC EMSG Paper: Towards the Establishment of a Weight of Evidence Approach to Prioritizing Action in Relation to Endocrine Disruption. OECD (2010b). Workshop report on OECD countries activities and management of endocrine disrupters. OECD Series on Testing and Assessment no. 118. Organisation for Economic Cooperation and Development, Paris. 83 pp. + appendices. In Part 2, page 172.

S. Christiansen, H. Holbech, P. Bjerregaard, U. Hass (2015). Information/testing strategies for identification of substances with endocrine disrupting properties Toxicology Letters, Volume 238, Issue 2, Supplement, 16 October, Page S41-105

DK EPA (2011). Establishment of Criteria for Endocrine Disruptors and Options for Regulation, Annex A: Report on Criteria for Endocrine disrupters, Danish Centre on Endocrine Disrupters. Danish Centre on Endocrine Disrupters. http://eng.mst.dk/media/mst/Attachments/DKEDcriteria110517_finalcorr1.pdf

ECHA (2016a) [REACH, exposure assessment guidance]. Available at http://echa.europa.eu/documents/10162/13632/information_requirements_r16_en.pdf and http://echa.europa.eu/documents/10162/13564/ir_csr_r16_draft_peg_v30_en.pdf

EFSA (2013). Scientific Opinion on the hazard assessment of endocrine disruptors: Scientific criteria for identification of endocrine disruptors and appropriateness of existing test methods for assessing effects mediated by these substances on human health and the environment. EFSA Journal, 11, 3132.

EXTEND (2010). Japanese Ministry of the Environment’s program on endocrine disruption “EXTEND2010” available at https://www.env.go.jp/en/chemi/ed/extend2010_full.pdf

Hutchinson TH, Odum J and Gourmelon A (2013). Application of the OECD Conceptual Framework for Assessing the Human Health and Ecological Effects of Endocrine Disrupters. In “Endocrine Disrupters: Hazard Testing and Assessment Methods” Ed.P Matthiessen. Pub. Wiley & Sons, Hoboken. pp. 341-372.

JRC (2016) [ FOCUS exposure assessment for Ground water and surface water in Europe in support of PPP regs 1107/2009] http://esdac.jrc.ec.europa.eu/projects/focus-dg-sante

Juberg, D. R., Gehen, S. C., Coady, K. K., LeBaron, M. J., Kramer, V. J., Lu, H., and Marty, M. S. (2013). Chlorpyrifos: weight of evidence evaluation of potential interaction with the estrogen, androgen, or thyroid pathways. Regul. Toxicol. Pharmacol. 66(3), 249-263

Lutter, R., Abbott, L., Becker, R., Borgert, C., Bradley, A., Charnley, G., Dudley, S., Felsot, A., Golden, N., Gray, G., Juberg, D., Mitchell, M., Rachman, N., Rhomberg, L., Solomon, K., Sundlof, S., and Willett, K. (2015). Improving weight of evidence approaches to chemical evaluations. Risk Anal. 35(2), 186-192.

McRobb, F. M., Kufareva, I., and Abagyan, R. (2014a). In silico identification and pharmacological evaluation of novel endocrine disrupting chemicals that act via the ligand-binding domain of the estrogen receptor alpha. Toxicol. Sci. 141(1), 188-197.

McRobb, F. M., Sahagun, V., Kufareva, I., and Abagyan, R. (2014b). In silico analysis of the conservation of human toxicity and endocrine disruption targets in aquatic species. Environ. Sci. Technol. 48(3), 1964-1972.

Moermond CTA, Beasley A, Breton R, Junghans M, Laskowski R, Solomon KR, Zahner H. (2016). Improving the regulatory evaluation of ecotoxicity studies I: Assessing reliability. Integrated Environmental Assessment and Management - Submitted.

OECD (2012a). OECD Conceptual Framework for Testing and Assessment of Endocrine Disrupters. http://www.oecd.org OECD (2012). Guidance Document on Standardised Test Guidelines for Evaluating Chemicals for Endocrine Disruption. OECD Series on Testing and Assessment no. 150 Available at http://www.oecd.org/env/ehs/testing/OECD%20Conceptual%20Framework%20for%20Testing%20and%20Assessment%20of%20Endocrine%20Disrupters%20for%20the%20public%20website.pdf

OECD (2012b). Guidance Document on Standardised Test Guidelines for Evaluating Chemicals for Endocrine Disruption. No. 150. 2012. OECD Environmental Health and Safety Publications, Series on Testing and Assessment. Available at http://www.oecd.org/officialdocuments/publicdisplaydocumentpdf/?cote=env/jm/mono%282012%2922&doclanguage=en

OECD (2016). OECD Work Related to Endocrine Disrupters. Available at http://www.oecd.org/env/ehs/testing/oecdworkrelatedtoendocrinedisrupters.htm#CONCEPTUAL

Schneider K, Schwarz M, Burkholder I, Kopp-Schneider A, Edler L, Kinsner-Ovaskainen A, Hartung T, Hoffmann S. (2009). ToxRTool, a new tool to assess the reliability of toxicological data. Toxicology Letters (Amsterdam) 189:138-144.

USEPA (2011a). Endocrine Disruptor Screening Program. Weight-of-Evidence: Evaluating Results of EDSP Tier 1. Screening to Identify the Need for Tier 2 Testing. Available at http://www.regulations.gov/#!documentDetail;D=EPA-HQ-OPPT-2010-0877-0021

USEPA (2016a). EPA-Expo-Box (A Toolbox for Exposure Assessors). Available at http://www.epa.gov/expobox

USEPA (2016b). Toxcast assay results. Available at http://actor.epa.gov/dashboard/#chemical/57966-95-7

Van Der Kraak GJ, Hosmer AJ, Hanson ML, Kloas W, Solomon KR. (2014). Effects of atrazine in fish, amphibians, and reptiles: An analysis based on quantitative weight of evidence. Critical Reviews in Toxicology 44(S5):1-66.

Weltje, L., Wheeler, J. R., Weyers, A., and Galay-Burgos, M. (2013). Refinement of the ECETOC approach to identify endocrine disrupting properties of chemicals in ecotoxicology. Toxicol. Lett. 223(3), 291-294.

WHO/IPCS (2002). Global Assessment of the State of the Science of Endocrine Disruptors. Damstra, T., Barlow, S., Bergman, A., Kavlock, R. and Van Der Kraak, G. (eds). World Health Organisation, Geneva. Publication no. WHO/PCS/EDC/02.2.

Zoeller, R. T., Bergman, A., Becher, G., Bjerregaard, P., Bornman, R., Brandt, I., Iguchi, T., Jobling, S., Kidd, K. A., Kortenkamp, A., Skakkebaek, N. E., Toppari, J., and Vandenberg, L. N. (2015). A path forward in the debate over health impacts of endocrine disrupting chemicals. Environ. Health 14(1), 118.
