# Supplementary material for: Recommended Approaches to the Scientific Evaluation of Ecotoxicological Hazards and Risks of Endocrine-Active Substances
Source: Integr Environ Assess Manag. Author manuscript; Available in PMC 2018 Aug 1. (PMC6069525; doi:10.1002/ieam.1885)
Supplement: Supplement3 — S3 — Perchlorate Table S2–4 [file NIHMS1500348-supplement-Supplement3.docx]

**Supplemental Data S2**

**Draft Case Study for the Ecotoxicological Hazard and Risk Assessment of Perchlorate**

S. Marty^1^, J. Chambers^2^, L. Constantine^3^, W. Kloas^4^, A. Kumar^5^, D Pickford^6^, T. Verslycke^7^, Kunihiko Yamazaki^8^

^1^ The Dow Chemical Company, Midland, MI, USA

^2^ Mississippi State University, Mississippi State, MS, USA

^3^ Pfizer, Inc., New York City, NY, USA

^4^ Leibniz-Institute of Freshwater Ecology and Inland Fisheries, Berlin, Germany

^5^ The Commonwealth Scientific and Industrial Research Organization (CSIRO), Adelaide, S. Australia

^6^ Syngenta UK Ltd., Cambridge, United Kingdom

^7^ Gradient, Cambridge, MA, USA

^8^ Department of Environmental Health, Ministry of the Environment, Tokyo, Japan

**1. Introduction**

Perchlorate was used as a case study chemical to evaluate whether hazard-based or risk-based assessment should be conducted with this ‘well studied’ thyroid toxicant. Workshop participants also were charged with identifying critical factors that were important when considering a risk- vs. hazard-based assessment. This case study is not intended to be a full risk assessment, but rather, a brief discussion of some critical factors to consider when making this decision.

**2. Background Information (Brief Hazard and risk discussion for Endocrine Disruptors)**

**2.1 Some Uses of Perchlorate**

Perchlorate salts have been used as propellants for rockets and missiles, in matches, munition, pyrotechnics, and automobile airbag ignitions. It also has been used in the production of paints and enamels, lubricating oils and electroplating. Perchlorate also is used in medicine to manage hyperthyroidism and amiodarone thyroiditis.

**2.2 Perchlorate as a Thyroid Hazard**

The thyroid gland is the primary target organ of perchlorate toxicity. Perchlorate ion (ClO_4_^-^) is a similar size to iodide and thus, competes with iodide for active transport into the thyroid by the sodium-iodide symporter (NIS). Iodide is an essential component of the thyroid hormones, triiodothyronine (T3) and thyroxine (T4). This inhibition of iodide uptake results in decreased synthesis and release of T3 and T4 from the thyroid gland. As blood levels of thyroid hormones decrease, the thyroid gland is stimulated in an effort to maintain thyroid hormone homeostasis. This occurs when the hypothalamic-pituitary-thyroid (HPT) axis is activated *via* thyroid releasing hormone (TRH) from the hypothalamus and thyroid stimulating hormone (TSH) from the pituitary. TSH stimulates the thyroid to increase thyroid hormone synthesis and release, producing signs of thyroid activation, which can be seen histopathologically as follicular cell hypertrophy and decreased colloid (due to increased uptake of thyroid hormone from the available pool). With perchlorate, homeostatic levels of T3 and T4 are difficult to achieve, because of insufficient levels of iodide for thyroid hormone synthesis. If TSH stimulation is more protracted, follicular cell hyperplasia (and subsequent thyroid tumors) may result (Wolff, 1998; Saito et al., 1983). Therefore, processes directly dependent upon appropriate levels of the thyroid hormones (e.g., development) can be adversely impacted by perchlorate, resulting in a spectrum of effects from developmental delays to altered development.

**3. Methods**

**3.1 Literature Search and Reference Selection**

A literature search was conducted, which included regulatory and publicly available literature. Search terms were designed to capture perchlorate references that included toxicity, endocrine, thyroid, thyroid hormone/triiodothyronine (T3)/thyroxine (T4), and thyroid stimulating hormone (TSH). The initial search identified approximately 6,945 references.

The subsequent selection of references was focused on: 1) Reproductive/developmental toxicity studies, because development is a sensitive life stage for thyroid hormone insufficiency and these studies included exposure during the critical/sensitive window for effects. When available, published guideline or “guideline like” studies were used as these studies included accepted study designs, sufficient sample sizes and often were used for regulatory purposes; 2) Studies that looked at factors potentially related to population level effects (i.e., effects on survival, reproduction, and growth/development - motor activity, etc.); and 3) Studies that examined perchlorate mode of action (MOA) to confirm similar MOAs across species. Studies were selected to represent a variety of taxa/species. ToxCast data also were included in the analysis. Within relevant study types, not all available studies were used; representative studies were selected to make data evaluation more manageable. This approach is is consistent with the goal of this exercise as a proof of concept, not a full risk assessment. During workshop discussions, some additional references were gathered to expand available information in some areas (e.g., exposure).

Human studies (including clinical and epidemiology studies) were not included in the current assessment. Also, studies had to be publicly available.

**3.2 Quality Evaluation of Relevant data**

The selected reference papers were evaluated using the ToxRTool, a simple Excel-based system derived from the widely-used Klimisch method (Klimisch et al., 1997, Schneider et al., 2009). References that were judged to be a Klimisch 1 and 2 were used in the analysis, but references that were given a Klimisch score of 3 or 4 were not used; this approach is consistent to the methods used for REACH assessments. If a reviewer had difficulty applying the ToxRTool to some study types, professional judgement was used to assign Klimisch scores for study quality.

**3.3 Discuss methodology assessing the data**

Unlike compounds whose activity is unknown, thyroid perturbations by perchlorate are well documented; therefore, a formalized weight of evidence to determine endocrine activity was not needed. Studies judged to be relevant and reliable were evaluated to characterize endocrine effects in accordance with the the OECD conceptual framework for Endocrine Disruptors (OECD, 2012). Each reviewer conducted a weight of evidence analysis by taxa and sensitive endpoints/life stages.

For exposure assessment, predicted exposure concentration (PEC) values were primarily derived from government agency reports on perchlorate.

To the extent possible, data were considered in a manner similar to the procedure outlined in the OECD Conceptual Framework (OECD, 2012) levels (i.e., Level 1 – Physical-chemical structure; Level 2 – *in vitro* assays providing data about selected endocrine mechanisms/pathways (e.g. ToxCast data); Level 3 – *in vivo* data assessing specific endocrine pathways; Level 4 – *in vivo* data on endocrine-relevant endpoints and adverse effects; and Level 5 – *in vivo* data providing more comprehensive information on reproduction, development and other endocrine-relevant endpoints.

**4. Physical-Chemistry, Fate Characteristics, and Estimated Environmental Concentrations of Perchlorate (Level 1)**

**4.1 Physico-chemical Properties and Fate**

Perchlorate is the ionic form (ClO_4_^-^) of a number of salts, including potassium, sodium, and ammonium perchlorate (NH_2_ClO_4_). Due to the high solubility of perchlorate salts and the ubiquitous environmental distribution of their associated cations (Na^+^, K^+^, NH_4_^+^), this toxicological review assumes that the contribution from the cations to overall toxicity is negligible. This may not be true for all aquatic (or semi-aquatic, e.g. amphibian) species.

Perchlorate is persistent in the environment.

**4.2 Metabolism**

Perchlorate is an inorganic anion; therefore, metabolism is not relevant to its toxicity.

**4.3 Potential Exposure Routes and Taxa Affected**

Perchlorate is water soluble and persistent and can be found in many lakes and rivers.

Thus, the primary route for perchlorate exposure is via water, either surface waters (i.e., fish, amphibians, etc.) or drinking water (i.e., terrestrial mammals, birds, etc.). Based on the exposure route, exposure potential and available data, the primary taxa that were considered in this assessment included fish (e.g., stickleback, eastern mosquitofish), frogs (e.g., Xenopus laevis), birds (e.g., quail), and mammals (e.g., rodents as surrogates for other terrestrial species).

**5. *In vitro* Perchlorate Data (Level 2)**

**5.1 ToxCast Data:**

ToxCast (<https://actor.epa.gov/dashboard/>) was searched in December 2015 for the bioactivity profile of perchlorate. The graphs below (Figure S2-1) indicate that positive results were only seen for nuclear receptor assays (ammonium perchlorate on the left; potassium perchlorate on the right).

Figure S2-1


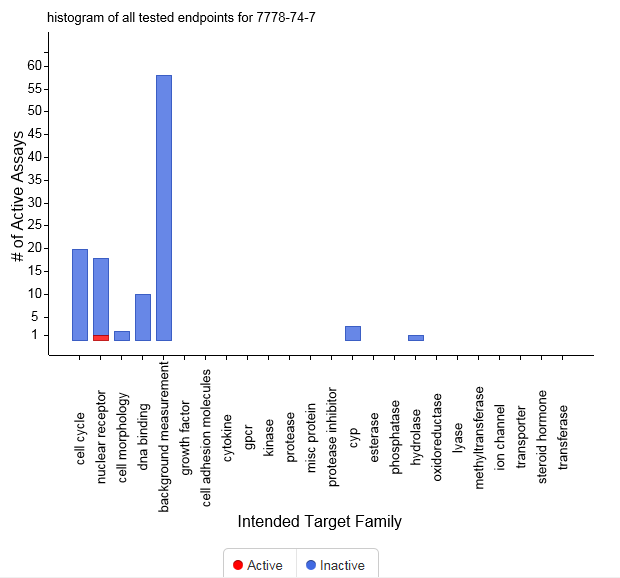

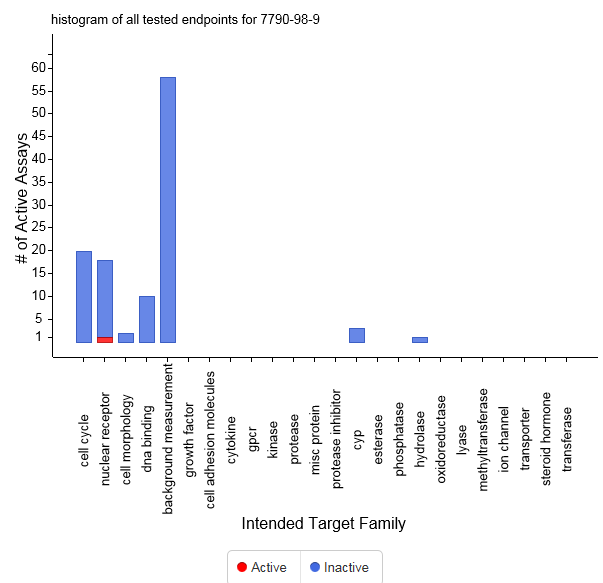


However, it is important to note that perchlorate has not been evaluated across all available ToxCast endocrine-sensitive assays. The endocrine assessment with perchlorate was limited to Tox21 endocrine assays. Both ammonium perchlorate and potassium perchlorate (graphs shown above) were positive in the ER-alpha agonist assay, Tox21_ERa _LUC_ BG1_agonist assay; however, this activity was only seen at concentrations above the cytotoxicity limit (Figure S2-2 below, showing data for ammonium perchlorate) and therefore, may not be specific.

Figure S2-2


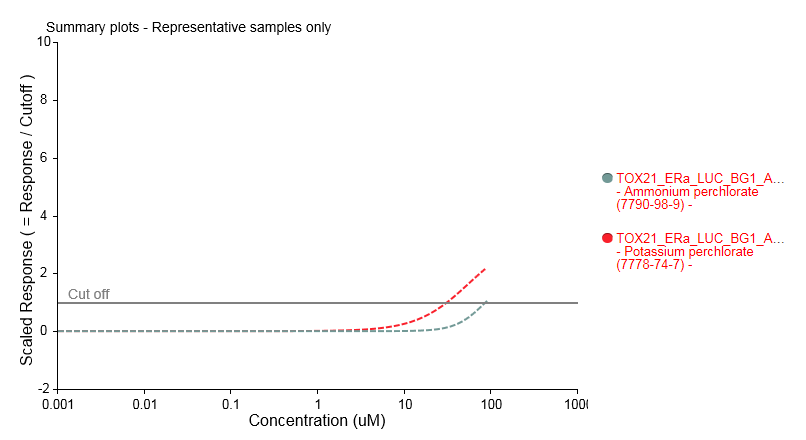


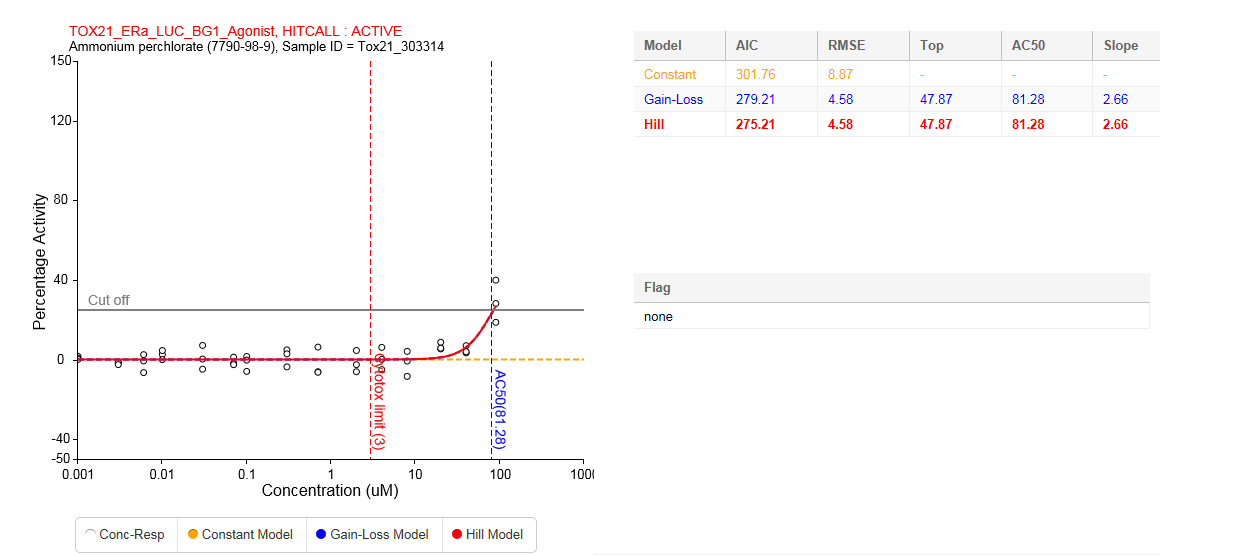


Ammonium and potassium perchlorate were negative in the other ER-alpha reporter gene assays for agonism and negative in both ER-alpha antagonist assays. There were no other positive assays identified across two AR reporter gene agonist assays, two AR reporter gene antagonist assays, one TR reporter gene agonist assay and one TR reporter gene antagonist assay.

At this time, there is no assay in ToxCast to evaluate sodium-iodide symporter functionality and iodide transport.

**6. *In vivo* Perchlorate Data Assessing Specific Endocrine Pathways (Level 3)**

**6.1 Amphibian Metamorphosis Assay (AMA):**

The AMA is level 3 in the OECD Conceptual Framework; therefore, any studies similar in design and including AMA development were included in level 3. For perchlorate, this includes the studies by Tietge et al (2005, 2010). Similarly, the Goleman et al. (2002, 2006) studies should be included here; while the study design is different (i.e., longer exposure, from shortly after fertilization), the endpoints are the similar and are informative on the same mechanisms/pathways.

**7.0 *In Vivo* Assays Providing Data on Adverse Effects on Endocrine Relevant Endpoints (Level 4):**

Level 4 assays are potentially sensitive to a variety of MOAs for endocrine disruption. These studies include repeated-dose toxicity studies with endocrine-sensitive endpoints, studies that examine a portion of the life cycle (e.g., developmental stages, sexual development, and reproduction studies. This level included the majority of perchlorate *in vivo* studies across taxa.

**8.0 *In Vivo* Assays Providing More Comprehensive Data on Adverse Effects on Endocrine Relevant Endpoints (Level 5):**

Level 5 tests provide comprehensive data on adverse effects on endocrine-relevant endpoints over more extensive parts of the life cycle of the organism. This level includes full life cycle tests, two-generation studies and extended one-generation reproductive toxicity studies. Studies in Level 5 are the most informative for endocrine effects, but across taxa, these studies were limited due to their large, complex nature.

A weight of evidence (WOE) assessment was used to examine the potential endocrine activity of perchlorate. As a proof of concept, a WOE for the estrogen, androgen, and thyroid pathways in mammals appears in Appendix 1. Note that the evaluation of potential effects on steroidogenesis was not done separately, but was included as an increase/decrease in estrogen, androgen or thyroid signaling in the respective evaluations. As stated above, thyroid perturbations by perchlorate are well documented. Therefore, WOE assessments across the other taxa focused on thyroid, the relevant target organ for perchlorate toxicity.

**7. Weight of Evidence Assessment for Endocrine Activity:**

**7.1 Estrogen Pathway (Including Disruption of Steroidogenesis for Estrogen Signaling)**

In a weight of evidence assessment, there was no evidence of direct interaction of perchlorate with the estrogen pathway in the *in vitro* and *in vivo* studies examined.

**7.2 Androgen Pathway (Including Disruption of Steroidogenesis for Androgen Signaling)**

**7.2.1 Does the chemical exhibit the potential for interaction with the androgen pathway *in vitro* or *in vivo*?** In a weight of evidence assessment, there was limited evidence to indicate an interaction of perchlorate with the androgen signaling pathway in the *in vitro* and *in vivo* studies examined. In one study, perchlorate exposure during development of stickleback caused abnormal growth and proliferation in the thyroid, gonads and kidney (Petersen et al., 2015). Gonadal development was altered (increase in average spermatogenic stage in adult testes) with increased dose. The majority of exposed adults had advanced or complete spermatogenesis (with 100 ppm adults having 1.5x testes area) versus controls that were in early stages. In juveniles, no changes in testes stages with dose were observed; however, testes area were 4x at 100 ppm versus controls. An increase in the number of early stage ovarian follicles with dose in both juvenile and adults was observed. Exposure of stickleback embryos to 10 ppm sodium perchlorate for few hours [0 days post-fertilization (dpf)] resulted in elevated 11-keto-testosterone (11-KT) levels in the embryos. Over 5 days dpf of development, the greatest effect was on elevated 11-KT levels (3-6X) versus controls, which was observed at 10 and 30 ppm. The pattern weakened through 28 days and after 28 dpf, no measureable effects on whole body 11-KT concentrations were observed at any dose. Kidney size increased significantly in adults at 10 and 100 ppm supporting androgenic effects of perchlorate as kidney is the site for spiggin production (glue protein for nest building) in male stickleback and considered a reproductive organ. Effects on whole body thyroid hormone levels appeared to follow a horseshoe-shaped dose response curve, while responses of the gonad to perchlorate follow standard linear dose response curve. The authors concluded that the different response curves further suggest perchlorate independently affects multiple pathways.

There was no evidence for perchlorate interaction with the androgen pathway in papers reviewed for other species. Effects on testicular development have been reported in male rats when marked hypothyroidism is induced from birth to postnatal day 25 (Cooke and Meisami, 1991); however, there was no indication of this effect in the perchlorate studies that were reviewed.

**7.2.2 Does the chemical exhibit adverse effects potentially mediated by androgen pathway?** Based on the studies reviewed, there were no adverse population-level effects that indicated a toxicologically signficant alteration in androgen signaling. In fish studies, apical endpoints that would indicate an adverse ‘population relevant’ effect associated with increased 11-ketotestosterone (11-KT) were not assessed. Higher concentrations of perchlorate may have potential effects on cross-talk with the androgen signaling pathway, but if present, these effects occur secondary to significant thyroid perturbations.

**7.2.3 Are the adverse effects observed in higher tiered tests corroborated by lower tiered tests and can they be concluded to be a consequence of endocrine activity?** Vertebrate development may be affected via thyroid mechanisms that cross-talk to other endocrine pathways such as the androgen-signaling pathway. Additional testing of more components of the hypothalamic-pituitary-gonadal (HPT) axis are needed (e.g., pituitary hormones, thyroid hormone binding proteins, local changes in T4/T3 ratios). Further experiments also are needed to clarify the mechanism responsible for changes in embryonic 11-KT levels.

**7.3 Thyroid Pathway**

**7.3.1 Does the chemical exhibit the potential for interaction with the thyroid pathway *in vitro* or *in vivo*?** Perchlorate interacts with the thyroid signaling pathway through a recognized MOA (i.e., interfering with iodide uptake by the sodium-iodide symporter, resulting in decreased thyroidal iodide levels and decreased hormone synthesis).

**7.3.2 Does the chemical exhibit adverse effects potentially mediated by thyroid pathway?**

Yes, population-level adverse effects have been identified in perchlorate studies. The most sensitive adverse effects by taxa appear in Table S-2.

**7.3.3 Are the adverse effects observed in higher tiered tests corroborated by lower tiered tests and can they be concluded to be a consequence of endocrine activity?**

Yes. There is sufficient evidence supporting an endocrine MOA for perchlorate. Both mechanistic data showing alterations in NIS iodide transport, biomarker-related effects on thyroid signaling (Table S-1) and adverse effects (Table S-2) have been demonstrated with perchlorate exposure.

**8.0 Derivation of No effect levels per taxa being assesse**d

**8.1 Consider ‘endocrine’ effects relative to overall no observed effect levels (NOEL) and lowest observed effect levels (LOEL). Also, are endocrine effects based on non-population endpoints (e.g. vitellogenin, gonad histopathology) or on population-level parameters that are indicative of endocrine disruption (e.g. change in sex ratios)?**

It is important to distinguish biomarkers of exposure from biomarkers that indicate adverse, population-relevant effects. On example is thyroid histopathology, which has been reported across taxa with perchlorate exposure. Thyroid histopathology can serves as a biomarker of perchlorate exposure, indicating altered thyroid signaling, but there are no population effects directly attributed to thyroid histopathology. For some endpoints, population impacts are not well understood, which makes this determination difficult.

- Thyroid histopathology is a sensitive biomarker of exposure for thyroid-active compounds. Thyroid histopathology changes may be adaptive initially (activation of HPT) and subsequently become more marked (e.g., follicular cell hyperplasia possibly progressing to thyroid tumors). However, thyroid histopathology generally would not affect population-relevant endpoints.
- Frogs are a sensitive species for thyroid perturbations, because tadpole metamorphosis is linked to the presence of sufficient levels of thyroid hormone during the proper developmental stages. Thus, altered thyroid histopathology can serve as a biomarker of altered thyroid signaling which, when coupled with an adverse effect like significantly delayed or arrested metamorphosis, indicates an endocrine finding that is considered to have population relevance.
- Zebrafish also showed thyroid histopathology at low concentrations similar to those affecting Xenopus laevis thyroid histopathology; however, a corresponding thyroid-related adverse effect has not been definitively identified.
- The use of thyroid histopathology as a biomarker of exposure is context dependent. For thyroid mimicking agents, assays with pre-metamorphic tadpoles should include metamorphosis endpoints, because thyroid histopathology is less sensitive to alterations with this MOA.

**Table S-1. Thyroid Histology as a Biomarker of Exposure**

| Taxa | Perchlorate | Estimated Conc. (LOEC) | NOEC |
| --- | --- | --- | --- |
| Rat | Ammonium perchlorate | 13.5 mg/L^a^ | 1.35 mg/L |
| Xenopus | Sodium perchlorate | 16 µg/L | --- |
| Zebrafish | Ammonium perchlorate | 11 µg/L^b^ | --- |
| Zebrafish | Potassium perchlorate | 250 µg/L | 125 µg/L |
| Quail | Ammonium perchlorate | 120 µg/L | 12 µg/L |

^a^ Estimated based on dose delivered, estimated body weight and estimated water intake in Sprague-Dawley rats.

^b^ Paper sent to Jeff Wolfe for evaluation of histopathology

**Table S-2. Thyroid Adverse Effects Across Taxa in Ecotoxicity Studies^1^**

| Taxa | Perchlorate | “Adverse” Effect | Estimated Conc. (LOEC) | NOEC |
| --- | --- | --- | --- | --- |
| Xenopus | Sodium perchlorate | Devtl stage | 250 ug/L | 62.5 ug/L |
| Zebrafish | Potassium perchlorate | Condition factor | 125 ug/L | 62.5 ug/L |
| Quail | Ammonium perchlorate | Growth (decreased tibia/femur length) | 4000 mg/L | 2000 mg/L |
| Quail | Ammonium perchlorate | Growth (decreased thyroid weight) | 500 mg/L | 250 mg/L |

^1^ The papers reviewed had very limited data on invertebrates; however, a recent Japanese Risk Assessment (2011) confirms that invertebrates are considerably less sensitive to perchlorate.

**9.0 Risk Assessment:**

**9.1 Estimated Environmental Concentrations**

There is variability in the levels of perchlorate in the environment with some “hot spots” (e.g., near military bases). Generally, perchlorate levels in surface waters (not near hot spots) are <1-5 µg/L. Background Iodide levels also can modulate the effects of perchlorate; therefore, it is useful if water/food samples in an area include an assessment of iodide. Below are some environmental monitoring studies for perchlorate:

- - In a US Geological Survey (Kalkhoff et al., 2010), samples were collected from 171 sites on rivers and streams during the summer and early fall of 2004. Samples were collected from surface-water sites in 19 states. Perchlorate was detected in samples collected in 15 states and was detected in 34 of 182 samples from rivers and streams at concentrations equal to or greater than 0.4 micrograms per liter. Perchlorate concentrations were 1.0 micrograms per liter or greater in surface-water samples from seven states. Only one surface-water had concentrations greater than 5.0 micrograms per liter. Perchlo­rate concentrations in follow-up samples collected from 1 to 3 months after the initial sample were unchanged at four of five stream sites.
  - In other US reports with exposure data, 147 samples yielded perchlorate levels of 10.5 ppm-130 ppm for surface water at six US locations of concern (Dean et al., 2004). Urbansky (1998) provided values of < 0.1 mg/L perchlorate away from sites of concern, but up to 3700 ppm in ground water (highest) in “hot spots”.
  - Perchlorate levels of 4-16 ppb were reported at Lake Mead in Nevada (Kucharzyk et al., 2009).
  - The Japanese Ministry of Environment (2011) reported that surface water has < 1 ug/L perchlorate, whereas the ambient surface water high concentration is 17 ug/L (not at “hot spots”). Tap water reportedly has a geometric mean of 0.53 µg/L with a maximum level of 4.4 µg/L. The Tone River is reportedly in the 10-20 ug/L perchlorate. Values of 44-1500 ug/L were reported for other rivers in Japan when sampled below effluent.

**9.2 Risk Assessment (Based on “Non Hot Spot” Areas)**

For risk assessment, a constant exposure is assumed with estimated exposures at < 1-5 µg/L (not near “hot spots”). The identified No Observed Effect Concentration (NOEC) based on available data is 62.5 µg/L based on effects in Xenopus laevis and zebrafish. However, there were several sources of uncertainty identified in this review (e.g., species, duration and timing of exposure, additional stressors or mitigating factors such as iodide). Using a 10X uncertainty factor for factors such as lab-to-field variability, the predicted no effect concentration (PNEC) is 6.15 µg/L. Thus, typical surface water concentrations are below the PNEC and would not be expected to produce adverse endocrine-mediated effects on wildlife.

**10.0 Sources of Uncertainty/Points to Consider for Risk Assessment**

When considering risk assessment, it is important to evaluate the uncertainties in the available data. Uncertainties my arise from several factors, including study limitations and biological variability. Some examples are listed below.

- Difficulty with husbandry conditions may indicate delayed metamorphosis in control frogs (lower proportion of controls metamorphosing in the expected time frame, resulting in reduced sensitivity).
  - Inconsistent study designs make it difficult to evaluate weight-of-evidence. For example, different exposure periods and/or different life stages can result in different responses, which may make weight of evidence more complicated. For example:
  - Effects on gonadal steroidogenesis can be seen with thyroid changes at the end of metamorphosis.
  - With a fast growing thyroid, decreased colloid may be seen instead of follicular hypertrophy.
- Normal variability in thyroid hormone levels across life stages are not well characterized.
- In fish, a T3:T4 ratio is a better indicator of thyroid effects than T4. In mammals, T4 is most often reported as changes are more easily detected than T3 (tightly regulated in many cases)
  - Many studies have only one or two concentrations, so the shape of the dose response curve (e.g., NMDR) cannot be discerned.
  - Background levels of iodide can influence the outcome of studies and may not be reported. 0.5 mg/L I – no change, but with 1 mg/L the impact of perchlorate can be mitigated, but there is some toxicity from potassium iodide itself.

**11.0 Other Factors to Consider in Endocrine Risk Assessments**

There are numerous other points to consider when considering the potential to use risk assessment. Factors include identification of the most sensitive species, sensitive windows of exposure, shape of the concentration-effect curve, reversibility and recovery, identification of a threshold, and transgenerational effects. These factors are briefly discussed in the context of the perchlorate case study.

**11.1 Sensitive Species:**

For perchlorate, fish and frogs appear to be sensitive species as adverse effects on growth and/or development have been seen at similar concentrations.

- In Zebrafish, the perchlorate LOEC results in changes in condition factor (body weight (g) / [total length (cm)]^3^ x 100). This indicates decreased growth, which is considered an adverse effect.
- In Xenopus laevis, the perchlorate LOEC produced changes in the developmental distribution of tadpoles such that the median stage of developmental was 1-2 stages earlier with perchlorate treatment. However, tadpole development is a subjective endpoint and the time to completion of different developmental stages varies; therefore, measurement of ‘time to completion of metamorphosis’ would have greater utility.
- Birds were less sensitive than fish and frogs. This may be related to thyroid hormone storage in birds, which may require more protracted thyroid perturbations for effects to be seen.
- Mammalian effects can vary depending on the concentration of perchlorate, duration of exposure and species sensitivity to thyroid perturbations. In the data examined, rats were less sensitive than fish and frogs to thyroid perturbations by perchlorate.

**11.2 Sensitive Windows of Exposure**

Sufficient thyroid hormone levels are needed for normal development in most species. Thus, there is life stage-specific sensitivity to perchlorate-induced thyroid perturbations because the most sensitive effects are seen in developing animals. In frogs, this finding is consistent with thyroid hormone data where levels are low in adult frogs and are less affected by thyroid perturbations.

**11.3 Shape of the Concentration-Effect Curve [Non-monotonic Dose Response (NMDR)]**

Across the studies evaluated, there were some responses that showed NMDR relationships.

- - In one study in stickleback fish (Petersen et al., 2015), T3 and T4 levels were affected at the low concentration, but not at two higher concentrations across life stages (larval, juvenile and adult stages). There were no apical endpoints evaluated in this study, so the relevance of these hormone changes cannot be determined.
  - In the mammalian two-generation reproductive toxicity study (York et al., 2001), increases in thyroid hormone (T4) levels were seen at lower dose levels (0.3 and/or 3 mkd) with a decrease in T4 levels at the high dose (30 mkd). This effect was seen at some time points (P1 adult males, F1 weanling females, F1 adult males, F2 weanling females), but was inconsistent with respect to dose, gender and generation. This may be related to variability in hormone measurements or may be related to the magnitude of effect, the time of exposure and the compensatory capacity of the hypothalamic-pituitary-thyroid axis to reestablish homeostasis (i.e., establishment of a new thyroid set point, which takes longer to manifest at lower doses). NMDR was not seen in more biologically significant endpoints (e.g., reproduction) that would be associated with population level effects.

These NMDR were not associated with population-level adverse effects; therefore, NMDR was not considered to impact the current assessment.

**11.4 Recovery and Reversibility**

Many of the references evaluated reversibility for perchlorate effects across different taxa. Here are a few examples of perchlorate reversibility.

- In human clinical medicine, where perchlorate was once used to inhibit hyperthyroidism, the effects of perchlorate have been considered to be reversible (e.g., Wolff, 1998).
- A unique feature of thyroid function, namely that the thyroid is an endocrine gland capable of hormone storage, appears to be the key reason why thyroidal T4 content is the most sensitive indicator and plasma thyroid hormone concentrations are much less useful indicators of thyroid alterations. Thus, exposure to perchlorate or other agents that cause decreases in circulating thyroid hormones, results in feedback activation of the hypothalamic-pituitary-thyroid axis (increased thyrotropin release) that leads to the release of stored hormones. This release of stored hormone provides at least temporary compensation and restoration of euthyroid levels of circulating thyroid hormones (Delange and Ermans, 1996; Taurog, 1996). There is evidence in the mammalian literature that sustained exposure to thyroid inhibitors can lead to a cyclic pattern of responses with temporary restoration of euthyroid levels of circulating hormone followed by the return of hypothyroid conditions (with continued exposure to the inhibitor), renewed HPT axis activation leading to more stored hormone release, etc. (York et al. 2001a,b). Thus, effects on thyroid hormone endpoints at earlier time points were not significant at later time points. This change in endpoint sensitivity over time may indicate some adaptation or compensatory response.
- The patterns of plasma thyroid hormones in our study were consistent with the interpretation that such cyclic responses were occurring in our studies of ammonium perchlorate effects in bobwhite quail. Reversibility of ammonium perchlorate effects on quail chicks has been examined (McNabb et al., 2004). Specifically, the rate of thyroidal T4 loss was most rapid in the first two weeks of ammonium perchlorate exposure, then slowed between two and eight weeks of exposure. This suggests some compensatory increase in hormone production, although decreases in hormone degradation and loss from the body also could be playing a role in this response (Chen et al., 2008, 2009). Thyroid hypertrophy in these birds suggests that thyrotropin stimulated increases in thyroid function played a role in increasing hormone supply and reducing the rate of thyroidal T4 loss. In nature, ground dwelling, nonmigratory birds experience sustained exposure to environmental chemicals, so these adaptive responses will be important in the magnitude and time course of ammonium perchlorate effects.
- With removal of perchlorate exposure, tadpoles will complete metamorphosis; therefore, effects of perchlorate are reversible in frogs.
- The reversal of perchlorate effects by potassium iodide supports a monotonic response to perchlorate (ATSDR, 2005; Thalmann and Meier, 2013). Increased iodide levels can ‘out compete’ perchlorate at the NIS, restoring adequate thyroidal iodide and hormone synthesis.
- It is anticipated that some developmental effects would not be reversible (e.g., neurodevelopmental effects in mammals); however, these effects were not reported in the current studies. It is anticipated that these effects could occur in wildlife species at higher concentrations of perchlorate.

**11.5 Identification of a Threshold Dose**

There was no evidence of toxicologically significant NMDR in the reports evaluated. Furthermore, the effects of perchlorate are reversible with potassium iodide, which indicates a reasonable understanding of dose-response and MOA. Thus, there was a general consensus among the workshop participants that a threshold could be identified for population-level adverse effects by perchlorate. This supports the use of risk assessment for this compound.

**11.6 Transgenerational Effect**

There were limited data on transgenerational effects; however, there were some studies where the parental generation was exposed to perchlorate during development and into adulthood. The subsequent offspring, which were not exposed, did not exhibit perchlorate-related (e.g., Bernhardt et al., 2011).

**12.0 What constitutes an adverse ‘population level’ effect?**

**12.1 Amphibians**

**12.1.1.** **Screening Study Data**

Screening studies may rely on a **delay in metamorphosis in pre-metamorphic tadpoles**. This would require follow-up through the entire metamorphosis process. With greater information (LAGDA), it may be possible to extrapolate delays in metamorphosis with effect on overall metamorphosis duration. However, LAGDA continues exposure from pre-metamorphosis through adulthood; many frog species will transition to land after metamorphosis; therefore, perhaps exposure should be discontinued at the end of metamorphosis.

**12.1.2 Test Data**

With ecotox tests, longer time to **completion of metamorphosis** can be detected. For population level protection, this ‘increased time to complete metamorphosis’ is interpreted as an adverse effect. Thus, time to completion of metamorphosis is an appropriate point of departure (POD) for risk assessment based on available knowledge. This interpretation is conservative and therefore, protective; however, it may change with an improved understanding of:

- **Variability in Metamorphosis Timing Across Individuals** - Stochastic variability in time to complete metamorphosis is observed in the laboratory. This variability is likely to be greater in environmental conditions. Therefore, small increases in the time to complete metamorphosis may not be appropriate for determining effects on a population level.
- **Variable Stage Length During Metamorphosis** - Differences in developmental stage in frogs may not carry through to a difference in time to complete metamorphosis, depending on the time during development that the tadpoles are evaluated (i.e., stages are not uniform length, so the stage at evaluation could make a difference)
- **Impact of Water vs. Terrestrial Conditions** - There is a data gap related to conditions in water vs. terrestrial conditions that could determine the impact of delays in metamorphosis
- **Field Biology Studies to Determine Population Impact** - Few if any papers address population effects in field biology studies to determine critical parameters that determine population impact (e.g., what magnitude of delay in metamorphosis has an effect on adult life stage?).

Time to completion of metamorphosis may not be appropriate for determining effects on the population level. Additional data will help clarify impacts of delays of different magnitude on metamorphosis and population well being.

Ecotox tests also can detect an **inability to complete metamorphosis**, which would be adverse at the population level.

**12.2. Mammals**

**12.2.1 Altered Thyroid Parameters in Mammalian Studies**

- In mammals, endpoints designated as adverse effects in the individual (e.g., thyroid histopathology) would not be considered a ‘population level adverse effect’. In the perchlorate data sets examined, there was a developmental delay (increased delayed ossification) in the rat developmental toxicity study at doses that caused maternal/fetal thyroid changes, but no overt toxicity. The delay in ossification occurred in ‘late ossifying bones’ and ossification continues postnatally; therefore, there would be no lasting effect. Thus, the delay in ossification would not affect survival or function in postnatal animals. This effect would not be considered population relevant
- Thus, based on the publications evaluated for this review, there were no effects on survival, reproduction, offspring growth, or neurobehavioral endpoints assessed (e.g., motor activity, auditory startle, passive avoidance, etc.) that might adversely affect survival. Across these studies, there was no evidence of population level effects at doses ≤ 10 mg/kg/day ammonium perchlorate.

**13.0 Conclusions for the Case Study – Hazard and Risk assessment**

There was no evidence of significant NMDRs in the reports evaluated; therefore, a threshold could be established for perchlorate. Reversal by potassium iodide also supports a monotonic response to perchlorate. At the workshop, the perchlorate group concluded that risk assessment is possible.

**REFERENCES**

ATSDR (2005). Agency for Toxic Substances and Disease Registry. Toxicological profile for perchlorates. Available at: <http://www.atsdr.cdc.gov/toxprofiles/tp162-c3.pdf>

Bernhardt, R.R., Von Hippel, F.A., and O’Hara, T.M. (2011). Chronic perchlorate exposure causes morphological abnormalities in developing stickleback. *Environ. Toxicol. Chem.* **30(6),** 1468-1478.

Chen, Y., Sible, J.C., and McNabb, F.M.A. (2008). Effects of maternal exposure to ammonium perchlorate on thyroid function and the expression of thyroid-responsive genes in Japanese quail embryos. *General and Comparative Endocrinology* **159,** 196-207.

Chen, Y., McNabb, F.M.A., and Sible, J.C. (2009). Perchlorate exposure induces hypothyroidism and affects thyroid-responsive genes in liver but not brain of quail chicks. *Arch. Environ. Contam. Toxicol.* **57,** 598-607.

Cooke, P. S., and Meisami, E. (1991). Early hypothyroidism in rats increases adult testis and reproductive organ size but does not change testosterone levels. *Endocrinology* **129,** 237–243.

Dean, K.E., Palachek, R.M., Noel, J.M., Warbritton, R., Aufderheide, J., and Wireman, J. (2004). Development of freshwater water-quality criteria for perchlorate. *Environ. Toxicol.* **23(6),** 1441-1451.

Delange, F. M., and Ermans, A-M. (1996). Iodine deficiency. In: *Werner and Ingbar’s The Thyroid* (L. E. Braverman, R. D. Utiger, Eds.), 7th ed., pp. 296–316. Lippincott-Raven, Philadelphia

Goleman, W.L., Urquidi, L.J., Anderson, T.A., Smith, E.E., Kendall, R.J., and Carr, JA. (2002). Environmentally relevant concentrations of ammonium perchlorate inhibit development and metamorphosis in Xenopus laevis. *Environ. Toxicol. Chem.* **21(2),** 424-430.

Goleman, W.L., and Carr, J.A. (2006). Contribution of ammonium ions to the lethality and antimetamorphic effects of ammonium perchlorate. *Environ. Toxicol. Chem.* **25(4),** 1060-1067.

Japan Ministry of the Environment. (2011). Initial environmental risk assessment of perchlorate. March 2011 (kindly provided by K. Yamasaki).

Kalkhoff, S.J., Stetson, S.J., Lund, K.D., Wanty, R.B., and Linder, G.L. (2010). Perchlorate data for streams and groundwater in selected areas of the United States, 2004: U.S. Department of the Interior. U.S. Geological Survey Data Series 495, 43 p. with appendix.

Klimisch, H.-J., Andreae, M., and Tillmann, U. (1997). A systematic approach for evaluating the quality of experimental toxicological and ecotoxicological data. *Regul. Toxicol. Pharmacol.* **25,** 1–5.

Kucharzyk, K.H., Crawford, R.L., Cosens, B., and Hess, T.F. (2009). Development of drinking water standards for perchlorate in the United States. *Journal of Environmental Management* **91,** 303-310.

McNabb F.M.A., Larsen, C.T., and Pooler, P.S. (2004). Ammonium perchlorate effects on thyroid function and growth in Bobwhite quail chicks. *Environ. Toxicol. Chem.* **23(4),** 997-1003.

OECD (2012). OECD Conceptual Framework for Testing and Assessment of Endocrine Disrupters (as revised in 2012 from the version in Guidance Document 150 (Annex 1.4)). <http://www.oecd.org/env/ehs/testing/OECD%20Conceptual%20Framework%20for%20Testing%20and%20Assessment%20of%20Endocrine%20Disrupters%20for%20the%20public%20website.pdf>)

Petersen, A.M., Dillon, D., Bernhardt, R.A., Torunsky, R., Postlethwait, J.H., von Hippel, F.A., Buck, C.L., and Cresko, W.A. (2015). Perchlorate disrupts embryonic androgen synthesis and reproductive development in threespine stickleback without changing whole-body levels of thyroid hormone. *Gen. Comp. Endocrinol.* **210,** 130-144.

Saito, K, Yamamoto, K., Takai, T., and Yoshida, S. (1983). Inhibition of iodine accumulation by perchlorate and thiocyanate in a model of the thyroid iodide transport system. *Acta Endocrinol. (Copenh).* **104,** 456-461.

Schneider, K. Schwarz, M., Burkholder, I., Kopp-Schneider, A., Edler, L., Kinsner-Ovaskainen, A., Hartung, T., and Hoffmann, S. (2009) “ToxRTool”, a new tool to assess the reliability of toxicological data. *Toxicol. Lett.* **189,** 138-144. <https://eurl-ecvam.jrc.ec.europa.eu/about-ecvam/archive-publications/toxrtool>

Siglin, J.C., Mattie, D.R., Dodd, D.E., Hildebrandt, P.K., and Baker, W.H. (2000). A 90-day drinking water toxicity study in rats of the environmental contaminant ammonium perchlorate. *Toxicol. Sci.* **57,** 61-74.

Taurog, A. (1996). Hormone synthesis: Thyroid iodine metabolism. In: *Werner and Ingbar’s The Thyroid* (L. E. Braverman, R. D. Utiger, Eds.), 7th ed., pp. 47–81. Lippincott-Raven, Philadelphia.

Tietge, J.E., Butterworth, B.C., Haselman, J.T., Holcombe, G.W., Hornung, M.W., Korte, J.J., Kosian, P.A., Wolfe, M., and Degitz, S.J. (2010). Early temporal effects of three thyroid hormone synthesis inhibitors in Xenopus laevis. *Aquatic Toxicol.* **98(1),** 44-50.

Tietge, J.E., Holcombe, G.W., Flynn, K.M., Kosian, P.A., Korte, J.J., Anderson, L.E., Wolf, D.C., and Degitz, S.J. (2005). Metamorphic inhibition of Xenopus laevis by sodium perchlorate: Effects on development and thyroid histology. *Environ. Toxicol. Chem.* **24(4),** 926-933.

Urbansky, E.T. (1998). Perchlorate Chemistry: Implications for Analysis and Remediation. CRC Press LLC. Available at: <https://clu-in.org/download/contaminantfocus/perchlorate/urbansky2.pdf>

Thalmann, S., and Meier, C.A. (2013). Chapter 11B: Effects of drugs on TSH secretion, thyroid hormones absorption, synthesis, metabolism and action. In: *Werner & Ingbar's The Thyroid: A Fundamental and Clinical Text.* Tenth Edition (L.E. Braverman, D.S Cooper, Eds.) Wolters Kluwer/Lippincott Williams & Wilkins, New York.

Wolff, J. (1998). Perchlorate and the thyroid gland. *Pharmacol. Rev.* **60,** 89-105.

York, R.G., Brown, W.R., Girard, M.F., and Dollarhide, J.S. (2001a). Two-generation reproduction study of ammonium perchlorate in drinking water in rats evaluates thyroid toxicity. *Intl. J. Toxicol.* **20,** 183-197.

York, R.G., Brown, W.R., Girard, M.F., and Dollarhide, J.S. (2001b). Oral (drinking water) developmental toxicity study of ammonium perchlorate in New Zealand white rabbits. *Intl. J. Toxicol.* **20,** 199-205.

York, R.G., Funk, K.A., Girard, M.F., Mattie, D., and Strawson, J.E. (2003). Oral (drinking water) developmental toxicity study of ammonium perchlorate in Sprague-Dawley rats. *Int. J. Toxicol.* **22,** 453-464.

**Appendix 1**

**A WOE for Perchlorate Effects on the Estrogen, Androgen, and Thyroid Signaling Pathways in Mammals**

Table S-3. OECD Revised Conceptual Framework for Testing and Assessment of Endocrine Disrupters (OECD, 2012)

| **Mammalian and non mammalian Toxicology** | | |
| --- | --- | --- |
| **Level 1**  Existing data and non-test information | - Physical & chemical properties, e.g., MW reactivity, volatility, biodegradability. - All available (eco)toxicological data from standardized or non-standardized tests. - Read across, chemical categories, QSARs and other *in silico* predictions, and ADME model predictions. | |
| **Level 2**  *In vitro* assays providing data about selected endocrine mechanism(s) / pathways(s)  (Mammalian and non mammalian methods) | - Estrogen or androgen receptor binding affinity - Estrogen receptor transactivation (OECD TG 455) - Androgen or thyroid transactivation (If/when Test Guidelines (TG) are available) - Steroidogenesis *in vitro* (OECD TG 456) - MCF-7 cell proliferation assays (ER ant/agonist) - Other assays as appropriate | |
|  | **Mammalian Toxicology** | **Non-Mammalian Toxicology** |
| **Level 3**  *In vivo* assays providing data about selected endocrine mechanism(s) / pathway(s)^1^ | - Uterotrophic assay (OECD TG 440) - Hershberger assay (OECD TG 441) | - *Xenopus* embryo thyroid signalling assay (when/if TG is available) - Amphibian metamorphosis assay (OECD TG 231) Fish reproductive screening assay (OECD TG 229) - Fish screening assay (OECD TG 230) - Androgenized female stickleback screen (Guidance Document (GD) 140) |
| **Level 4**  *In vivo* assays providing data on adverse effects on endocrine relevant endpoints^2^ | - Repeated dose 28-day study (OECD TG 407) - Repeated dose 90-day study (OECD TG 408) - 1-generation reproduction toxicity study (OECD TG 415) - Male pubertal assay (see Guidance Document (GD) 150 Chapter C4.3)^3^ - Female pubertal assay (see Guidance Document (GD) 150 Chapter C4.4)^3^ - Intact adult male endocrine screening assay (see Guidance Document (GD) 150 Chapter Annex 2.5) - Prenatal developmental toxicity study (OECD TG 414) - Chronic toxicity and carcinogenicity studies (OECD TG 451-3) - Reproductive screening test (OECD TG 421 if enhanced) - Combined 28-day/reproductive screening assay (OECD TG 422 if enhanced) - Developmental neurotoxicity (OECD TG 426) | - Fish sexual development test (Draft OECD TG 234) - Fish reproduction Partial Lifecycle Test (when/if TG is available) - Larval amphibian growth & development assay (OECD 241) - Avian reproduction assay (OECD TG 206) - Mollusc partial lifecycle assays (when TG is available)^4^ - Chironomid toxicity test (TG 218-219)^4^ - *Daphnia* reproduction test (with male induction) (OECD TG 211)^4^ - Earthworm reproduction test (OECD TG 222, 2004)^4^ - Enchytraeid reproduction test (OECD TG 220, 2004)^4^ - Sediment water *Lumbriculus* toxicity test using spiked sediment (OECD TG 225, 2007)^4^ - Predatory mite reproduction test in soil (OECD TG 226, 2008)^4^ - Collembolan reproduction test in soil (TG OECD 232, 2009)^4^ |
| **Level 5**  *In vivo* assays providing more comprehensive data on adverse effects on endocrine relevant endpoints over more extensive parts of the life cycle of the organism^2^ | - Extended one-generation reproductive toxicity study (OECD TG 443)^5^ - 2-Generation reproduction toxicity study (OECD TG 416 most recent update) | - Fish lifecycle toxicity test (FLCTT) (when TG is available) - *Medaka* extended one-generation test (MEOGRTS) (OECD 240) - Avian 2 generation reproductive toxicity assay (when TG is available) - Mysid lifecycle toxicity test (when TG is available)^4^ - Copepod reproduction and development test (when TG is available)^4^ - Sediment water chironomid life cycle toxicity test (OECD TG 233)^4^ - Mollusc full lifecycle assays (when TG is available)^4^ - *Daphnia* multigeneration assay (if/when TG is available)^4^ |

^1^ Some assays may also provide some evidence of adverse effects.

^2^ Effects can be sensitive to more than one mechanism and may be due to non-ED mechanisms.

^3^ Depending on the guideline/protocol used, the fact that a substance may interact with a hormone system in these assays does not necessarily mean that when the substance is used it will cause adverse effects in humans or ecological systems.

^4^ At present, the available invertebrate assays solely involve apical endpoints which are able to respond to some endocrine disrupters and some non-EDs. Those in Level 4 are partial lifecycle tests, while those in Level 5 are full- or multiple lifecycle tests.

^5^ The new EOGRT study (OECD TG 443) is preferable for detecting endocrine disruption because it provides an evaluation of a number of endocrine endpoints in the juvenile and adult F1, which are not included in the 2-generation study (OECD TG 416) adopted in 2001.

**TABLE S-4. Summary of data available in relation to the OECD’s Conceptual Framework**

| **Mammalian and non-mammalian Toxicology** | | |
| --- | --- | --- |
| **Level 1**  Existing data and non-test information | - Physical & chemical properties, e.g., MW reactivity, volatility, biodegradability - Read across, chemical categories, QSARs and other *in silico* predictions, and ADME model predictions | |
| **Level 2**  *In vitro* assays providing data about selected endocrine mechanism(s) / pathways(s)  (Mammalian and non mammalian methods) | - In vitro assays – ToxCast results | |
|  | **Mammalian Toxicology** | **Non-Mammalian Toxicology** |
| **Level 3**  *In vivo* assays providing data about selected endocrine mechanism(s) / pathway(s) | - 90-day thyroid toxicity study (Wu et al., 2010) - Perchlorate discharge test (Coelho-Palermo Cunha, 2007) |  |
| **Level 4**  *In vivo* assays providing data on adverse effects on endocrine relevant endpoints | - 90-day toxicity study (Siglin et al., 2000) - 2 studies: Repeated dose 90-day neurotoxicity study (OECD 424) - 4 studies: Chronic toxicity and carcinogenicity study (OECD 451-3) - 4 Prenatal developmental toxicity studies (OECD 414) (Thuett et al., 2002a, b; York et al., 2001, 2003) - 2 Prenatal developmental neurotoxicity studies   (York et al., 2004, 2005) |  |
| **Level 5**  *In vivo* assays providing more comprehensive data on adverse effects on endocrine relevant endpoints over more extensive parts of the life cycle of the organism | - 2-Generation reproduction toxicity study (York et al., 2001) | - None available |

**TABLE S-5.** **Estrogenic/Anti-Estrogenic Pathway for Perchlorate in Mammalian Species**

| **Lines of Evidence Indicating Potential Interaction with the Estrogenic/Anti-Estrogenic Pathway for Perchlorate^1^** | | | | | | | | | | |
| --- | --- | --- | --- | --- | --- | --- | --- | --- | --- | --- |
| CF Level | *2 -In Vitro* | | | 4 - *In Vivo* Mammalian | | | | | | 5 - *In Vivo* Repro |
| **Study Type /**  **Literature Citation** | **ToxCast** | **ToxCast** | | **90-day Study in Rats**  **(Siglin et al., 2000)** | **Devtl Toxicity Study – Deer Mice**  **(Thuett et al., 2002a)** | **Devtl Toxicity Study – Rat**  **(York et al., 2003)** | **Devtl Toxicity Study – Rabbit**  **(York et al., 2001)** | **Devtl Neurotoxicity Study – Rat**  **(York et al., 2004)** | **Refined Devtl Neurotox. Study – Rat**  **(York et al., 2005)** | **2-Generation Repro Tox – Rat**  **(York et al., 2001)** |
| **Perchlorate (A=ammonium; K = potassium; S = sodium)** | **A** | **K** | | **A** | **A** | **A** | **A** | **A** | **A** | **A** |
| **Other Scientifically Relevant Information (OSRI)** | | | | | | | | | | |
| ER Transactivation | N^2^ | N^2^ |  | |  |  |  |  |  |  |
| Steroidogenesis (Aromatase Inhibition) | N | N |  | |  |  |  |  |  |  |
| Adrenal Weight (steroidogenesis/stress) |  |  |  | | N |  |  |  |  | N |
| Testis Weight |  |  |  | | N |  |  |  |  | N |
| Pituitary Weight |  |  |  | |  |  |  |  |  | N |
| Ovary weight |  |  |  | | N |  |  |  |  | N |
| Adrenal Histopathology |  |  | N | |  |  |  |  |  | N |
| Testis Histopathology |  |  | N | |  |  |  |  |  | N |
| Ovary Histopathology |  |  | N | |  |  |  |  |  | N |
| Oviduct Histopathology |  |  |  | |  |  |  |  |  | N |
| Uterus Histopathology |  |  | N | |  |  |  |  |  | N |
| Vagina Histopathology |  |  | N | |  |  |  |  |  | N |
| Cervix Histopathology |  |  |  | |  |  |  |  |  | N |
| Mammary Histopathology |  |  | N | |  |  |  |  |  | N |
| Pituitary Histopathology |  |  | N | |  |  |  |  |  | N |
| Epididymis Histopath |  |  | N | |  |  |  |  |  | N |
| Seminal Vesicle Histopath |  |  | N | |  |  |  |  |  | N |
| Prostate Histopathology |  |  | N | |  |  |  |  |  | N |
| Coagulating Gland Histo |  |  |  | |  |  |  |  |  | N |
| Estrous Cyclicity |  |  | N | |  |  |  |  |  | N |
| Implantation Number |  |  |  | |  | N | N | N | N | N |
| Mean Litter Size |  |  |  | | N | N | N | N | N | N |
| Number Viable Fetuses/Pups (term) |  |  |  | |  | N | N | N | N | N |
| Number Dead Fetuses/Pups (term) |  |  |  | |  | N | N | N | N | N |
| Late Resorptions |  |  |  | |  | N | N | N |  |  |
| Post-implantation Loss |  |  |  | |  | N | N | N |  |  |
| Pre-implantation Loss |  |  |  | |  | N | N | N |  |  |
| Early Resorptions^5^ |  |  |  | |  | N | N | N |  |  |
| Corpora Lutea Number |  |  |  | |  | N | N | N |  |  |
| Fetal/pup body weight |  |  |  | | N | N | N | N | N | N |
| Sex Ratio |  |  |  | | N | N | N | N | N | N |
| Gravid Uterine Weights |  |  |  | |  | N | N |  |  |  |
| Fetal External Alteration |  |  |  | |  | N | N | N |  |  |
| Fetal internal alteration (e.g., ectopic testes) |  |  |  | |  | N | N |  |  |  |
| Time to mating |  |  |  | |  |  |  |  |  | N |
| Gestation length |  |  |  | |  |  |  | N | N | N |
| Maternal behavior |  |  |  | |  |  |  | N |  | N |
| Gestation body weight |  |  |  | |  | N | N | N | N | N |
| No. rats that mated |  |  |  | |  |  |  | N | N | N |
| Dams w/ no live pups |  |  |  | |  |  |  |  |  | N |
| Dams with stillborn pups |  |  |  | |  |  |  | N | N | N |
| Postnatal pup survival |  |  |  | | N |  |  | N | N | N |
| Fertility indices |  |  |  | |  |  |  |  |  | N |
| Mating indices |  |  |  | |  |  |  |  |  | N |
| Viability index |  |  |  | |  |  |  | N | N | N |
| Gestation index |  |  |  | |  |  |  |  | N |  |
| Lactation index |  |  |  | |  |  |  | N |  | N |
| Age at vaginal opening |  |  |  | |  |  |  | N | N | N |

^1^ Key to responses: Positive (P), negative (N) or equivocal (E) observation; arrows (↓ or ↑) indicate the direction of the response; A shaded cell indicates that parameter was not evaluated or is not applicable.

^2^ Perchlorate was positive in one (Tox21_ERα_LUC_BG1_Agonist) of two ERα agonist assays at concentrations greater than the cytotoxicity limit (3 µM); perchlorate was negative in both ERα antagonist assays (iCSS ToxCast Dashboard, Jan. 2016).

**TABLE S-6. Androgenic/Anti-Androgenic Pathway for Perchlorate in Mammalian Species**

| **Lines of Evidence Indicating Potential Interaction with the Androgenic/Anti-Androgenic Pathway for Perchlorate^1^** | | | | | | | | | | | |
| --- | --- | --- | --- | --- | --- | --- | --- | --- | --- | --- | --- |
| CF Level | *2 -In Vitro* | | | 4 - *In Vivo* Mammalian | | | | | | | 5 - *In Vivo* Repro |
| **Study Type /**  **Literature Citation** | **ToxCast** | **ToxCast** | | **90-day Study in Rats**  **(Siglin et al., 2000)** | | **Devtl Toxicity Study – Deer Mice**  **(Thuett et al., 2002a)** | **Devtl Toxicity Study – Rat**  **(York et al., 2003)** | **Devtl Toxicity Study – Rabbit**  **(York et al., 2001)** | **Devtl Neurotoxicity Study – Rat**  **(York et al., 2004)** | **Refined Devtl Neurotox. Study – Rat**  **(York et al., 2005)** | **2-Generation Repro Tox – Rat**  **(York et al., 2001)** |
| **Perchlorate (A=ammonium; K = potassium; S = sodium)** | **A** | **K** | | **A** | | **A** | **A** | **A** | **A** | **A** | **A** |
| **Other Scientifically Relevant Information (OSRI)** | | | | | | | | | | | |
| AR Transactivation | N | | N | |  |  |  |  |  |  |  |
| Steroidogenesis (Aromatase Inhibition) | N | | N | |  |  |  |  |  |  |  |
| Adrenal Weight (steroidogenesis/stress) |  | |  | |  | N |  |  |  |  | N |
| Testis Weight |  | |  | |  | N |  |  |  |  | N |
| Pituitary Weight |  | |  | |  |  |  |  |  |  | N |
| Ovary weight |  | |  | |  | N |  |  |  |  | N |
| Adrenal Histopathology |  | |  | | N |  |  |  |  |  | N |
| Testis Histopathology |  | |  | | N |  |  |  |  |  | N |
| Ovary Histopathology |  | |  | | N |  |  |  |  |  | N |
| Uterus Histopathology |  | |  | | N |  |  |  |  |  | N |
| Mammary Histopathology |  | |  | | N |  |  |  |  |  | N |
| Pituitary Histopathology |  | |  | | N |  |  |  |  |  | N |
| Epididymis Histopath |  | |  | | N |  |  |  |  |  | N |
| Seminal Vesicle Histopath |  | |  | | N |  |  |  |  |  | N |
| Prostate Histopathology |  | |  | | N |  |  |  |  |  | N |
| Coagulating Gland Histopathology |  | |  | |  |  |  |  |  |  | N |
| Sperm Motility |  | |  | | N |  |  |  |  |  | N |
| Sperm Count |  | |  | | N |  |  |  |  |  | N |
| Sperm Density (Concentration) |  | |  | | N |  |  |  |  |  | N |
| Sperm Morphology |  | |  | | N |  |  |  |  |  | N |
| Testosterone Levels (total) |  | |  | |  | N |  |  |  |  |  |
| Implantation Number |  | |  | |  |  | N | N | N | N | N |
| Mean Litter Size |  | |  | |  | N | N | N | N | N | N |
| Number Viable Fetuses/Pups (term) |  | |  | |  |  | N | N | N | N | N |
| Number Dead Fetuses/Pups (term) |  | |  | |  |  | N | N | N | N | N |
| Fetal/pup body weight |  | |  | |  | N | N | N | N | N | N |
| Sex Ratio |  | |  | |  | N | N | N | N | N | N |
| Gravid Uterine Weights |  | |  | |  |  | N | N |  |  |  |
| Fetal External Alteration |  | |  | |  |  | N | N | N |  |  |
| Fetal internal alteration (e.g., ectopic testes) |  | |  | |  |  | N | N |  |  |  |
| Time to mating |  | |  | |  |  |  |  |  |  | N |
| No. rats that mated |  | |  | |  |  |  |  | N | N | N |
| Fertility indices |  | |  | |  |  |  |  |  |  | N |
| Mating indices |  | |  | |  |  |  |  |  |  | N |
| Age at preputial separation |  | |  | |  |  |  |  | N | N | N |

^1^ Key to responses: Positive (P), negative (N) or equivocal (E) observation; arrows (↓ or ↑) indicate the direction of the response; A shaded cell indicates that parameter was not evaluated or is not applicable.

**TABLE S-7. Thryoid/Anti-Thyroid Pathway for Perchlorate in Mammalian Species**

| CF Level | *2 -In Vitro* | | *3 – In Vivo Mammalian Limited MoA* | | | 4 - *In Vivo* Mammalian | | | | | | | 5 - *In Vivo* Repro | |
| --- | --- | --- | --- | --- | --- | --- | --- | --- | --- | --- | --- | --- | --- | --- |
| **Study Type /**  **Literature Citation** | **ToxCast** | **ToxCast** | **90-day Thyroid Study - Rat (gavage)**  **(Wu et al., )** | | **Perchlorate Discharge Test – Rat**  **(Coelho-Palermo Cunha, 2007)** | **90-day Study - Rat**  **(Siglin et al., 2000)** | **Devtl Toxicity Study – Deer Mice**  **(Thuett et al., 2002a)** | **Devtl Toxicity Study – Deer Mice**  **(Thuett et al., 2002b)** | **Devtl Toxicity Study – Rat**  **(York et al., 2003)** | **Devtl Toxicity Study – Rabbit**  **(York et al., 2001)** | **Devtl Neurotoxicity Study – Rat**  **(York et al., 2004)** | **Refined Devtl Neurotox. Study – Rat**  **(York et al., 2005)** | **2-Generation Repro Tox – Rat**  **(York et al., 2001)** | |
| **Perchlorate (A=ammonium; K = potassium; S = sodium)** | **A** | **K** | **A** | | **A** | **A** | **A** | **A** | **A** | **A** | **A** | **A** | **A** | |
| TR Transactivation | N | N |  |  | |  |  |  |  |  |  |  |  |  |
| T3 Levels |  |  | E |  | | P ↓ |  | NA/N | P ↓/P ↓ | N/NA | NA/P ↓ | P ↓/P ↓ | P ↑(M)^2^; N (F)/  N (M, F) |  |
| T4 Levels |  |  | P ↓ |  | | P ↓ |  | NA/P ↑^2^ | P ↓/P ↓ | P ↓/NA | NA/P ↓ | P ↓/P ↓ | P ↓(M); P ↑(F)^2^/  P ↑↓(M)^2^; N (F) |  |
| TSH Levels |  |  | P ↑ |  | | P ↑ |  |  | P ↑/P ↑ | N/NA | NA/P ↑ | P ↑/P ↑ | P ↑(M); N (F)/  P ↑(M,F) |  |
| Thyroid Unbound I Release |  |  |  | P ↑ | |  |  |  |  |  |  |  |  |  |
| Thyroid Weight |  |  | P ↑ |  | | P ↑ |  |  | P ↑/NA | N/NA | N/NA | P ↑/P ↑ | P ↑/P ↑ |  |
| Liver Weight Increase (enhanced T4 clearance) |  |  |  |  | |  | NA/N |  |  |  |  |  | N |  |
| Thyroid Histopathology (follicular cells and/or colloid) |  |  |  |  | | P^3,4^ |  | NA/P^4,5^ | P^3,4,6^/P^4^ | P^3^/NA | P^2,4^/P^3,5,6^ | P^3,4,6^/P^4^ | P^3,4,5,6^/P^3,6^ |  |
| Thyroid Morphology |  |  |  |  | |  |  | NA/P^2,7^ |  |  | NA/P^8^ |  |  |  |
| Brain Morphometry |  |  |  |  | |  |  |  |  |  | NA/P^9^ |  |  |  |
| Sodium-iodide symporter mRNA |  |  | P ↑ |  | |  |  |  |  |  |  |  |  |  |
| Thyroglobulin mRNA |  |  | P ↓ |  | |  |  |  |  |  |  |  |  |  |
| Thyroperoxidase mRNA |  |  | P ↓ |  | |  |  |  |  |  |  |  |  |  |
| Pituitary Histopathology |  |  |  |  | | N |  |  |  |  |  |  | N |  |
| Fetal/Pup Developmental Delay |  |  |  |  | |  |  |  | P^10^ | N | N |  | N |  |
| Fetal body weight |  |  |  |  | |  |  |  | N | N |  | N |  |  |
| Pup body weight |  |  |  |  | |  | N |  |  |  | N | N | N |  |
| F1 Neurobehavioral Effects |  |  |  |  | |  |  |  |  |  | N |  |  |  |
| Overt toxicity observed^11^ |  |  |  | **X^12^** | |  |  |  |  |  |  |  |  |  |

^1^ Key to responses: Positive (P), negative (N) or equivocal (E) observation; Not applicable (NA); arrows (↓ or ↑) indicate the direction of the response; A shaded cell indicates that parameter was not evaluated or is not applicable. Studies with ‘/’ are parental/fetal results or parental P1/offspring F1 results.

^2^ Effect did not follow a monotonic dose-response pattern ^3^ Follicular cell hypertrophy

^4^ Decreased colloid ^5^ Decreased size and/or number of active follicles

^6^ Follicular cell hyperplasia ^7^ Decreased follicle number per unit area

^8^ Decreased thyroid follicular lumen diameter and area and increased follicular epithelium height ^9^ Increased corpus callosum thickness on LD 12.

^10^ Delayed ossification ^11^ An “X” in this row indicates that the effect(s) observed in the

^12^ High doses tested (520 mkd); decreased body weight by 13% assay occurred in the presence of overt toxicity.
