# Supplementary material for: Recommended Approaches to the Scientific Evaluation of Ecotoxicological Hazards and Risks of Endocrine-Active Substances
Source: Integr Environ Assess Manag. Author manuscript; Available in PMC 2018 Aug 1. (PMC6069525; doi:10.1002/ieam.1885)
Supplement: Supplement4 — S4 — Case Study Summary Propiconazole S4 — Case Study Summary TBT S4 — TBT Table S4–1 S4 — TBT Table S4–2 S4 — TBT Table S4–3 [file NIHMS1500348-supplement-Supplement4.docx]

**Supplemental Data S3**

**Draft Case Study for the Ecotoxicological Hazard and Risk Evaluation of Propiconazole**

Ellen M. Mihaich†*, Christoph Schäfers‡, David Dreier§, Lisa Ortego#, Yukio Kawashima††, Zhi-Chao Dang‡‡, Keith Solomon§§

†Environmental and Regulatory Resources, Durham, NC USA, emihaich@nc.rr.com

‡Fraunhofer IME Applied Ecology, Schmallenberg, Germany

§Center for Environmental and Human Toxicology, Department of Physiological Sciences, College of Veterinary Medicine, University of Florida, Gainesville, FL USA

#Bayer CropScience, Research Triangle Park, NC USA

††Japan NUS Co., Ltd., Tokyo, Japan

‡‡RIVM, Bilthoven, the Netherlands

§§Centre for Toxicology, School of Environmental Sciences, University of Guelph, Guelph, Ontario, Canada

**Introduction**

Propiconazole (CAS 60207-90-1) was chosen as one of the case study compounds for evaluation in the SETAC Pellston Workshop™ titled Environmental Hazard and Risk Assessment Approaches for Endocrine-Active Substances (EHRA). The aim of the workshop was to use data-rich chemicals that had indications of being endocrine active, but which were not necessarily endocrine disruptors according to current definition, to evaluate hazard and risk-based approaches for assessing endocrine activity. As stated in the main text of this paper, this case study evaluation is not a comprehensive safety evaluation for propiconazole.

Propiconazole is a broad spectrum triazole fungicide used on numerous food and feed crops and also on turf and ornamentals. Propiconazole is also used as a material and wood preservative that serves to protect products such as adhesives, paints, coatings, leather, paper, and textiles against fungal infestation. These use patterns can result in exposure to non-target organisms via ingestion (food and drinking water) and contact. Commonly used modeling methods predict the occurrence of propiconazole in surface waters..

The mode of action of propiconazole is demethylation of C-14 during ergosterol biosynthesis, leading to accumulation of C-14 methyl sterols. In addition, propiconazole is a cytochrome P450 enzyme inhibitor (USEPA 2006). Propiconazole has several metabolites of toxicological interest according to US EPA (2006). These are 1,2,4-triazole and the conjugates triazole alanine and triazole acetic acid. EPA reports potentially endocrine-related effects in mammals from the 1,2,4-triazole but not from the conjugates.

Physical-chemical data for propiconazole were taken from ENVIROfacts Syngenta Crop Protection (<http://www.syngentacropprotection.com/env_stewardship/futuretopics/prop8-16-05.pdf>). Propiconazole water solubility is reported as 0.10 g/L at 20^o^C. The vapor pressure of 4.2 x 10-7 mm Hg at 25^o^C indicates low volatility. The log octanol-water partition coefficient (log Kow) is 3.72 at 25^o^C and the organic carbon to water (Koc) partition coefficient is 382-1817, both indications that it binds and is not very mobile.

From an exposure perspective, propiconazole is not a standard analyte in US monitoring programs, though one paper on water quality analyses in US streams from 2005–2006 reported that propiconazole was detected in four of 29 sites evaluated in 18 of 103 samples, with a maximum concentration of 1.15 μg/L, and a mean detection of 0.291 μg/L (Battaglan et al. 2011). In Japan, the Ministry of the Environment has collected the results of monitoring surveys of agricultural chemicals, including propiconazole, in golf course drains, which is conducted by local governments and regional environment offices from FY2003 to FY2013. The maximum concentration was 53.0 μg/L detected in FY2003 (The Ministry of the Environment, Japan, 2004) . Regulatory authorities (US EPA, European Commission) use typical aquatic modeling for propiconazole and consider the results relevant for environmental protection. Some of these modeling results are available, but are use pattern specific and dated for some documents and may no longer be representative. The exposure information was deemed not critical since the case-study group discussions led away from a purely hazard and risk-based assessment and more to difficulties in interpretation of data and establishment of adverse outcome pathways.

**Methods**

The first literature search list delivered to the propiconazole group consisted of 3246 paper summaries, grouped broadly into those addressing assay types (1372), general (362), glands/organs (1190), vertebrate hormone class (404), invertebrate hormones (185), miscellaneous (1155), and vertebrate hormones (418). When reviewing the subgroups it appeared that most of the literature was on mammalian issues, and for many papers the context for propiconazole was unclear. Thus, the titles and summaries were re-checked for the following keywords: environmental risk assessment, ecotoxicology, invertebrate endocrine system, fish, fungi, snail, plants, bird, insect, wildlife, mollusk, crustacean, small mammal, mode of action, aromatase inhibition, androgenic, estrogenic. The abstracts with hits were checked for thematic relevance for environmental hazard or risk assessment. Thus, all papers addressing effectiveness of use, or toxicology issues excluding mechanistic effects were excluded. Appendix S3-1 contains a list of citations reviewed for relevance and then data quality from the initial down-select of the 3246 papers identified in the literature search. After this review we chose 56 papers including 2 on environmental risk assessment, 19 on different taxa other than used in standard toxicology assays, 16 on mechanistic work concerning aromatase inhibition (8 on toxicology, 8 on ecotoxicology) and 19 of other potential interest, along with a number of unpublished study reports, summarized in regulatory documents and dossier submissions, for the case study (Appendix S3-2).

The reliability of the studies obtained from the literature was assessed based on the criteria described by Klimisch *et al*. (1997). Each study was assigned to one of four categories on the basis of compliance with the criteria, as follows:

1. **Reliable without restrictions** – studies conducted according to testing guidelines (preferably Good Laboratory Practice [GLP]) or in which all of the criteria are fully documented and reported.
2. **Reliable with restrictions** – studies that do not follow broadly accepted testing guidelines (or are not GLP), but that document and report compliance with a substantial majority of the criteria.
3. **Not reliable** – studies in which there are notable deficiencies in scientific integrity (e.g. interferences between the measuring system and the test substance) or that document and report compliance with relatively few of the criteria.
4. **Not assignable** – usually reserved for abstracts, secondary literature, subject reviews or book reviews.

For studies that were unpublished but reviewed by a regulatory authority and accepted for regulatory purposes, the assumption was that they were of appropriate quality. These studies were not given a Klimisch rating. In many cases we were not able to review the actual report so we used either the regulatory summary or the registrant summary in our assessment. When possible we reviewed the data evaluation record prepared by the US EPA or the European Chemicals Agency dossier submissions and summaries.

**High Throughput Assays**

*In vitro* studies have provided useful mechanistic information to identify compounds that have endocrine activity. In addition to these studies, high-throughput screening approaches are also useful to identify activity. For example, the ToxCast program has screened over 1,800 compounds with 800 high-throughput assays, including those measuring ER, AR, T, and aromatase function (Filer et al., 2014). Here, ToxCast data were compiled from the EDSP21 dashboard (accessed 1 Feb 2016) and summarized in Table S3-1, where propiconazole was active in 5/10 androgen receptor (AR) assays, 4/17 estrogen receptor (ER) assays, 1/4 thyroid receptor (TR) assays, and 1/1 aromatase inhibition assay. Moreover, the USEPA has used data from these assays to develop ER and AR agonist/antagonist area under the curve (AUC) models (Browne et al. 2015). These models aim to reduce false positives/negatives by employing data from multiple assays to determine pathway signaling using a systems biology approach (Friedman et al., 2016). In the ER model, propiconazole had an AUC of 0.0000437, which is less than the positive threshold of 0.1. However, the AR antagonist model has a positive threshold of 0.05, and propiconazole had an AUC of 0.111 indicating weak AR antagonism. From this, data from high-throughput screening indicate that propiconazole has the potential to inhibit aromatase activity and act as a weak AR antagonist. However, both of these activities are near the cytotoxicity limit so caution should be used in interpretating the results.

**Table S3-1**

| **Taxonomic Group/ Species name** | **Study type** | **Klimisch Score** | **Concentrations Tested** | **Results** | **Reference** |
| --- | --- | --- | --- | --- | --- |
| MDA-kb2 cells (Homo sapiens) | AR antagonist transactivation assay | 2 | 0.01–10 μM | AR Antagonism: IC50 = 7.71 μM | Aït-Aïssa et al. 2010 |
| COS-1 cells (Non-human primate) | CAR3 (human and mouse) reporter assay | 2 | 1, 3, 10, and 30 μM | Mouse: LOEC = 3 μM  Human: LOEC = 30 μM | Currie et al. 2014 |
| H295R cells (Homo sapiens) | Steroid (progesterone, estradiol, and testosterone) synthesis | 2 | 1, 3, 10, 30, 100 μM | Decreased estradiol: LOEC = 30 μM | Goetz et al. 2009 |
| MCF-7 cells (Homo sapiens)  CHO cells (Hamster)  H295R cells (Homo sapiens) | Cell proliferation assay (E2 and T)  AR antagonist transactivation assay  Steroid (E2 and T) synthesis assay | 1 | 0.001-150 μM (20 concentrations)  0.025-30 μM (12 concentrations)  0.01-3 μM (6 concentrations) | MCF-7 (E2): EC50 = 55 μM  MCF-7 (T): IC50 = 32 μM  AR Antagonism: IC50 = 18 μM  H295R (E2): 0.9 μM  H295R (T): 5.3 μM | Kjaerstad et al. 2010 |
| MVLN cells (Homo sapiens)  CHO-K1 cells (Hamster)  JEG-3 cells (Homo sapiens) | ER agonist transactivation assay  AR antagonist transactivation assay  Aromatase inhibition assay | 1 | ER agonist: 1 × 10^−10^ to 1 × 10^−4^ M  AR antagonist: 1 × 10^−10^ to 1 × 10^−5^ M  Aromatase inhibition: 1 × 10^−9^ to 1 × 10^−4^ M | ER agonism: EC50 = 1.2 × 10^−5^ M  AR antagonism: IC50 = 2.8 × 10^−6^ M  Aromatase inhibition: LOEC = 1.0 × 10^−7^ M | Kjeldsen et al. 2013 |
| CHO cells (Hamster) | ER agonist transactivation assay  AR antagonist transactivation assay | 1 | Concentrations ≤10^-5^ M | ER agonism: No activity  AR antagonism: RIC20 = 6.2 × 10^−6^ M | Kojima et al. 2003 |
| JEG-3 (Homo sapiens) | Aromatase inhibition assay | 1 | 1, 3, 10 μM | Aromatase inhibition: LOEC = 1 μM | Laville et al. 2006 |
| Homo sapiens | Cell-free aromatase inhibition assay | 2 | 7 concentrations, 3 orders of magnitude | Aromatase inhibition: IC50 = 3.2 μM | Trösken et al. 2004 |
| Homo sapiens | Cell-free aromatase inhibition assay | 3 | 3 orders of magnitude | Aromatase inhibition: IC50 = 199 μM | Trösken et al. 2006 |
| CHO-K1 cells (Hamsters) | AR antagonist transactivation assay | 1 | 1, 3, 10 30 μM | AR antagonism: 3 μM < IC25 <10 μM | Vinggard et al. 2008 |

**Summary WoE for Estrogen, Androgen, Thyroid and Steroidogenesis Pathways**

This weight of the evidence evaluation used is consistent with the general approach proposed in OECD Guidance Document No. 150: Guidance document on standardized test guidelines for evaluating chemicals for endocrine disruption (OECD, 2012) and follows the hypothesis testing approach described by Borgert et al. (2014). The conceptual framework described in OECD Guidance Document No. 150 is not a testing strategy to be followed from level 1 to level 5. It does organize available study types and can provide guidance as to what additional information might be needed to help understand potential endocrine activity. The conceptual framework levels are:

Level 1 – Existing Data and Non-Test Information

Level 2 – *In Vitro* Assays Providing Data About Selected Endocrine

Mechanism(s)/Pathway(s)

Level 3 - *In Vivo* Assays Providing Data About Selected Endocrine

Mechanism(s)/Pathway(s)

Level 4 – *In Vivo* Assays Providing Data on Adverse Effects on Endocrine-Relevant

Endpoints

Level 5 - *In Vivo* Assays Providing More Comprehensive Data on Adverse Effects on

Endocrine-Relevant Endpoints Over More Extensive Parts of the Life Cycle of

the Organism

A summary of the studies reviewed and evaluated can be found in Appendix S3-1.

***Estrogen Pathway***

To test the hypothesis that propiconazole has the potential to interact as either an agonist or antagonist with components of the estrogen pathway, endpoints relevant for these interactions as outlined in Borgert et al. (2014) were considered, along with additional endpoints from higher level studies that are informative for these hypotheses.

In the high throughput assays (i.e. ToxCast) relevant for the ER model, propiconazole was inactive. In the Conceptual Framework Level 2 ER binding study, propiconazole did not appear to interact with the estrogen receptor using MCF-7 cells (IC50 = 55 μM) except for at the highest concentration (10^-3^ M) which is at or near the limit of solubility for the substance (Kjeldsen et al. 2010). According to a review by the USEPA (USEPA WoE 2015) the response is likely caused by instability in the test system at this high concentration rather than actual receptor interaction, so the data do not provide convincing evidence of binding. Propiconazole was active in a transactivation assay (EC50 = 12 μM), indicating weak estrogen activity (Kjeldsen et al., 2010; Kjeldsen et al., 2013). However, in an assessment of 200 pesticides, propiconazole was not active in a different transactivation assay (Kojima et al., 2003), indicating disparate results for estrogen activity.

Propiconazole has been shown to inhibit aromatase activity in *in vitro* studies with cell lines (LOEC = 30 μM, Goetz et al. 2009; LOEC = 0.1 μM, Kjeldsen et al. 2013; LOEC = 1 μM, Laville et al. 2006) and cell-free systems (IC50 = 3.2 μM, Trösken et al. 2004), although as will be discussed, the response of the estrogen endpoint might not be specifically related to the inhibition of aromatase. In the steroidogenesis assay (Kjaerstad et al. 2010, Goetz et al. 2009) synthesis of both estradiol and testosterone were inhibited by propiconazole at high concentrations (>10 µM).

Moving into Level 3 studies, propiconazole did not increase uterine weight in the uterotrophic assay or produce any consistent estrogenic, anti-estrogenic, or HPG axis effects in rats in the male and female pubertal assays or other relevant regulatory studies (USEPA WoE 2015).

At the highest test concentration (1.0 mg/L) in a guideline fish short term reproduction study (FSTRA) observations of decreased fecundity, fertility, and female vitellogenin (VTG), along with increased gonadal somatic index (GSI) and female gonadal histopathological findings were noted (US EPA WoE 2015). In another 21-day study with fathead minnows, an increase in female GSI and reductions in estradiol, female VTG, and fecundity, with no effects on fertility or hatching success were noted (Skolness et al. 2013).

No treatment-related effects were noted in a guideline compliant conceptual framework level 4 reproduction study with bobwhite quail at concentrations up to 1000 ppm (USEPA WoE, 2015).

Two level 5 fish life-cycle studies are available that can add relevant information to the assessment. No concentration dependent effects were noted on embryo hatchability, length and weight of larvae, development or F0 survival in a sheepshead minnow life-cycle study at concentrations of 0.016, 0.038, 0.068, 0.15, 0.29, and 0.55 mg/L (measured). Fecundity was reduced at > 0.15 mg/L, F1 hatching reduced at 0.29 and 0.55 mg/L, and there was no F1 post-hatch survival in the 0.55 mg/L treatment, although no other adverse effects on survival were noted (EU Review Program 1989). The NOEC for this study is 0.068 mg/L based on the effects on fecundity.

The second fish full life-cycle performed with fathead minnow, included additional endpoints to help better characterize potential endocrine activity (Syngenta 2015). Measured concentrations in this study were 0.0079, 0.021, 0.063, 0.188, and 0.558 mg/L. Effects on reproductive parameters occurred at the 0.558 mg/L concentration, where fecundity and spawning frequency were reduced in both F0 and F1 generations. Additional treatment related effects included an increase in male tubercle score at concentrations > 0.021 mg/L and a statistically significant reduction in vitellogenin concentration at 0.558 mg/L. Histopathological effects were noted in F1 gonads and livers at concentrations > 0.063 mg/L. The NOEC based on reproductive parameters was 0.188 mg/L.

In summary, *in vitro* assays indicate that propiconazole has the potential to inhibit aromatase. However, no evidence of estrogen-related effects in the mammalian studies suggest that the *in vitro* effects on estradiol production are not relevant to mammals. In the FSTRA and the fish life-cycle studies, however, there were effects on estrogen-related endpoints that could potentially be attributed to altered steroidogenesis. However, the hepatotoxicity observed in the life-cycle study could impact vitellogenin production, noted in both the FSTRA and full life-cycle studies, with subsequent effects on reproduction.

***Androgen***

The potential for propiconazole to interact with the androgen pathway was evaluated similarly to the estrogen pathway with a consideration of relevant endpoints identified by Borgert et al. (2014) and the USEPA (USEPA WoE 2015). In the androgen receptor binding study, propiconazole was a weak binder compared to the positive control, dihydrotestosterone (Bauer et al. 2002). Several studies have shown that propiconazole can antagonize the androgen receptor (IC50 = 7.71 μM; RIC20 = 6.2 μM; 3 μM < IC25 <10 μM), as measured by transactivation assays with co-treatment of androgen agonists (Aït-Aïssa et al., 2010; Kjeldsen et al., 2010; Vinggard et al., 2008). Testosterone production was inhibited at high concentrations (> 10 µM) in H295R cells in the steroidogenesis assay (Kjaerstad et al. 2010).

Evaluating the mammalian literature, to further define the weight of evidence testing the hypothesis for androgen pathway interaction, there was some evidence of potential interaction *in* *vivo* from rodent studies where there was an increase in anogenital distance in male rats, increased testosterone concentrations in offspring on post-natal day 92, and increased testes weight at post-natal day 50 (USEPA WoE, 2015). However, testosterone concentrations and absolute testes weights were not affected at other time intervals, and there were no effects on androgen-sensitive secondary sex organ weights. In addition, there were no treatment related responses on endpoints such as sperm measurements, reproduction, or histopathology. No other available regulatory studies in rodents demonstrated any indication of either androgenic or

antiandrogenic effects of propiconazole.

In one fish study, male GSI was significantly increased while tubercle score was decreased and effects on male gonadal histopathology were noted (USEPA WoE, 2015). The highest concentration tested (0.558 mg/L) in a fathead minnow life-cycle study, resulted in an increase in male tubercle score and decreases in fecundity, spawning frequency and female vitellogenin, while hepatotoxicity was noted at lower concentrations (Syngenta 2015). Recent studies investigating the constitutive androstane receptor (CAR) and pregnane X receptor (PXR) mode of action of propiconazole in the liver (Goetz and Dix, 2009; Murphy et al. 2012), suggest that it is possible that the limited effects on endocrine endpoints observed in the *in vivo* studies are modulated by effects on the liver.

In summary, there is some indication that propiconazole can interact weakly with the androgen receptor, possibly as an antagonist. However, results from *in vivo* studies do not provide strong evidence for a direct interaction with the androgen pathway as either an agonist or antagonist. In wildlife, while responses between male and female fish were not necessarily consistent for an interaction with the androgen pathway, as noted for the estrogen pathway, the effects observed could potentially be a result of altered steroidogenesis, or as an indirect result of effects on liver

***Thyroid***

Propiconazole did not demonstrate effects relevant to the thyroid pathway.  High throughput studies for thyroid activity are limited, but no significant activity was noted.  *In vivo* studies that evaluated thyroid activity included a one-generation reproduction study, extended female and male pubertal studies in rodents, and an amphibian metamorphosis assay (USEPA WoE, 2015).  In the rodent studies, there were no effects on thyroid weights or histopathological changes in the thyroid gland.  No significant changes in serum T3, T4 or TSH were noted where measured.  In another *in vivo* study using male rats, Wolf *et al.* (2006) examined propiconazole effects over time on the thyroid.  Sporadic changes were noted but were not deemed to be dose-dependent.  The effects noted in the liver in this study, indicate that any thyroid effect may be secondary to liver enzyme induction.  There were no changes in the thyroid histopathology or any developmental effects in the amphibian metamorphosis assay.  Overall, there is no evidence that suggests propiconazole affects the hypothalamic-thyroid-pituitary axis.

**Endpoint Evaluation in an AOP context**

The adverse outcome pathway (AOP) framework has become an important tool to support ecotoxicology research and risk assessment. In the case of propiconazole, there are several competing AOPs that may be used to explain toxicity pathways leading to endpoints relevant to environmental risk assessment. Of these AOPs, there is direct evidence propiconazole can inhibit aromatase activity (Goetz et al. 2009; Kjeldsen et al. 2013; Laville et al. 2006), which in fish could lead to impaired vitellogenesis and decreased fecundity (Skolness et al., 2013). In addition to this toxicity pathway, there is comparative evidence demonstrating propiconazole increases CAR/PXR activity in mammals (Goetz and Dix, 2009). These changes are shown to increase E2 metabolism, where decreases in plasma levels could impact VTG production and related endpoints in fish. High-throughput screening data also support this toxicity pathway, as propiconazole induces a number of cytochrome P450 enzymes related to E2 metabolism (USEPA WoE, 2015). In addition, the observed hepatotoxicity in the fathead minnow life-cycle study (Syngenta 2015) could also result in reduced female VTG production with subsequent impacts on fecundity. Multiple AOPs that result in the same adverse response make it difficult to definitely conclude that the adverse outcomes associated with the exposure to propiconazole are directly linked to an endocrine mode of action.

**Sources of Uncertainty/Data gaps for hazard and risk assessment**

As a commonly-used fungicide, propiconazole is a data rich substance. Nevertheless, the number of chronic ecotoxicological studies with endpoints specific for endocrine action that are published in the open literature is limited. Based on summaries of regulatory studies and published papers, the potential uncertainties or data needs can be evaluated as follows:

i. *Transgenerational effects*For both hazard and risk assessment, the potential for effects to occur in subsequent generations that are not tested is an uncertainty. In the case of propiconazole, there are long term studies available that provide information on more than just the parental generation. Breteler (1988) indicated in a full life cycle test with sheepshead minnow a higher sensitivity for effects on hatching success of the F1 early life stages compared to the F0 early life stages by a factor of ≥3.7. This could be a result of the impacts on fecundity occurring already at the next lower concentration step. This is further evidenced, in a full life cycle fathead minnow study performed to current guidelines, there were no differences in sensitivity between generations (Syngenta, 2015). Thus, transgenerational apical effects could be addressed in the assessment.

ii. *Sensitive species*

For fish, two full life cycle studies with different species are available (Breteler 1988, Syngenta 2015). The NOEC for both studies was based on fecundity, with that from the fathead minnow being 0.188 mg/L, while the sheepshead minnow NOEC was 0.068 mg/L, just one concentration level lower. For invertebrates, the NOEC from a mysid chronic study was 0.205 mg/L based on survival and reproduction (Hollister 1981), which is similar to the reproductive and survival NOECs from a *Daphnia magna* reproduction study of 0.31 mg/L (LeBlanc and Mastrone 1981). While additional studies with other species will help reduce any species sensitivity uncertainties, the relative concordance of reproductive and survival endpoints suggests that there is not an obvious species bias with exposure to propiconazole.

iii. *Sensitive windows of exposure*

Similar to transgenerational effects, ensuring that sensitive windows of exposure or sensitive life stages are assessed is important to both hazard and risk assessment. Propiconazole currently has been tested in two full life cycle studies in fathead minnow and sheepshead minnow which would cover all potentially sensitive time windows. This information is adequate for risk assessment purposes.

iv. *Potency*

Potency is an important factor in an assessment of potential risks of a substance. Propiconazole has been demonstrated to have relatively low potency concerning specific effects as compared to other DMI fungicides (Schäfers 2007).

v. *Understanding the Dose-Response Curve*

There are no hints of non-monotonic concentration response of apical endpoints or lack of a threshold dose. Thus there seem to be no gaps with dose setting.

vi. *Discerning Mode of Action*

There is uncertainty concerning the linkage of mechanistically informative endpoints to apical effects, as data gaps exist concerning the ability to differentiate between the consequences of aromatase inhibition and, for example, non-endocrine liver responses. For a better understanding of this, more data on liver weight, histopathology and affinity to the different CYP 450 enzymes could be generated. In addition, further work on how the CYP 450 enzymes are being impacted and regulated would be useful.

**Conclusion**

An assessment of the *in vitro* peer-reviewed literature determined propiconazole has limited effects on estrogen and androgen function, but observable effects on aromatase activity. No unusual dose-response functions observed in the large *in vivo* database of both mammalian and wildlife studies that would call into question the ability to determine a threshold concentration. Data to determine appropriate exposure levels in the environment from both monitoring and modeling are available. Thus there does not appear to be any obstacle for defining both effect and exposure endpoints for use in a risk assessment. As a conclusion, there is no scientific reason not to manage propiconazole on the basis of a full risk assessment, utilizing hazard and exposure information.

**References**

Aït-Aïssa S, Laskowski S, Laville N, Porcher JM, Brion F. 2010. Anti-androgenic activities of environmental pesticides in the MDA-kb2 reporter cell line. Toxicol In Vitro. 24: 1979-1985.

Battaglin WA, Sandstrom MW, Kuivila KM, Kolpin DW, Meyer MT. 2011. Occurrence of azoxystrobin, propiconazole, and selected other fungicides in US streams, 2005-2006. Water Air Soil Pollut. 218:307-322.

Bauer, E.R.S., Bitsch, N., Brunn, H., Sauerwein, H., Meyer, H.H.D. 2002. Development of an immunoimmobilized androgen receptor assay (IRA) and its application for the characterization of the receptor binding affinity of different pesticides. Chemosphere 46(7):1107-1115. MRID 48176001.

Borgert CJ, Stuchal LD, Mihaich EM, Becker RA, Bentley KS, Brausch JM, Coady K, Geter DR, Gordon E, Guiney PD, et al. 2014. Relevance Weighting of Tier 1 Endocrine Screening Endpoints by Rank Order. Birth Def Res Part B 101:90-113.

Browne P, Judson RS, Casey W, Kleinstreuer N, Thomas RS. 2015. Screening Chemicals for

Estrogen Receptor Bioactivity Using a Computational Model. Environmental science &

technology. Jun 12.

Currie RA, Peffer RC, Goetz AK, Omiecinski CJ, Goodman JI. 2014. Phenobarbital and propiconazole toxicogenomic profiles in mice show major similarities consistent with the key role that constitutive androstane receptor (CAR) activation plays in their mode of action. Toxicology 321:80-88.

EU Review Program. 1989. EU review program for existing active substances. Propiconazole. Volume 1. Report and Proposed Decision. Plant Production Inspection Centre, Pesticide Division, Helsinki, Finland.

Friedman KP, Papineni S, Marty MS, Yi KD, Goetz A, Rasoulpour RJ, Kwiatkowski P, Wolf DC, Blacker A, Peffer RC. 2016. A predictive data-driven framework for endocrine prioritization: A triazole fungicide case study. Crit Rev Toxicol 46(9):785-833.

Goetz AK and Dix DJ. 2009. Mode of action for reproductive and hepatic toxicity inferred from a genomic study of triazole antifungals. Toxicol Sci. 110(2):449-462.

Goetz, A, Rockett, J., Ren, H., Thillainadarajah, I. and Dix, D. (2009). Inhibition of Rat and Human Steroidogenesis by Triazole Antifungals. Syst. Biol. Reprod. Med. 55: 214-226.

Kjærstad, M. B., Taxvig, C., Nellemann, C., Vinggaard, A. M., and Andersen, H. R. (2010). Endocrine disrupting effects in vitro of conazole antifungals used as pesticides and

pharmaceuticals. *Reprod Toxicol*. 30(4):573-582.

Kjeldsen, L. S., Ghisari, M., and Bonefeld-Jørgensen, E. C. (2013). Currently used pesticides and their mixtures affect the function of sex hormone receptors and aromatase enzyme activity. Toxicol Appl Pharmacol. 272(2):453-464.

Klimisch HJ, Andreae M, Tillmann U. 1997. A systematic approach for evaluating the quality of experimental toxicological and ecotoxicological data. Reg Toxicol Pharmacol. 25(1):1-5.

Kojima, H., Katsura, E., Takeuchi, S., Niiyama, K., and Kobayashi, K. (2004). Screening for estrogen and androgen receptor activities in 200 pesticides by in vitro reporter gene assays using Chinese hamster ovary cells. Environ Health Perspect. 112(5):524-31. MRID 48033008.

Laville, N.P., Brion, F., Hinfray, N., Casellas, C., Porcher, J-M., and Ait-Aissa, S. (2006). Modulation of aromatase activity and mRNA by various selected pesticides in the human choriocarcinoma JEG-3 cell line. Toxicol 228: 98-108. MRID 48074115.

Murphy, L. A., Moore, T., and Nesnow, S. (2012). Propiconazole-enhanced hepatic cell proliferation is associated with dysregulation of the cholesterol biosynthesis pathway leading to activation of Erk1/2 through Ras farnesylation. Toxicol Appl Pharmacol. 260(2):146-154.

OECD. 2012. Guidance document on standardised test guidelines for evaluating chemicals for endocrine disruption no 150. Organisation for Economic Cooperation and Development, Paris, 24-Aug-2012. Available at: http://search.oecd.org/officialdocuments/displaydocumentpdf/

?cote=ENV/JM/MONO(2012)22&doclanguage=en.

Schäfers C. 2007. Expert Report - Assessment of the safety of an extrapolation from growth

data of Early Life Stage- and Juvenile Growth Tests (OECD 210, 204, 215) to the NOEC of Fish Full Life Cycle Tests in the risk assessment of DMI-fungicides. Fraunhofer-Institut, Schmallenberg, Germany.

Skolness SY, Blanksma CA, Cavallin JE, Curchill JJ, Durhan EJ, Jensen KM, Johnson RD, Kahl MD, Makynen EA, Villeneuve DL, et al. 2013. Propiconazole inhibits steroidogenesis and reproduction in the fathead minnow (Pimephales promelas). Toxicol Sci 132:284-297.

Syngenta 2015. Propiconazole M-CA 8 Section 8 Supplement - Ecotoxicological Studies. Submitted to European Food Safety Authority, pesticide dossier. Available from: <http://registerofquestions.efsa.europa.eu/roqFrontend/wicket/page?4>

The Ministry of the Environment , Japan. (2004) FY2003 Survey Results of Water Pollution by Agricultural Chemicals Used at Golf Courses. <http://www.env.go.jp/en/press/2004/1112a.html>

Trosken, E.R., Scholz, K., Lutz, R.W., Volkel, W., Zam, J.A.Z., and Lutz, W.K. (2004). Comparative Assessment of the Inhibition of Recombinant Human CYP19 (Aromatase) by Azoles Used in Agriculture and as Drugs for Humans. Endocrine Res. 3:387-394. MRID 48242002.

Trosken, E.R., Fischer, K., Volkel, W., and Lutz, W.K. (2006). Inhibition of human CYP19 by azoles used as antifungal agents and aromatase inhibitors, using a new LC-MS/MS method for the analysis of estradiol product formation. Toxicol, 219:33-40. MRID 48242001.

USEPA (2006) Propiconazole: Phase 4, HED Chapter of the Re-registration Eligibility

Decision Document (RED). PC Code: 122101. Reregistration Case No. 3125. DP Barcode

D329668.United States Environmental Protection Agency, Health Effects Division. June 28,

2006.

USEPA WoE 2015. EDSP Tier 1 assessments Washington, DC: US EPA. Available from <https://www.epa.gov/endocrine-disruption/endocrine-disruptor-screening-program-edsp-tier-1-assessments#find-results>

Vinggard AM, Niemelia J, Wedebye EB, Jensen GE. 2008. Screening of 397 chemicals and development of a quantitative structure-activity relationship model for androgen receptor antagonism. Chem Res Toxicol. 21:813-823.

Wolf, D., Allen, J., George, M., Hester, S., Sun, G., Moore, T., Thai, S-H., Delker, D., Winkfield, E., Leavitt, S., Nelson, G., Roop, B., Jones, C., Thibodeaux, J. and Nesnow, S. (2006). Toxicity profiles in rats treated with tumorigenic and non-tumorigenic triazole conazole fungicides: propiconazole, propiconazole and myclobutanil. Toxicol. Pathol. 34: 895-902. MRID 48264601.

**Appendix S3-1**

List of citations evaluated for use in the case study after the down-select from the original literature search identifying 3246 papers, provided to the propiconazole case study group. This list also includes unpublished reports identified through review of various regulatory submission summaries, but not identified in the literature search. This list was further evaluated for relevance and data quality for use in the case study. The case study was not a comprehensive evaluation of all data and primarily focused on ecotoxicological studies.

# High throughput/In vitro/cell based

Ait-Aissa S, Laskowski S, Laville N, Porcher JM, Brion F (2010). Anti-androgenic activities of environmental pesticides in the MDA-kb2 reporter cell line. *Toxicol. In Vitro* **24**: 1979-1985.

Anderson AM, Carter KW, Anderson D. Wise MJ (2012). Coexpression of nuclear Receptors and histone methylation modifying genes in the testis: Implications for endocrine disruptor modes of action. *PLOS One.* **7(4)**: 1-11.

Currie, RA, Peffer, RC, Goetz, AK, Omiecinski, CJ, Goodman, JI. 2014. Phenobarbital and propiconazole toxicogenomic profiles in mice show major similarities consistent with the key role that constitutive androstane receptor (CAR) activation plays in their mode of action. *Toxicology*, 321:80-88.

Goetz, AK, Dix, DJ. 2009. Mode of action for reproductive and hepatic toxicity inferred from a genommic study of triazole antifungals. *Toxicol Sci*., 110(2):449-462.

Goetz AK, Rockett JC, Ren H, Thillainadarajah I, Dix DJ (2009a). Inhibition of rat and human steroidogenesis by triazole antifungals. *Systems Biol. Repro. Med*. **55**: 214–226.

Goetz, AK, Bao, W, Schmid, JE, Wood, C, Ren, H, Best, DS, Murrell, RN, Rockett, JC, Narotsky, MG, Wolf DC. Date not shown. Gene Expression profiling in testis and liver of mice to identify modes of action of conazole toxicities. Journal not shown.

Goetz, AK, Bao, W, Ren HZ, Schmid, JE, Tully, DB, Wood, C, Rockett, JC, Narotsky, MG, Sun, GB, Lambert, GR, Thai, SF, Wolf DC, Nesnow, S, Dix, DJ. 2006. Gene expression profiling in the liver of DC-1 mice to characterize the hepatotoxicity of triazole fungicides.  *Toxicol Appl Pharmacol*, 215(3):274-284.

Judson R S; Houck K A; Kavlock R J; Knudsen T B; Martin M T; Mortensen H M; Reif D M; Rotroff D M; Shah I; Richard A M; Dix D J. 2010. In Vitro Screening of Environmental Chemicals for Targeted Testing Prioritization: The ToxCast Project. *Environmental Health Perspectives* 118(4) 485-492

Kjaerstad MB, Taxvig C, Nellemann C, Vinggaard AM, Andersen HR (2010). Endocrine disrupting effects in vitro of conazole antifungals used as pesticides and pharmaceuticals. *Reproduct. Toxicol.* **30**: 573-582.

Kjeldsen LS, Ghisari M and Bonefeld-Jorgensen EC (2013). Currently used pesticides and their mixtures affect the function of sex hormone receptors and aromatase enzyme activity. *Tox. App. Pharmacol.* **272**: 453-464.

[Kojima H](http://www.ncbi.nlm.nih.gov/sites/entrez?Db=pubmed&Cmd=Search&Term=%22Kojima%20H%22%5BAuthor%5D&itool=EntrezSystem2.PEntrez.Pubmed.Pubmed_ResultsPanel.Pubmed_DiscoveryPanel.Pubmed_RVAbstractPlus), [Katsura E](http://www.ncbi.nlm.nih.gov/sites/entrez?Db=pubmed&Cmd=Search&Term=%22Katsura%20E%22%5BAuthor%5D&itool=EntrezSystem2.PEntrez.Pubmed.Pubmed_ResultsPanel.Pubmed_DiscoveryPanel.Pubmed_RVAbstractPlus), [Takeuchi S](http://www.ncbi.nlm.nih.gov/sites/entrez?Db=pubmed&Cmd=Search&Term=%22Takeuchi%20S%22%5BAuthor%5D&itool=EntrezSystem2.PEntrez.Pubmed.Pubmed_ResultsPanel.Pubmed_DiscoveryPanel.Pubmed_RVAbstractPlus), [Niiyama K](http://www.ncbi.nlm.nih.gov/sites/entrez?Db=pubmed&Cmd=Search&Term=%22Niiyama%20K%22%5BAuthor%5D&itool=EntrezSystem2.PEntrez.Pubmed.Pubmed_ResultsPanel.Pubmed_DiscoveryPanel.Pubmed_RVAbstractPlus), [Kobayashi K](http://www.ncbi.nlm.nih.gov/sites/entrez?Db=pubmed&Cmd=Search&Term=%22Kobayashi%20K%22%5BAuthor%5D&itool=EntrezSystem2.PEntrez.Pubmed.Pubmed_ResultsPanel.Pubmed_DiscoveryPanel.Pubmed_RVAbstractPlus) (2004). Screening for estrogen and androgen receptor activities in 200 pesticides by in vitro reporter gene assays using Chinese hamster ovary cells. [*Environ. Health Perspect.*](javascript:AL_get(this,%20'jour',%20'Environ%20Health%20Perspect.');) **112(5)**: 524-31.

Laville N, Balaguer P, Brion F, Hinfray N, Casellas C, Porcher J-M, Ait-Aissa S (2006). Modulation of aromatase activity and mRNA by various selected pesticides in the human choriocarcinoma JEG-3 cell line. *Toxicol.* **228**: 98-108.

Martin M T; Knudsen T B; Reif D M; Houck K A; Judson R S; Kavlock R J; Dix D J. 2011. Predictive model of rat reproductive toxicity from ToxCast high throughput screening. *Biology Of Reproduction* 85(2) 327-339

Padilla, S.; Corum, D.; Padnos, B.; Hunter, D. L.; Beam, A.; Houck, K. A.; Sipes, N.; Kleinstreuer, N.; Knudsen, T.; Dix, D. J.; Reif, D. M. 2012. Zebrafish developmental screening of the ToxCast Phase I chemical library. *Reproductive Toxicology*, 33(2), 174-187

Trosken ER, Fischer K, Volkel W, Lutz WK (2006). Inhibition of human CYP19 by azoles used as antifungal agents and aromatase inhibitors, using a new LC-MS/MS method for the analysis of estradiol product formation. *Toxicol.* **219**: 33-40.

Trosken ER, Scholz K, Lutz RW, Volkel W, Zarn JAZ, and Lutz WK (2004). Comparative assessment of the inhibition of recombinant human CYP19 (aromatase) by azoles used in agriculture and as drugs for humans. *Endocrine Res.* **30(3)**: 387-394.

Tully DB, Bao W, Goetz AK, Blystone CR, Ren H, Schmid JE, Strader LF, Wood CR, Best DS, Narotsky MG, Wolf DC, Rockett JC, Dix DJ (2006) .Gene expression profiling in liver and testis of rats to characterize the toxicity of triazole fungicides. *Tox. App. Pharmacol.* **215**: 260-273.

Vinggaard AM, Niemela J, Wedebye EB, Jensen GE (2008). Screening of 397 chemicals and development of a quantitative structure-activity relationship model for androgen receptor antagonism. *Chem. Res. Toxicol.* **21(4)**: 813-823.

Wambaugh J F; Setzer R W; Reif David M; Gangwal S; Mitchell-Blackwood J; Arnot J A; Joliet O; Frame A; Rabinowitz J; Knudsen T B; Judson R S; Egeghy P; Vallero D; Cohen Hubal E A. 2013. High-Throughput Models for Exposure-Based Chemical Prioritization in the ExpoCast Project. *Environmental Science & Technology* 47(15) 8479-8488

Warrilow AG, Parker JE, Kelly DE, Kelly SL (2013). Azole affinity of sterol 14α-demethylase (CYP51) enzymes from *Candida albicans* and *Homo sapiens*. *Antimicrobial Agents and Chemotherapy* **57(3)**: 1352-1360.

# Fish

Breteler, RJ. 1988 Data Evaluation Record, Fish Life-Cycle Toxicity Stuy. Sheepshead minnow. MRID No. 408820-01 & 401833-10. Available at: https://archive.epa.gov/pesticides/chemicalsearch/chemical/foia/web/pdf/122101/122101-2006-02-06a.pdf

Dong, W, Macaulay, LJ, Kwok, KW, Hinton DE, Stapleton HM. 2013. Using whole mount in situ hybridization to examine thyroid hormone deiodinase expression in embryonic and larval zebrafish: a tool for examining OH-BDE toxicity to early life stages. Aquat Toxicol, 132-133:190-199.

Hinfray, N, Porcher, JM, Brion, F. 2006. Inhibition of rainbow trout (*Oncorhynchus mykiss*) P450 aromatase activities in brain and ovarian microsomes by various environmental substances. Comp Biochem Physiol C - Toxicol Pharmacol, 144(3):252-262.

Isales, GM, Hipszer, RA, Raftery, TD, Chen, A, Stapleton HM, Volz, DC. 2015. Triphenyl phosphate-induced developmental toxicity in zebrafish: potential role of the retinoic acid receptor. Aquat Toxicol, 161:221-230.

K-CA 8.2.2.2/02 (2014): Propiconazole – A Fish Life-Cycle Toxicity Test with the Fathead Minnow (Pimephales promelas), Report Number 528A-249A (Derived from EU regulatory submission document: Propiconazole. NOTIFICATION OF AN ACTIVE SUBSTANCE UNDER COMMISSION REGULATION (EU) 844/2012 DOCUMENT M-CA, Section 8 Supplement. ECOTOXICOLOGICAL STUDIES. Downloaded from EFSA Register of Questions.

Knauer, K.; Lampert, C.; Gonzalez-Valero, J. 2007. Comparison of in vitro and in vivo acute fish toxicity in relation to toxicant mode of action. Chemosphere, 68(8), 1435-1441

Levine, SL, Oris, JT, Denison, MS. 1999. Modulation of CYP1A expression in rainbow trout by a technical grade formulation of propiconazole. *Environ Toxicol Chem*, 18(11):2565-2573.

Li ZH, Zlabek V, Li P, Grabic R, Velisek J, Machova J, Randak T. (2010a). Biochemical and physiological responses in liver and muscle of rainbow trout after long-term exposure to propiconazole. *Ecotoxicology and Environmental Safety* **73(6):** 1391-1396.

Li ZH, Zlabek V, Grabic R, Li P, Randak T. (2010b). Modulation of glutathione-related antioxidant defense system of fish chronically treated by the fungicide propiconazole. *Comparative Biochemistry and Physiology, Part C* **152(3):** 392-398.

Li ZH, Zlabek V, Li P, Grabic R, Velisek J, Machova J, Randak T. (2010c). Effects of exposure to sublethal propiconazole on the antioxidant defense system and Na+–K+-ATPase activity in brain of rainbow trout, *Oncorhynchus mykiss. Aquatic Toxicology* **98(3):** 297-303.

Li ZH, Zlabek V, Grabic R, Li P, Machova J, Velisek J, Randak T. (2010d). Effects of exposure to sublethal propiconazole on intestine-related biochemical responses in rainbow trout, *Oncorhynchus mykiss.* *Chemico-Biological Interactions* **185(3):** 241-246.

Li ZH, Zlabek V, Velisek J, Grabic R, Machova J, Kolarova J, Li P, Randak T. (2011a). Antioxidant responses and plasma biochemical characteristics in the freshwater rainbow trout, *Oncorhynchus mykiss*, after acute exposure to the fungicide propiconazole. *Czech J. Anim. Sci.,* **56 (2):** 61-69.

Li ZH, Zlabek V, Grabic R, Li P, Randak T. (2011b). Biochemical responses in gills of rainbow trout exposed to propiconazole. *Central European Journal of Biology* **6(1):** 84-90

Li, ZH, Velisek, J, Grabic, R, Li, P, Kolarova, J, Randak, T. 2011c. Use of hematological and plasma biochemical parameters to assess the chronic effects of a fungicide propiconazole on a freshwater teleost. Chemosphere, 83(4):572-578.

Li ZH, Zlabek V, Velisek J, Grabic R, Machova J, Kolarova J, Li P, Randak T. (2012) Multiple Biomarkers Responses in Juvenile Rainbow Trout, *Oncorhynchus mykiss*, After Acute Exposure to a Fungicide Propiconazole. *Environmental Toxicology* **28(3): 119-126**

Li , JS, Sun, LB, Zuo, ZH, Chen, M, Geng, H, Wang, CG. 2012. Exposure to paclobutrazol disrupts spermatogenesis in male Sebastiscus marmoratus. Aquatic Toxicol, 122:120-124.

Li, Zhi-Hua; Zlabek, Vladimir; Velisek, Josef; Grabic, Roman; Machova, Jana; Kolarova, Jitka; Li, Ping; Randak, Tomas. 2013. Multiple Biomarkers Responses in Juvenile Rainbow Trout, Oncorhynchus mykiss, After Acute Exposure to a Fungicide Propiconazole. *Environmental Toxicology*, 28, No. 3, pp. 119-126

Scornaienchi ML, Thornton C, Willett KL, Wilson JY, (2010) Cytochrome P450-mediated 17ᵝ-estradiol metabolism in zebrafish (*Danio rerio*). *Journal of Endocrinology*. **210:** 317-325.

Skolness SY, Blanksma CA, Cavallin JE, Curchill JJ, Durhan EJ, Jensen KM, Johnson RD, Kahl MD, Makynen EA, Villeneuve DL, Ankley GT (2013). Propiconazole inhibits steroidogenesis and reproduction in the fathead minnow (*Pimephales promelas*). *Tox. Sci.* **132(2)**: 284-297.

Stanley Morrison, AM, Goldstone JV, Lamb DC, Kubota A, Lemaire B, Stegeman JJ (2013). Identification, modeling and ligand affinity of early deuterostome CYP51s, and functional characterization of recombinant zebrafish sterol 14α-demethylase. *Biochimica et Biophysica Acta*. E-pub ahead of print.

Suprenant DC. 1987. The toxicity of CGA-64250 (Propiconazole) to fathead minnow (Pimephales promelas) embryos and larvae. Study No. 87-8-2476. Springborn Life Sciences, Inc. Wareham, MA. MRID No. 417203-02. https://archive.epa.gov/pesticides/chemicalsearch/chemical/foia/web/pdf/122101/122101-1992-07-22b.pdf

York, D.O. (2012). Propiconazole- Fish Short-Term Reproduction Assay with Fathead Minnow (*Pimephales promelas*). Unpublished study performed by Smithers Viscient, Wareham, MA. Laboratory Study No: 1781.6772, June 14, 2012. Study sponsored by Syngenta Crop Protection, LLC, Greensboro, NC. MRID 48673302

Yu, L, Chen, ML, Liu, YH, Gui, WJ, Zhu, GN. 2013. Thyroid endocrine disruption in zebrafish larvae following exposure to hexaconazole and tebuconazole. *Aquatic Toxicol*, 138:35-42.

# Amphibians

Lee, Michael R. (2012). Propiconazole- Amphibian Metamorphosis Assay with African Clawed Frog (*Xenopus laevis*). Unpublished study performed by Smithers Viscient, Wareham, MA. Laboratory Report No: 1781.6771, June 19, 2012. MRID 48673301.

# invertebrates

Betancourt-Lozano Miguel; Baird Donald J; Sangha Ravinder S; Gonzalez-Farias Fernando. 2006. Induction of morphological deformities and moulting alterations in Litopenaeus vannamei (Boone) juveniles exposed to the triazole-derivative fungicide tilt. *Arch Environm Cont Toxicol*, 51(1):69-78

Bringolf, RB, Cope, WG, Eads, CB, Lazaro, PR, Barnhart MC, Shea D. 2007. Acute and chronic toxicity of technical-grade pesticides to glochidia and juveniles of freshwater mussels (Unionidae). *Environ Toxicol Chem*. 26(10):2086-2093

Coors A, et al., (2014). Predicting effect of wood preservative products in *Daphnia magna* and *Pseudokirchneriella subcapitata* based on the concept of concentration addition. *Environm Toxicol Chem,* Vol. 33, No. 2, pp. 382–393

Elston, C, Thompson, HM, Walters KFA. 2013. Sub-lethal effects of thiamethoxam, a neonicotinoid pesticide, and propiconazole, a DMI fungicide, on colony initiation in bumblebee (Bombus terrestris) micro-colonies. *Apidologie*, 44(5):563-574.

Flatt, T, Heyland, A, Rus, F, Porpiglia, E, Sherlock, C, Yamamoto, R, Garbuzov, A, Palli, SR, Tatar, M, Siverman, N. 2008. Hormonal regulation of the humoral innate immune response in Drosophila melanogaster. *J Exp Biol*. 211

Fournier AE, 2014. Propiconazole – Full Life-Cycle Toxicity Test with Water Fleas, *Daphnia magna*, Under Static-Renewal Conditions, Report Number 1781.6953, Smithers Viscient. 790 Main Street, Wareham, MA 02571-1037, USA. (Summary derived from EU regulatory submission document: Propiconazole. NOTIFICATION OF AN ACTIVE SUBSTANCE UNDER COMMISSION REGULATION (EU) 844/2012 DOCUMENT M-CA, Section 8 Supplement. ECOTOXICOLOGICAL STUDIES. Downloaded from EFSA Register of Questions)

Gao Minling; Song Wenhua; Zhang Jinyang; Guo Jing. 2013. Effect on enzymes and histopathology in earthworm (Eisenia foetida) induced by triazole fungicides. *Environ Toxicol Pharmacol*, (2013 May) Vol. 35, No. 3, pp.427-33.

Hollister TA. 1981. Chronic toxicity of CGA-64250 to mysid shrimp (Mysidopsis bahia). Summarized in EU Review Program. 1989. EU review program for existing active substances. Propiconazole. Volume 1. Report and Proposed Decision. Plant Production Inspection Centre, Pesticide Division, Helsinki, Finland.

Jubeaux G, Simon R, Salvador A, Quéau H, Chaumot A, Geffard O. (2012) Vitellogenin-like proteins in the freshwater amphipod *Gammarus fossarum* (Koch, 1835): Functional characterization throughout reproductive process, potential for use as an indicator of oocyte quality and endocrine disruption biomarker in males. *Aquatic Toxicology* **112-113**: 72-82.

Le Blanc GA, Mastone JD, et al. 1981. The chronic toxicity of CGA-624250 to the water flea (*Daphnia magna*). Unpublished study performed by EG&G Bionomics. Laboratory Report No. BW-81-11-1043. MRID No. 00163165. https://archive.epa.gov/pesticides/chemicalsearch/chemical/foia/web/pdf/122101/122101-2005-09-23b.pdf

Norgaard, KB, Dedergreen, N. 2010. Pesticide cocktails can interact synergistically on aquatic crustaceans. *Environ Sci Pollut Res*, 17(4):957-967.

Soetaert, A, Moens, LN, Van der Ven, K, Van Leemput, K, Naudts, B, Blust, R, DeCoen, WM. 2006. Molecular impact of propiconazole on *Daphnia magna* using a reproduction-related cDNA array. Published paper, *Comparative Biochemistry and Physiology, Part C.* **142:** 66-76.

# terrestrial/plants/rodent

Beavers JB. 1982, One-generation reproduction study - Bobwhite Quail, CGA-64250 Technical, Final Report. Wildlife International Ltd. Laboratory Project No. 108-202. Acc. #072210. MRID No. 00133369. Unpublished. https://archive.epa.gov/pesticides/chemicalsearch/chemical/foia/web/pdf/122101/122101-1984-03-01a.pdf

Borders C and Salamon C, 1985. Two-generation reproduction study in albino rats with CGA-64250 technical. Toxigenics, Inc., 1800 East Pershing Road, Decatur, IL 62526, USA. Laboratory Report No.450-1202 issue date 12 March 1985. Unpublished. MRID No. 00151514.

da Silva, RA, Quintela, ED, Mascarin, GM, Barrigossi, JAF, Liao, LM. 2013. Compatibility of conventional agrochemicals used in rice crops with the entomopathogenic fungus Metarhizium anisopliae. Scientia Agricola, 70(3):152-160.

Fink R., Beavers JB et al. 1982, One-generation reproduction study – Mallard Duck, CGA-64250 Technical, Final Report. Wildlife International Ltd. Laboratory Project No. 108-203. MRID No. 00134502. Unpublished. https://archive.epa.gov/pesticides/chemicalsearch/chemical/foia/web/pdf/122101/122101-2005-09-23a.pdf

Goetz AK et al, 2007. Disruption of testosterone homeostasis as a mode of action for the reproductive toxicity of triazole fungicides in the male rat. *Tox. Sci.* **95(1)**: 227–239.

Hancock, HG, Weete, JD. 1985. Effects of triazoles on fungi. IV. Growth and lipids of cercospora-arachidicola and cercosporidium-personatum. *Pestic. Biochem Physiol* 24(3):395-405.

Hunter B et al, 1982. CGA64250: Potential tumorigenic and toxic effects in prolonged dietary administration to rats. Huntingdon Research Centre, Huntingdon, Cambridgeshire, UK. Laboratory Report No. CBG 193/821113, 30 September 1982 (plus addendum to final report issued 25 June 1985; Laboratory Report No. CBG 193/821113). Unpublished. MRID No. 00129918.

Hunter B et al, 1982. CGA64250: Long-term feeding study in mice. Huntingdon Research Centre Ltd., Huntingdon, Cambridgeshire, UK. Laboratory Report No. CBG/196/81827, 26 October 1982. Unpublished. Acc No. 250784

Hunter B et al, 1984. CGA64250: Long term feeding study in mice (addendum to final report). Huntingdon Research Centre PLC., Huntingdon, Cambridgeshire, UK. Laboratory Report No. CBG 196/821114/2 addendum to CBG/196/81827, 15 November 1984. Unpublished. MRID No. 00129570.

Johansen, NS, Moen, LH, Egaas, E. 2007. Sterol demethylation inhibitor fungicides as disruptors of insect development and inducers of glutathione S-transferase activities in Mamestra brassicae. *Comp Biochem Physiol C: Toxicol Pharmacol*, 145(3):473-483

Johnson W, 1985. One year subchronic oral toxicity study in Beagle dogs with CGA64250 technical. Food and Drug Research Laboratories, Inc., Route 17C, PO Box 107, Waverly, NY 14892-0107, USA. Laboratory Report No. 7737, 28 May 1985. Unpublished. MRID No. 00151515.

Kone, D, Badou, OJ, Bomisso, EL, Camara, B, Ake, S. 2009. In vitro activity of different fungicides on the growth in Mycosphaerella fijiensis var. difformis Stover and Dickson, Cladosporium musae Morelet et Deightoniella torulosa (Syd.) Ellis, isolated parasites of the banana phyllosphere in the Ivoery Coast. *Comptes Rendus Biologies*, 332(5):448-455.

Li, MH, Hansen, LG. 1996. Enzyme induction and acute endocrine effects in prepubertal female rats receiving environmental PCB/PCDF/PCDD mixtures. *Environ Health Perspect*, 104(7)712-722.

Mallows S et al, 1987. CGA64250 technical: A modified teratology (segment II) study in albino rats. Pharmaceuticals Division, Ciba-Geigy Corporation, 556 Morris Ave., Summit, New Jersey 07901, USA. Laboratory Report No. 86189, 06 February 1987. Unpublished. MRID No. 40425002.

Marcsisin et al, 1987. CGA64250 technical: Teratology (segment II) study in rats. Research Department, Toxicology/Pathology Division, Reproductive Toxicology Subdivision, Safety Evaluation Facility, Summit, New Jersey 07901, USA. Laboratory Report No. 86004, 28 January 1987. Unpublished. MRID No. 40425001.

Pilling, ED, Jepson PC. 1993. Synergism between EBI fungicides and a pyrethroid insecticide in the honeybee (Apis mellifera). *Pesticide Science* 39(4):293-297.

Raab DM et al, 1986. CGA64250 technical: Teratology study in rabbits. Research Department, Pharmaceuticals Division, Ciba-Geigy Corporation, Summit, New Jersey 07901, USA. Laboratory Report No. 86043, 01 August 1986. Unpublished. Acc No. 265796.

Rieke S, Schmidt F, Heise T, Pfeil R, Niemann L, Marx-Stoelting P (2013). Triazole fungicide effects on the adrenal gland *in vivo* in a broad dose range. *Naunyn-Schmiedeberg´s Arch. Pharmacol.* **(Suppl. 1)**: S1–S10.

Rockett JC, Narotsky MG, Thompson KE, Thillainadarajah I, Blystone CR, Goetz AK, Ren H, Best DS, Murrell RN, Nichols HP, Schmid JE, Wolf DC and Dix DJ (2006). Effect of conazole fungicides on reproductive development in the female rat. *Repro. Toxicol.* **22**: 647-658.

Ronis, MJJ, Celander, M, Badger, TM. 1998. Cytochrome P450 enzymes in the kidney of the bobwhite quail (Colinus virginianus): induction and inhibition by ergosterol biosynthesis inhibiting fungicides. *Comp Biochem Physiol C: Pharmacol Toxicol Endocrinol*, 121(1-3):221-229.

Sachsse K, 1979. CGA64250: 3-Month toxicity study on dogs. Ciba-Geigy Ltd., Basle, Switzerland. Laboratory Report No. 785751, 09 August 1979. Unpublished. MRID No. 00058606.

Schmidt F, Rieke S, Heise T, Niemann L, Pfeil R, Marx-Stoelting P (2013). Toxic effects of triazole fungicides on rat male reproductive system in prostate gland and testis. *Naunyn-Schmiedeberg´s Arch. Pharmacol.* **(Suppl. 1)**: S1–S10.

Stanley, J, Sah, K, Jain, SK, Bhatt, JC, Sushil, SN. 2015. Evaluation of pesticide toxicity at their field recommended doses to honeybees, Apis cerana and A. mellifera through laboratory, semi-field and field studies. *Chemosphere*, 119:668-674.

Taxvig C et al, 2008. Endocrine-disrupting properties *in vivo* of widely used azole fungicides. *Int. J. Andrology* 31: 170-177.

Wolf, DC, Allen, JW, George, MH, Hester SD, Sun, GB, Moore, T, Thai, SF, Delker, D, Winkfield, E, Leavitt, S, Nelson, G, Roop, BC, Jones, C, Thibodeaux, J, Nesnow, S. 2006, Toxicity profiles in rats treated with tumorigenic and non-tumorigenic triazole conazole fungicides: Propiconazole, triadimefon, and myclobutanil. *Toxicol Pathol*, 34(7): 895-902.

# reviews and other literature

Castillo, LE, Ruepert, C, Solis E. 2000. Pesticide residues in the aquatic environment of banana plantation areas in the north Atlantic zone of Costa Rica. *Environ Toxicol* Chem 19(8):1942-1950.

Christen, V, Crettaz, P, Fent, K. 2014. Additive and synergistic antiandrogenic activities of mixtures of azol fungicides and vinclozolin. *Toxicol Appl Pharmacol*. 279(3):455-466

EU Review Program. 1989. EU review program for existing active substances. Propiconazole. Volume 1. Report and Proposed Decision. Plant Production Inspection Centre, Pesticide Division, Helsinki, Finland.

EU regulatory submission document: Propiconazole. NOTIFICATION OF AN ACTIVE SUBSTANCE UNDER COMMISSION REGULATION (EU) 844/2012 DOCUMENT M-CA, Section 8 Supplement. ECOTOXICOLOGICAL STUDIES. Downloaded from EFSA Register of Questions, <http://registerofquestions.efsa.europa.eu/roqFrontend/wicket/page?4> – Referenced as Syngenta 2015 in this paper.

Goetz AK, Ren H, Schmid JE, Blystone CR, Thillainadarajah I, Best DS, Nichols HP, Strader LF, Wolf DC, Narotsky MG, Rockett JC, Dix DJ (2007). Disruption of testosterone homeostasis as a mode of action for the reproductive toxicity of triazole fungicides in the male rat. *Toxicol. Sci.* **95(1)**: 227–239.

Goetz AK and Dix DJ. 2009. Toxicogenomic effects common to triazole antifungals and conserved between rats and humans. *Tox. App. Pharmacol.* **238**: 80-89.

Kjaerstad MB, Andersen HR, Taxvig C, Hass U, Axelstad M, Metzdorff S, Vinggaard AM (2007). Effects of azole fungicides on the function of sex and thyroid hormones. Pesticides Research 111. Danish Ministry of the Environment, Environmental Protection Agency, Strandgade 29, 1401 Copenhagen K, Denmark.

Kjaerstad M B; Taxvig C; Nellemann C; Vinggaard A M; Andersen H R. 2008. Endocrine-disrupting properties in vivo of widely used azole fungicides. International Journal Of Andrology, 31(2) 170-7

Kwok, IMY, Loeffler RT. 1993. The biochemical mode of action of some newer azole fungicides. Pesticide Science 39(1):1-11.

Taxvig C, Vinggaard AM, Hass U, Axelstad M, Metzdorff S, Nellemann C (2008). Endocrine-disrupting properties *in vivo* of widely used azole fungicides. *Int. J Andrology* **31**: 170-177.

Taxvig C, Hadrup N, Boberg J, Axelstad M, Bossi R, Bonefeld-Jorgensen EC, Vinggaard AM (2013). *In vitro* - *in vivo* correlations for endocrine activity of a mixture of currently used pesticides. *Tox. App. Pharmacol.* **272**: 757-766.

US EPA. 2015. EDSP weight of evidence analysis of potential interaction with estrogen, androgen, or thyroid pathways: Propiconazole. Office of Pesticide Programs and Office of Science Coordination and Policy, US EPA. https://www.epa.gov/sites/production/files/2015-06/documents/propiconazole-122101_2015-06-29_txr0057144.pdf

# General reference

Brown RP, Greer RD, Mihaich EM, Guiney PD (2001). A critical review of the scientific literature on potential endocrine-mediated effects in fish and wildlife. *Ecotoxicol. Environ. Safety* **49**: 17-25.

Beresford AP, (1993). CYP1A1: Friend or Foe? *Drug Metabolism Reviews* **25(4):** 503-517

CEFIC EMSG (1999). Towards the establishment of a weight of the evidence approach to prioritising action in relation to endocrine disruption. CEFIC.

DeVito,M, Biegel L, Brouwer A, Brown S, Brucker-Davis F, Cheek AO, Christensen R, Colborn T, Cooke P, Crissman J, Crofton K, Doerge D, Gray E, Hauser P, Hurley P, Kohn M, Lazar J, McMaster S, McClain M, McConnell E, Meier C, Miller R, Tietge J, Tyl R. 1999. Screening methods for thyroid hormone disruptors. Environ Health Perspect, 107(5):407-415.

Jung, EM, An, BS, Yang, H, Choi, KC, Jeung, EB. 2012. Biomarker genes for detecting estrogenic activity of endocrine disruptors via estrogen receptors. Int J Environ Res Public Health. 9(3):698-711.

Kjaerstadt MB, Taxvig C, Andersen HR, Nellemann C (2010b). Mixture effects of endocrine disrupting compounds *in vitro*. *Int. J. Andrology* **33**: 425-433.

Klimisch HJ, Andreae M, Tillmann U (1997). A systematic approach for evaluating the quality of experimental toxicological and ecotoxicological data. *Reg. Tox. Pharmacol.* **25**: 1-5.

OECD, 2012. OECD Guidance Document No. 150: Guidance document on standardised test guidelines for evaluating chemicals for endocrine disruption. <http://search.oecd.org/officialdocuments/displaydocumentpdf/?cote=env/jm/mono(2012)22&doclanguage=en> (DoA: 20 May 2014).

Rotroff DM, Dix DJ, Houck KA, Knudsen TB, Martin MT, McLaurin KW, Reif DM, Crofton KM, Singh AV, Xia M, Huan R, Judson RS (2013). Using *in Vitro* high throughput screening assays to identify potential endocrine-disrupting chemicals. *Environ. Health Perspec.* **121**: 7-14.

WHO/IPCS (2002). Global assessment of the state-of-the-science of endocrine disruptors. WHO/IPCS/EDC/02.2. <http://www.who.int/ipcs/publications/new_issues/endocrine_disruptors/en/> (DoA: 20 May 2014).

**Appendix S3-2**

**Summary and Evaluation of Studies Identified in the Literature Search for Propiconazole. Some summaries were taken directly from regulatory documents (e.g. US EPA WoE 2015, Syngenta 2015, EU Review Program 1989) as the study reports were not available to the authors.**

# High throughput/In vitro/cell based

| **Published**  **Paper:** | Aït-Aïssa S, Laskowski S, Laville N, Porcher J-M, Brion F. 2010. Anti-androgenic activities of environmental pesticides in the MDA-kb2 reporter cell line. *Toxicology in Vitro*. 24:1979–1985. |
| --- | --- |

**Guidelines:** NA

**GLP:** No

**Klimisch:** 2

**Culture conditions:** 10^4^ cells (MDA-kb2) per well, 24 h at 37 °C in L15 medium (with phenol red)

**Study design:** Transcriptional activity of the androgen receptor (AR) was measured in the MDA-kb2 reporter cell line for 30 pesticides (including propiconazole). MDA-MB-453 cells were stably transfected by the luciferase reporter gene driven by the MMTV promoter, which is up-regulated by the AR and glucocorticoid receptor (GR). Cells were pre-incubated for 24 h at 37° C in L15 medium (with phenol red). After pre-incubation, cells were dosed in triplicate with solvent alone, as well as positive controls (DHT, AR agonism; DHT + flutamide, AR antagonism). Chemicals were first screened with two concentrations (1 and 10 µM), and positive chemicals were tested with additional concentrations (0.01 to 10 µM). After exposure, media was replaced with a phenol red-free solution and luciferase activity was measured. Each chemical was tested in at least three independent studies. In addition, no significant cytoxicity was observed after exposure to individual pesticides, as determined by a MTT assay.

**Results:**

- 14 of 30 pesticides behaved as AR antagonists
- Propiconazole was the 4^th^ weakest AR antagonist with an IC50 of 7.71 µM

**Conclusions and remarks**

- This study has moderate reliability beccuase:
  - Chemical purity was not defined
  - The MMTV promoter is controlled by more than one nuclear receptor (AR and GR)
  - Different number of concentrations were used for each chemical
- Results confirmed published data supporting propiconazole is a weak AR antagonist

| **Published**  **Paper:** | Anderson AM, Carter KW, Anderson D, Wise MJ. 2012. Coexpression of Nuclear Receptors and Histone Methylation Modifying Genes in the Testis: Implications for Endocrine Disruptor Modes of Action. *PLoS ONE*. 7:e34158. |
| --- | --- |

**This study was not reviewed because it did not assess endpoints relevant to endocrine activity.**

| **Published**  **Paper:** | Currie RA, Peffer RC, Goetz AK, Omiecinski CJ, Goodman JI. 2014. Phenobarbital and propiconazole toxicogenomic profiles in mice show major similarities consistent with the key role that constitutive androstane receptor (CAR) activation plays in their mode of action. *Toxicology*. 321:80–88. |
| --- | --- |

**Guidelines:** 104-week carcinogenicity study – USEPA Guideline 83-2(B)

**GLP**: No

**Klimisch**: 2

60-day cell proliferation study in CD-1 mice – NA

CAR3 reporter assay - NA

**Culture conditions:** 104-week carcinogenicity study – Not described, referred to guidelines

60-day cell proliferation study in CD-1 mice – Housed individually in standard cages (macrolon type 2) on soft wood bedding at 22±2 °C, relative humidity of 45-65%, and 12 h light/dark cycle. Animals were acclimatized to lab conditions for 11 days.

CAR3 reporter assay – Primate-derived COS-1 cells, separate lines with human and mouse CAR receptor, CYP2B6 response element fused to luciferase reporter

**Study design:** This report includes multiple experiments with propiconazole including a 104-week carcinogenicity study, 60-day cell proliferation study in CD-1 mice, CAR3 reporter assay, and toxicogenomic data analysis. In the 104-week study, groups of 52 male and females CD-1 mice were treated with propiconazole for two years at dietary concentrations of 0, 100,500, and 2500 ppm. A satellite group was also treated and sacrificed after one year of treatment. After sacrifice, tissues were examined using microscopy and evaluated by an independent pathologist. In the 60-day cell proliferation study, groups of 5 young adult male CD-1 mice were fed propiconazole at dietary concentrations of 0, 850, and 2500 ppm for 1-10 days (1, 2, 3, 4, 7, 14, 28, 60 days). Propiconazole was administered through a pelleted standard rodent chow for *ad libitum* consumption. Animals were sacrificed by bleeding under ether anaesthesia, weighed, and livers were excised and quickly weighed. Tissue slices were obtain from the left lobe, right medial, and lateral liver lobes and processed for histology. Serial sections were prepared from paraffin blocks and stained via hematoxylin and eosin or BrdU staining. In the CAR3 reporter assays, primate-derived COS-1 cells were used to measure human and mouse CAR3 transactivation using a CYP2B6 transcriptional response element and luciferase reporter. Propiconazole was evaluated at 1, 3, 10, and 30 µM for each construct with the appropriate positive controls (CITCO, human CAR3; TCPOBOP, mouse CAR3; clotrimazole, mouse and rat CAR3). Finally, the toxicogenomics data analysis used a common transcriptomic data set downloaded from gene expression omnibus. In this assessment, three different pathway analysis approaches from the literature were used to evaluate difference among these approaches.

**Results:**

- Propiconazole caused hepatocellular proliferation in CD-1 mice
- Propiconazole was a potent activator of the mouse CAR3 nuclear receptor, less potency was observed in human CAR3
- Different pathway analysis tools produced variable results, although some common pathways (CAR/PXR signalling, DAN damage response, cell cycle/proliferation, cholesterol pathway, retinoic acid pathway, oxidative stress, apoptosis, endoplasmic reticulum stress) were observed in most/all analyses

**Conclusions and remarks:**

- The *in vivo* experiments in this study are a reliable source of data because:
  - USEPA guidelines were used for the 104-week carcinogenicity study
  - There was a detailed description of the 60-day cell proliferation study in CD-1 mice
  - Chemical purity was determined
- The CAR3 reporter assays are less reliable because:
  - The cell source was not defined
  - Culture conditions were not described with detail
  - Cytotoxicity was not assessed

| **Published**  **Paper:** | Goetz AK, Bao W, Ren H, Schmid JE, Tully DB, Wood C, Rockett JC, Narotsky MG, Sun G, Lambert GR, Thai S-F, Wolf DC, Nesnow S, Dix DJ. 2006. Gene expression profiling in the liver of CD-1 mice to characterize the hepatotoxicity of triazole fungicides. *Toxicology and Applied Pharmacology*. 215:274–284. |
| --- | --- |

**Guidelines:** NA

**GLP**: No

**Klimisch**: 1

**Culture conditions:** 12:12 light/dark cycle, controlled temperature (72 °C) and humidity (45%), unlimited access to feed and water, singly housed

**Study design:** Adult mice were exposed to propiconazole and three other triazole fungicides to determine hepatotoxicity and gene expression profiles in the liver. Briefly, CD-1 male mice, 35-39 days old, were acclimated for 10 days to culture conditions and placed into experimental groups consisting of 6 mice. Mice were dosed with 10, 75, and 150 mg/kg/day propiconazole once daily by gavage for 14 consecutive days beginning at 51 days of age. Animals in the propiconazole group were sacrificed 24 h after the final exposure via IP injection of Nembutal. Multiple tissues (i.e., liver, thyroid, spleen, adrenals, brain, epididymis, testes, seminal vesicles, and prostate) were excised and weighed immediately after euthanasia. Several pieces of liver were snap frozen in liquid nitrogen for RNA isolation and microarray analysis. In addition, RNA was used for a targeted gene expression analysis using Quantative PCR. Other pieces of the liver were used to prepare microsomes for AROD assays and used for histopathology.

**Results:**

- All triazole fungicides, including propiconazole, caused hepatocyte hypertrophy
- Several biotransformation genes (CYP enzymes, xenobiotic metabolizing enzymes) were differentially expressed from propiconazole and other triazole fungicides
- Differentially expressed biotransformation genes were associated with PXR and CAR at the pathway level

**Conclusions and remarks:**

- Results from this study elucidate gene networks responsible for hepatotoxicity
- Transcriptomic tools were applied with excellent QA/QC
- This study is reliable because:
  - It provided a clear and detailed description of culture conditions and experimental design
  - Chemical purity was defined
- However, Nembutal euthanasia may influence results as this compound can affect the activity of expression of CYPs

| **Published**  **Paper:** | Goetz AK, Dix DJ. 2009. Mode of Action for Reproductive and Hepatic Toxicity Inferred from a Genomic Study of Triazole Antifungals. *Toxicol. Sci.* 110:449–462. |
| --- | --- |

**Guidelines:** NA

**GLP**: No

**Klimisch**: 2

**Culture conditions (Provided in Goetz et al. 2007):** 12 h light/dark cycle, controlled temperature (72 °F) and humidity (45%), unlimited access to feed and water, adults were individually housed, offspring were housed with respective mothers until weaning at PND23, males and female pups were housed by treatment in same-sex pairs until PND50, and males were singly housed after PND50

**Study design:** Timed pregnant Wistar Han IGS rats were feed 500 and 2500 ppm propiconazole in acetone vehicle with 5002 Certified Rodent Diet. Dams began treated feed diets on GD6, and feeding continued through gestation, parturition, and lactation. The F1 generation continued this feeding regimen after weaning at PND23. From each litter, one male was sacrificed on PND92 for transcriptional profiling analysis in the liver and testes. RNA was extracted using TRI Reagent and assessed using a NanoDrop Fluorospectrometer and 2100 bioanalyzer. RNA from the liver or testes of 3-7 individuals was hybridized to Affymetrix GeneChip Rate Genome 230 2.0 plus microarrays. Data were extracted, transformed, and analysed using the JMP Genomics program, and Ingenuity Pathway Analysis was used to evaluate signalling pathways.

**Results:**

- All triazole fungicides, including propiconazole, altered fatty acid, steroid, and xenobiotic metabolism pathways in the liver
- CAR/PXR signalling regulated these pathways with propiconazole exposure
- These changes may underlie reproductive toxicity from disrupted steroid homeostasis

**Conclusions and remarks:**

- This paper is part of a larger study evaluating reproductive toxicity from propiconazole
- Changes in the liver transcriptome elucidated cellular responses to increased serum testosterone levels
- Most transcriptomic changes were observed in the liver, and it was suggested that the testis are not a target organ for triazole reproductive toxicity
- In conjunction with Goetz et al. 2007, this study is reliable because culture conditions, chemical purity, experimental design, and toxicogenomic analyses are explained with detail

| **Published**  **Paper:** | Goetz AK, Rockett JC, Ren H, Thillainadarajah I, Dix DJ. 2009. Inhibition of Rat and Human Steroidogenesis by Triazole Antifungals. *Systems Biology in Reproductive Medicine*. 55:214–226. |
| --- | --- |

**Guidelines:** NA

**GLP**: No

**Klimisch**: 2

**Culture conditions:** H295R human adrenocortical carcinoma cells grown in 75 cm^2^ flasks with 12.5 mL of supplemented medium (Dulbecco’s modified Eagle’s medium with modified Ham’s F-12 mixture) at 37 °C, 5% CO_2_ atmosphere

**Study design:** H295R human adrenocortical carcinoma cells were exposed to 1, 3, 10, 30, and 100 µM propiconazole (DMSO vehicle) in 6-well tissue culture plates. Nu-Serum was replaced with 2.5% charcoal dextran treated FBS to minimize hormone effects from the serum. After a 48 h exposure, media was collected and shipped frozen to USEPA for hormone measurements. Estradiol, progesterone, and testosterone were measured in duplicate using radioimmunoassay kids according to manufacturer’s instructions. Minimal cytotoxicity was observed through microscopic inspection and a live/dead cell viability kit.

**Results:**

- Propiconazole increased estradiol at lower doses (1 and 3 µM), but levels decreased at higher concentrations
- Progesterone and testosterone decreased at higher propiconazole doses

**Conclusions and remarks:**

- Although the H295R adrenocortical carcinoma cell line is useful for characterizing impaired steroidogenesis, related enzymes differ from *in vivo* expression and activity
- This study demonstrates propiconazole can decrease testosterone synthesis *in vitro*, although testosterone typically increases *in vivo*
- This study described culture conditions, experimental design, and controls effectively, but failed to verify chemical purity, decent reliability

| **Unpublished**  **Report** | Goetz AK. 2004. Gene expression profiling in testis and liver of mice to identify modes of action of conazole toxicities. [cited 19 January 2016]. Available from http://repository.lib.ncsu.edu/ir/handle/1840.16/1235. |
| --- | --- |

This study was not reviewed because it is a master’s thesis and is likely part of other published work by this author.

| **Published**  **Paper:** | Judson RS, Houck KA, Kavlock RJ, Knudsen TB, Martin MT, Mortensen HM, Reif DM, Rotroff DM, Shah I, Richard AM, Dix DJ. 2010. In Vitro Screening of Environmental Chemicals for Targeted Testing Prioritization: The ToxCast Project. *Environ Health Perspect*. 118:485–492. |
| --- | --- |

This study was not reviewed because it does not explicitly include propiconazole.

| **Published**  **Paper:** | Kjærstad MB, Taxvig C, Nellemann C, Vinggaard AM, Andersen HR. 2010. Endocrine disrupting effects in vitro of conazole antifungals used as pesticides and pharmaceuticals. *Reproductive Toxicology*. 30:573–582. |
| --- | --- |

**Guidelines:** NA

**GLP**: No

**Klimisch**: 2

**Culture conditions:** MCF-7 cell proliferation assay: Dulbecco’s modified Eagle’s medium with phenol red and 10% FCS, glutamine, HEPES, and gentamicin sulfate 5% CO_2_

AR reporter gene assay: AR-transfected Chinese Hamster Ovary cells with vector pSVARO and the MMTV-LUC reporter plasmid

Steroid synthesis in the human adrenocortical carcinoma cell line H295R: DMEM/F12 medium and 2.0% Nu-serum, 1% ITS + premix, 37 °C in a humidified atmosphere of 5% CO_2_

**Study design:** The estrogenicity of propiconazole (and other triazoles/imidazoles) was first assessed in the MCF-7 proliferation assay. Cells were seeded in 96-well plates at an initial density of 4,500 cells per well. After 24 h incubation, media was replaced with experimental media, test compounds were added in triplicate. Anti-estrogenic effects were tested with co-treatment of E2, and aromatase inhibition was tested with co-treatment of T. After exposure, cells were fixed, dyed, and measured on the plate. Propiconazole was tested in twenty concentrations between 0.001 and 150 µM. Cytotoxicity was assessed using the Cytotox 96 Non-Radioactive Cytotoxicity assay (LDH activity). In the AR reporter gene assay, propiconazole was added in twelve concentrations between 0.025-50 µM to AR-transfected CHO cells in quadruplicate. AR antagonism was measured with co-treatment of R1881. After exposure, luciferase activity was measured and normalized to controls to determine AR activity. Cytotoxicity was assessed as described above. Steroid synthesis was measured in the human adrenocortical carcinoma cell line H295R, where cells were seeded at a density of 3 × 10^5^ cells/well and exposed to azole compounds in triplicate at six concentrations (0.1-30 µM). After 48 h incubation the media was removed and stored (-20 °C) until analysis of hormones with commercial hormone kits. Cytotoxicity was measured by adding resazurin to the cells and measuring fluorescence after 2-4 h.

**Results:**

- Propiconazole inhibited the E2-induced response, indicating an anti-estrogenic effect, however it also induced cell proliferation on its own, indicating weak estrogenic activity
- Propiconazole decreased AR activity from R8811 in the reporter gene assay, indicating AR antagonism
- Propiconazole also reduced testosterone and estradiol production and increased progesterone concentration

**Conclusions and remarks:**

- Conflicting results on estrogenicity, the anti-estrogenic effect is likely due to aromatase inhibition
- For some assays, cytoxicity was close to measured endocrine effects
- The study is reliable from extensive QA/QC measures including:
  - Detailed culture conditions
  - Comprehensive experimental design
  - Defined chemical purity
  - Cytotoxicity assessment

| **Published**  **Paper:** | Kjeldsen LS, Ghisari M, Bonefeld-Jørgensen EC. 2013. Currently used pesticides and their mixtures affect the function of sex hormone receptors and aromatase enzyme activity. *Toxicology and Applied Pharmacology*. 272:453–464. |
| --- | --- |

**Guidelines:** NA

**GLP**: No

**Klimisch**: 2

**Culture conditions:** ER transactivation: MVLN cells cultured at 37 °C in a humidified atmosphere of 5% CO_2_ in phenol red-free Dulbecco’s Modified Eagle’s Medium with supplements

AR transactivation assay: Chinese hamster ovary cell line CHO-K1 co-transfected with MMTV_LUC reporter vector, cultured at 37 °C in a humidified atmosphere of 5% CO_2_ in Dulbecco’s Modified Eagle’s Medium Nutrient Mixture F-12 with phenol red and supplements

Aromatase enzyme activity assay: Human choriocarcinoma JEG-3 cell line cultured at 37 °C in a humidified atmosphere of 5% CO_2_ in Minimum Essential Medium with phenol red with supplements

**Study design:** ER transactivation was measured in MVLN cells carrying an estrogen response element luciferase reporter vector. Cells were seeded in white 96-well plates at a density of 4 × 10^4^ cells per well and exposed to serial dilutions of pesticides (prepared in DMEM with supplements) for 24 h. After exposure, fluorometric measurements of luciferase were made and normalized to protein content. In the AR transactivation assay, CHO-K1 cells were seeded in 96-well plates with a density of 8000 cells per well in DMEM/F-12. Cells were exposed to pesticides for 20 h and luciferase activity was measured at the end of exposure. In the aromatase enzyme activity assay, JEG-2 cells were seeded in 24-well plates at a density of 4 × 10^4^ cells and exposed to pesticides for 18 h. Radio-labelled androgens were used to measure aromatase activity for 2 h with a scintillation counter. Cytotoxicity was assessed in each assay using a cytotoxicity detection kit measuring LDH activity.

**Results:**

- Propiconazole caused weak ER activity, 10^6^-fold less than positive control E2
- AR activity was decreased by propiconazole, indicating AR antagonism
- Aromatase activity decreased at high concentrations of propiconazole, although these effects were near cytotoxicity

**Conclusions and remarks:**

- This report confirms other *in vitro* studies of ER, AR, and aromatase activity by propiconazole
- Excellent QA/QC, reliable paper

| **Published**  **Paper:** | Kojima H, Katsura E, Takeuchi S, Niiyama K, Kobayashi K. 2003. Screening for Estrogen and Androgen Receptor Activities in 200 Pesticides by In Vitro Reporter Gene Assays Using Chinese Hamster Ovary Cells. *Environmental Health Perspectives*. 112:524–531. |
| --- | --- |

**Guidelines:** NA

**GLP**: No

**Klimisch**: 2

**Culture conditions:** CHO-K1 cells, grown in DMEM/F-12 phenol red-free media supplemented with 10% FBS and antibiotics, 37 °C in an atmosphere of 5% CO_2_ with saturating humidity

**Study design:** Cells were plated in 96-well plates at a density of 8,400 cells/well and exposed to 200 pesticides individually, including propiconazole. hERα, hERβ, and hAR reporter gene constructs were used to measure gene transactivation. Compounds with 20% or greater activity of positive controls (E2 and DHT) were considered agonists. Antagonistic activity was measured by co-treating cells with E2 and DHT to measure inhibition. Assays were performed for pesticides at concentrations less than 10^-5^ M to avoid cell toxicity.

**Results:**

- Propiconazole did not induce ER activity for either receptor isoform
- AR antagonism was observed at an RIC of 6.2 × 10^-6^ M

**Conclusions and remarks:**

- This study confirms propiconazole can cause AR antagonism
- Stringent criteria were used to define ER agonists
- Reliable study as chemical purity, study design, and cytoxicity were defined

| **Published**  **Paper:** | Laville N, Balaguer P, Brion F, Hinfray N, Casellas C, Porcher J-M, Aït-Aïssa S. 2006. Modulation of aromatase activity and mRNA by various selected pesticides in the human choriocarcinoma JEG-3 cell line. *Toxicology*. 228:98–108. |
| --- | --- |

**Guidelines:** NA

**GLP**: No

**Klimisch**: 2

**Culture conditions:** JEG-3 choriocarcinoma cells in DMEM supplemented with 10% foetal calf serum, 5% HEPES, 5% non-essential amino acids, 1% antibiotic in 5% CO_2_ at 37 °C

**Study design:** Cells were plated at 50,000 cells/well and exposed to test compounds at three concentrations (1, 3, and 10 µM) for 2 and 24 h. After exposure, aromatase activity was measured using a tritium release assay. Cytotoxicity was assessed using a MMT assay.

**Results:**

- Propiconazole cause a concentration-dependent inhibition of aromatase activity at 2 h
- Aromatase activity did not decrease from any propiconazole concentrations at 24 h

**Conclusions and remarks:**

- There is a temporal gradient of aromatase inhibition by propiconazole
- Effective quality assurance controls were used, providing a reliable study

| **Published**  **Paper:** | Martin MT, Knudsen TB, Reif DM, Houck KA, Judson RS, Kavlock RJ, Dix DJ. 2011. Predictive Model of Rat Reproductive Toxicity from ToxCast High Throughput Screening. *Biol Reprod*. 85:327–339. |
| --- | --- |

This study was not reviewed because it did not produce primary data for propiconazole.

| **Published**  **Paper:** | Padilla S, Corum D, Padnos B, Hunter DL, Beam A, Houck KA, Sipes N, Kleinstreuer N, Knudsen T, Dix DJ, Reif DM. 2012. Zebrafish developmental screening of the ToxCast^TM^ Phase I chemical library. *Reproductive Toxicology*. 33:174–187. |
| --- | --- |

**Guidelines:** NA

**GLP**: No

**Klimisch**: 2

**Culture conditions:** Zebrafish embryos kept in a 26 ± 0.1 °C incubator with 14:10 h light-dark cycle

**Study design:** Zebrafish embryos were placed in 96-well plates 6-8 h after fertilization with one embryo per well. After a single concentration study, embryos were exposed to 11 concentrations of test compounds spanning 5 orders of magnitude. Exposure media (with chemicals) was changed every 24 h. After day 5 of exposure, embryos were washed-out in Hanks’ buffer for 1 day before lethality, hatching, and malformation assessments at 6 dpf. Embryos were considered dead if there were sign of coagulation, decay, or no visible heartbeat. Embryos did not hatch if they remained in the chorion. Malformation criteria included the following assessments: spine, fins, cranial/facial, thorax, abdomen, and position in the water column.

**Results:**

- Based on these three endpoints, propiconazole had an AC_50_ of 26.64 µM
- Of 320 tested chemical, this AC_50_ is ranked 140 of 192 active chemicals

**Conclusions and remarks:**

- High-throughput study provides information about the relative acute and developmental toxicity of propiconazole
- No endocrine endpoints
- Excellent quality assurance and consistency provides reliability

| **Published**  **Paper:** | Trösken E, Scholz K, Lutz R, Völkel W, Zarn JA, Lutz WK. 2004. Comparative Assessment of the Inhibition of Recombinant Human CYP19(Aromatase) by Azoles Used in Agriculture and as Drugs for Humans. *Endocrine Research*. 30:387–394. |
| --- | --- |

**Guidelines:** NA

**GLP**: No

**Klimisch**: 2

**Culture conditions:** NA, cell-free assay

**Study design:** A reaction mixture containing phosphate buffer solutions, human aromatase enzyme, cofactors, and substrates was used to measure aromatase activity. Various azole compounds (including propiconazole) were added to the reaction mixture at 7 concentrations in duplicate, where fluorescence of the substrate dibenzylfluorescein was used to determine aromatase activity.

**Results:**

- Propiconazole inhibited aromatase activity at an IC_50_ of 3.2 µM

**Conclusions and remarks:**

- Assay conditions were not clear
- Chemical purity was not defined

| **Published**  **Paper:** | Trösken ER, Fischer K, Völkel W, Lutz WK. 2006. Inhibition of human CYP19 by azoles used as antifungal agents and aromatase inhibitors, using a new LC–MS/MS method for the analysis of estradiol product formation. *Toxicology*. 219:33–40. |
| --- | --- |

**Guidelines:** NA

**GLP**: No

**Klimisch**: 3

**Culture conditions:** NA, cell-free assays

**Study design:** A reaction mixture containing phosphate buffer solution, enzyme cofactors, and substrate (testosterone) were added with different azole compounds individually. After 5 min pre-incubation at 37 °C, microsomes with human aromatase were added to begin the reaction. The reaction was terminated after 40 min with isopropanol, where protein was reomoved by centrifugation at 15,000 × g for 5 min. The supernatant was removed and analysed by a new LC-APPI-MS/MS method optimized by the analyte estradiol.

**Results:**

- Propiconazole inhibited aromatase activity at an IC_50_ of 199 µM
- This concentration is much higher than previous studies for aromatase inhibition

**Conclusions and remarks:**

- This study is predominately a methods paper
- The IC_50_ of propiconazole is very high in this cell-free assay
- Cell-free assays have less WOE than other *in vitro* studies
- Few details on experimental design, no information on chemical purity

| **Published**  **Paper:** | Tully DB, Bao W, Goetz AK, Blystone CR, Ren H, Schmid JE, Strader LF, Wood CR, Best DS, Narotsky MG, Wolf DC, Rockett JC, Dix DJ. 2006. Gene expression profiling in liver and testis of rats to characterize the toxicity of triazole fungicides. *Toxicology and Applied Pharmacology*. 215:260–273. |
| --- | --- |

**Guidelines:** NA

**GLP**: No

**Klimisch**: 2

**Culture conditions:** Rats were singly housed in polycarbonate cages with pine shaving bedding, 12:12-h light:dark cycle with controlled temperature (72 °F) and humidity (45%) with ad libitum access to feed and water

**Study design:** Adult male SD rats at PND60 were exposed to 10, 75, and 150 mg/kg/day propiconazole (n = 6/group) via oral gavage for 14 days. At the end of exposure, rats were sacrificed by guillotine and necropsied for trunk blood, liver, testes, thyroid, adrenals, brain, epididymis, seminal vesicles, spleen, and ventral prostate. Hormones were measured using radioimmunoassay (T), emzymeimmunoassay (FSH), or fluorometric immunoassay (E2, LH) kits. In addition, sperm motility and morphology were assessed using microscopy. Liver and testes tissue were used for DNA microarrays and qPCR for gene expression profiling.

**Results:**

- Propiconazole increased liver weight at 75 and 150 mg/kg/day and decreased adrenal weight at 150 mg/kd/day
- Histology revealed hepatocyte hypertrophy at higher doses
- Propiconazole did not change T, FSH, E2, or LH levels
- Propiconazole changed the expression of 134 and 51 genes in the liver and testes, respectively, which predominately included genes from biotransformation pathways
- These genes were validated with qPCR

**Conclusions and remarks:**

- There were no measured changes in reproductive endpoints (i.e., hormone levels), although genes related to steroidogenesis were differentially expressed
- Morphological and histological changes indicate systemic toxicity
- This study is highly reliable, but does not report reproductive toxicity for propiconazole

| **Published**  **Paper:** | Vinggaard AM, Niemelä J, Wedebye EB, Jensen GE. 2008. Screening of 397 Chemicals and Development of a Quantitative Structure−Activity Relationship Model for Androgen Receptor Antagonism. *Chem. Res. Toxicol.* 21:813–823. |
| --- | --- |

**Guidelines:** NA

**GLP**: No

**Klimisch**: 2

**Culture conditions:** CHO-K1 cells in DMEM/F12 supplemented with penicillin, streptomycin, amphotericin, and 10% FBS at 37 °C and 5% CO_2_

**Study design:** CHO-K1 cells with a transfected with an AR expression vector (pSVAR0) and reporter plasmid (MMTV-LUC) were exposed to 1, 3, 10, and 30 µM of test compounds (including propiconazole) with the androgen agonist R1881 to measure AR antagonism. After 20 h exposure, cells were lysed and luciferase activity was measured using a luminometer. Data from 397 chemicals were used to develop and validate a QSAR model for AR antagonism.

**Results:**

- Propiconazole was classified with a potency value of “5”, indicating an IC25 between 3 and 10 µM

**Conclusions and remarks:**

- This study confirms propiconazole can act as a weak AR antagonist
- Cytotoxicity methods are unclear
- This study has decent reliability, although chemical purity is not defined

| **Published**  **Paper:** | Wambaugh JF, Setzer RW, Reif DM, Gangwal S, Mitchell-Blackwood J, Arnot JA, Joliet O, Frame A, Rabinowitz J, Knudsen TB, Judson RS, Egeghy P, Vallero D, Cohen Hubal EA. 2013. High-Throughput Models for Exposure-Based Chemical Prioritization in the ExpoCast Project. *Environ. Sci. Technol.* 47:8479–8488. |
| --- | --- |

This study was not reviewed because it does not explicitly include propiconazole.

| **Published**  **Paper:** | Warrilow AG, Parker JE, Kelly DE, Kelly SL. 2013. Azole Affinity of Sterol 14α-Demethylase (CYP51) Enzymes from Candida albicans and Homo sapiens. *Antimicrob Agents Chemother*. 57:1352–1360. |
| --- | --- |

This study was not reviewed because it did not assess endpoints relevant to endocrine disruption.

# FISH

| **Report:** | York, D.O. (2012). Propiconazole- Fish Short-Term Reproduction Assay with Fathead Minnow (*Pimephales promelas*). Unpublished study performed by Smithers Viscient, Wareham, MA. Laboratory Study No: 1781.6772, June 14, 2012. Study sponsored by Syngenta Crop Protection, LLC, Greensboro, NC. MRID 48673302 |
| --- | --- |

**Guidelines:** OCSPP 890.1350, OECD 229

**GLP:** Yes.

**Klimisch**: 1

**Study design:** Fathead minnow were continually exposed under flow-through conditions, to propiconazole (95.2% purity) at mean measured concentrations of 0.010, 0.120, and 1.000 mg/L in a short-term reproduction assay for 21 days. The test was performed with adult fish (16 spawning groups; 2 males and 4 females in each group; ~22 weeks old, mean body weight at test initiation: 3.0 (2.6-3.7) g for males, 1.5 (1.2-1.7) g for females). The test system was maintained at 24 to 26°C and a pH of 6.9 to 7.6.

**Effects on endpoints relevant for assessment of potential for endocrine disruption:**

At the high treatment level, there were significant increases of 13 and 4.2% in female body weight and total length, respectively, compared to the control. There were no effects on male body weight or total length. At the high treatment level, fecundity and fertility were significantly decreased by 68 and 22%, respectively.

Plasma vitellogenin (VTG) was significantly decreased by 82% in females at the high treatment level; male VTG was unaffected. At the high treatment level, gonado-somatic index (GSI) was significantly increased by 59 and 38% in males and females, respectively. Male nuptial tubercle score was significantly decreased in males at the high treatment level. Plasma sex steroid hormones were not measured. Histopathological effects in females included an increased incidence in severity and frequency of oocyte atresia in the high treatment group compared to the negative control. There were sporadic histopathological gonadal effects in males, but these findings were not considered to be treatment-related. The performance and validity criteria were met for this study: spawning was observed in all control replicates at least once every four days.

| York 2012: FSTRA fathead minnow | | | | | |
| --- | --- | --- | --- | --- | --- |
| Concentrations | negative control | Low | medium | | High |
| Mean-measured (µg a.i./L) | <2.93 | 10 | 120 | | 1000 |
| Effects, apical endpoints |  |  |  | |  |
| Overall survival (%) | 100 | 88 | 100 | | 88 |
| Clinical signs | 0 | 0 | 1 male loss of equilibrium | | 1 female bloated |
| Female body weight (g) | 1.31 | 1.29 | 1.33 | | 1.48 * |
| Female total length (mm) | 39.7 | 39.4 | 40.3 | | 41.4 * |
| Male body weight (g) | 3.18 | 2.68 | 2.89 | | 2.88 |
| Male total length (mm) | 52.0 | 49.1 | 50.4 | | 53.3 |
| Fecundity (eggs per female per day per replicate) | 12 | 16 | 17 | | 3.9 * |
| Fertilization success (%) | 98 | 97 | 96 | | 77 * |
| Effects, indicative endpoints |  |  |  | |  |
| Mean Nuptial tubercle score males | 31 | 31 | 33 | | 25 * |
| Mean GSI males (%) | 1.2 | 1.1 | 1.3 | | 2.0 * |
| Mean GSI females (%) | 14 | 14 | 15 | | 19 * |
| Gonadal staging | No difference to negative control | | | | |
| Plasma VTG females | 1.13x10^6^ | 1.22x10^6^ | | 1.06x10^6^ | 0.20x10^6^* |
| Plasma VTG males | 196 | 2228 | | 208 | 480 |
| Sex steroids | Not investigated | | | | |
| Gonad histopath females | - | - | | - | Oocyte atresia |
| Gonad histopath males | No treatment related difference to negative control | | | | |
| Liver histopath | Not investigated | | | | |

| **Report:** | Breteler, RJ. 1988 Data Evaluation Record, Fish Life-Cycle Toxicity Study. Sheepshead minnow. MRID No. 408820-01 & 401833-10. Available at: https://archive.epa.gov/pesticides/chemicalsearch/chemical/foia/web/pdf/122101/122101-2006-02-06a.pdf |
| --- | --- |

**Guidelines:** EPA Guidelines 72-5

**GLP:** Yes.

**Klimisch**: 1

**Study design:** Sheepshead minnow were continually exposed under flow-through conditions, to CGA62450 at mean measured concentrations of 0.55, 0.29, 0.15, 0.068, 0.038 and 0.016 mg/L. Exposure was from F0 egg to F1 egg and a subsequent 28 day post hatch phase in the F1 generation. Total study duration was 100 days.

**F0 –** Hatching success of 200 embryos (100 per replicate) was monitored per concentration until hatching was complete when the larvae were thinned to 100 per concentration (50 per replicate). Mortality and general observations of toxicity were evaluated daily throughout the post-hatch phase. At days 28 (fish thinned to 50 per concentration) and 45 post-hatch length of the fish in all concentrations was photographically determined. Thinned fish from day 28 post-hatch were also measured and weighed. Four reproductive groups (5 females and 2 males) per replicate (two replicates) were initiated after 48 days post-hatch and reproduction was monitored for the duration of the study. At study termination weights and lengths of all remaining fish were determined.

**F1 –** Hatching success, post-hatch survival, weight and length were determined for 100 embryos and larvae of the F1 generation

**Additional short-term exposure –** Additional sheepshead minnows at 14 days post-hatch were also exposed to a short-term (18 days) exposure of CGA62450 at concentrations of 0.29 and 0.55 mg/L. After 18 days the exposure was terminated and the fish grown to sexual maturity where monitored for reproductive performance.

**Effects on endpoints relevant for assessment of potential for endocrine disruption:**

No effects were observed on hatching success or survival of F0, neither on weight and length of F0 larvae. Reproduction was significantly reduced at ≥0.15 mg/L, F1 hatching at ≥0.29 mg/L and F1 28 day post-hatch survival at 0.55 mg/L. Effects on hatching success were observed at concentrations where reproduction was severely decreased and thus this information is likely to reflect the quality of eggs produced. Reproductive performance after short-term exposure was not affected.

| Breteler 1988: FLCT sheepshead minnow | | | | | | | |
| --- | --- | --- | --- | --- | --- | --- | --- |
| Mean measured concentrations (µg a.i./L) |  | 16 | 38 | 68 | 150 | 290 | 550 |
| Effects, apical endpoints | | | | | | | |
| F0 Hatching success (%) |  | - | - | - | - | - | - |
| F0 survival (%) |  | - | - | - | - | - | - |
| F0 juvenile growth |  | - | - | - | - | - | - |
| F0 Clinical signs | Not summarized | | | | | | |
| F0 Reproduction |  | - | - | - | * | * | * |
| F0 body weight (%) | Not summarized | | | | | | |
| F0 total length (%) | Not summarized | | | | | | |
| F1 Hatching success (%) |  | - | - | - | - | * | * |
| F1 survival (%) |  | - | - | - | - | - | * |
| Effects, indicative endpoints | | | | | | | |
| Plasma VTG, Sex steroids | Not investigated | | | | | | |
| GSI | Not investigated | | | | | | |
| Gonad, liver histopath | Not investigated | | | | | | |

| **Report:** | K-CA 8.2.2.2/02 (2014): Propiconazole – A Fish Life-Cycle Toxicity Test with the Fathead Minnow (Pimephales promelas), Report Number 528A-249A (Derived from EU regulatory submission document: Propiconazole. NOTIFICATION OF AN ACTIVE SUBSTANCE UNDER COMMISSION REGULATION (EU) 844/2012 DOCUMENT M-CA, Section 8 Supplement. ECOTOXICOLOGICAL STUDIES. Downloaded from EFSA Register of Questions. |
| --- | --- |

**Guidelines:** Designed with elements of: OPPTS 890.1500 (*draft)* Fish full life cycle toxicity; OECD Draft Proposal for Fish Two-Generation Test Guideline (2002), OPPTS Guideline 890.1350 (2009), OECD Guideline 229 (2009)

**GLP:** Yes.

**Klimisch**: 1

**Taken from M-CA-8 Summary:**

The chronic toxicity of propiconazole to fathead minnow (*Pimephales promelas*) was assessed through constant exposure to five concentrations of propiconazole and a water control. Mean measured concentrations in the study were 7.8, 21, 63, 188 and 558 μg/L. Propiconazole solutions were analysed using high performance liquid chromatography with tandem mass spectrometric detection (LC/MS/MS).

The F0 phase of the fish full life-cycle was initiated (according to OECD 229) by placing 8 males and 16 females per concentration divided equally among four replicates. Eggs from spawns ≥50 eggs from this reproductive phase were pooled and used to initiate the F1 phase. The F1 phase consisted of 200 eggs divided equally amongst 4 replicates – due to significant reproductive effects in the highest concentration the F1 generation was initiated with control eggs for this concentration. On day 214 of the study (196 post-hatch F1) F1 reproductive groups were formed (in independent aquaria) with 8 males and 16 females divided equally amongst 4 replicates.

Dissolved oxygen, temperature, pH, total hardness, total alkalinity and specific conductance were measured at least weekly.

The biological endpoints evaluated in the first generation (F0) were survival, fecundity, fertility and spawning frequency. The second generation (F1) endpoints measured were survival, hatching success, photographic length at Day 28 and 56 post-hatch; and lengths, weights and histological sex on all thinned fish at Day 192 post-hatch (at formation of reproductive groups). Reproductive fish from the F1 generation were monitored for the following endpoints: egg production, spawning frequency, lengths, weights, histological determination of sex, secondary sex characteristics, plasma vitellogenin concentration, and gonadal and liver histopathology. Survival and hatching success were monitored for the F2 generation.

Observations and measurements of fathead minnow were used to estimate the overall mean measured test concentration producing no adverse effect on the exposed organisms (No-Observed-Adverse Effect Concentration, NOAEC).

In addition to the exposed groups recovery of organisms from short (21 day) and long-term (192 days) exposure was monitored in specific groups. Fish from F0 reproductive groups showing statistically impaired reproduction were not terminated at day 21 but instead were moved into recovery without further exposure, survival, fecundity, fertility and spawning frequency were determined. At formation of the F1 reproductive groups extra reproductive groups were formed in test vessels without exposure to propiconazole, egg production, spawning frequency, lengths, weights, histological determination of sex, secondary sex characteristics, plasma vitellogenin concentration, and gonadal histopathology were determined.

**Results:**

There were 36 weekly analytical sampling intervals scheduled during this fish full life cycle study that lasted over 253 days. Measured concentrations of samples collected during this lengthy time period were within 20% of the mean measured values in all test concentrations.

Results are reported as mean measured concentrations.

*F0 Generation:*

There were no effects on survival at any concentrations. Effects on reproductive parameters were

restricted to the highest concentration (558 μg/L) where a decrease in cumulative eggs, eggs/female/day and spawning frequency were observed. No effects on fertility were observed in any concentrations.

The effects observed in highest concentration returned to levels comparable with the control fish shortly after moving the fish into the recovery phase.

*F1 Generation:*

Survival and hatch of the F1 generation was not affected at any concentration tested throughout the F1 generation. Statistically significant effects in length were observed in all concentrations at various time points throughout the study. Although the increases in length were statistically significant, it was not dose responsive, the increase was light, ranging from 1 to 7% and there was no comparable increase in weight. In addition this variation in length is considered, within this testing laboratory, to be within the range of normal biological variability. No effects were observed on sex ratio at any concentration tested. Statistically significant reproductive effects (cumulative number of eggs, eggs/female/day, and spawning frequency) were observed at the highest tested concentration (558 μg/L). No effects on fertility were observed at any concentration tested.

Additional treatment related effects include increase in male tubercle score at and above concentrations of 21 μg/L and statistically significant reduction in vitellogenin concentration at 558 μg/L. Histopathological effects in the gonads and liver of the F1 Generation (Day 235) were restricted to:

- Interstitial cell hyperplasia / hypertrophy and increased spermatozoa in the testes of males
- Decreased yolk formation, decreased post-ovulatory follicles, and decreased mean ovarian stage scores in the ovaries of females
- Hepatocyte nuclear pleomorphism, hepatocyte anisocytosis, hepatocellular necrosis, increased numbers of multinucleated hepatocytes, cystic degeneration, and pigmented macrophage aggregates in the livers of both sexes
- Reduced hepatic basophilia in the livers of females only

The lowest concentration at which histological effects were observed (interstitial cell hyperplasia /hypertrophy in males) was 63 μg/L.

*F2 Generation:*

Hatching and survival of the F2 generation was unaffected at all concentrations tested.

**Conclusions**

Based on mean measured concentrations and adverse effects on reproductive endpoints, the NOAEC for propiconazole was 188 μg/L.

| FLCT fathead minnow | | | | | | |
| --- | --- | --- | --- | --- | --- | --- |
| Mean measured concentrations (µg a.i./L) | Dilution water | 7.8 | 21 | 63 | 188 | 558 |
| Effects, apical endpoints | | | | | | |
| F0 Reproduction |  | - | - | - | - | * |
| F0 body weight (%) |  | - | - | - | - | - |
| F0 total length (%) |  | - | - | - | - | - |
| F1 Hatching success (%) |  | - | - | - | - | - |
| F1 survival (%) |  | - | - | - | - | - |
| F1 juvenile growth |  | - | - | - | - | - |
| F1 sex ratio |  | - | - | - | - | - |
| F1 Reproduction |  | - | - | - | - | * |
| F1 time to first spawn |  | - | - | - | - | * |
| F1 body weight (%) |  | - | - | - | - | - |
| F1 total length (%) |  | - | - | - | - | - |
| F1 male plasma VTG |  | - | - | - | - | - |
| F1 female plasma VTG decrease |  | - | - | - | - | * |
| F1 Nuptial tubercle score males increase |  |  | * | * | * | * |
| F2 Hatching success (%) |  | - | - | - | - | - |
| F2 survival (%) |  | - | - | - | - | - |

| **Published Paper:** | Skolness SY, Blanksma CA, Cavallin JE, Curchill JJ, Durhan EJ, Jensen KM, Johnson RD, Kahl MD, Makynen EA, Villeneuve DL, Ankley GT (2013). Propiconazole inhibits steroidogenesis and reproduction in the fathead minnow (*Pimephales promelas).* Tox. Sci. 132(2): 284-297 |
| --- | --- |

**Guidelines:** OCSPP 890.1350, OECD 229 with additional indicative endpoints

**GLP:** No.

**Klimisch**: 2

**Study design:** 21-day flow-through study with fathead minnows, 5-6 months old, 14-day acclimation before exposure. 12 pairs (replicates) per concentration, 20 L total volume, flow through rate: approx. 3.5 times/day.

| Skolness et al. 2013: FSTRA+; fathead minnow | | | | | | |
| --- | --- | --- | --- | --- | --- | --- |
| Mean measured concentrations (µg a.i./L) | Ctrl | | 5.8 | 53 | 563 | 1056 |
| Effects, apical endpoints | | | | | | |
| Survival, Clinical signs, male/female body weight | |  | - | - | - | - |
| Fecundity reduced | |  | *^1^ | - | * | * |
| Fertility | |  | - | - | - | - |
| F1 Hatching success (%) | |  | - | - | - | - |
| Effects, indicative endpoints | | | | | | |
| GSI males, females, (increase) | |  | - | - | * | * |
| Female plasma VTG decrease | |  | - | * | * | * |
| Female E2 decrease | |  | - | - | * | * |
| Female plasma cholesterol decrease | |  | * | * | * | - |
| Male plasma cholesterol | |  | - | - | - | - |
| Male/female plasma triglyceride | |  | - | - | - | - |
| Gonad histopath females | |  | - | - | - |  |
| Ex vivo E2 increase | |  | - | - | * | - |
| Ex vivo testosterone | |  | - | - | - | - |
| Liver histopath | | Not investigated | | | | |
| Gonad tissue gene expression | | | | | | |
| Ovary *fshr* mRNA increase (testes – no effect) | |  | * | - | * | * |
| Ovary testosterone production rate | |  | - | - | - | - |
| Ovary E2 production rate increase | |  | - | - | * | - |
| Female testicular testosterone decrease | |  | - | * | - | * |
| Ovary *hmgr* mRNA decrease (testes – no effect) | |  | - | - | * | * |
| Ovary *cyp51* mRNA decrease (testes – no effect) | |  | - | - | * | * |
| Ovary *cyp17* mRNA increase | |  | - | - | * | * |
| Male testicular *cyp17* mRNA increase | |  | - | - | * | - |
| Ovary *cyp19a1a* mRNA increase (male – not tested) | |  | - | - | * | * |
| Ovary *cyp11a* mRNA increase (testes – no effect) | |  | - | - | - | * |
| Ovary *star* mRNA increase (testes – no effect) | |  | - | - | * | * |
| Male/female low dens lipid *ldlr* mRNA | |  | - | - | - | - |
| Liver tissue gene expression | | | | | | |
| Female *cyp1a1* mRNA increase | |  | * | * | * | * |
| Male *cyp1a1* mRNA increase | |  | - | - | * | * |
| Male *cyp3a* mRNA increase | |  | - | - | * | * |
| Female *cyp3a* mRNA increase | |  | - | - | - | * |
| Male *cyp51* mRNA increase (female – no effect) | |  | - | - | - | * |
| Female *hmgr* mRNA decrease | |  | - | - | * | * |
| Male *hmgr* mRNA decrease | |  | - | - | - | * |
| Male *fdps* mRNA decrease (female – no effect) | |  | - | - | * | * |
| Male fatty acid synthase (*fasn*) mRNA down | |  | - | - | - | * |

**^1^ Authors note that the reliability of this statistically significant reduction is uncertain given that there was no dose response and the lack of other endocrine endpoint responses in this treatment group.**

# Amphibians

| **Report:** | Lee, Michael R. (2012). Propiconazole- Amphibian Metamorphosis Assay with African Clawed Frog (*Xenopus laevis*). Unpublished study performed by Smithers Viscient, Wareham, MA. Laboratory Report No: 1781.6771, June 19, 2012. MRID 48673301. |
| --- | --- |

**Guidelines:** OECD 231; EPA 890.1100

**GLP:** Yes

**Klimisch score:** 1

- 21-day amphibian metamorphosis assay using African clawed frogs (Xenopus laevis)
- Flow-through conditions
- Amphibian larvae (80/control and treatment group; 20 per replicate) at Nieuwkoop-Faber (NF) Stage 51 were exposed to propiconazole (95.2% purity)
- Nominal propiconazole concentrations of 0 (negative control), 0.0060, 0.060, and 0.60 mg a.i./L; mean-measured concentrations were <0.00293 (<LOQ; negative control), 0.0056, 0.056, and 0.57 mg a.i./L.
- The test system was maintained at 21 to 23°C and a pH of 7.3 to 8.1.

The study followed the guidelines with the following exceptions:

- Recommended photoperiod is 12:12 and 16L:8D was used
- Detailed mortality data was not provided

All performance and validity criteria were met.

EPA did not consider either of these deviations affected the acceptability of the study

**EPA Summary:**

Results:

- Survival at study termination was 95% for the negative control and 75% in all propiconazole-treated groups.
- Spinal deformities at test termination were observed in 78% of negative control tadpoles and 80, 77, and 71% of tadpoles in the low, mid, and high treatment groups, respectively; however, these effects were not considered to be treatment-related.
- At the high treatment level, snout-to-vent length (SVL) was significantly decreased (p<0.01, Dunnett’s) by 9 and 5% and body weight was significantly decreased (p<0.01, Dunnett’s) by 20 and 17% on Days 7 and 21, respectively.
- No effects on Day 7 or Day 21 hind-limb length (HLL) at any treatment level relative to the negative control.
- No significant acceleration or delay (p>0.05) of median NF developmental stage on either Day 7 or 21 at any treatment level
- No asynchronous development was observed in the negative control or treatment groups.
- There were no remarkable histopathological findings in the negative control or treatment groups.
- Only effects were increased mortality and decreased growth at the high dose, which although not reflected in the EPA review, may indicate non-endocrine specific toxicity

# invertebrates

| **Published**  **Paper:** | Coors A. (2014) Predicting Acute and Chronic Effects of Wood Preservative Products in *Daphnia Magna* and *Pseudokirchneriella Subcapitata* Based on the Concept of Concentration Addition. *Environmental Toxicology and Chemistry* **33(2):** 382-393 |
| --- | --- |

**Guidelines:** OECD 211 (version 2008)

**GLP:** No.

**Klimisch**: 2

**Culture conditions:** T: 20±2^o^C, photoperiod: 18L/6h; Semi-static: media renewed three times weekly

**Study design:** The chronic effects of propiconazole on the reproduction of *Daphnia magna* was determined over 21 days under static-renewal conditions (renewal 3 times per week) also using a seven nominal concentration geometric dilution series (highest concentration 180 µg /L), alongside a dilution water control with at least 10 replicates (each replicate being 1 individual female) per treatment. Daphnids were always fed after being transferred to fresh medium. The food level in the first series of tests amounted to 0.03 mg C/daphnid/d to 0.06 mg C/daphnid/d throughout the test(low food level), while the food level in the second series of tests started with 0.1 mg C/daphnid/d and increased to 0.2 mg C/daphnid/d (high food level) from day 5 onward. Mortality and number of living offspring were recorded daily for each individual Daphnia. To confirm nominal concentrations of the test substances, samples were taken from the highest, a medium, and the lowest test concentration level at the start of each test.

**Results:**

- good agreement between measured and nominal concentrations 104.6% (SD=21.5%) .
- NOEC and EC10 at low food level for the offspring number are 34.6 and 25.6 µg/L, respectively
- At high food level, no effects on reproduction at the highest tested dose (180 µg propiconazole/L), i.e. NOEC/EC10 > 180 µg/L.

| **Report:** | Fournier AE, 2014. Propiconazole – Full Life-Cycle Toxicity Test with Water Fleas, *Daphnia magna*, Under Static-Renewal Conditions, Report Number 1781.6953, Smithers Viscient. 790 Main Street, Wareham, MA 02571-1037, USA. (Summary derived from EU regulatory submission document: Propiconazole. NOTIFICATION OF AN ACTIVE SUBSTANCE UNDER COMMISSION REGULATION (EU) 844/2012 DOCUMENT M-CA, Section 8 Supplement. ECOTOXICOLOGICAL STUDIES. Downloaded from EFSA Register of Questions) |
| --- | --- |

**Guidelines:** OECD Guidelines for Testing of Chemicals, Guideline 211: Daphnia magna Reproduction test (2012)

US EPA Ecological Effects Test Guidelines, OCSPP 850.1300: Daphnia Chronic Test (1996)

US EPA Ecological Effects Test Guidelines, OPPTS 850.1000: Special Considerations for Conducting Aquatic Laboratory studies (1996)

**GLP:** Yes

**Klimisch**: 1

**Study design:** Individually housed daphnids (10 replicates per concentration) were continually exposed for 21 days under static renewal conditions to 5 mean measured concentrations of 0.18, 0.37, 0.73, 1.5 and 2.9 mg propiconazole/L, and a diluent water control. Determinations of adult survival, offspring production were made at regular intervals throughout the study. Production of male offspring was assessed for the second brood of each concentration and control. In addition length and weight of all surviving animals were assessed at test termination. Results were based on mean measured concentrations.

**Results:**

- Survival was significantly reduced at 2.9 mg/L
- Growth was significantly reduced at 0.73 and 1.5 mg/L
- Reproduction was significantly reduced in remaining individuals at 1.5 mg/L
- No males were produced in any treatment level.

No males were produced during the study at any treatment level and as a sensitive endocrine endpoint this is strong evidence for no effects on the endocrine system. Reproduction was reduced at a concentration below that at which survival was affected. However there were also growth effects at the same and lower concentrations which is suggestive of systemic toxicity.

| **Published**  **Paper:** | Jubeaux G., et al, 2012. Vitellogenin-like proteins in the freshwater amphipod Gammarus fossarum (Koch, 1835): Functional characterization throughout reproductive process, potential for use as an indicator of oocyte quality and endocrine disruption biomarker in males. *Aquatic Toxicololgy.* 112-113 (2012) 72-82. |
| --- | --- |

**Guidelines:** None.

**GLP:** No

**Klimisch**: 3

**Culture conditions:** T: 12±1^o^C, photoperiod: 16L/8h; Semi-static: media renewed every day

**Study design:** Wild caught male *Gammarus fossarum* (21 per concentration, 3 replicates per treatment) were exposed to propiconazole over 21 days at nominal concentrations of 0.001, 0.1, 10 and 1000 µg/L as well as water and acetone (0.005%) controls. At the end of exposure individuals were weighed and frozen in liquid nitrogen until measurement of Vg (a vitellogenin-like protein) was conducted. Individuals were subsequently homogenised and an extraction procedure followed to extract Vg samples for LC-MS/MS analysis. Inductions of male Vg levels were analysed for statistical significance against controls

**Results:**

- no mortality was observed, with survival rates higher than 80%
- a small increase of Vg in males observed only at the concentration of 0.1 µg/L; but, no effects were seen at higher concentrations of 10 and 1000 µg/L.

**Conclusions and remarks**

- Reliability of the study: not reliable because the concentrations were not measured
- This study is the first example of such measurements in males. Thus the reliability and accuracy of detecting such small inductions could be questioned considering the low levels in which this protein is likely to be expressed in males. Not considered to be a relevant effect.
- The function of Vtg in males is not clear.

| **Published**  **Paper:** | Kast-Hutcheson K., Rider CV, and LeBlanc, G.A. 2001. The fungicide propiconazole interferes with embryonic development of the crustacean Daphnia magna. *Environmental Toxicology and Chemistry* **20:** 502-509 |
| --- | --- |

**Guidelines:**

American Society for Testing and Materials. 1988. *Standard Guide for Conducting Renewal Life-Cycle Toxicity Tests with* Daphnia magna. E1193. Philadelphia, PA, USA, pp 1–17.

**GLP:** No

**Klimisch**: 3

**Culture conditions:** T: 20^o^C, photoperiod: 16L/8h; Semi-static: media renewed three times weekly

**Study design:** The chronic effects of propiconazole on the reproduction of *Daphnia magna* was determined over 21 days under static/renewal conditions, with 10 replicates (each replicate being 1 individual female) per treatment at nominal concentrations of 0.015, 0.03, 0.06, 0.12 and 0.25 mg/L as well as ethanol controls (0.0005%, v/v). Survival, molts, days to reproductive maturity and number of offspring were recorded.

**Results:**

- No adverse effects were observed on survival, molts, days to reproductive maturity and number of offspring. NOECs for these endpoints are ≥ 0.25 mg/L.
- The incidence of embryo toxicity was significantly elevated at the concentrations of 0.12 and 0.25 mg/L. The NOEC for embryo toxicity is 0.06 mg/L.

**Conclusions and remarks**

- Reliability of the study: not reliable because the concentrations were not measured.
- The authors further studied the embryoic toxicity and concluded that propiconazole interferes with the later stages of daphnid embryonic development, and that this toxicity is manifested largely via maternal exposure to the fungicide.

| **Report:** | Le Blanc GA, Mastone JD, et al. 1981. The chronic toxicity of CGA-624250 to the water flea (*Daphnia magna*). Unpublished study performed by EG&G Bionomics. Laboratory Report No. BW-81-11-1043. MRID No. 00163165. https://archive.epa.gov/pesticides/chemicalsearch/chemical/foia/web/pdf/122101/122101-2005-09-23b.pdf |
| --- | --- |

**Guidelines:** the study was performed before the first version of TG211 in 1984.

**GLP:** Yes (1978)

**Klimisch**: 2

**Culture conditions:** T: 22±1^o^C, photoperiod: 16L/8h; flow-through

**Study design:** The chronic effects of propiconazole on the reproduction of *Daphnia magna* was determined over 21 days under flow-through conditions, with 4 replicates per treatment (20 daphnids per test replicate) at measured concentrations of 0.05, 0.14, 0.31, 0.69 and 1.3 mg/L as well as water and triethylene glycol (0.046 mL/L) controls. Adult survival, time to first brood release and number of offspring were recorded.

**Results:**

- NOEC for mortality was 0.31 mg/L, with survival around 90% for controls and lower concentration groups, 85% for the group of 0.69 and 0% for the group of 1.3 mg/L.
- Reproduction was retarded at the 0.69 mg/L with the first offspring observed on day 12; whereas offspring were first produced on day 8 and 9 in the controls and groups of lower concentrations. NOEC for reproduction was 0.31 mg/L.

| **Published Paper:** | Soetaert A., et al, 2006. Molecular impact of propiconazole on *Daphnia magna* using a reproduction-related cDNA array. Published paper, *Comparative Biochemistry and Physiology, Part C.* **142:** 66-76. |
| --- | --- |

**Guidelines:** None.

**GLP:** No

**Klimisch**: 3

**Culture conditions:** T: 20±1^o^C, photoperiod: 14L/10h; Semi-static: media renewed three times weekly

**Study design:** Acute assessment according to OECD 202 was conducted at 0.32, 1, 3.2, 10 and 32 mg/L to generate EC_50_ values in order to select doses for the chronic (8-day) study.

Three replicates containing 25 neonate daphnia were exposed to concentrations of 0.25, 0.5 and 1 mg/L dissolved in acetone (0.001%) along with a control and solvent control. After four days exposure 7 daphnids from each replicate were measured for length and 18 were used for gene expression analysis (among others genes for vitellogenin). The same process was repeated with an 8 day exposure but in addition neonates were collected and observed for abnormalities.

**Results:**

- a decrease in length after 4 and 8 days exposure to 1 mg/L
- an increase in embryo abnormalities in first brood neonates after 8 days exposure to 1 mg/L.
- downregulation of genes coding for vitellogenin after 4 days exposure at 1 mg/L
- No difference in regulation of genes coding for vitellogenin after 8 days exposure at any concentration

**Conclusions and remarks**

- Reliability of the study: not reliable because the concentrations were not measured
- This is a microarray study, in which genes are not always affected in a concentration-dependent way. A link is made in this publication between the down regulation of vitellogenin and the abnormalities observed in embryos. It is not clear whether the down regulation at 4 days was not as a result of reduced growth and slower maturation or from another mechanism. In addition the reduction in vitellogenin gene expression was not observed in those individuals who were observed as having abnormal neonates. In addition effects on growth are observed in adults at both time points and thus general toxicity is likely to be interfering with any mechanistic results.

| **Published Paper:** | Betancourt-Lozano Miguel; Baird Donald J; Sangha Ravinder S; Gonzalez-Farias Fernando. 2006. Induction of morphological deformities and moulting alterations in Litopenaeus vannamei (Boone) juveniles exposed to the triazole-derivative fungicide tilt. Archives of environmental contamination and toxicology, 51(1):69-78 |
| --- | --- |

**Guidelines:** None.

**GLP:** No

**Klimisch**: 3

**Culture conditions:** T: 28± 1^o^C, photoperiod: 12L/12h; flow through, sea water, 35 ± 1‰

**Study design:** Juveniles of Pacific white shrimp, Litopenaeus vannamei (1.14 ± 0.42 g) were exposed to the Tilt, the commercial formulation of propiconazole, at concentration of 0, 0.367, 0.459, 0.55, 0.633, 0.706, 0.78 and 0.825 mg/L for 32 days (Note, analytical measurement showed mean recoveries ± 1 SD (%) of propiconazole were 91.2 ± 5% (n = 6). All Tilt treatments are reported as actual concentrations of the active compound propiconazole). Intermoult duration was evaluated as an endpoint by individually recording the time of moulting events. Qualitative behavioural observations and mortality were also recorded during the experiment. At the end of the exposure, the animals were preserved in the fixative for studying morphological abnormalities, including rostrum, paraeopods, and uropods (hereinafter called deformities). Additional deformities were found in the setal development of maxillipeds (mouthparts), uropods, antennal scale, and telson, but no quantification was performed.

**Results:**

- Mortality occurred only at 0.367, 0.706, and 0.825 mg/L, but at relatively low levels of 10, 5, and 15%, respectively.
- Tilt treatments of 0.459, 0.55, 0.633, 0.706, and 0.825 mg/L different from controls (indicating that those treatments presented longer intermould periods), but no differences for the treatments of 0.367 and 0.78 mg/L.
- Concentration dependent morphological abnormalities (rostrum, paraeopods, and uropods)

**Conclusions and remarks**

- Reliability of the study: not reliable because there is no statistical analysis on deformities.
- Tilt 250E (commercial grade, 25% propiconazole asactive compound) formulation are emulsifiers (3.5% of calcium dodecylphenylsulphonate, 4.5% of castor-oil polyglycolether, 2% of oleyl polyethoxyethanol), 4% of deionised water, and 60.2% of solvents (a mixture of unspecified aromatic hydrocarbons). Based on the discussion of the paper, both propiconazole and adjuvants may elicit toxicity.
- NOEC could not be determined because of the effect on intermolt is not concentration dependent and there is not statistical analysis on deformities.

| **Published Paper:** | Elston, C, Thompson, HM, Walters KFA. 2013. Sub-lethal effects of thiamethoxam, a neonicotinoid pesticide, and propiconazole, a DMI fungicide, on colony initiation in bumblebee (Bombus terrestris) micro-colonies. Apidologie, 44(5):563-574. |
| --- | --- |

**Guidelines:** None.

**GLP:** No

**Klimisch**: 2

**Culture conditions:** T: 21^o^C, photoperiod: not mentioned;

**Study design:** This study investigated the effects of propiconazole on nest building or brood production in queenless *Bombus terrestris* micro-colonies in the laboratory. Bees were exposed to honey water and pollen paste containing propiconazole (23, 230 mg/kg) for 28 days. Solvent control micro-colonies were fed honey water solution with 2,000 μg/kg of acetone and pollen dosed with 2,000 μg/kg acetone (the same level of acetone as in the treatments). Untreated micro-colonies were fed honey-water solution and untreated pollen dough throughout the experiment. The quantity of honey water solution consumed was assessed by weighing and replacing the feeders at 2-day intervals. Food consumption was calculated from the difference between feeder weight at the start and end of each 2-day period, and these were combined to calculate the total consumption over the 28-day experimental period. It was not possible to assess pollen consumption due to the incorporation of the pollen supplied into the nest by the bees. Daily assessments were made of worker mortality, nest building activity and egg laying, and the behaviour of the bees was noted (including lack of co-ordination, aggression, stumbling and grooming behaviour).

**Results:**

- propiconazole treatments and both controls remained active throughout the experiment and displayed no uncoordinated movement or excessive grooming.
- Both doses reduced consumption of honey water solution.
- Mortality during the 28-day period was not affected.
- The number of brood (eggs and larvae) was decreased by the treatments

| **Report:** | Hollister TA. 1981. Chronic toxicity of CGA-64250 to mysid shrimp (Mysidopsis bahia). Summarized in EU Review Program. 1989. EU review program for existing active substances. Propiconazole. Volume 1. Report and Proposed Decision. Plant Production Inspection Centre, Pesticide Division, Helsinki, Finland. |
| --- | --- |

**Guidelines:** None

**GLP:** Yes

**Klimisch**: 2

**Study design:** Mysid shrimp (5 per vessel, 4 replicates per concentration) were continually exposed for 28 days under flow-through conditions to 5 mean measured concentrations of propiconazole (0.054, 0.114, 0.205, 0.507 and 0.882 mg/L) and a solvent control. Determinations of adult survival and offspring production were made at regular intervals throughout the study. Results were based on mean measured concentrations.

**Effects on endpoints relevant for assessment of potential for endocrine disruption**

- Survival was significantly reduced at 0.507 and 0.882 mg/L
- Reproduction was significantly reduced in remaining individuals at 0.507 and 0.882 mg/L

Effects on survival occur at the same concentrations as those at which reproductive effects are observed therefore general toxicity is considered to be driving effects.

| **Published**  **Paper:** | Norgaard, KB, Dedergreen, N. 2010. Pesticide cocktails can interact synergistically on aquatic crustaceans. *Environ Sci Pollut Res*, 17(4):957-967. |
| --- | --- |

This paper is not summarised because it is an acute toxicity study.

| **Published**  **Paper:** | Bringolf, RB, Cope, WG, Eads, CB, Lazaro, PR, Barnhart MC, Shea D. 2007. Acute and chronic toxicity of technical-grade pesticides to glochidia and juveniles of freshwater mussels (Unionidae). *Environ Toxicol Chem*. 26(10):2086-2093 |
| --- | --- |

This paper is not summarised because there is no chronic toxicity data on propiconazole available.

| **Published**  **Paper:** | Flatt, T, Heyland, A, Rus, F, Porpiglia, E, Sherlock, C, Yamamoto, R, Garbuzov, A, Palli, SR, Tatar, M, Siverman, N. 2008. Hormonal regulation of the humoral innate immune response in Drosophila melanogaster. *J Exp Biol*. 211 |
| --- | --- |

This paper is not summarised because it did not test propiconazole.

| **Published Paper:** | Gao Minling; Song Wenhua; Zhang Jinyang; Guo Jing. 2013. Effect on enzymes and histopathology in earthworm (*Eisenia foetida*) induced by triazole fungicides. *Environ Toxicol Pharmacol*, 35(3):427-33. |
| --- | --- |

This paper is not summarised because it is an acute toxicity study.

# terrestrial/plants/rodent

| **Report:** | Beavers JB. 1982, One-generation reproduction study - Bobwhite Quail, CGA-64250 Technical, Final Report. Wildlife International Ltd. Laboratory Project No. 108-202. Acc. #072210. MRID No. 00133369. Unpublished. https://archive.epa.gov/pesticides/chemicalsearch/chemical/foia/web/pdf/122101/122101-1984-03-01a.pdf |
| --- | --- |

**Guidelines:** None but generally conforming to: OECD 206

**GLP:** Yes.

**Klimisch: 1**

**Study design:**

- Pairs of reproducing bobwhite quail exposed to dietary concentrations of propiconazole (25, 100, 300 and 1000 mg/kg) and a corn oil control.
- Birds were exposed for 20 weeks.
- Birds were observed for signs of mortality, abnormal behaviour (daily), body weight (weeks 0, 2, 4, 6, 8 and 20), egg production, egg shell thickness, egg quality, viability of embryos, hatchability, number and weight of hatchlings, hatchling survival and gross pathology.

**Results**

Chronic data for the bobwhite quail (MRID 00133369) showed that no treatment related effects were observed at any of the test levels up to 1000 ppm-diet. Only one mortality was observed, in the highest test level, during week 20. In the re-registration eligibility document (2006) and the data evaluation record (Beavers 1982) EPA agreed with the author’s conclusions of no treatement related effects up to the high dose of 1000 ppm.

| **Report: Beavers and Fink 1982, 1 gen quail** | Fink R., Beavers JB et al. 1982, One-generation reproduction study – Mallard Duck, CGA-64250 Technical, Final Report. Wildlife International Ltd. Laboratory Project No. 108-203. MRID No. 00134502. Unpublished. https://archive.epa.gov/pesticides/chemicalsearch/chemical/foia/web/pdf/122101/122101-2005-09-23a.pdf |
| --- | --- |

**Guidelines:** None but generally conforming to: OECD 206

**GLP:** Yes.

**Klimisch:** According to US EPA evaluation, which appears to have been performed 23 years after the study was conducted, the rationale for that designation is that the treated feed was not analyzed and there was a high level of mortality in the 14-day old survivors of eggs set in the control group. This level of mortality however was similar across the treatment levels and may reflect a response that was typical at that time. There was no statistically-significant effect of treatment on adult birds or reproductive responses detected.

**Study design:**

- Pairs of reproducing mallard ducks exposed to dietary concentrations of propiconazole (25, 100, 300 and 1000 mg/kg) and a corn oil control.
- Birds were exposed for 20 weeks.
- Birds were observed for signs of mortality, abnormal behaviour (daily), body weight (weeks 0, 2, 4, 6, 8 and 20), egg production, egg shell thickness, egg quality, viability of embryos, hatchability, number and weight of hatchlings, hatchling survival and gross pathology.

**Results**

Chronic data for the mallard duck (MRID 00134502) showed that no treatment related effects were observed at any of the test levels up to 1000 ppm-diet. Although EPA deemed the study not valid, the lack of any reproductive effect and the concordance with the bobwhite quail reproduction study suggests that propiconazole is not having any chronic effect on mallard duck.

| **Report: Beavers and Fink 1982, 1 gen quail** | Borders C and Salamon C, 1985. Two-generation reproduction study in albino rats with CGA-64250 technical. Toxigenics, Inc., 1800 East Pershing Road, Decatur, IL 62526, USA. Laboratory Report No.450-1202 issue date 12 March 1985. Unpublished. MRID No. 00151514. |
| --- | --- |

**Guidelines:** OECD 416 (2001): Draft FIFRA guidelines (1982)

**GLP:** Yes.

**Klimisch**: 1

**Study design:**

- Propiconazole was administered continuously to groups of Charles River CD rats/sex/dose in diet at dose levels of 0 (control), 100, 500 or 2500 ppm.
- Following a 12 week pre-mating period, mating was initiated with the P males and females (1 male to 2 females) for up to 20 days to produce the “a” litters.
- Mating trials for the “b” litters were initiated approximately 2 weeks following completion of the “a” litters (males paired with females different to those to provide “a” litters and no sibling pairings). Females that failed to produce an “a” litter were not rebred.
- When breeding was confirmed, the females were housed individually.
- Gravid animals were allowed to deliver their litters and the litters were weaned at 21 days of age.
- 15 males and 30 females were selected from the F1b progeny at weaning to serve as the F1 parental animals.
- After a 12 week pre-mating period, these F1 parents were treated as described above to produce F2a and F2b litters.

**EPA Summary**

Results

- No compound-related clinical observations or mortality were reported in parental animals.
- Female body weights in the F0 and F1 generation were significantly reduced in the high dose group at most intervals (↓6-18%); body weight gains were also significantly reduced during pre-mating (12 weeks) as well as gestation and lactation periods (↓15-23%). Correspondingly, high dose females also had significantly reduced food intake (↓12-17%).
- In the F0 and F1 generation male body weights were reduced in the high dose groups compared to controls (↓4-11%) although significance was attained only once in each generation; body weight gains in this group were decreased by 6-9% for the premating period and during the entire duration of the study (76 months). Food consumption was reduced significantly in high dose F0 males at week 1 (65% of the control) and week 7 (86% of the control) and in high dose F1 males and females at week 2, 6 and 10 (84-88% of controls).
- Hepatic “cellular swelling” was significantly increased in mid-dose males and high-dose males and females of the F0 generation. In the F1 parental animals, increase in the incidence of this finding was significant for both sexes in the mid- and high-dose groups.
- The incidence of “hepatic clear-cell change” was significantly increased in F0 high-dose males, F1 mid-dose and high-dose males and F1 high-dose females (p<0.05).
- The LOAEL for parental toxicity was at 500 ppm (42 mg/kg/day) and the NOAEL for parental toxicity was 100 ppm (8 mg/kg/day).
- No effects on reproductive parameters (mating, fecundity, gestation, male and female fertility indices, litter resorptions and gestation duration).
- No treatment-related changes were noted in absolute or relative weights of the testes (with epididymides) or ovaries, and no histopathological lesions were noted in the testes, seminal vesicle, prostate, ovaries, uterus or vagina.
- No effects in the number and percent of viable pups at birth and surviving through weaning for either the F1a and F1b litters. In the F2a litters, however, the number of pups delivered, delivered viable and surviving to day 4 of lactation were significantly reduced in the high-dose group. The percentages of high-dose pups delivered viable and surviving to day 4 were also reduced. The F2b litters of these dams had significantly reduced survival rates (both number and percent of surviving pups) at lactation days 7, 14, and 21. The mean body weights of high-dose progeny were significantly reduced at days 14 and 21 for pups of both generations (72-81% of controls). Reductions were also significant on days 4 and 7 (except for F1b litters) and at birth (F2b litters only).
- At necropsy, no treatment related anomalies, organ weight changes and gross pathology findings were noted in pups. Histopathological evaluation of selected organs from F1b and F2b progeny revealed significantly (p<0.01) increased incidences of hepatic “cellular swelling” in high-dose males and females. This was considered to be a compound related effect.
- The LOAEL and NOAEL for developmental toxicity were 2500 ppm (192-263 mg/kg/day) and 500 ppm (43-52 mg/kg/day), respectively, based on decreased offspring survival and body weights and an increased incidence of hepatic lesions (cellular swelling).

A principal finding was histopathological changes in the liver.

Overt toxicity was noted at the high dose (2500 ppm; 238-263 mg/kg/day) based on decreased survival of the F2 pups. In addition, female body weights in the F0 and F1 generations were significantly reduced in the high dose group at most intervals (↓6-18%), and body weight gains were also significantly reduced during pre-mating (12 weeks) as well as gestation and lactation periods (↓15-23%). Male body weights were also reduced in the high dose group compared to controls (↓4-11%) although significance was attained only once in each generation. Liver toxicity was noted in both parental animals and offspring at the mid- and high dose, and consisted of histopathological changes (hypertrophy).

| **Report: Beavers and Fink 1982, 1 gen quail** | Raab DM et al, 1986. CGA64250 technical: Teratology study in rabbits. Research Department, Pharmaceuticals Division, Ciba-Geigy Corporation, Summit, New Jersey 07901, USA. Laboratory Report No. 86043, 01 August 1986. Unpublished. Acc No. 265796. |
| --- | --- |

**Guidelines:** EPA 83-3.

**GLP:** Yes.

**Klimisch:** 1

**Study design:**

- Propiconazole was administered to three groups of 19 pregnant New Zealand White rabbits at doses of 100, 250 or 400 mg/kg once daily by gavage during gestational days 7-19 inclusive.
- A fourth (control) group received an equivalent volume (5 mL/kg/day) of vehicle only (3% cornstarch in 0.5% Tween 80).
- On gestational day 20, all dams were sacrificed and a laparohysterectomy was performed followed by an examination of the reproductive tract and its contents.
- The females were examined daily for clinical signs of toxicity and weighed on GD 0, 7, 10, 14, 20, 24 and 29; food consumption was measure daily from GD 5 through 29.
- On GD 29, all surviving females were sacrificed and examined for any macroscopic abnormalities in the reproductive tract, the number of corpora lutea in each ovary, gravid uterine weight, number of live fetuses, and placental weight. Fetuses were weighed and examined for external malformation/variations. Each fetus was examined viscerally by fresh dissection and the sex determined. All carcasses were eviscerated and processed for skeletal examination.

**EPA Summary**

Results

- Decreases in food consumption and body weight were noted in in mid- and high-dose females.
- At the high dose, propiconazole induced early delivery and abortion in 5/19 pregnant rabbits.
- Although there appeared to be an increase in the value of mean number of resorptions, the apparent increase was heavily influenced by one completely resorbed litter.
- No treatment-related changes were noted in pregnancy rate, number of corpora lutea, number of resorptions implantations, number of live fetuses/litter, fetal sex ratio, or soft tissue abnormalities at any dose.
- There was no effect on mean fetal weights
- There were no significant differences in visceral and skeletal malformations between the treated and control fetuses. Although increased incidence of the formation of 13th rib was observed, this incidence was shown to be associated with maternal toxicity in other studies. The experimental results did not show any increase in the incidence of cleft lip or palate in treated animals.
- The LOAEL for maternal toxicity is 250 mg/kg based on reduced body weight and food consumption during dosing, and the NOAEL is 100 mg/kg. The LOAEL for developmental toxicity is 400 mg/kg/day base on increased incidences of abortion and fetuses/litter with 13th rib, and the NOAEL is 250 mg/kg/day.
- No overt toxicity was noted in the developmental toxicity rabbit study, and the only finding of systemic toxicity were reductions in food consumption and body weights in the mid- and high-dose groups (250 and 400 mg/kg/day).

| **Report: Beavers and Fink 1982, 1 gen quail** | Mallows S et al, 1987. CGA64250 technical: A modified teratology (segment II) study in albino rats. Pharmaceuticals Division, Ciba-Geigy Corporation, 556 Morris Ave., Summit, New Jersey 07901, USA. Laboratory Report No. 86189, 06 February 1987. Unpublished. MRID No. 40425002. |
| --- | --- |

**Guidelines:** EPA 83-3.

**GLP:** Yes.

**Klimisch**: 1

**Study design:**

- Propiconazole was evaluated for its embryotoxic and teratogenic potential (specifically cleft palate).
- Propiconazole was orally dosed to a single group of 189 pregnant (sperm positive) Crl:COBS CD (SD)BR rats at a dose of 300 mg/kg bw/day on days 6 to 15 of gestation (the day sperm was present in the vaginal washing was designated day 0 of gestation).
- A control group of 178 pregnant rats received vehicle (3% cornstarch in Tween 80) only. All rats were dosed at a rate of 10 mL/kg body weight.
- On GD 20, the surviving dams were euthanized and subjected to gross necropsy.
- All fetuses were weighed, sexed and examined only for gross external alterations.

**EPA Summary**

- Four of the treated dams died during the study, and severe compound-related maternal toxicity was observed at 300 mg/kg/day, including significantly increased incidences of ataxia, coma, lethargy, prostration, audible respiration, labored respiration, and salivation.
- Body weights in the treated group significantly decreased by 3% on GD 20 and overall body weight gain was reduced by 17%.
- Body weight gains were significantly decreased in the mid and high-dose groups by 56 and 62%, respectively on GD 6-8, and food consumption was significantly decreased in the mid- and high dose groups at several intervals.
- No treatment-related changes were seen in the pregnancy rate, number of corpora lutea, number of resorptions /implantations, or fetal sex ratio at 300 mg/kg/day.
- The fetal weight of both males and females was significantly reduced by 5% at 300 mg/kg/day.
- Fetuses were examined for external abnormalities only and there were no statistically treatment-related, external, gross observations among fetuses.
- Cleft palate was reported in 2/2064 fetuses of dosed animals and 0/2122 of control fetuses. The incidence of cleft palate in controls for all teratology studies (not including this one) conducted at this laboratory during 1983-1985 was 0/5431. EPA concluded that this minor incidence at a dose that was maternally toxic and caused reductions in fetal weight did not indicate teratogenicity.

| **Published**  **Paper:** | Goetz AK et al, 2007. Disruption of testosterone homeostasis as a mode of action for the reproductive toxicity of triazole fungicides in the male rat. *Tox. Sci.* **95(1)**: 227–239. |
| --- | --- |

**Guidelines:** None.

**GLP:** No.

**Klimisch**: 2

**Study design:**

- Groups of time mated Wistar Han rats were fed diet containing 0 (control), 100, 500 or 2500 ppm propiconazole from gestation day 6, continuing through gestation, parturition, and lactation. Dams were allowed to deliver naturally, with day of delivery designated as postnatal day (PND) 0 for the F1 offspring. On PND8, litters were weighed and then culled to eight pups per dam, retaining males preferentially, to maximize uniformity in growth rates.
- Dam feed intake and body weights were measured weekly during gestation and lactation periods and at necropsy.
- The F1 generation continued on the same treated diets at weaning on PND23 until PND120. F1 offspring feed intake and body weights were measured weekly until necropsy.
- Offspring food consumption was measured from PND 23 to termination.
- Anogenital distance was measured on PND 0, and PPS was determined in the
- males, beginning on PND 38, and continuing until complete cleavage of the epithelium lining the prepuce of the penis was observed.
- Body weights were recorded on the day this criterion was attained.
- Blood was collected on PND 22, 50, 92, and 99 and analyzed for serum hormone levels. Estradiol, testosterone, total T3, total T4, and TSH levels were determined by radioimmunoassay; luteinizing hormone (LH) levels were measured by fluorometric immunoassay.
- One male from each litter was randomly selected for necropsy on PND 1, 22, 50, or 92.
- Terminal body weights were recorded, and the brain, hypothalamus, hippocampus, pituitary, thyroid, liver, ventral prostate, testis, epididymis and seminal vesicles were removed, weighed, fixed, stained and examined microscopically. One cauda epididymis from each rat was used for determination of sperm morphology and motility.
- Mating assays were conducted using PND 78 or older treated males with untreated virgin PND 56 or older females. These females were acclimated for 10 days and daily vaginal smears were taken to determine estrus cycle stage. Mating behavior was observed at 5 and 20 minutes after pairing.
- Females were euthanized and necropsied with removal of the intact uterus and ovaries on GD 13-16 to evaluate fertility outcome. Ovaries were examined for gross abnormalities and corpora lutea were counted. The numbers of live fetuses and resorptions were counted, and the fetuses were examined for gross abnormalities. Uteri not containing fetuses or having visible resorption sites were stained with 2% ammonium sulfide to visualize implantation sites.

Results:

- The study authors noted that dams exposed to 2500 ppm propiconazole had decreased food consumption during the last two weeks of gestation (data not provided), but recovered close to control levels during lactation.
- In the 2500 ppm male offspring, body weights were slightly decreased (p<0.05) by 5-8% during PND 22-92 (except PND 23-29), and food consumption was sporadically decreased (p<0.05) by 8-9% during PND 30-64.
- The mean compound intakes (calculated by the reviewers) for these animals during PND 23-92 at dietary concentrations of 100, 500, and 2500 ppm were 10.1, 48.9, and 243.7 mg/kg/day, respectively.
- Litters exposed to 2500 ppm propiconazole had survival rates comparable to controls (93.48% treated vs. 95.65% controls), with only 4 of 20 litters having deaths and 1 total litter loss.
- There were no treatment-related deaths in pups that survived beyond PND 8.
- Anogenital distance at PND 0 was increased (p<0.05) in the 2500 ppm male pups, with an increasing (p<0.013) trend across doses.
- The time to preputial separation was similar to the controls in the 2500 ppm male pups (43.2 days treated vs. 42.5 days controls), although these pups had slightly decreased (p<0.05) body weights on the day of attainment (↓5%) and on PND 43 (↓9%).
- At 2500 ppm, relative (to body) liver weights were increased (p<0.001) by 16% on PND 1, absolute and relative liver weights were increased by 17-26% on PND 50, and relative liver weights were increased (p<0.001) by 24% on PND 92.
- Relative, but not absolute, testes weights were increased (p<0.05) at 2500 ppm by 7% on PND 22 and by 13% on PND 50. Absolute and relative testes weights were increased (p<0.05) by 13-19% at 500 ppm on PND 50. However, no other changes in testes weights were noted at PND 0, 22 or 92, and there were no other effects on weight of the epididymis or the accessory sex organs. No histopathological changes were noted in the testes or epididymis.
- Slight decreases (p<0.05) in relative brain weights were noted at 100 ppm on PND 22 (↓16), at 100 and 500 ppm on PND 50 (↓6-7%), and at 500 ppm on PND 92 (↓4%).
- Microscopically, centrilobular and slight midzonal hepatocyte hypertrophy were noted in 4/5 males treated with 2500 ppm propiconazole on PND 50, and mild centrilobular hepatocyte hypertrophy was observed in 2/5 males at 2500 ppm on PND 92.
- Serum testosterone levels were increased (p<0.05) at 500 and 2500 ppm on PND 92; serum estradiol and LH levels were unaffected by treatment.
- Total T4, total T3, and TSH levels were unaffected by treatment.
- There were no effects of treatment with propiconazole on sperm morphology or motility, and no effects of treatment were observed on breeding performance. There were no effects of treatment on the total number of implantation sites, live or dead fetuses, live or dead embryos, or the number of resorptions. All successful pregnancies produced normal, healthy litters with little to no post-implantation loss.

No overt toxicity was noted in the study, but liver weight was increased by 16-26% at the high dose, along with an increase in the incidence of hepatocyte hypertrophy. The authors concluded that the reproductive effects observed in these studies were consistent with the disruption of testosterone homeostasis as a key event in the mode of action for triazole-induced reproductive toxicity. However, the effect noted on relative, but not absolute, testes weight at two time points in the middle of the study but not other time points in the high dose, without any other corroborating responses, or consistent change in serum testosterone, suggests that these minor apparent effects on isolated endpoints are unlikely to reflect an actual effect of treatment and most likely reflect normal biological variation.

| **Report:** | Hunter B et al, 1982. CGA64250: Potential tumorigenic and toxic effects in prolonged dietary administration to rats. Huntingdon Research Centre, Huntingdon, Cambridgeshire, UK. Laboratory Report No. CBG 193/821113, 30 September 1982 (plus addendum to final report issued 25 June 1985; Laboratory Report No. CBG 193/821113). Unpublished. MRID No. 00129918. |
| --- | --- |

**Guidelines:** OECD 453: 87/302/EEC B.33.

**GLP:** Yes.

**Klimisch**: 1

**Study design:**

Propiconazole was administered to groups of 80 male and 80 female Sprague-Dawley CD rats in the diet at concentrations of 0 (control), 100, 500 and 2500 ppm (equivalent to an achieved intake of 0, 3.60, 18.10 and 96.46 mg/kg/day for males and 0, 4.57, 23.32 and 130.63 mg/kg/day for females) for up to approximately 2 years (107/109 weeks for males/females, respectively).

**EPA Summary**

- There were no compound-related clinical signs.
- Survival was not affected by treatment.
- Food consumption was significantly lower (p<0.001) for high dose females throughout the study and for high dose males from week 27 to termination (p<0.01).
- Body weight gains of high dose male rats were significantly lower (p<0.001; ↓16% during the first year, and ↓17% over the two year period).
- High dose female rats showed reduced body weight gain (↓35% during the first year and ↓34% during the entire two years).
- These decreases in body weight gain were compound-related.
- No toxicologically significant treatment-related effects were noted in hematology, blood
- chemistry and urinalysis parameters or in the ophthalmoscopy or hearing tests.
- No macroscopic findings in rats sacrificed at 52 weeks were considered to be related to treatment.
- Liver weights were increased in high dose animals (p<0.001; 122% and 144% of controls for males and females, respectively) at 52 weeks. Liver weights were also increased in high dose animals (p<0.001; 125% and 121% of controls for males and females, respectively) at termination.
- Necropsy observations showed an increased incidence of grossly enlarged livers among high dose males which died during the study or were sacrificed at termination (18/45 vs 6/40 in controls for males and 19/45 vs 12/28 in controls for females at termination).
- Lipid deposition in liver cells was also increased in high dose males (6/10 vs 2/10 in controls).
- An increased incidence of foci of enlarged liver cells in high dose females was reported (13/67 vs 1/67 in controls) and it concluded that to be a treatment related effect.
- Livers of high dose males showed increased vacuolated hepatocytes (44/65 vs 26/64 in controls) and ballooned cells (25/65 vs 15/64 in controls) which also exceeded historical control range suggesting a treatment-related effect.
- A dose-related increase in liver cell lipid deposition in males was also apparent (4/64, 7/67, 15/66 and 17/65 in control, low, mid and high dose groups, respectively).
- No treatment-related changes were seen in absolute or relative weights of the testes, ovaries, thyroid, adrenal or pituitary glands.
- Also, an increased incidence of discolored foci or puncta were found in the lungs of high dose females (17/45 vs 4/28 in controls at termination).
- Luminal dilatation of the uterus also appeared to be a dose-related effect (4/58, 10/63, 9/63, 17/65 in control, low, mid and high dose groups, respectively). The incidence of this finding in the 2500 ppm group exceeded both the concurrent and overall historical control values and was considered treatment-related.
- No treatment-related histopathological lesions were observed in the testes, seminal vesicle, prostate, ovaries, cervix, adrenals or pituitary glands.
- There were no treatment-related increases in the incidence of malignant tumors in treated rats.
- The incidence of dermal fibroma was increased in the high dose males (5/61 vs 0/59 in the control).
- There was an apparent increase in thyroid follicular adenocarcinoma (3/67) in high dose females vs 0/59 in controls. Additional data subsequently submitted by the registrant (Accession No. 07391829) in regard to these lesions revealed that because there was no dose-related trend in the incidences of dermal fibromas (8%) in males and thyroid follicular cell adenocarcinomas in females (2/67; 3%) at 2500 ppm and the incidences were within their respective historical range, the occurrence of these was not considered to be treatment-related.
- The LOAEL was 2500 ppm (96.4 mg/kg/day) based on liver lesions (vacuolation of hepatocytes in males, ballooned cells in the liver of males, foci of enlarged hepatocytes in females, and increased incidence of luminal dilation of the uterus) and reduced body weight gain in both males and females. The NOAEL was 500 ppm (18.1 mg/kg/day).
- The test material was not carcinogenic at the doses tested.

| **Report:** | Hunter B et al, 1982. CGA64250: Long-term feeding study in mice. Huntingdon Research Centre Ltd., Huntingdon, Cambridgeshire, UK. Laboratory Report No. CBG/196/81827, 26 October 1982. Unpublished. Acc No. 250784  Hunter B et al, 1984. CGA64250: Long term feeding study in mice (addendum to final report). Huntingdon Research Centre PLC., Huntingdon, Cambridgeshire, UK. Laboratory Report No. CBG 196/821114/2 addendum to CBG/196/81827, 15 November 1984. Unpublished. MRID No. 00129570. |
| --- | --- |

**Guidelines**: Carcinogenicity – Mouse (feeding). US EPA Guideline 83-2(B).

**GLP:** Yes.

**Klimisch**: 1

**Study design:**

Propiconazole was administered in the diet to four groups of 64 male and 64 female young adult CD-1 mice at concentrations of 0 (control), 100, 500 or 2500 ppm for up to 2 years.

**EPA Summary**

- No treatment-related clinical signs were observed.
- An increase in mortality was noted in males of the 2500 ppm group during the first 6 months. This finding is considered compound-related.
- Survival at 104 weeks for the control, 100, 500 and 2500 ppm groups was 46%, 38%, 40%, and 27% for the males and 54%, 63%, 46% and 62% for the females, respectively.
- Sporadic decreases in body weight gain, particularly in the high dose male and female groups were noted.
- Food consumption was increased in high dose male mice only.
- There were no compound-related effects on hematological parameters examined.
- SGPT and SGOT were significantly increased in high dose males and females at 52 weeks and in high dose males at 100 weeks. SAP was increased in high dose males at week 100. These changes are considered indicative of liver damage.
- Urinalysis results did not reveal any treatment-related effects.
- No treatment-related changes were seen absolute or relative weights of the testes, ovaries, thyroid, adrenal or pituitary glands.
- Increased liver weight was noted in high and mid dose males and in high dose females both at interim and terminal sacrifice.
- There was good correlation between gross and microscopic findings.
- Enlarged livers containing gross pathological changes were seen in high dose animals.
- Non-neoplastic changes in high dose males and females consisted of hepatocyte enlargement, vacuolation and fat deposition. Liver histopathology of low and mid dose mice was comparable to those of controls. Amyloidosis occurred more frequently in treated animals compared to controls, but was not dose-related. Necropsy observations at the termination of the study indicated a treatment-related increase in liver lesions (masses/raised areas/ swellings/nodular areas mainly) among mid- and high-dose males (150% and 140% of controls, respectively) and in high-dose females (367% of control).
- Propiconazole treatment was associated with early expression of malignant liver cell tumors in male mice. The incidences of malignant (presumably carcinomas) liver tumors at the one year interim sacrifice were 0/11, 0/11 1/11, and 3/9 in the control, low, mid and high dose males, respectively. No liver tumors were found in any of the female mice sacrificed at the 1-year interim sacrifice. The total incidences of combined liver adenomas/carcinomas in males for the control, 100, 500 and 2500 ppm groups were 28/64, 14/64, 25/62 and 48/64, respectively. For females the incidence was 5/64, 1/64, 2/64 and 8/64 in the control, 100, 500 and 2500 ppm groups, respectively. The combined incidence of liver tumors was statistically significant at the high dose level for males.
- No treatment-related histopathological lesions were observed in the testes, seminal vesicle, prostate, ovaries, uterus mammary glands, thyroid, adrenals or pituitary glands.
- The LOAEL was 500 ppm (49.5 mg/kg/day) based on non-neoplastic liver effects (increased liver weight in males and increase in liver lesions (masses/raised areas/ swellings/nodular areas mainly). The NOAEL was 100 ppm (10 mg/kg/day).

Of Note:

For propiconazole, a point of departure (POD) of 10.0 mg/kg/day used for risk assessment is

based on hepatotoxicity observed at 50 mg/kg/day (LOAEL) in the carcinogenicity study in

mice. This POD is based on the most sensitive endpoint (hepatotoxicity) in the most

sensitive species (mice), and is approximately 12 fold lower than the lowest dose (125

mg/kg/day) where a potentially endocrine related effect was observed in the male rats in a

published study (Goetz et al., 2007). A number of studies have shown the CAR/PXR mode of

action of propiconazole in the liver (USEPA, 2011c; Goetz and Dix, 2009; and Murphy et al.

2012). Therefore, it is possible that the limited effects on endocrine endpoints observed in the *in*

*vivo* studies are modulated by effects on the liver.

| **Report:** | Johnson W, 1985. One year subchronic oral toxicity study in Beagle dogs with CGA64250 technical. Food and Drug Research Laboratories, Inc., Route 17C, PO Box 107, Waverly, NY 14892-0107, USA. Laboratory Report No. 7737, 28 May 1985. Unpublished. MRID No. 00151515. |
| --- | --- |

**Guidelines**: US FIFRA 80-1-4, 82-1, 83-1 Draft 1982; OECD 409 and 452, 1981.

**GLP:** Yes.

**Klimisch**: 1

**Study design:**

- Propiconazole was administered in the diet to groups of 5 male and 5 female Beagle dogs for 53 consecutive weeks at dose levels of 0 (control), 5, 50 or 250 ppm (calculated to be equivalent to 0.25, 1.25 and 6.25 mg/kg/day, respectively). An additional 2 dogs/sex at 0 and 250 ppm were treated in an identical way and held for an additional 28 days as a recovery group during which time they were fed basal diet containing no test material.
- All animals were sacrificed after 52 weeks except for two males and two females of the control and 250 ppm were sacrificed after a four week recovery period.

**EPA Summary**

- All dogs survived the 12 month treatment.
- No treatment-related effects were noted in mean body weights, body weight gains, mean food consumption, hematologic and clinical chemistry, ophthalmological findings, electrocardiograms, gross pathological findings and organ weights (including testes with epididymides, ovaries, uterus, vagina, thyroid, adrenal or pituitary glands.
- Histopathological examinations revealed hyperemia of the mucosa of the stomach in 3/5 of the 250 ppm males, and no comparable findings were seen in the control males.
- Functional hypertrophy of the mammary gland was reported in 1/5 control females, 2/5 receiving 50 ppm, and 3/5 receiving 250 ppm of the test material.
- All other findings including the necropsy and histopathological examination of the dogs in the recovery period were unremarkable.
- No treatment-related histopathological lesions were observed in the testes, epididymides, prostate, ovaries, uterus, vagina, thyroid, adrenals or pituitary glands.
- The LOAEL was 250 ppm (6.25 mg/kg/day), based on hyperemia of the stomach in males (indicating mild irritation of the mucosa). The NOAEL was 50 ppm (1.25 mg/kg/day).

| **Published**  **Paper:** | Li, MH, Hansen, LG. 1996. Enzyme induction and acute endocrine effects in prepubertal female rats receiving environmental PCB/PCDF/PCDD mixtures. *Environ Health Perspect*, 104(7)712-722. |
| --- | --- |

This paper is not summarised because it did not test propiconazole.

| **Report:** | Marcsisin JF et al, 1987. CGA64250 technical: Teratology (segment II) study in rats. Research Department, Toxicology/Pathology Division, Reproductive Toxicology Subdivision, Safety Evaluation Facility, Summit, New Jersey 07901, USA. Laboratory Report No. 86004, 28 January 1987. Unpublished. MRID No. 40425001. |
| --- | --- |

**Guidelines:** EPA 83-3.

**GLP:** Yes.

**Klimisch**: 1

**Study design:**

- Propiconazole was administered to three groups of 24 pregnant Crl:COBS CD (SD)BR VAF/PLUS rats at doses of 30, 90 or 360/300 mg/kg once daily by gavage during gestational days 6-15.
- A fourth (control) group received an equivalent volume (10 mL/kg/day) of vehicle only (3% cornstarch in 0.5% Tween 80).
- On gestational day 20, all dams were sacrificed and a laparohysterectomy was performed followed by an examination of the reproductive tract and its contents.

**EPA Summary**

- One of the control dams died during the study, although there were no clinical signs in the controls. There was severe compound-related maternal toxicity observed at 360/300 mg/kg/day.
- Body weight gains were significantly decreased in the mid and high-dose groups.
- There were no significant treatment-related effects on any of the reproductive parameters examined.
- The NOEL for maternal toxicity was 30 mg/kg/day, based on reduced female body weight gains at 90 mg/kg/day.
- The NOEL for fetotoxicity was 30 mg/kg/day based on an increased incidence of unossified sternebrae and a non-statistically significant increased incidence of rudimentary ribs and shortened or absent renal papillae at 90 mg/kg/day.
- An increased incidence of cleft palate (1/302 fetuses at 90 mg/kg/day and 2/285 at 360/300 mg/kg/day) is probably compound-related but not necessarily teratogenic.

| **Abstract:** | Rieke S, Schmidt F, Heise T, Pfeil R, Niemann L, Marx-Stoelting P (2013). Triazole fungicide effects on the adrenal gland *in vivo* in a broad dose range. *Naunyn-Schmiedeberg´s Arch. Pharmacol.* **(Suppl. 1)**: S1–S10. Abstract only |
| --- | --- |

This paper is not summarised because it was only an abstract.

| **Published**  **Paper:** | Rockett JC, Narotsky MG, Thompson KE, Thillainadarajah I, Blystone CR, Goetz AK, Ren H, Best DS, Murrell RN, Nichols HP, Schmid JE, Wolf DC and Dix DJ (2006). Effect of conazole fungicides on reproductive development in the female rat. *Repro. Toxicol.* **22**: 647-658. |
| --- | --- |

**Guidelines:** None.

**GLP:** No.

**Klimisch**: 2

**Study design:**

- Groups of time mated Wistar Han rats were fed diet containing 0 (control), 100, 500 or 2500 ppm propiconazole from gestation day 6, continuing through gestation, parturition, and lactation.
- Female offspring continued to receive the test diets up through postnatal day 99.
- Pups (F1 offspring) were weighed on the day of delivery (postnatal day (PND) 0), and litters were culled to eight pups on PND 8 using blinded random selection. The selected pups were weaned on PND 22. Female weanlings were administered the same diet as their respective dams until
- termination. Dams were weighed weekly through weaning, and pups were weighed on PND 0 and weekly thereafter until termination.
- Food consumption was determined weekly through the study to determine consumed dosages of test substances.
- The following parameters were examined in the F1 rats: (1) AGD on PND 0; (2) age (days) and weight at vaginal opening (VO), with monitoring beginning on PND 29; and (3) estrous cyclicity via daily vaginal smears starting 1 day post-VO and continuing for 2 weeks, weeks 5-6 post-VO, and PND 92 to first proestrous day after PND 99 (weeks 9-10 post-VO).
- For estrous cyclicity, animals were categorized as being either proestrous, estrous, or diestrous, and estrous cyclicity for each 2-week interval tested was categorized as regular (4- or 5-day cycle), extended (3-4 days of consecutive estrous or 4-5 days of consecutive diestrous), or abnormal (>4 days of consecutive estrous or >5 days of consecutive diestrous).
- F1 rats were terminated on the first proestrous day after PND 99, and blood and organs were collected.
- The brain, hippocampus (subset), hypothalamus, liver, pituitary, thyroid, uterus, and ovaries were collected, weighed, fixed, sectioned, stained and examined for histopathology.
- The liver was scored for the severity of hepatocyte hypertrophy and vacuolation
- The thyroid was scored for colloid depletion, follicular cell hypertrophy, and/or follicular cell hyperplasia
- For ovaries, every twentieth section was examined for edema, cystic follicles, and retained oocytes.
- The concentration of estradiol in serum was measured using a commercial radioimmunoassay kit.

Results

- For dams, the overall mean dose levels for propiconazole during gestation weeks 1-2 and lactation weeks 1-3 were 12.0 mg/kg/day at 100 ppm, 56.4 mg/kg/day at 500 ppm, and 263.0 mg/kg/day at 2500 ppm.
- For pups, mean weekly dose levels generally declined from postnatal weeks 4 through 14 (PND 29 to 99). The overall mean dose levels for pups was 10.0 mg/kg/day at 100 ppm, 47.0 mg/kg/day at 500 mg/kg/day, and 236.2 mg/kg/day at 2500 ppm.
- Feed intake increased for dams through each week of gestation and lactation for all groups. A dose-related decrease in feed consumption was observed at nearly all time points, but was only significant (p<0.05) at the highest dose (↓11-24% for gestation weeks 2-3).
- For the F1 female offspring, treatment with propiconazole resulted in a dose-related decrease in feed consumption at all dose levels; significant (p<0.05) reductions were observed at the low dose for postnatal weeks 4, 6, 11, and 13 (↓9-10%; PND 29, 43, 78 and 92); at the mid dose for postnatal weeks 4, 6, 8 and 11-14 (↓9-18%; PND 29, 43, 57 and 78-99); and at the high dose for all weeks except 7, 8, and 9 (↓9-19%; PND 50, 57 and 71).
- In this study, treatment with propiconazole at the high dose significantly (p<0.05) altered estrous cyclicity only at Week 1-2 after vaginal opening (VO; treated group ~20% normal vs ~60% normal in controls). No such effect was noted in animals assessed at 5-6 or 9-10 weeks after vaginal opening.
- The authors reported significant differences at the mid and high dose when compared with controls by three outcomes (regular vs. extended vs. abnormal) and at the high dose when compared by two outcomes [(regular vs. (extended + normal)]. The effect was attributed to a high incidence of animals with abnormal cycles at the mid dose and to a high incidence of animals with extended cycles at the high dose.
- There was no correlation between anogenital distance (AGD) and VO, or between AGD and estrous cyclicity at Weeks 1-2 post-VO.
- No significant treatment-related effects were observed on litter size; gender ratio; female pup body weight; AGD at PND 0; average age at VO; body weight at PND 0, VO
- No significant treatment-related effects were noted for necropsy findings; mean relative organ weights for brain, hypothalamus, uterus, or ovaries.
- No significant treatment-related effects were noted for histopathological findings in the thyroid or ovaries
- No effects on serum estradiol levels.
- With respect to organ weights, for the high dose, liver weights were significantly (p<0.001) increased, which was attributed to hypertrophy.
- In histopathological testing, the incidence of hepatocyte hypertrophy in the livers from the high dose group (incidence of 2.6 mean lesions/group) was significantly higher (p<0.05) than controls (incidence of 0.2 mean lesions/group).

| **Report:** | Sachsse K, 1979. CGA64250: 3-Month toxicity study on dogs. Ciba-Geigy Ltd., Basle, Switzerland. Laboratory Report No. 785751, 09 August 1979. Unpublished. MRID No. 00058606. |
| --- | --- |

**Guidelines:** Predates regulatory guidelines, equivalent/similar to: OECD 409 (1998): OPPTS 870.3150 (1998): 2001/59/EC B.27 (2001).

**GLP:** This study was carried out prior to the implementation of GLP. A Statement of Data Correctness dated 19 June 1990 is included in the report.

Klimisch: 2

**Study design:**

In a subchronic toxicity study propiconazole was administered to groups of 4 male and 4 female Beagle dogs in diet at dose levels of 0, 50, 250 and 1250 ppm for up to three months.

**EPA Summary**

- In a sub-chronic toxicity study, propiconazole technical (88.0% purity) was administered to pure-bred Beagle dogs (4/sex/dose) at dietary concentrations of 0, 50, 250, or 1250 ppm for 13 weeks, which are equivalent to doses of 0, 1.25, 6.25 and 31.25 mg/kg/day, respectively.
- The initial age of the dogs was 19-28 weeks and body weights ranged from 7.9-13.0 kg for males and 6.0-11.6 kg for females.

**Results**

- Some animals of all groups including controls showed slight to moderate diarrhea during the study.
- Survival, body weight gain, food consumption, clinical chemistry, urinalysis, ophthalmic and auditory examinations and organ weights revealed no treatment related effects.
- No treatment related changes were seen in absolute or relative weights of the testes, ovaries, thyroid, adrenal, or pituitary glands
- Necropsy showed that in 3/4 of male dogs from the highest dose group (1250 ppm), slightly granular surface in the pyloric and propyloric part of the stomach was noted. Apart from this finding no gross anatomical changes were seen in treated or in control dogs.
- Microscopically, in 3 out of 4 male dogs from the highest dose-group and 1 out of 4 female dogs from the 250 ppm group, slightly increased amount of lymphoid follicles in the mucous membrane of the pyloric part of the stomach was seen. However, this was not seen in the high dose females. This histological finding was considered to be compound-related.
- No treatment-related histopathological lesions were seen in the testes, prostate, ovaries, uterus, mammary glands, thyroid, adrenal or pituitary glands.
- The LOAEL was 250 ppm (6.25 mg/kg/day) based on the finding of lymphoid follicles in the mucous membrane of the pyloric part of the stomach. The NOAEL was 50 ppm (1.25 mg/kg/day).

| **Published**  **Paper:** | Schmidt F, Rieke S, Heise T, Niemann L, Pfeil R, Marx-Stoelting P (2013). Toxic effects of triazole fungicides on rat male reproductive system in prostate gland and testis. *Naunyn-Schmiedeberg´s Arch. Pharmacol.* **(Suppl. 1)**: S1–S10. – Abstract only |
| --- | --- |

This paper is not summarised because it was only an abstract.

| **Published**  **Paper:** | Stanley, J, Sah, K, Jain, SK, Bhatt, JC, Sushil, SN. 2015. Evaluation of pesticide toxicity at their field recommended doses to honeybees, Apis cerana and A. mellifera through laboratory, semi-field and field studies. Chemosphere, 119:668-674. |
| --- | --- |

This paper reports on contact toxicity to 2 species of bees for many pesticides reportedly at field relevant rates. There were no effects of concern for propiconazole. The paper was not relevant to this exercise and was not reviewed.

| **Published**  **Paper:** | Taxvig C et al, 2008. Endocrine-disrupting properties *in vivo* of widely used azole fungicides. *Int. J. Andrology* 31: 170-177. |
| --- | --- |

**Guidelines:** None.

**GLP:** No.

**Klimisch**: 2 (but not relevant for this exercise)

**Study design:**

Time-mated, young adult female Wistar rats were dosed by oral gavage with vehicle (corn oil) or 50 mg/kg/day propiconazole (in addition to other azole fungicides) from gestation day (GD) 7 to GD 21.

**Results:**

- This was a mixture study so it is not relevant for this exercise.

| **Published**  **Paper:** | Wolf, DC, Allen, JW, George, MH, Hester SD, Sun, GB, Moore, T, Thai, SF, Delker, D, Winkfield, E, Leavitt, S, Nelson, G, Roop, BC, Jones, C, Thibodeaux, J, Nesnow, S. 2006, Toxicity profiles in rats treated with tumorigenic and non-tumorigenic triazole conazole fungicides: Propiconazole, triadimefon, and myclobutanil. *Toxicol Pathol*, 34(7): 895-902. |
| --- | --- |

**Guidelines:** None.

**GLP:** No.

**Klimisch**: 2

**Study design:**

- Approximately 7-week old male Wistar/Han rats (13/dose group/time point) were treated orally with propiconazole (94.2% purity) in their diet at 0, 100, 500, or 2500 ppm (0, 5.5, 26.2, and 128.5 mg/kg/day) for 4, 30, or 90 days.
- At interim time points and the end of the experiment, rats were euthanized and necropsied.
- Blood was collected and serum separated and stored at -80°C for hormone and other biochemical analyses.
- Brain, pituitary, thyroid, liver, kidney, and testicles were examined macroscopically, fixed and frozen (-80°C) for further analyses including histopathology, biochemistry, or gene expression. Liver weights were recorded at necropsy.
- Quantitative assessment was made of thyroid and liver histopathology.
- The liver alterations were scored based on severity of hepatocyte hypertrophy.
- The thyroid gland was scored for colloid depletion, follicular cell hypertrophy, and/or follicular cell hyperplasia.
- In addition to lesion scores, cell proliferation indices were quantified for hepatocytes and thyroid follicular cells. The tissues were stained for the presence of proliferating cell nuclear antigen (PCNA). The number of labeled nuclei per approximately 1,000 total nuclei was identified for each organ to calculate a labeling index.
- Total circulating serum thyroid hormone levels of T3, T4, and TSH were measured.
- Serum levels for cholesterol, high density lipoproteins, and triglycerides were measured.
- Liver microsomes were prepared, and cytochrome P450 enzyme activities were assessed with alkoxyresorufin Odealkylation (AROD) assays. These assays were based on activity measures of ethoxyresorufin O-dealkylation (EROD), pentoxyresorufin O-dealkylation (PROD), and methoxyresorufin O-dealkylation (MROD). AROD activities were expressed at rates of resorufin formation, and were calculated based on the fluorescence of a standard curve of resorufin.
- Uridine diphosphoglucuronosyl transferase (UDPGT) was assayed using 1-naphthol as a substrate and continuously monitoring the formation of 1-naphthyl-β-D-glucuronide using a fluorometer.

**EPA Summary**

A study by **Wolf *et al.* (2006)** was designed to characterize the time- and dose-dependent thyroid

alterations induced by propiconazole.

Effects

- All rats survived to the appointed termination and no significant body weight changes were observed across treatments.
- The principle effects noted in the study were on the liver. At the high dose, absolute and relative liver weight was increase as was the incidences of hepatocellular hypertrophy.
- Rat liver cell proliferation was increased (p<0.05) at 2500 ppm after 4 days by 6.6-fold.
- Liver microsomal activities were increased (p<0.05) as follows: (i) EROD at 2500 ppm after 4 and 30 days (3.7- to 4.2-fold induction); (ii) EROD at 500 ppm after 4 days (3.2-fold induction); (iii) PROD at 500 and 2500 ppm after 4, 30, and 90 days (10.6- to 25-fold induction); (iv) PROD at 100 ppm after 4 days (2.8-fold induction); (v) MROD at 2500 ppm after 4, 30, and 90 days (1.8- to 8.1-fold induction); and (vi) MROD at 500 ppm after 4 and 30 days (2.0- to 3.6-fold induction).
- There was a time-dependent increase of cytochrome P450 metabolizing activity based on the PROD assays.
- Elevated UDPGT activity was noted after 4, 30, and 90 days.
- At 2500 ppm, both cholesterol and HDL were increased (p<0.05) by 25% after 4 days.
- Propiconazole had no effect on thyroid histology at up to 2500 ppm, although a decrease in cell proliferation was noted at the 30-day interval at the high dose.
- Sporadic changes in levels of thyroid hormone were also noted, but the effects were generally not dose-dependent. T4 levels were decreased by 12 and 20% at the mid and high dose on Day 4 and by 16% at the high dose on Day 30, but no changes were noted on Day 90. T3 levels were not affected on Day 4 or 90, but were decreased at all three doses by 20-30% on Day 30. TSH levels were decreased by 38 and 50% at the low and high dose on Day 3, but no effects were noted on Days 30 or 90..
- There was a dose-dependent increase in liver weight but not body weight for all treatments.
- The indication of cytochrome induction (PROD activity) had a dose-related increase at each time point.
- UDPGT, the T4 metabolizing enzyme measured as glucuronidation of 1-naphthol, was
- induced to a similar extent after 30 and 90 days.
- Livers from all high dose treated rats had centrilobular hepatocyte hypertrophy after 4 days, and propiconazole treated rats had hepatocyte hypertrophy after 30 days.
- A dose-dependent decrease in T4 was present after 4 days and 30 days.
- Thyroid hormone levels did not differ from control values after 90 days, and TSH was not increased in any exposure group.

These data demonstrate that propiconazole can modulate hepatic enzyme activity and can effect thyroid hormone concentrations (up to Day 30).

The effects on liver indicate that the any thyroid effect may be secondary to effects on liver (enzyme induction).

Overt toxicity was not observed in the study by Wolf *et al.* (2006) examining propiconazole

effects on liver function and thyroid hormones at dietary dose up to 2500 ppm (128.5

mg/kg/day). Following 90 days of dosing, no significant changes were noted in body weights,

but both absolute and relative liver weights were increased by 24 and 21%, respectively, at the

high dose. In addition, the incidence and severity of hepatocyte hypertrophy were increased after

4 and 30 days of dosing.

| **Published**  **Paper:** | Hancock, HG, Weete, JD. 1985. Effects of triazoles on fungi. IV. Growth and lipids of cercospora-arachidicola and cercosporidium-personatum. *Pestic. Biochem Physiol* 24(3):395-405. |
| --- | --- |

This study concerns mode of action of propiconazole in fungi. It is not relevant to this exercise and was not reviewed.

| **Published**  **Paper:** | Johansen, NS, Moen, LH, Egaas, E. 2007. Sterol demethylation inhibitor fungicides as disruptors of insect development and inducers of glutathione S-transferase activities in Mamestra brassicae. *Comp Biochem Physiol C: Toxicol Pharmacol*, 145(3):473-483 |
| --- | --- |

This paper measures apical (growth, development and reproduction) endpoints in cabbage moth after exposure to propiconazole. The paper is not relevant to this exercise and was not reviewed.

| **Published**  **Paper:** | Kone, D, Badou, OJ, Bomisso, EL, Camara, B, Ake, S. 2009. In vitro activity of different fungicides on the growth in Mycosphaerella fijiensis var. difformis Stover and Dickson, Cladosporium musae Morelet et Deightoniella torulosa (Syd.) Ellis, isolated parasites of the banana phyllosphere in the Ivoery Coast. *Comptes Rendus Biologies*, 332(5):448-455. |
| --- | --- |

This paper reports on propiconazole efficacy in fungi pests. It is in French with an abridged English summary. The paper is not relevant to this exercise and was not reviewed.

| **Published**  **Paper:** | Pilling, ED, Jepson PC. 1993. Synergism between EBI fungicides and a pyrethroid insecticide in the honeybee (Apis mellifera). *Pesticide Science* 39(4):293-297. |
| --- | --- |

This paper reports bee mortality data of propiconazole in combination with lambda-cyhalothrin. It is not relevant to this exercise and was not reviewed.

| **Published**  **Paper:** | Ronis, MJJ, Celander, M, Badger, TM. 1998. Cytochrome P450 enzymes in the kidney of the bobwhite quail (*Colinus virginianus*): induction and inhibition by ergosterol biosynthesis inhibiting fungicides. *Comp Biochem Physiol C: Pharmacol Toxicol Endocrinol*, 121(1-3):221-229. |
| --- | --- |

This paper reports on a study comparing metabolic capacity of quail liver and kidney microsomes with that of the rat.
